# Enantioselective synthesis of chiral porphyrin macrocyclic hosts and kinetic enantiorecognition of viologen guests†

**DOI:** 10.1039/d0sc05233g

**Published:** 2021-01-13

**Authors:** Pieter J. Gilissen, Annemiek D. Slootbeek, Jiangkun Ouyang, Nicolas Vanthuyne, Rob Bakker, Johannes A. A. W. Elemans, Roeland J. M. Nolte

**Affiliations:** Institute for Molecules and Materials, Radboud University Heyendaalseweg 135 6525 AJ Nijmegen The Netherlands r.nolte@science.ru.nl j.elemans@science.ru.nl; Aix Marseille Univ, CNRS, Centrale Marseille, iSm2 Marseille France

## Abstract

The construction of macromolecular hosts that are able to thread chiral guests in a stereoselective fashion is a big challenge. We herein describe the asymmetric synthesis of two enantiomeric *C*_2_-symmetric porphyrin macrocyclic hosts that thread and bind different viologen guests. Time-resolved fluorescence studies show that these hosts display a factor 3 kinetic preference (ΔΔ*G*^‡^_on_ = 3 kJ mol^−1^) for threading onto the different enantiomers of a viologen guest appended with bulky chiral 1-phenylethoxy termini. A smaller kinetic selectivity (ΔΔ*G*^‡^_on_ = 1 kJ mol^−1^) is observed for viologens equipped with small chiral *sec*-butoxy termini. Kinetic selectivity is absent when the *C*_2_-symmetric hosts are threaded onto chiral viologens appended with chiral tails in which the chiral moieties are located in the centers of the chains, rather than at the chain termini. The reason is that the termini of the latter guests, which engage in the initial stages of the threading process (entron effect), cannot discriminate because they are achiral, in contrast to the chiral termini of the former guests. Finally, our experiments show that the threading and de-threading rates are balanced in such a way that the observed binding constants are highly similar for all the investigated host–guest complexes, *i.e.* there is no thermodynamic selectivity.

## Abstract 2

Chiral guests display kinetic stereoselective threading through chiral porphyrin cages if their chirality is located at the chain ends and not in the centers, supporting the previously reported entron effect of threading.

## Graphical abstract

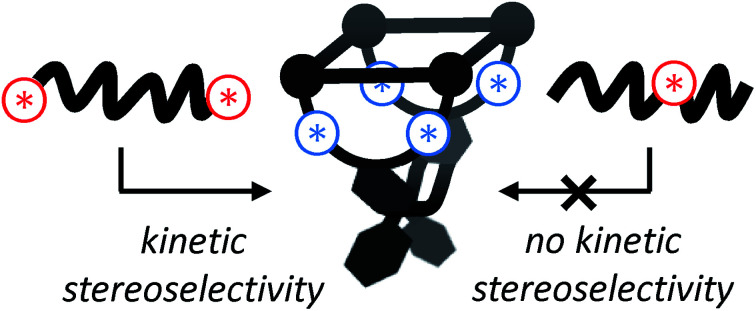

## Introduction

Chiral recognition and selection of substrate (guest) molecules are well-established features of enzymes and natural receptors.^1,2^ Inspired by these naturally occurring chiral hosts, chemists have developed a variety of synthetic chiral macromolecular architectures that function as receptors. Examples include macrocyclic arenes,^3–8^ cyclodextrins,^9^ and metal–organic cages.^10–13^ Calixarenes have been reported that display enantiorecognition towards chiral carboxylates^3^ and chiral amines.^4–6^ Amino acid recognition has been achieved with calixarenes,^4^ cyclodextrins,^9^ and metal–organic cages.^10^ Chiral metal–organic cages have also been used for the enantioseparation of small alcohols and carboxylic acids,^11,12^ and for the separation of atropisomeric compounds, such as 1,1′-bi-2-naphthol (BINOL) derivatives.^11^ Triptycene-based hosts have been developed for the recognition of chiral trimethylammonium compounds.^14^ The reported receptors showed different degrees of thermodynamic enantiorecognition (up to 12-fold difference in binding constant *K*_assoc_ for the binding of the two enantiomers). Studies on kinetic enantiorecognition, such as differences in threading rate constants (*k*_on_-values), however, have rarely been reported.^15^

**Fig. 1:**
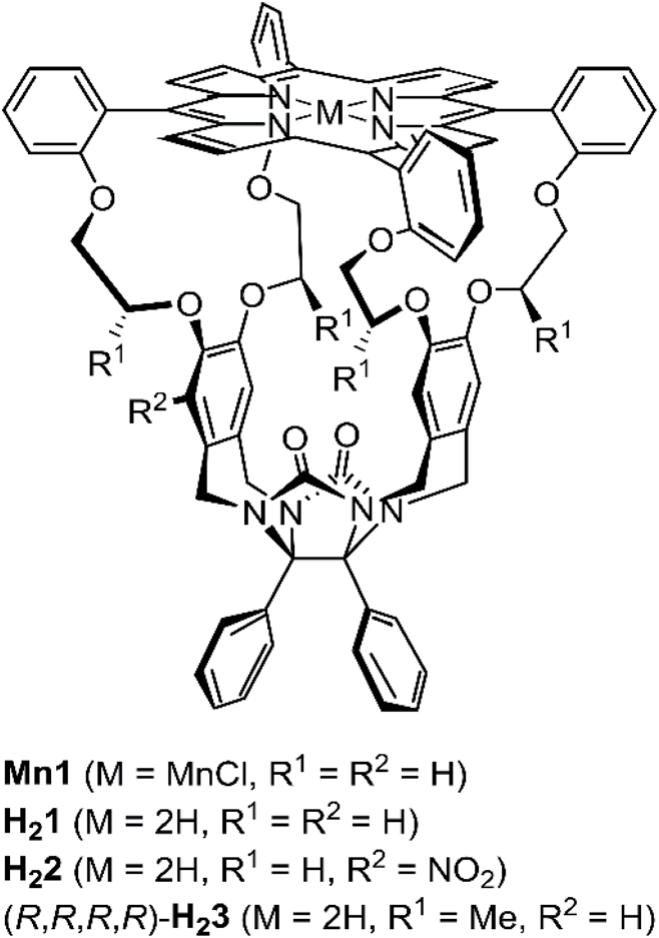
Chemical structures of porphyrin macrocycles.

Our research aims at encoding information into synthetic polymers using a macrocyclic porphyrin catalyst based on the glycoluril framework, *i.e.***Mn1** (Fig. 1) that can thread onto a polymer containing alkene double bonds, *e.g.* a polybutadiene chain, and processively epoxidize these double bonds.^16–18^ Since we intend to write a binary code on said polymer in the form of chiral epoxides ((*R*,*R*)-epoxide = digit 0, (*S*,*S*)-epoxide = digit 1), our desired writing system requires a chiral variant of **Mn1** that is capable of performing sequential processive threading and catalysis. Two important requirements for sequential processive catalysis are spatiotemporal control and unidirectionality,^19^ both of which are determined by kinetic factors. We hypothesized that unidirectional threading of polymer chain through a catalytic machine might be accomplished by aligning chiral structural information in both the catalyst and the polymer. Therefore, it is crucial that the catalytic machine displays a kinetic preference for one of the enantiomers of a chiral (polymeric) guest. In earlier work, we reported on the post-modification of the achiral porphyrin cage **H21**^20^ by providing it with a nitro function, yielding a racemic mixture of planar chiral nitro-functionalized porphyrin cage **H22**.^21^ The enantiomers of **H22** could be resolved by chiral HPLC^22^ and by installing a chiral auxiliary on the corresponding amino-functionalized host.^23^ The enantiopure hosts investigated earlier were shown to have small differences in affinity for chiral viologen guests (up to 3-fold difference in binding constant *K*_assoc_).^22,23^ In this paper we report an alternative approach to synthesize a chiral *C*_2_-symmetric porphyrin cage compound, *i.e.* a host in which the porphyrin macrocycle is linked to the glycoluril framework *via* chiral linkers (**H23**, Fig. 1). Furthermore, we show that the enantiomers of **H23** display a kinetic preference for the threading and binding of viologen guests equipped with a chiral head group.

## Results and discussion

### Synthesis and characterization

**Fig. 2:**
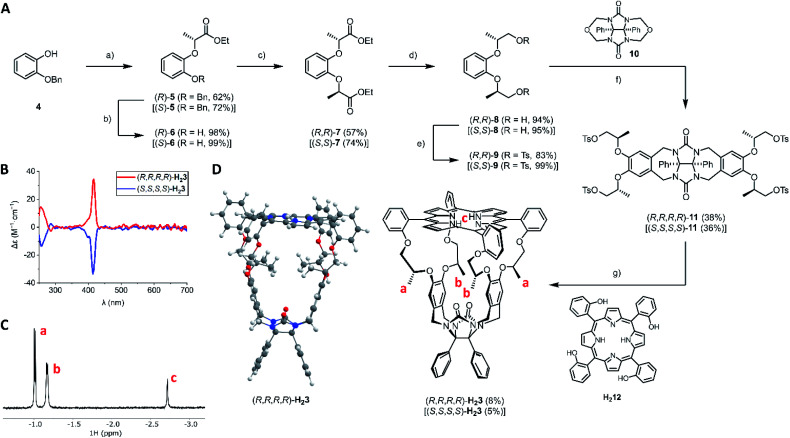
Synthesis and characterization of **H23**. (A) Synthesis of **H23**; reagents and conditions: (a) ethyl (*S*)-lactate (for (*R*)-**5**) or ethyl (*R*)-lactate (for (*S*)-**5**), DIAD, PPh_3_, toluene, 0 → 20 °C; (b) H_2_, Pd/C, EtOAc, 20 °C; (c) ethyl (*S*)-lactate (for (*R*,*R*)-**7**) or ethyl (*R*)-lactate (for (*S*,*S*)-**7**), DIAD, PPh_3_, toluene, 0 → 20 °C; (d) LiAlH_4_, THF, 0 → 20 °C; (e) TsCl, pyridine, CH_2_Cl_2_, 0 → 20 °C; (f) ZnCl_2_, SOCl_2_, CH_2_Cl_2_, 20 °C; (g) K_2_CO_3_, CH_3_CN, reflux. For clarity, the synthetic scheme only displays the structures of all (*R*)-derivatives; yields of all (*S*)-derivatives are indicated in square brackets. (B) ECD spectra of (*R*,*R*,*R*,*R*)-**H23** and (*S*,*S*,*S*,*S*)-**H23** (*c* = 3 × 10^−5^ M in CHCl_3_/CH_3_CN, 1 : 1, v/v). (C) Negative chemical shift region of the ^1^H NMR spectrum of (*R*,*R*,*R*,*R*)-**H23** (400 MHz, *c* = 10^−3^ M in CDCl_3_/CD_3_CN, 1 : 1, v/v) with assignment of protons **a–c**. (D) Front view of the DFT-optimized structure of (*R*,*R*,*R*,*R*)-**H23** at the B3LYP/6-311+G(d) level. Color code: white = hydrogen, grey = carbon, blue = nitrogen, red = oxygen.

The synthetic route to the novel porphyrin macrocyclic hosts (*R*,*R*,*R*,*R*)-**H23** and (*S*,*S*,*S*,*S*)-**H23** is depicted in Fig. 2. The key synthesis step was the introduction of the chiral centers in intermediate **7**. Burkard and Effenberger reported the synthesis of (*R*,*R*)-**7** in one step from pyrocatechol and the very reactive triflate ester of ethyl (*S*)-lactate under basic conditions.^24^ According to their communicated procedure, the resulting diester (*R*,*R*)-**7** was obtained diastereomerically pure. In our hands, however, this procedure was not scalable and it resulted in an inseparable 94 : 6 diastereomeric mixture of (*R*,*R*)-**7** and the *meso*-compound (*R*,*S*)-**7** (*via* epimerization of one of the chiral centers). To prevent epimerization, we resided to the Mitsunobu reaction. Initially, we attempted a double Mitsunobu reaction on pyrocatechol with ethyl (*S*)-lactate to afford diester (*R*,*R*)-**7** in a single step, which however failed. In contrast to the lack of literature procedures for Mitsunobu reactions on pyrocatechol, there are successful examples of Mitsunobu reactions on 2-alkoxyphenols employing enantiopure lactates.^25–27^

Hence, we designed a stepwise protocol for the introduction of the chiral centers. The Mitsunobu reaction of 2-benzyloxyphenol with ethyl (*S*)-lactate or ethyl (*R*)-lactate afforded esters (*R*)-**5** and (*S*)-**5** in 62% and 72% yield, respectively. After quantitative removal of the benzyl protecting group, the other chiral center was installed with a second Mitsunobu reaction. In this way, diesters (*R*,*R*)-**7** and (*S*,*S*)-**7** were obtained in reasonable yields (57% and 74%, respectively) and with excellent diastereomeric ratios (>98 : 2). Both diesters **7** were reduced with lithium aluminum hydride, to afford the corresponding primary alcohols **8** in excellent yields. The primary alcohols were then transformed into tosylate leaving groups, affording (*R*,*R*)-**9** (83%) and (*S*,*S*)-**9** (99%). According to the established procedure for the synthesis of achiral cage compound **H21**,^28^ the *N*,*O*-acetals of compound **10** were activated with zinc chloride in the presence of thionyl chloride, and the resulting *N*-acyliminium intermediates were reacted with electron-rich arenes (*R*,*R*)-**9** and (*S*,*S*)-**9** to afford chiral clip molecules (*R*,*R*,*R*,*R*)-**11** (38%) and (*S*,*S*,*S*,*S*)-**11** (36%). The syntheses of the two enantiomers of **H23** were completed by reacting chiral clips **11** under highly dilute basic conditions with 1 equivalent of porphyrin tetraol **H212**. After two chromatographic purification steps (alumina followed by 60H silica), the enantiopure cage compounds (*R*,*R*,*R*,*R*)-**H23** (8%) and (*S*,*S*,*S*,*S*)-**H23** (5%) were obtained as purple solids. Electronic circular dichroism (ECD) measurements (Fig. 2B) showed that the chirality of the flexible spacers is clearly transferred to the porphyrin, as opposite signs of the CD signals were observed for the Soret bands (*λ*_max_ = 416 nm) of the enantiomeric hosts (*R*,*R*,*R*,*R*)-**H23** and (*S*,*S*,*S*,*S*)-**H23**. ^1^H NMR analysis (Fig. 2C) revealed that the methyl groups of the *C*_2_-symmetric macrocycles are in uncommon – highly shielded – chemical environments, as their protons resonate at negative chemical shift (assigned as protons **a** and **b**). This effect can be explained by the close proximity of the shielding area of the aromatic porphyrin ring, indicating that the methyl groups are pointing into the cavity of the cage. 2D ROESY experiments indicated the close proximity of the methyl groups **a** and **b**, as we observed mutual NOE contacts. DFT calculations (Fig. 2D) confirmed that the minimum energy structure of **H23** is the one with the methyl groups pointing into the cavity of the porphyrin macrocyclic host.

### Host–guest binding and threading studies

**Fig. 3:**
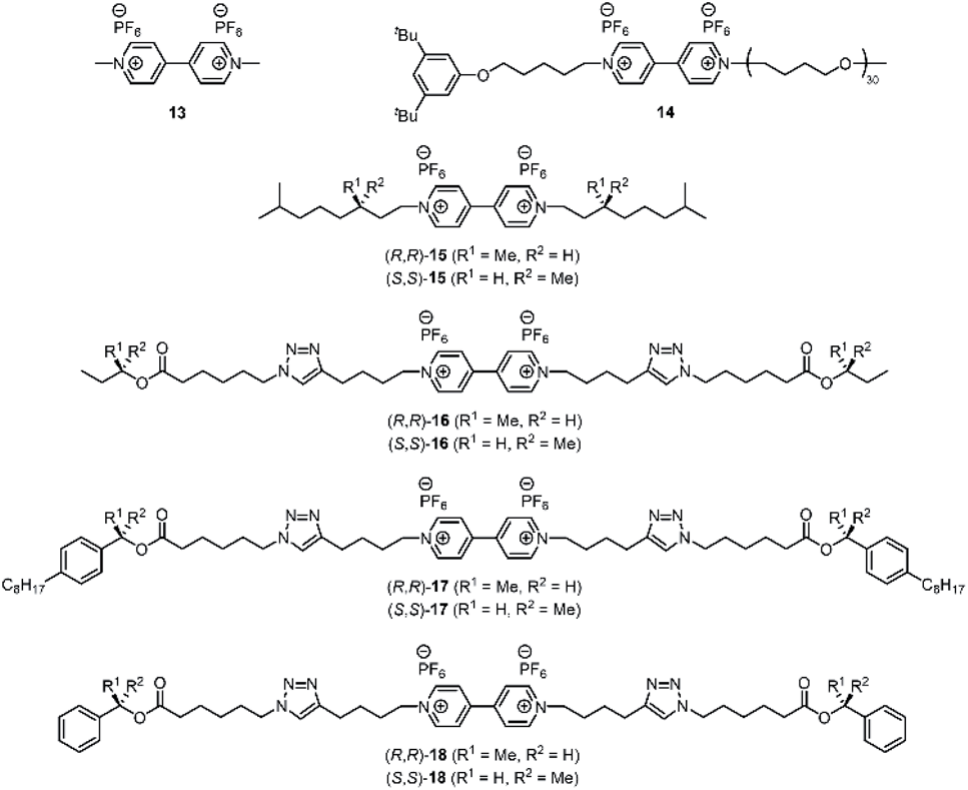
Chemical structures of viologen guests **13–18**.

**Fig. 4:**
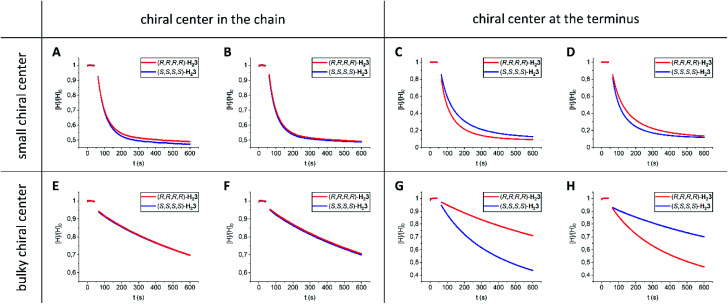
Threading of porphyrin macrocyclic hosts **H23** onto chiral viologens **15–18** in CHCl_3_/CH_3_CN (1 : 1, v/v) at 298 K. Normalized fluorescence intensity of hosts **H23** as a function of time after the addition (*t* = 50 s) of 1 equivalent of guest (A) (*R*,*R*)-**15** (*c* = 2.5 × 10^−7^ M), (B) (*S*,*S*)-**15** (*c* = 2.5 × 10^−7^ M), (C) (*R*,*R*)-**16** (*c* = 3 × 10^−6^ M), (D) (*S*,*S*)-**16** (*c* = 3 × 10^−6^ M), (E) (*R*,*R*)-**17** (*c* = 10^−5^ M), (F) (*S*,*S*)-**17** (*c* = 10^−5^ M), (G) (*R*,*R*)-**18** (*c* = 10^−5^ M), and (H) (*S*,*S*)-**18** (*c* = 10^−5^ M).

We reported earlier that **H21** is an excellent host for the binding of viologen derivatives.^20^ Hence, we investigated the effect of the chiral spacers in the *C*_2_-symmetric hosts **H23** on the binding thermodynamics and the threading kinetics (Table 1) of various achiral and chiral viologen guests (Fig. 3). We studied threading by using approach-to-equilibrium fluorescence quenching spectroscopy, in which we follow the fluorescence intensity of the host as a function of time, as it is quenched upon binding the viologen moiety (Fig. 4, for details see the ESI, Fig. S8–S55†). The initial stages of this process follow second-order kinetics.^29,30^ Subsequently, de-threading of the viologen guests from the hosts was studied by dilution-induced fluorescence recovery, for which the initial stages follow first-order kinetics (see the ESI, Fig. S56–S103†).^29,30^ Binding constants were obtained by dividing the threading *k*_on_-values by the de-threading *k*_off_-values. For the smallest possible viologen, *i.e.* methyl viologen **13**, the kinetics of threading and de-threading were too fast to measure, and therefore the binding constants of this guest in the enantiomers of host **H23** were obtained by fluorescence titrations (see the ESI, Tables S4–S6†). The obtained binding constants with guest **13** were slightly higher than the binding constant between **13** and the parent porphyrin macrocyclic host **H21** (Table 1, entries 1–3).^20^ This result implies that the methyl groups, which are inside the cavity, do not have a negative effect on the thermodynamics of the binding process. In fact, a ^1^H NMR titration of (*S*,*S*,*S*,*S*)-**H23** with viologen **13** showed a remarkably large effect on the methyl protons **a** and **b** of the chiral spacers (see the ESI, Fig. S104†). Upon binding the guest, the signals of these protons were deshielded by ∼2 ppm units, indicating that the methyl groups orient themselves out of the cavity, thereby facilitating binding of the guest inside the cavity (see the ESI, Fig. S105†). Apparently, this structural reorganization of the host has no negative effect on the binding strength of **13**. An explanation for the slightly enhanced binding may be the presence of stabilizing van der Waals interactions between the bound guest and the spacer methyl groups of the host.

**Table 1:** Kinetic and thermodynamic data for porphyrin macrocyclic host–guest complexes acquired by fluorescence spectroscopy in CHCl_3_/CH_3_CN (1 : 1, v/v) at 298 K

Entry	Guest	Host	*k*_on_ d (M^−1^ s^−1^)	Δ*G*^‡^_on_ (kJ mol^−1^)	ΔΔ*G*^‡^_on_ (kJ mol^−1^)	*k*_off_ d (s^−1^)	Δ*G*^‡^_off_ (kJ mol^−1^)	*K*_assoc_ e (M^−1^)	Δ*G*_assoc_ (kJ mol^−1^)
1^a^	**13**	**H21**	—	—	—	—	—	6.0 × 10^5^	−33.0
2^b^	**13**	(*R*,*R*,*R*,*R*)-**H23**	—	—	—	—	—	1.9 × 10^6^	−35.8
3^b^	**13**	(*S*,*S*,*S*,*S*)-**H23**	—	—	—	—	—	1.6 × 10^6^	−35.4
4^c^	**14**	**H21**	4.0 × 10^4^	46.7	—	1.4 × 10^−3^	89.3	2.9 × 10^7^	−42.5
5	**14**	(*R*,*R*,*R*,*R*)-**H23**	2.0 × 10^3^	54.1	+0.0	1.3 × 10^−4^	95.2	1.5 × 10^7^	−41.0
6	**14**	(*S*,*S*,*S*,*S*)-**H23**	2.0 × 10^3^	54.1		1.2 × 10^−4^	95.3	1.7 × 10^7^	−41.2
7^b^	(*R*,*R*)-**15**	**H21**	—	—	—	—	—	8.6 × 10^6^	−39.6
8	(*R*,*R*)-**15**	(*R*,*R*,*R*,*R*)-**H23**	4.2 × 10^4^	46.6	+0.1	4.0 × 10^−3^	86.7	1.1 × 10^7^	−40.1
9	(*R*,*R*)-**15**	(*S*,*S*,*S*,*S*)-**H23**	4.4 × 10^4^	46.5		3.9 × 10^−3^	86.7	1.1 × 10^7^	−40.2
10^b^	(*S*,*S*)-**15**	**H21**	—	—	—	—	—	1.1 × 10^7^	−40.1
11	(*S*,*S*)-**15**	(*R*,*R*,*R*,*R*)-**H23**	4.4 × 10^4^	46.5	+0.1	3.7 × 10^−3^	86.9	1.2 × 10^7^	−40.4
12	(*S*,*S*)-**15**	(*S*,*S*,*S*,*S*)-**H23**	4.5 × 10^4^	46.4		4.1 × 10^−3^	86.6	1.1 × 10^7^	−40.2
13	(*R*,*R*)-**16**	**H21**	3.6 × 10^4^	47.0	—	2.3 × 10^−3^	88.0	1.5 × 10^7^	−41.0
14	(*R*,*R*)-**16**	(*R*,*R*,*R*,*R*)-**H23**	7.8 × 10^3^	50.8	−1.1	4.7 × 10^−4^	92.0	1.7 × 10^7^	−41.2
15	(*R*,*R*)-**16**	(*S*,*S*,*S*,*S*)-**H23**	4.9 × 10^3^	51.9		3.7 × 10^−4^	92.6	1.3 × 10^7^	−40.6
16	(*S*,*S*)-**16**	**H21**	3.7 × 10^4^	46.9	—	2.4 × 10^−3^	87.9	1.5 × 10^7^	−41.0
17	(*S*,*S*)-**16**	(*R*,*R*,*R*,*R*)-**H23**	4.7 × 10^3^	52.0	+1.0	3.4 × 10^−4^	92.8	1.4 × 10^7^	−40.8
18	(*S*,*S*)-**16**	(*S*,*S*,*S*,*S*)-**H23**	7.0 × 10^3^	51.0		4.4 × 10^−4^	92.1	1.6 × 10^7^	−41.1
19	(*R*,*R*)-**17**	**H21**	1.8 × 10^3^	54.4	—	1.5 × 10^−4^	94.9	1.2 × 10^7^	−40.4
20	(*R*,*R*)-**17**	(*R*,*R*,*R*,*R*)-**H23**	7.2 × 10^1^	62.4	+0.0	3.1 × 10^−5^	98.7	2.3 × 10^6^	−36.3
21	(*R*,*R*)-**17**	(*S*,*S*,*S*,*S*)-**H23**	7.1 × 10^1^	62.4		2.9 × 10^−5^	98.9	2.5 × 10^6^	−36.5
22	(*S*,*S*)-**17**	**H21**	1.9 × 10^3^	54.3	—	1.4 × 10^−4^	94.9	1.3 × 10^7^	−40.6
23	(*S*,*S*)-**17**	(*R*,*R*,*R*,*R*)-**H23**	7.1 × 10^1^	62.4	+0.0	2.8 × 10^−5^	99.0	2.5 × 10^6^	−36.5
24	(*S*,*S*)-**17**	(*S*,*S*,*S*,*S*)-**H23**	7.2 × 10^1^	62.4		3.0 × 10^−5^	98.8	2.4 × 10^6^	−36.4
25	(*R*,*R*)-**18**	**H21**	4.5 × 10^3^	52.1	—	2.6 × 10^−4^	93.4	1.7 × 10^7^	−41.3
26	(*R*,*R*)-**18**	(*R*,*R*,*R*,*R*)-**H23**	7.2 × 10^1^	62.4	+3.0	1.1 × 10^−5^	101.3	6.6 × 10^6^	−38.9
27	(*R*,*R*)-**18**	(*S*,*S*,*S*,*S*)-**H23**	2.5 × 10^2^	59.3		2.3 × 10^−5^	99.4	1.1 × 10^7^	−40.1
28	(*S*,*S*)-**18**	**H21**	5.1 × 10^3^	51.8	—	2.4 × 10^−4^	93.6	2.1 × 10^7^	−41.8
29	(*S*,*S*)-**18**	(*R*,*R*,*R*,*R*)-**H23**	2.2 × 10^2^	59.6	−2.9	2.4 × 10^−5^	99.3	9.2 × 10^6^	−39.7
30	(*S*,*S*)-**18**	(*S*,*S*,*S*,*S*)-**H23**	6.7 × 10^1^	62.6		1.4 × 10^−5^	100.7	4.8 × 10^6^	−38.1

a Values taken from ref. 20.

b Determined by fluorescence titration.

c Values taken from ref. 30.

d Estimated error 20%.

e *K*_assoc_ = *k*_on_/*k*_off_, unless stated otherwise, estimated error 30%. See the ESI for details.

We then studied the threading of achiral polymer **14** through macrocyclic hosts **H21**^30^ and **H23** (Table 1, entries 4–6). The *k*_on_-value for the threading of polymer **14** through **H21** was 20 times higher than that for the threading through the two enantiomers of **H23**. In addition, the *k*_off_-value for the de-threading of polymer **14** from **H21** was 14 times higher than that for the de-threading of **14** from the enantiomers of **H23**. Even though the kinetic processes of both threading through and de-threading from **H21** are a lot faster compared to those determined for **H23**, the on- and off-rates are balanced, resulting in similar *K*_assoc_ values. The NMR spectra of the host–guest complexes of **H23** with **14** again showed deshielding of methyl protons **a** and **b** by ∼2 ppm units (see the ESI, Fig. S106†).

To investigate whether enantiomeric viologens with chiral *N*-substituents would exhibit stereoselective threading behavior through the cavities of the enantiomers of **H23**, we examined the threading and de-threading processes of chiral viologens (*R*,*R*)-**15** and (*S*,*S*)-**15** (Table 1, entries 7–12).^23^ The threading experiments (Fig. 4A and B) revealed that the enantiomers of **H23** display no kinetic preference for the binding of either (*R*,*R*)-**15** or (*S*,*S*)-**15**. Also the de-threading (see the ESI, Fig. S60–S67†) of these guests from both enantiomers of **H23** occurred at the same rate. As a consequence, there is no thermodynamic preference for the binding of the chiral guests. The binding constants of all complexes **H23·15** were verified independently by fluorescence titrations and the values were similar to those of **H21·15** (see the ESI, Tables S7–S13†). The ^1^H NMR spectra of all combinations of host–guest complexes **H23·15** were nearly fully superimposable (see the ESI, Fig. S107†). The spectra again showed deshielding of the methyl groups of the chiral spacers of the hosts, as we also observed for the host–guest complexes of **H23** with achiral guests. We propose that the lack of kinetic selectivity of chiral hosts **H23** for threading onto chiral guests **15** arises from the absence of chirality at the termini of the viologen substituents, *i.e.* the part of the guest molecule that has to initiate the threading process. Previously, we have shown that the threading of polymeric viologens through **H21** involves a kinetically favorable ‘entron’ effect, which involves an internal filling of the cavity of the porphyrin macrocyclic host by the first 5–8 atoms of the polymer chain.^30^ Starting from the chain termini, the stereogenic centers of viologens **15** only appear at the 6^th^ chain atom. We hypothesize that the remote location of the chirality in the chain inhibits the kinetic stereoselectivity of the threading process, and that the initial entron effect is only governed by the threading of the achiral isopropyl termini of the chains of **15**, which are identical for both enantiomers. Alternatively, the lack of stereoselectivity could be due to the lack of steric bulk near the chiral centers of guest **15**.

To investigate these hypotheses further, we designed and synthesized chiral guest molecules **16–18** (see the ESI, Scheme S1†). Guest **16** contains small *sec*-butoxy chiral groups at the chain termini and guest **17** contains bulky 1-(4-octylphenyl)ethoxy chiral groups in the center of the chains. Guest **18** contains a viologen binding station appended at the chain termini with chiral 1-phenylethoxy moieties.

We found that guests **16** (Table 1, entries 13–18) display a kinetic preference of a factor 1.6 (ΔΔ*G*^‡^_on_ = 1 kJ mol^−1^) for threading through the enantiomers of **H23**, *i.e.* (*R*,*R*)-**16** prefers (*R*,*R*,*R*,*R*)-**H23** over (*S*,*S*,*S*,*S*)-**H23** and (*S*,*S*)-**16** prefers (*S*,*S*,*S*,*S*)-**H23** over (*R*,*R*,*R*,*R*)-**H23** (Fig. 4C and D). The small selectivity is attributed to the location of the small chiral moieties at the termini of the guests.

Then, we investigated guests **17** (Table 1, entries 19–24), which contain bulky chiral moieties in locations remote from the chain termini. We found that threading of hosts **H23** onto guests **17** proceeded very slowly, *i.e.* 1–3 orders of magnitude slower than measured for the other investigated guests. This deceleration is caused by the steric bulk in the chains of the guests. The threading experiments (Fig. 4E and F) also indicated that guests **17** do not display kinetic selectivity, which is line with the experiments involving guests **15**. Hence, for such selectivity the location of the chiral moiety at the terminus of the guest is pivotal.

In a final set of experiments, we investigated the threading of guests **18** (Table 1, entries 25–30), in which the bulky chiral moieties are positioned at the chain termini. The threading rates of guests **17** and **18** were similar, which is expected based on the similar degree of steric bulk. More interestingly, (*R*,*R*)-**18** showed a factor 3 (ΔΔ*G*^‡^_on_ = 3 kJ mol^−1^) kinetic preference for threading host (*S*,*S*,*S*,*S*)-**H23** over (*R*,*R*,*R*,*R*)-**H23** (Fig. 4G). The opposite kinetic selectivity was observed when guest (*S*,*S*)-**18** was threaded (Fig. 4H). The results indicate that the chiral environment of the chain termini attached to the viologen moiety determines the extent of kinetic selectivity. Interestingly, de-threading of guests **16–18** from hosts **H23** (see the ESI, Fig. S68–S103†) followed the same trends as observed for the corresponding threading processes, *i.e.* a kinetic preference in the threading process is associated with a kinetic preference in the de-threading process. As a result, the thermodynamic selectivity for all diastereomeric complexes of guests **16–18** with hosts **H23** is low, *i.e.* all binding constants are similar. Moreover, all the host–guest complexes **H23·16**, **H23·17**, and **H23·18** display similar ^1^H NMR spectra (see the ESI, Fig. S108–S110†). The methyl protons **a** and **b** are again deshielded by ∼2 ppm units. In addition, the aromatic viologen protons are shielded by 2–4 ppm units. The complexation induced shifts (CIS-values) for the protons of the macrocyclic hosts **H23** and the protons of the guests **16–18** near the viologen binding station are nearly identical for all host–guest complexes **H23·16**, **H23·17**, and **H23·18** (see the ESI, Tables S65–S67†). Finally, for all the investigated guests **16–18**, the kinetic events of threading and de-threading involving **H21** occur faster than those involving **H23**, but the resulting thermodynamic equilibria remain mostly unaffected.

## Conclusions

We have successfully synthesized chiral *C*_2_-symmetric porphyrin macrocyclic hosts (*R*,*R*,*R*,*R*)-**H23** and (*S*,*S*,*S*,*S*)-**H23** in an enantioselective fashion from the naturally abundant compounds ethyl (*S*)-lactate and ethyl (*R*)-lactate, respectively. The chiral information present in the flexible spacers of (*R*,*R*,*R*,*R*)-**H23** and (*S*,*S*,*S*,*S*)-**H23** is effectively transferred throughout the entire macrocycle up to the porphyrin, as was evidenced by CD spectroscopy and confirmed by DFT calculations. The macrocyclic hosts display the characteristic features of the achiral macrocycle **H21**, such as binding and threading of viologen guests. While the thermodynamics of binding achiral guests in **H21** and **H23** are very similar, the underlying kinetics are completely different, since threading of the guests through **H23** is significantly slower than threading through **H21**. This deceleration allows for a more controlled threading process. As we aim to store data on polymers *via* catalytic reactions on a polymer chain, gaining spatio-temporal control is highly desired. Another step in this direction is the realization of diastereoselectivity in the threading process. Chiral viologen guests **16** and **18**, equipped with chiral *sec*-butoxy and 1-phenylethoxy termini, respectively, display a significant kinetic preference (ΔΔ*G*^‡^_on_ = 1–3 kJ mol^−1^) for binding in one enantiomer of **H23** over the other. In contrast, dihydrocitronellyl-appended viologens **15** and 1-(4-octylphenyl)ethoxy-appended viologens **17** do not display such a kinetic preference. The reason for this difference in kinetic stereoselectivity is that the chirality of guests **16** and **18** is located at the termini of their chain substituents, which are involved in the initial interaction with the chiral cavities of hosts **H23**. The rate of the subsequent threading process is dictated by the relative stereochemistry of host and guest. The termini of guests **15** and **17**, however, are achiral, and therefore the initial interactions of these termini with the cavities of the enantiomers of hosts **H23** are similar. Therefore, we conclude that chirality at the chain terminus of the viologen guest is a prerequisite for realizing kinetic stereoselectivity. This conclusion fits with the previously observed ‘entron’ effect, which states that an initial binding event between the chain terminus of the guest inside the cavity of the host dictates the rate of the threading process.^30^ A bulky chiral moiety at the chain terminus further enhances the kinetic stereoselectivity, either by steric interactions or by favourable supramolecular interactions of the phenyl substituent. We are currently investigating manganese(iii) derivatives of (*R*,*R*,*R*,*R*)-**H23** and (*S*,*S*,*S*,*S*)-**H23**, which may potentially be used as processive catalysts for the stereoselective epoxidation of double bond-containing chiral polymer chains.

## Conflicts of interest

There are no conflicts to declare.

## Supplementary Material

SC-012-D0SC05233G-s001
