# Supplementary material for: Enantioselective synthesis of chiral porphyrin macrocyclic hosts and kinetic enantiorecognition of viologen guests
Source: Chem Sci. 2021 Jan 13;12(5):1661–7. doi: 10.1039/d0sc05233g (PMC8179042; doi:10.1039/d0sc05233g)
Supplement: SC-012-D0SC05233G-s001 [file SC-012-D0SC05233G-s001.pdf]

Electronic Supplementary Material (ESI) for Chemical Science.
This journal is © The Royal Society of Chemistry 2021

Electronic Supplementary Information

Enantioselective synthesis of chiral porphyrin macrocyclic hosts and kinetic enantiorecognition of viologen guests

Pieter J. Gilissen,[a,‡] Annemiek D. Slootbeek,[a,‡] Jiangkun Ouyang,[a] Nicolas Vanthuyne,[b] Rob Bakker,[a] Johannes A. A. W. Elemans[a,*] and Roeland J. M. Nolte[a,*]

[a]*Institute for Molecules and Materials, Radboud University, Heyendaalseweg 135, 6525 AJ Nijmegen, The Netherlands. E-mail: r.nolte@science.ru.nl; j.elemans@science.ru.nl*

[b]*Aix Marseille Univ, CNRS, Centrale Marseille, iSm2, Marseille, France.*

‡ These authors contributed equally to this work.

1. Table of contents

2. Experimental section 3
2.1. General information 3
2.2. Experimental details 4
2.2.1. Synthesis 4
2.2.2. Resolution of secondary alcohol *rac*-**21** 23
2.2.3. Fluorescence host-guest titrations 30
2.2.4. Fluorescence threading studies 33
2.2.5. Fluorescence dethreading studies 63
2.2.6. NMR host-guest titration 93
2.2.7. NMR characterization of 1:1 host-guest complexes 94
3. DFT calculations 105
4. References 110
5. Copies of NMR and optical spectra of new compounds 111

2. Experimental section

2.1. General information

N,*O*-Acetal **10**,[S1] porphyrin tetraol **$H_2$12**,[S1] methyl viologen bis(hexafluorophosphate) **13**,[S2] polymer-appended viologen bis(hexafluorophosphate) **14**,[S3] dihydrocitronellyl-appended viologen bis(hexafluorophosphate) (*R*,*R*)-**15**,[S4] dihydrocitronellyl-appended viologen bis(hexafluorophosphate) (*S*,*S*)-**15**,[S4] and hex-5-yn-1-yl-appended viologen bis(hexafluorophosphate) **29**[S5] were prepared according to literature procedures. Dichloromethane was distilled from calcium hydride under a nitrogen atmosphere. Tetrahydrofuran was distilled from potassium under a nitrogen atmosphere. Toluene was distilled from sodium under a nitrogen atmosphere. Chloroform was distilled from phosphorus pentoxide under a nitrogen atmosphere, and subsequently filtered through anhydrous potassium carbonate and purged with argon before use. Acetonitrile was distilled from calcium hydride under an argon atmosphere and purged with argon before use. Deuterated chloroform and acetonitrile were dried over molecular sieves (4Å) and purged with argon before use. Other solvents and reagents were obtained from commercial suppliers and used without further purification. Reactions were followed by using thin-layer chromatography (TLC) on silica gel-coated plates (Merck 60 F254). Detection was performed with UV light at 254 nm and/or by charring at 150 °C after dipping in an aqueous solution of potassium permanganate. Column chromatography was performed manually using Acros silica gel, 0.035−0.070 mm, 60A, Merck silica gel, 60H, and Acros aluminium oxide, 0.050–0.200 mm, 60A. Optical rotations were measured on an Anton Paar MCP100 polarimeter. Melting points were taken on a polarization microscope with a programmable hot-stage. NMR spectra were recorded at 298 K on a Bruker Avance III 500 spectrometer (500 MHz) equipped with a Prodigy BB cryoprobe or on a Bruker Avance III 400 spectrometer (400 MHz) equipped with a BBFO probe. ^{1}H NMR chemical shifts (δ_H) are given in parts per million (ppm) and were referenced to tetramethylsilane (TMS, 0.00 ppm). Carbon chemical shifts (δ_C) are given in ppm and were referenced to the carbon signals of the deuterated solvents ($CDCl_3$ δ_C = 77.16, CD_3CN δ_C = 118.26). Data for ^{1}H NMR spectra are reported as follows: chemical shift (multiplicity, coupling constant, integration, assignment). Data for ^{13}C NMR spectra are reported as follows: chemical shift (assignment). Multiplicities are abbreviated as s (singlet), d (doublet), t (triplet), q (quartet), p (quintet), m (multiplet), b (broad). Coupling constants are reported as *J* values in Hertz (Hz). Assignments were based on ^{1}H, ^{13}C, COSY, HSQC, HMBC, and ROESY NMR spectra. Mass spectra were recorded on a JEOL AccuTOF CS JMS-T100CS mass spectrometer (ESI) and on a JEOL TMS-100GCv mass spectrometer (EI). UV-vis spectra were recorded at 298 K on a JASCO V-630 UV-vis spectrophotometer (1 cm quartz cell). Fluorescence spectra were recorded at 298 K on a JASCO FP-8300ST spectrofluorometer (1 cm quartz cell). ECD spectra were recorded at 298 K on a JASCO J-815 CD spectrophotometer (2 mm quartz cell). DFT calculations were performed at B3LYP/6-311+G(d) level.

2.2. Experimental details

2.2.1. Synthesis

Fig. 2 (See main text) describes the synthesis of the enantiomers of chiral porphyrin macrocyclic host **$H_2$3**. Scheme S1 describes the synthesis of chiral viologens **16**–**18**.

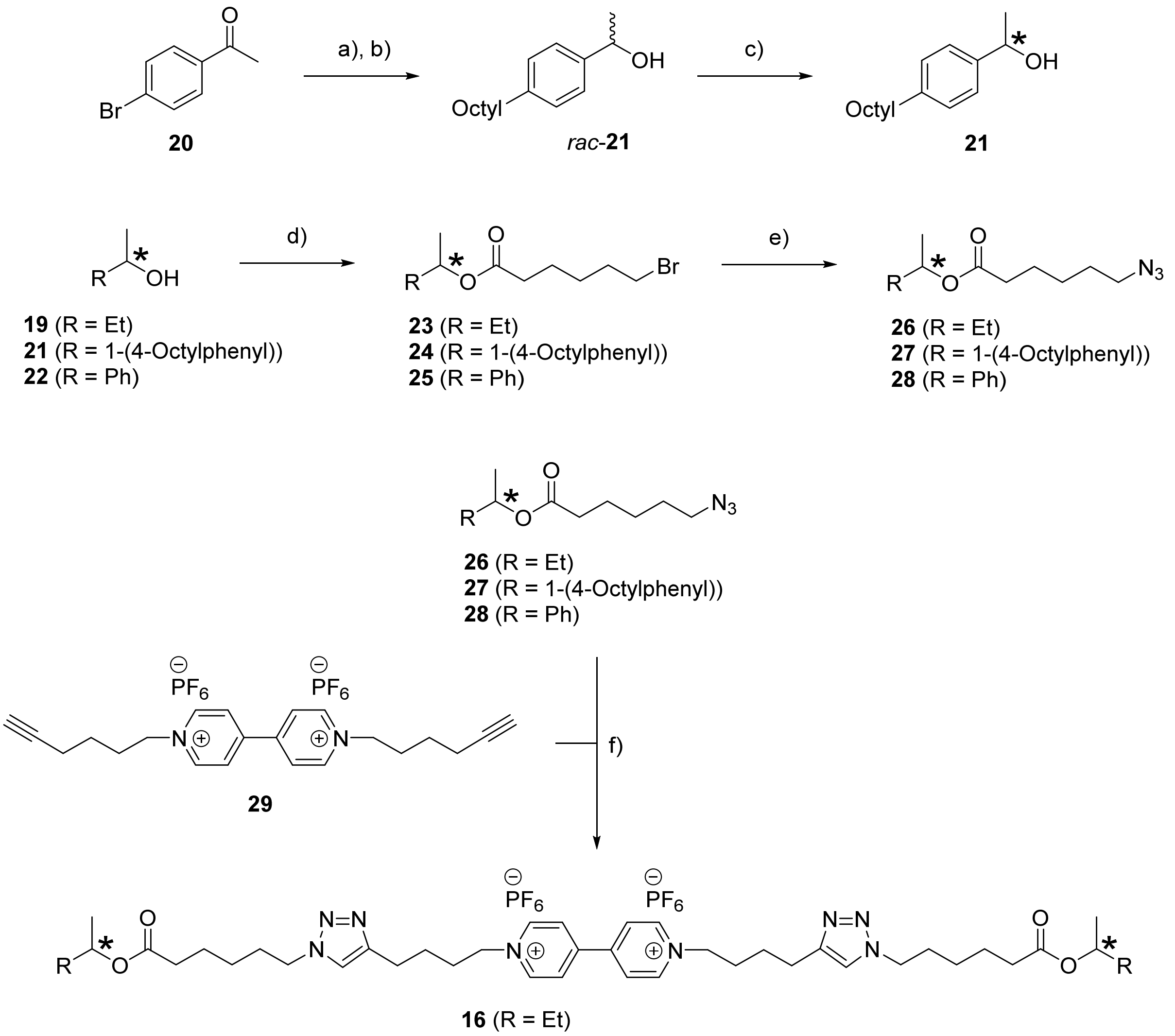

*Scheme S1. Synthesis of chiral viologens **16–18**; reagents and conditions: a) octylboronic acid, Pd(dppf)Cl_2·CH_2Cl_2, K_2CO_3, dioxane/H_2O, reflux; b) $NaBH_4$, CH_2Cl_2/MeOH, 20 °C; c) Lux-Cellulose-1, hexane/isopropanol; d) 6-bromohexanoyl chloride, CH_2Cl_2, 0 → 20 °C; e) NaN_3, DMF, 70 °C; f) $CuSO_4$·$5H_2O$, ascorbic acid, DMF, 20 °C; then NH_4PF_6. The asterisk (*) indicates enantiopure compounds.*

Ethyl (R)-2-(2-(benzyloxy)phenoxy) propanoate ((R)-5)

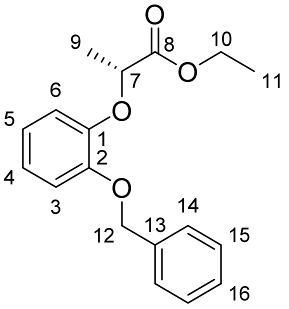

(R)-**5**

DIAD (4.20 mL, 21.6 mmol, 1.25 equiv) was added dropwise at 0 °C to a stirred solution of 2-benzyloxyphenol (3.46 g, 17.3 mmol, 1.0 equiv), ethyl (*S*)-lactate (1.98 mL, 17.3 mmol, 1.0 equiv) and triphenylphosphine (5.67 g, 21.6 mmol, 1.25 equiv) in dry toluene (20 mL) under an argon atmosphere. The yellow solution was stirred at 0 °C for 3 hours, and then at 20 °C for 3 hours. Upon completion (indicated by TLC, eluent: CH_2Cl_2), the white precipitate was filtered off and washed with toluene (20 mL). The combined filtrate was evaporated to dryness and the resulting oil was purified by 60A silica gel column chromatography (eluent: CH_2Cl_2/*n*-heptane, 1:1 → 1:0, v/v) to afford ester (*R*)-**5** (3.22 g, 62%) as a colorless transparent oil. $[\alpha]_D^{20}$ +76.3° (c = 1.353 g/100 mL, EtOAc); ^{1}H NMR (500 MHz, $CDCl_3$) δ 7.47–7.43 (m, 2H, 14-C*H*), 7.39–7.34 (m, 2H, 15-C*H*), 7.33–7.28 (m, 1H, 16-C*H*), 6.97–6.90 (m, 3H, 3-C*H* + 4-C*H* + 6-C*H*), 6.88 (ddd, *J* = 7.6, 5.6, 3.6 Hz, 1H, 5-C*H*), 5.15 (d, *J* = 12.1 Hz, 1H, 12-CH_a), 5.11 (d, *J* = 12.1 Hz, 1H, 12-CH_b), 4.77 (q, *J* = 6.8 Hz, 1H, 7-C*H*), 4.19 (dq, *J* = 10.8, 7.1 Hz, 1H, 10-CH_a), 4.16 (dq, *J* = 10.8, 7.1 Hz, 1H, 10-CH_b), 1.61 (d, *J* = 6.8 Hz, 3H, 9-CH_3), 1.23 (t, *J* = 7.1 Hz, 3H, 11-CH_3); ^{13}C NMR (126 MHz, $CDCl_3$) δ 172.48 (8-*C*), 149.74 (2-*C*), 148.01 (1-*C*), 137.41 (13-*C*), 128.58 (15-*C*), 127.92 (16-*C*), 127.46 (14-*C*), 123.13 (4-*C*), 121.75 (5-*C*), 118.18 (6-*C*), 115.67 (3-*C*), 74.89 (7-*C*), 71.43 (12-*C*), 61.18 (10-*C*), 18.80 (9-*C*), 14.29 (11-*C*); HRMS (ESI) calcd. for $[C_{18}H_{20}O_4 + Na]^+$ 323.1259, found 323.1257.

Ethyl (S)-2-(2-(benzyloxy)phenoxy) propanoate ((S)-5)

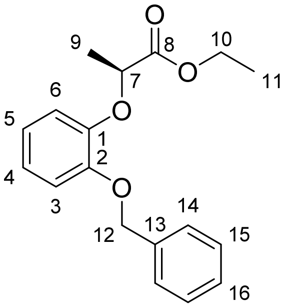

(S)-**5**

According to the procedure for (*R*)-**5**, the reaction of 2-benzyloxyphenol (9.0 g, 45 mmol, 1.0 equiv), ethyl (*R*)-lactate (5.2 mL, 45 mmol, 1.0 equiv), DIAD (11 mL, 56 mmol, 1.25 equiv) and triphenylphosphine (15 g, 56 mmol, 1.25 equiv) in dry toluene (50 mL) afforded (*S*)-**5** (9.76 g, 72%) as a colorless transparent oil. $[\alpha]_D^{20}$ −87.2° (c = 1.285 g/100 mL, EtOAc). Other spectral data were in agreement with those obtained for (*R*)-**5**.

*Ethyl (R)-2-(2-hydroxyphenoxy) propanoate ((R)-**6**)*

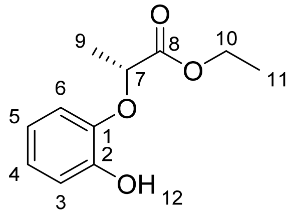

(*R*)-**6**

Palladium on carbon (10 w%, 1.0 g, 1.0 mmol, 0.10 equiv) was added to a stirred solution of aryl benzyl ether (*R*)-**5** (3.0 g, 10 mmol, 1.0 equiv) in EtOAc (150 mL) under an argon atmosphere. The black suspension was purged with hydrogen (balloon) and the resulting mixture was stirred at 20 °C for 4.5 hours under a hydrogen atmosphere. Upon completion (indicated by TLC, eluent: EtOAc/*n*-heptane, 1:3, v/v), the mixture was filtered through a plug of celite and the filter cake was washed with EtOAc (50 mL). The combined filtrate was evaporated to dryness and dried under high vacuum to afford phenol (*R*)-3 (2.05 g, 98%) as a colorless transparent oil. $[\alpha]_D^{20}$ +38.0° (c = 1.500 g/100 mL, EtOAc); ^{1}H NMR (500 MHz, $CDCl_3$) δ 6.99 (s, 1H, 12-O*H*), 6.97–6.90 (m, 2H, 3-C*H* + 4-C*H*), 6.87 (dd, *J* = 8.0, 1.4 Hz, 1H, 6-C*H*), 6.78 (ddd, *J* = 8.0, 6.2, 2.7 Hz, 1H, 5-C*H*), 4.65 (q, *J* = 6.9 Hz, 1H, 7-C*H*), 4.22 (dq, *J* = 10.7, 7.1 Hz, 1H, 10-CH_a), 4.18 (dq, *J* = 10.7, 7.1 Hz, 1H, 10-CH_b), 1.66 (d, *J* = 7.0 Hz, 3H, 9-CH_3), 1.23 (t, *J* = 7.2 Hz, 3H, 11-CH_3). Note: 12-O*H* signal displayed variable chemical shift; ^{13}C NMR (126 MHz, $CDCl_3$) δ 173.44 (8-*C*), 147.95 (2-*C*), 145.64 (1-*C*), 124.16 (4-*C*), 120.17 (5-*C*), 117.26 (6-*C*), 116.32 (3-*C*), 76.63 (7-*C*), 61.92 (10-*C*), 19.05 (9-*C*), 14.07 (11-*C*); HRMS (ESI) calcd. for $[C_{11}H_{14}O_4 + Na]^+$ 233.0790, found 233.0778.

*Ethyl (S)-2-(2-hydroxyphenoxy) propanoate ((S)-**6**)*

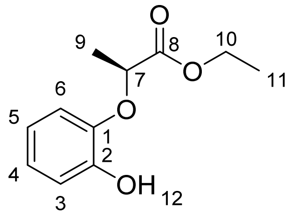

(*S*)-**6**

According to the procedure for (*R*)-**6**, the reaction of (*S*)-**5** (9.4 g, 31 mmol, 1.0 equiv), palladium on carbon (10 w%, 1.6 g, 1.6 mmol, 0.05 equiv) and hydrogen (balloon) in EtOAc (300 mL) afforded (*S*)-**6** (6.6 g, 99%) as a colorless transparent oil. $[\alpha]_D^{20}$ −35.8° (c = 1.873 g/100 mL, EtOAc). Other spectral data were in agreement with those obtained for (*R*)-**6**.

Diethyl 2,2′-(1,2-phenylenebis(oxy))(2R,2′R)-dipropionate ((R,R)-7)

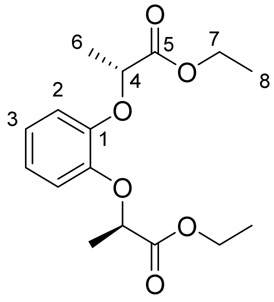

(*R*,*R*)-**7**

DIAD (2.20 mL, 11.3 mmol, 1.25 equiv) was added dropwise at 0 °C to a stirred solution of phenol (*R*)-**6** (1.9 g, 9.0 mmol, 1.0 equiv), ethyl (*S*)-lactate (1.04 mL, 9.0 mmol, 1.0 equiv) and triphenylphosphine (3.0 g, 11.3 mmol, 1.25 equiv) in dry toluene (15 mL) under an argon atmosphere. The yellow solution was stirred at 0 °C for 3.5 hours, and then at 20 ° for 1 hour. Upon completion (indicated by TLC, eluent: CH_2Cl_2), the white precipitate was filtered off and washed with toluene (15 mL). The combined filtrate was evaporated to dryness and the resulting oil was purified by 60A silica gel column chromatography (eluent: CH_2Cl_2/*n*-heptane, 1:1 → 1:0, v/v) to afford diester (*R*,*R*)-**7** (1.61 g, 57%) as a colorless transparent oil. $[\alpha]_D^{20}$ +103.7° (c = 1.447 g/100 mL, EtOAc); ^{1}H NMR (500 MHz, $CDCl_3$) δ 6.97–6.88 (m, 4H, 2-C*H* + 3-C*H*), 4.65 (q, *J* = 6.8 Hz, 2H, 4-C*H*), 4.21 (dq, *J* = 10.7, 7.1 Hz, 2H, 7-CH_a), 4.18 (dq, *J* = 10.7, 7.1 Hz, 2H, 7-CH_b), 1.65 (d, *J* = 6.8 Hz, 6H, 6-CH_3), 1.25 (t, *J* = 7.1 Hz, 6H, 8-CH_3); ^{13}C NMR (126 MHz, $CDCl_3$) δ 172.47 (5-*C*), 148.60 (1-*C*), 122.99 (3-*C*), 118.00 (2-*C*), 74.65 (4-*C*), 61.21 (7-*C*), 18.79 (6-*C*), 14.27 (8-*C*); HRMS (ESI) calcd. for $[C_{16}H_{22}O_6 + Na]^+$ 333.1314, found 333.1318.

Diethyl 2,2′-(1,2-phenylenebis(oxy))(2S,2′S)-dipropionate ((S,S)-7)

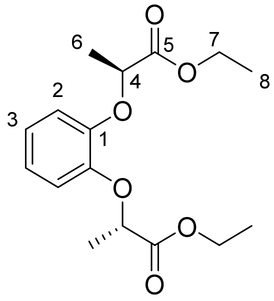

(*S*,*S*)-**7**

According to the procedure for (*R*,*R*)-**7**, the reaction of (*S*)-**6** (6.0 g, 29 mmol, 1.0 equiv), ethyl (*R*)-lactate (3.3 mL, 29 mmol, 1.0 equiv), DIAD (6.9 mL, 36 mmol, 1.25 equiv) and triphenylphosphine (9.4 g, 36 mmol, 1.25 equiv) in dry toluene (35 mL) afforded (*S*,*S*)-**7** (6.56 g, 74%) as a colorless transparent oil. $[\alpha]_D^{20}$ −100.1° (c = 1.858 g/100 mL, EtOAc). Other spectral data were in agreement with those obtained for (*R*,*R*)-**7**.

(2R,2′R)-2,2′-(1,2-Phenylenebis(oxy))bis(propan-1-ol) ((R,R)-8)

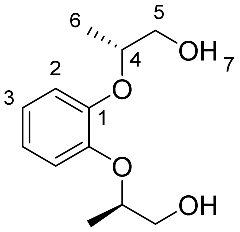

(*R*,*R*)-**8**

$LiAlH_4$ (1.06 g, 28 mmol, 6.0 equiv) was added portionwise at 0 °C to a stirred solution of diester (*R*,*R*)-**7** (1.45 g, 4.7 mmol, 1.0 equiv) in dry THF (60 mL) under an argon atmosphere. The grey suspension was stirred at 0 °C for 2 hours, and then at 20 °C for 1 hour. Upon completion (indicated by TLC, eluent: EtOAc), the reaction was quenched by the careful addition of water (5 mL). Subsequently, aqueous 1M NaOH (5 mL) and water (50 mL) were added and the product was extracted with CH_2Cl_2 (3 × 100 mL). The combined organic extracts were washed with water (100 mL), dried over Na_2SO_4 and the solvent was removed in vacuo to afford diol (*R*,*R*)-**8** (0.99 g, 94%) as a colorless transparent oil. $[\alpha]_D^{20}$ −53.6° (c = 1.474 g/100 mL, EtOAc); ^{1}H NMR (500 MHz, $CDCl_3$) δ 7.03–6.95 (m, 4H, 2-C*H* + 3-C*H*), 4.35 (pd, *J* = 6.3, 2.8 Hz, 2H, 4-C*H*), 3.75 (dd, *J* = 11.8, 2.2 Hz, 2H, 5-CH_a), 3.63 (dd, *J* = 12.0, 6.1 Hz, 2H, 5-CH_b), 3.40 (bs, 2H, 7-O*H*), 1.32 (d, *J* = 6.2 Hz, 6H, 6-CH_3). Note: 7-O*H* signal displayed variable chemical shift; ^{13}C NMR (126 MHz, $CDCl_3$) δ 149.06 (1-*C*), 123.26 (3-*C*), 119.57 (2-*C*), 78.45 (4-*C*), 65.99 (5-*C*), 16.23 (6-*C*); HRMS (ESI) calcd. for $[C_{12}H_{18}O_4 + Na]^+$ 249.1103, found 249.1094.

(2S,2′S)-2,2′-(1,2-Phenylenebis(oxy))bis(propan-1-ol) ((S,S)-8)

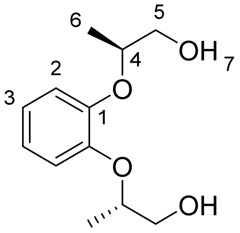

(*S*,*S*)-**8**

According to the procedure for (*R*,*R*)-**8**, the reaction of (*S*,*S*)-**7** (6.0 g, 19 mmol, 1.0 equiv) and $LiAlH_4$ (4.4 g, 0.12 mol, 6.0 equiv) in dry THF (200 mL) afforded (*S*,*S*)-**8** (4.16 g, 95%) as a colorless transparent oil. $[\alpha]_D^{20}$ +53.4° (c = 1.722 g/100 mL, EtOAc). Other spectral data were in agreement with those obtained for (*R*,*R*)-**8**.

(2R,2'R)-(1,2-Phenylenebis(oxy))bis(propane-2,1-diyl) bis(4-methylbenzenesulfonate) ((R,R)-9)

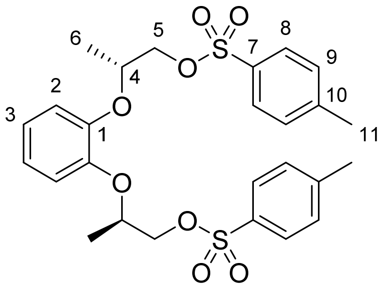

(*R*,*R*)-**9**

Pyridine (1.7 mL, 21 mmol, 6.0 equiv) was added at 0 °C to a stirred solution of diol (*R*,*R*)-**8** (0.80 g, 3.5 mmol, 1.0 equiv) in dry CH_2Cl_2 (10 mL) under an argon atmosphere. TsCl (2.0 g, 11 mmol, 3.0 equiv) was added portionwise over 5 minutes, the ice bath was removed and the resulting mixture was stirred at 20 °C for 21 hours. Upon completion (indicated by TLC, eluent: EtOAc), the mixture was diluted with CH_2Cl_2 (10 mL) and the organic layer was successively washed with water (10 mL), aqueous 6M HCl (10 mL), water (10 mL), and brine (10 mL). The resulting organic layer was dried over Na_2SO_4 and the solvent was removed in vacuo. The residual oil was purified by 60A silica gel column chromatography (eluent: CH_2Cl_2/*n*-heptane, 1:1 → 1:0, v/v) to afford ditosylate (*R*,*R*)-**9** (1.57 g, 83%) as a colorless sticky oil. $[\alpha]_D^{20}$ +23.1° (c = 1.256 g/100 mL, EtOAc); ^{1}H NMR (500 MHz, $CDCl_3$) δ 7.77 (d, *J* = 8.3 Hz, 4H, 8-C*H*), 7.32 (d, *J* = 8.0 Hz, 4H, 9-C*H*), 6.91–6.85 (m, 2H, 3-C*H*), 6.84–6.78 (m, 2H, 2-C*H*), 4.44 (qdd, *J* = 6.3, 5.6, 4.5 Hz, 2H, 4-C*H*), 4.12 (dd, *J* = 10.4, 5.6 Hz, 2H, 5-CH_a), 4.05 (dd, *J* = 10.4, 4.5 Hz, 2H, 5-CH_b), 2.43 (s, 6H, 11-CH_3), 1.26 (d, *J* = 6.3 Hz, 6H, 6-CH_3); ^{13}C NMR (126 MHz, $CDCl_3$) δ 148.45 (1-*C*), 145.03 (10-*C*), 132.98 (7-*C*), 130.01 (9-*C*), 128.07 (8-*C*), 123.05 (3-*C*), 118.99 (2-*C*), 73.67 (4-*C*), 71.81 (5-*C*), 21.78 (11-*C*), 16.23 (6-*C*); HRMS (ESI) calcd. for $[C_{26}H_{30}O_8S_2 + Na]^+$ 557.1280, found 557.1264.

(2S,2'S)-(1,2-Phenylenebis(oxy))bis(propane-2,1-diyl) bis(4-methylbenzenesulfonate) (S,S)-9)

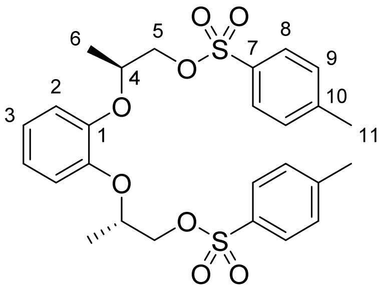

(*S*,*S*)-**9**

According to the procedure for (*R*,*R*)-**9**, the reaction of (*S*,*S*)-**8** (3.9 g, 17 mmol, 1.0 equiv), TsCl (9.9 g, 52 mmol, 3.0 equiv) and pyridine (8.3 mL, 0.10 mol, 6.0 equiv) in dry CH_2Cl_2 (50 mL) afforded (*S*,*S*)-**9** (9.2 g, 99%) as a colorless sticky oil. $[\alpha]_D^{20}$ −24.4° (c = 1.272 g/100 mL, EtOAc). Other spectral data were in agreement with those obtained for (*R*,*R*)-**9**.

(R,R,R,R)-Tetratosyl clip ((R,R,R,R)-11)

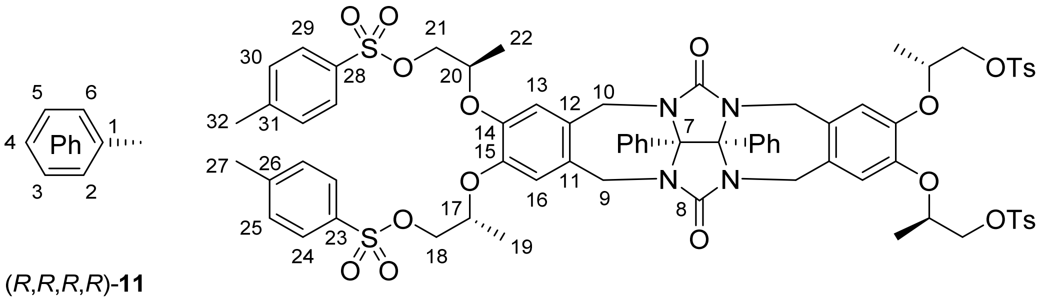

(*R*,*R*,*R*,*R*)-**11**

Zinc(II) chloride (1.3 g, 9.4 mmol, 9.0 equiv) and thionyl chloride (1.5 mL, 21 mmol, 20 equiv) were successively added to a stirred solution of *N*,*O*-acetal **10** (0.40 g, 1.05 mmol, 1.0 equiv) and ditosylate (*R*,*R*)-**9** (1.4 g, 2.6 mmol, 2.5 equiv) in dry CH_2Cl_2 (10 mL) under an argon atmosphere. The dark green suspension was stirred at 20 °C for 5.5 hours. Upon complete conversion of **10** (indicated by TLC, eluent: $CHCl_3/CH_3CN$, 9:1, v/v), the mixture was diluted with CH_2Cl_2 (20 mL), washed with aqueous 1M HCl (2 × 20 mL) and brine (20 mL). The organic layer was dried over Na_2SO_4 and the solvent was removed in vacuo. The residual off-white foam was purified by 60A silica gel column chromatography (eluent: $CHCl_3/CH_3CN$, 27:1 → 9:1, v/v) to afford chiral clip (*R*,*R*,*R*,*R*)-**11** (0.56 g, 38%) as a white solid. m.p. 94–95 °C; $[\alpha]_D^{20}$ +9.9° (c = 1.209 g/100 mL, EtOAc); ^{1}H NMR (500 MHz, $CDCl_3$) δ 7.68 (d, *J* = 8.5 Hz, 4H, 29-C*H*), 7.66 (d, *J* = 8.4 Hz, 4H, 24-C*H*), 7.24 (d, *J* = 8.0 Hz, 4H, 30-C*H*), 7.18–7.05 (m, 10H, 2-C*H* + 3-C*H* + 4-C*H* + 5-C*H* + 6-C*H*), 7.11 (d, *J* = 8.4 Hz, 4H, 25-C*H*), 6.69 (s, 2H, 16-C*H*), 6.67 (s, 2H, 13-C*H*), 4.64 (d, *J* = 15.9 Hz, 4H, 9-CH_a + 10-CH_a), 4.28 (qt, *J* = 6.4, 5.0 Hz, 2H, 17-C*H*), 4.14 (qdd, *J* = 6.3, 5.5, 4.7 Hz, 2H, 20-C*H*), 4.08 (d, *J* = 15.8 Hz, 2H, 9-CH_b), 4.07 (d, *J* = 15.8 Hz, 2H, 10-CH_b), 4.01 (d, *J* = 5.0 Hz, 4H, 18-CH_2), 4.00 (dd, *J* = 10.5, 5.5 Hz, 2H, 21-CH_a), 3.93 (dd, *J* = 10.5, 4.7 Hz, 2H, 21-CH_b), 2.38 (s, 6H, 32-CH_3), 2.33 (s, 6H, 27-CH_3), 1.12 (d, *J* = 6.4 Hz, 6H, 19-CH_3), 1.05 (d, *J* = 6.3 Hz, 6H, 22-CH_3); ^{13}C NMR (126 MHz, $CDCl_3$) δ 157.81 (8-*C*), 146.92 (14-*C*), 146.68 (15-*C*), 144.98 (26-*C* or 31-*C*)*, 144.96 (26-*C* or 31-*C*)*, 133.65 (1-*C*), 132.85 (28-*C*), 132.72 (23-*C*), 131.56 (11-*C* or 12-*C*)*, 131.53 (11-*C* or 12-*C*)*, 129.99 (30-*C*), 129.88 (25-*C*), 129.02 (2-*C* or 3-*C* or 4-*C* or 5-*C* or 6-*C*)*, 128.85 ((2×) 2-*C* or 3-*C* or 4-*C* or 5-*C* or 6-*C*)*, 128.35 (2-*C* or 3-*C* or 4-*C* or 5-*C* or 6-*C*)*, 128.31 (2-*C* or 3-*C* or 4-*C* or 5-*C* or 6-*C*)*, 127.97 (24-*C*), 127.91 (29-*C*), 119.75 (16-*C*), 119.64 (13-*C*), 85.50 (7-*C*), 73.35 (20-*C*), 73.20 (17-*C*), 71.91 (18-*C*), 71.81 (21-*C*), 44.93 (9-*C*), 44.87 (10-*C*), 21.72 (32-*C*), 21.68 (27-*C*), 16.92 (22-*C*), 16.55 (19-*C*). Note: carbon signals marked with an asterisk (*) could not be assigned unambiguously; HRMS (ESI) calcd. for $[C_{72}H_{74}N_4O_{18}S_4 + Na]^+$ 1433.3779, found 1433.3729.

(S,S,S,S)-Tetratosyl clip ((S,S,S,S)-11)

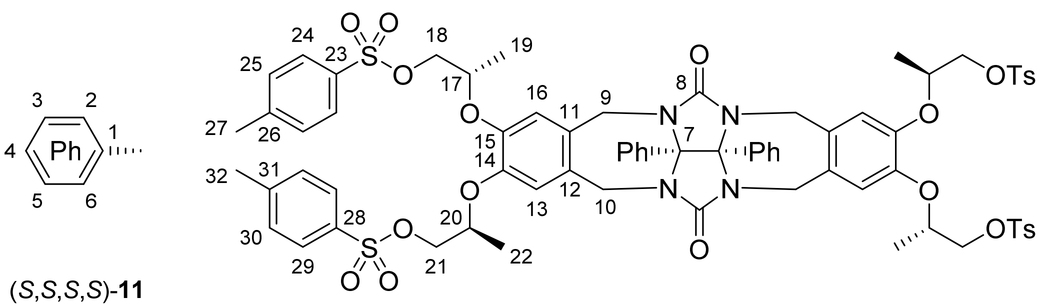

(*S*,*S*,*S*,*S*)-**11**

According to the procedure for (*R*,*R*,*R*,*R*)-**11**, the reaction of **10** (3.0 g, 7.8 mmol, 1.0 equiv), (*S*,*S*)- **9** (8.8 g, 16 mmol, 2.1 equiv), zinc(II) chloride (9.6 g, 70 mmol, 9.0 equiv) and thionyl chloride (11 mL, 0.16 mol, 20 equiv) in dry CH_2Cl_2 (80 mL) afforded (*S*,*S*,*S*,*S*)-**11** (3.89 g, 36%) as a white solid. $[\alpha]_D^{20}$ −9.0° (c = 1.443 g/100 mL, EtOAc). Other spectral data were in agreement with those obtained for (*R*,*R*,*R*,*R*)-**11**.

(R,R,R,R)-Porphyrin macrocyclic host ((R,R,R,R)-$H_2$3)

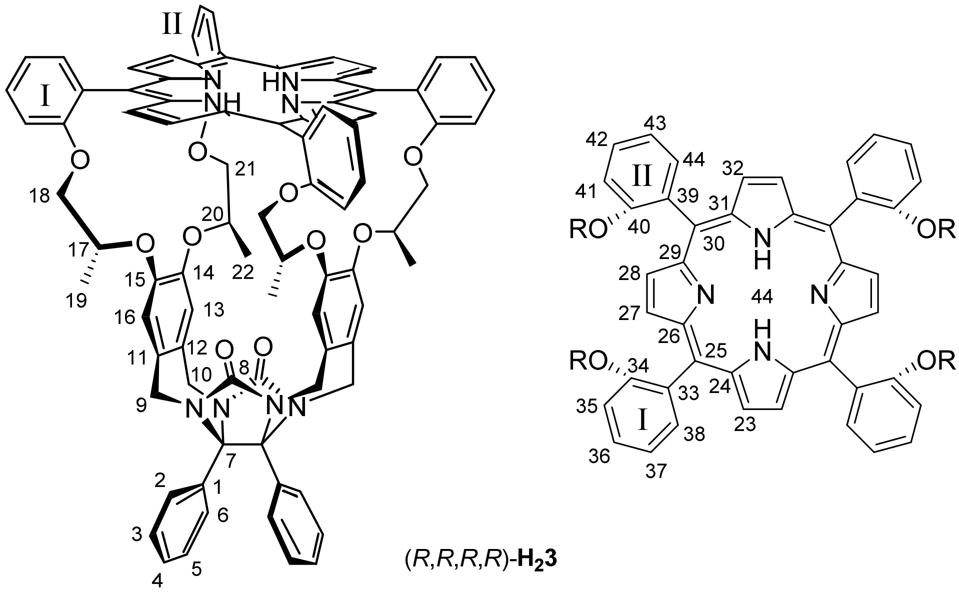

(*R*,*R*,*R*,*R*)-**$H_2$3**

K_2CO_3 (0.39 g, 2.8 mmol, 10 equiv) was added to a solution of porphyrin tetraol **$H_2$12** (0.19 g, 0.28 mmol, 1.0 equiv) and clip (*R*,*R*,*R*,*R*)-**11** (0.40 g, 0.28 mmol, 1.0 equiv) in MeCN (380 mL), and the resulting mixture was heated at reflux for 7 days under an argon atmosphere. After cooling, the mixture was filtered and the filter cake was washed with $CHCl_3$ until colorless washings were obtained. The combined filtrate was evaporated to dryness and purified by Alumina III column chromatography (eluent: $CHCl_3$). The purple fraction was evaporated to dryness and purified by 60H silica gel column chromatography (eluent: $CHCl_3$/CH_3CN, 50:1 → 20:1, v/v). The purified material was dissolved in a minimal amount of CH_2Cl_2 (5 mL) and precipitated by the addition of *n*-heptane (10 mL). Most CH_2Cl_2 was evaporated under reduced pressure and the resulting suspension was centrifuged. The supernatant was removed, and the pallet was washed with *n*-pentane (3 × 10 mL). The residual material was dried under high vacuum to afford porphyrin macrocyclic host (*R*,*R*,*R*,*R*)-**$H_2$3** (30 mg, 8%) as a purple solid. m.p. >300 °C (dec.); $[\alpha]_D^{20}$ could not be measured due to the intense purple color of the compound; ^{1}H NMR (500 MHz, $CDCl_3$) δ 8.77 (d, *J* = 4.7 Hz, 2H, 23-C*H*), 8.71 (d, *J* = 4.6 Hz, 2H, 28-C*H*), 8.69 (d, *J* = 4.8 Hz, 2H, 32-C*H*), 8.67 (d, *J* = 4.6 Hz, 2H, 27-C*H*), 8.33 (dd, *J* = 7.3, 1.7 Hz, 2H, 38-C*H*), 8.21 (dd, *J* = 7.4, 1.8 Hz, 2H, 44-C*H*), 7.79–7.73 (m, 4H, 36-C*H* + 42-C*H*), 7.51–7.43 (m, 4H, 37-C*H* + 43-C*H*), 7.32 (dd, *J* = 8.4, 1.2 Hz, 2H, 41-C*H*), 7.24 (dd, *J* = 8.2, 1.1 Hz, 2H, 35-C*H*), 7.09–7.04 (m, 2H, 5-C*H*), 6.98 (tt, *J* = 7.3, 1.3 Hz, 2H, 4-C*H*), 6.95–6.86 (m, 4H, 3-C*H* + 6-C*H*), 6.77 (dt, *J* = 8.3, 1.7 Hz, 2H, 2-C*H*), 6.35 (s, 2H, 16-C*H*), 6.09 (s, 2H, 13-C*H*), 4.27 (d, *J* = 15.5 Hz, 2H, 10-CH_a), 4.16 (d, *J* = 15.8 Hz, 2H, 9-CH_a), 3.98–3.89 (m, 2H, 20-C*H*), 3.80 (d, *J* = 4.5 Hz, 4H, 21-CH_2), 3.74 (d, *J* = 15.7 Hz, 2H, 9-CH_b), 3.73 (d, *J* = 15.4 Hz, 2H, 10-CH_b), 3.67 (dd, *J* = 8.3, 4.0 Hz, 2H, 18-CH_a), 3.37 (t, *J* = 8.5 Hz, 2H, 18-CH_b), 2.96–2.89 (m, 2H, 17-C*H*), −0.82 (d, *J* = 6.1 Hz, 6H, 19-CH_3), −1.18 (d, *J* = 5.2 Hz, 6H, 22-CH_3), −2.66 (s, 2H, 44-N*H*); ^{13}C NMR (126 MHz, $CDCl_3$) δ 159.76 (34-*C*), 159.47 (40-*C*), 157.17 (8-*C*), 146.94 (14-*C*), 143.25 (15-*C*), 134.85 (44-*C*), 133.86 (1-*C*), 133.67 (38-*C*), 133.46 (39-*C*), 132.78 (33-*C*), 131.85 (12-*C*), 130.06 (36-*C*), 130.01 (42-*C*), 129.12 (11-*C*), 128.76 (4-*C*), 128.66 (3-*C*), 128.52 (5-*C*), 128.44 (2-*C*), 127.77 (6-*C*), 124.59 (16-*C*), 121.00 (43-*C*), 120.46 (37-*C*), 115.43 (41-*C*), 115.41 (25-*C*), 115.06 (30-*C*), 114.22 (13-*C*), 113.73 (35-*C*), 85.15 (7-*C*), 73.34 (21-*C*), 72.69 (18-*C*), 72.37 (17-*C*), 70.63 (20-*C*), 44.69 (9-*C*), 44.06 (10-*C*), 14.96 (19-*C*), 12.83 (22-*C*). Note: carbon signals (23, 24, 26, 27, 28, 29, 31, 32) of the pyrrole units could not be observed, due to rapid tautomerization on the NMR timescale and hence broadening of the signal; HRMS (ESI) calcd. for $[C_{88}H_{72}N_8O_{10} + H]^+$ 1401.5450, found 1401.5444; UV-vis ($CHCl_3$/CH_3CN, 1:1, v/v) λ/nm (log(ε/$M^{-1}\cdot cm^{-1}$)) 416 (5.64), 512 (4.32), 543 (3.74), 589 (3.76), 644 (3.18); Fluorescence

(CHCl$_3$/CH$_3$CN, 1:1, v/v, λ_{ex} = 416 nm) λ_{em}/nm 647, 713; ECD (CHCl$_3$/CH$_3$CN, 1:1, v/v) λ/nm (Δε/M^{-1}·cm^{-1}) 257 (+14.1), 286 (−2.7), 416 (+34.4).

(S,S,S,S)-Porphyrin macrocyclic host ((S,S,S,S)-H$_2$3)

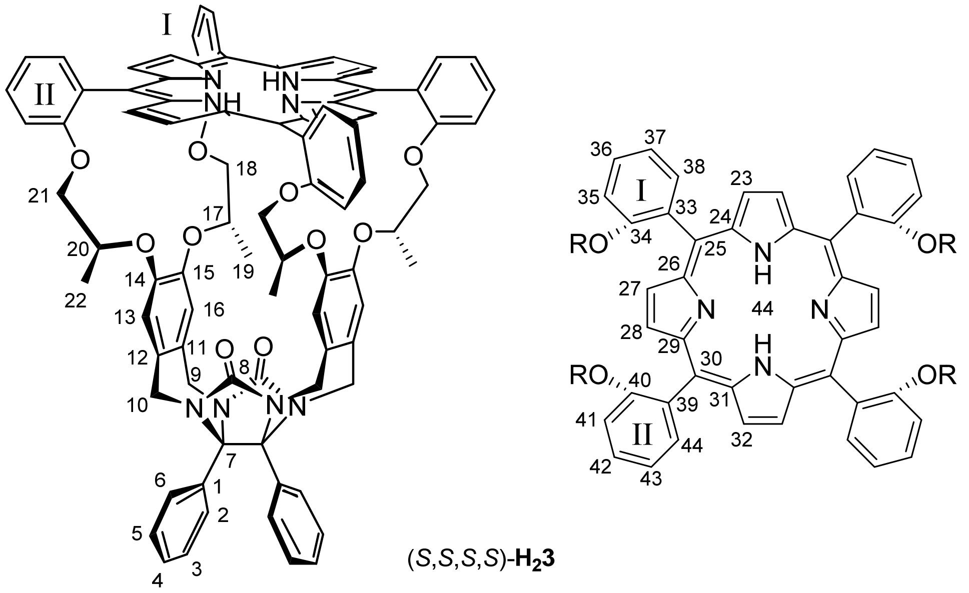

(*S*,*S*,*S*,*S*)-**H$_2$3**

According to the procedure for (*R*,*R*,*R*,*R*)-**H$_2$3**, the reaction of **H$_2$12** (0.30 g, 0.44 mmol, 1.0 equiv), (*S*,*S*,*S*,*S*)-**11** (0.62 g, 0.44 mmol, 1.0 equiv) and K$_2$CO$_3$ (0.61 g, 4.4 mol, 10 equiv) in CH$_3$CN (500 mL) afforded (*S*,*S*,*S*,*S*)-**H$_2$3** (32 mg, 5%) as a purple solid. ECD (CHCl$_3$/CH$_3$CN, 1:1, v/v) λ/nm (Δε/M^{-1}·cm^{-1}) 257 (−13.4), 286 (+2.4), 416 (−31.7). Other spectral data were in agreement with those obtained for (*R*,*R*,*R*,*R*)-**H$_2$3**.

*(R)-1-(4-Octylphenyl)ethan-1-ol ((R)-**21**) and (S)-1-(4-octylphenyl)ethan-1-ol ((S)-**21**)*

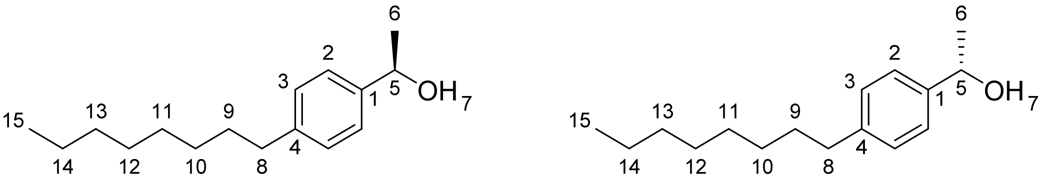

(*R*)-**21** (*S*)-**21**

A Schlenk flask was charged with 4'-bromoacetophenone (1.0 g, 5.0 mmol, 1.0 equiv), octylboronic acid (2.4 g, 15 mmol, 3.0 equiv), K_2CO_3 (4.1 g, 30 mmol, 6.0 equiv) and Pd(dppf)Cl_2·CH_2Cl_2 (90 mg, 0.10 mmol, 2 mol%). The flask was evacuated and refilled with argon three times. Then, degassed 1,4-dioxane/water (25 mL, 4:1, v/v) was added and the resulting mixture was refluxed for 7 hours under an argon atmosphere. Upon completion (indicated by TLC, eluent: EtOAc/*n*-heptane, 1:4, v/v), the mixture was diluted with EtOAc (50 mL). The organic layer was washed with water (50 mL), dried over Na_2SO_4 and the solvent was removed in vacuo. The residual oil was purified by 60A silica gel column chromatography (eluent: EtOAc/*n*-heptane, 1:15, v/v) to afford 4'-octylbenzophenone (0.89 g, 76%) as a colorless transparent oil. Then, 4'-octylbenzophenone (0.80 g, 3.45 mmol, 1.0 equiv) was dissolved in CH_2Cl_2/CH_3OH (100 mL, 1:4, v/v). Sodium borohydride (0.26 g, 6.9 mmol, 2.0 equiv) was added to the solution and the resulting mixture was stirred at 20 °C for 45 minutes. Upon completion (indicated by TLC, eluent: CH_2CL_2), the reaction was quenched by the addition of water (400 mL) and the product was extracted with CH_2Cl_2 (2 × 200 mL). The combined organic extracts were dried over Na_2SO_4 and the solvent was removed in vacuo. The residual oil was purified by 60A silica gel column chromatography (eluent: EtOAc/*n*-heptane, 1:10, v/v) to afford *rac*-**21** (0.75 g, 92%) as a colorless transparent oil. *Rac*-**21** (0.68 g, 2.9 mmol, 1.0 equiv) was purified by chiral HPLC (see section 2.2.2 for details) to afford (*R*)-**21** (309 mg, 45%) and (*S*)-**21** (307 mg, 45%), both as colorless transparent oils. ^{1}H NMR (500 MHz, $CDCl_3$) δ 7.30–7.25 (m, 2H, 2-C*H*), 7.18–7.13 (m, 2H, 3-C*H*), 4.86 (q, *J* = 6.4 Hz, 1H, 5-C*H*), 2.62–2.56 (m, 2H, 8-C*H*$_2$), 1.80 (bs, 1H, 7-O*H*), 1.65–1.55 (m, 2H, 8-C*H*$_2$), 1.48 (d, *J* = 6.4 Hz, 3H, 6-C*H*$_3$), 1.38–1.19 (m, 10H, 10-C*H*$_2$ + 11-C*H*$_2$ + 12-C*H*$_2$ + 13-C*H*$_2$ + 14-C*H*$_2$), 0.88 (t, *J* = 6.9 Hz, 3H, 15-C*H*$_3$); ^{13}C NMR (126 MHz, $CDCl_3$) δ 143.17 (1-*C*), 142.43 (4-*C*), 128.66 (3-*C*), 125.49 (2-*C*), 70.43 (5-*C*), 35.78 (8-*C*), 32.03 (13-*C*), 31.67 (9-*C*), 29.62 (10-*C* or 11-*C* or 12-*C*)*, 29.49 (10-*C* or 11-*C* or 12-*C*)*, 29.40 (10-*C* or 11-*C* or 12-*C*)*, 25.14 (6-*C*), 22.81 (14-*C*), 14.24 (15-*C*). Note: carbon signals marked with an asterisk (*) could not be assigned unambiguously; HRMS (ESI) calcd. for $[C_{16}H_{26}O + Na]^+$ 257.1881, found 257.1903.

(S)-sec-Butyl 6-bromohexanoate ((S)-23)

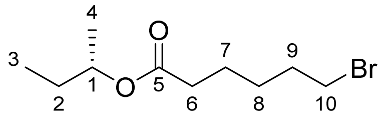

(*S*)-**23**

A flask was charged with (*S*)-**19** (0.30 g, 4.1 mmol, 1.0 equiv) and 6-bromohexanoyl chloride (1.06 g, 5.0 mmol, 1.2 equiv) under an argon atmosphere. Dry CH_2Cl_2 (16 mL) was added and the mixture was cooled to 0 °C and stirred for 30 minutes. Then the reaction mixture was allowed to warm to 20 °C, and stirred for 16 hours. The organic layer was washed with water (3 × 30 mL) and concentrated under reduced pressure. The residue was purified by 60A silica gel column chromatography (eluent: CH_2Cl_2/*n*-heptane, 1:4, v/v) to afford bromide (*S*)-**23** (0.83 g, 81%) as a colorless oil. $[\alpha]_D^{20}$ +13.3° (c = 1.0 g/100 mL, EtOH); ^{1}H NMR (500 MHz, $CDCl_3$) δ 4.88–4.82 (m, 1H, 1-C*H*), 3.41 (t, *J* = 6.7 Hz, 2H, 10-CH_2), 2.30 (t, *J* = 7.5 Hz, 2H, 6-CH_2), 1.93–1.84 (m, 2H, 9-CH_2), 1.70–1.62 (m, 2H, 7-CH_2), 1.62–1.44 (m, 4H, 2-CH_2 + 8-CH_2), 1.20 (d, *J* = 6.3 Hz, 3H, 4-CH_3), 0.90 (t, *J* = 7.5 Hz, 3H, 3-CH_3); ^{13}C NMR (126 MHz, $CDCl_3$) δ 173.25 (5-*C*), 72.20 (1-*C*), 34.55 (6-*C*), 33.60 (10-*C*), 32.54 (9-*C*), 28.92 (2-*C*), 27.76 (8-*C*), 24.32 (7-*C*), 19.61 (4-*C*), 9.82 (3-*C*).

(R)-sec-Butyl 6-bromohexanoate ((R)-23)

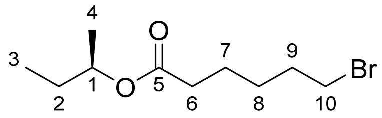

(*R*)-**23**

According to the procedure for (*S*)-**23**, the reaction of (*R*)-**19** (0.30 g, 4.1 mmol, 1.0 equiv) and 6-bromohexanoyl chloride (1.06 g, 5.0 mmol, 1.0 equiv) in dry CH_2Cl_2 (16 mL) afforded (*R*)-**23** (0.85 g, 84%) as a colorless oil. $[\alpha]_D^{20}$ −14.1° (c = 1.0 g/100 mL, EtOH). Other spectral data were in agreement with those obtained for (*S*)-**23**.

(S)-1-(4-Octylphenyl)ethyl 6-bromohexanoate ((S)-24)

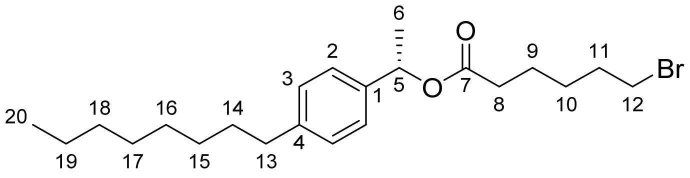

(*S*)-**24**

A flask was charged with (*S*)-**21** (0.19 g, 0.65 mmol, 1.0 equiv) and 6-bromohexanoyl chloride (0.17 g, 0.78 mmol, 1.2 equiv) under an argon atmosphere. Dry CH_2Cl_2 (4 mL) was added and the mixture was cooled to 0 °C and stirred for 30 minutes. Then the reaction mixture was allowed to warm to 20 °C, and stirred for 16 hours. The organic layer was washed with water (3 × 30 mL) and concentrated under reduced pressure. The residue was purified by 60A silica gel column chromatography (eluent: EtOAc/*n*-heptane, 1:20, v/v) to afford bromide (*S*)-**24** (150 mg, 56%) as a colorless oil. $[\alpha]_D^{20}$ −31.0° (c = 1.0 g/100 mL, EtOH); ^{1}H NMR (500 MHz, $CDCl_3$) δ 7.25 (d, *J* = 8.1 Hz, 2H, 3-C*H*), 7.15 (d, *J* = 8.2 Hz, 2H, 2-C*H*), 5.87 (q, *J* = 6.6 Hz, 1H, 5-C*H*), 3.37 (t, *J* = 6.8 Hz, 2H, 12-CH_2), 2.61–2.55 (m, 2H, 13-CH_2), 2.38–2.27 (m, 2H, 8-CH_2), 1.89–1.80 (m, 2H, 11-CH_2), 1.69–1.55 (m, 4H, 9-CH_2 + 14-CH_2), 1.52 (d, *J* = 6.6 Hz, 3H, 6-CH_3), 1.48–1.40 (m, 2H, 10-CH_2), 1.37–1.20 (m, 10H, 15-CH_2 + 16-CH_2 + 17-CH_2 + 18-CH_2 + 19-CH_2), 0.88 (t, *J* = 7.2 Hz, 3H, 20-CH_3); ^{13}C NMR (126 MHz, $CDCl_3$) δ 172.84 (7-*C*), 142.83 (4-*C*), 138.97 (1-*C*), 128.61 (2-*C*), 126.22 (3-*C*), 72.27 (5-*C*), 35.80 (13-*C*), 34.49 (8-*C*), 33.58 (12-*C*), 32.53 (11-*C*), 32.02 (18-*C*), 31.58 (14-*C*), 29.60 (15-*C* or 16-*C* or 17-*C*)*, 29.50 (15-*C* or 16-*C* or 17-*C*)*, 29.38 (15-*C* or 16-*C* or 17-*C*)*, 27.74 (10-*C*), 24.22 (9-*C*), 22.80 (19-*C*), 22.22 (6-*C*), 14.23 (20-*C*). Note: carbon signals marked with an asterisk (*) could not be assigned unambiguously.

(R)-1-(4-Octylphenyl)ethyl 6-bromohexanoate ((R)-24)

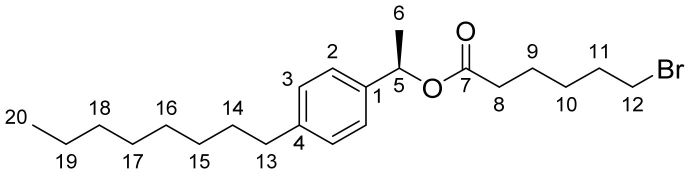

(*R*)-**24**

According to the procedure for (*S*)-**24**, the reaction of (*R*)-**21** (0.19 g, 0.65 mmol, 1.0 equiv) and 6-bromohexanoyl chloride (0.17 g, 0.78 mmol, 1.2 equiv) in dry CH_2Cl_2 (4 mL) afforded (*R*)-**24** (134 mg, 50%) as a colorless oil. $[\alpha]_D^{20}$ +33.0° (c = 1.0 g/100 mL, EtOH). Other spectral data were in agreement with those obtained for (*S*)-**24**.

(S)-1-Phenylethyl 6-bromohexanoate ((S)-25)

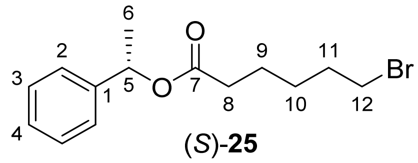

(*S*)-**25**

A flask was charged with (*S*)-**22** (0.50 g, 4.1 mmol, 1.0 equiv) and 6-bromohexanoyl chloride (0.87 g, 4.1 mmol, 1.0 equiv) under an argon atmosphere. Dry CH_2Cl_2 (16 mL) was added and the mixture was cooled to 0 °C and stirred for 30 minutes. Then the reaction mixture was allowed to warm to 20 °C, and stirred for 16 hours. The organic layer was washed with water (3 × 30 mL) and concentrated under reduced pressure. The residue was purified by 60A silica gel column chromatography (eluent: CH_2Cl_2/*n*-heptane, 1:4, v/v) to afford bromide (*S*)-**25** (0.99 g, 81%) as a colorless oil. $[\alpha]_D^{20}$ −50.6° (c = 0.968 g/100 mL, CH_2Cl_2); ^{1}H NMR (500 MHz, $CDCl_3$) δ 7.37–7.32 (m, 4H, 2-C*H* + 3-C*H*), 7.32–7.26 (m, 1H, 4-C*H*), 5.89 (q, *J* = 6.6 Hz, 1H, 5-C*H*), 3.38 (t, *J* = 6.8 Hz, 2H, 12-CH_2), 2.40–2.29 (m, 2H, 8-CH_2), 1.89–1.81 (m, 2H, 11-CH_2), 1.69–1.61 (m, 2H, 9-CH_2), 1.54 (d, *J* = 6.6 Hz, 3H, 6-CH_3), 1.49–1.41 (m, 2H, 10-CH_2); ^{13}C NMR (126 MHz, $CDCl_3$) δ 172.77 (7-*C*), 141.84 (1-*C*), 128.61 (2-*C*), 127.97 (4-*C*), 126.19 (3-*C*), 72.32 (5-*C*), 34.45 (8-*C*), 33.57 (12-*C*), 32.50 (11-*C*), 27.71 (10-*C*), 24.20 (9-*C*), 22.34 (6-*C*); HRMS (EI) molecular ion could not be detected, hence fragments are reported: calcd. for $[C_6H_{10}{}^{79}BrO]^+$ 176.9915, found 176.9888; calcd. for $[C_8H_{10}O]^+$ 122.0732, found 122.0710; calcd. for $[C_8H_8]^+$ 104.0626, found 104.0606.

(R)-1-Phenylethyl 6-bromohexanoate ((R)-25)

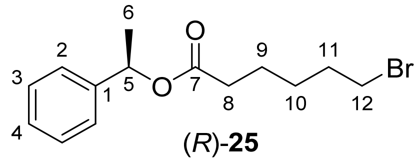

(*R*)-**25**

According to the procedure for (*S*)-**25**, the reaction of (*R*)-**22** (0.50 g, 4.1 mmol, 1.0 equiv) and 6-bromohexanoyl chloride (0.87 g, 4.1 mmol, 1.0 equiv) in dry CH_2Cl_2 (16 mL) afforded (*R*)-**25** (0.96 g, 79%) as a colorless oil. $[\alpha]_D^{20}$ +49.1° (c = 0.968 g/100 mL, CH_2Cl_2). Other spectral data were in agreement with those obtained for (*S*)-**25**.

(S)-sec-Butyl 6-azidohexanoate ((S)-26)

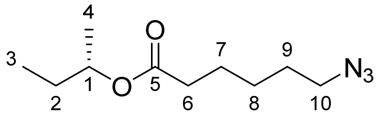

(*S*)-**26**

A flask was charged with (*S*)-**23** (0.80 g, 3.2 mmol, 1.0 equiv) and sodium azide (1.04 g, 16 mmol, 5.0 equiv) under an argon atmosphere. Dry DMF (5 mL) was added and the mixture was heated to 70 °C and stirred for 16 hours. After cooling, water (20 mL) and CH_2Cl_2 (50 mL) were added. The organic layer was separated, washed with water (3 × 20 mL), dried over Na_2SO_4 and concentrated under reduced pressure. The residue was purified by 60A silica gel column chromatography (eluent: CH_2Cl_2/*n*-heptane, 1:4, v/v) to afford azide (*S*)-**26** (0.62 g, 91%) as a colorless oil. $[\alpha]_D^{20}$ +12.7° (c = 1.0 g/100 mL, EtOH); ^{1}H NMR (500 MHz, CD_3CN) δ 4.82–4.74 (m, 1H, 1-C*H*), 3.28 (t, *J* = 6.9 Hz, 2H, 10-CH_2), 2.27 (t, *J* = 7.3 Hz, 2H, 6-CH_2), 1.64–1.48 (m, 6H, 2-CH_2 + 7-CH_2 + 9-CH_2), 1.41–1.33 (m, 2H, 8-CH_2), 1.17 (d, *J* = 6.3 Hz, 3H, 4-CH_3), 0.87 (t, *J* = 7.5 Hz, 3H, 3-CH_3); ^{13}C NMR (126 MHz, $CDCl_3$) δ 173.83 (5-*C*), 72.60 (1-*C*), 51.95 (10-*C*), 34.93 (6-*C*), 29.50 (2-*C*), 29.14 (9-*C*), 26.86 (8-*C*), 25.30 (7-*C*), 19.79 (4-*C*), 9.99 (3-*C*).

(R)-sec-Butyl 6-azidohexanoate ((R)-26)

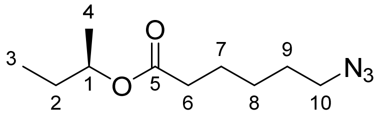

(*R*)-**26**

According to the procedure for (*S*)-**26**, the reaction of (*R*)-**23** (0.80 g, 3.2 mmol, 1.0 equiv) and sodium azide (1.04 g, 16 mmol, 5.0 equiv) in dry DMF (5 mL) afforded (*R*)-**26** (0.61 g, 89%) as a colorless oil. $[\alpha]_D^{20}$ −14.1° (c = 1.0 g/100 mL, EtOH). Other spectral data were in agreement with those obtained for (*S*)-**26**.

(S)-1-(4-Octylphenyl)ethyl 6-azidohexanoate ((S)-27)

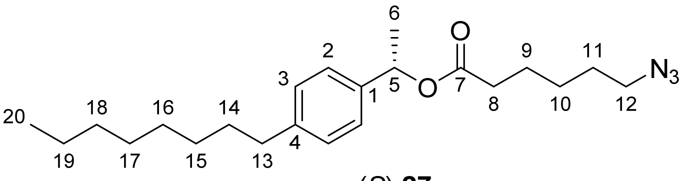

(*S*)-**27**

A flask was charged with (*S*)-**24** (100 mg, 0.24 mmol, 1.0 equiv) and sodium azide (79 mg, 1.2 mmol, 5.0 equiv) under an argon atmosphere. Dry DMF (3 mL) was added and the mixture was heated to 70 °C and stirred for 16 hours. After cooling, water (20 mL) and CH_2Cl_2 (30 mL) were added. The organic layer was separated, washed with water (3 × 20 mL), dried over Na_2SO_4 and concentrated under reduced pressure. The residue was purified by 60A silica gel column chromatography (eluent: CH_2Cl_2/*n*-heptane, 1:4, v/v) to afford azide (*S*)-**27** (84 mg, 93%) as a colorless oil. $[\alpha]_D^{20}$ −20.9° (c = 1.0 g/100 mL, EtOH); ^{1}H NMR (500 MHz, CD_3CN) δ 7.25 (d, *J* = 8.2 Hz, 2H, 3-C*H*), 7.17 (d, *J* = 8.2 Hz, 2H, 2-C*H*), 5.79 (q, *J* = 6.6 Hz, 1H, 5-C*H*), 3.25 (t, *J* = 6.8 Hz, 2H, 12-CH_2), 2.61–2.54 (m, 2H, 13-CH_2), 2.31 (t, *J* = 7.4 Hz, 2H, 8-CH_2), 1.64–1.51 (m, 6H, 9-CH_2 + 11-CH_2 + 14-CH_2), 1.47 (d, *J* = 6.6 Hz, 3H, 6-CH_3), 1.38–1.21 (m, 12H, 10-CH_2 + 15-CH_2 + 16-CH_2 + 17-CH_2 + 18-CH_2 + 19-CH_2), 0.88 (t, *J* = 7.1 Hz, 3H, 20-CH_3); ^{13}C NMR (126 MHz, CD_3CN) δ 173.45 (7-*C*), 143.56 (4-*C*), 140.56 (1-*C*), 129.39 (2-*C*), 126.86 (3-*C*), 72.77 (5-*C*), 51.94 (12-*C*), 36.07 (13-*C*), 34.86 (8-*C*), 32.59 (18-*C*), 32.27 (14-*C*), 30.11 (15-*C* or 16-*C* or 17-*C*)*, 29.99 (15-*C* or 16-*C* or 17-*C*)*, 29.96 (15-*C* or 16-*C* or 17-*C*)*, 29.11 (11-*C*), 26.81 (10-*C*), 25.20 (9-*C*), 23.35 (19-*C*), 22.52 (6-*C*), 14.35 (20-*C*). Note: carbon signals marked with an asterisk (*) could not be assigned unambiguously; HRMS (ESI) calcd. for $[C_{22}H_{35}N_3O_2 + Na]^+$ 396.2627, found 396.2630.

(R)-1-(4-Octylphenyl)ethyl 6-azidohexanoate ((R)-27)

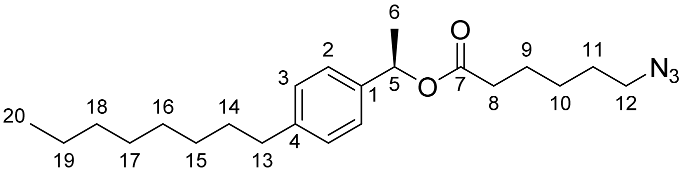

(*R*)-**27**

According to the procedure for (*S*)-**27**, the reaction of (*R*)-**24** (100 mg, 0.24 mmol, 1.0 equiv) and sodium azide (79 mg, 1.2 mmol, 5.0 equiv) in dry DMF (3 mL) afforded (*R*)-**27** (86 mg, 96%) as a colorless oil. $[\alpha]_D^{20}$ +30.6° (c = 1.0 g/100 mL, EtOH). Other spectral data were in agreement with those obtained for (*S*)-**27**.

(S)-1-Phenylethyl 6-azidohexanoate ((S)-28)

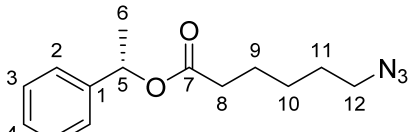

(*S*)-**28**

A flask was charged with (*S*)-**25** (0.50 g, 1.65 mmol, 1.0 equiv) and sodium azide (0.55 g, 8.25 mmol, 5.0 equiv) under an argon atmosphere. Dry DMF (5 mL) was added and the mixture was heated to 70 °C and stirred for 16 hours. After cooling, water (20 mL) and CH_2Cl_2 (50 mL) were added. The organic layer was separated, washed with water (3 × 20 mL), dried over Na_2SO_4 and concentrated under reduced pressure. The residue was purified by 60A silica gel column chromatography (eluent: CH_2Cl_2/*n*-heptane, 1:4, v/v) to afford azide (*S*)-**28** (0.41 g, 92%) as a colorless oil. $[\alpha]_D^{20}$ −62.5° (c = 0.935 g/100 mL, CH_2Cl_2); ^{1}H NMR (500 MHz, $CDCl_3$) δ 7.37–7.32 (m, 4H, 2-C*H* + 3-C*H*), 7.32–7.26 (m, 1H, 4-C*H*), 5.89 (q, *J* = 6.6 Hz, 1H, 5-C*H*), 3.23 (t, *J* = 6.9 Hz, 2H, 12-CH_2), 2.40–2.29 (m, 2H, 8-CH_2), 1.69–1.55 (m, 4H, 9-CH_2 + 11-CH_2), 1.53 (d, *J* = 6.6 Hz, 3H, 6-CH_3), 1.42–1.33 (m, 2H, 10-CH_2); ^{13}C NMR (126 MHz, $CDCl_3$) δ 172.76 (7-*C*), 141.84 (1-*C*), 128.60 (2-*C*), 127.97 (4-*C*), 126.19 (3-*C*), 72.32 (5-*C*), 51.32 (12-*C*), 34.46 (8-*C*), 28.64 (11-*C*), 26.28 (10-*C*), 24.56 (9-*C*), 22.32 (6-*C*).

(R)-1-Phenylethyl 6-azidohexanoate ((R)-28)

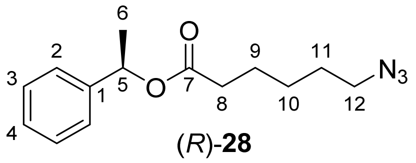

(*R*)-**28**

According to the procedure for (*S*)-**28**, the reaction of (*R*)-**25** (0.50 g, 1.65 mmol, 1.0 equiv) and sodium azide (0.55 g, 8.25 mmol, 5.0 equiv) in dry DMF (5 mL) afforded (*R*)-**28** (0.42 g, 96%) as a colorless oil. $[\alpha]_D^{20}$ +65.2° (c = 0.935 g/100 mL, CH_2Cl_2). Other spectral data were in agreement with those obtained for (*S*)-**28**.

*1,1'-Bis(4-(1-(6-oxo-6-((S)-sec-butoxy)hexyl)-1H-1,2,3-triazol-4-yl)butyl)-[4,4'-bipyridine]-1,1'-diium hexafluorophosphate ((S,S)-**16**)*

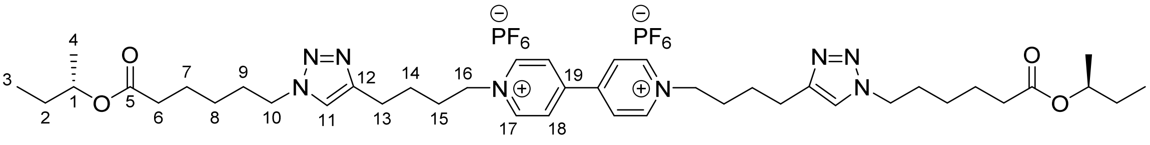

(*S*,*S*)-**16**

A flask was charged with azide (*S*)-**26** (61.5 mg, 0.28 mmol, 2.9 equiv), diyne **29** (58.5 mg, 96 µmol, 1.0 equiv) and copper(II) sulfate pentahydrate (16 mg, 64 µmol, 0.67 equiv) under an argon atmosphere. Dry DMF (3 mL) was added and the mixture was stirred at 20 °C for 30 minutes. Then, ascorbic acid (40 mg, 0.23 mmol, 2.4 equiv) was added and the mixture was stirred at 20 °C for 16 hours. The solvent was removed under high vacuum and the residue was suspended in acetonitrile (10 mL). The solid material was filtered off and the filtrate was evaporated to dryness and redissolved in acetonitrile (2 mL). Aqueous saturated ammonium hexafluorophosphate (3 mL) was added, resulting in the formation of a white precipitate. The precipitate was filtered off, washed with water (3 × 2 mL) and dried under high vacuum. The solid was redissolved in acetonitrile (2 mL) and precipitated by the addition of diethyl ether (20 mL). The precipitate was filtered off and dried under high vacuum to afford (*S*,*S*)-**16** (52 mg, 52%) as a white solid. m.p. 256–260 °C; $[\alpha]_D^{20}$ +11.0° (c = 1.0 g/100 mL, CH_3CN); ^{1}H NMR (500 MHz, CD_3CN) δ 8.90 (d, *J* = 7.0 Hz, 4H, 17-C*H*), 8.37 (d, *J* = 6.9 Hz, 4H, 18-C*H*), 7.52 (s, 2H, 11-C*H*), 4.80–4.72 (m, 2H, 1-C*H*), 4.65 (t, *J* = 7.5 Hz, 4H, 16-CH_2), 4.29 (t, *J* = 7.1 Hz, 4H, 10-CH_2), 2.74 (t, *J* = 7.4 Hz, 4H, 13-CH_2), 2.29–2.21 (m, 4H, 6-CH_2), 2.13–2.04 (m, 4H, 15-CH_2), 1.88–1.80 (m, 4H, 9-CH_2), 1.78–1.70 (m, 4H, 14-CH_2), 1.63–1.55 (m, 4H, 7-CH_2), 1.55–1.47 (m, 4H, 2-CH_2), 1.32–1.24 (m, 4H, 8-CH_2), 1.15 (d, *J* = 6.3 Hz, 6H, 4-CH_3), 0.86 (t, *J* = 7.5 Hz, 6H, 3-CH_3); ^{13}C NMR (126 MHz, CD_3CN) δ 173.80 (5-*C*), 150.92 (19-*C*), 147.52 (12-*C*), 146.53 (17-*C*), 128.18 (18-*C*), 122.46 (11-*C*), 72.64 (1-*C*), 62.79 (16-*C*), 50.50 (10-*C*), 34.84 (6-*C*), 31.35 (15-*C*), 30.60 (9-*C*), 29.45 (2-*C*), 26.53 (8-*C*), 26.39 (14-*C*), 25.31 (13-*C*), 25.10 (7-*C*), 19.76 (4-*C*), 9.97 (3-*C*); HRMS (ESI) calcd. for $[C_{42}H_{64}F_{12}N_8O_4P_2 - PF_6]^+$ 889.4692, found 889.4656.

*1,1'-Bis(4-(1-(6-oxo-6-((R)-sec-butoxy)hexyl)-1H-1,2,3-triazol-4-yl)butyl)-[4,4'-bipyridine]-1,1'-diium hexafluorophosphate ((R,R)-**16**)*

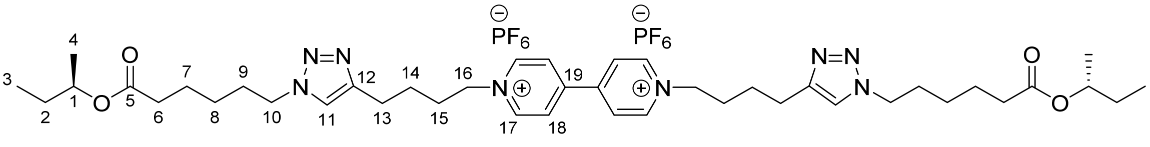

(*R*,*R*)-**16**

According to the procedure for (*S*,*S*)-**16**, the reaction of (*R*)-**26** (61.5 mg, 0.28 mmol, 2.9 equiv), **29** (58.5 mg, 96 µmol, 1.0 equiv), copper(II) sulfate pentahydrate (16 mg, 64 µmol, 0.67 equiv) and ascorbic acid (40 mg, 0.23 mmol, 2.4 equiv) in dry DMF (3 mL) afforded (*R*,*R*)-**16** (48 mg, 48%) as a white solid. $[\alpha]_D^{20}$ −10.1° (c = 1.0 g/100 mL, CH_3CN). Other spectral data were in agreement with those obtained for (*S*,*S*)-**16**.

1,1'-Bis(4-(1-(6-oxo-6-((S)-1-(4-octylphenyl)ethoxy)hexyl)-1H-1,2,3-triazol-4-yl)butyl)-[4,4'-bipyridine]-1,1'-diium hexafluorophosphate ((S,S)-17)

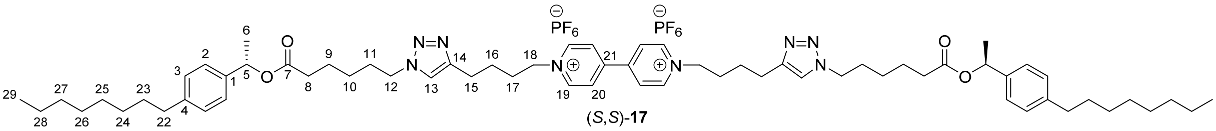

(*S*,*S*)-**17**

A flask was charged with azide (*S*)-**27** (60 mg, 0.16 mmol, 3.0 equiv), diyne **29** (33 mg, 53 µmol, 1.0 equiv) and copper(II) sulfate pentahydrate (16 mg, 64 µmol, 1.2 equiv) under an argon atmosphere. Dry DMF (3 mL) was added and the mixture was stirred at 20 °C for 30 minutes. Then, ascorbic acid (40 mg, 0.23 mmol, 4.2 equiv) was added and the mixture was stirred at 20 °C for 16 hours. The solvent was removed under high vacuum and the residue was suspended in acetonitrile (10 mL). The solid material was filtered off and the filtrate was evaporated to dryness and redissolved in acetonitrile (2 mL). Aqueous saturated ammonium hexafluorophosphate (3 mL) was added, resulting in the formation of a white precipitate. The precipitate was filtered off, washed with water (3 × 2 mL) and dried under high vacuum. The solid was redissolved in acetonitrile (2 mL) and precipitated by the addition of diethyl ether (20 mL). The precipitate was filtered off and dried under high vacuum to afford (*S*,*S*)-**17** (30 mg, 41%) as a white solid. m.p. 99–107 °C; $[\alpha]_D^{20}$ −3.7° (c = 1.0 g/100 mL, CH_3CN); ^{1}H NMR (500 MHz, CD_3CN) δ 8.88 (d, *J* = 7.0 Hz, 4H, 19-C*H*), 8.34 (d, *J* = 6.3 Hz, 4H, 20-C*H*), 7.49 (s, 2H, 13-C*H*), 7.24 (d, *J* = 8.2 Hz, 4H, 3-C*H*), 7.17 (d, *J* = 8.1 Hz, 4H, 2-C*H*), 5.76 (q, *J* = 6.6 Hz, 2H, 5-C*H*), 4.64 (t, *J* = 7.5 Hz, 4H, 18-CH_2), 4.26 (t, *J* = 7.1 Hz, 4H, 12-CH_2), 2.73 (t, *J* = 7.4 Hz, 4H, 15-CH_2), 2.61–2.52 (m, 4H, 22-CH_2), 2.34–2.26 (m, 4H, 8-CH_2), 2.11–2.03 (m, 4H, 17-CH_2), 1.87–1.78 (m, 4H, 11-CH_2), 1.78–1.70 (m, 4H, 16-CH_2), 1.62–1.52 (m, 8H, 9-CH_2 + 23-CH_2), 1.45 (d, *J* = 6.6 Hz, 3H, 6-CH_3), 1.35–1.20 (m, 24H, 10-CH_2 + 24-CH_2 + 25-CH_2 + 26-CH_2 + 27-CH_2 + 28-CH_2), 0.87 (t, *J* = 7.1 Hz, 3H, 29-CH_3); ^{13}C NMR (126 MHz, CD_3CN) δ 173.45 (7-*C*), 150.89 (21-*C*), 147.45 (14-*C*), 146.52 (19-*C*), 143.59 (4-*C*), 140.50 (1-*C*), 129.40 (2-*C*), 128.17 (20-*C*), 126.83 (3-*C*), 122.41 (13-*C*), 72.81 (5-*C*), 62.80 (18-*C*), 50.46 (12-*C*), 36.05 (22-*C*), 34.76 (8-*C*), 32.56 (27-*C*), 32.26 (23-*C*), 31.36 (17-*C*), 30.58 (11-*C*), 30.08 (24-*C* or 25-*C* or 26-*C*)*, 29.98 (24-*C* or 25-*C* or 26-*C*)*, 29.94 (24-*C* or 25-*C* or 26-*C*)*, 26.51 (10-*C*), 26.39 (16-*C*), 25.30 (15-*C*), 24.99 (9-*C*), 23.32 (28-*C*), 22.51 (6-*C*), 14.34 (29-*C*). Note: carbon signals marked with an asterisk (*) could not be assigned unambiguously; HRMS (ESI) calcd. for $[C_{66}H_{96}F_{12}N_8O_4P_2 + Na]^+$ 1377.6736, found 1377.6774.

1,1'-Bis(4-(1-(6-oxo-6-((R)-1-(4-octylphenyl)ethoxy)hexyl)-1H-1,2,3-triazol-4-yl)butyl)-[4,4'-bipyridine]-1,1'-diium hexafluorophosphate ((R,R)-17)

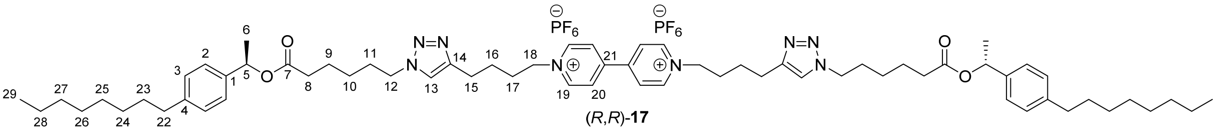

(*R*,*R*)-**17**

According to the procedure for (*S*,*S*)-**17**, the reaction of (*R*)-**27** (60 mg, 0.16 mmol, 3.0 equiv), **29** (33 mg, 53 µmol, 1.0 equiv), copper(II) sulfate pentahydrate (16 mg, 64 µmol, 1.2 equiv) and ascorbic acid (40 mg, 0.23 mmol, 4.2 equiv) in dry DMF (3 mL) afforded (*R*,*R*)-**17** (28 mg, 39%) as a white solid. $[\alpha]_D^{20}$ +3.8° (c = 1.0 g/100 mL, CH_3CN). Other spectral data were in agreement with those obtained for (*S*,*S*)-**17**.

*1,1'-Bis(4-(1-(6-oxo-6-((S)-1-phenylethoxy)hexyl)-1H-1,2,3-triazol-4-yl)butyl)-[4,4'-bipyridine]-1,1'-diium hexafluorophosphate ((S,S)-**18**)*

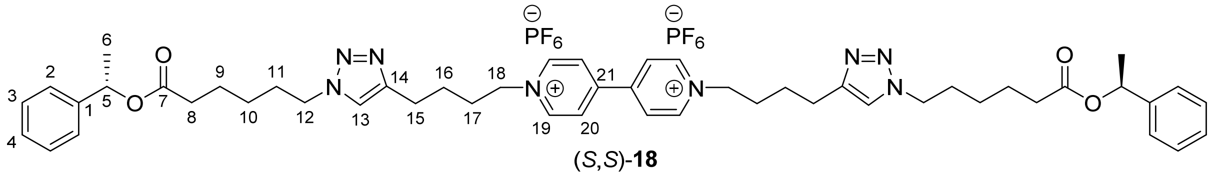

(*S*,*S*)-**18**

A flask was charged with azide (*S*)-**28** (86 mg, 0.33 mmol, 2.0 equiv), diyne **29** (100 mg, 0.16 mmol, 1.0 equiv) and copper(II) sulfate pentahydrate (20 mg, 80 µmol, 0.5 equiv) under an argon atmosphere. Dry DMF (7.5 mL) was added and the mixture was stirred at 20 °C for 30 minutes. Then, ascorbic acid (80 mg, 0.45 mmol, 2.8 equiv) was added and the mixture was stirred at 20 °C for 16 hours. The solvent was removed under high vacuum and the residue was suspended in acetonitrile (10 mL). The solid material was filtered off and the filtrate was evaporated to dryness and redissolved in acetonitrile (2 mL). Aqueous saturated ammonium hexafluorophosphate (3 mL) was added, resulting in the formation of a white precipitate. The precipitate was filtered off, washed with water (3 × 2 mL) and dried under high vacuum. The solid was redissolved in acetonitrile (2 mL) and precipitated by the addition of diethyl ether (20 mL). The precipitate was filtered off and dried under high vacuum to afford (*S*,*S*)-**18** (103 mg, 57%) as a white solid. m.p. 224–226 °C; $[\alpha]_D^{20}$ −23.9° (c = 0.209 g/100 mL, CH_3CN); ^{1}H NMR (500 MHz, CD_3CN) δ 8.88 (d, *J* = 7.0 Hz, 4H, 19-C*H*), 8.34 (d, *J* = 6.9 Hz, 4H, 20-C*H*), 7.49 (s, 2H, 13-C*H*), 7.38–7.32 (m, 8H, 2-C*H* + 3-C*H*), 7.32–7.26 (m, 2H, 4-C*H*), 5.79 (q, *J* = 6.6 Hz, 2H, 5-C*H*), 4.64 (t, *J* = 7.5 Hz, 4H, 18-CH_2), 4.27 (t, *J* = 7.1 Hz, 4H, 12-CH_2), 2.73 (t, *J* = 7.4 Hz, 4H, 15-CH_2), 2.36–2.25 (m, 4H, 8-CH_2), 2.11–2.03 (m, 4H, 17-CH_2), 1.87–1.79 (m, 4H, 11-CH_2), 1.74 (p, *J* = 7.5 Hz, 4H, 16-CH_2), 1.59 (p, *J* = 7.4 Hz, 4H, 9-CH_2), 1.47 (d, *J* = 6.6 Hz, 6H, 6-CH_3), 1.31–1.21 (m, 4H, 10-CH_2); ^{13}C NMR (126 MHz, CD_3CN) δ 173.44 (7-*C*), 150.90 (21-*C*), 147.26 (14-*C*), 146.52 (19-*C*), 143.35 (1-*C*), 129.43 (2-*C*), 128.66 (4-*C*), 128.17 (20-*C*), 126.78 (3-*C*), 122.72 (13-*C*), 72.90 (5-*C*), 62.76 (18-*C*), 50.67 (12-*C*), 34.73 (8-*C*), 31.31 (17-*C*), 30.50 (11-*C*), 26.48 (10-*C*), 26.31 (16-*C*), 25.17 (15-*C*), 24.97 (9-*C*), 22.60 (6-*C*); HRMS (ESI) calcd. for $[C_{50}H_{64}F_{12}N_8O_4P_2 - PF_6]^+$ 985.4692, found 985.4673.

*1,1'-Bis(4-(1-(6-oxo-6-((R)-1-phenylethoxy)hexyl)-1H-1,2,3-triazol-4-yl)butyl)-[4,4'-bipyridine]-1,1'-diium hexafluorophosphate ((R,R)-**18**)*

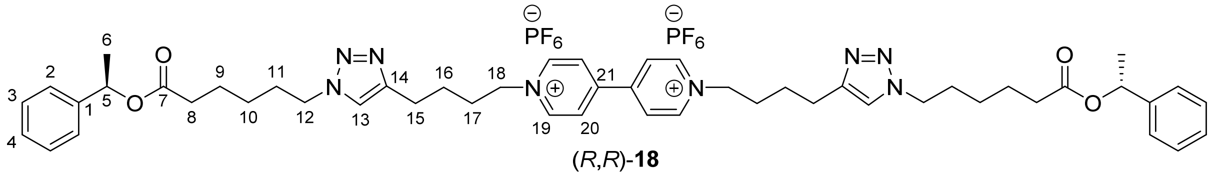

(*R*,*R*)-**18**

According to the procedure for (*S*,*S*)-**18**, the reaction of (*R*)-**28** (86 mg, 0.33 mmol, 2.0 equiv), **29** (100 mg, 0.16 mmol, 1.0 equiv), copper(II) sulfate pentahydrate (20 mg, 80 µmol, 0.5 equiv) and ascorbic acid (80 mg, 0.45 mmol, 2.8 equiv) in dry DMF (7.5 mL) afforded (*R*,*R*)-**18** (121 mg, 65%) as a white solid. $[\alpha]_D^{20}$ +22.2° (c = 0.209 g/100 mL, CH_3CN). Other spectral data were in agreement with those obtained for (*S*,*S*)-**18**.

2.2.2. Resolution of secondary alcohol *rac*-21

Analytical chiral HPLC separation for compound **21**

• The sample is dissolved in dichloromethane, injected on the chiral column, and detected with an UV detector at 220 nm and with a polarimeter. The flow-rate is 1 mL/min.

Column	Mobile Phase	t1	k1	t2	k2	α	Rs
**Chiralcel OD-3**	Heptane / 2-PrOH (97/3)	7.61	1.58	8.73	1.96	1.24	3.27

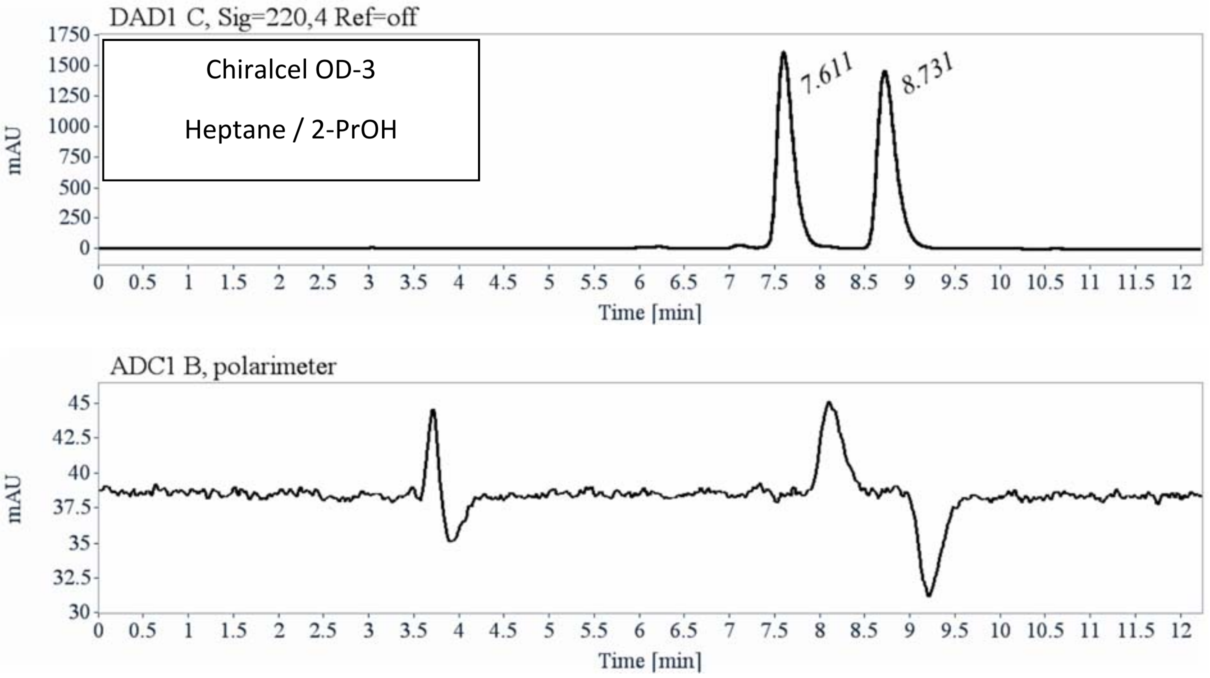

*Fig. S1. Chiral HPLC chromatogram of rac-**21** with UV detection (top) and polarimeter detection (bottom).*

RT [min]	Area	Area%	Capacity Factor	Enantioselectivity	Resolution (USP)
7.61	20460	49.57	1.58		
8.73	20817	50.43	1.96	1.24	3.27
Sum	41276	100.00			

Preparative separation for compound **21**:

• Sample preparation: 680 mg of compound **21** was dissolved in 40.5 mL of a mixture of hexane and 2-PrOH (95/5).

• Chromatographic conditions: Lux-Cellulose-1 (250 x 10 mm), hexane / 2-PrOH (98.75/1.25) as mobile phase, flow-rate = 5 mL/min, UV detection at 220 nm.

• Injections (stacked): 135 times 300 µL, every 6 minutes.

• First fraction: 309 mg of the first eluted enantiomer with ee > 99.5 %

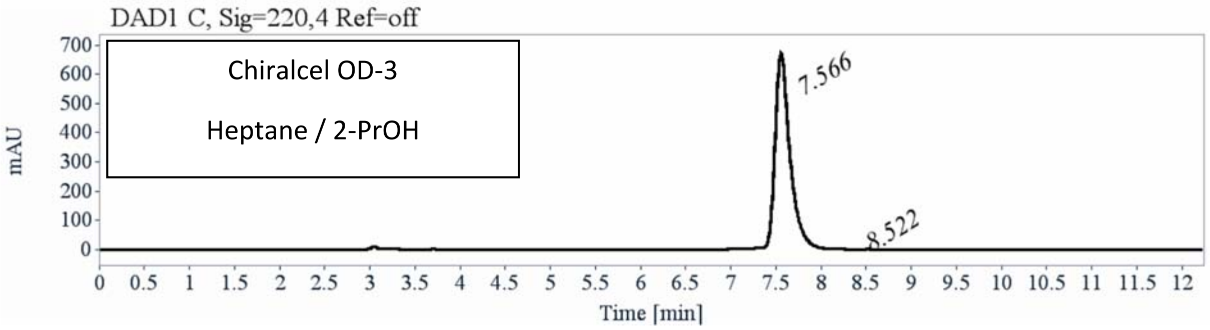

*Fig. S2. Chiral HPLC chromatogram of the first eluted enantiomer (R)-**21**.*

RT [min]	Area	Area%
7.57	7740	99.96
8.52	3	0.04
Sum	7742	100.00

• Second fraction: 307 mg of the second eluted enantiomer with ee > 99.5 %

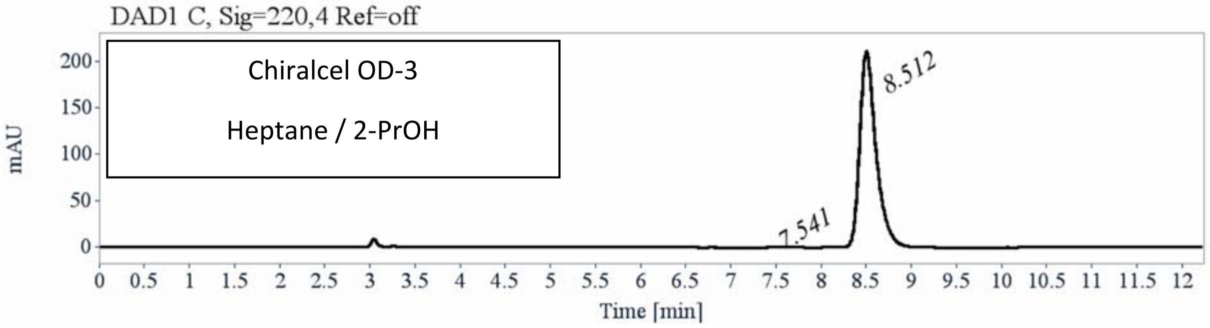

*Fig. S3. Chiral HPLC chromatogram of the second eluted enantiomer (S)-**21**.*

RT [min]	Area	Area%
7.54	4	0.14
8.51	2670	99.86
Sum	2674	100.00

• Intermediate fraction: 63 mg

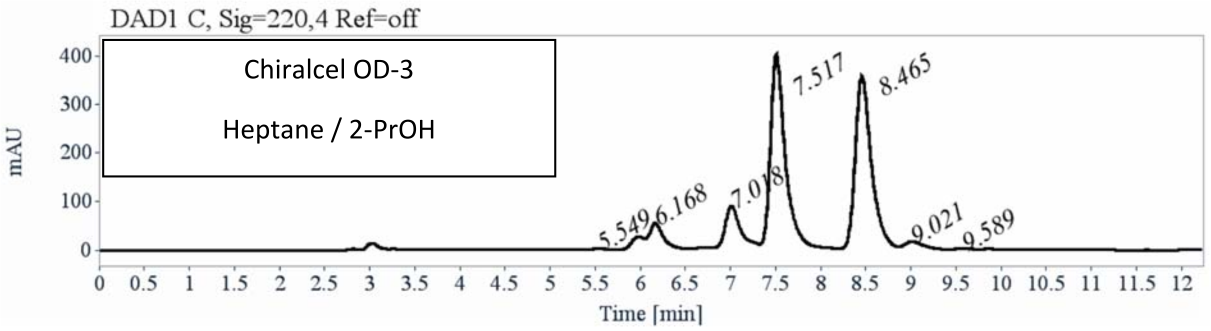

Fig. S4. Chiral HPLC chromatogram of the intermediate fraction.

<u>Optical rotations</u>

Optical rotations were measured on a Jasco P-2000 polarimeter with a halogen lamp (589, 578, 546, 436, 405, 365 and 325 nm), in a 10 cm cell, thermostated at 25°C with a Peltier controlled cell holder.

*Table S1. Optical rotations of both enantiomers of **21** measured at different wavelengths.*

λ (nm)	Compound **21**<br>First eluted enantiomer on Chiralcel OD-3<br>$[\alpha]_{\lambda}^{25}$ (CH_2Cl_2, c = 0.53)	Compound **21**<br>Second eluted enantiomer on Chiralcel OD-3<br>$[\alpha]_{\lambda}^{25}$ (CH_2Cl_2, c = 0.59)
589	+ 29	- 29
578	+ 30	- 30
546	+ 35	- 35
436	+ 60	- 60
405	+ 74	- 74
365	+ 102	- 102
325	+ 131	- 131

Electronic Circular Dichroism

ECD and UV spectra were measured on a JASCO J-815 spectrometer equipped with a JASCO Peltier cell holder PTC-423 to maintain the temperature at 25.0 ± 0.2 °C. A CD quartz cell of 1 mm of optical pathlength was used. The CD spectrometer was purged with nitrogen before recording each spectrum, which was baseline subtracted.

The baseline was always measured for the same solvent and in the same cell as the samples.

The spectra are presented without smoothing and further data processing.

first eluted enantiomer : green solid line, concentration = 0.356 mmol.L^{-1} in acetonitrile.

second eluted enantiomer : red dotted line, concentration = 0.351 mmol.L^{-1} in acetonitrile.

Acquisition parameters: 0.1 nm as intervals, scanning speed 50 nm/min, band width 2 nm, and 3 accumulations per sample.

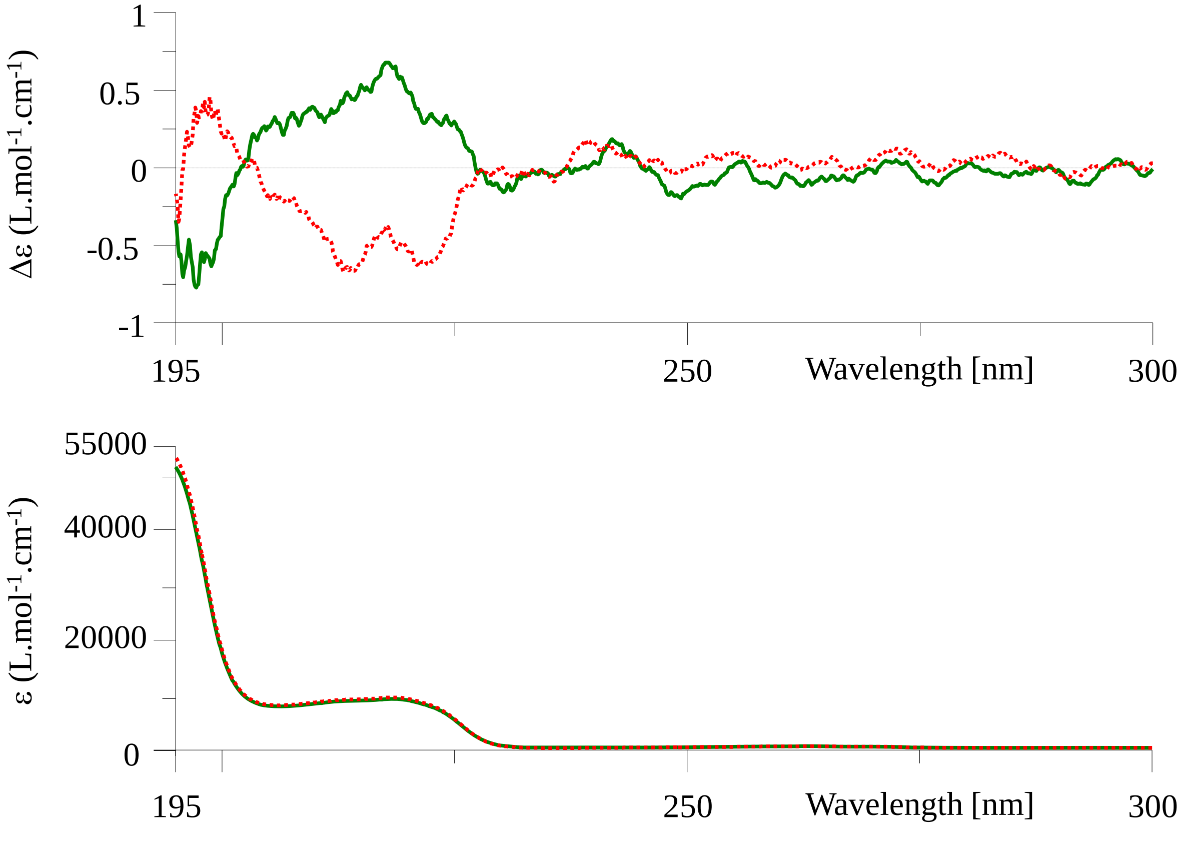

*Fig. S5. ECD (top) and UV-vis (bottom) spectra of both enantiomers of **21**. First eluted enantiomer = green solid line; second eluted enantiomer = red dotted line.*

Comparison with 1-phenylethanol (compound **22**).

Column	Mobile Phase	t1	k1	t2	k2	α	Rs
**Chiralcel OD-3**	Heptane / 2-PrOH (90/10)	6.32 (*R*, +)	1.14	7.16 (*S*, -)	1.43	1.25	3.72

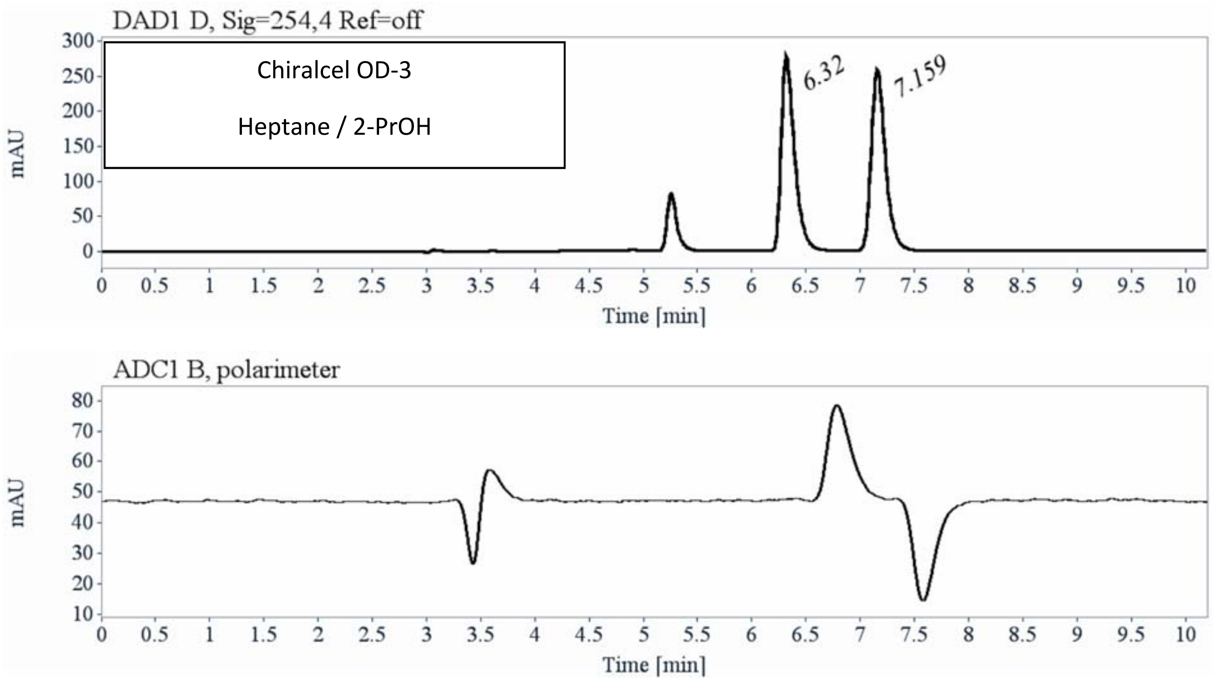

*Fig. S6. Chiral HPLC chromatogram of 1-phenylethanol (rac-**22**) with UV detection (top) and polarimeter detection (bottom).*

RT [min]	Area	Area%	Capacity Factor	Enantioselectivity	Resolution (USP)
6.32	2379	50.06	1.14		
7.16	2373	49.94	1.43	1.25	3.72
Sum	4752	100.00			

Optical rotations

Optical rotations were measured on a Jasco P-2000 polarimeter with a sodium lamp (589 nm), a halogen lamp (578, 546, 436, 405, 365 and 325 nm), in a 10 cm cell, thermostated at 25°C with a Peltier controlled cell holder.

*Table S2. Optical rotations of both enantiomers of 1-phenylethanol (**22**) measured at different wavelengths.*

λ (nm)	(*R*)-enantiomer (*R*)-**22**<br>first eluted on Chiralcel OD-3<br>$[\alpha]_{\lambda}^{25}$ (CH_2Cl_2, c =1.08)	(*S*)-enantiomer (*S*)-**22**<br>second eluted on Chiralcel OD-3<br>$[\alpha]_{\lambda}^{25}$ (CH_2Cl_2, c =1.1)
589	+50	-50
578	+52	-52
546	+60	-60
436	+103	-103
405	+124	-124
365	+169	-169
325	+218	-216

Comparison of molar specific rotation for compound **21** and 1-phenylethanol (**22**)

*Table S3. Molar specific rotations of (R)-**22** and the first eluted enantiomer of **21** measured at different wavelengths.*

λ (nm)	(*R*)-enantiomer of 1-phenylethanol (*R*)-**22**<br>first eluted on Chiralcel OD-3<br>$[\phi]_{\lambda}^{25}$ (CH_2Cl_2)	Compound **21**<br>first eluted on Chiralcel OD-3<br>$[\phi]_{\lambda}^{25}$ (CH_2Cl_2)
589	+ 61	+ 68
578	+ 64	+ 70
546	+ 73	+ 82
436	+ 126	+ 141
405	+ 152	+ 173
365	+ 207	+ 239
325	+ 266	+ 307

Electronic Circular Dichroism of 1-phenylethanol (**22**)

first eluted enantiomer on Chiralcel OD-3: green solid line, concentration = 2.59 mmol.L^{-1} in acetonitrile.

second eluted enantiomer on Chiralcel OD-3: red dotted line, concentration = 2.56 mmol.L^{-1} in acetonitrile.

Acquisition parameters: 0.1 nm as intervals, scanning speed 50 nm/min, band width 1 nm, and 3 accumulations per sample.

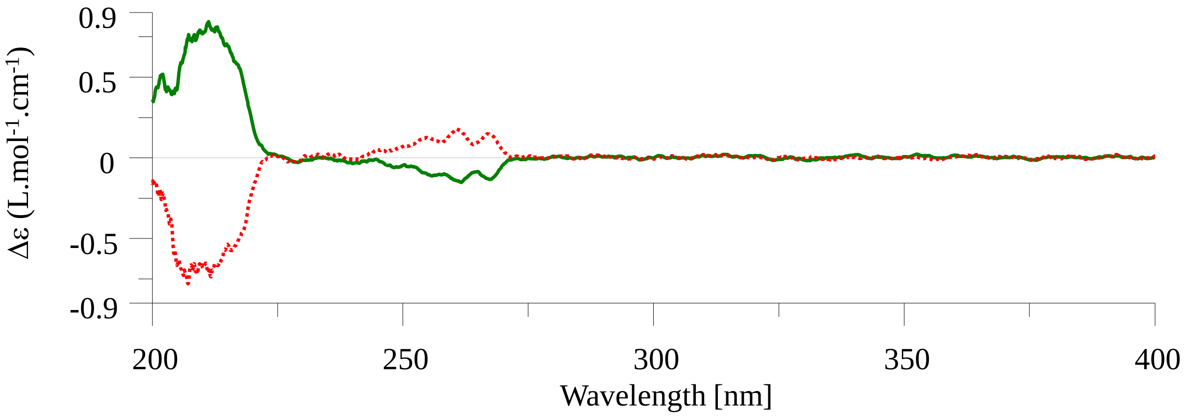

*Fig. S7. ECD (top) spectra of both enantiomers of **22**. First eluted enantiomer = green solid line; second eluted enantiomer = red dotted line.*

The (*R*)-1-phenylethanol ((*R*)-**22**) is the first eluted enantiomer on Chiralcel OD-3 with a mixture heptane / 2-PrOH as mobile phase, has a positive specific rotation between 325 and 589 nm, and shows a very weak positive ECD signal between 200 and 225 nm.

The first eluted enantiomer of compound **21** on Chiralcel OD-3 has a positive specific rotation between 325 and 589 nm, and shows a very weak positive ECD signal between 200 and 225 nm. Therefore, the first eluted enantiomer of compound **21** is (*R*)-**21** and the second eluted enantiomer is (*S*)-**21**.

2.2.3. Fluorescence host-guest titrations

Titration of host H_2***3*** *with guest* ***13***

A solution of host H_2**3** (2 mL, c = 10^{-6} M in $CHCl_3$/CH_3CN, 1:1, v/v, 298 K) was titrated with a solution containing guest **13** (c = 4 × 10^{-5} M) and host H_2**3** (c = 10^{-6} M) according to Table S4. Each combination of host and guest was titrated in duplicate. The fluorescence intensities (λ_{ex} = 416 nm) of the host at λ = 647 and 713 nm were plotted as a function of the concentration of guest. The resulting data files were uploaded to the web application supramolecular.org, which could fit the data to 1:1 host-guest binding models.[S6–S7] The calculated binding constants, fitting errors, and hyperlinks are presented in Tables S5–6.

Table S4. Fluorescence titration scheme for guest ***13****.*

Exp #	$V_{initial}$ (µL)	V_{added} (µL)	V_{final} (µL)	[host] (× 10^{-6} M)	[guest] (× 10^{-6} M)
1	2000	0	2000	1	0.00
2	2000	5	2005	1	0.0998
3	2005	5	2010	1	0.199
4	2010	5	2015	1	0.298
5	2015	5	2020	1	0.396
6	2020	5	2025	1	0.494
7	2025	5	2030	1	0.591
8	2030	5	2035	1	0.688
9	2035	5	2040	1	0.784
10	2040	5	2045	1	0.880
11	2045	5	2050	1	0.976
12	2050	5	2055	1	1.07
13	2055	5	2060	1	1.17
14	2060	5	2065	1	1.26
15	2065	5	2070	1	1.35
16	2070	10	2080	1	1.54
17	2080	10	2090	1	1.72
18	2090	10	2100	1	1.90
19	2100	50	2150	1	2.79
20	2150	50	2200	1	3.64
21	2200	50	2250	1	4.44
22	2250	250	2500	1	8.00
23	2500	500	3000	1	13.3

Table S5. Association constants of (R,R,R,R)-H_2***3*** *with* ***13****.*

Entry	Host	Guest	K_{assoc} (× 10^6 M^{-1})	Fitting error (%)	URL
1	(*R*,*R*,*R*,*R*)-H_2**3**	**13**	1.89	2.9	http://app.supramolecular.org/bindfit/view/49353942-9920-4cc0-8ad0-684fd4f7d8b3
2	(*R*,*R*,*R*,*R*)-H_2**3**	**13**	1.90	2.5	http://app.supramolecular.org/bindfit/view/0c2aa503-994e-45c4-8e76-4375cb7d3dd5
**AVERAGE**	(*R*,*R*,*R*,*R*)-H_2**3**	**13**	1.9		

Table S6. Association constants of (S,S,S,S)-H_2***3*** *with* ***13****.*

Entry	Host	Guest	K_{assoc} (× 10^6 M^{-1})	Fitting error (%)	URL
1	(*S*,*S*,*S*,*S*)-H_2**3**	**13**	1.61	3.2	http://app.supramolecular.org/bindfit/view/20f86da2-ce40-4d14-b12a-0be218fc75ba
2	(*S*,*S*,*S*,*S*)-H_2**3**	**13**	1.67	2.7	http://app.supramolecular.org/bindfit/view/66104552-829f-4d7e-984b-4578e3625ce9
**AVERAGE**	(*S*,*S*,*S*,*S*)-H_2**3**	**13**	1.6		

Titration of host $H_2$1 or $H_2$3 with guest 15

A solution of host **$H_2$1** or **$H_2$3** (2 mL, c = 10^{-6} M in $CHCl_3/CH_3CN$, 1:1, v/v, 298 K) was titrated with a solution containing guest **15** (c = 8 × 10^{-5} M) and host **$H_2$1** or **$H_2$3** (c = 10^{-6} M) according to Table S7. For the experiments involving host **$H_2$3**, in order to reach the chemical equilibrium, we employed a waiting time of 90 seconds between the addition of another aliquot of the host-guest solution and the acquisition of the spectrum. Each combination of host and guest was titrated in duplicate. The fluorescence intensities (λ_{ex} = 416 nm) of host **$H_2$1** (at λ = 649 and 713 nm) or **$H_2$3** (at λ = 647 and 713 nm) were plotted as a function of the concentration of guest. The resulting data files were uploaded to the web application supramolecular.org, which could fit the data to 1:1 host-guest binding models.[S6–S7] The calculated binding constants, fitting errors, and hyperlinks are presented in Tables S8–13.

*Table S7. Fluorescence titration scheme for guest **15**.*

**Exp #**	**$V_{initial}$ (μL)**	**V_{added} (μL)**	**V_{final} (μL)**	**[host] (× 10^{-6} M)**	**[guest] (× 10^{-6} M)**
1	2000	0	2000	1	0.00
2	2000	5	2005	1	0.1996
3	2005	5	2010	1	0.398
4	2010	5	2015	1	0.596
5	2015	5	2020	1	0.792
6	2020	5	2025	1	0.988
7	2025	5	2030	1	1.182
8	2030	5	2035	1	1.376
9	2035	5	2040	1	1.568
10	2040	5	2045	1	1.760
11	2045	5	2050	1	1.952
12	2050	5	2055	1	2.14
13	2055	5	2060	1	2.34
14	2060	5	2065	1	2.52
15	2065	5	2070	1	2.70
16	2070	10	2080	1	3.08
17	2080	10	2090	1	3.44
18	2090	10	2100	1	3.80
19	2100	50	2150	1	5.58
20	2150	50	2200	1	7.28
21	2200	50	2250	1	8.88
22	2250	250	2500	1	16.0
23	2500	500	3000	1	26.6

*Table S8. Association constants of **$H_2$1** with (R,R)-**15**.*

**Entry**	**Host**	**Guest**	**K_{assoc} (× 10^6 M^{-1})**	**Fitting error (%)**	**URL**
1	**$H_2$1**	(*R*,*R*)-**15**	6.60	3.4	http://app.supramolecular.org/bindfit/view/0ad9973f-5d7e-4cc5-9edd-fb60be2e3261
2	**$H_2$1**	(*R*,*R*)-**15**	10.6	13.9	http://app.supramolecular.org/bindfit/view/76fc1c2f-de55-4e2b-9f4d-ee033a72286b
**AVERAGE**	**$H_2$1**	(*R*,*R*)-**15**	8.6		

*Table S9. Association constants of (R,R,R,R)-**H₂3** with (R,R)-**15**.*

Entry	Host	Guest	K_{assoc} (× 10^7 M^{-1})	Fitting error (%)	URL
1	(*R*,*R*,*R*,*R*)-**$H_2$3**	(*R*,*R*)-**15**	2.14	17.4	http://app.supramolecular.org/bindfit/view/9e9f25b7-f4b0-4c6e-9b44-c8d7815a8ac3
2	(*R*,*R*,*R*,*R*)-**$H_2$3**	(*R*,*R*)-**15**	1.59	3.1	http://app.supramolecular.org/bindfit/view/ae4e5e99-04fc-4329-ab7c-dd83c7e5e60b
**AVERAGE**	(*R*,*R*,*R*,*R*)-**$H_2$3**	(*R*,*R*)-**15**	1.9		

*Table S10. Association constants of (S,S,S,S)-**$H_2$3** with (R,R)-**15**.*

Entry	Host	Guest	K_{assoc} (× 10^7 M^{-1})	Fitting error (%)	URL
1	(*S*,*S*,*S*,*S*)-**$H_2$3**	(*R*,*R*)-**15**	2.23	10.0	http://app.supramolecular.org/bindfit/view/bda108bd-f4b7-4004-ab08-582610ac8433
2	(*S*,*S*,*S*,*S*)-**$H_2$3**	(*R*,*R*)-**15**	1.99	11.3	http://app.supramolecular.org/bindfit/view/61469ae0-4bf2-4963-88d6-fa07088b17c4
**AVERAGE**	(*S*,*S*,*S*,*S*)-**$H_2$3**	(*R*,*R*)-**15**	2.1		

*Table S11. Association constants of **$H_2$1** with (S,S)-**15**.*

Entry	Host	Guest	K_{assoc} (× 10^6 M^{-1})	Fitting error (%)	URL
1	**$H_2$1**	(*S*,*S*)-**15**	11.7	12.5	http://app.supramolecular.org/bindfit/view/f60abd93-80f6-4d16-a327-d1cacef1b353
2	**$H_2$1**	(*S*,*S*)-**15**	9.68	11.8	http://app.supramolecular.org/bindfit/view/7fd76f23-8e44-4961-9291-d02c0fc42bcd
**AVERAGE**	**$H_2$1**	(*S*,*S*)-**15**	10.7		

*Table S12. Association constants of (R,R,R,R)-**$H_2$3** with (S,S)-**15**.*

Entry	Host	Guest	K_{assoc} (× 10^7 M^{-1})	Fitting error (%)	URL
1	(*R*,*R*,*R*,*R*)-**$H_2$3**	(*S*,*S*)-**15**	2.22	14.7	http://app.supramolecular.org/bindfit/view/228010a3-4d2c-4723-b00b-6a17ecf52739
2	(*R*,*R*,*R*,*R*)-**$H_2$3**	(*S*,*S*)-**15**	2.49	18.1	http://app.supramolecular.org/bindfit/view/6ab837e1-666c-4cb5-86ef-118ab53210f3
**AVERAGE**	(*R*,*R*,*R*,*R*)-**$H_2$3**	(*S*,*S*)-**15**	2.4		

*Table S13. Association constants of (S,S,S,S)-**$H_2$3** with (S,S)-**15**.*

Entry	Host	Guest	K_{assoc} (× 10^7 M^{-1})	Fitting error (%)	URL
1	(*S*,*S*,*S*,*S*)-**$H_2$3**	(*S*,*S*)-**15**	1.57	7.4	http://app.supramolecular.org/bindfit/view/6136713d-5f82-4b8d-906d-30c01eaf89a2
2	(*S*,*S*,*S*,*S*)-**$H_2$3**	(*S*,*S*)-**15**	1.73	9.3	http://app.supramolecular.org/bindfit/view/2012314f-4dd1-43d3-a39e-b5c98163de11
**AVERAGE**	(*S*,*S*,*S*,*S*)-**$H_2$3**	(*S*,*S*)-**15**	1.7		

2.2.4. Fluorescence threading studies

General information

Host (H)-guest (G) complexation (threading) and decomplexation (dethreading) are described by equations 1–3.

$$H + G \xrightarrow{k_{on}} HG \tag{1}$$

$$HG \xrightarrow{k_{off}} H + G \tag{2}$$

$$K_{assoc} = \frac{k_{on}}{k_{off}} \tag{3}$$

Although complexation is reversible, the initial stages of the threading process follow second-order kinetics according to equations 4–6.

$$\frac{\partial[HG]}{\partial t} = k_{on}[H][G] \tag{4}$$

If we assure that the initial concentrations of host and guest are equal, *i.e.* $[H]_0 = [G]_0$, and $[HG] = [H]_0 - [H]$, then

$$[H]_0 - \frac{\partial[H]}{\partial t} = k_{on}[H]^2 \tag{5}$$

The solution to this differential equation is

$$\frac{1}{[H]} = \frac{1}{[H]_0} + k_{on}t \tag{6}$$

By plotting the inverse concentration of host as a function of time, a linear fit is obtained in which the rate constant k_{on} equals the slope.

Threading of host $H_2$3 onto guest 14.
A solution of **$H_2$3** (2 mL, c = 10^{-5} M in $CHCl_3/CH_3CN$, 1:1, v/v, 298 K) was added to a 1 cm quartz cuvette and the cuvette was placed inside the fluorescence spectrometer. The fluorescence intensity of **$H_2$3** (λ_{ex} = 512 nm, λ_{em} = 647 nm) was monitored as a function of time (10 datapoints per second). At t ≈ 40 s, the cuvette was removed from the spectrometer. At t ≈ 50 s, a solution of **14** (10 µL, c = 2 × 10^{-3} M, 1 equiv) was added to the solution containing **$H_2$3**. The contents of the cuvette were shaken vigorously for 5 seconds. At t ≈ 60 s, the cuvette was placed back inside the spectrometer. At t = 600 s, the experiment was stopped. The fluorescence intensity of the host was normalized to the average fluorescence intensity during the initial 40 seconds. Each combination of host and guest was tested in triplicate. The kinetic plots for the threading of (*R*,*R*,*R*,*R*)-**$H_2$3** onto **14** are depicted in Fig. S8–S9 and the results are summarized in Table S14. The kinetic plots for the threading of (*S*,*S*,*S*,*S*)-**$H_2$3** onto **14** are depicted in Fig. S10–S11 and the results are summarized in Table S15.

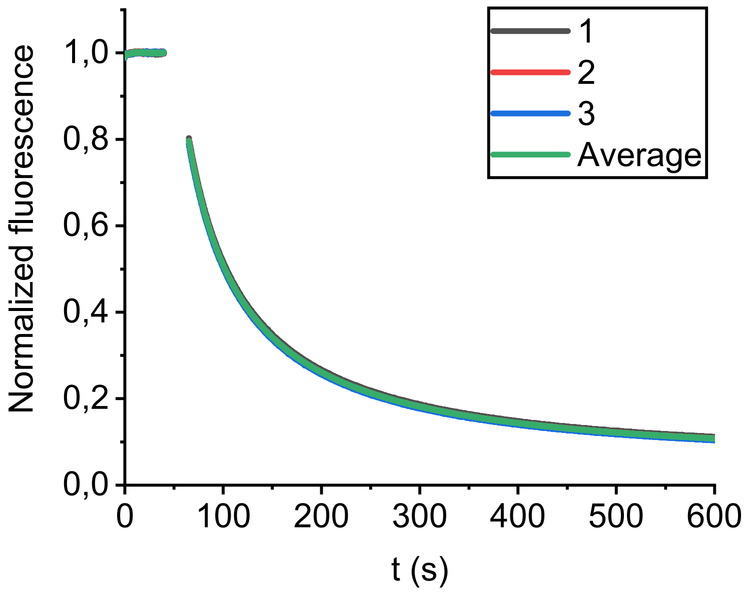

*Fig. S8. Normalized fluorescence intensity of host (R,R,R,R)-**$H_2$3** as a function of time after the addition (t = 50 s) of 1 equivalent of guest **14** (c = 10^{-5} M in $CHCl_3/CH_3CN$, 1:1, v/v, 298 K). The experiment was performed in triplicate.*

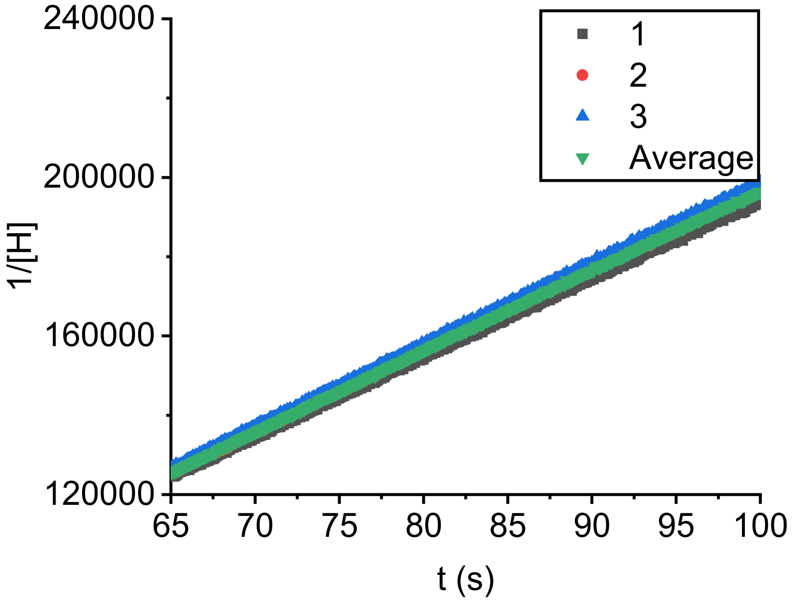

*Fig. S9. Second-order kinetics plots for the complexation of host (R,R,R,R)-**$H_2$3** to guest **14** (c = 10^{-5} M in $CHCl_3/CH_3CN$, 1:1, v/v, 298 K).*

*Table S14. Kinetic data for the threading of host (R,R,R,R)-**$H_2$3** onto guest **14**.*

**Entry**	**Host**	**Guest**	**k_{on} (× 10^3 $M^{-1}\cdot s^{-1}$)**	**R^2**
1	(*R*,*R*,*R*,*R*)-**$H_2$3**	**14**	1.99	0.999
2	(*R*,*R*,*R*,*R*)-**$H_2$3**	**14**	2.05	0.999
3	(*R*,*R*,*R*,*R*)-**$H_2$3**	**14**	2.06	0.999
**AVERAGE**	(*R*,*R*,*R*,*R*)-**$H_2$3**	**14**	2.03	0.999

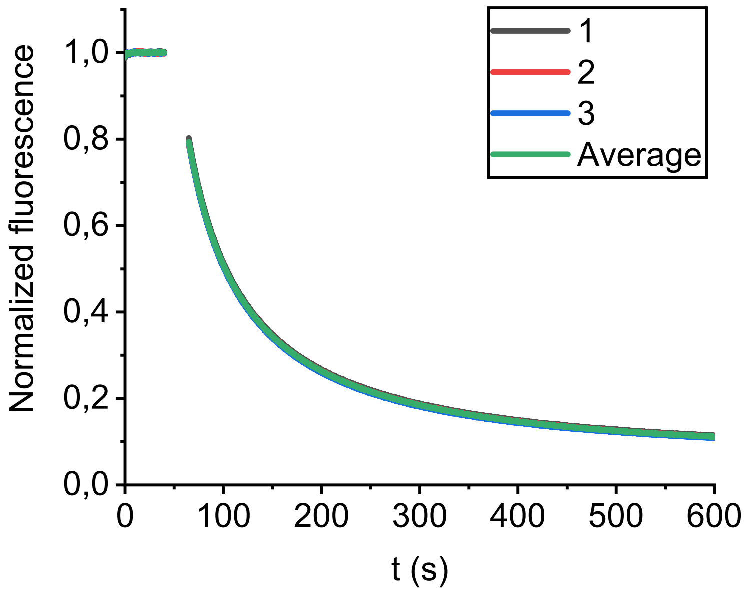

*Fig. S10. Normalized fluorescence intensity of host (S,S,S,S)-**H₂3** as a function of time after the addition (t = 50 s) of 1 equivalent of guest **14** (c = 10^{-5} M in $CHCl_3/CH_3CN$, 1:1, v/v, 298 K). The experiment was performed in triplicate.*

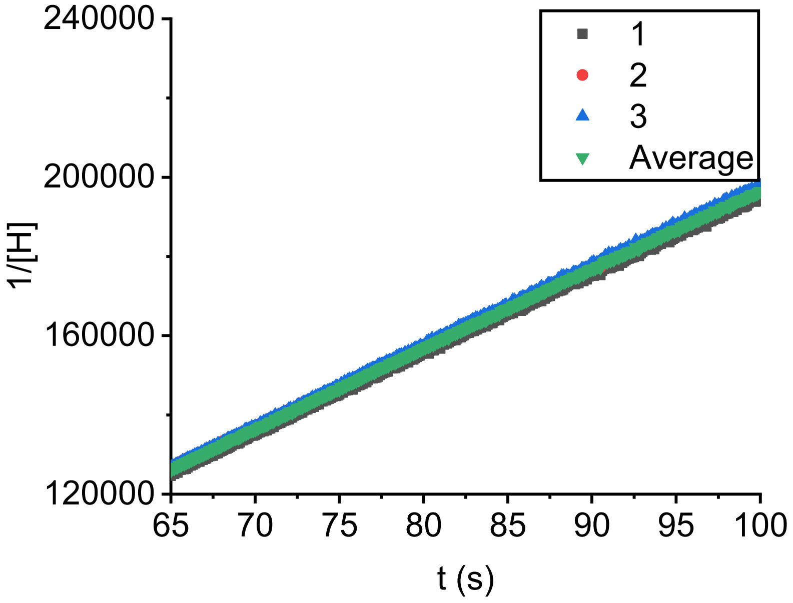

*Fig. S11. Second-order kinetics plots for the complexation of host (S,S,S,S)-**H₂3** to guest **14** (c = 10^{-5} M in $CHCl_3/CH_3CN$, 1:1, v/v, 298 K).*

*Table S15. Kinetic data for the threading of host (S,S,S,S)-**H₂3** onto guest **14**.*

**Entry**	**Host**	**Guest**	**k_{on} (× 10^3 $M^{-1}\cdot s^{-1}$)**	**R^2**
1	(*S*,*S*,*S*,*S*)-**$H_2$3**	**14**	2.00	0.999
2	(*S*,*S*,*S*,*S*)-**$H_2$3**	**14**	2.00	0.999
3	(*S*,*S*,*S*,*S*)-**$H_2$3**	**14**	2.03	0.999
**AVERAGE**	(*S*,*S*,*S*,*S*)-**$H_2$3**	**14**	2.01	0.999

Threading of host $H_2$3 onto guest 15.

A solution of **$H_2$3** (2 mL, c = 2.5 × 10^{-7} M in $CHCl_3/CH_3CN$, 1:1, v/v, 298 K) was added to a 1 cm quartz cuvette and the cuvette was placed inside the fluorescence spectrometer. The fluorescence intensity of **$H_2$3** (λ_{ex} = 416 nm, λ_{em} = 647 nm) was monitored as a function of time (10 datapoints per second). At t ≈ 40 s, the cuvette was removed from the spectrometer. At t ≈ 50 s, a solution of **15** (10 µL, c = 5 × 10^{-5} M, 1 equiv) was added to the solution containing **$H_2$3**. The contents of the cuvette were shaken vigorously for 5 seconds. At t ≈ 60 s, the cuvette was placed back inside the spectrometer. At t = 600 s, the experiment was stopped. The fluorescence intensity of the host was normalized to the average fluorescence intensity during the initial 40 seconds. Each combination of host and guest was tested in triplicate. The kinetic plots for the threading of (*R*,*R*,*R*,*R*)-**$H_2$3** onto (*R*,*R*)-**15** are depicted in Fig. S12–S13 and the results are summarized in Table S16. The kinetic plots for the threading of (*S*,*S*,*S*,*S*)-**$H_2$3** onto (*R*,*R*)-**15** are depicted in Fig. S14–S15 and the results are summarized in Table S17. The kinetic plots for the threading of (*R*,*R*,*R*,*R*)-**$H_2$3** onto (*S*,*S*)-**15** are depicted in Fig. S16–S17 and the results are summarized in Table S18. The kinetic plots for the threading of (*S*,*S*,*S*,*S*)-**$H_2$3** onto (*S*,*S*)-**15** are depicted in Fig. S18–S19 and the results are summarized in Table S19.

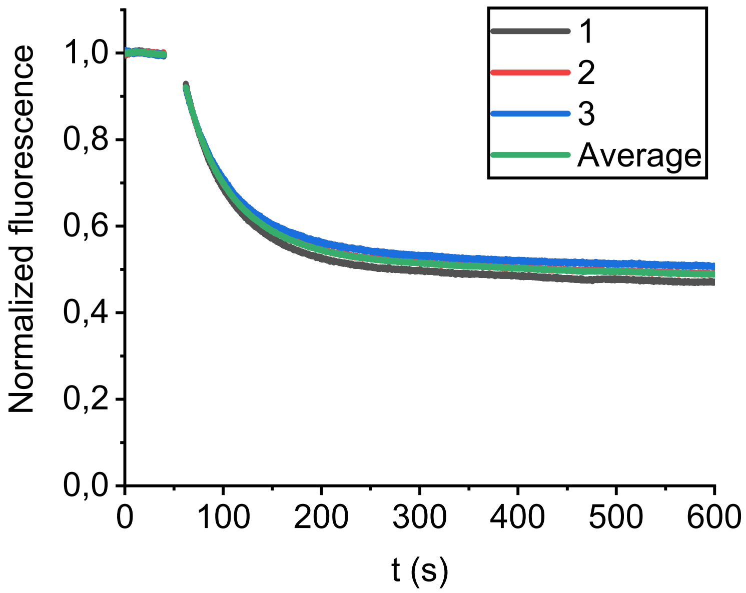

*Fig. S12. Normalized fluorescence intensity of host (R,R,R,R)-**H₂3** as a function of time after the addition (t = 50 s) of 1 equivalent of guest (R,R)-**15** (c = 2.5 × 10^{-7} M in $CHCl_3$/CH_3CN, 1:1, v/v, 298 K). The experiment was performed in triplicate.*

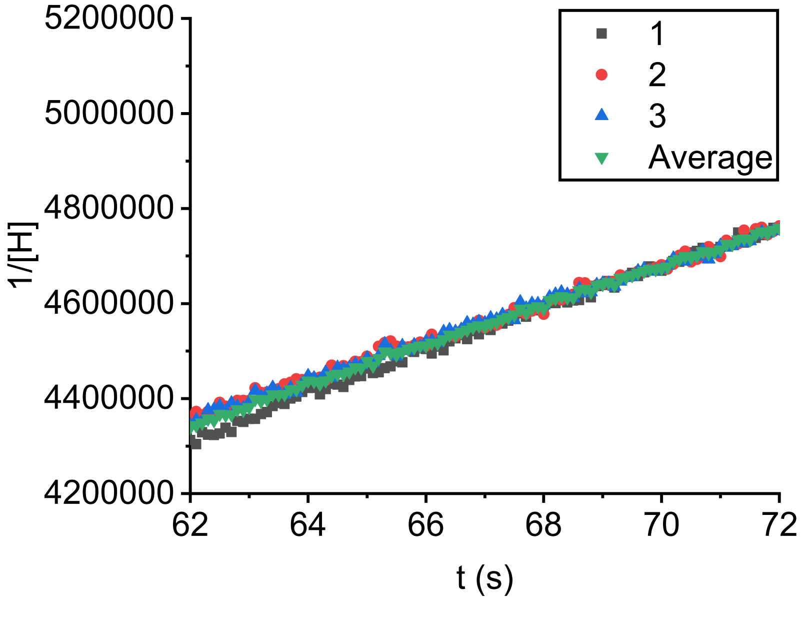

*Fig. S13. Second-order kinetics plots for the complexation of host (R,R,R,R)-**H₂3** to guest (R,R)-**15** (c = 2.5 × 10^{-7} M in $CHCl_3$/CH_3CN, 1:1, v/v, 298 K).*

*Table S16. Kinetic data for the threading of host (R,R,R,R)-**H₂3** onto guest (R,R)-**15**.*

**Entry**	**Host**	**Guest**	k_{on} **(× 10^4 $M^{-1}\cdot s^{-1}$)**	R^2
1	(*R*,*R*,*R*,*R*)-**$H_2$3**	(*R*,*R*)-**15**	4.52	0.997
2	(*R*,*R*,*R*,*R*)-**$H_2$3**	(*R*,*R*)-**15**	3.96	0.994
3	(*R*,*R*,*R*,*R*)-**$H_2$3**	(*R*,*R*)-**15**	4.01	0.995
**AVERAGE**	(*R*,*R*,*R*,*R*)-**$H_2$3**	(*R*,*R*)-**15**	4.16	0.998

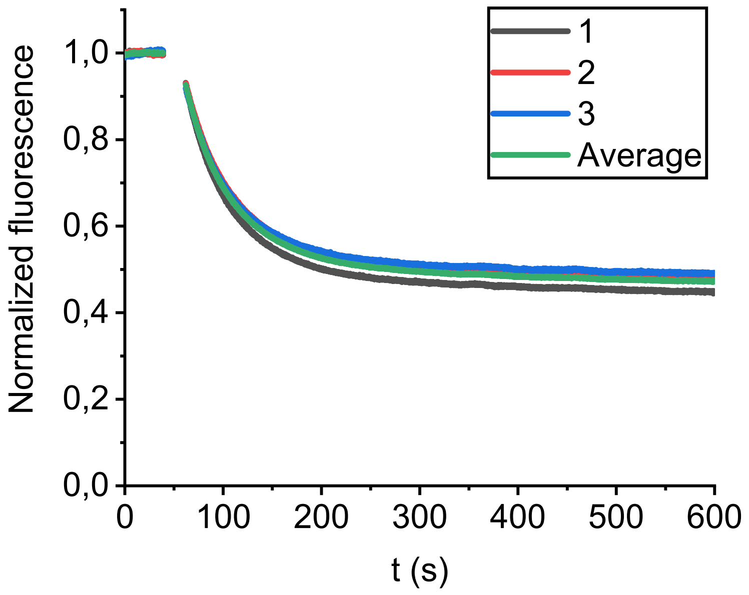

*Fig. S14. Normalized fluorescence intensity of host (S,S,S,S)-**$H_2$3** as a function of time after the addition (t = 50 s) of 1 equivalent of guest (R,R)-**15** (c = 2.5 × 10^{-7} M in $CHCl_3$/CH_3CN, 1:1, v/v, 298 K). The experiment was performed in triplicate.*

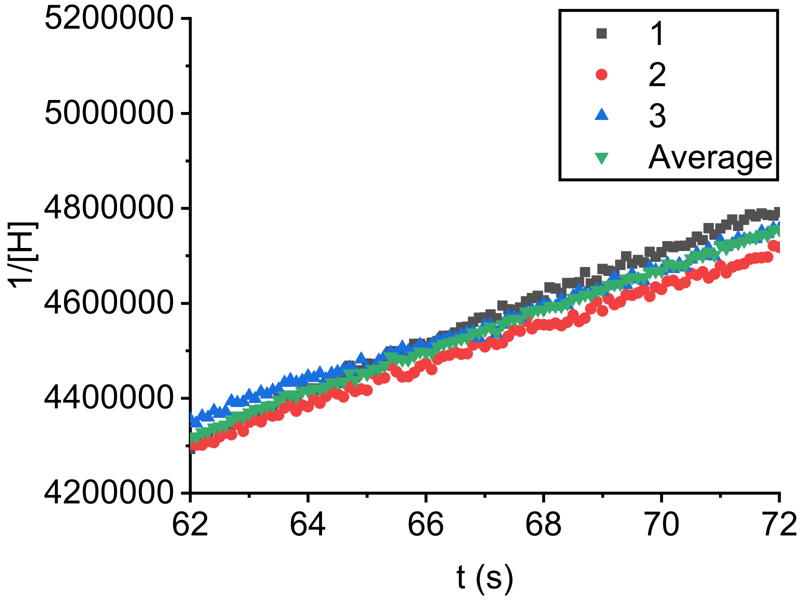

*Fig. S15. Second-order kinetics plots for the complexation of host (S,S,S,S)-**$H_2$3** to guest (R,R)-**15** (c = 2.5 × 10^{-7} M in $CHCl_3$/CH_3CN, 1:1, v/v, 298 K).*

*Table S17. Kinetic data for the threading of host (S,S,S,S)-**$H_2$3** onto guest (R,R)-**15**.*

**Entry**	**Host**	**Guest**	**k_{on} (× 10^4 M^{-1}·s^{-1})**	**R^2**
1	(*S,S,S,S*)-**$H_2$3**	(*R,R*)-**15**	4.92	0.996
2	(*S,S,S,S*)-**$H_2$3**	(*R,R*)-**15**	4.11	0.995
3	(*S,S,S,S*)-**$H_2$3**	(*R,R*)-**15**	3.98	0.995
**AVERAGE**	(*S,S,S,S*)-**$H_2$3**	(*R,R*)-**15**	4.33	0.999

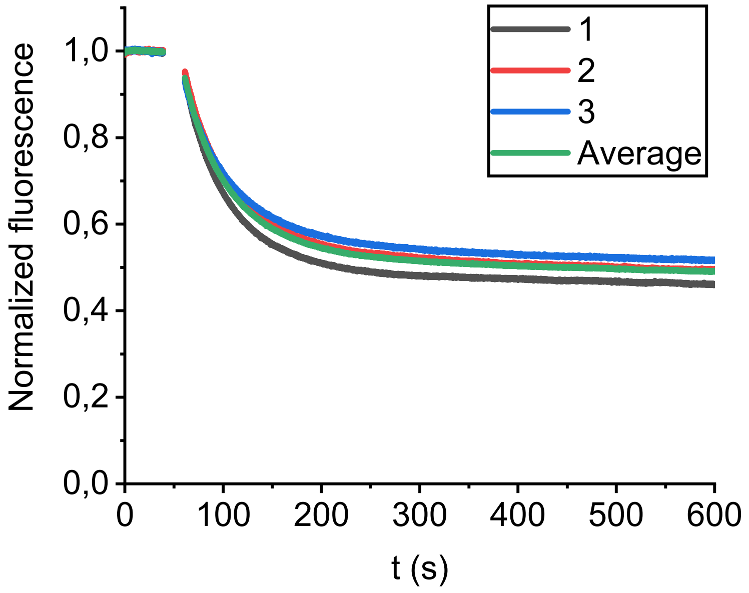

*Fig. S16. Normalized fluorescence intensity of host (R,R,R,R)-**H₂3** as a function of time after the addition (t = 50 s) of 1 equivalent of guest (S,S)-**15** (c = 2.5 × 10⁻⁷ M in CHCl₃/CH₃CN, 1:1, v/v, 298 K). The experiment was performed in triplicate.*

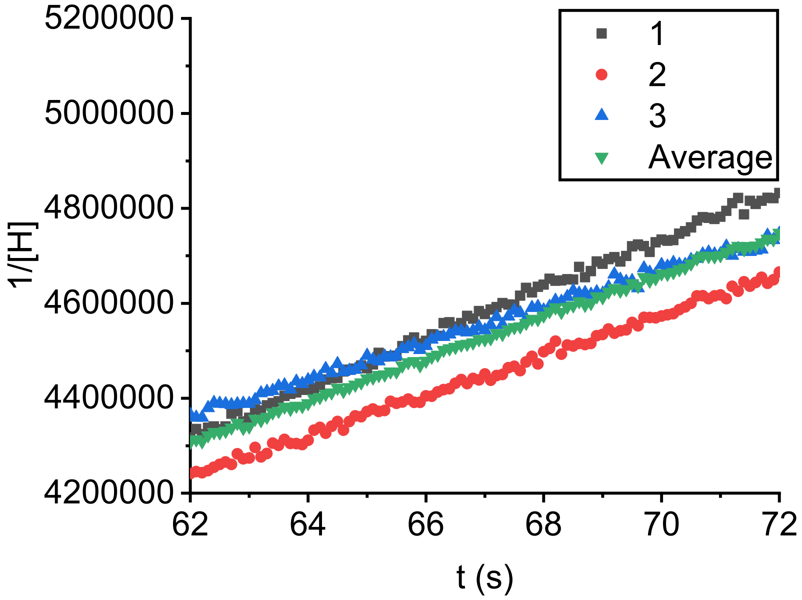

*Fig. S17. Second-order kinetics plots for the complexation of host (R,R,R,R)-**H₂3** to guest (S,S)-**15** (c = 2.5 × 10⁻⁷ M in CHCl₃/CH₃CN, 1:1, v/v, 298 K).*

*Table S18. Kinetic data for the threading of host (R,R,R,R)-**H₂3** onto guest (S,S)-**15**.*

**Entry**	**Host**	**Guest**	**k_{on} (× 10^4 $M^{-1}\cdot s^{-1}$)**	**R^2**
1	(*R*,*R*,*R*,*R*)-**$H_2$3**	(*S*,*S*)-**15**	5.21	0.997
2	(*R*,*R*,*R*,*R*)-**$H_2$3**	(*S*,*S*)-**15**	4.23	0.996
3	(*R*,*R*,*R*,*R*)-**$H_2$3**	(*S*,*S*)-**15**	3.82	0.995
**AVERAGE**	(*R*,*R*,*R*,*R*)-**$H_2$3**	(*S*,*S*)-**15**	4.42	0.999

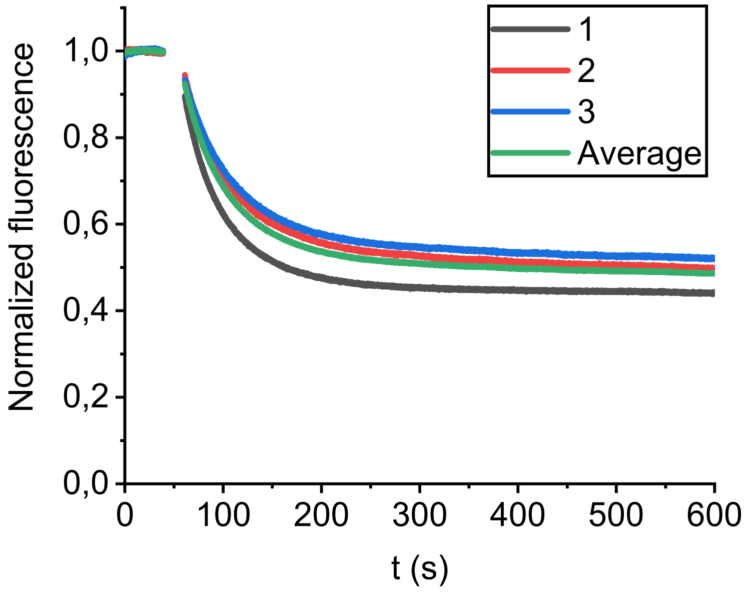

*Fig. S18. Normalized fluorescence intensity of host (S,S,S,S)-**H₂3** as a function of time after the addition (t = 50 s) of 1 equivalent of guest (S,S)-**15** (c = 2.5 × 10^{-7} M in $CHCl_3/CH_3CN$, 1:1, v/v, 298 K). The experiment was performed in triplicate.*

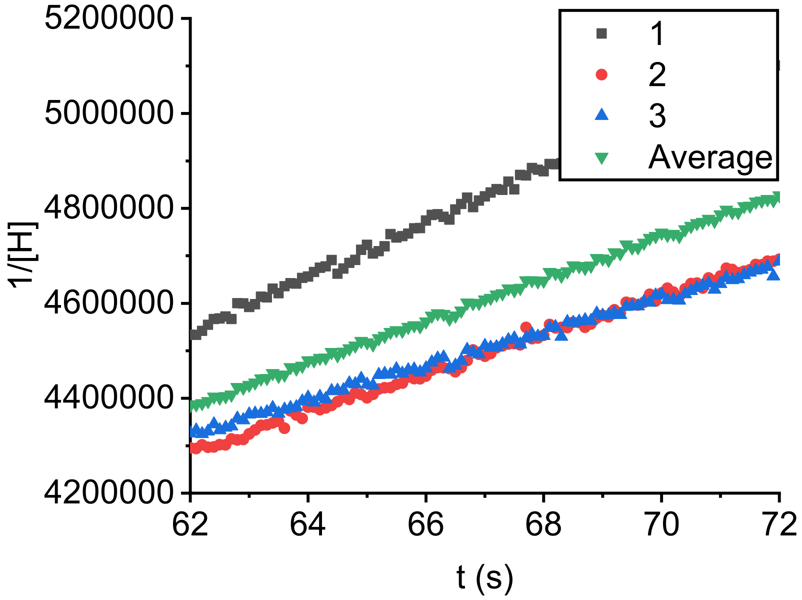

*Fig. S19. Second-order kinetics plots for the complexation of host (S,S,S,S)-$\mathbf{H_23}$ to guest (S,S)-**15** (c = 2.5 × 10^{-7} M in $CHCl_3/CH_3CN$, 1:1, v/v, 298 K).*

*Table S19. Kinetic data for the threading of host (S,S,S,S)-$\mathbf{H_23}$ onto guest (S,S)-**15**.*

**Entry**	**Host**	**Guest**	k_{on} **(× 10^4 $M^{-1}\cdot s^{-1}$)**	R^2
1	(*S,S,S,S*)-$\mathbf{H_23}$	(*S,S*)-**15**	5.70	0.997
2	(*S,S,S,S*)-$\mathbf{H_23}$	(*S,S*)-**15**	4.10	0.996
3	(*S,S,S,S*)-$\mathbf{H_23}$	(*S,S*)-**15**	3.55	0.995
**AVERAGE**	(*S,S,S,S*)-$\mathbf{H_23}$	(*S,S*)-**15**	4.45	0.999

Threading of host $H_2$1 or $H_2$3 onto guest 16.

A solution of host (2 mL, c = 3 × 10^{-6} M in $CHCl_3/CH_3CN$, 1:1, v/v, 298 K) was added to a 1 cm quartz cuvette and the cuvette was placed inside the fluorescence spectrometer. The fluorescence intensity of the host (λ_{ex} = 512 nm, λ_{em} = 647 nm) was monitored as a function of time (10 datapoints per second). At t ≈ 40 s, the cuvette was removed from the spectrometer. At t ≈ 50 s, a solution of **16** (3 µL, c = 2 × 10^{-3} M, 1 equiv) was added to the solution containing host. The contents of the cuvette were shaken vigorously for 5 seconds. At t ≈ 60 s, the cuvette was placed back inside the spectrometer. At t = 250 s (for **$H_2$1**) or at t = 600 s (for **$H_2$3**), the experiment was stopped. The fluorescence intensity of the host was normalized to the average fluorescence intensity during the initial 40 seconds. Each combination of host and guest was tested in triplicate. The kinetic plots for the threading of **$H_2$1** onto (*R*,*R*)-**16** are depicted in Fig. S20–S21 and the results are summarized in Table S20. The kinetic plots for the threading of (*R*,*R*,*R*,*R*)-**$H_2$3** onto (*R*,*R*)-**16** are depicted in Fig. S22–S23 and the results are summarized in Table S21. The kinetic plots for the threading of (*S*,*S*,*S*,*S*)-**$H_2$3** onto (*R*,*R*)-**16** are depicted in Fig. S24–S25 and the results are summarized in Table S22. The kinetic plots for the threading of **$H_2$1** onto (*S*,*S*)-**16** are depicted in Fig. S26–S27 and the results are summarized in Table S23. The kinetic plots for the threading of (*R*,*R*,*R*,*R*)-**$H_2$3** onto (*S*,*S*)-**16** are depicted in Fig. S28–S29 and the results are summarized in Table S24. The kinetic plots for the threading of (*S*,*S*,*S*,*S*)-**$H_2$3** onto (*S*,*S*)-**16** are depicted in Fig. S30–S31 and the results are summarized in Table S25.

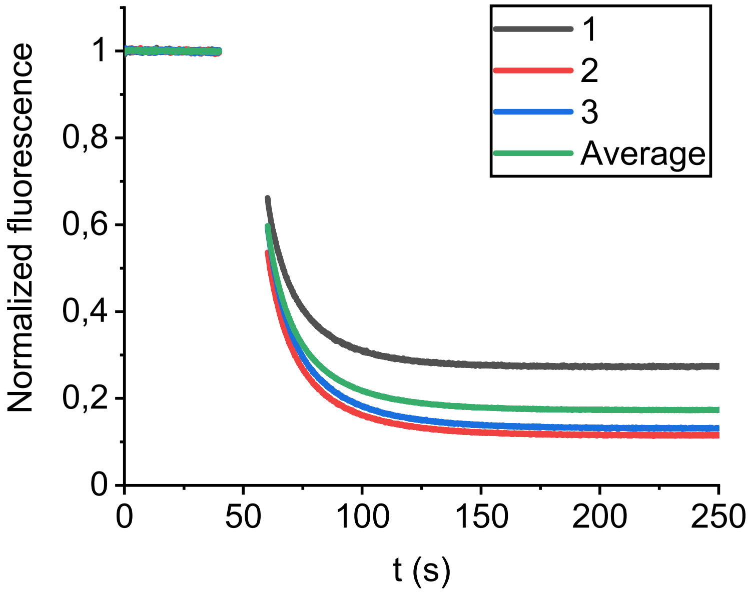

*Fig. S20. Normalized fluorescence intensity of host **H₂1** as a function of time after the addition (t = 50 s) of 1 equivalent of guest (R,R)-**16** (c = 3 × 10⁻⁶ M in CHCl₃/CH₃CN, 1:1, v/v, 298 K). The experiment was performed in triplicate.*

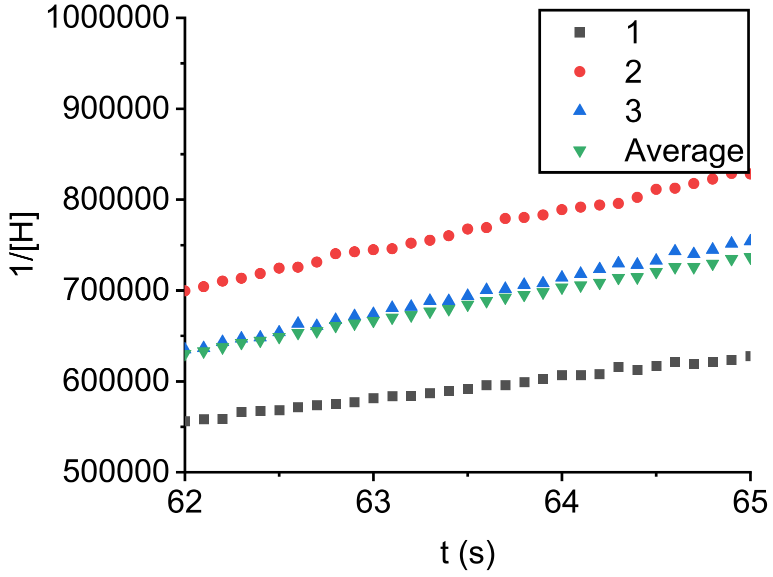

*Fig. S21. Second-order kinetics plots for the complexation of host **H₂1** to guest (R,R)-**16** (c = 3 × 10⁻⁶ M in CHCl₃/CH₃CN, 1:1, v/v, 298 K).*

*Table S20. Kinetic data for the threading of host **H₂1** onto guest (R,R)-**16**.*

**Entry**	**Host**	**Guest**	k_{on} (× 10^4 $M^{-1}\cdot s^{-1}$)	R^2
1	**$H_2$1**	(*R*,*R*)-**16**	2.24	0.994
2	**$H_2$1**	(*R*,*R*)-**16**	4.30	0.997
3	**$H_2$1**	(*R*,*R*)-**16**	3.99	0.997
**AVERAGE**	**$H_2$1**	(*R*,*R*)-**16**	3.56	0.999

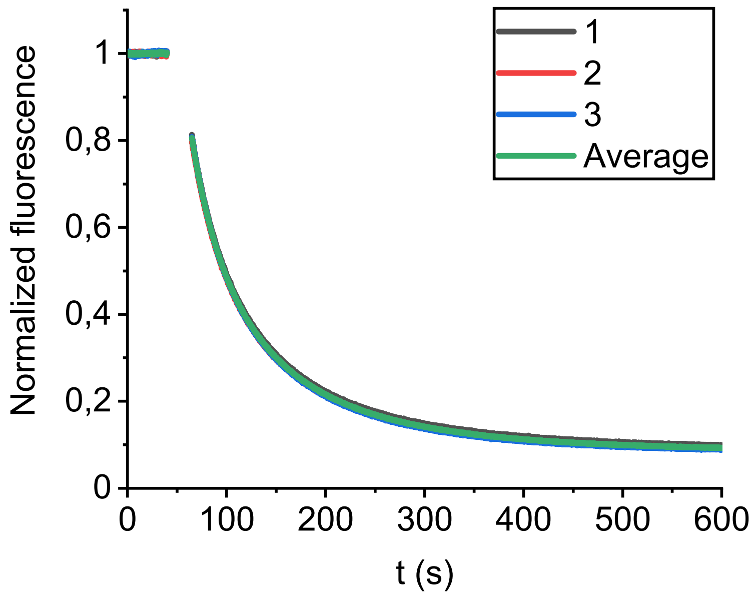

*Fig. S22. Normalized fluorescence intensity of host (R,R,R,R)-**$H_2$3** as a function of time after the addition (t = 50 s) of 1 equivalent of guest (R,R)-**16** (c = 3 × 10^{-6} M in $CHCl_3$/CH_3CN, 1:1, v/v, 298 K). The experiment was performed in triplicate.*

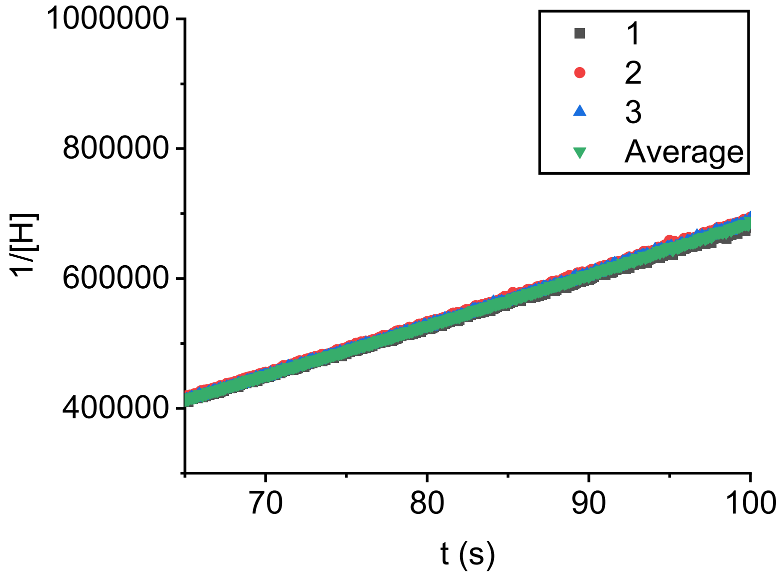

*Fig. S23. Second-order kinetics plots for the complexation of host (R,R,R,R)-**$H_2$3** to guest (R,R)-**16** (c = 3 × 10^{-6} M in $CHCl_3$/CH_3CN, 1:1, v/v, 298 K).*

*Table S21. Kinetic data for the threading of host (R,R,R,R)-**$H_2$3** onto guest (R,R)-**16**.*

**Entry**	**Host**	**Guest**	**k_{on} (× 10^3 M^{-1}·s^{-1})**	**R^2**
1	(*R*,*R*,*R*,*R*)-**$H_2$3**	(*R*,*R*)-**16**	7.65	0.999
2	(*R*,*R*,*R*,*R*)-**$H_2$3**	(*R*,*R*)-**16**	7.86	0.999
3	(*R*,*R*,*R*,*R*)-**$H_2$3**	(*R*,*R*)-**16**	7.87	0.999
**AVERAGE**	(*R*,*R*,*R*,*R*)-**$H_2$3**	(*R*,*R*)-**16**	7.79	0.999

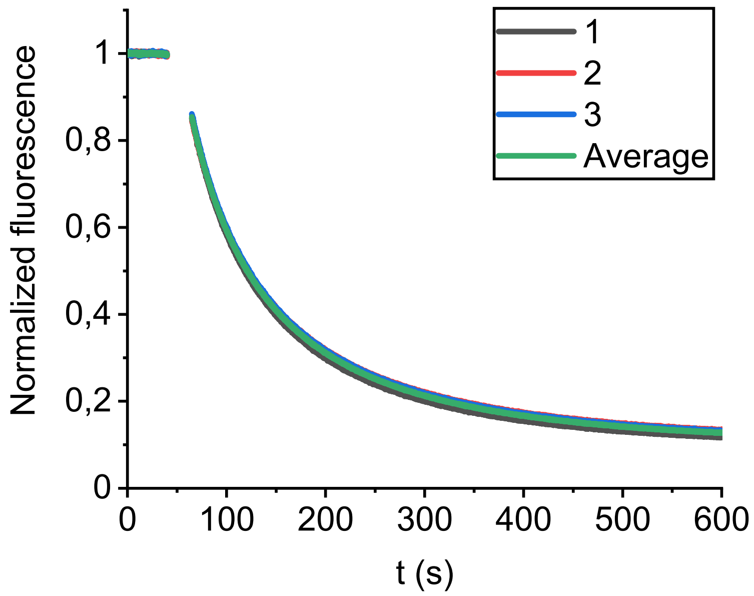

*Fig. S24. Normalized fluorescence intensity of host (S,S,S,S)-**$H_2$3** as a function of time after the addition (t = 50 s) of 1 equivalent of guest (R,R)-**16** (c = 3 × 10^{-6} M in $CHCl_3/CH_3CN$, 1:1, v/v, 298 K). The experiment was performed in triplicate.*

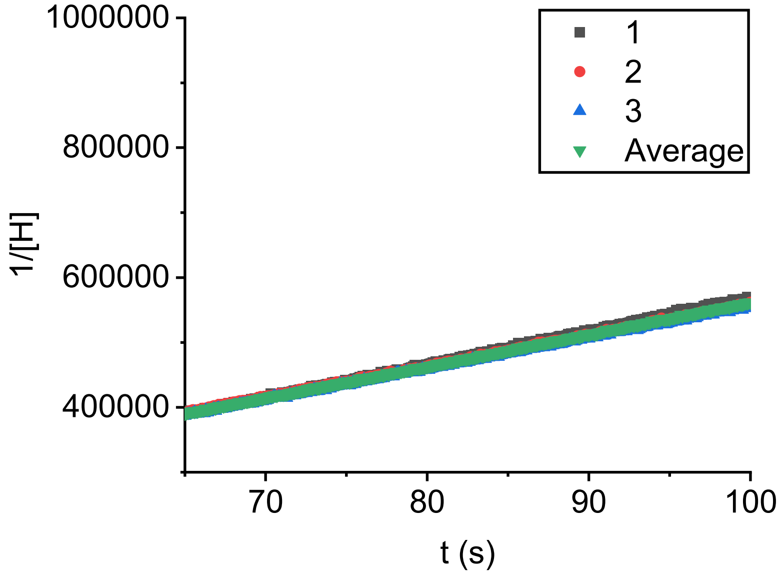

*Fig. S25. Second-order kinetics plots for the complexation of host (S,S,S,S)-**$H_2$3** to guest (R,R)-**16** (c = 3 × 10^{-6} M in $CHCl_3/CH_3CN$, 1:1, v/v, 298 K).*

*Table S22. Kinetic data for the threading of host (S,S,S,S)-**$H_2$3** onto guest (R,R)-**16**.*

**Entry**	**Host**	**Guest**	**k_{on} (× 10^3 $M^{-1}\cdot s^{-1}$)**	**R^2**
1	(*S*,*S*,*S*,*S*)-**$H_2$3**	(*R*,*R*)-**16**	5.09	0.999
2	(*S*,*S*,*S*,*S*)-**$H_2$3**	(*R*,*R*)-**16**	4.78	0.999
3	(*S*,*S*,*S*,*S*)-**$H_2$3**	(*R*,*R*)-**16**	4.80	0.999
**AVERAGE**	(*S*,*S*,*S*,*S*)-**$H_2$3**	(*R*,*R*)-**16**	4.89	0.999

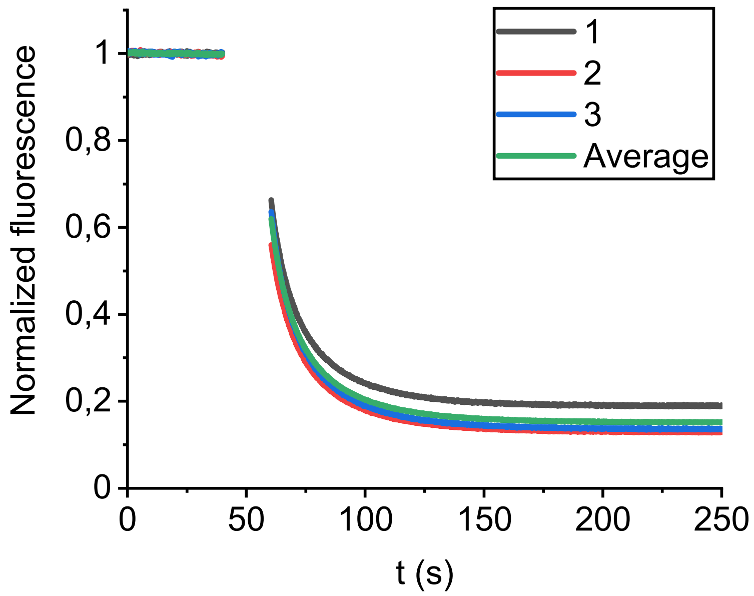

*Fig. S26. Normalized fluorescence intensity of host **$H_2$1** as a function of time after the addition (t = 50 s) of 1 equivalent of guest (S,S)-**16** (c = 3 × 10^{-6} M in $CHCl_3/CH_3CN$, 1:1, v/v, 298 K). The experiment was performed in triplicate.*

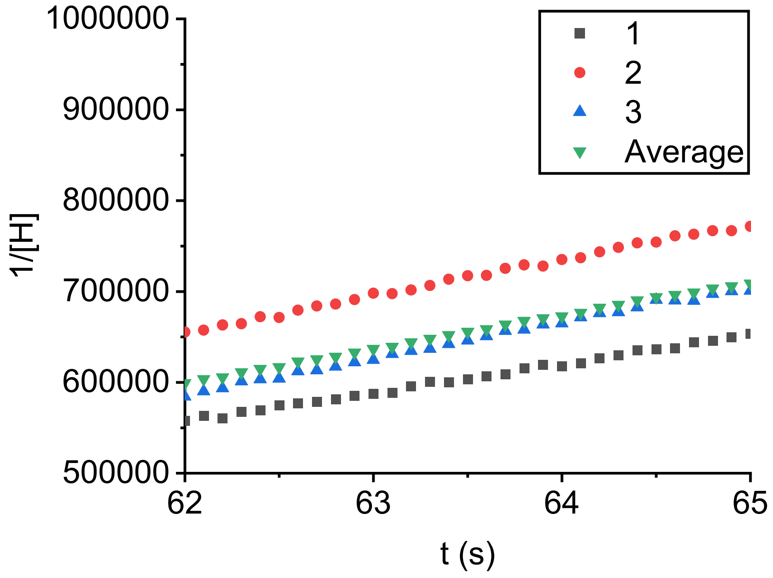

*Fig. S27. Second-order kinetics plots for the complexation of host **$H_2$1** to guest (S,S)-**16** (c = 3 × 10^{-6} M in $CHCl_3/CH_3CN$, 1:1, v/v, 298 K).*

*Table S23. Kinetic data for the threading of host **$H_2$1** onto guest (S,S)-**16**.*

**Entry**	**Host**	**Guest**	**k_{on} (× 10^4 M^{-1}·s^{-1})**	**R^2**
1	**$H_2$1**	(*S*,*S*)-**16**	3.17	0.997
2	**$H_2$1**	(*S*,*S*)-**16**	3.99	0.997
3	**$H_2$1**	(*S*,*S*)-**16**	3.97	0.997
**AVERAGE**	**$H_2$1**	(*S*,*S*)-**16**	3.71	0.999

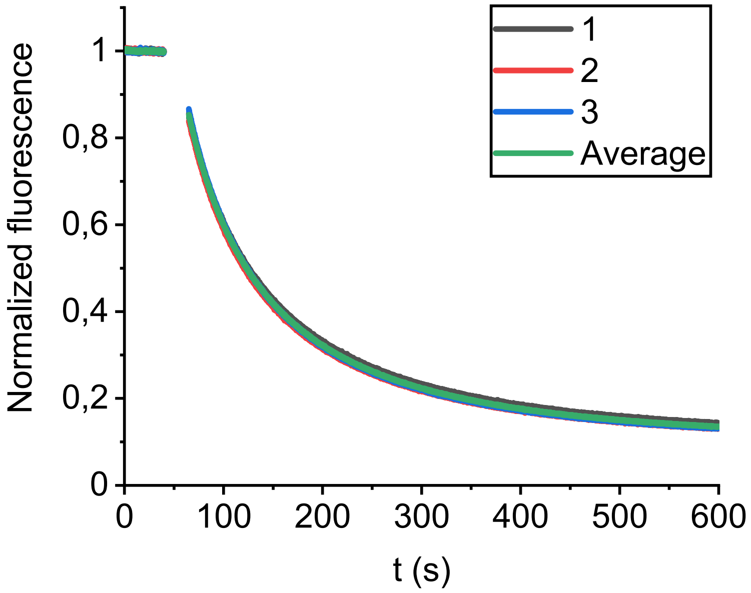

*Fig. S28. Normalized fluorescence intensity of host (R,R,R,R)-**H₂3** as a function of time after the addition (t = 50 s) of 1 equivalent of guest (S,S)-**16** (c = 3 × 10^{-6} M in $CHCl_3/CH_3CN$, 1:1, v/v, 298 K). The experiment was performed in triplicate.*

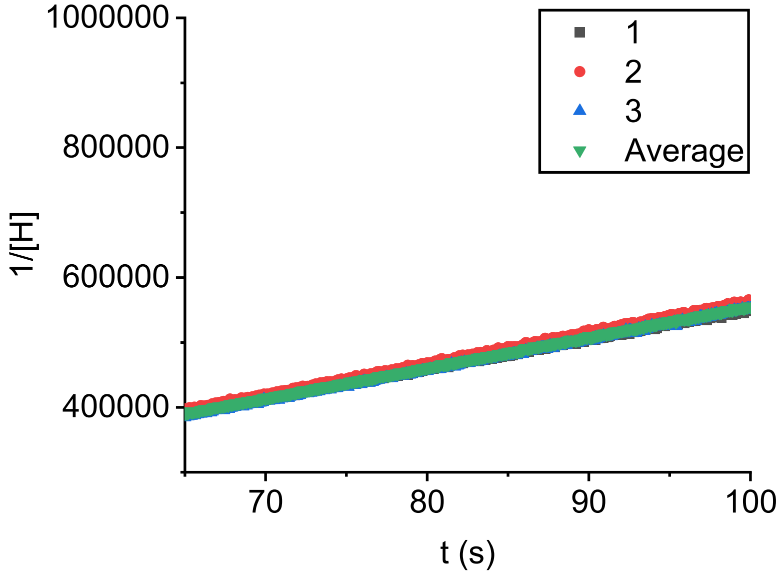

*Fig. S29. Second-order kinetics plots for the complexation of host (R,R,R,R)-**$H_2$3** to guest (S,S)-**16** (c = 3 × 10^{-6} M in $CHCl_3/CH_3CN$, 1:1, v/v, 298 K).*

*Table S24. Kinetic data for the threading of host (R,R,R,R)-**$H_2$3** onto guest (S,S)-**16**.*

**Entry**	**Host**	**Guest**	**k_{on} (× 10^3 M^{-1}·s^{-1})**	**R^2**
1	(*R*,*R*,*R*,*R*)-**$H_2$3**	(*S*,*S*)-**16**	4.56	0.999
2	(*R*,*R*,*R*,*R*)-**$H_2$3**	(*S*,*S*)-**16**	4.80	0.999
3	(*R*,*R*,*R*,*R*)-**$H_2$3**	(*S*,*S*)-**16**	4.76	0.999
**AVERAGE**	(*R*,*R*,*R*,*R*)-**$H_2$3**	(*S*,*S*)-**16**	4.71	0.999

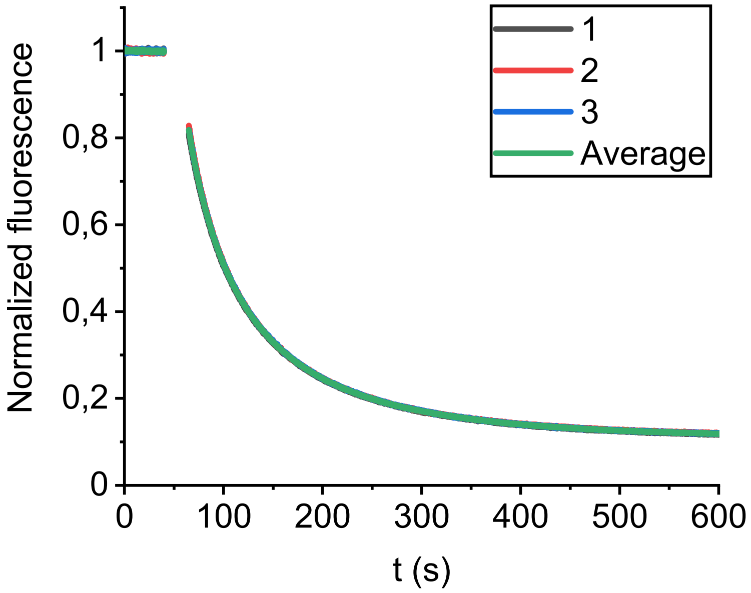

*Fig. S30. Normalized fluorescence intensity of host (S,S,S,S)-**$H_2$3** as a function of time after the addition (t = 50 s) of 1 equivalent of guest (S,S)-**16** (c = 3 × 10^{-6} M in $CHCl_3$/CH_3CN, 1:1, v/v, 298 K). The experiment was performed in triplicate.*

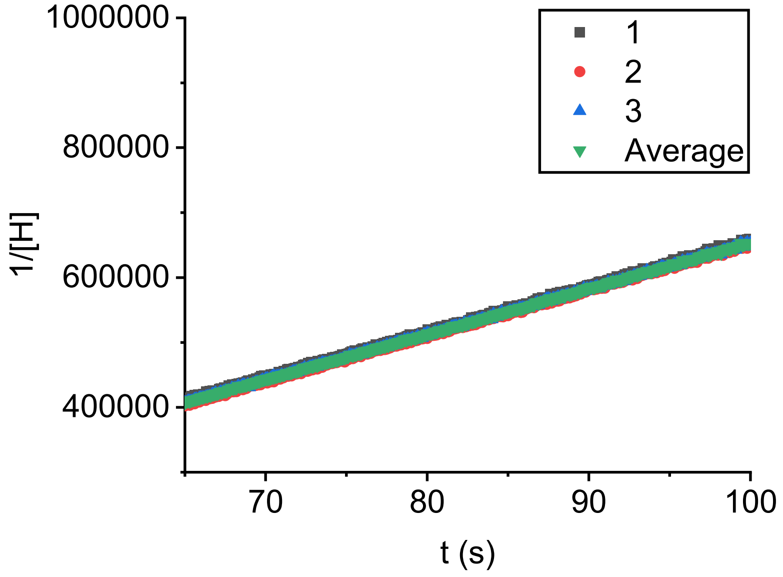

*Fig. S31. Second-order kinetics plots for the complexation of host (S,S,S,S)-**$H_2$3** to guest (S,S)-**16** (c = 3 × 10^{-6} M in $CHCl_3$/CH_3CN, 1:1, v/v, 298 K).*

*Table S25. Kinetic data for the threading of host (S,S,S,S)-**$H_2$3** onto guest (S,S)-**16**.*

**Entry**	**Host**	**Guest**	**k_{on} (× 10^3 M^{-1}·s^{-1})**	**R^2**
1	(*S*,*S*,*S*,*S*)-**$H_2$3**	(*S*,*S*)-**16**	7.00	0.999
2	(*S*,*S*,*S*,*S*)-**$H_2$3**	(*S*,*S*)-**16**	7.05	0.999
3	(*S*,*S*,*S*,*S*)-**$H_2$3**	(*S*,*S*)-**16**	7.01	0.999
**AVERAGE**	(*S*,*S*,*S*,*S*)-**$H_2$3**	(*S*,*S*)-**16**	7.02	0.999

Threading of host $H_2$1 or $H_2$3 onto guest 17.

A solution of host (2 mL, c = 10^{-5} M in $CHCl_3$/CH_3CN, 1:1, v/v, 298 K) was added to a 1 cm quartz cuvette and the cuvette was placed inside the fluorescence spectrometer. The fluorescence intensity of the host (λ_{ex} = 512 nm, λ_{em} = 647 nm) was monitored as a function of time (10 datapoints per second). At t ≈ 40 s, the cuvette was removed from the spectrometer. At t ≈ 50 s, a solution of **17** (10 µL, c = 2 × 10^{-3} M, 1 equiv) was added to the solution containing host. The contents of the cuvette were shaken vigorously for 5 seconds. At t ≈ 60 s, the cuvette was placed back inside the spectrometer. At t = 600 s, the experiment was stopped. The fluorescence intensity of the host was normalized to the average fluorescence intensity during the initial 40 seconds. Each combination of host and guest was tested in triplicate. The kinetic plots for the threading of **$H_2$1** onto (*R*,*R*)-**17** are depicted in Fig. S32–S33 and the results are summarized in Table S26. The kinetic plots for the threading of (*R*,*R*,*R*,*R*)-**$H_2$3** onto (*R*,*R*)-**17** are depicted in Fig. S34–S35 and the results are summarized in Table S27. The kinetic plots for the threading of (*S*,*S*,*S*,*S*)-**$H_2$3** onto (*R*,*R*)-**17** are depicted in Fig. S36–S37 and the results are summarized in Table S28. The kinetic plots for the threading of **$H_2$1** onto (*S*,*S*)-**17** are depicted in Fig. S38–S39 and the results are summarized in Table S29. The kinetic plots for the threading of (*R*,*R*,*R*,*R*)-**$H_2$3** onto (*S*,*S*)-**17** are depicted in Fig. S40–S41 and the results are summarized in Table S30. The kinetic plots for the threading of (*S*,*S*,*S*,*S*)-**$H_2$3** onto (*S*,*S*)-**17** are depicted in Fig. S42–S43 and the results are summarized in Table S31.

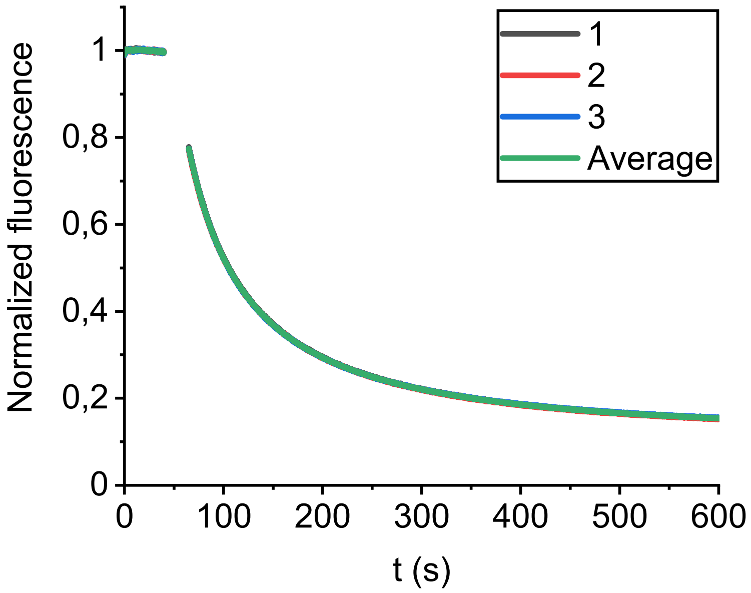

*Fig. S32. Normalized fluorescence intensity of host **H₂1** as a function of time after the addition (t = 50 s) of 1 equivalent of guest (R,R)-**17** (c = 10^{-5} M in $CHCl_3/CH_3CN$, 1:1, v/v, 298 K). The experiment was performed in triplicate.*

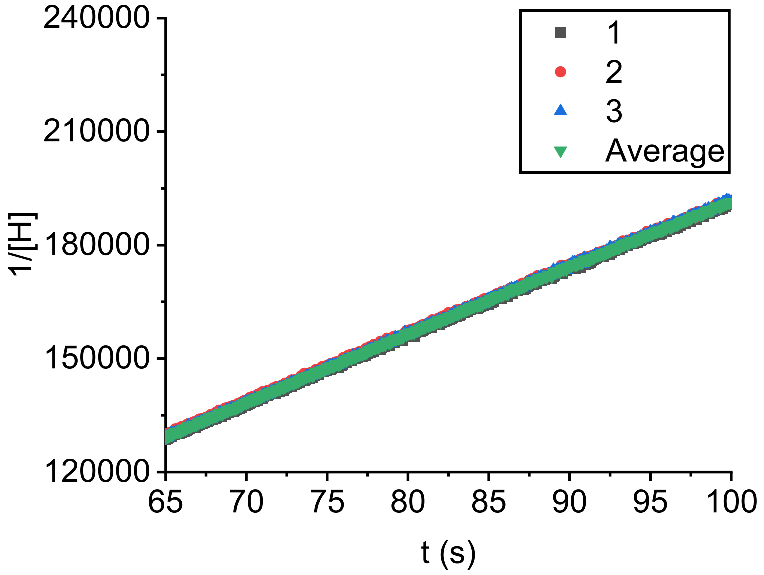

*Fig. S33. Second-order kinetics plots for the complexation of host **H₂1** to guest (R,R)-**17** (c = 10^{-5} M in $CHCl_3/CH_3CN$, 1:1, v/v, 298 K).*

*Table S26. Kinetic data for the threading of host **H₂1** onto guest (R,R)-**17**.*

**Entry**	**Host**	**Guest**	k_{on} **(× 10³ M⁻¹·s⁻¹)**	R^2
1	**H₂1**	(*R*,*R*)-**17**	1.78	0.999
2	**H₂1**	(*R*,*R*)-**17**	1.77	0.999
3	**H₂1**	(*R*,*R*)-**17**	1.79	0.999
**AVERAGE**	**H₂1**	(*R*,*R*)-**17**	1.78	0.999

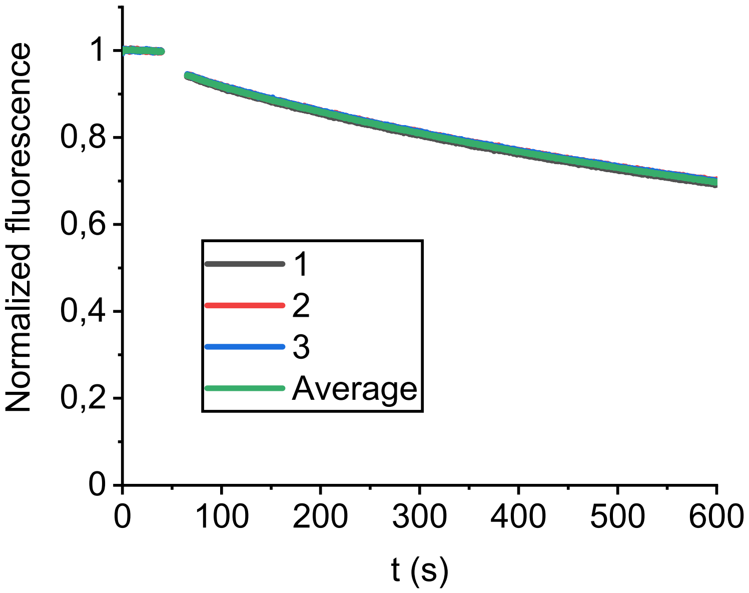

*Fig. S34. Normalized fluorescence intensity of host (R,R,R,R)-**$H_2$3** as a function of time after the addition (t = 50 s) of 1 equivalent of guest (R,R)-**17** (c = 10^{-5} M in $CHCl_3$/CH_3CN, 1:1, v/v, 298 K). The experiment was performed in triplicate.*

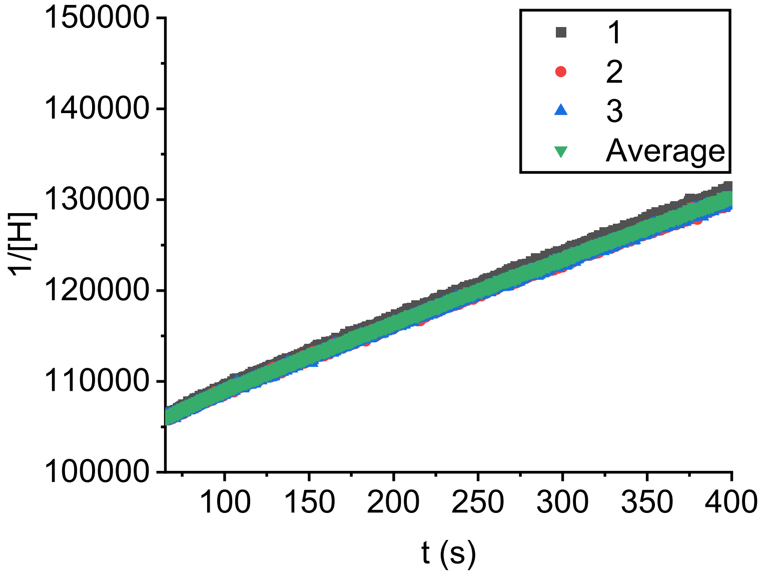

*Fig. S35. Second-order kinetics plots for the complexation of host (R,R,R,R)-**$H_2$3** to guest (R,R)-**17** (c = 10^{-5} M in $CHCl_3$/CH_3CN, 1:1, v/v, 298 K).*

*Table S27. Kinetic data for the threading of host (R,R,R,R)-**$H_2$3** onto guest (R,R)-**17**.*

**Entry**	**Host**	**Guest**	k_{on} (× 10^1 $M^{-1}\cdot s^{-1}$)	R^2
1	(*R*,*R*,*R*,*R*)-**$H_2$3**	(*R*,*R*)-**17**	7.33	0.999
2	(*R*,*R*,*R*,*R*)-**$H_2$3**	(*R*,*R*)-**17**	7.08	0.999
3	(*R*,*R*,*R*,*R*)-**$H_2$3**	(*R*,*R*)-**17**	7.09	0.999
**AVERAGE**	(*R*,*R*,*R*,*R*)-**$H_2$3**	(*R*,*R*)-**17**	7.17	0.999

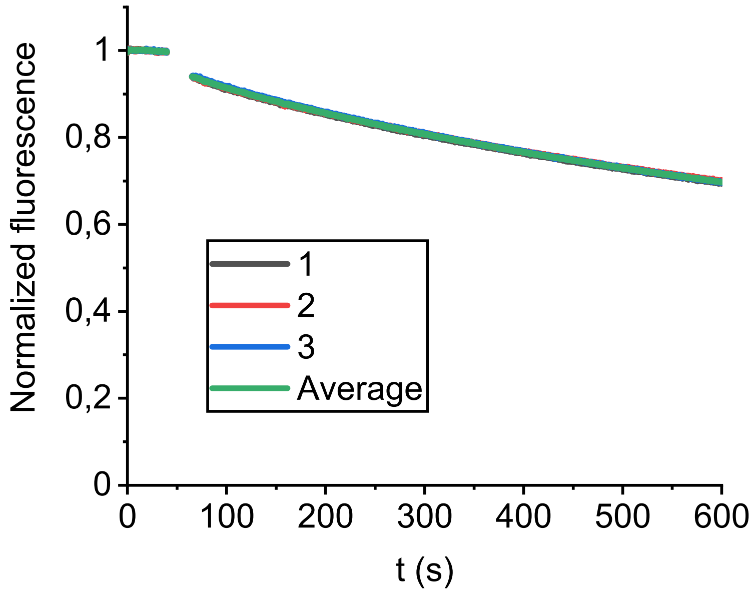

*Fig. S36. Normalized fluorescence intensity of host (S,S,S,S)-**$H_2$3** as a function of time after the addition (t = 50 s) of 1 equivalent of guest (R,R)-**17** (c = 10^{-5} M in $CHCl_3/CH_3CN$, 1:1, v/v, 298 K). The experiment was performed in triplicate.*

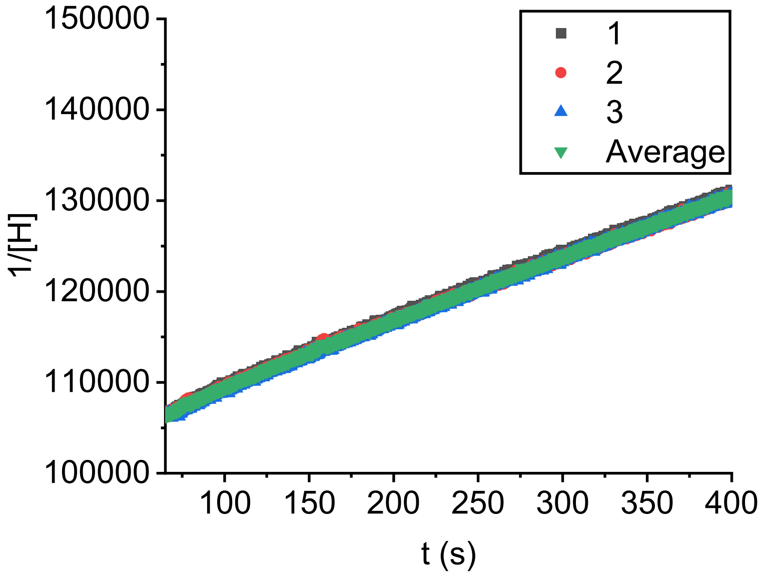

*Fig. S37. Second-order kinetics plots for the complexation of host (S,S,S,S)-**$H_2$3** to guest (R,R)-**17** (c = 10^{-5} M in $CHCl_3/CH_3CN$, 1:1, v/v, 298 K).*

*Table S28. Kinetic data for the threading of host (S,S,S,S)-**$H_2$3** onto guest (R,R)-**17**.*

**Entry**	**Host**	**Guest**	**k_{on} (× 10^1 M^{-1}·s^{-1})**	**R^2**
1	(*S*,*S*,*S*,*S*)-**$H_2$3**	(*R*,*R*)-**17**	7.15	0.998
2	(*S*,*S*,*S*,*S*)-**$H_2$3**	(*R*,*R*)-**17**	7.01	0.998
3	(*S*,*S*,*S*,*S*)-**$H_2$3**	(*R*,*R*)-**17**	7.15	0.999
**AVERAGE**	(*S*,*S*,*S*,*S*)-**$H_2$3**	(*R*,*R*)-**17**	7.10	0.999

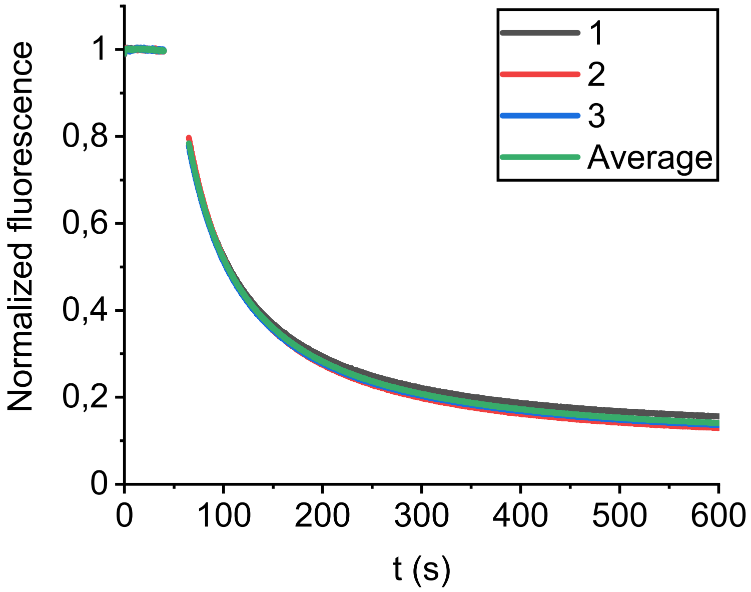

*Fig. S38. Normalized fluorescence intensity of host **$H_2$1** as a function of time after the addition (t = 50 s) of 1 equivalent of guest (S,S)-**17** (c = 10^{-5} M in $CHCl_3/CH_3CN$, 1:1, v/v, 298 K). The experiment was performed in triplicate.*

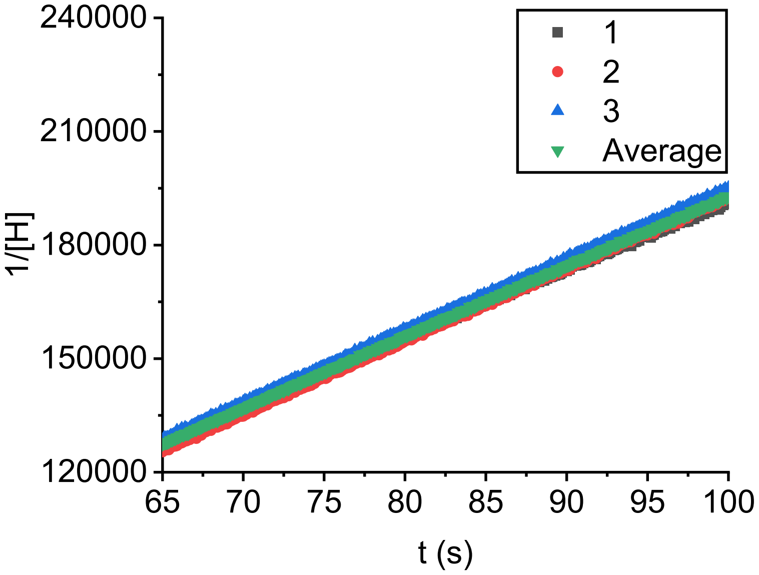

*Fig. S39. Second-order kinetics plots for the complexation of host **$H_2$1** to guest (S,S)-**17** (c = 10^{-5} M in $CHCl_3/CH_3CN$, 1:1, v/v, 298 K).*

*Table S29. Kinetic data for the threading of host **$H_2$1** onto guest (S,S)-**17**.*

**Entry**	**Host**	**Guest**	**k_{on} (× 10^3 $M^{-1}\cdot s^{-1}$)**	**R^2**
1	**$H_2$1**	(*S*,*S*)-**17**	1.81	0.999
2	**$H_2$1**	(*S*,*S*)-**17**	1.92	0.999
3	**$H_2$1**	(*S*,*S*)-**17**	1.89	0.999
**AVERAGE**	**$H_2$1**	(*S*,*S*)-**17**	1.87	0.999

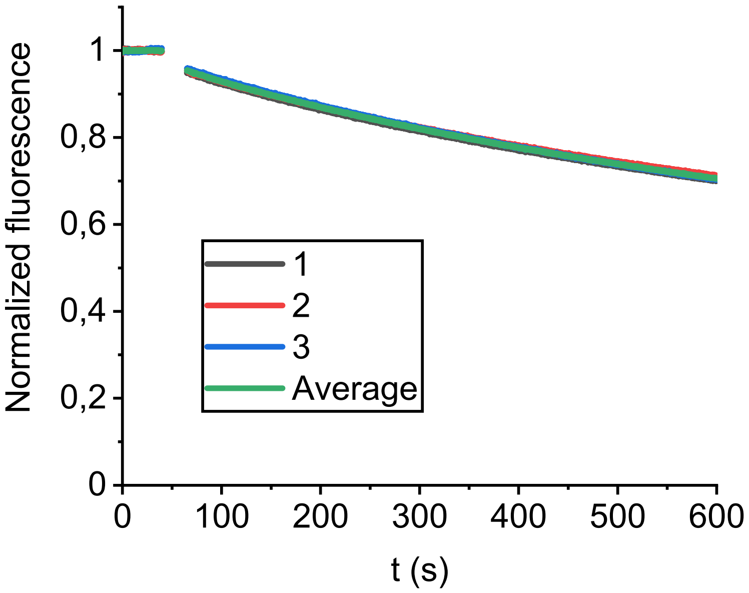

*Fig. S40. Normalized fluorescence intensity of host (R,R,R,R)-**$H_2$3** as a function of time after the addition (t = 50 s) of 1 equivalent of guest (S,S)-**17** (c = 10^{-5} M in $CHCl_3$/CH_3CN, 1:1, v/v, 298 K). The experiment was performed in triplicate.*

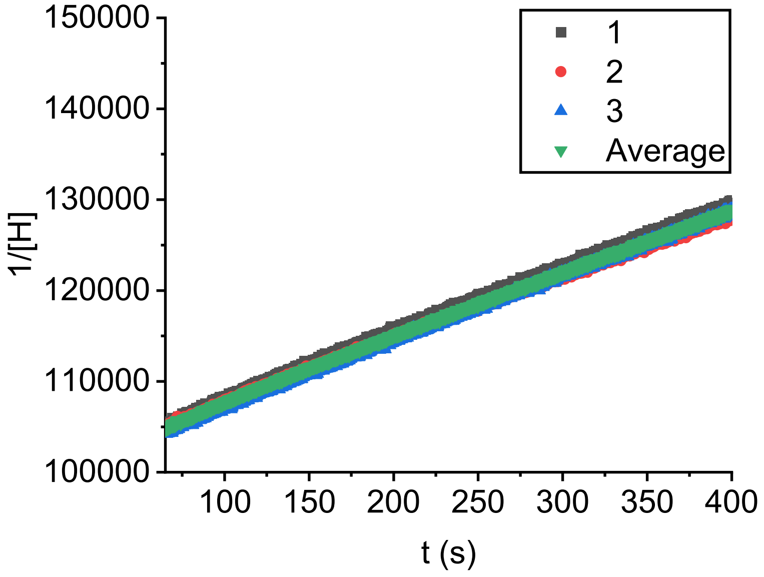

*Fig. S41. Second-order kinetics plots for the complexation of host (R,R,R,R)-**$H_2$3** to guest (S,S)-**17** (c = 10^{-5} M in $CHCl_3$/CH_3CN, 1:1, v/v, 298 K).*

*Table S30. Kinetic data for the threading of host (R,R,R,R)-**$H_2$3** onto guest (S,S)-**17**.*

**Entry**	**Host**	**Guest**	**k_{on} (× 10^1 M^{-1}·s^{-1})**	**R^2**
1	(*R*,*R*,*R*,*R*)-**$H_2$3**	(*S*,*S*)-**17**	7.16	0.999
2	(*R*,*R*,*R*,*R*)-**$H_2$3**	(*S*,*S*)-**17**	6.87	0.999
3	(*R*,*R*,*R*,*R*)-**$H_2$3**	(*S*,*S*)-**17**	7.21	0.999
**AVERAGE**	(*R*,*R*,*R*,*R*)-**$H_2$3**	(*S*,*S*)-**17**	7.08	0.999

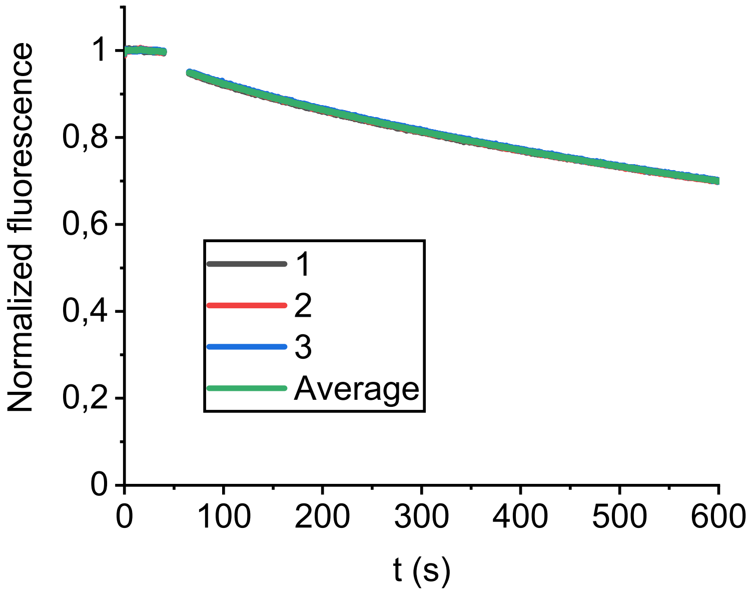

*Fig. S42. Normalized fluorescence intensity of host (S,S,S,S)-**H₂3** as a function of time after the addition (t = 50 s) of 1 equivalent of guest (S,S)-**17** (c = 10^{-5} M in $CHCl_3/CH_3CN$, 1:1, v/v, 298 K). The experiment was performed in triplicate.*

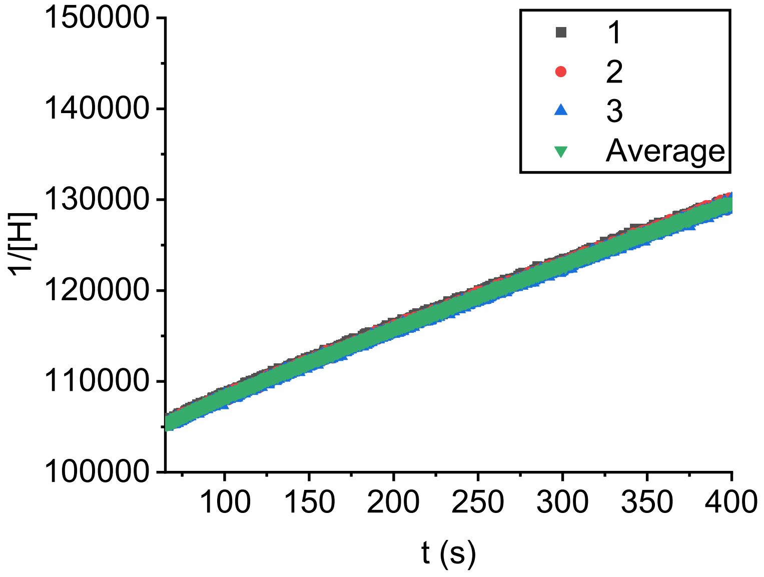

*Fig. S43. Second-order kinetics plots for the complexation of host (S,S,S,S)-**H₂3** to guest (S,S)-**17** (c = 10^{-5} M in $CHCl_3/CH_3CN$, 1:1, v/v, 298 K).*

*Table S31. Kinetic data for the threading of host (S,S,S,S)-**H₂3** onto guest (S,S)-**17**.*

**Entry**	**Host**	**Guest**	**k_{on} (× 10^1 M^{-1}·s^{-1})**	**R^2**
1	(*S,S,S,S*)-**$H_2$3**	(*S,S*)-**17**	7.20	0.999
2	(*S,S,S,S*)-**$H_2$3**	(*S,S*)-**17**	7.22	0.999
3	(*S,S,S,S*)-**$H_2$3**	(*S,S*)-**17**	7.18	0.999
**AVERAGE**	(*S,S,S,S*)-**$H_2$3**	(*S,S*)-**17**	7.20	0.999

Threading of host $H_2$1 or $H_2$3 onto guest 18.

A solution of host (2 mL, c = 10^{-5} M in $CHCl_3$/CH_3CN, 1:1, v/v, 298 K) was added to a 1 cm quartz cuvette and the cuvette was placed inside the fluorescence spectrometer. The fluorescence intensity of the host (λ_{ex} = 512 nm, λ_{em} = 647 nm) was monitored as a function of time (10 datapoints per second). At t ≈ 40 s, the cuvette was removed from the spectrometer. At t ≈ 50 s, a solution of **18** (10 µL, c = 2 × 10^{-3} M, 1 equiv) was added to the solution containing host. The contents of the cuvette were shaken vigorously for 5 seconds. At t ≈ 60 s, the cuvette was placed back inside the spectrometer. At t = 600 s, the experiment was stopped. The fluorescence intensity of the host was normalized to the average fluorescence intensity during the initial 40 seconds. Each combination of host and guest was tested in triplicate. The kinetic plots for the threading of **$H_2$1** onto (*R*,*R*)-**18** are depicted in Fig. S44–S45 and the results are summarized in Table S32. The kinetic plots for the threading of (*R*,*R*,*R*,*R*)-**$H_2$3** onto (*R*,*R*)-**18** are depicted in Fig. S46–S47 and the results are summarized in Table S33. The kinetic plots for the threading of (*S*,*S*,*S*,*S*)-**$H_2$3** onto (*R*,*R*)-**18** are depicted in Fig. S48–S49 and the results are summarized in Table S34. The kinetic plots for the threading of **$H_2$1** onto (*S*,*S*)-**18** are depicted in Fig. S50–S51 and the results are summarized in Table S35. The kinetic plots for the threading of (*R*,*R*,*R*,*R*)-**$H_2$3** onto (*S*,*S*)-**18** are depicted in Fig. S52–S53 and the results are summarized in Table S36. The kinetic plots for the threading of (*S*,*S*,*S*,*S*)-**$H_2$3** onto (*S*,*S*)-**18** are depicted in Fig. S54–S55 and the results are summarized in Table S37.

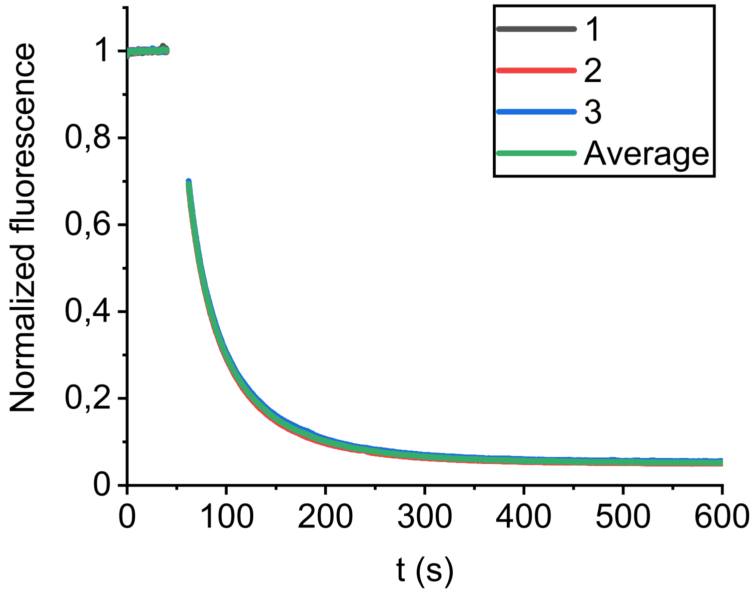

Fig. S44. Normalized fluorescence intensity of host ***$H_2$1*** *as a function of time after the addition (t = 50 s) of 1 equivalent of guest (R,R)-**18** (c = 10^{-5} M in $CHCl_3/CH_3CN$, 1:1, v/v, 298 K). The experiment was performed in triplicate.*

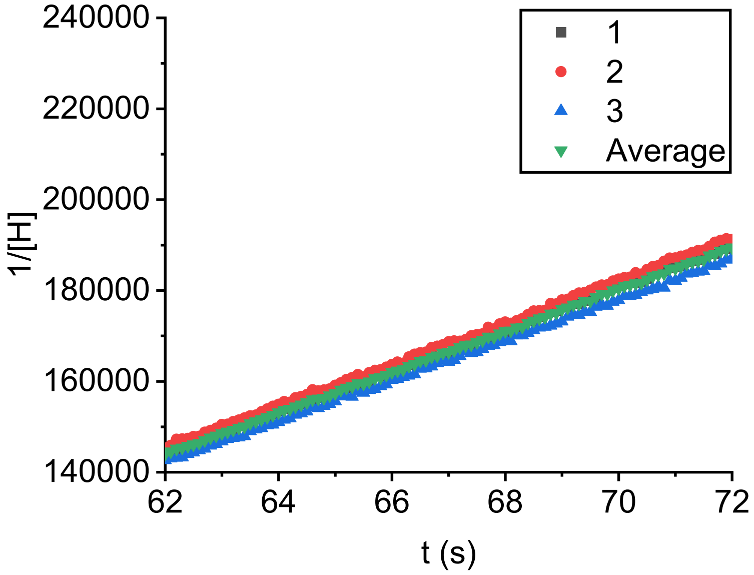

Fig. S45. Second-order kinetics plots for the complexation of host ***$H_2$1*** *to guest (R,R)-**18** (c = 10^{-5} M in $CHCl_3/CH_3CN$, 1:1, v/v, 298 K).*

Table S32. Kinetic data for the threading of host ***$H_2$1*** *onto guest (R,R)-**18**.*

**Entry**	**Host**	**Guest**	**k_{on} (× 10^3 M^{-1}·s^{-1})**	**R^2**
1	**$H_2$1**	(*R*,*R*)-**18**	4.57	0.999
2	**$H_2$1**	(*R*,*R*)-**18**	4.60	0.999
3	**$H_2$1**	(*R*,*R*)-**18**	4.46	0.999
**AVERAGE**	**$H_2$1**	(*R*,*R*)-**18**	4.54	0.999

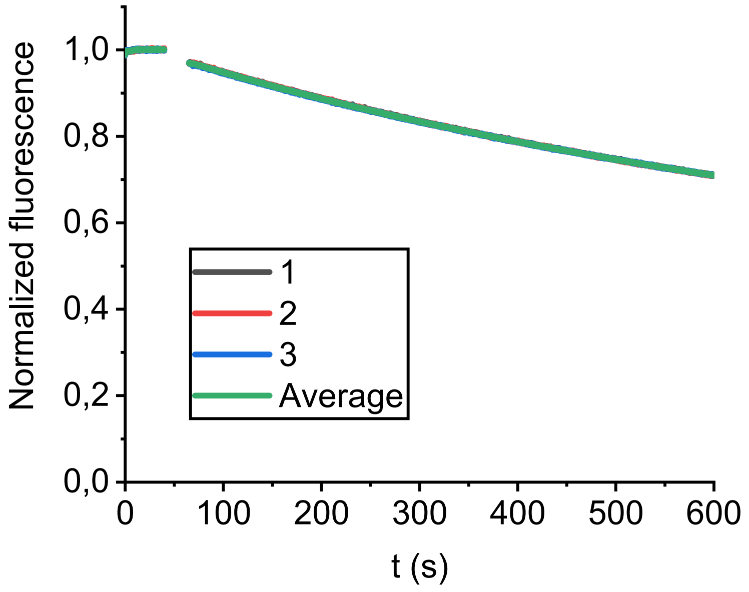

*Fig. S46. Normalized fluorescence intensity of host (R,R,R,R)-**$H_2$3** as a function of time after the addition (t = 50 s) of 1 equivalent of guest (R,R)-**18** (c = 10^{-5} M in $CHCl_3$/CH_3CN, 1:1, v/v, 298 K). The experiment was performed in triplicate.*

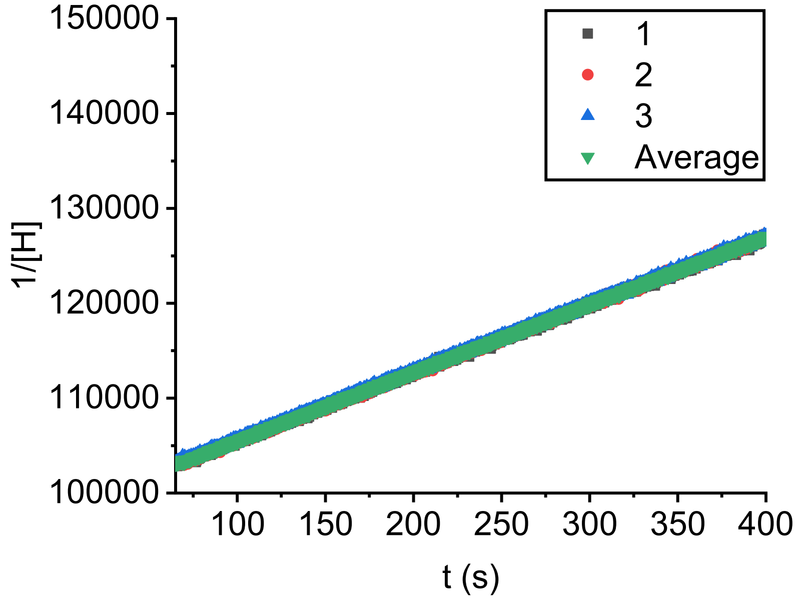

*Fig. S47. Second-order kinetics plots for the complexation of host (R,R,R,R)-**$H_2$3** to guest (R,R)-**18** (c = 10^{-5} M in $CHCl_3$/CH_3CN, 1:1, v/v, 298 K).*

*Table S33. Kinetic data for the threading of host (R,R,R,R)-**$H_2$3** onto guest (R,R)-**18**.*

**Entry**	**Host**	**Guest**	**k_{on} (× 10^1 M^{-1}·s^{-1})**	**R^2**
1	(*R*,*R*,*R*,*R*)-**$H_2$3**	(*R*,*R*)-**18**	7.15	0.999
2	(*R*,*R*,*R*,*R*)-**$H_2$3**	(*R*,*R*)-**18**	7.19	0.999
3	(*R*,*R*,*R*,*R*)-**$H_2$3**	(*R*,*R*)-**18**	7.14	0.999
**AVERAGE**	(*R*,*R*,*R*,*R*)-**$H_2$3**	(*R*,*R*)-**18**	7.16	0.999

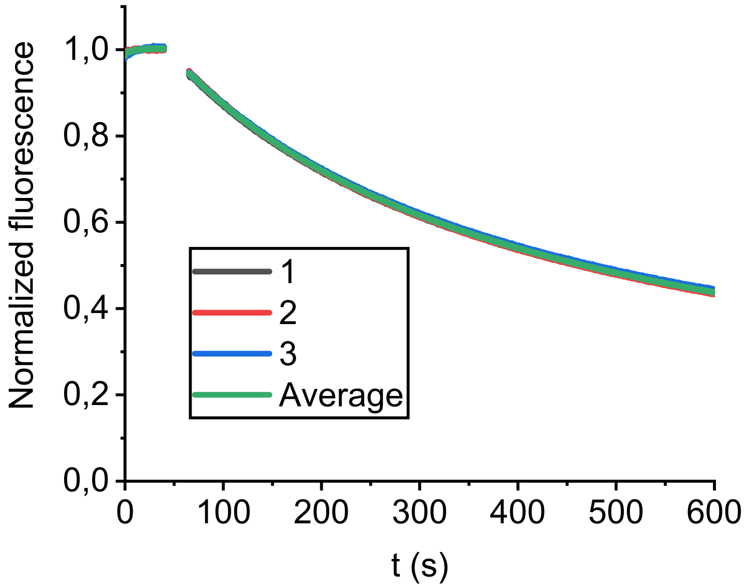

*Fig. S48. Normalized fluorescence intensity of host (S,S,S,S)-**$H_2$3** as a function of time after the addition (t = 50 s) of 1 equivalent of guest (R,R)-**18** (c = 10^{-5} M in $CHCl_3/CH_3CN$, 1:1, v/v, 298 K). The experiment was performed in triplicate.*

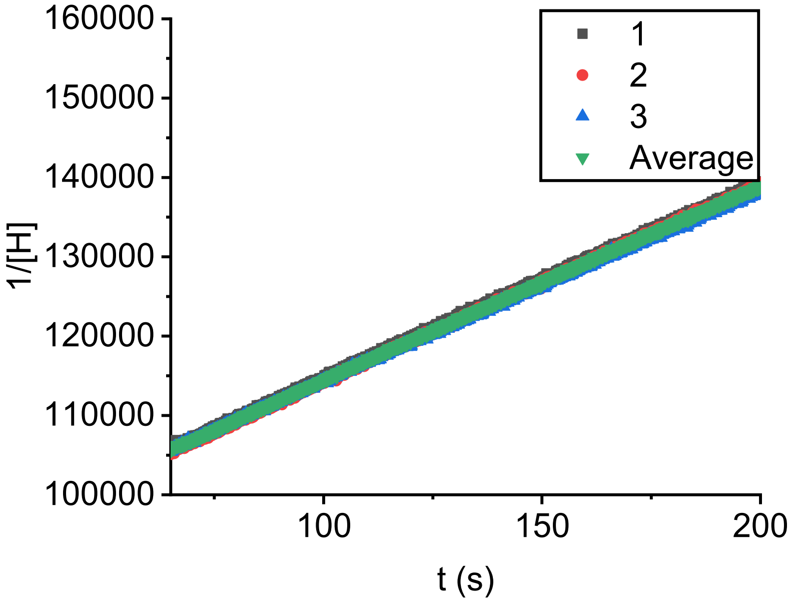

*Fig. S49. Second-order kinetics plots for the complexation of host (S,S,S,S)-**$H_2$3** to guest (R,R)-**18** (c = 10^{-5} M in $CHCl_3/CH_3CN$, 1:1, v/v, 298 K).*

*Table S34. Kinetic data for the threading of host (S,S,S,S)-**$H_2$3** onto guest (R,R)-**18**.*

**Entry**	**Host**	**Guest**	**k_{on} (× 10^2 M^{-1}·s^{-1})**	**R^2**
1	(*S*,*S*,*S*,*S*)-**$H_2$3**	(*R*,*R*)-**18**	2.47	0.999
2	(*S*,*S*,*S*,*S*)-**$H_2$3**	(*R*,*R*)-**18**	2.49	0.999
3	(*S*,*S*,*S*,*S*)-**$H_2$3**	(*R*,*R*)-**18**	2.42	0.999
**AVERAGE**	(*S*,*S*,*S*,*S*)-**$H_2$3**	(*R*,*R*)-**18**	2.46	0.999

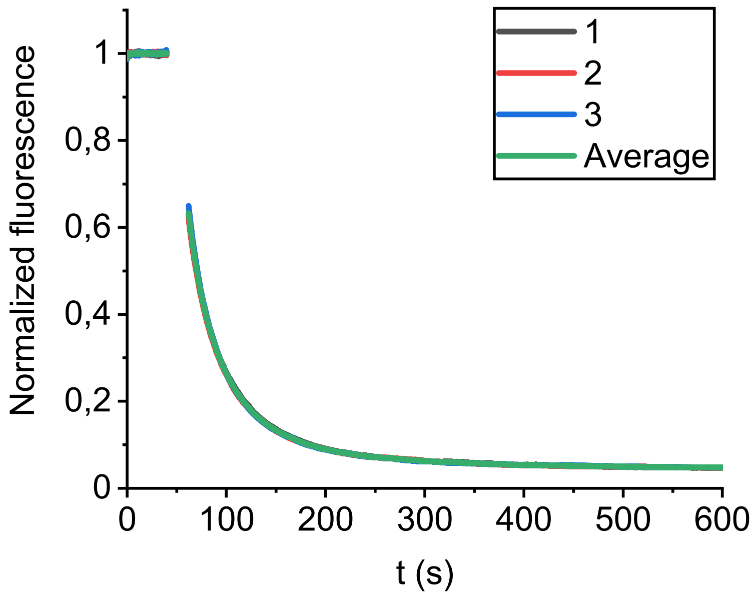

*Fig. S50. Normalized fluorescence intensity of host **$H_2$1** as a function of time after the addition (t = 50 s) of 1 equivalent of guest (S,S)-**18** (c = 10^{-5} M in $CHCl_3/CH_3CN$, 1:1, v/v, 298 K). The experiment was performed in triplicate.*

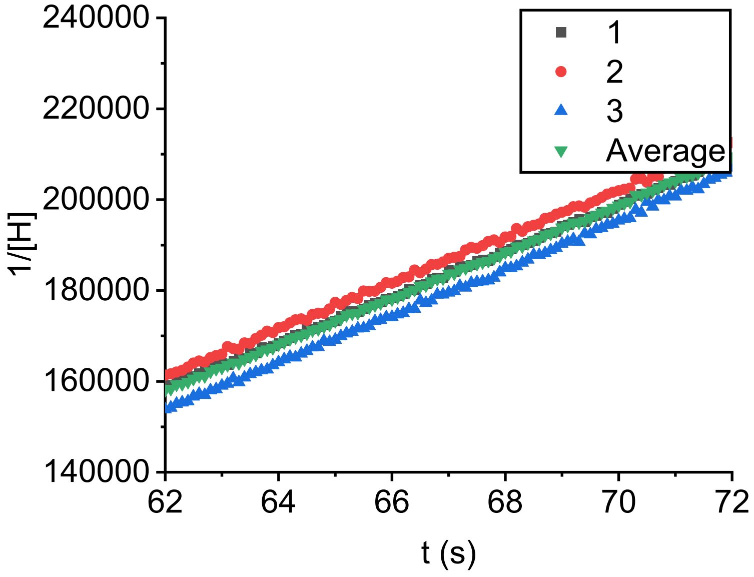

*Fig. S51. Second-order kinetics plots for the complexation of host **$H_2$1** to guest (S,S)-**18** (c = 10^{-5} M in $CHCl_3/CH_3CN$, 1:1, v/v, 298 K).*

*Table S35. Kinetic data for the threading of host **$H_2$1** onto guest (S,S)-**18**.*

**Entry**	**Host**	**Guest**	**k_{on} (× 10^3 $M^{-1}\cdot s^{-1}$)**	**R^2**
1	**$H_2$1**	(*S*,*S*)-**18**	5.03	0.999
2	**$H_2$1**	(*S*,*S*)-**18**	5.12	0.999
3	**$H_2$1**	(*S*,*S*)-**18**	5.24	0.999
**AVERAGE**	**$H_2$1**	(*S*,*S*)-**18**	5.13	0.999

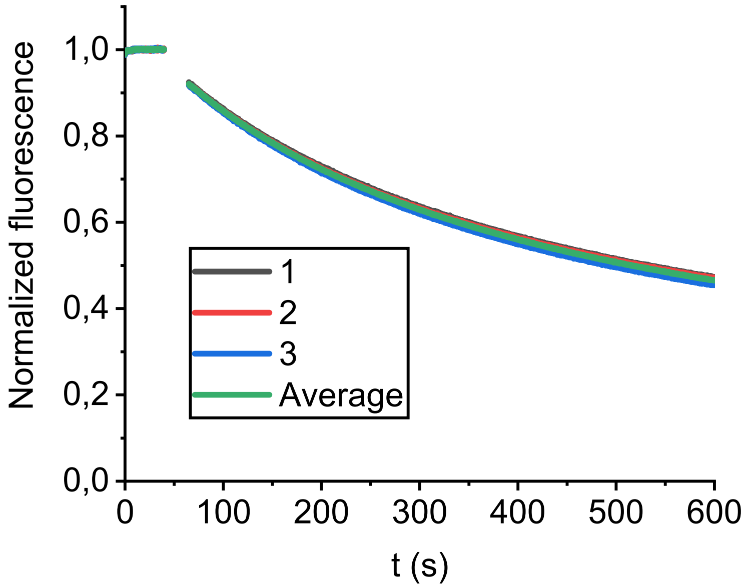

*Fig. S52. Normalized fluorescence intensity of host (R,R,R,R)-**$H_2$3** as a function of time after the addition (t = 50 s) of 1 equivalent of guest (S,S)-**18** (c = 10^{-5} M in $CHCl_3/CH_3CN$, 1:1, v/v, 298 K). The experiment was performed in triplicate.*

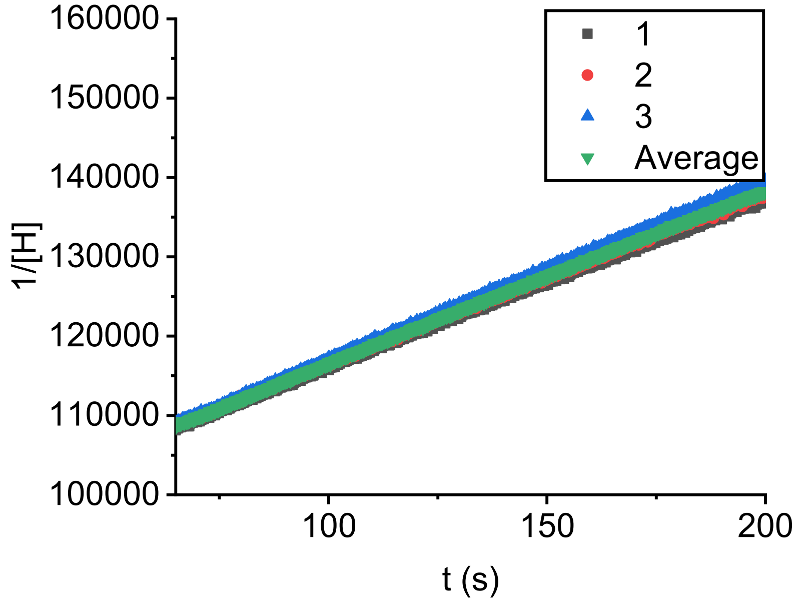

*Fig. S53. Second-order kinetics plots for the complexation of host (R,R,R,R)-**$H_2$3** to guest (S,S)-**18** (c = 10^{-5} M in $CHCl_3/CH_3CN$, 1:1, v/v, 298 K).*

*Table S36. Kinetic data for the threading of host (R,R,R,R)-**$H_2$3** onto guest (S,S)-**18**.*

**Entry**	**Host**	**Guest**	**k_{on} (× 10^2 $M^{-1}·s^{-1}$)**	**R^2**
1	(*R*,*R*,*R*,*R*)-**$H_2$3**	(*S*,*S*)-**18**	2.14	0.999
2	(*R*,*R*,*R*,*R*)-**$H_2$3**	(*S*,*S*)-**18**	2.17	0.999
3	(*R*,*R*,*R*,*R*)-**$H_2$3**	(*S*,*S*)-**18**	2.28	0.999
**AVERAGE**	(*R*,*R*,*R*,*R*)-**$H_2$3**	(*S*,*S*)-**18**	2.20	0.999

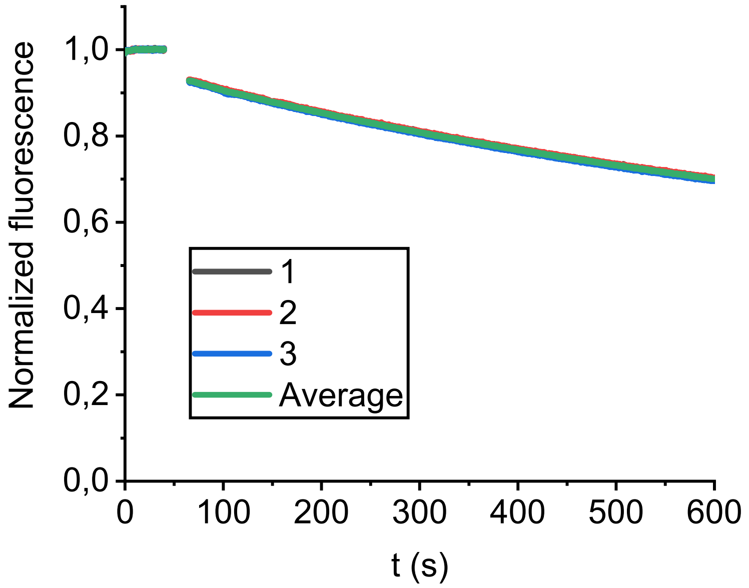

*Fig. S54. Normalized fluorescence intensity of host (S,S,S,S)-**$H_2$3** as a function of time after the addition (t = 50 s) of 1 equivalent of guest (S,S)-**18** (c = 10^{-5} M in $CHCl_3/CH_3CN$, 1:1, v/v, 298 K). The experiment was performed in triplicate.*

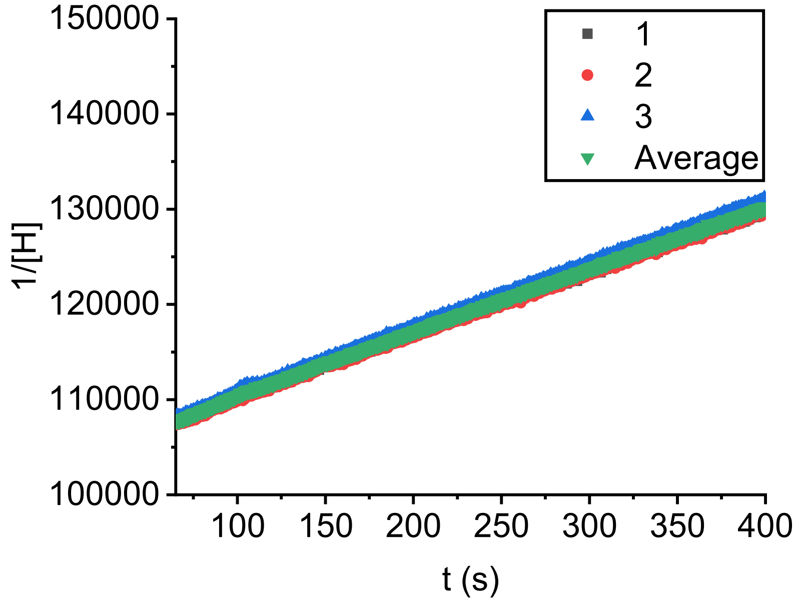

*Fig. S55. Second-order kinetics plots for the complexation of host (S,S,S,S)-**$H_2$3** to guest (S,S)-**18** (c = 10^{-5} M in $CHCl_3/CH_3CN$, 1:1, v/v, 298 K).*

*Table S37. Kinetic data for the threading of host (S,S,S,S)-**$H_2$3** onto guest (S,S)-**18**.*

**Entry**	**Host**	**Guest**	**k_{on} (× 10^1 M^{-1}·s^{-1})**	**R^2**
1	(*S,S,S,S*)-**$H_2$3**	(*S,S*)-**18**	6.57	0.999
2	(*S,S,S,S*)-**$H_2$3**	(*S,S*)-**18**	6.61	0.999
3	(*S,S,S,S*)-**$H_2$3**	(*S,S*)-**18**	6.77	0.999
**AVERAGE**	(*S,S,S,S*)-**$H_2$3**	(*S,S*)-**18**	6.65	0.999

2.2.5. Fluorescence dethreading studies

General information

As mentioned for complexation (threading), also decomplexation (dethreading) is reversible. Nonetheless, the initial stages of the dethreading process can be described by first-order kinetics according to equations 7–9.

$$\frac{\partial[H]}{\partial t} = k_{off}[HG] \qquad (7)$$

If we assure that the total concentrations of host and guest are equal, *i.e.* $[H]_T = [G]_T$, and $[HG] = [H]_T - [H]$, then

$$\frac{\partial[H]}{\partial t} = k_{off}([H]_T - [H]) \qquad (8)$$

The solution to this differential equation is

$$\ln([H]_T - [H]) = \ln([H]_T - [H]_0) - k_{off}t \qquad (9)$$

By plotting the natural logarithm of the total concentration of host minus the actual concentration of host as a function of time, a linear fit is obtained in which the rate constant k_{off} equals the negative slope.

Dethreading of 14 from $H_2$3.

Solvent mixture (3 mL, $CHCl_3/CH_3CN$, 1:1, v/v, 298 K) was added to a 1 cm quartz cuvette and the cuvette was placed inside the fluorescence spectrometer. The fluorescence intensity (λ_{ex} = 416 nm, λ_{em} = 647 nm) was monitored as a function of time (10 datapoints per second). At t ≈ 40 s, the cuvette was removed from the spectrometer. At t ≈ 50 s, a solution of **$H_2$3·14** (3 µL, c = 10^{-3} M, 1:1, host:guest) in the same solvent mixture was added to the cuvette. The contents of the cuvette were shaken vigorously for 5 seconds. At t ≈ 60 s, the cuvette was placed back inside the spectrometer. At t = 600 s, the experiment was stopped. The fluorescence intensity of the host was normalized to the average fluorescence intensity of host in a separate experiment in which **$H_2$3** (3 µL, c = 10^{-3} M) was added to the solvent mixture (3 mL, $CHCl_3/CH_3CN$, 1:1, v/v, 298 K). Each combination of host and guest was tested in triplicate. The kinetic plots for the dethreading of **14** from (*R*,*R*,*R*,*R*)-**$H_2$3** are depicted in Fig. S56–S57 and the results are summarized in Table S38. The kinetic plots for the threading of **14** from (*S*,*S*,*S*,*S*)-**$H_2$3** are depicted in Fig. S58–S59 and the results are summarized in Table S39.

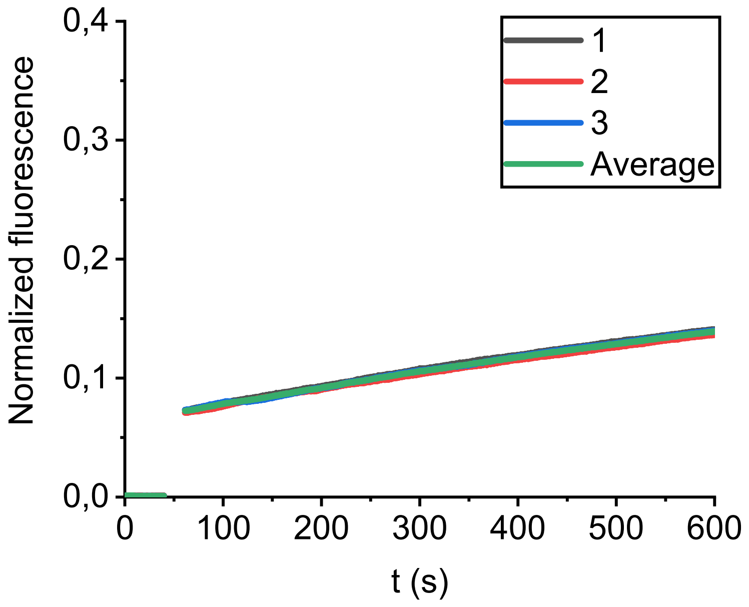

*Fig. S56. Normalized fluorescence intensity of host (R,R,R,R)-**$H_2$3** as a function of time after dilution induced decomplexation of (R,R,R,R)-**$H_2$3·14** (addition at t = 50 s, c = 10^{-6} M in $CHCl_3/CH_3CN$, 1:1, v/v, 298 K). The experiment was performed in triplicate.*

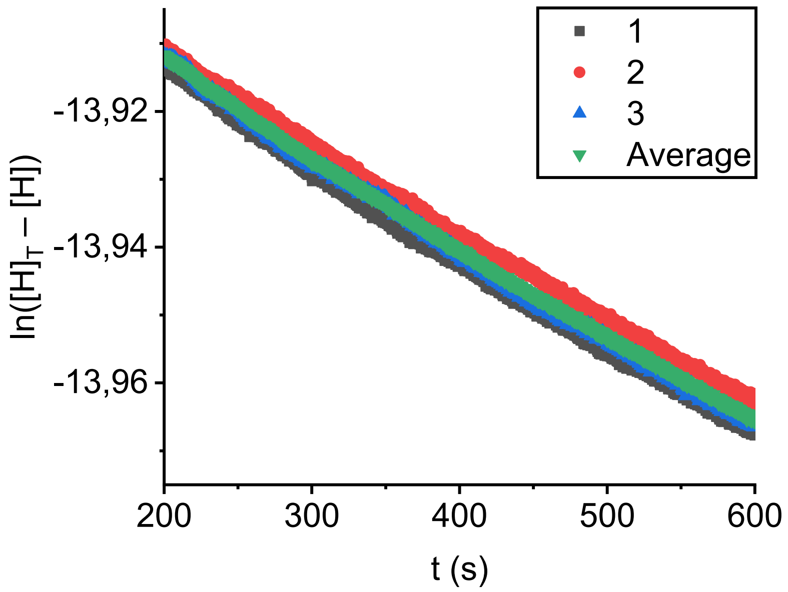

*Fig. S57. First-order kinetics plots for the decomplexation of host-guest complex (R,R,R,R)-**$H_2$3·14** (c = 10^{-6} M in $CHCl_3/CH_3CN$, 1:1, v/v, 298 K).*

*Table S38. Kinetic data for the dethreading of guest **14** from host (R,R,R,R)-**$H_2$3**.*

**Entry**	**Host**	**Guest**	**k_{off} (× 10^{-4} s^{-1})**	**R^2**
1	(*R*,*R*,*R*,*R*)-**$H_2$3**	**14**	1.34	0.997
2	(*R*,*R*,*R*,*R*)-**$H_2$3**	**14**	1.30	0.999
3	(*R*,*R*,*R*,*R*)-**$H_2$3**	**14**	1.34	0.998
**AVERAGE**	(*R*,*R*,*R*,*R*)-**$H_2$3**	**14**	1.33	0.998

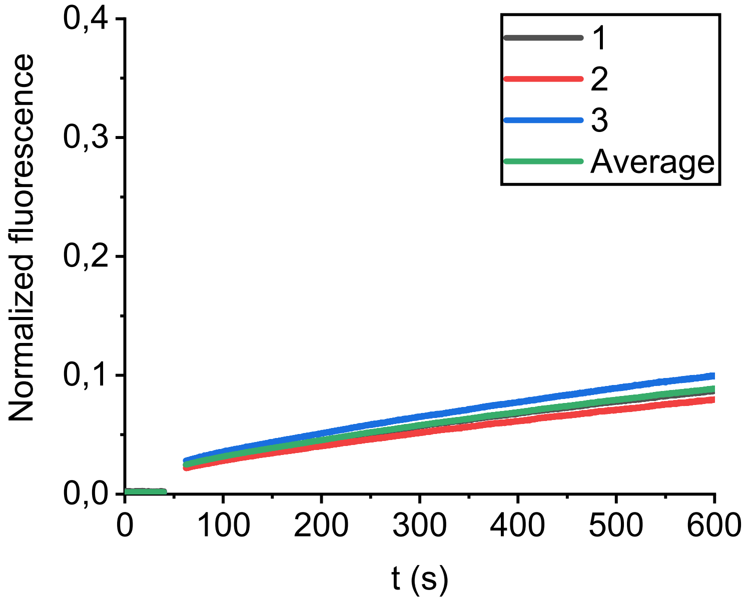

*Fig. S58. Normalized fluorescence intensity of host (S,S,S,S)-**$H_2$3** as a function of time after dilution induced decomplexation of (S,S,S,S)-**$H_2$3·14** (addition at t = 50 s, c = 10^{-6} M in $CHCl_3$/CH_3CN, 1:1, v/v, 298 K). The experiment was performed in triplicate.*

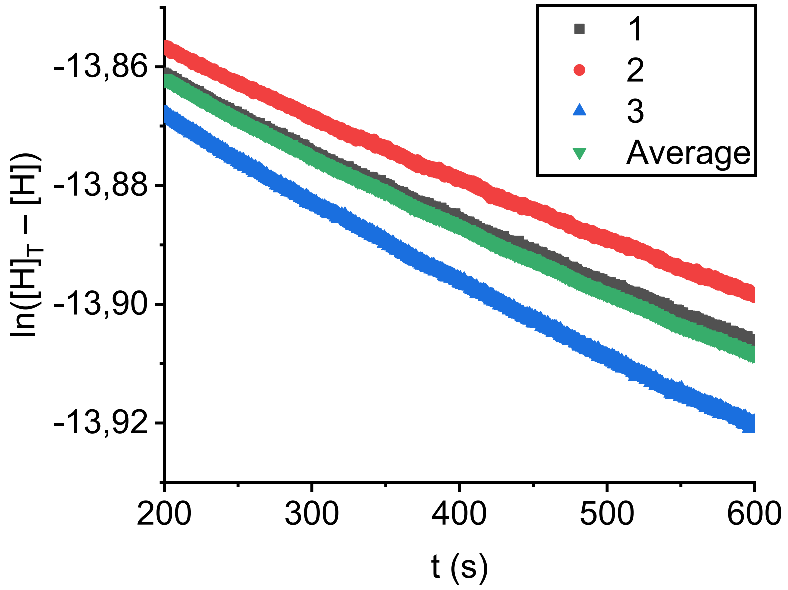

*Fig. S59. First-order kinetics plots for the decomplexation of host-guest complex (S,S,S,S)-**$H_2$3·14** (c = 10^{-6} M in $CHCl_3$/CH_3CN, 1:1, v/v, 298 K).*

*Table S39. Kinetic data for the dethreading of guest **14** from host (S,S,S,S)-**$H_2$3**.*

**Entry**	**Host**	**Guest**	**k_{off} (× 10^{-4} s^{-1})**	**R^2**
1	(*S,S,S,S*)-**$H_2$3**	**14**	1.12	0.998
2	(*S,S,S,S*)-**$H_2$3**	**14**	1.04	0.998
3	(*S,S,S,S*)-**$H_2$3**	**14**	1.31	0.998
**AVERAGE**	(*S,S,S,S*)-**$H_2$3**	**14**	1.15	0.998

Dethreading of 15 from $H_2$3.

Solvent mixture (2 mL, $CHCl_3/CH_3CN$, 1:1, v/v, 298 K) was added to a 1 cm quartz cuvette and the cuvette was placed inside the fluorescence spectrometer. The fluorescence intensity (λ_{ex} = 416 nm, λ_{em} = 647 nm) was monitored as a function of time (10 datapoints per second). At t ≈ 40 s, the cuvette was removed from the spectrometer. At t ≈ 50 s, a solution of **$H_2$3·15** (10 µL, c = 5 × 10^{-5} M, 1:1, host:guest) in the same solvent mixture was added to the cuvette. The contents of the cuvette were shaken vigorously for 5 seconds. At t ≈ 60 s, the cuvette was placed back inside the spectrometer. At t = 600 s, the experiment was stopped. The fluorescence intensity of the host was normalized to the average fluorescence intensity of host in a separate experiment in which **$H_2$3** (10 µL, c = 5 × 10^{-5} M) was added to the solvent mixture (2 mL, $CHCl_3/CH_3CN$, 1:1, v/v, 298 K). Each combination of host and guest was tested in triplicate. The kinetic plots for the dethreading of (*R*,*R*)-**15** from (*R*,*R*,*R*,*R*)-**$H_2$3** are depicted in Fig. S60–S61 and the results are summarized in Table S40. The kinetic plots for the dethreading of (*R*,*R*)-**15** from (*S*,*S*,*S*,*S*)-**$H_2$3** are depicted in Fig. S62–S63 and the results are summarized in Table S41. The kinetic plots for the dethreading of (*S*,*S*)-**15** from (*R*,*R*,*R*,*R*)-**$H_2$3** are depicted in Fig. S64–S65 and the results are summarized in Table S42. The kinetic plots for the dethreading of (*S*,*S*)-**15** from (*S*,*S*,*S*,*S*)-**$H_2$3** are depicted in Fig. S66–S67 and the results are summarized in Table S43.

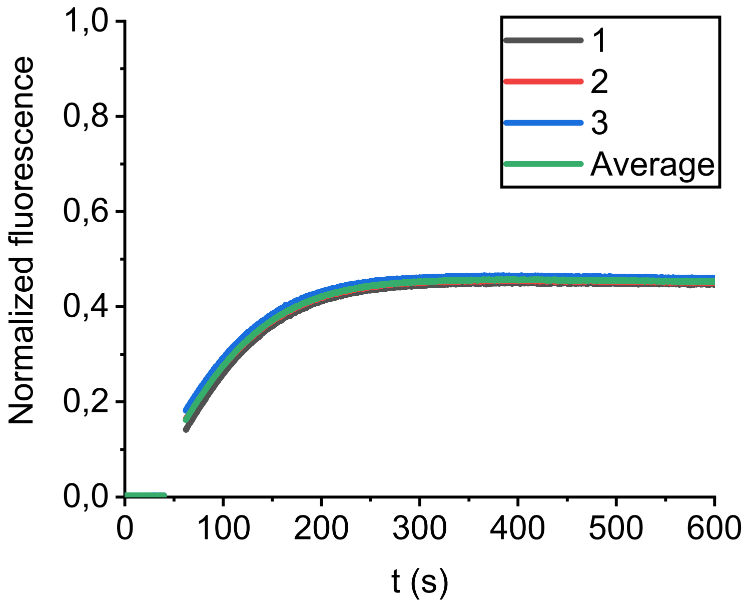

*Fig. S60. Normalized fluorescence intensity of host (R,R,R,R)-**H₂3** as a function of time after dilution induced decomplexation of (R,R,R,R)-**H₂3**·(R,R)-**15** (addition at t = 50 s, c = 2.5 × 10^{-7} M in $CHCl_3/CH_3CN$, 1:1, v/v, 298 K). The experiment was performed in triplicate.*

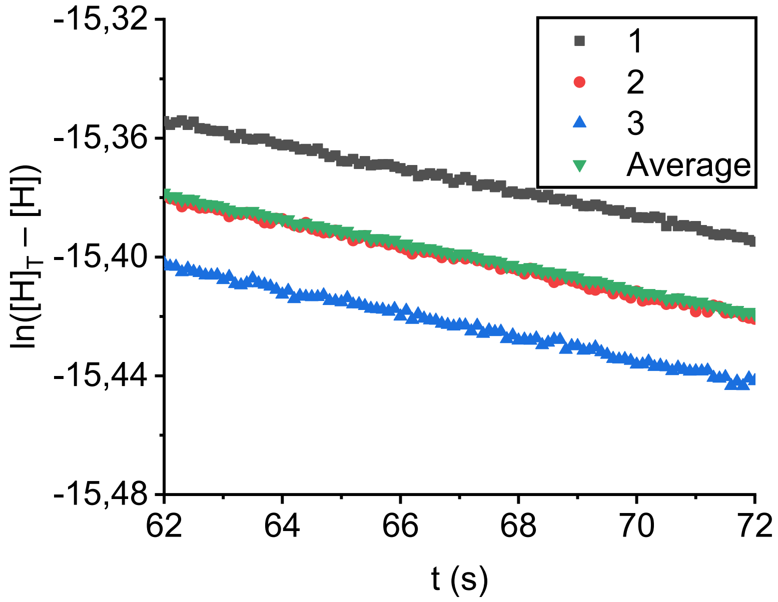

*Fig. S61. First-order kinetics plots for the decomplexation of host-guest complex (R,R,R,R)-**H₂3**·(R,R)-**15** (c = 2.5 × 10^{-7} M in $CHCl_3/CH_3CN$, 1:1, v/v, 298 K).*

*Table S40. Kinetic data for the dethreading of guest (R,R)-**15** from host (R,R,R,R)-**H₂3**.*

**Entry**	**Host**	**Guest**	**k_{off} (× 10^{-3} s^{-1})**	**R^2**
1	(*R*,*R*,*R*,*R*)-**$H_2$3**	(*R*,*R*)-**15**	4.01	0.997
2	(*R*,*R*,*R*,*R*)-**$H_2$3**	(*R*,*R*)-**15**	4.05	0.995
3	(*R*,*R*,*R*,*R*)-**$H_2$3**	(*R*,*R*)-**15**	3.98	0.994
**AVERAGE**	(*R*,*R*,*R*,*R*)-**$H_2$3**	(*R*,*R*)-**15**	4.01	0.999

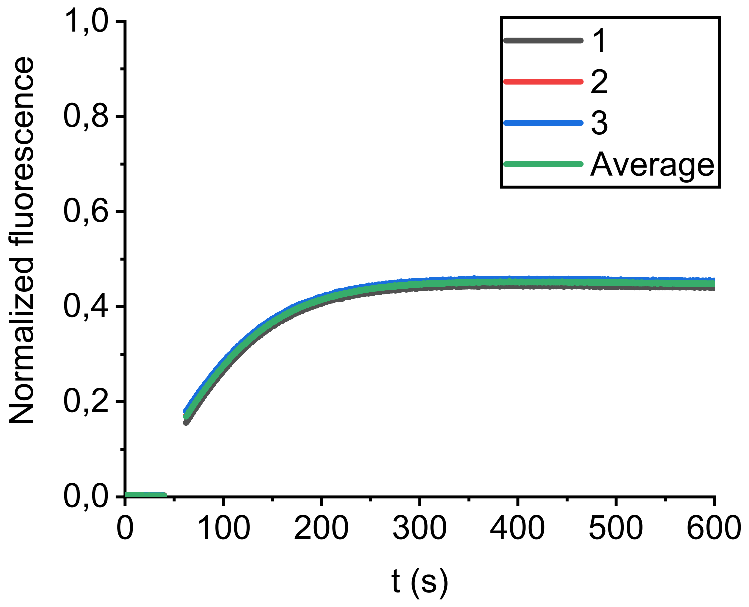

*Fig. S62. Normalized fluorescence intensity of host (S,S,S,S)-**H₂3** as a function of time after dilution induced decomplexation of (S,S,S,S)-**H₂3**·(R,R)-**15** (addition at t = 50 s, c = 2.5 × 10^{-7} M in $CHCl_3/CH_3CN$, 1:1, v/v, 298 K). The experiment was performed in triplicate.*

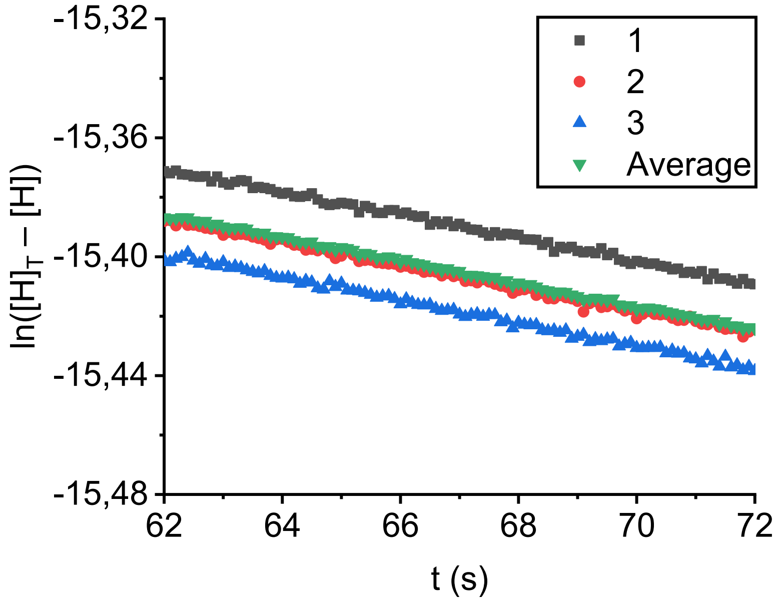

*Fig. S63. First-order kinetics plots for the decomplexation of host-guest complex (S,S,S,S)-**H₂3**·(R,R)-**15** (c = 2.5 × 10^{-7} M in $CHCl_3/CH_3CN$, 1:1, v/v, 298 K).*

*Table S41. Kinetic data for the dethreading of guest (R,R)-**15** from host (S,S,S,S)-**H₂3**.*

**Entry**	**Host**	**Guest**	**k_{off} (× 10^{-3} s^{-1})**	**R^2**
1	(*S*,*S*,*S*,*S*)-**$H_2$3**	(*R*,*R*)-**15**	3.87	0.995
2	(*S*,*S*,*S*,*S*)-**$H_2$3**	(*R*,*R*)-**15**	3.83	0.995
3	(*S*,*S*,*S*,*S*)-**$H_2$3**	(*R*,*R*)-**15**	3.86	0.994
**AVERAGE**	(*S*,*S*,*S*,*S*)-**$H_2$3**	(*R*,*R*)-**15**	3.85	0.998

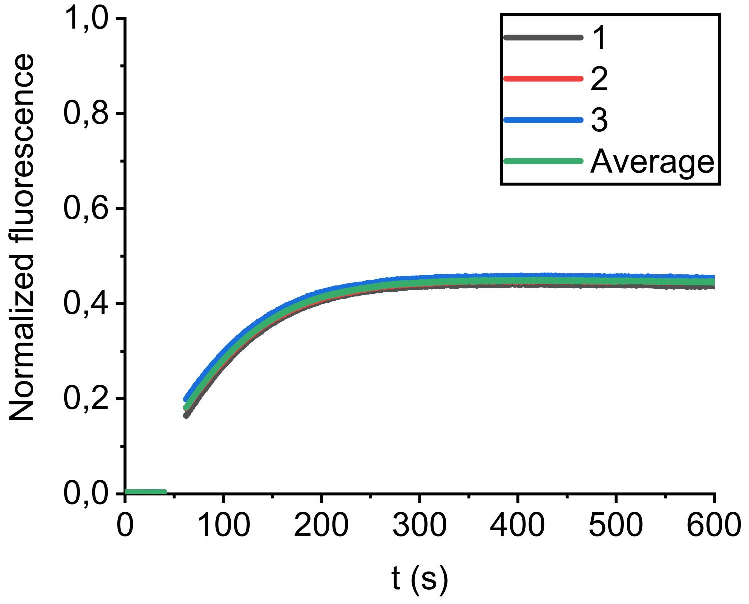

*Fig. S64. Normalized fluorescence intensity of host (R,R,R,R)-**$H_2$3** as a function of time after dilution induced decomplexation of (R,R,R,R)-**$H_2$3**·(S,S)-**15** (addition at t = 50 s, c = 2.5 × 10^{-7} M in $CHCl_3$/CH_3CN, 1:1, v/v, 298 K). The experiment was performed in triplicate.*

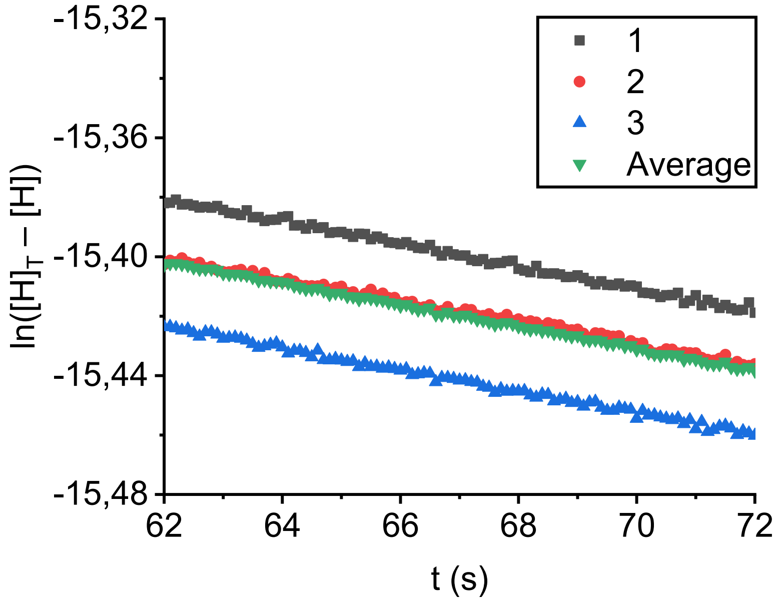

*Fig. S65. First-order kinetics plots for the decomplexation of host-guest complex (R,R,R,R)-**$H_2$3**·(S,S)-**15** (c = 2.5 × 10^{-7} M in $CHCl_3$/CH_3CN, 1:1, v/v, 298 K).*

*Table S42. Kinetic data for the dethreading of guest (S,S)-**15** from host (R,R,R,R)-**$H_2$3**.*

**Entry**	**Host**	**Guest**	**k_{off} (× 10^{-3} s^{-1})**	**R^2**
1	(*R*,*R*,*R*,*R*)-**$H_2$3**	(*S*,*S*)-**15**	3.79	0.994
2	(*R*,*R*,*R*,*R*)-**$H_2$3**	(*S*,*S*)-**15**	3.57	0.993
3	(*R*,*R*,*R*,*R*)-**$H_2$3**	(*S*,*S*)-**15**	3.64	0.994
**AVERAGE**	(*R*,*R*,*R*,*R*)-**$H_2$3**	(*S*,*S*)-**15**	3.67	0.998

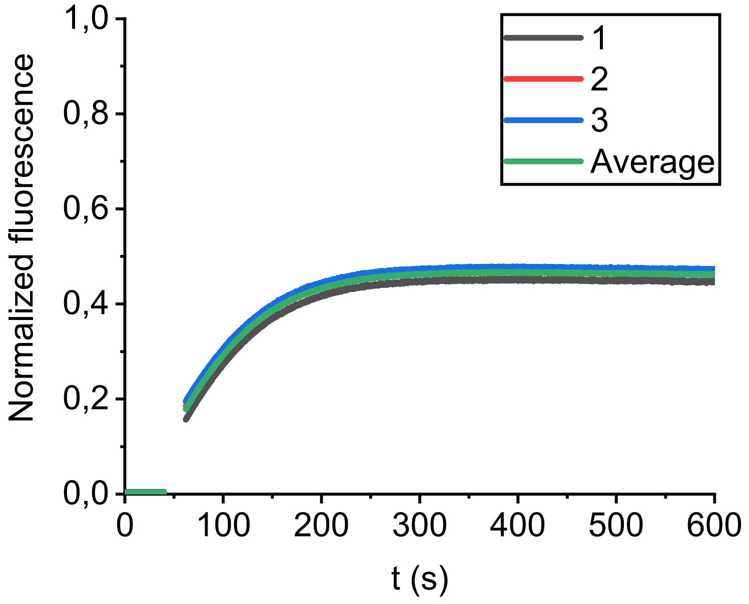

*Fig. S66. Normalized fluorescence intensity of host (S,S,S,S)-**H₂3** as a function of time after dilution induced decomplexation of (S,S,S,S)-**H₂3**·(S,S)-**15** (addition at t = 50 s, c = 2.5 × 10⁻⁷ M in CHCl₃/CH₃CN, 1:1, v/v, 298 K). The experiment was performed in triplicate.*

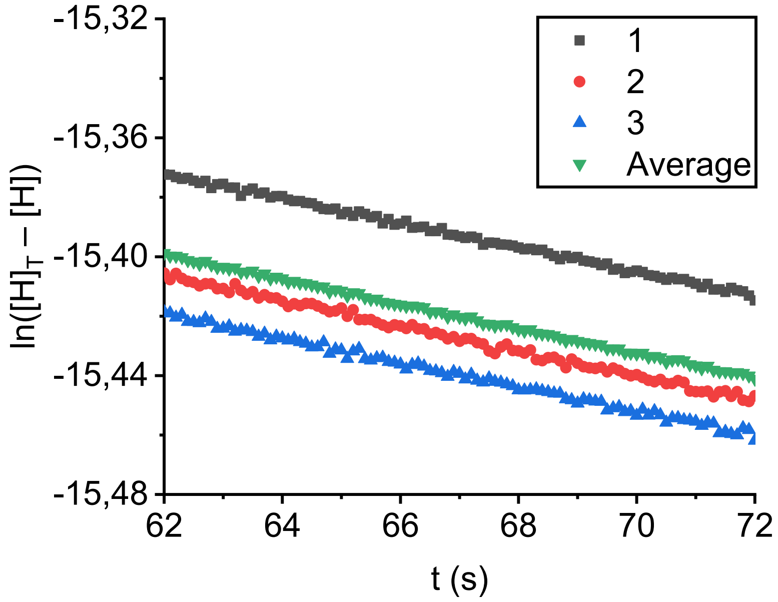

*Fig. S67. First-order kinetics plots for the decomplexation of host-guest complex (S,S,S,S)-**H₂3**·(S,S)-**15** (c = 2.5 × 10⁻⁷ M in CHCl₃/CH₃CN, 1:1, v/v, 298 K).*

*Table S43. Kinetic data for the dethreading of guest (S,S)-**15** from host (S,S,S,S)-**H₂3**.*

**Entry**	**Host**	**Guest**	k_{off} **(× 10^{-3} s^{-1})**	R^2
1	(*S*,*S*,*S*,*S*)-H_2**3**	(*S*,*S*)-**15**	4.11	0.997
2	(*S*,*S*,*S*,*S*)-H_2**3**	(*S*,*S*)-**15**	4.24	0.994
3	(*S*,*S*,*S*,*S*)-H_2**3**	(*S*,*S*)-**15**	4.08	0.994
**AVERAGE**	(*S*,*S*,*S*,*S*)-H_2**3**	(*S*,*S*)-**15**	4.14	0.999

*Dethreading of **16** from H_2**1** or H_2**3**.*

Solvent mixture (3 mL, $CHCl_3/CH_3CN$, 1:1, v/v, 298 K) was added to a 1 cm quartz cuvette and the cuvette was placed inside the fluorescence spectrometer. The fluorescence intensity (λ_{ex} = 416 nm, λ_{em} = 647 nm) was monitored as a function of time (10 datapoints per second). At t ≈ 40 s, the cuvette was removed from the spectrometer. At t ≈ 50 s, a solution of **H$_2$1·16** or **H$_2$3·16** (3 µL, c = 10^{-4} M, 1:1, host:guest) in the same solvent mixture was added to the cuvette. The contents of the cuvette were shaken vigorously for 5 seconds. At t ≈ 60 s, the cuvette was placed back inside the spectrometer. At t = 600 s, the experiment was stopped. The fluorescence intensity of the host was normalized to the average fluorescence intensity of host in a separate experiment in which **H$_2$1** or **H$_2$3** (3 µL, c = 10^{-4} M) was added to the solvent mixture (3 mL, $CHCl_3/CH_3CN$, 1:1, v/v, 298 K). Each combination of host and guest was tested in triplicate. The kinetic plots for the dethreading of (*R*,*R*)-**16** from **H$_2$1** are depicted in Fig. S68–S69 and the results are summarized in Table S44. The kinetic plots for the dethreading of (*R*,*R*)-**16** from (*R*,*R*,*R*,*R*)-**H$_2$3** are depicted in Fig. S70–S71 and the results are summarized in Table S45. The kinetic plots for the dethreading of (*R*,*R*)-**16** from (*S*,*S*,*S*,*S*)-**H$_2$3** are depicted in Fig. S72–S73 and the results are summarized in Table S46. The kinetic plots for the dethreading of (*S*,*S*)-**16** from **H$_2$1** are depicted in Fig. S74–S75 and the results are summarized in Table S47. The kinetic plots for the dethreading of (*S*,*S*)-**16** from (*R*,*R*,*R*,*R*)-**H$_2$3** are depicted in Fig. S76–S77 and the results are summarized in Table S48. The kinetic plots for the dethreading of (*S*,*S*)-**16** from (*S*,*S*,*S*,*S*)-**H$_2$3** are depicted in Fig. S78–S79 and the results are summarized in Table S49.

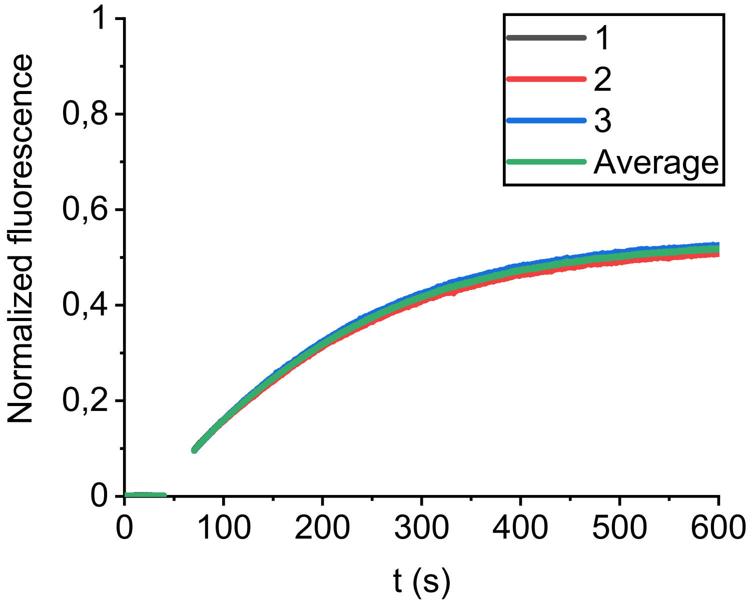

Fig. S68. Normalized fluorescence intensity of host ***$H_2$1*** *as a function of time after dilution induced decomplexation of* ***$H_2$1***·*(R,R)-***16** *(addition at t = 50 s, c = 10^{-7} M in $CHCl_3$/CH_3CN, 1:1, v/v, 298 K). The experiment was performed in triplicate.*

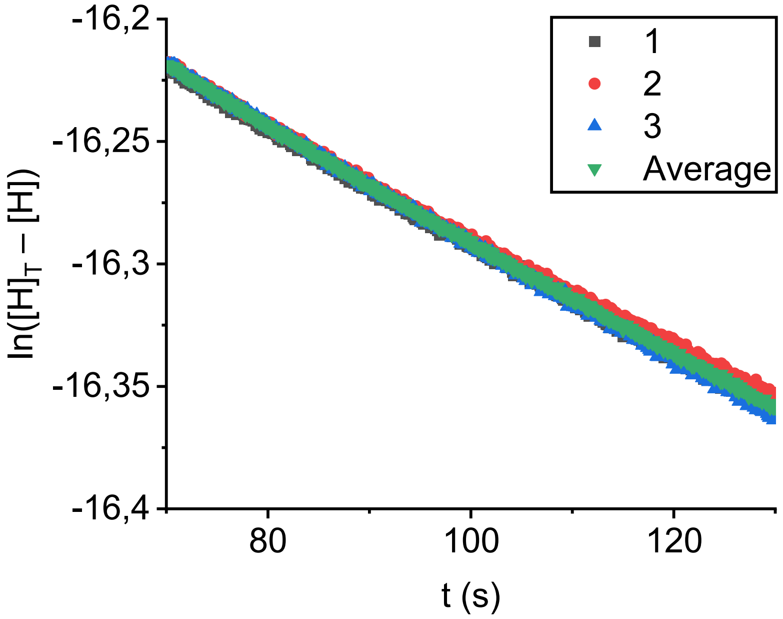

Fig. S69. First-order kinetics plots for the decomplexation of host-guest complex ***$H_2$1***·*(R,R)-***16** *(c = 10^{-7} M in $CHCl_3$/CH_3CN, 1:1, v/v, 298 K).*

*Table S44. Kinetic data for the dethreading of guest (R,R)-***16** *from host* ***$H_2$1***.

**Entry**	**Host**	**Guest**	**k_{off} (× 10^{-3} s^{-1})**	**R^2**
1	**$H_2$1**	(*R*,*R*)-**16**	2.32	0.999
2	**$H_2$1**	(*R*,*R*)-**16**	2.26	0.998
3	**$H_2$1**	(*R*,*R*)-**16**	2.39	0.999
**AVERAGE**	**$H_2$1**	(*R*,*R*)-**16**	2.32	0.999

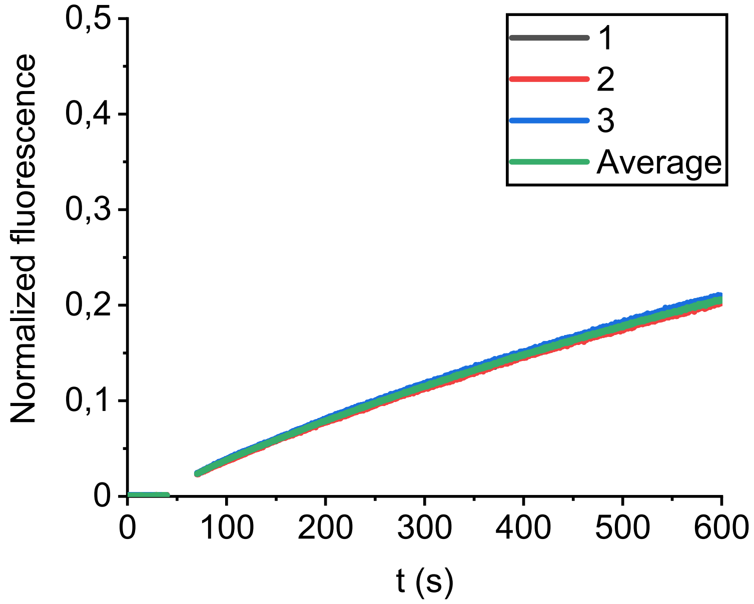

*Fig. S70. Normalized fluorescence intensity of host (R,R,R,R)-**$H_2$3** as a function of time after dilution induced decomplexation of (R,R,R,R)-**$H_2$3**·(R,R)-**16** (addition at t = 50 s, c = 10^{-7} M in $CHCl_3$/CH_3CN, 1:1, v/v, 298 K). The experiment was performed in triplicate.*

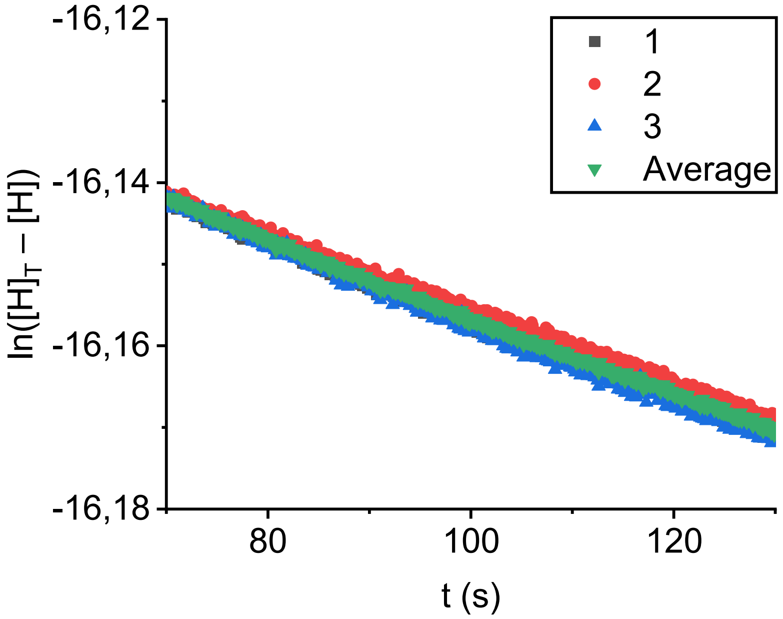

*Fig. S71. First-order kinetics plots for the decomplexation of host-guest complex (R,R,R,R)-**$H_2$3**·(R,R)-**16** (c = 10^{-7} M in $CHCl_3$/CH_3CN, 1:1, v/v, 298 K).*

*Table S45. Kinetic data for the dethreading of guest (R,R)-**16** from host (R,R,R,R)-**$H_2$3**.*

**Entry**	**Host**	**Guest**	**k_{off} (× 10^{-4} s^{-1})**	**R^2**
1	(*R*,*R*,*R*,*R*)-**$H_2$3**	(*R*,*R*)-**16**	4.75	0.996
2	(*R*,*R*,*R*,*R*)-**$H_2$3**	(*R*,*R*)-**16**	4.56	0.997
3	(*R*,*R*,*R*,*R*)-**$H_2$3**	(*R*,*R*)-**16**	4.87	0.995
**AVERAGE**	(*R*,*R*,*R*,*R*)-**$H_2$3**	(*R*,*R*)-**16**	4.73	0.998

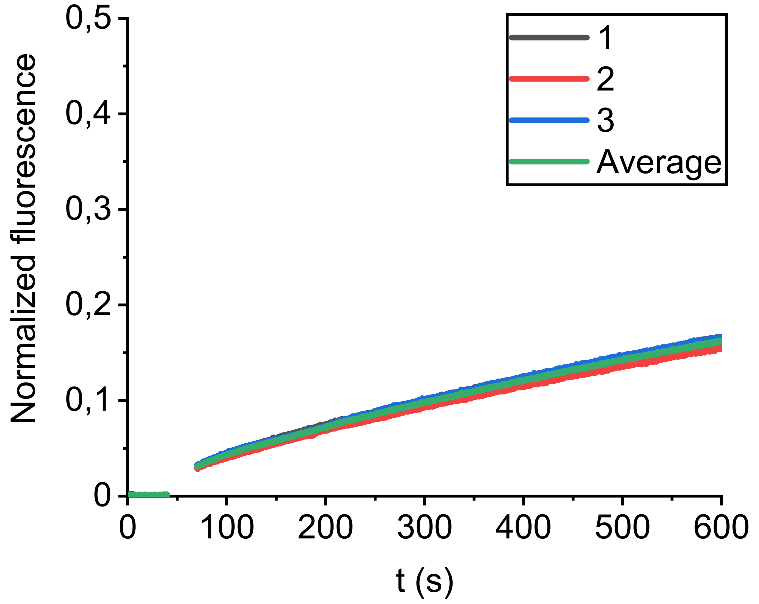

*Fig. S72. Normalized fluorescence intensity of host (S,S,S,S)-**H₂3** as a function of time after dilution induced decomplexation of (S,S,S,S)-**H₂3**·(R,R)-**16** (addition at t = 50 s, c = 10^{-7} M in $CHCl_3/CH_3CN$, 1:1, v/v, 298 K). The experiment was performed in triplicate.*

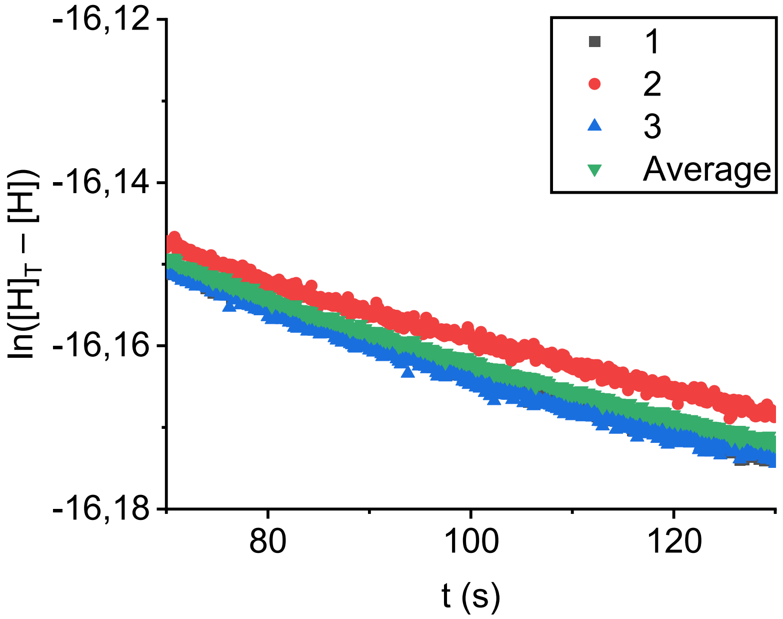

*Fig. S73. First-order kinetics plots for the decomplexation of host-guest complex (S,S,S,S)-**H₂3**·(R,R)-**16** (c = 10^{-7} M in $CHCl_3/CH_3CN$, 1:1, v/v, 298 K).*

*Table S46. Kinetic data for the dethreading of guest (R,R)-**16** from host (S,S,S,S)-**H₂3**.*

**Entry**	**Host**	**Guest**	k_{off} **(× 10^{-4} s^{-1})**	R^2
1	(S,S,S,S)-**$H_2$3**	(R,R)-**16**	3.89	0.992
2	(S,S,S,S)-**$H_2$3**	(R,R)-**16**	3.37	0.989
3	(S,S,S,S)-**$H_2$3**	(R,R)-**16**	3.84	0.986
**AVERAGE**	(S,S,S,S)-**$H_2$3**	(R,R)-**16**	3.70	0.993

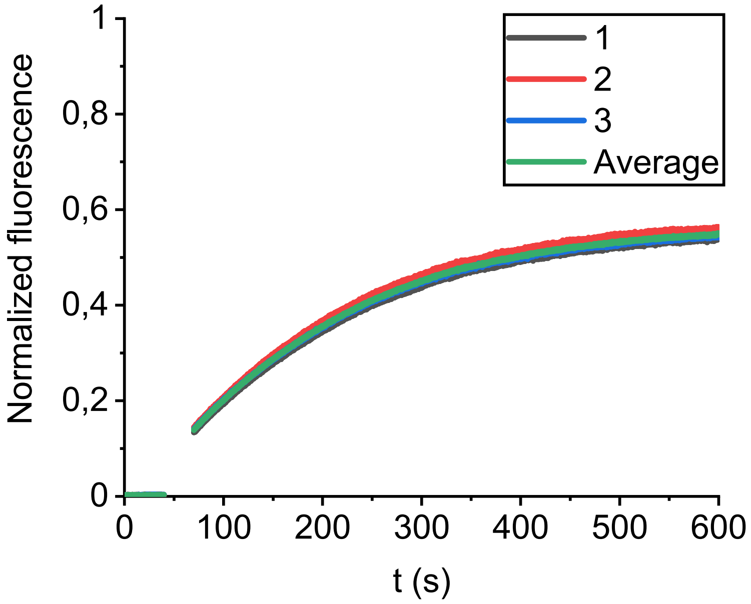

Fig. S74. Normalized fluorescence intensity of host ***$H_2$1*** *as a function of time after dilution induced decomplexation of* ***$H_2$1***·*(S,S)-***16** *(addition at t = 50 s, c = 10^{-7} M in $CHCl_3$/CH_3CN, 1:1, v/v, 298 K). The experiment was performed in triplicate.*

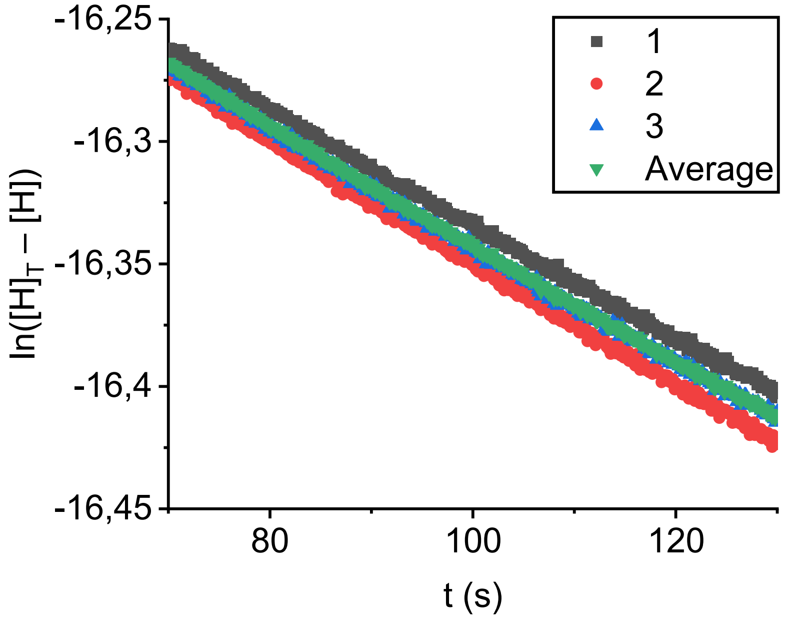

Fig. S75. First-order kinetics plots for the decomplexation of host-guest complex ***$H_2$1***·*(S,S)-***16** *(c = 10^{-7} M in $CHCl_3$/CH_3CN, 1:1, v/v, 298 K).*

*Table S47. Kinetic data for the dethreading of guest (S,S)-***16** *from host* ***$H_2$1***.

**Entry**	**Host**	**Guest**	**k_{off} (× 10^{-3} s^{-1})**	**R^2**
1	**$H_2$1**	(*S*,*S*)-**16**	2.34	0.999
2	**$H_2$1**	(*S*,*S*)-**16**	2.48	0.999
3	**$H_2$1**	(*S*,*S*)-**16**	2.39	0.999
**AVERAGE**	**$H_2$1**	(*S*,*S*)-**16**	2.41	0.999

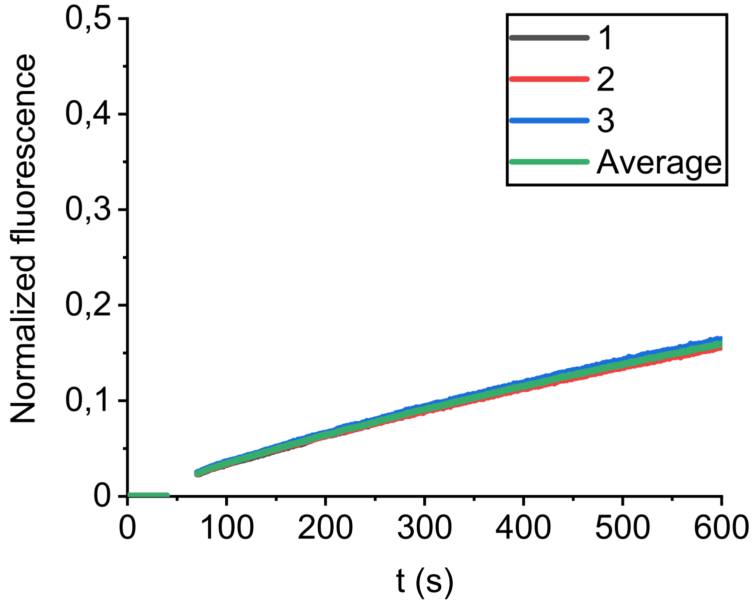

*Fig. S76. Normalized fluorescence intensity of host (R,R,R,R)-**$H_2$3** as a function of time after dilution induced decomplexation of (R,R,R,R)-**$H_2$3**·(S,S)-**16** (addition at t = 50 s, c = 10^{-7} M in $CHCl_3$/CH_3CN, 1:1, v/v, 298 K). The experiment was performed in triplicate.*

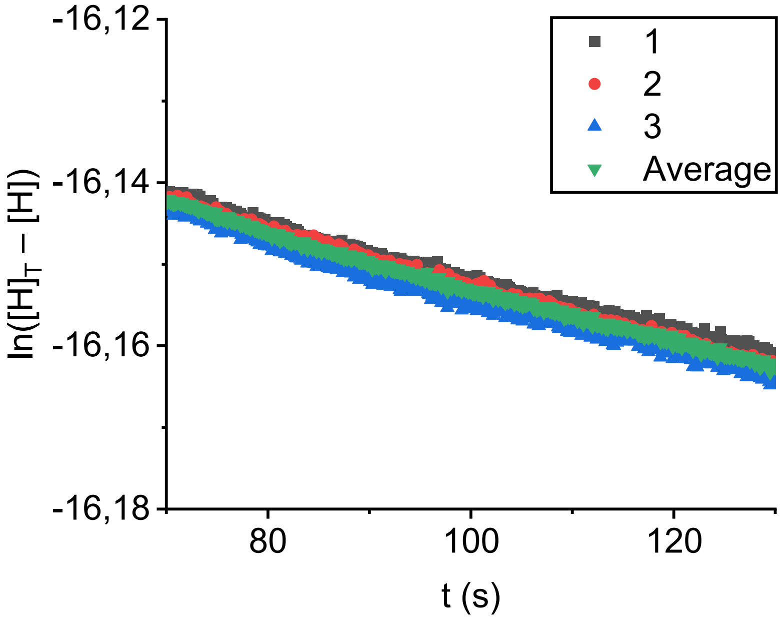

*Fig. S77. First-order kinetics plots for the decomplexation of host-guest complex (R,R,R,R)-**$H_2$3**·(S,S)-**16** (c = 10^{-7} M in $CHCl_3$/CH_3CN, 1:1, v/v, 298 K).*

*Table S48. Kinetic data for the dethreading of guest (S,S)-**16** from host (R,R,R,R)-**$H_2$3**.*

**Entry**	**Host**	**Guest**	**k_{off} (× 10^{-4} s^{-1})**	**R^2**
1	(*R,R,R,R*)-**$H_2$3**	(*S,S*)-**16**	3.29	0.992
2	(*R,R,R,R*)-**$H_2$3**	(*S,S*)-**16**	3.41	0.994
3	(*R,R,R,R*)-**$H_2$3**	(*S,S*)-**16**	3.35	0.987
**AVERAGE**	(*R,R,R,R*)-**$H_2$3**	(*S,S*)-**16**	3.35	0.995

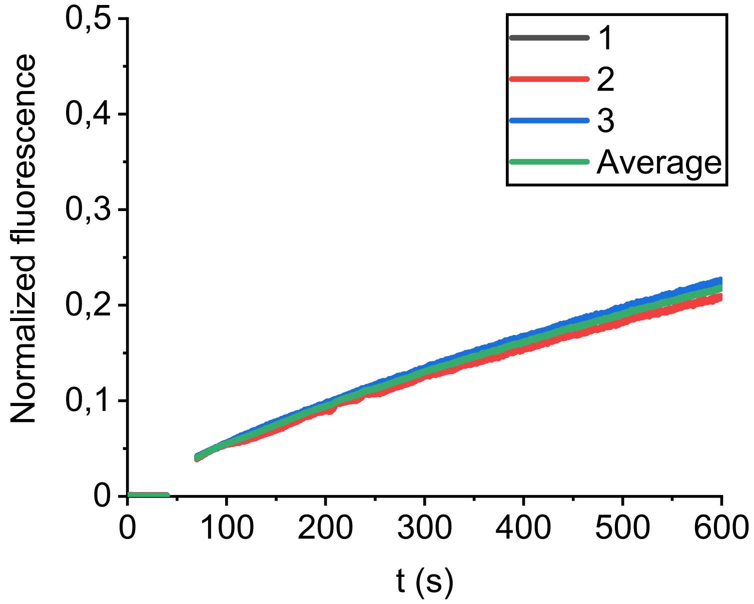

*Fig. S78. Normalized fluorescence intensity of host (S,S,S,S)-**$H_2$3** as a function of time after dilution induced decomplexation of (S,S,S,S)-**$H_2$3**·(S,S)-**16** (addition at t = 50 s, c = 10^{-7} M in $CHCl_3/CH_3CN$, 1:1, v/v, 298 K). The experiment was performed in triplicate.*

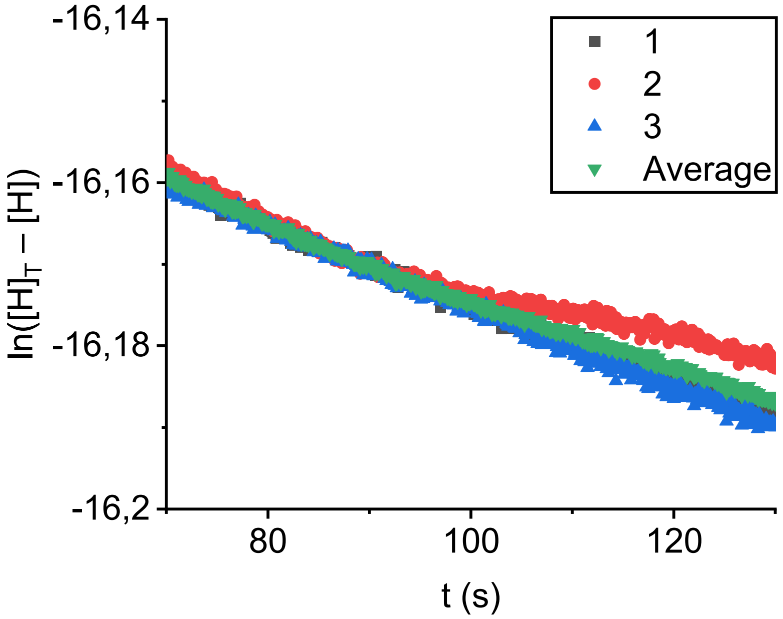

*Fig. S79. First-order kinetics plots for the decomplexation of host-guest complex (S,S,S,S)-**$H_2$3**·(S,S)-**16** (c = 10^{-7} M in $CHCl_3/CH_3CN$, 1:1, v/v, 298 K).*

*Table S49. Kinetic data for the dethreading of guest (S,S)-**16** from host (S,S,S,S)-**$H_2$3**.*

Entry	Host	Guest	k_{off} (× 10^{-4} s^{-1})	R^2
1	(*S,S,S,S*)-**$H_2$3**	(*S,S*)-**16**	4.75	0.995
2	(*S,S,S,S*)-**$H_2$3**	(*S,S*)-**16**	3.63	0.954
3	(*S,S,S,S*)-**$H_2$3**	(*S,S*)-**16**	4.92	0.996
**AVERAGE**	(*S,S,S,S*)-**$H_2$3**	(*S,S*)-**16**	4.43	0.993

Dethreading of 17 from $H_2$1 or $H_2$3.

Solvent mixture (3 mL, $CHCl_3/CH_3CN$, 1:1, v/v, 298 K) was added to a 1 cm quartz cuvette and the cuvette was placed inside the fluorescence spectrometer. The fluorescence intensity (λ_{ex} = 416 nm, λ_{em} = 647 nm) was monitored as a function of time (10 datapoints per second). At t ≈ 40 s, the cuvette was removed from the spectrometer. At t ≈ 50 s, a solution of **$H_2$1·17** or **$H_2$3·17** (3 µL, c = 10^{-4} M, 1:1, host:guest) in the same solvent mixture was added to the cuvette. The contents of the cuvette were shaken vigorously for 5 seconds. At t ≈ 60 s, the cuvette was placed back inside the spectrometer. At t = 600 s, the experiment was stopped. The fluorescence intensity of the host was normalized to the average fluorescence intensity of host in a separate experiment in which **$H_2$1** or **$H_2$3** (3 µL, c = 10^{-4} M) was added to the solvent mixture (3 mL, $CHCl_3/CH_3CN$, 1:1, v/v, 298 K). Each combination of host and guest was tested in triplicate. The kinetic plots for the dethreading of (*R*,*R*)-**17** from **$H_2$1** are depicted in Fig. S80–S81 and the results are summarized in Table S50. The kinetic plots for the dethreading of (*R*,*R*)-**17** from (*R*,*R*,*R*,*R*)-**$H_2$3** are depicted in Fig. S82–S83 and the results are summarized in Table S51. The kinetic plots for the dethreading of (*R*,*R*)-**17** from (*S*,*S*,*S*,*S*)-**$H_2$3** are depicted in Fig. S84–S85 and the results are summarized in Table S52. The kinetic plots for the dethreading of (*S*,*S*)-**17** from **$H_2$1** are depicted in Fig. S86–S87 and the results are summarized in Table S53. The kinetic plots for the dethreading of (*S*,*S*)-**17** from (*R*,*R*,*R*,*R*)-**$H_2$3** are depicted in Fig. S88–S89 and the results are summarized in Table S54. The kinetic plots for the dethreading of (*S*,*S*)-**17** from (*S*,*S*,*S*,*S*)-**$H_2$3** are depicted in Fig. S90–S91 and the results are summarized in Table S55.

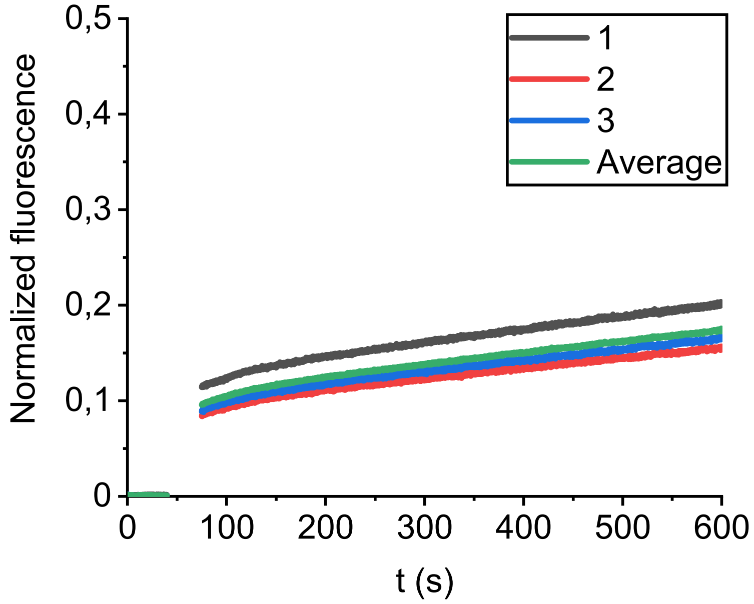

Fig. S80. Normalized fluorescence intensity of host ***$H_2$1*** *as a function of time after dilution induced decomplexation of* ***$H_2$1***·*(R,R)-***17** *(addition at t = 50 s, c = 10^{-7} M in $CHCl_3/CH_3CN$, 1:1, v/v, 298 K). The experiment was performed in triplicate.*

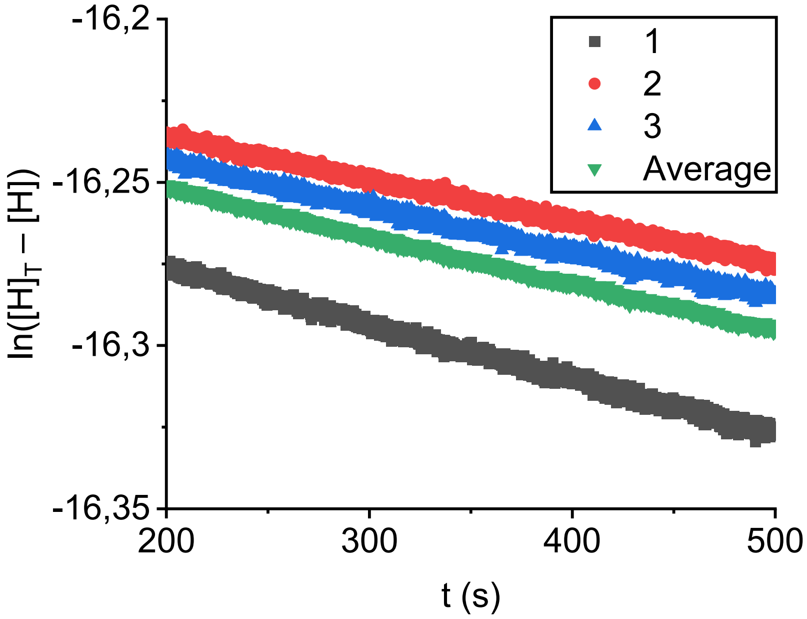

Fig. S81. First-order kinetics plots for the decomplexation of host-guest complex ***$H_2$1***·*(R,R)-***17** *(c = 10^{-7} M in $CHCl_3/CH_3CN$, 1:1, v/v, 298 K).*

*Table S50. Kinetic data for the dethreading of guest (R,R)-***17** *from host* ***$H_2$1***.

**Entry**	**Host**	**Guest**	**k_{off} (× 10^{-4} s^{-1})**	**R^2**
1	**$H_2$1**	(*R*,*R*)-**17**	1.69	0.994
2	**$H_2$1**	(*R*,*R*)-**17**	1.29	0.993
3	**$H_2$1**	(*R*,*R*)-**17**	1.39	0.993
**AVERAGE**	**$H_2$1**	(*R*,*R*)-**17**	1.46	0.998

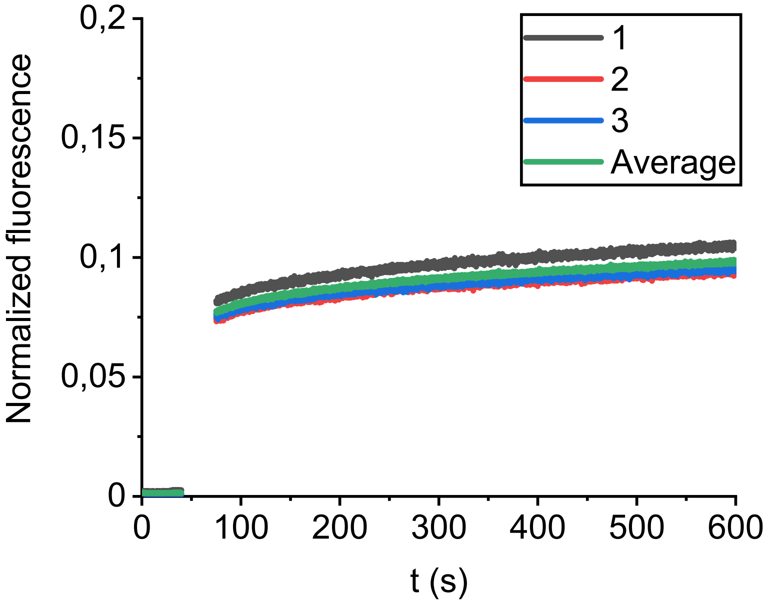

*Fig. S82. Normalized fluorescence intensity of host (R,R,R,R)-**H₂3** as a function of time after dilution induced decomplexation of (R,R,R,R)-**H₂3**·(R,R)-**17** (addition at t = 50 s, c = 10^{-7} M in $CHCl_3/CH_3CN$, 1:1, v/v, 298 K). The experiment was performed in triplicate.*

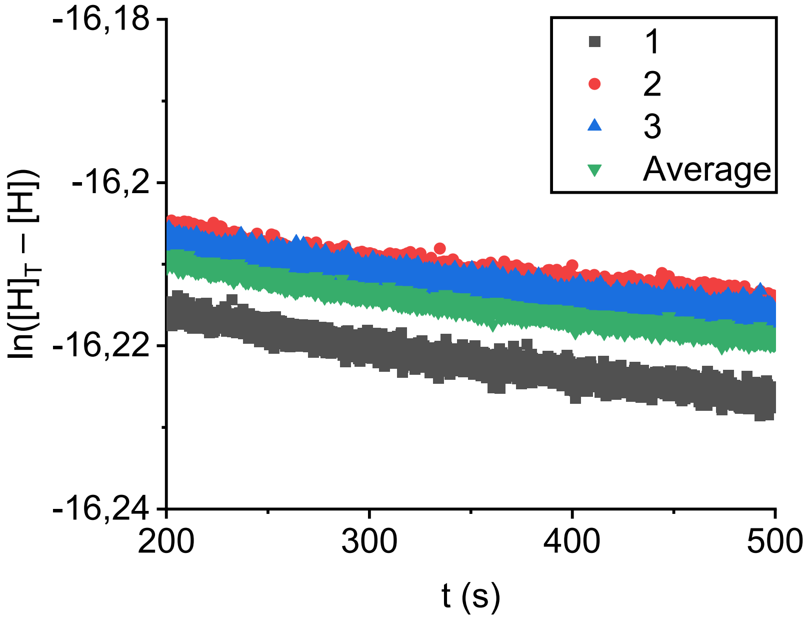

*Fig. S83. First-order kinetics plots for the decomplexation of host-guest complex (R,R,R,R)-**H₂3**·(R,R)-**17** (c = 10^{-7} M in $CHCl_3/CH_3CN$, 1:1, v/v, 298 K).*

*Table S51. Kinetic data for the dethreading of guest (R,R)-**17** from host (R,R,R,R)-**H₂3**.*

**Entry**	**Host**	**Guest**	k_{off} **(× 10^{-5} s^{-1})**	R^2
1	(*R*,*R*,*R*,*R*)-$\mathbf{H_23}$	(*R*,*R*)-**17**	3.53	0.931
2	(*R*,*R*,*R*,*R*)-$\mathbf{H_23}$	(*R*,*R*)-**17**	2.85	0.908
3	(*R*,*R*,*R*,*R*)-$\mathbf{H_23}$	(*R*,*R*)-**17**	2.99	0.915
**AVERAGE**	(*R*,*R*,*R*,*R*)-$\mathbf{H_23}$	(*R*,*R*)-**17**	3.12	0.964

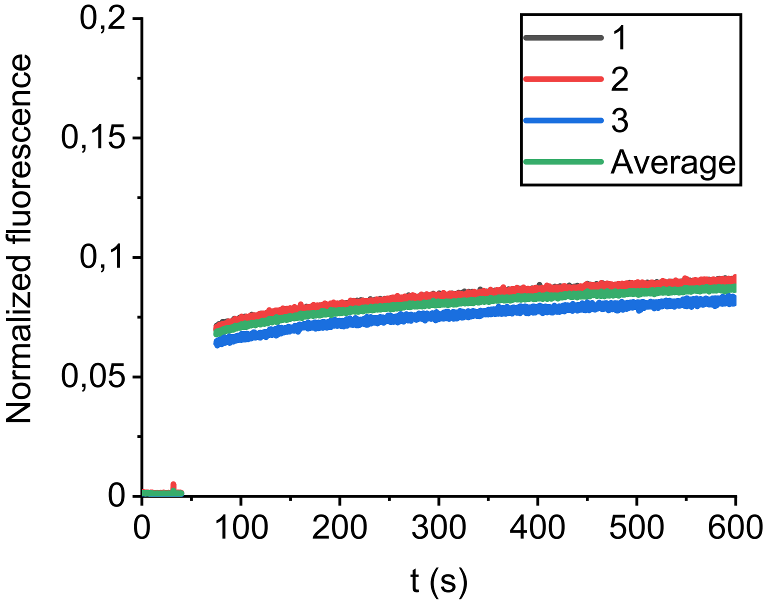

*Fig. S84. Normalized fluorescence intensity of host (S,S,S,S)-**H$_2$3** as a function of time after dilution induced decomplexation of (S,S,S,S)-**H$_2$3**·(R,R)-**17** (addition at t = 50 s, c = 10^{-7} M in $CHCl_3/CH_3CN$, 1:1, v/v, 298 K). The experiment was performed in triplicate.*

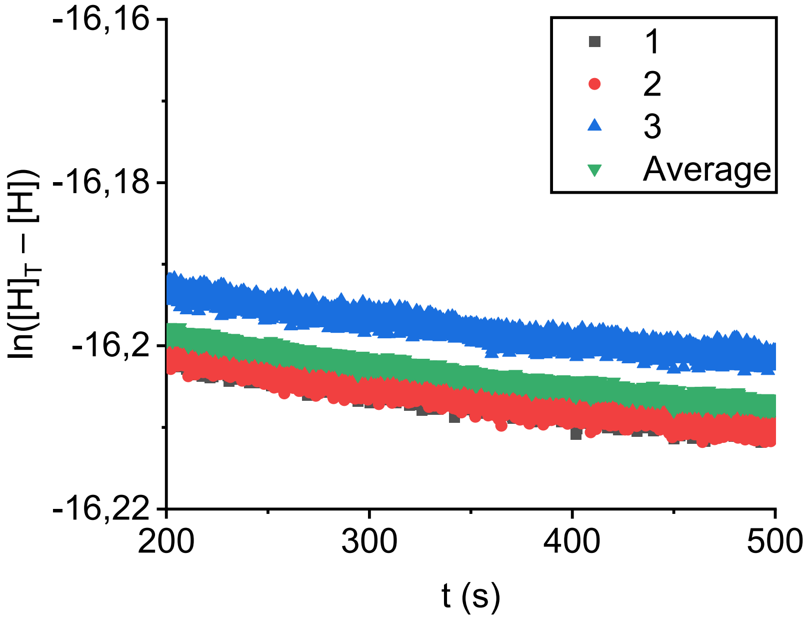

*Fig. S85. First-order kinetics plots for the decomplexation of host-guest complex (S,S,S,S)-**H$_2$3**·(R,R)-**17** (c = 10^{-7} M in $CHCl_3/CH_3CN$, 1:1, v/v, 298 K).*

*Table S52. Kinetic data for the dethreading of guest (R,R)-**17** from host (S,S,S,S)-**H$_2$3**.*

**Entry**	**Host**	**Guest**	**k_{off} (× 10^{-5} s^{-1})**	**R^2**
1	(*S,S,S,S*)-**H$_2$3**	(*R,R*)-**17**	2.97	0.922
2	(*S,S,S,S*)-**H$_2$3**	(*R,R*)-**17**	2.96	0.920
3	(*S,S,S,S*)-**H$_2$3**	(*R,R*)-**17**	2.73	0.917
**AVERAGE**	(*S,S,S,S*)-**H$_2$3**	(*R,R*)-**17**	2.89	0.966

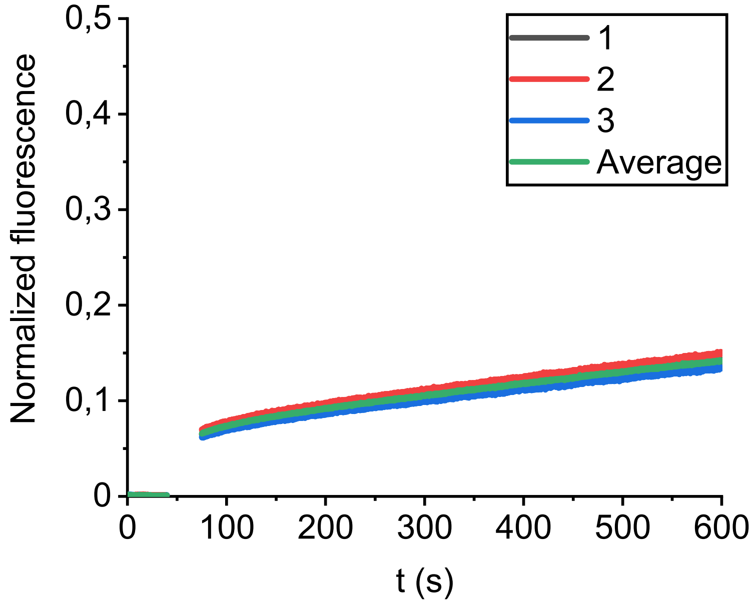

*Fig. S86. Normalized fluorescence intensity of host **$H_2$1** as a function of time after dilution induced decomplexation of **$H_2$1**·(S,S)-**17** (addition at t = 50 s, c = 10^{-7} M in $CHCl_3/CH_3CN$, 1:1, v/v, 298 K). The experiment was performed in triplicate.*

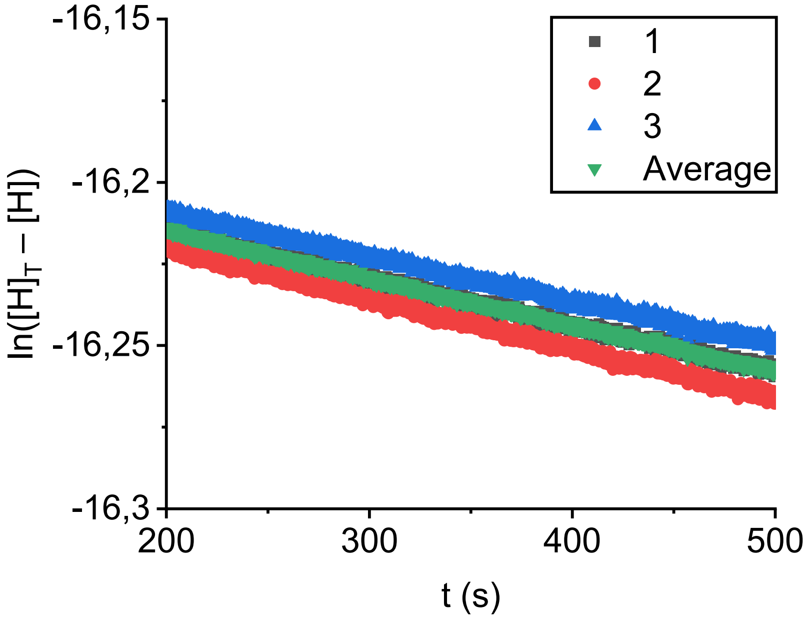

*Fig. S87. First-order kinetics plots for the decomplexation of host-guest complex **$H_2$1**·(S,S)-**17** (c = 10^{-7} M in $CHCl_3/CH_3CN$, 1:1, v/v, 298 K).*

*Table S53. Kinetic data for the dethreading of guest (S,S)-**17** from host **$H_2$1**.*

**Entry**	**Host**	**Guest**	**k_{off} (× 10^{-4} s^{-1})**	**R^2**
1	**$H_2$1**	(*S*,*S*)-**17**	1.43	0.995
2	**$H_2$1**	(*S*,*S*)-**17**	1.51	0.995
3	**$H_2$1**	(*S*,*S*)-**17**	1.36	0.995
**AVERAGE**	**$H_2$1**	(*S*,*S*)-**17**	1.43	0.998

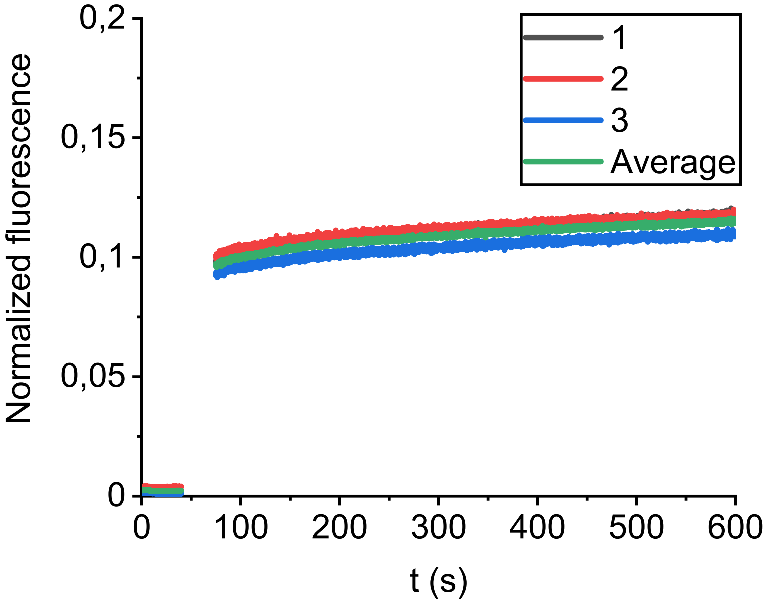

*Fig. S88. Normalized fluorescence intensity of host (R,R,R,R)-**$H_2$3** as a function of time after dilution induced decomplexation of (R,R,R,R)-**$H_2$3**·(S,S)-**17** (addition at t = 50 s, c = 10^{-7} M in $CHCl_3/CH_3CN$, 1:1, v/v, 298 K). The experiment was performed in triplicate.*

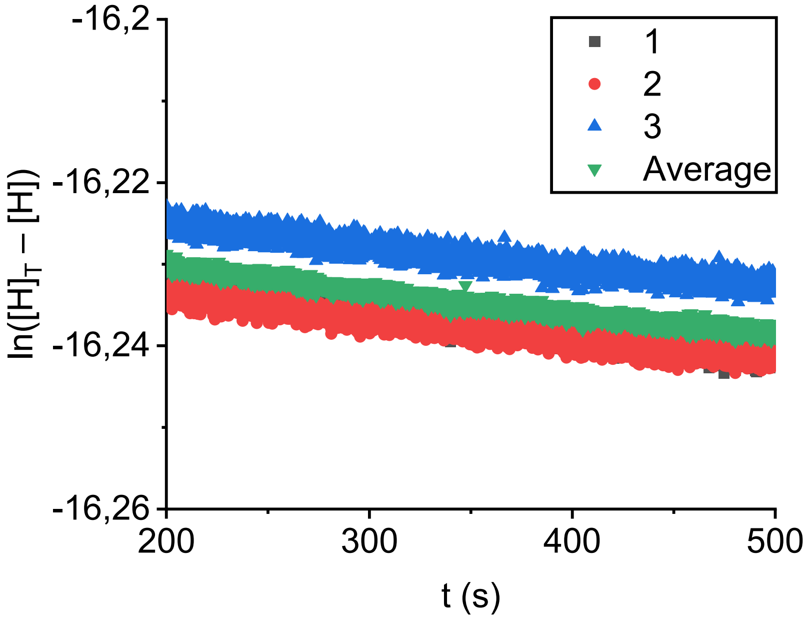

*Fig. S89. First-order kinetics plots for the decomplexation of host-guest complex (R,R,R,R)-**$H_2$3**·(S,S)-**17** (c = 10^{-7} M in $CHCl_3/CH_3CN$, 1:1, v/v, 298 K).*

*Table S54. Kinetic data for the dethreading of guest (S,S)-**17** from host (R,R,R,R)-**$H_2$3**.*

**Entry**	**Host**	**Guest**	k_{off} **(× 10^{-5} s^{-1})**	R^2
1	(*R*,*R*,*R*,*R*)-**$H_2$3**	(*S*,*S*)-**17**	3.22	0.915
2	(*R*,*R*,*R*,*R*)-**$H_2$3**	(*S*,*S*)-**17**	2.57	0.874
3	(*R*,*R*,*R*,*R*)-**$H_2$3**	(*S*,*S*)-**17**	2.57	0.878
**AVERAGE**	(*R*,*R*,*R*,*R*)-**$H_2$3**	(*S*,*S*)-**17**	2.79	0.958

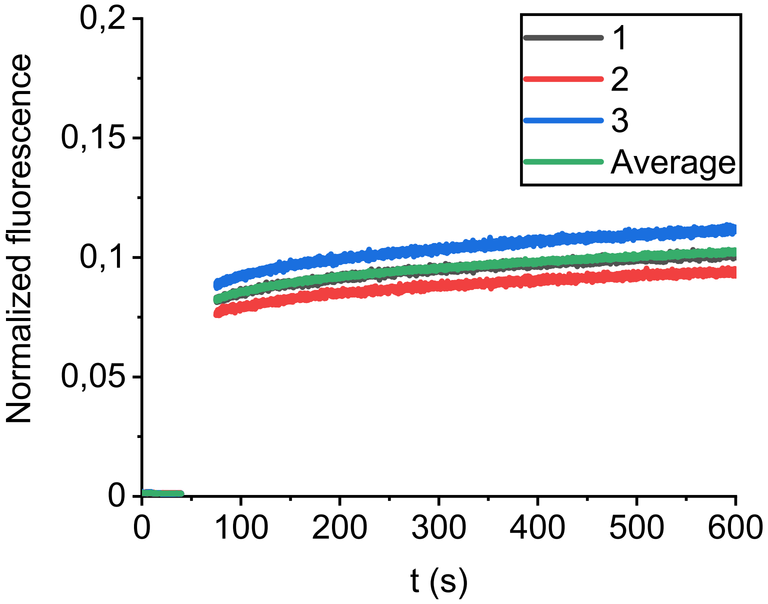

*Fig. S90. Normalized fluorescence intensity of host (S,S,S,S)-**$H_2$3** as a function of time after dilution induced decomplexation of (S,S,S,S)-**$H_2$3**·(S,S)-**17** (addition at t = 50 s, c = 10^{-7} M in $CHCl_3$/CH_3CN, 1:1, v/v, 298 K). The experiment was performed in triplicate.*

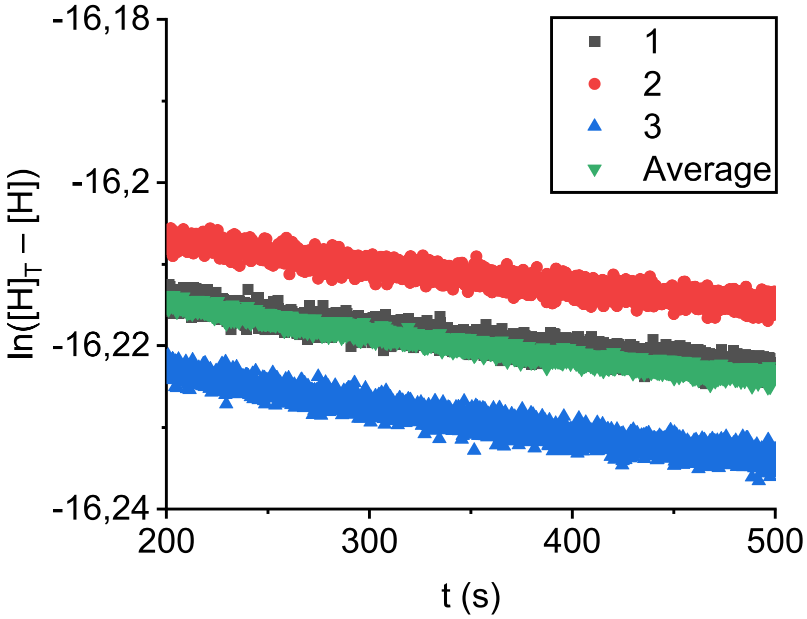

*Fig. S91. First-order kinetics plots for the decomplexation of host-guest complex (S,S,S,S)-**$H_2$3**·(S,S)-**17** (c = 10^{-7} M in $CHCl_3$/CH_3CN, 1:1, v/v, 298 K).*

*Table S55. Kinetic data for the dethreading of guest (S,S)-**17** from host (S,S,S,S)-**$H_2$3**.*

**Entry**	**Host**	**Guest**	k_{off} (× 10^{-5} s^{-1})	R^2
1	(*S,S,S,S*)-**$H_2$3**	(*S,S*)-**17**	2.81	0.901
2	(*S,S,S,S*)-**$H_2$3**	(*S,S*)-**17**	2.67	0.907
3	(*S,S,S,S*)-**$H_2$3**	(*S,S*)-**17**	3.64	0.931
**AVERAGE**	(*S,S,S,S*)-**$H_2$3**	(*S,S*)-**17**	3.04	0.964

Dethreading of ***18*** *from $H_2$1 or $H_2$3.*

Solvent mixture (3 mL, $CHCl_3/CH_3CN$, 1:1, v/v, 298 K) was added to a 1 cm quartz cuvette and the cuvette was placed inside the fluorescence spectrometer. The fluorescence intensity (λ_{ex} = 416 nm, λ_{em} = 647 nm) was monitored as a function of time (10 datapoints per second). At t ≈ 40 s, the cuvette was removed from the spectrometer. At t ≈ 50 s, a solution of **$H_2$1·18** or **$H_2$3·18** (3 µL, c = 10^{-4} M, 1:1, host:guest) in the same solvent mixture was added to the cuvette. The contents of the cuvette were shaken vigorously for 20 seconds. At t ≈ 75 s, the cuvette was placed back inside the spectrometer. At t = 600 s, the experiment was stopped. The fluorescence intensity of the host was normalized to the average fluorescence intensity of host in a separate experiment in which **$H_2$1** or **$H_2$3** (3 µL, c = 10^{-4} M) was added to the solvent mixture (3 mL, $CHCl_3/CH_3CN$, 1:1, v/v, 298 K). Each combination of host and guest was tested in triplicate. The kinetic plots for the dethreading of (*R*,*R*)-**18** from **$H_2$1** are depicted in Fig. S92–S93 and the results are summarized in Table S56. The kinetic plots for the dethreading of (*R*,*R*)-**18** from (*R*,*R*,*R*,*R*)-**$H_2$3** are depicted in Fig. S94–S95 and the results are summarized in Table S57. The kinetic plots for the dethreading of (*R*,*R*)-**18** from (*S*,*S*,*S*,*S*)-**$H_2$3** are depicted in Fig. S96–S97 and the results are summarized in Table S58. The kinetic plots for the dethreading of (*S*,*S*)-**18** from **$H_2$1** are depicted in Fig. S98–S99 and the results are summarized in Table S59. The kinetic plots for the dethreading of (*S*,*S*)-**18** from (*R*,*R*,*R*,*R*)-**$H_2$3** are depicted in Fig. S100–S101 and the results are summarized in Table S60. The kinetic plots for the dethreading of (*S*,*S*)-**18** from (*S*,*S*,*S*,*S*)-**$H_2$3** are depicted in Fig. S102–S103 and the results are summarized in Table S61.

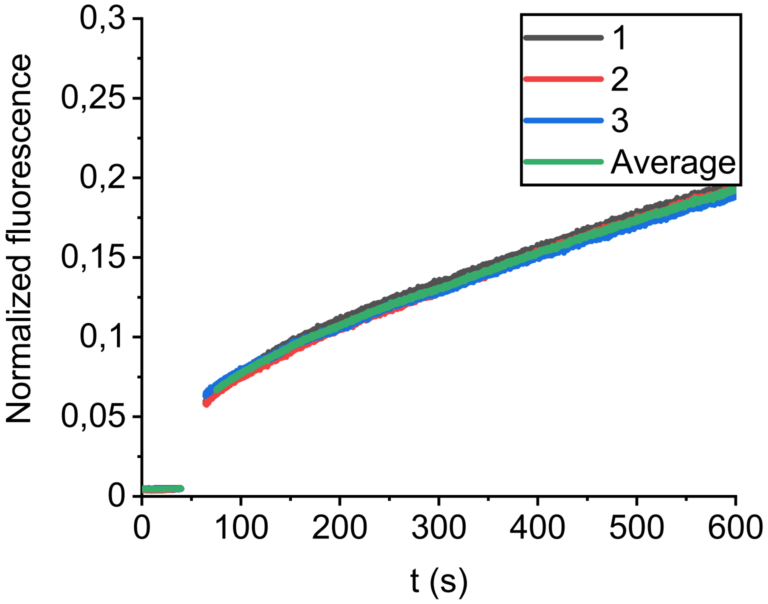

*Fig. S92. Normalized fluorescence intensity of host **$H_2$1** as a function of time after dilution induced decomplexation of **$H_2$1**·(R,R)-**18** (addition at t = 50 s, c = 10^{-7} M in $CHCl_3/CH_3CN$, 1:1, v/v, 298 K). The experiment was performed in triplicate.*

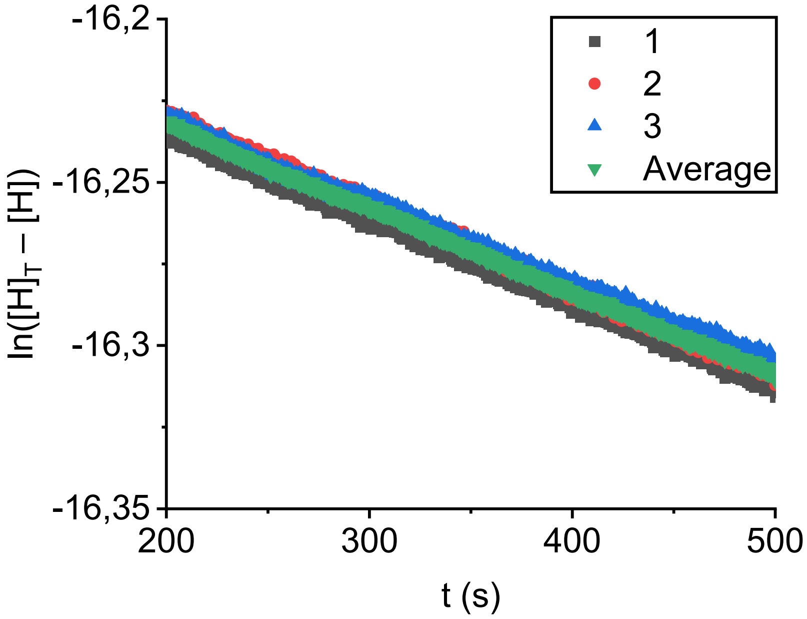

*Fig. S93. First-order kinetics plots for the decomplexation of host-guest complex **$H_2$1**·(R,R)-**18** (c = 10^{-7} M in $CHCl_3/CH_3CN$, 1:1, v/v, 298 K).*

*Table S56. Kinetic data for the dethreading of guest (R,R)-**18** from host **$H_2$1**.*

**Entry**	**Host**	**Guest**	**k_{off} ($\times 10^{-4}$ s^{-1})**	**R^2**
1	**$H_2$1**	(*R*,*R*)-**18**	2.55	0.998
2	**$H_2$1**	(*R*,*R*)-**18**	2.63	0.998
3	**$H_2$1**	(*R*,*R*)-**18**	2.47	0.997
**AVERAGE**	**$H_2$1**	(*R*,*R*)-**18**	2.55	0.999

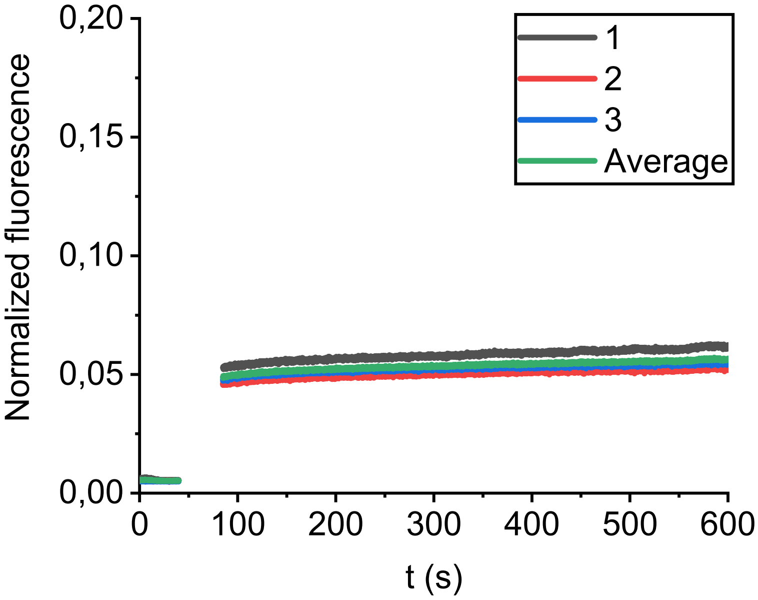

*Fig. S94. Normalized fluorescence intensity of host (R,R,R,R)-**$H_2$3** as a function of time after dilution induced decomplexation of (R,R,R,R)-**$H_2$3**·(R,R)-**18** (addition at t = 50 s, c = 10^{-7} M in $CHCl_3/CH_3CN$, 1:1, v/v, 298 K). The experiment was performed in triplicate.*

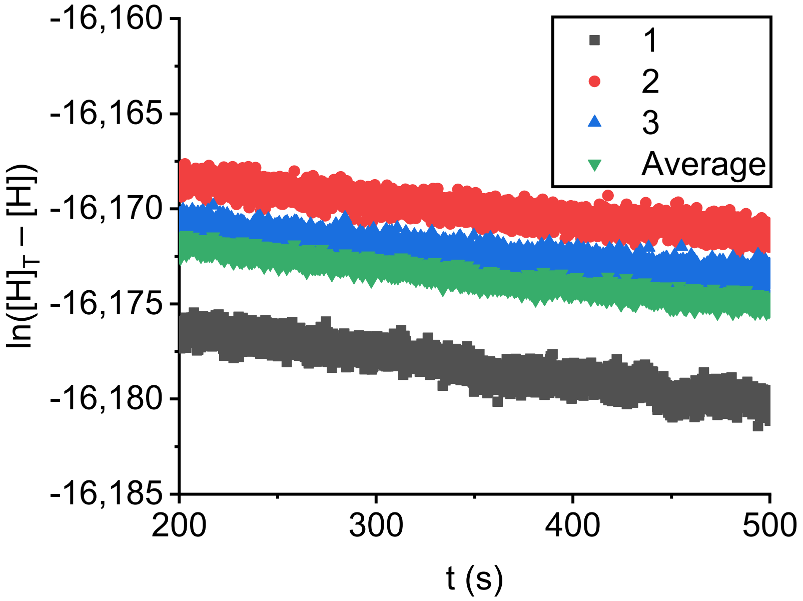

*Fig. S95. First-order kinetics plots for the decomplexation of host-guest complex (R,R,R,R)-**$H_2$3**·(R,R)-**18** (c = 10^{-7} M in $CHCl_3/CH_3CN$, 1:1, v/v, 298 K).*

*Table S57. Kinetic data for the dethreading of guest (R,R)-**18** from host (R,R,R,R)-**$H_2$3**.*

**Entry**	**Host**	**Guest**	k_{off} **(× 10^{-5} s^{-1})**	R^2
1	(*R*,*R*,*R*,*R*)-**$H_2$3**	(*R*,*R*)-**18**	1.33	0.873
2	(*R*,*R*,*R*,*R*)-**$H_2$3**	(*R*,*R*)-**18**	0.94	0.814
3	(*R*,*R*,*R*,*R*)-**$H_2$3**	(*R*,*R*)-**18**	0.92	0.794
**AVERAGE**	(*R*,*R*,*R*,*R*)-**$H_2$3**	(*R*,*R*)-**18**	1.06	0.933

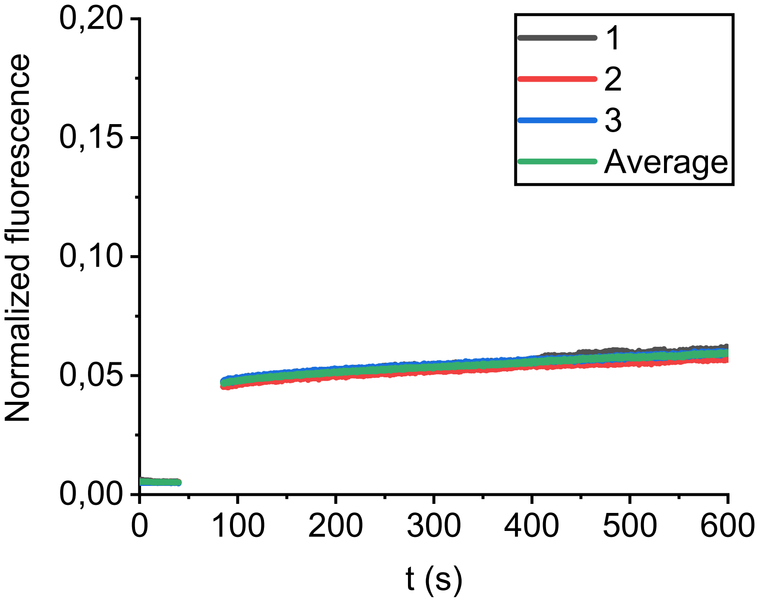

*Fig. S96. Normalized fluorescence intensity of host (S,S,S,S)-**$H_2$3** as a function of time after dilution induced decomplexation of (S,S,S,S)-**$H_2$3**·(R,R)-**18** (addition at t = 50 s, c = 10^{-7} M in $CHCl_3/CH_3CN$, 1:1, v/v, 298 K). The experiment was performed in triplicate.*

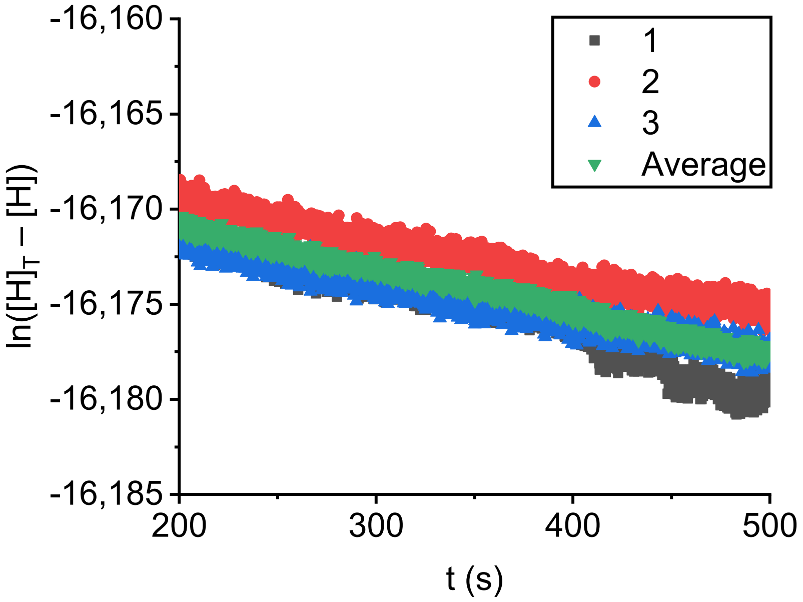

*Fig. S97. First-order kinetics plots for the decomplexation of host-guest complex (S,S,S,S)-**$H_2$3**·(R,R)-**18** (c = 10^{-7} M in $CHCl_3/CH_3CN$, 1:1, v/v, 298 K).*

*Table S58. Kinetic data for the dethreading of guest (R,R)-**18** from host (S,S,S,S)-**$H_2$3**.*

**Entry**	**Host**	**Guest**	**k_{off} (× 10^{-5} s^{-1})**	**R^2**
1	(*S*,*S*,*S*,*S*)-**$H_2$3**	(*R*,*R*)-**18**	2.83	0.941
2	(*S*,*S*,*S*,*S*)-**$H_2$3**	(*R*,*R*)-**18**	2.02	0.944
3	(*S*,*S*,*S*,*S*)-**$H_2$3**	(*R*,*R*)-**18**	1.93	0.943
**AVERAGE**	(*S*,*S*,*S*,*S*)-**$H_2$3**	(*R*,*R*)-**18**	2.26	0.984

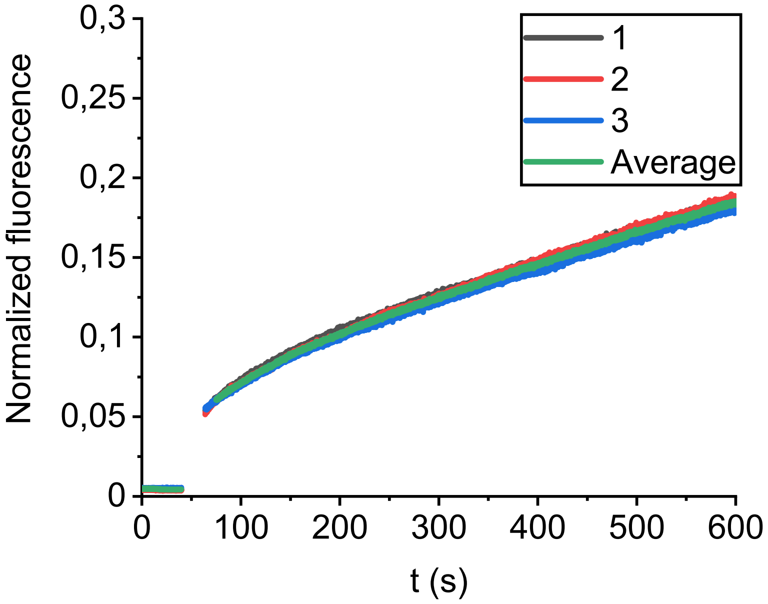

*Fig. S98. Normalized fluorescence intensity of host **$H_2$1** as a function of time after dilution induced decomplexation of **$H_2$1**·(S,S)-**18** (addition at t = 50 s, c = 10^{-7} M in $CHCl_3/CH_3CN$, 1:1, v/v, 298 K). The experiment was performed in triplicate.*

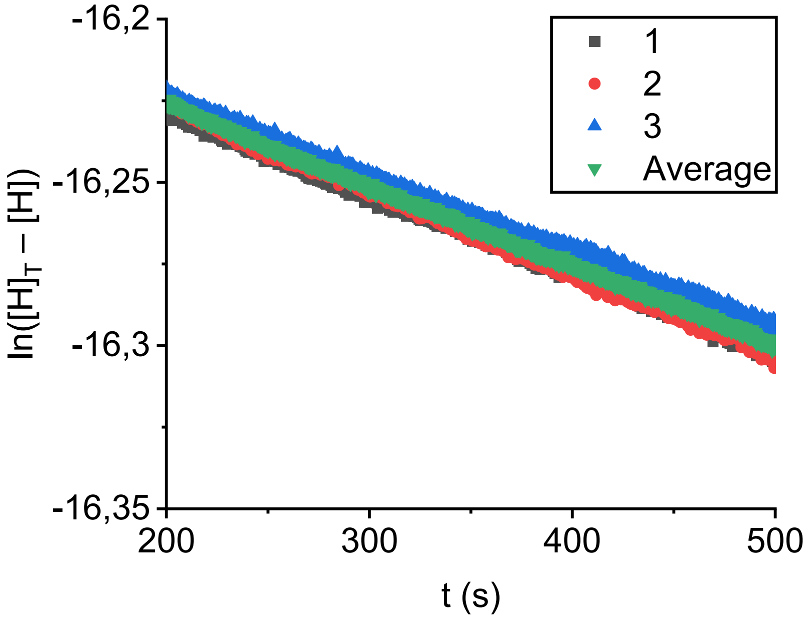

*Fig. S99. First-order kinetics plots for the decomplexation of host-guest complex **$H_2$1**·(S,S)-**18** (c = 10^{-7} M in $CHCl_3/CH_3CN$, 1:1, v/v, 298 K).*

*Table S59. Kinetic data for the dethreading of guest (S,S)-**18** from host **$H_2$1**.*

**Entry**	**Host**	**Guest**	**k_{off} (× 10^{-4} s^{-1})**	**R^2**
1	**$H_2$1**	(*S*,*S*)-**18**	2.42	0.997
2	**$H_2$1**	(*S*,*S*)-**18**	2.55	0.997
3	**$H_2$1**	(*S*,*S*)-**18**	2.35	0.997
**AVERAGE**	**$H_2$1**	(*S*,*S*)-**18**	2.44	0.999

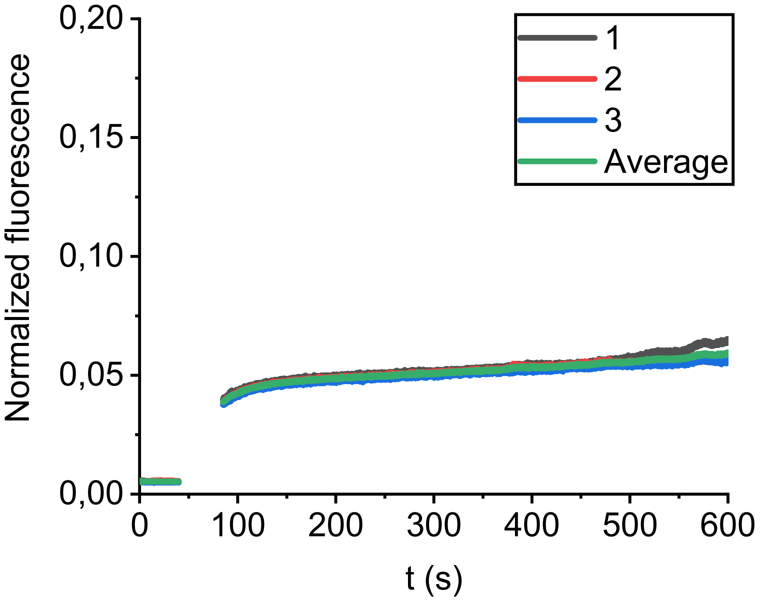

*Fig. S100. Normalized fluorescence intensity of host (R,R,R,R)-**$H_2$3** as a function of time after dilution induced decomplexation of (R,R,R,R)-**$H_2$3**·(S,S)-**18** (addition at t = 50 s, c = 10^{-7} M in $CHCl_3/CH_3CN$, 1:1, v/v, 298 K). The experiment was performed in triplicate.*

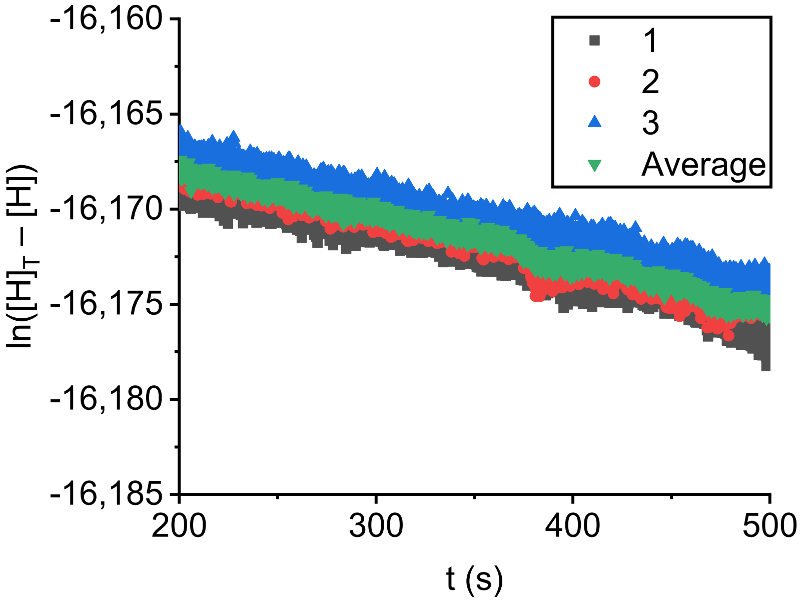

*Fig. S101. First-order kinetics plots for the decomplexation of host-guest complex (R,R,R,R)-**$H_2$3**·(S,S)-**18** (c = 10^{-7} M in $CHCl_3/CH_3CN$, 1:1, v/v, 298 K).*

*Table S60. Kinetic data for the dethreading of guest (S,S)-**18** from host (R,R,R,R)-**$H_2$3**.*

**Entry**	**Host**	**Guest**	k_{off} **(× 10^{-5} s^{-1})**	R^2
1	(*R*,*R*,*R*,*R*)-**$H_2$3**	(*S*,*S*)-**18**	2.47	0.954
2	(*R*,*R*,*R*,*R*)-**$H_2$3**	(*S*,*S*)-**18**	2.50	0.955
3	(*R*,*R*,*R*,*R*)-**$H_2$3**	(*S*,*S*)-**18**	2.31	0.959
**AVERAGE**	(*R*,*R*,*R*,*R*)-**$H_2$3**	(*S*,*S*)-**18**	2.43	0.984

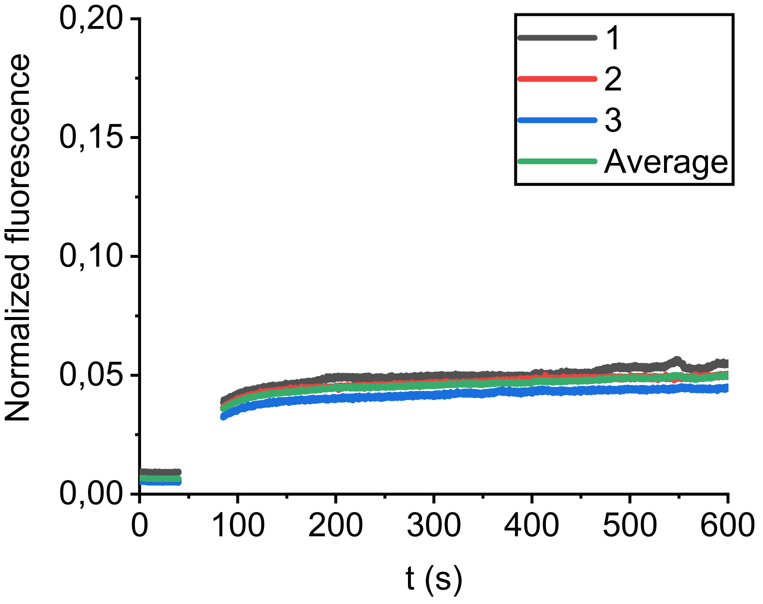

*Fig. S102. Normalized fluorescence intensity of host (S,S,S,S)-**H₂3** as a function of time after dilution induced decomplexation of (S,S,S,S)-**H₂3**·(S,S)-**18** (addition at t = 50 s, c = 10^{-7} M in $CHCl_3/CH_3CN$, 1:1, v/v, 298 K). The experiment was performed in triplicate.*

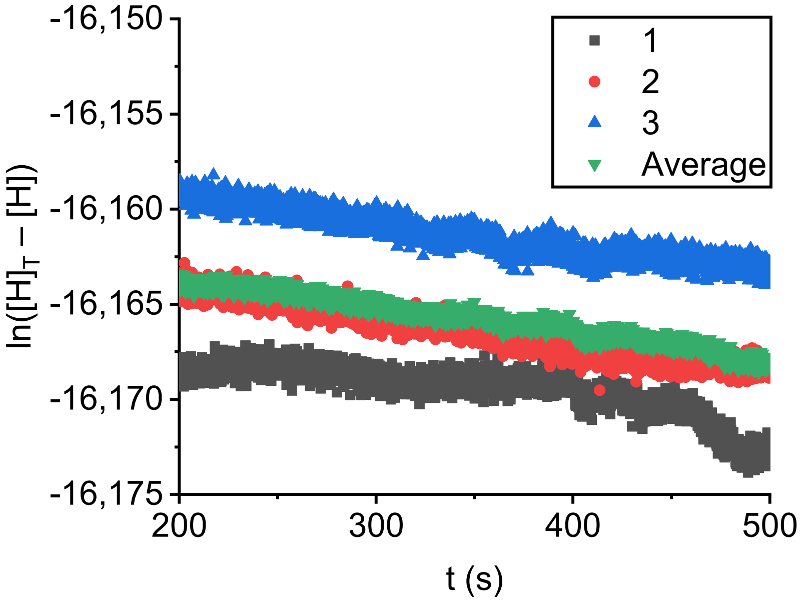

*Fig. S103. First-order kinetics plots for the decomplexation of host-guest complex (S,S,S,S)-**H₂3**·(S,S)-**18** (c = 10^{-7} M in $CHCl_3/CH_3CN$, 1:1, v/v, 298 K).*

*Table S61. Kinetic data for the dethreading of guest (S,S)-**18** from host (S,S,S,S)-**H₂3**.*

**Entry**	**Host**	**Guest**	k_{off} **(× 10^{-5} s^{-1})**	R^2
1	(*S*,*S*,*S*,*S*)-**H₂3**	(*S*,*S*)-**18**	1.25	0.690
2	(*S*,*S*,*S*,*S*)-**H₂3**	(*S*,*S*)-**18**	1.56	0.921
3	(*S*,*S*,*S*,*S*)-**H₂3**	(*S*,*S*)-**18**	1.35	0.883
**AVERAGE**	(*S*,*S*,*S*,*S*)-**H₂3**	(*S*,*S*)-**18**	1.38	0.956

2.2.6. NMR host-guest titration

A solution of host (*S,S,S,S*)-**H₂3** (0.6 mL, c = 10^{-3} M in $CDCl_3/CD_3CN$, 1:1, v/v, 298 K) was titrated with a solution containing guest **13** (c = 5 × 10^{-3} M) and host (*S,S,S,S*)-**H₂3** (c = 10^{-3} M) according to Table S62. Stacked ^{1}H NMR spectra (500 MHz) of the titration of (*S,S,S,S*)-**H₂3** with **13** are depicted in Fig. S104. The addition of aliquots of guest **13** initially resulted in broadening of the host signals, caused by rapid exchange of free host and host-guest complexes. The signals of the host became sharp again when more than 1 equivalent of guest was added, indicative of the formation of a 1:1 complex. Most host signals showed small changes upon binding the guest. The signals of the methyl groups, on the contrary, showed a remarkably large downfield shift from −1.00/−1.17 ppm to 1.05/0.59 ppm. The complexation induced shift (CIS) values for relevant proton signals of all the investigated host-guest complexes are provided in the next section.

Table S62. NMR titration scheme.

Exp #	$V_{initial}$ (µL)	V_{added} (µL)	V_{final} (µL)	[host] (× 10^{-3} M)	[guest] (× 10^{-3} M)
1	600	0	600	1	0
2	600	25	625	1	0.20
3	625	25	650	1	0.38
4	650	25	675	1	0.56
5	675	25	700	1	0.71
6	700	25	725	1	0.86
7	725	25	750	1	1.00
8	750	25	775	1	1.13
9	775	25	800	1	1.25
10	–	–	–	0	1

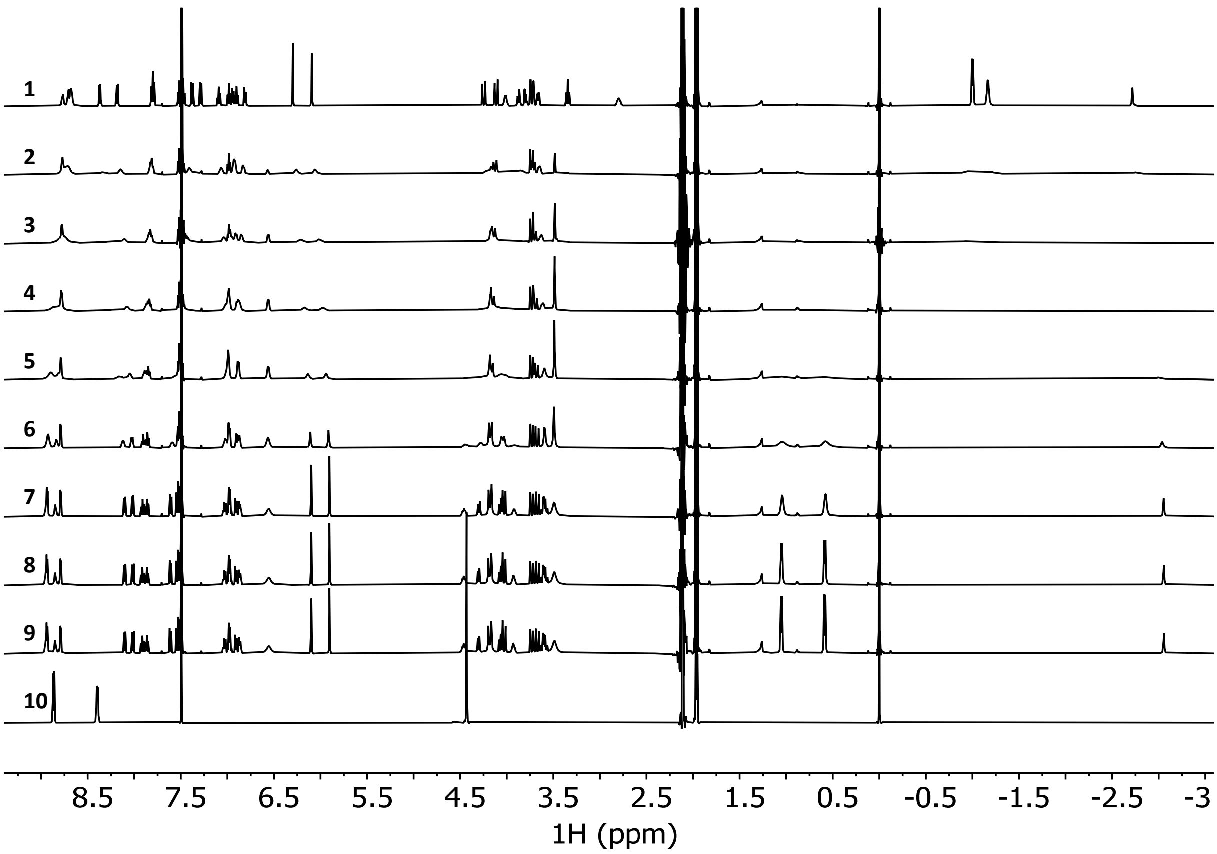

*Fig. S104. Stacked ^{1}H NMR spectra (500 MHz, $CDCl_3/CD_3CN$, 1:1, v/v, 298 K) of the titration of (S,S,S,S)-**H₂3** with **13**. See Table S62 for the host and guest concentrations in the spectra of solutions 1-10.*

2.2.7. NMR characterization of 1:1 host-guest complexes

Host-guest mixtures were prepared in $CDCl_3/CD_3CN$, 1:1, v/v. Except for the host-guest mixtures (*R*,*R*,*R*,*R*)-**H₂3·13** (1:2, host:guest) and (*S*,*S*,*S*,*S*)-**H₂3·13** (1:1.25, host:guest), the mixtures were prepared such that the molar ratio of host and guest was ~1:1. NMR characterizations (^{1}H, COSY, ROESY, HSQC) were recorded for all host-guest complexes derived from (*S*,*S*,*S*,*S*)-**$H_2$3**. For the host-guest complexes derived from (*R*,*R*,*R*,*R*)-**$H_2$3** only ^{1}H NMR spectra were recorded, which were identical to the ^{1}H NMR spectra corresponding to the enantiomeric (*S*,*S*,*S*,*S*)-**$H_2$3** complexes (Fig. S105–S110). CIS-values for relevant host protons H^a–H^k and guest protons H^l–H^m are provided in Tables S63–67.

Achiral guests 13 and 14

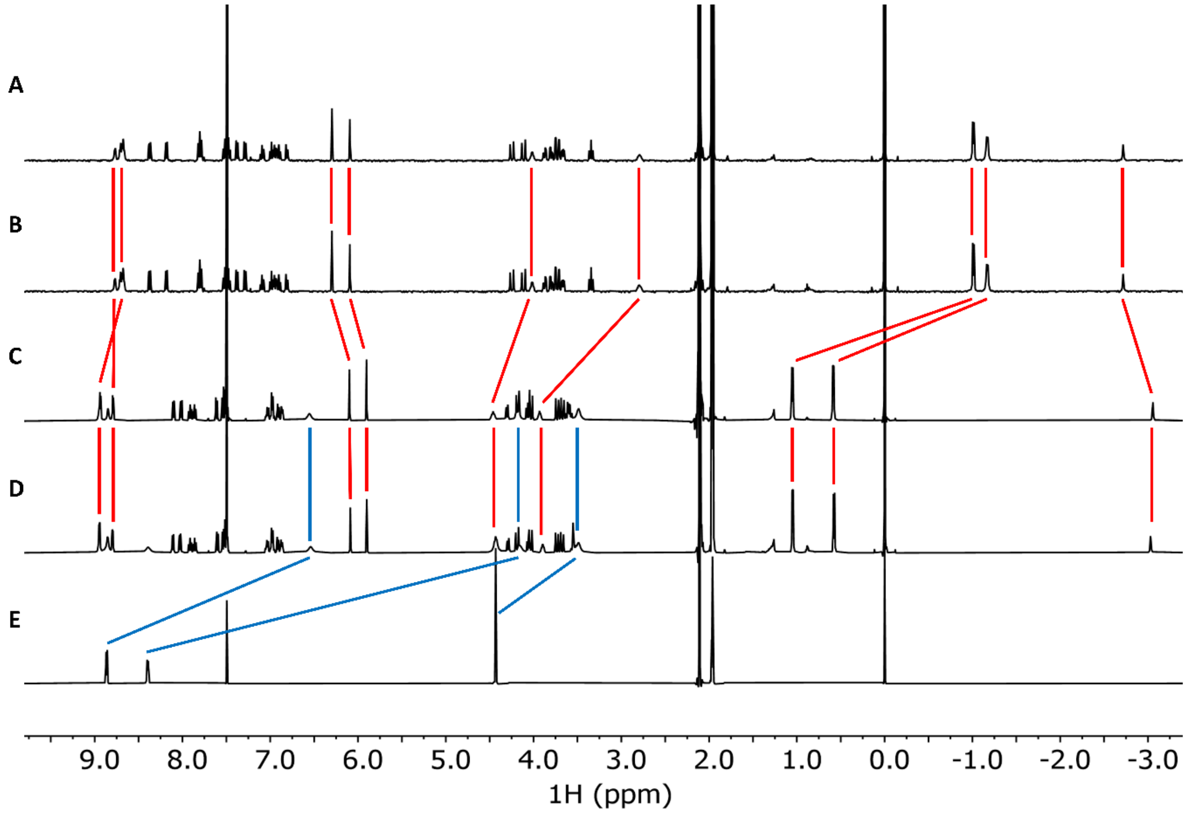

*Fig. S105. Stacked ^{1}H NMR spectra (c = 10^{-3} M in $CDCl_3/CD_3CN$, 1:1, v/v, 298 K) of (**A**) (S,S,S,S)-**$H_2$3** (400 MHz), (**B**) (R,R,R,R)-**$H_2$3** (400 MHz), (**C**) (S,S,S,S)-**$H_2$3·13** (500 MHz), (**D**) (R,R,R,R)-**$H_2$3·13** (500 MHz), and (**E**) **13** (500 MHz). Chemical shift changes of relevant host and guest protons are indicated with red and blue lines, respectively.*

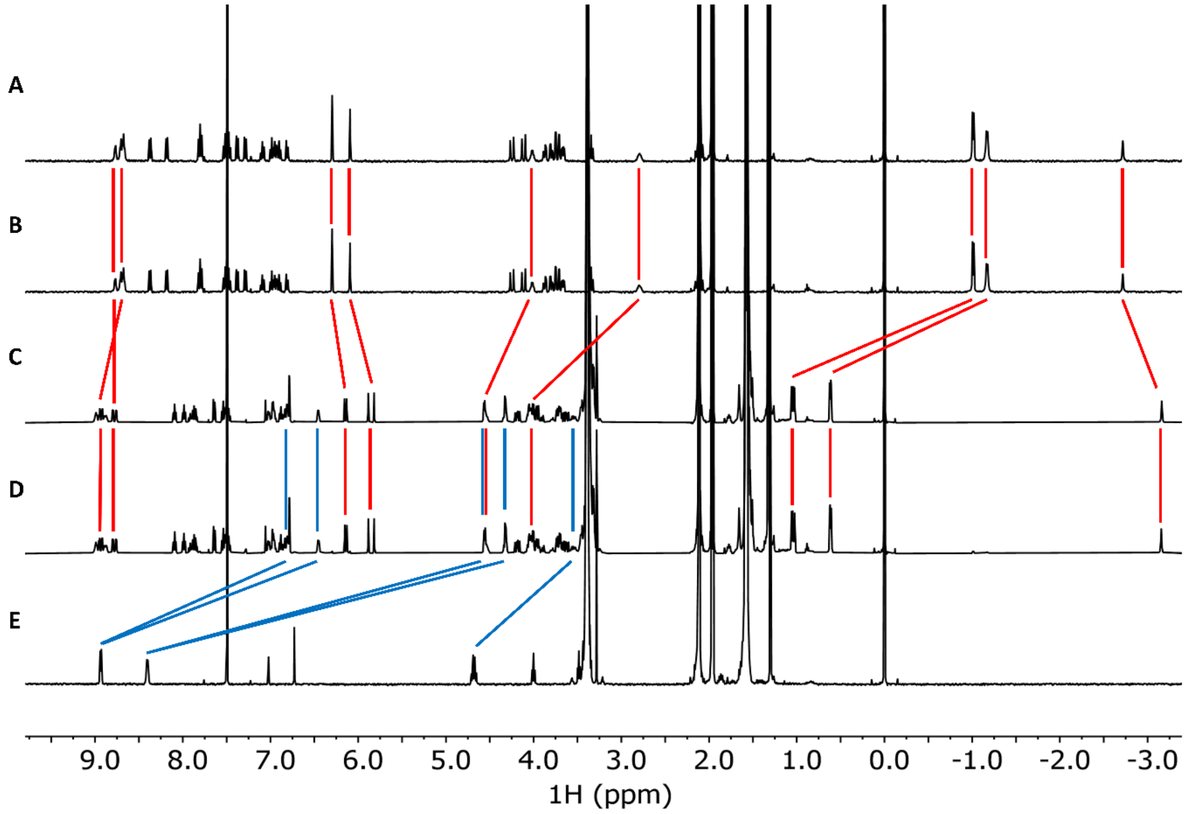

*Fig. S106. Stacked ^{1}H NMR spectra (c = 10^{-3} M in $CDCl_3/CD_3CN$, 1:1, v/v, 298 K) of (**A**) (S,S,S,S)-**$H_2$3** (400 MHz), (**B**) (R,R,R,R)-**$H_2$3** (400 MHz), (**C**) (S,S,S,S)-**$H_2$3·14** (500 MHz), (**D**) (R,R,R,R)-**$H_2$3·14** (500 MHz), and (**E**) **14** (400 MHz). Chemical shift changes of relevant host and guest protons are indicated with red and blue lines, respectively.*

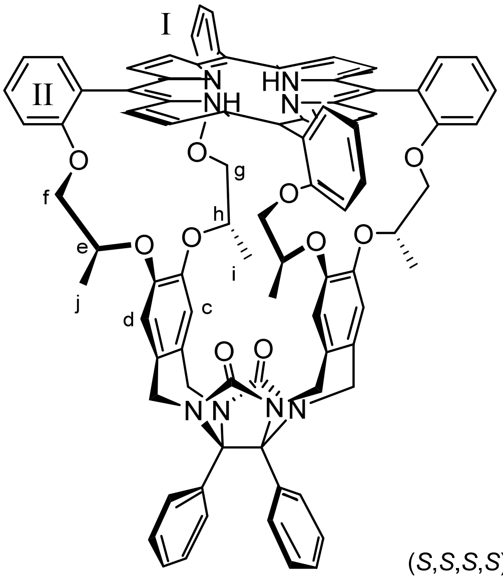

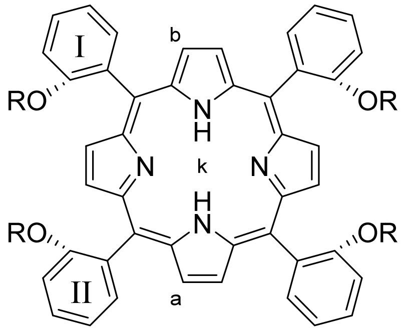

(*S*,*S*,*S*,*S*)-**$H_2$3**

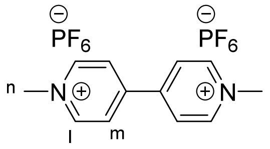

13

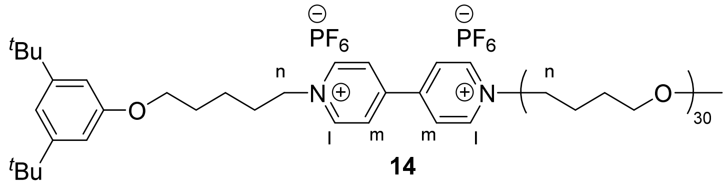

14

*Table S63. Chemical shift values and CIS-values for host **$H_2$3**, guests **13** and **14**, and the host-guest complexes **$H_2$3·13** and **$H_2$3·14** (c = 10^{-3} M in $CDCl_3/CD_3CN$, 1:1, v/v, 298 K). Relevant host and guest protons are indicated with the characters a–k and l–n, respectively.*

Proton	(*S*,*S*,*S*,*S*)-**$H_2$3** or (*R*,*R*,*R*,*R*)-**$H_2$3** δ (ppm)	**13** δ (ppm)	**14** δ (ppm)	(*S*,*S*,*S*,*S*)-**$H_2$3·13** or (*R*,*R*,*R*,*R*)-**$H_2$3·13** δ (ppm)	**CIS** Δδ (ppm)	(*S*,*S*,*S*,*S*)-**$H_2$3·14** or (*R*,*R*,*R*,*R*)-**$H_2$3·14** δ (ppm)‡	**CIS** Δδ (ppm)
a	8.77	–	–	8.79	+0.02	8.80; 8.76	+0.03; −0.01
b	8.67	–	–	8.93	+0.26	8.95; 8.92	+0.28; +0.25
c	6.30	–	–	6.10	−0.20	6.16; 6.13	−0.14; −0.17
d	6.10	–	–	5.90	−0.20	5.89; 5.82	−0.21; −0.28
e	4.01	–	–	4.47	+0.46	4.54*	+0.53
f	3.87; 3.80	–	–	4.30; 4.07	+0.43; +0.27	‖	‖
g	3.66; 3.35	–	–	3.63; 3.58	−0.03; +0.23	‖	‖
h	2.80	–	–	3.94	+1.14	4.04*	+1.24
i	−1.00	–	–	1.09	+2.09	1.05; 1.03	+2.05; +2.03
j	−1.17	–	–	0.59	+1.78	0.62; 0.61	+1.81; +1.80
k	−2.72	–	–	−3.06	−0.34	−3.16	−0.44
l	–	8.86	8.93*	6.56	−2.30	6.81; 6.46	−2.12; −2.47
m	–	8.40	8.41*	4.18	−4.22	4.57; 4.33	−3.84; −4.08
n	–	4.43	4.68*	3.49	−0.94	3.54*	−1.14

*Two overlapping signals. ‡Due to asymmetry of the viologen guest, every host signal splits up in two (non)identical signals. ‖Signals could not be assigned unambiguously.

*Chiral guests (R,R)-**15** and (S,S)-**15***

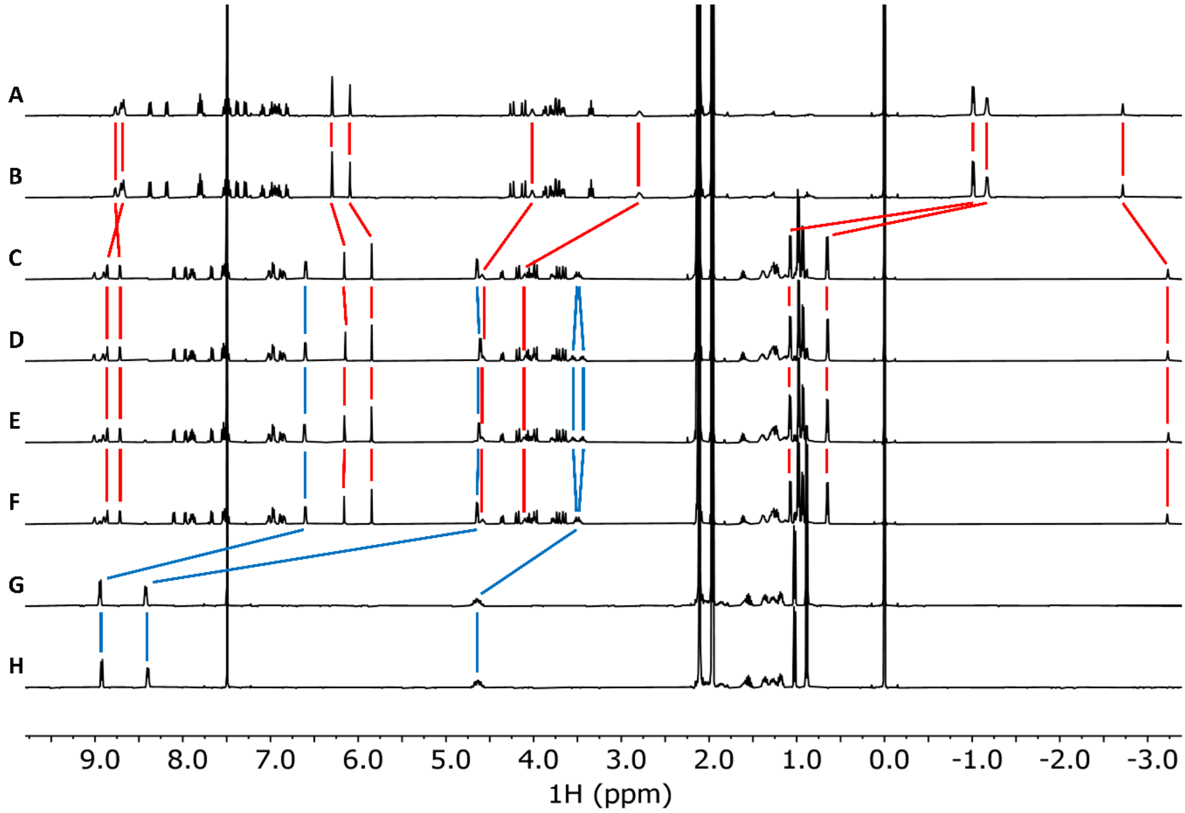

Fig. S107. Stacked ^{1}*H NMR spectra (c =* 10^{-3} *M in* $CDCl_3/CD_3CN$*, 1:1, v/v, 298 K) of (**A**) (S,S,S,S)-**H₂3** (400 MHz), (**B**) (R,R,R,R)-**H₂3** (400 MHz), (**C**) (S,S,S,S)-**H₂3**·(R,R)-**15** (500 MHz), (**D**) (R,R,R,R)-**H₂3**·(R,R)-**15** (500 MHz), (**E**) (S,S,S,S)-**H₂3**·(S,S)-**15** (500 MHz), (**F**) (R,R,R,R)-**H₂3**·(S,S)-**15** (500 MHz), (**G**) (S,S)-**15** (400 MHz), and (**H**) (R,R)-**15** (400 MHz). Chemical shift changes of relevant host and guest protons are indicated with red and blue lines, respectively.*

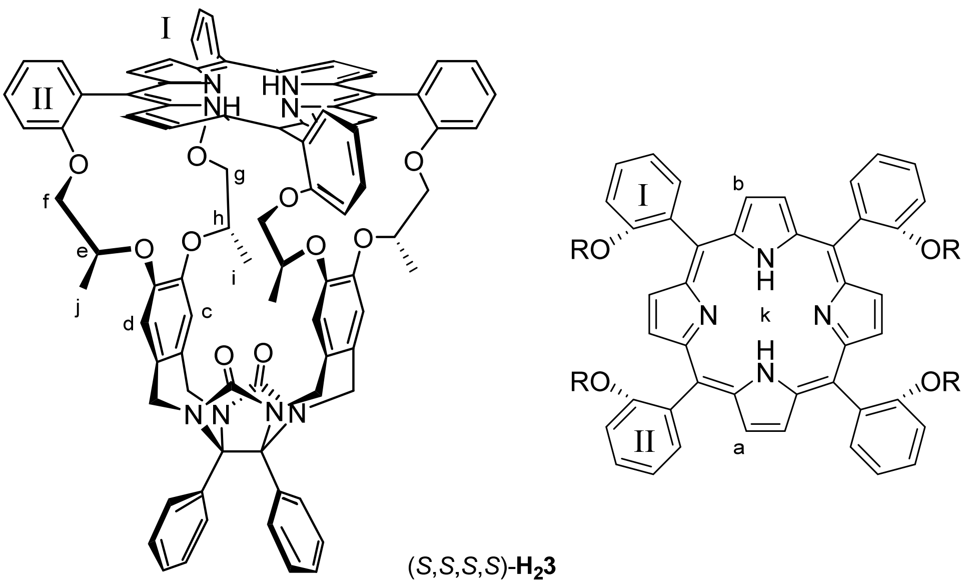

(*S*,*S*,*S*,*S*)-$\mathbf{H_23}$

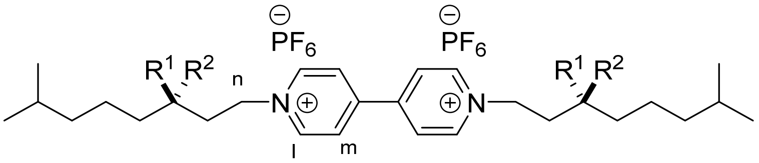

(*R*,*R*)-**15** (R^1 = Me, R^2 = H)
(*S*,*S*)-**15** (R^1 = H, R^2 = Me)

*Table S64. Chemical shift values and CIS-values for host $\mathbf{H_23}$, guest **15**, and the host-guest complexes $\mathbf{H_23{\cdot}15}$ (c = 10^{-3} M in $CDCl_3/CD_3CN$, 1:1, v/v, 298 K). Relevant host and guest protons are indicated with the characters a–k and l–n, respectively.*

**Proton**	(*S*,*S*,*S*,*S*)-$\mathbf{H_23}$ **or** (*R*,*R*,*R*,*R*)-$\mathbf{H_23}$ **δ (ppm)**	(*S*,*S*)-**15** **or** (*R*,*R*)-**15** **δ (ppm)**	(*S*,*S*,*S*,*S*)-$\mathbf{H_23}$**·**(*R*,*R*)-**15** **or** (*R*,*R*,*R*,*R*)-$\mathbf{H_23}$**·**(*S*,*S*)-**15** **δ (ppm)**	**CIS Δδ (ppm)**	(*S*,*S*,*S*,*S*)-$\mathbf{H_23}$**·**(*S*,*S*)-**15** **or** (*R*,*R*,*R*,*R*)-$\mathbf{H_23}$**·**(*R*,*R*)-**15** **δ (ppm)**	**CIS Δδ (ppm)**
a	8.77	–	8.72	−0.05	8.71	−0.06
b	8.67	–	8.86	+0.19	8.86	+0.19
c	6.30	–	6.16	−0.14	6.16	−0.14
d	6.10	–	5.85	−0.25	5.85	−0.25
e	4.01	–	4.59	+0.58	4.59	+0.58
f	3.87; 3.80	–	4.36; 4.05	+0.49; +0.25	4.36; 4.06	+0.49; +0.26
g	3.66; 3.35	–	4.01; 3.78	+0.35; +0.43	4.01; 3.78	+0.35; +0.43
h	2.80	–	4.10	+1.30	4.11	+1.31
i	−1.00	–	1.07	+2.07	1.08	+2.08
j	−1.17	–	0.65	+1.82	0.65	+1.82
k	−2.72	–	−3.24	−0.52	−3.24	−0.52
l	–	8.93	6.60	−2.33	6.61	−2.32
m	–	8.40	4.64	−3.76	4.62	−3.78
n	–	4.63*	3.50*	−1.13	3.56; 3.44	−1.07; −1.19

*Two overlapping signals of diastereotopic protons.

*Chiral guests (R,R)-**16** and (S,S)-**16***

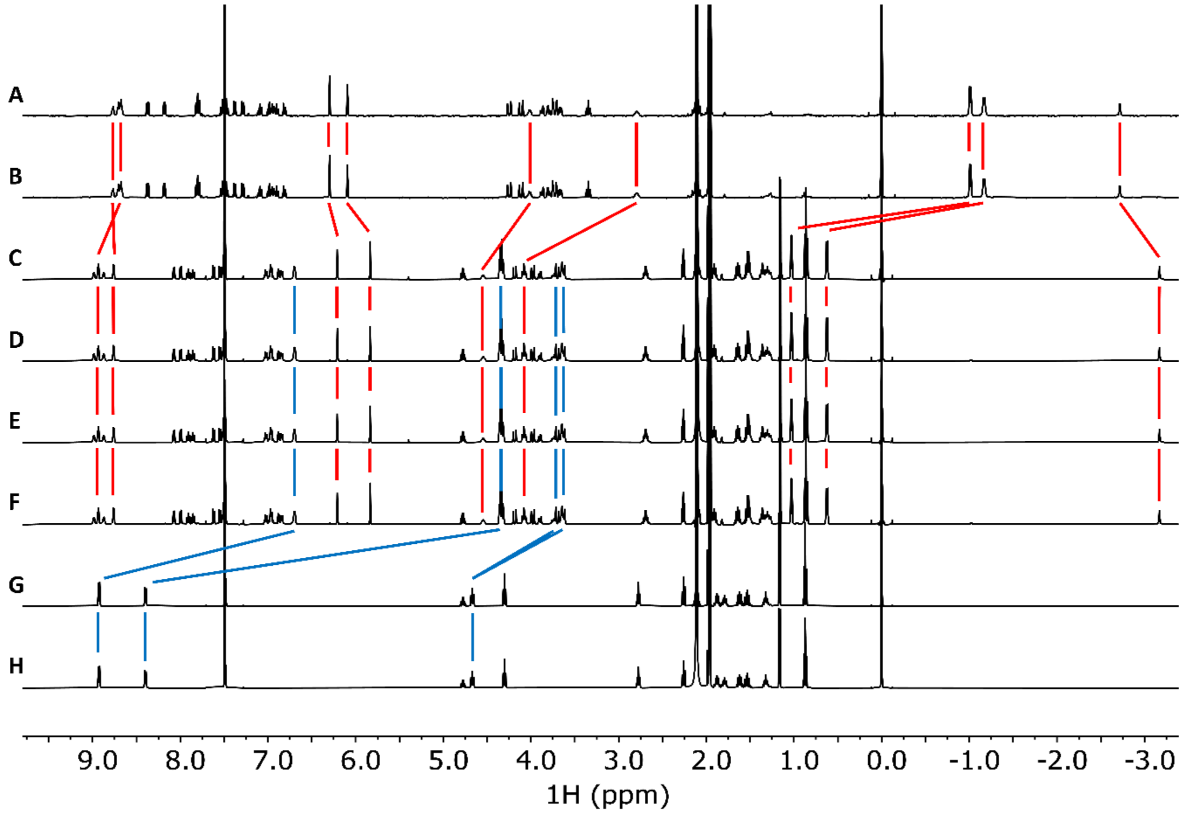

*Fig. S108. Stacked ^{1}H NMR spectra (c = 10^{-3} M in $CDCl_3/CD_3CN$, 1:1, v/v, 298 K) of (**A**) (S,S,S,S)-**$H_2$3** (400 MHz), (**B**) (R,R,R,R)-**$H_2$3** (400 MHz), (**C**) (S,S,S,S)-**$H_2$3**·(R,R)-**16** (500 MHz), (**D**) (R,R,R,R)-**$H_2$3**·(R,R)-**16** (500 MHz), (**E**) (S,S,S,S)-**$H_2$3**·(S,S)-**16** (500 MHz), (**F**) (R,R,R,R)-**$H_2$3**·(S,S)-**16** (500 MHz), (**G**) (S,S)-**16** (500 MHz), and (**H**) (R,R)-**16** (500 MHz). Chemical shift changes of relevant host and guest protons are indicated with red and blue lines, respectively.*

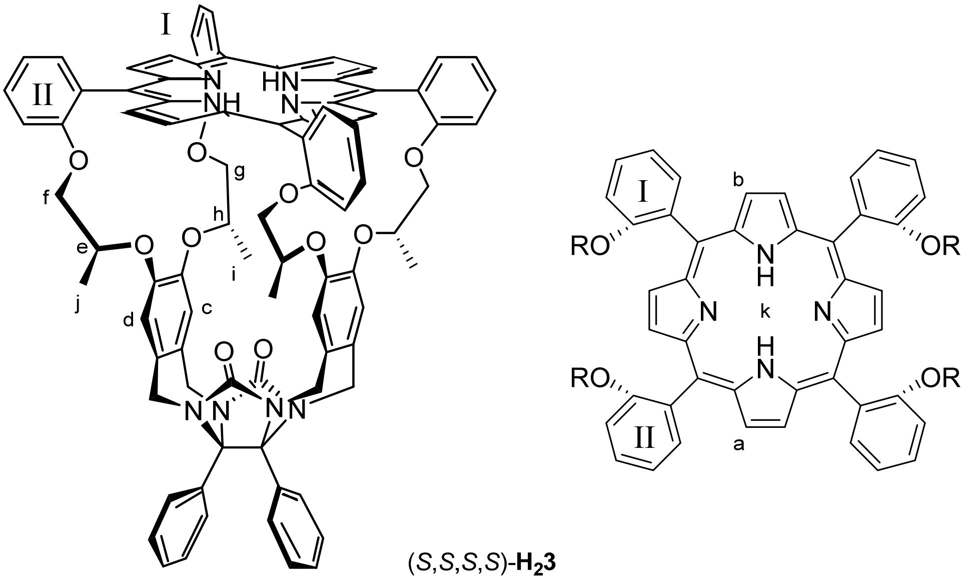

(*S*,*S*,*S*,*S*)-$\mathbf{H_23}$

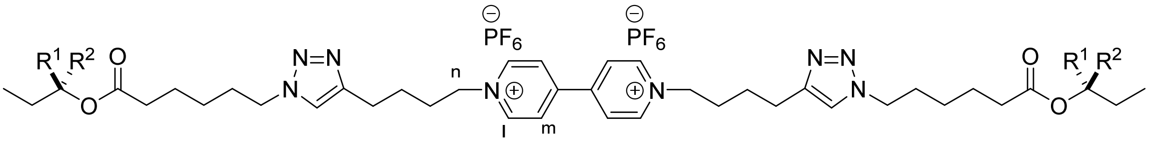

(*R*,*R*)-**16** (R^1 = Me, R^2 = H)
(*S*,*S*)-**16** (R^1 = H, R^2 = Me)

Table S65. Chemical shift values and CIS-values for host $\mathbf{H_23}$*, guest* ***16****, and the host-guest complexes* $\mathbf{H_23{\cdot}16}$ *(c = 10^{-3} M in $CDCl_3/CD_3CN$, 1:1, v/v, 298 K). Relevant host and guest protons are indicated with the characters a–k and l–n, respectively.*

**Proton**	(*S*,*S*,*S*,*S*)-$\mathbf{H_23}$ **or** (*R*,*R*,*R*,*R*)-$\mathbf{H_23}$ **δ (ppm)**	(*S*,*S*)-**16** **or** (*R*,*R*)-**16** **δ (ppm)**	(*S*,*S*,*S*,*S*)-$\mathbf{H_23}$·(*R*,*R*)-**16** **or** (*R*,*R*,*R*,*R*)-$\mathbf{H_23}$·(*S*,*S*)-**16** **δ (ppm)**	**CIS Δδ (ppm)**	(*S*,*S*,*S*,*S*)-$\mathbf{H_23}$·(*S*,*S*)-**16** **or** (*R*,*R*,*R*,*R*)-$\mathbf{H_23}$·(*R*,*R*)-**16** **δ (ppm)**	**CIS Δδ (ppm)**
a	8.77	–	8.76	−0.01	8.76	−0.01
b	8.67	–	8.94	+0.27	8.94	+0.27
c	6.30	–	6.21	−0.09	6.21	−0.09
d	6.10	–	5.83	−0.27	5.83	−0.27
e	4.01	–	4.55	+0.54	4.55	+0.54
f	3.87; 3.80	–	4.32; 4.08	+0.45; +0.28	4.32; 4.08	+0.45; +0.28
g	3.66; 3.35	–	3.90; 3.64	+0.24; +0.31	3.90; 3.64	+0.24; +0.31
h	2.80	–	4.08	+1.28	4.08	+1.28
i	−1.00	–	1.03	+2.03	1.03	+2.03
j	−1.17	–	0.62	+1.79	0.62	+1.79
k	−2.72	–	−3.17	−0.45	−3.17	−0.45
l	–	8.93	6.70	−2.23	6.70	−2.23
m	–	8.40	4.35	−4.05	4.35	−4.05
n	–	4.67	3.75; 3.64	−0.92; −1.03	3.75; 3.64	−0.92; −1.03

*Chiral guests (R,R)-**17** and (S,S)-**17***

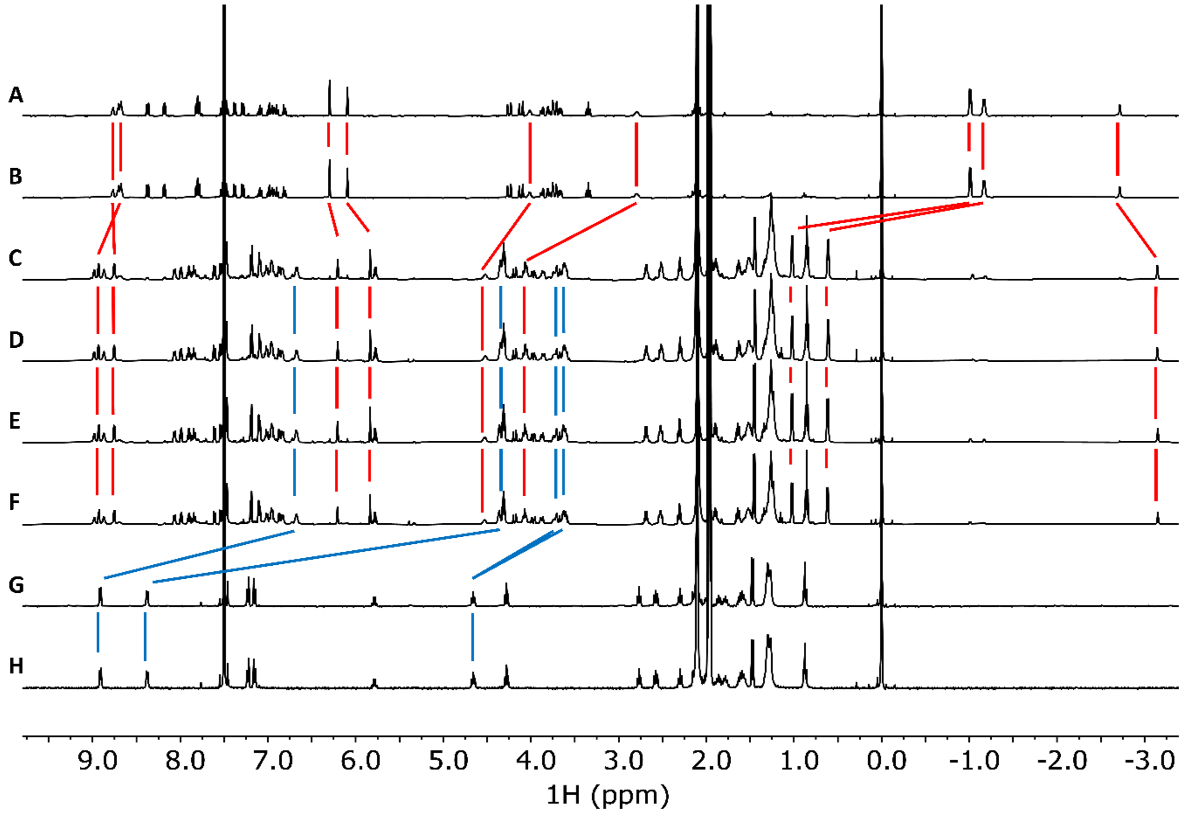

*Fig. S109. Stacked ^{1}H NMR spectra (c = 10^{-3} M in $CDCl_3/CD_3CN$, 1:1, v/v, 298 K) of (**A**) (S,S,S,S)-**$H_2$3** (400 MHz), (**B**) (R,R,R,R)-**$H_2$3** (400 MHz), (**C**) (S,S,S,S)-**$H_2$3**·(R,R)-**17** (500 MHz), (**D**) (R,R,R,R)-**$H_2$3**·(R,R)-**17** (500 MHz), (**E**) (S,S,S,S)-**$H_2$3**·(S,S)-**17** (500 MHz), (**F**) (R,R,R,R)-**$H_2$3**·(S,S)-**17** (500 MHz), (**G**) (S,S)-**17** (400 MHz), and (**H**) (R,R)-**17** (400 MHz). Chemical shift changes of relevant host and guest protons are indicated with red and blue lines, respectively.*

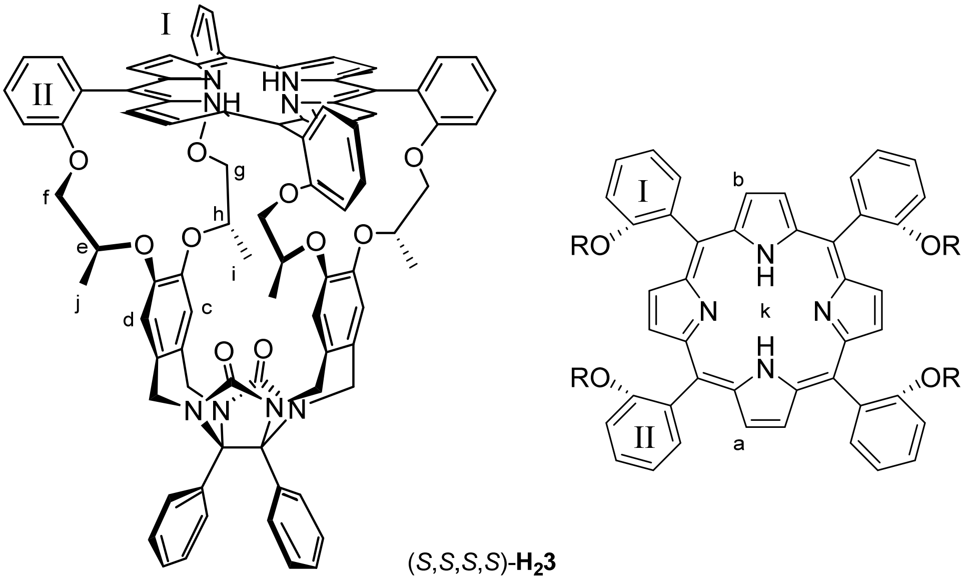

(*S*,*S*,*S*,*S*)-$\mathbf{H_23}$

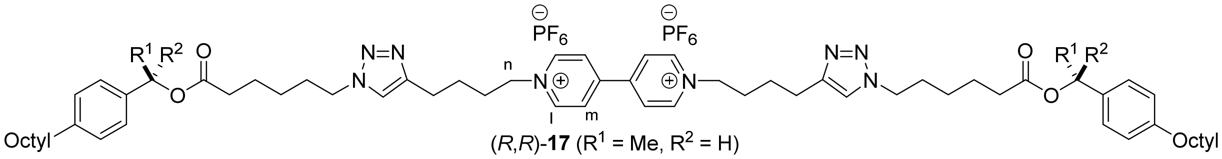

(*R*,*R*)-**17** (R^1 = Me, R^2 = H)
(*S*,*S*)-**17** (R^1 = H, R^2 = Me)

Table S66. Chemical shift values and CIS-values for host $\mathbf{H_23}$*, guest* **17***, and the host-guest complexes* $\mathbf{H_23{\cdot}17}$ *(c = 10^{-3} M in $CDCl_3/CD_3CN$, 1:1, v/v, 298 K). Relevant host and guest protons are indicated with the characters a–k and l–n, respectively.*

Proton	(*S*,*S*,*S*,*S*)-$\mathbf{H_23}$ or (*R*,*R*,*R*,*R*)-$\mathbf{H_23}$ δ (ppm)	(*S*,*S*)-**17** or (*R*,*R*)-**17** δ (ppm)	(*S*,*S*,*S*,*S*)-$\mathbf{H_23}$·(*R*,*R*)-**17** or (*R*,*R*,*R*,*R*)-$\mathbf{H_23}$·(*S*,*S*)-**17** δ (ppm)	CIS Δδ (ppm)	(*S*,*S*,*S*,*S*)-$\mathbf{H_23}$·(*S*,*S*)-**17** or (*R*,*R*,*R*,*R*)-$\mathbf{H_23}$·(*R*,*R*)-**17** δ (ppm)	CIS Δδ (ppm)
a	8.77	–	8.75	−0.02	8.75	−0.02
b	8.67	–	8.93	+0.26	8.93	+0.26
c	6.30	–	6.21	−0.09	6.21	−0.09
d	6.10	–	5.84	−0.26	5.84	−0.26
e	4.01	–	4.53	+0.52	4.53	+0.52
f	3.87; 3.80	–	4.32; 4.07	+0.45; +0.27	4.32; 4.07	+0.45; +0.27
g	3.66; 3.35	–	3.88; 3.62	+0.22; +0.27	3.88; 3.62	+0.22; +0.27
h	2.80	–	4.07	+1.27	4.07	+1.27
i	−1.00	–	1.02	+2.02	1.02	+2.02
j	−1.17	–	0.62	+1.79	0.62	+1.79
k	−2.72	–	−3.15	−0.43	−3.15	−0.43
l	–	8.91	6.68	−2.23	6.68	−2.23
m	–	8.38	4.36	−4.02	4.36	−4.02
n	–	4.66	3.73; 3.61	−0.93; −1.05	3.73; 3.62	−0.93; −1.04

*Chiral guests (R,R)-**18** and (S,S)-**18***

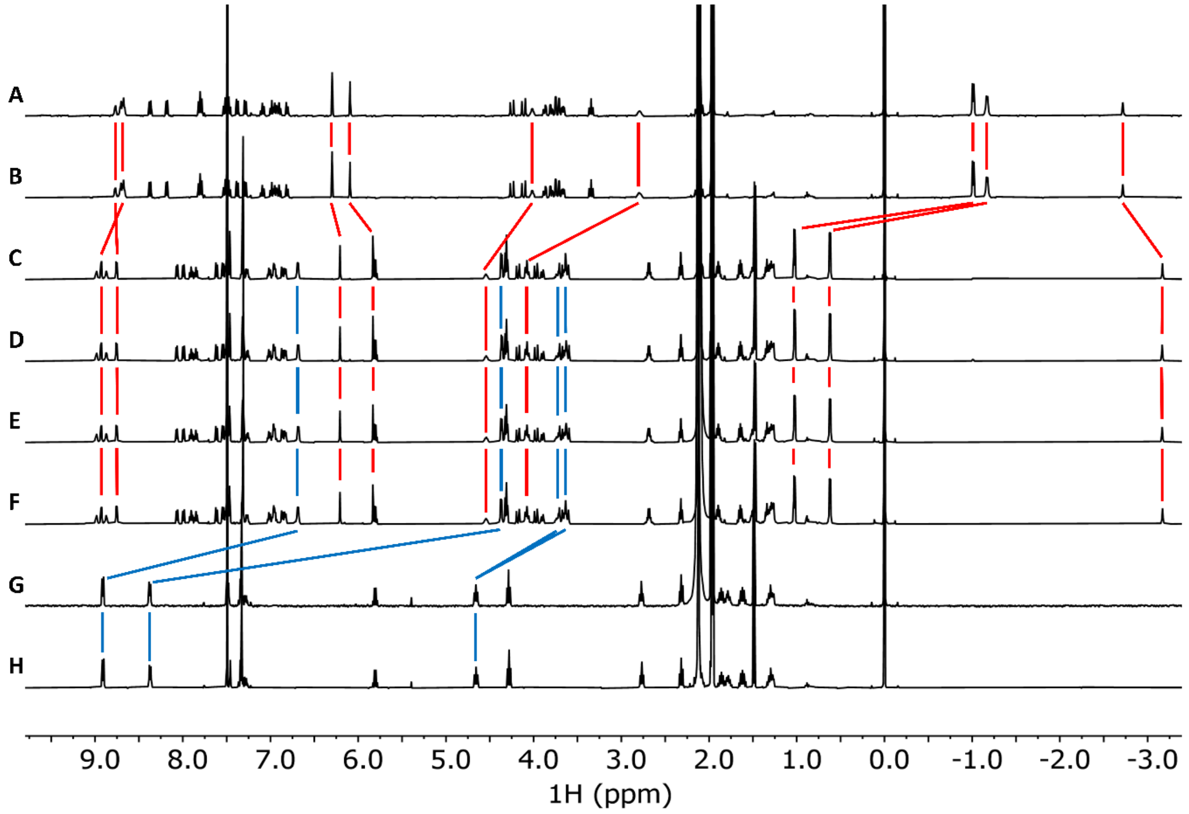

*Fig. S110. Stacked ^{1}H NMR spectra (c = 10^{-3} M in $CDCl_3$/CD_3CN, 1:1, v/v, 298 K) of (**A**) (S,S,S,S)-**$H_2$3** (400 MHz), (**B**) (R,R,R,R)-**$H_2$3** (400 MHz), (**C**) (S,S,S,S)-**$H_2$3**·(R,R)-**18** (500 MHz), (**D**) (R,R,R,R)-**$H_2$3**·(R,R)-**18** (500 MHz), (**E**) (S,S,S,S)-**$H_2$3**·(S,S)-**18** (500 MHz), (**F**) (R,R,R,R)-**$H_2$3**·(S,S)-**18** (500 MHz), (**G**) (S,S)-**18** (400 MHz), and (**H**) (R,R)-**18** (400 MHz). Chemical shift changes of relevant host and guest protons are indicated with red and blue lines, respectively.*

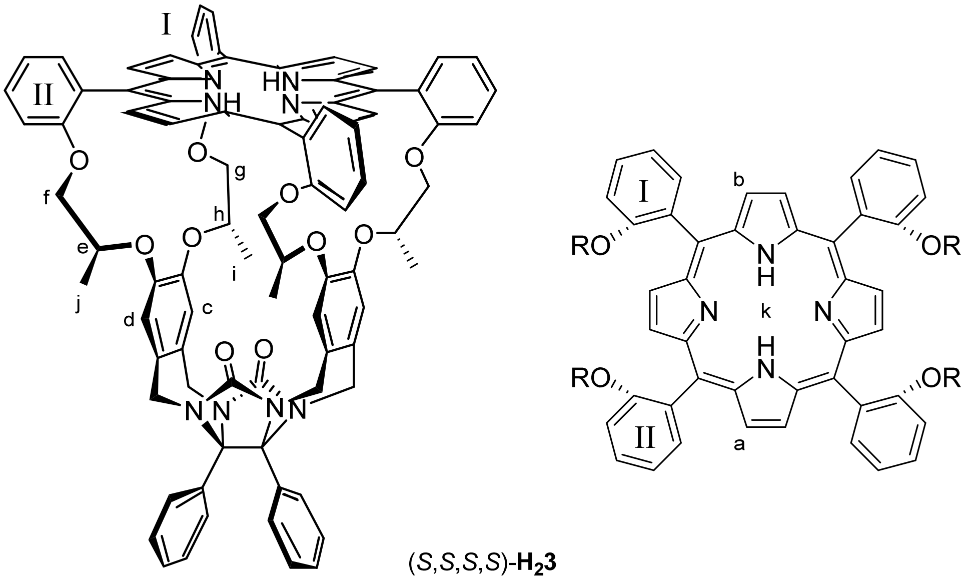

(S,S,S,S)-**H$_2$3**

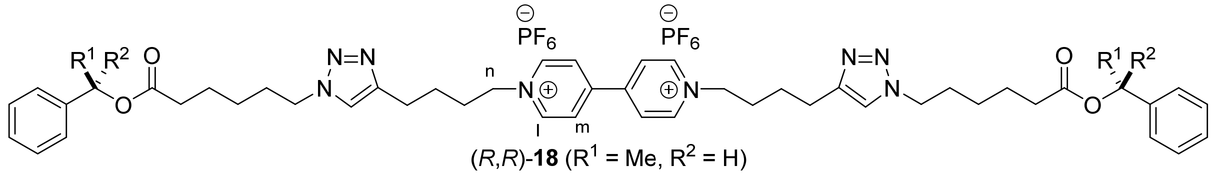

(R,R)-**18** (R^1 = Me, R^2 = H)
(S,S)-**18** (R^1 = H, R^2 = Me)

*Table S67. Chemical shift values and CIS-values for host **H$_2$3**, guest **18**, and the host-guest complexes **H$_2$3·18** (c = 10^{-3} M in CDCl$_3$/CD$_3$CN, 1:1, v/v, 298 K). Relevant host and guest protons are indicated with the characters a–k and l–n, respectively.*

**Proton**	(S,S,S,S)-**H$_2$3** **or** (R,R,R,R)-**H$_2$3** **δ (ppm)**	(S,S)-**18** **or** (R,R)-**18** **δ (ppm)**	(S,S,S,S)-**H$_2$3**·(R,R)-**18** **or** (R,R,R,R)-**H$_2$3**·(S,S)-**18** **δ (ppm)**	**CIS** **Δδ (ppm)**	(S,S,S,S)-**H$_2$3**·(S,S)-**18** **or** (R,R,R,R)-**H$_2$3**·(R,R)-**18** **δ (ppm)**	**CIS** **Δδ (ppm)**
a	8.77	–	8.75	−0.02	8.75	−0.02
b	8.67	–	8.93	+0.26	8.93	+0.26
c	6.30	–	6.21	−0.09	6.21	−0.09
d	6.10	–	5.83	−0.27	5.83	−0.27
e	4.01	–	4.55	+0.54	4.55	+0.54
f	3.87; 3.80	–	4.32; 4.08	+0.45; +0.28	4.31; 4.08	+0.44; +0.28
g	3.66; 3.35	–	3.91; 3.64	+0.25; +0.29	3.91; 3.63	+0.25; +0.28
h	2.80	–	4.09	+1.29	4.08	+1.28
i	−1.00	–	1.03	+2.03	1.03	+2.03
j	−1.17	–	0.62	+1.79	0.62	+1.79
k	−2.72	–	−3.18	−0.46	−3.17	−0.45
l	–	8.91	6.69	−2.22	6.69	−2.22
m	–	8.37	4.37	−4.00	4.37	−4.00
n	–	4.66	3.73; 3.63	−0.93; −1.03	3.74; 3.64	−0.92; −1.02

3. DFT calculations

DFT calculations were performed at the B3LYP/6-311+G(d) level. Fig. S46–S47 display the DFT-optimized structure of (*R*,*R*,*R*,*R*)-**$H_2$3**. The front view (Fig. S111) demonstrates that the methyl groups attached to the oxyethylene spacers fill up the cavity. As can be seen from the side view (Fig. S112), the chirality in the chiral spacers is effectively transferred throughout the entire molecule, giving it a twisted geometry. The Cartesian coordinates of DFT-optimized (*R*,*R*,*R*,*R*)-**$H_2$3** are provided in Table S68.

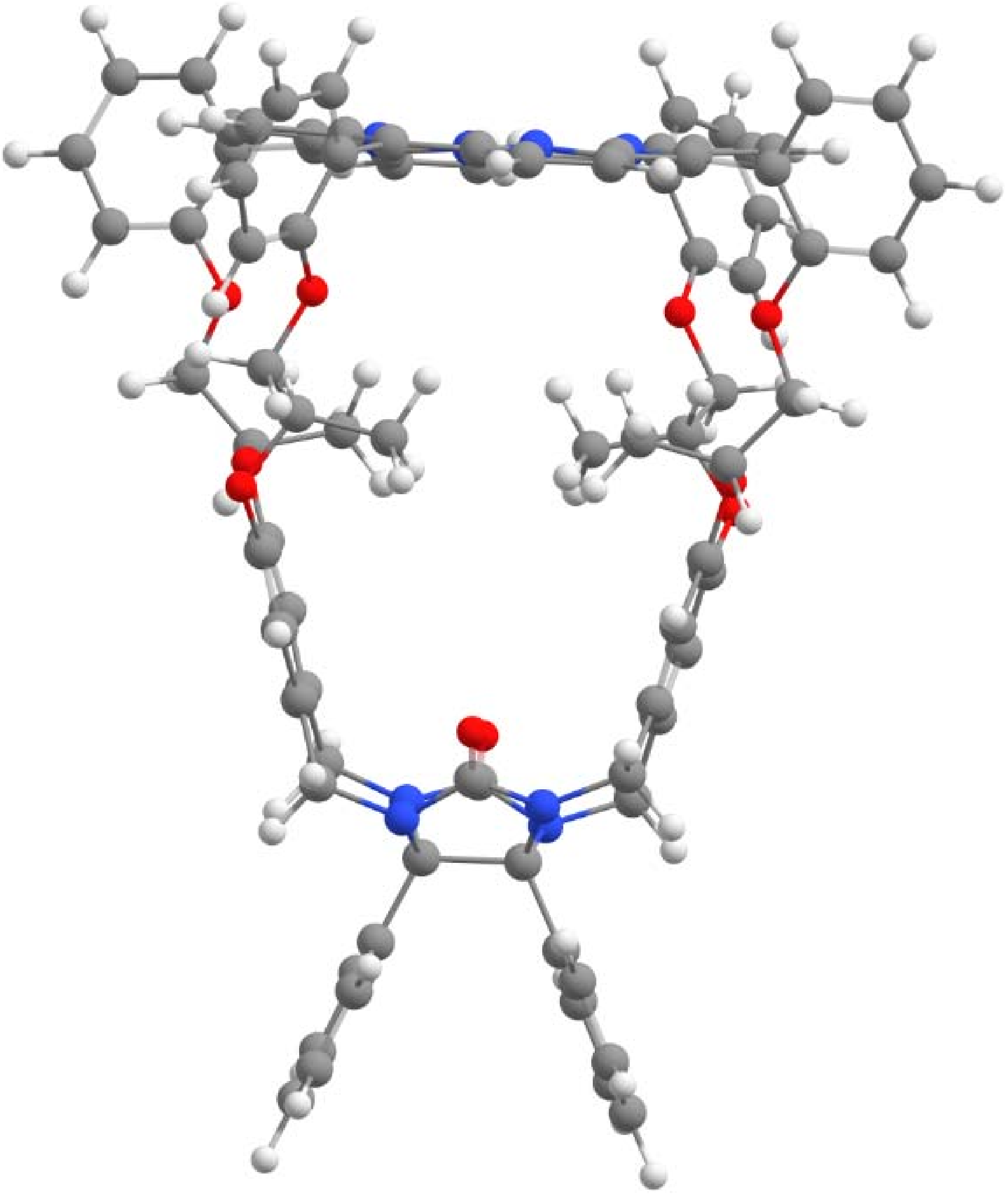

*Fig. S111. Front view of DFT-optimized (R,R,R,R)-**$H_2$3**. Color code: white = hydrogen, grey = carbon, blue = nitrogen, red = oxygen.*

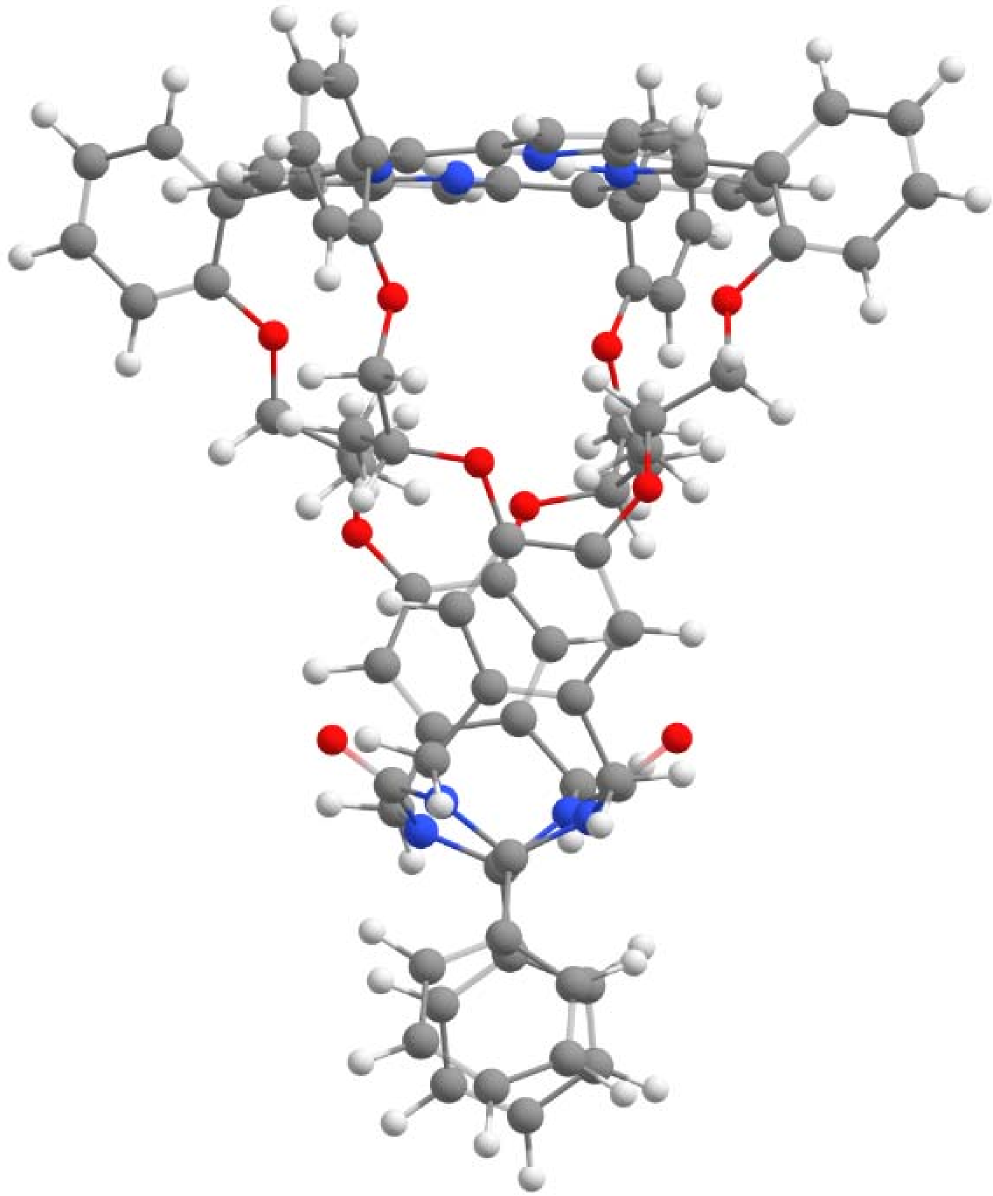

*Fig. S112. Side view of DFT-optimized (R,R,R,R)-**H₂3**. Color code: white = hydrogen, grey = carbon, blue = nitrogen, red = oxygen.*

*Table S68. Cartesian coordinates of the optimized structure of (R,R,R,R)-**H₂3** at the B3LYP/6-311+G(d) level.*

Atom	Atomic number	Cartesian coordinates (Å)		
		X	Y	Z
1	8	0.42053	-3.80115	-0.89656
2	8	-2.222393	-4.18727	-2.33189
3	8	4.572874	-0.20811	2.794237
4	8	0.838128	-3.99888	1.831893
5	8	4.572566	0.208102	-2.7941
6	7	5.693557	1.026521	1.188458
7	7	-4.765301	-1.98385	0.411273
8	7	-4.764933	1.98408	-0.41158
9	7	5.970354	1.193885	-1.22216
10	7	-4.728698	-0.42755	-2.06924
11	1	-4.789928	-0.22117	-1.07967
12	7	5.693291	-1.02666	-1.18845
13	8	0.42097	3.80129	0.897048

14	8	-2.288338	3.066939	-3.29461
15	7	-4.72895	0.427769	2.06897
16	1	-4.790189	0.221387	1.0794
17	7	5.97032	-1.19406	1.222122
18	8	-2.222024	4.186983	2.332476
19	8	0.838311	3.999063	-1.83134
20	8	-2.288636	-3.06652	3.294894
21	6	-4.633776	-1.69329	-2.59801
22	6	-4.687191	1.910677	-2.88222
23	6	-3.317723	-4.80541	-2.86834
24	6	-4.463038	2.764564	-4.09288
25	6	-5.443287	2.997667	-5.0543
26	1	-6.417072	2.53463	-4.92949
27	6	7.942162	-1.45857	-0.25199
28	6	-4.769618	3.012961	0.485596
29	6	6.574654	0.804478	0.045795
30	6	-4.662923	-2.89658	-1.88321
31	6	-4.64565	0.522616	-3.05988
32	6	6.574548	-0.80473	-0.04588
33	6	1.655249	-3.47241	-0.40792
34	6	5.128575	2.328931	1.520007
35	1	5.937388	3.067584	1.48866
36	1	4.789921	2.265411	2.554326
37	6	-4.560263	-4.16452	-2.67811
38	6	-4.493627	-1.53129	-4.01447
39	1	-4.403919	-2.34586	-4.71653
40	6	5.314531	-0.12965	1.831697
41	6	2.739837	-3.04939	-1.18455
42	1	2.628636	-2.92183	-2.25351
43	6	-4.645943	-0.5224	3.059602
44	6	3.984987	2.762222	0.619019
45	6	-4.633795	1.6935	2.597735
46	6	-4.414822	-6.57177	-4.11128
47	1	-4.347249	-7.50798	-4.65638
48	6	1.831589	-3.57479	0.986182
49	6	4.159732	-2.88117	0.765987
50	6	-5.198493	3.826432	-6.15236
51	1	-5.977768	4.001705	-6.88623
52	6	-4.560036	4.164756	2.67784
53	6	5.482865	2.559733	-1.43767
54	1	5.388448	2.698843	-2.51466
55	1	6.271103	3.236958	-1.09581
56	6	3.984626	-2.76224	-0.61883
57	6	-4.794412	2.570078	-1.6423
58	6	2.740272	3.049456	1.184877
59	1	2.629201	2.921887	2.253832
60	6	-4.769912	-3.01273	-0.4859
61	6	4.159955	2.881148	-0.76582
62	6	-4.79486	-2.56984	1.641992
63	6	7.942371	1.458141	0.251777
64	6	-4.463636	-2.76442	4.092568
65	6	-5.642637	-5.94271	-3.94577
66	1	-6.546213	-6.37555	-4.36121
67	6	5.314339	0.12956	-1.83166
68	6	-4.662751	2.896799	1.882935
69	6	3.071984	-3.28034	1.540662
70	6	-3.25458	-6.01061	-3.57679
71	1	-2.311854	-6.52581	-3.70895
72	6	-4.875078	-4.02142	1.522696
73	1	-4.940383	-4.72039	2.343248

74	6	-3.953842	4.431805	-6.28787
75	1	-3.753025	5.083	-7.13279
76	6	-4.494989	-0.19077	-4.29376
77	1	-4.401992	0.276006	-5.26196
78	6	5.482721	-2.55985	1.437691
79	1	5.388388	-2.69893	2.514693
80	1	6.270875	-3.23714	1.095777
81	6	-5.701724	-4.7475	-3.2282
82	1	-6.655287	-4.2506	-3.08084
83	6	-4.855398	-4.29735	0.199668
84	1	-4.899443	-5.26613	-0.27522
85	6	-5.701565	4.748035	3.22747
86	1	-6.65519	4.251352	3.079769
87	6	-4.855001	4.297586	-0.19997
88	1	-4.89906	5.266368	0.274918
89	6	-3.317409	4.805391	2.868502
90	6	-5.642477	5.943251	3.945028
91	1	-6.546114	6.376314	4.360101
92	6	-2.948939	4.211038	-5.34357
93	1	-1.981757	4.684585	-5.46336
94	6	5.128161	-2.32903	-1.51994
95	1	5.936915	-3.06776	-1.48867
96	1	4.78941	-2.26549	-2.55423
97	6	-4.874541	4.021659	-1.52301
98	1	-4.939693	4.720642	-2.34357
99	6	3.072157	3.280419	-1.54039
100	6	-3.199186	3.374761	-4.2545
101	6	-0.932486	-4.67223	-2.68657
102	1	-0.884976	-4.81668	-3.77271
103	1	-0.735383	-5.63227	-2.1973
104	6	0.158764	-3.6768	-2.30804
105	1	1.04664	-4.02163	-2.85078
106	6	8.437552	-1.67788	-1.54118
107	1	7.836541	-1.40584	-2.40146
108	6	8.735616	-1.81933	0.841554
109	1	8.36497	-1.65447	1.846325
110	6	-4.687601	-1.91047	2.881915
111	6	1.655653	3.472526	0.408351
112	6	-4.493691	1.53148	4.014195
113	1	-4.403931	2.346046	4.716258
114	6	-5.444104	-2.99765	5.053741
115	1	-6.417847	-2.53457	4.928788
116	6	-5.199589	-3.82663	6.1517
117	1	-5.979026	-4.00201	6.885373
118	6	1.83184	3.574929	-0.98576
119	6	-0.349323	-3.17796	1.968674
120	1	-0.990183	-3.34082	1.099178
121	6	-3.955007	-4.43211	6.287337
122	1	-3.754423	-5.08351	7.132164
123	6	-4.414585	6.572031	4.110979
124	1	-4.347	7.508242	4.656086
125	6	-3.254268	6.01059	3.57695
126	1	-2.311486	6.525591	3.70946
127	6	9.992882	-2.38809	0.650986
128	1	10.59369	-2.663	1.511984
129	6	0.159155	3.676664	2.308486
130	1	1.047004	4.021382	2.851335
131	6	8.735745	1.818874	-0.84184
132	1	8.364968	1.654119	-1.84658
133	6	-4.495211	0.190955	4.293487

134	1	-4.402306	-0.27584	5.261684
135	6	8.437936	1.677321	1.54092
136	1	7.836987	1.405306	2.401256
137	6	9.695302	-2.24627	-1.73336
138	1	10.06179	-2.41065	-2.74163
139	6	9.993104	2.387464	-0.65138
140	1	10.59384	2.662343	-1.51243
141	6	-2.949883	-4.21121	5.343318
142	1	-1.982764	-4.68485	5.46323
143	6	-1.042991	-3.74583	3.205766
144	1	-1.197649	-4.82409	3.092845
145	1	-0.433001	-3.56539	4.099197
146	6	-3.199815	-3.37465	4.254382
147	6	9.695778	2.245538	1.732986
148	1	10.0624	2.409812	2.741229
149	6	10.47736	-2.60466	-0.63758
150	1	11.45554	-3.05053	-0.78628
151	6	10.47775	2.603892	0.637146
152	1	11.456	3.049638	0.785759
153	6	-0.932139	4.672071	2.687043
154	1	-0.884515	4.816676	3.773159
155	1	-0.735156	5.632063	2.197612
156	6	-1.042282	3.745525	-3.20583
157	1	-1.196239	4.824021	-3.09414
158	1	-0.432106	3.563716	-4.09886
159	6	-0.349492	3.17858	-1.96781
160	1	3.16469	3.353531	-2.61876
161	1	3.164607	-3.35342	2.619017
162	1	-0.990679	3.34272	-1.0988
163	6	-0.033324	1.697925	-2.11663
164	1	0.429666	1.294363	-1.21452
165	1	0.645145	1.521059	-2.95658
166	1	-0.960045	1.150237	-2.29342
167	6	-0.174117	2.238328	2.687117
168	1	0.646663	1.564184	2.438771
169	1	-1.070845	1.907825	2.16369
170	1	-0.360655	2.16022	3.76199
171	6	-0.174453	-2.23854	-2.68697
172	1	0.646194	-1.56433	-2.43838
173	1	-1.071399	-1.90802	-2.16394
174	1	-0.36058	-2.16055	-3.76192
175	6	-0.032401	-1.69764	2.119313
176	1	-0.958912	-1.1496	2.296272
177	1	0.431316	-1.29331	1.21794
178	1	0.64571	-1.52211	2.959837

4. References

[S1] P. J. Gilissen, A. Swartjes, B. Spierenburg, J. P. J. Bruekers, P. Tinnemans, P. B. White, F. P. J. T. Rutjes, R. J. M. Nolte and J. A. A. W. Elemans, *Tetrahedron*, 2019, **75**, 4640–4647.

[S2] J. Luo, B. Hu, A. Sam and T. L. Liu, *Org. Lett.*, 2018, **20**, 361–364.

[S3] R. G. E. Coumans, J. A. A. W. Elemans, R. J. M. Nolte and A. E. Rowan, *Proc. Natl. Acad. Sci. USA*, 2006, **103**, 19647–19651.

[S4] S. Varghese, B. Spierenburg, J. P. J. Bruekers, A. Swartjes, P. B. White, J. A. A. W. Elemans and R. J. M. Nolte, *Eur. J. Org. Chem.*, 2019, 3525–3533.

[S5] M. T. Nguyen, D. P. Ferris, C. Pezzato, Y. Wang and J. F. Stoddart, *Chem*, 2018, **4**, 2329–2344.

[S6] Online tools for supramolecular chemistry research and analysis. http://supramolecular.org.

[S7] D. B. Hibbert and P. Thordarson, *Chem. Commun.*, 2016, **52**, 12792–12805.

5. Copies of NMR and optical spectra of new compounds

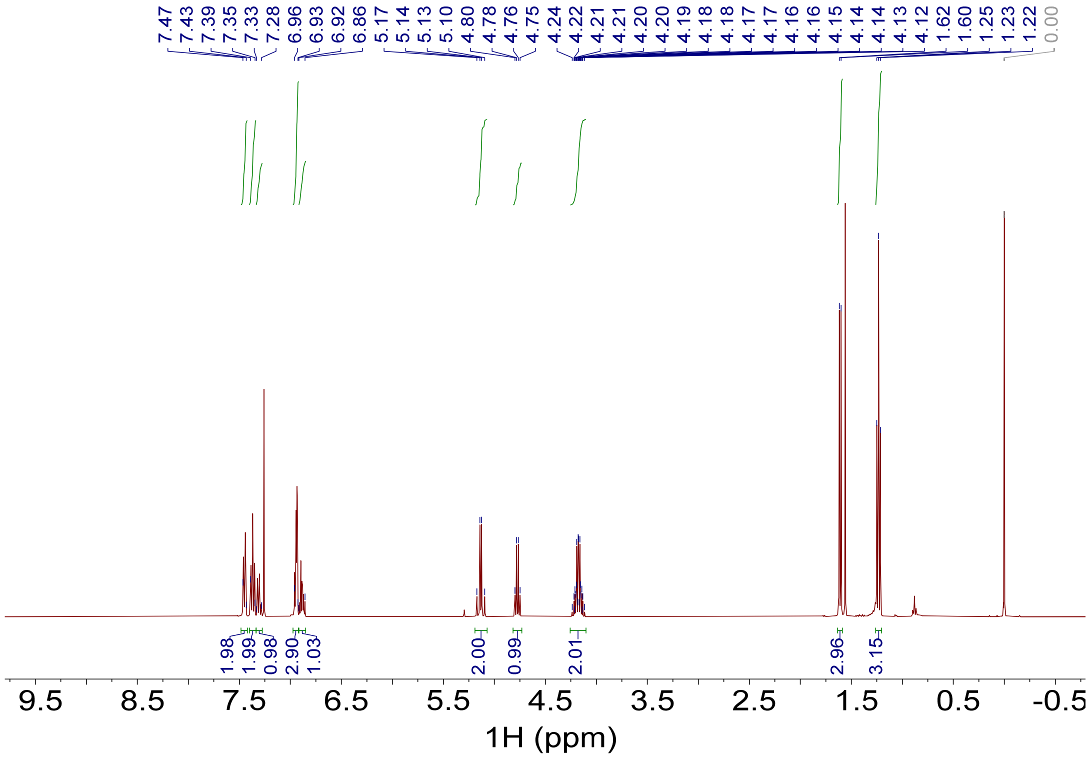

Fig. S113. *^{1}H NMR spectrum of (R)-**5** (400 MHz, $CDCl_3$, 298 K).*

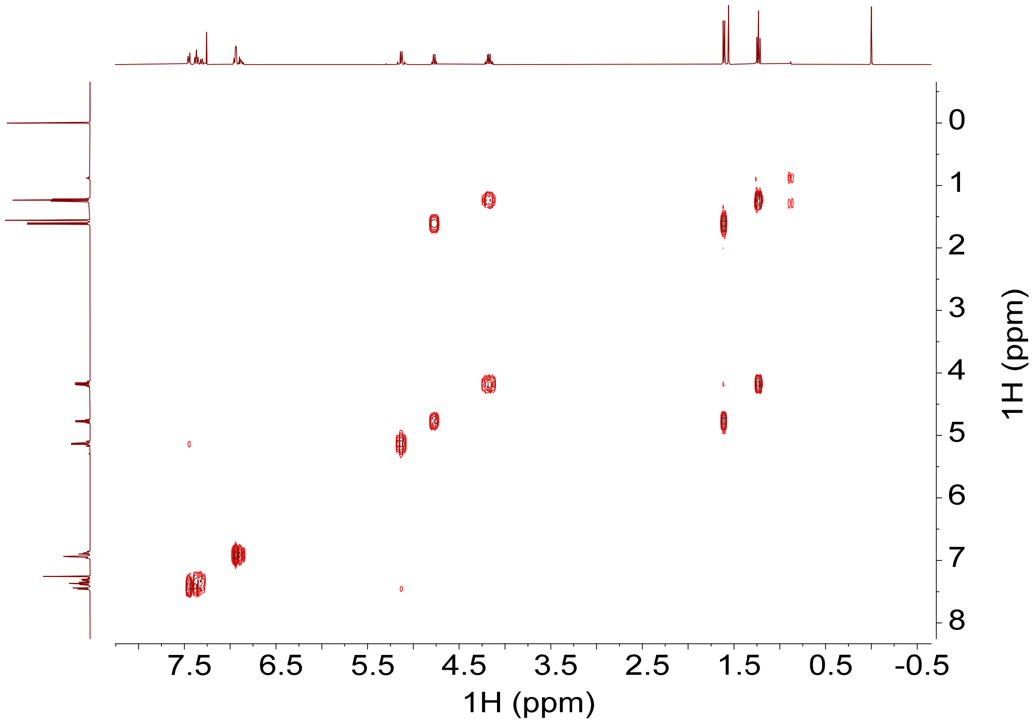

Fig. S114. *^{1}H-^{1}H COSY NMR spectrum of (R)-**5** (400 MHz, $CDCl_3$, 298 K).*

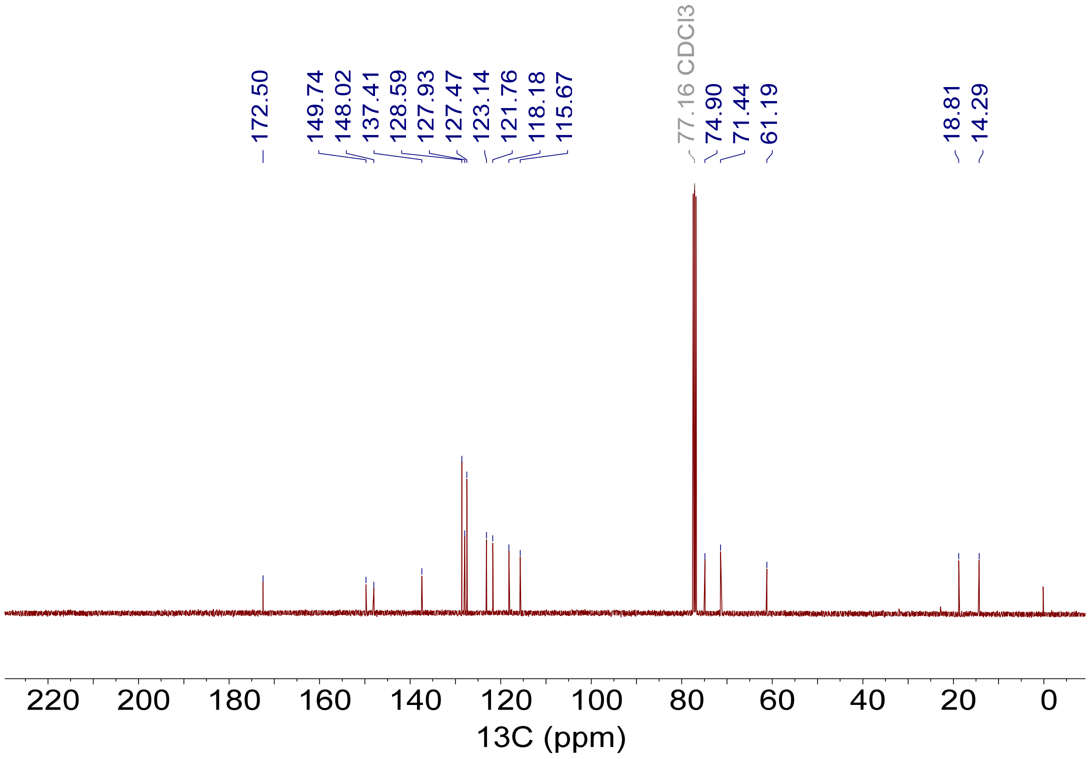

*Fig. S115. ^{13}C NMR spectrum of (R)-**5** (CPD, 101 MHz, $CDCl_3$, 298 K).*

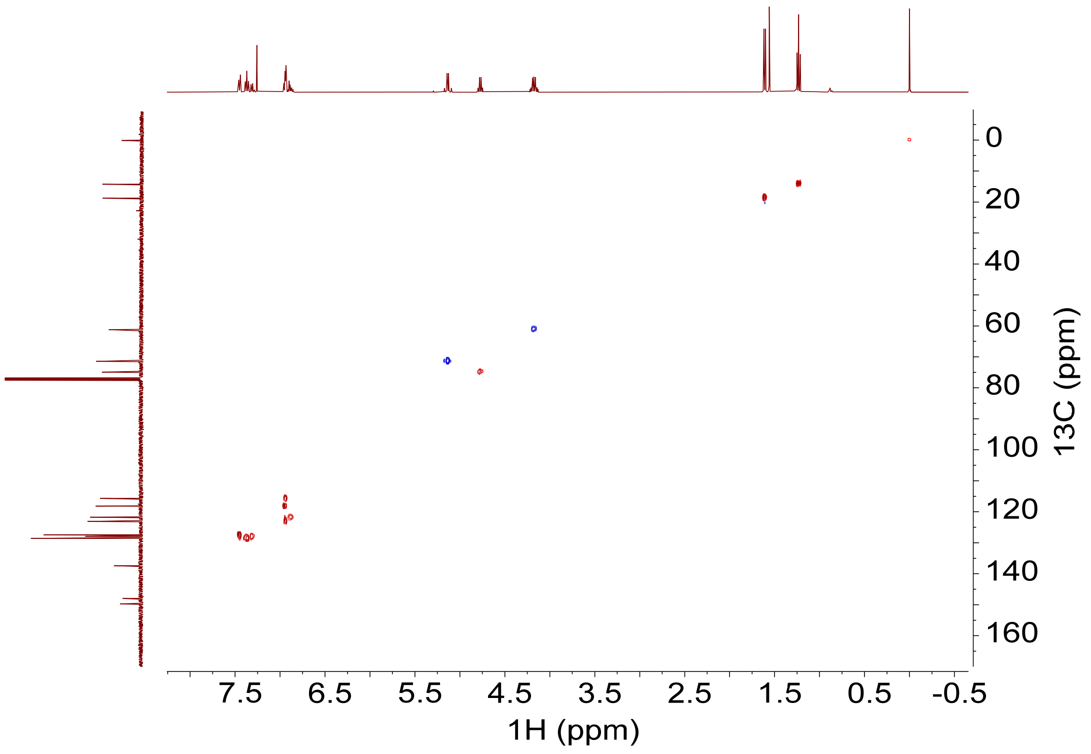

*Fig. S116. ^{1}H-^{13}C HSQC NMR spectrum of (R)-**5** (400 MHz, $CDCl_3$, 298 K).*

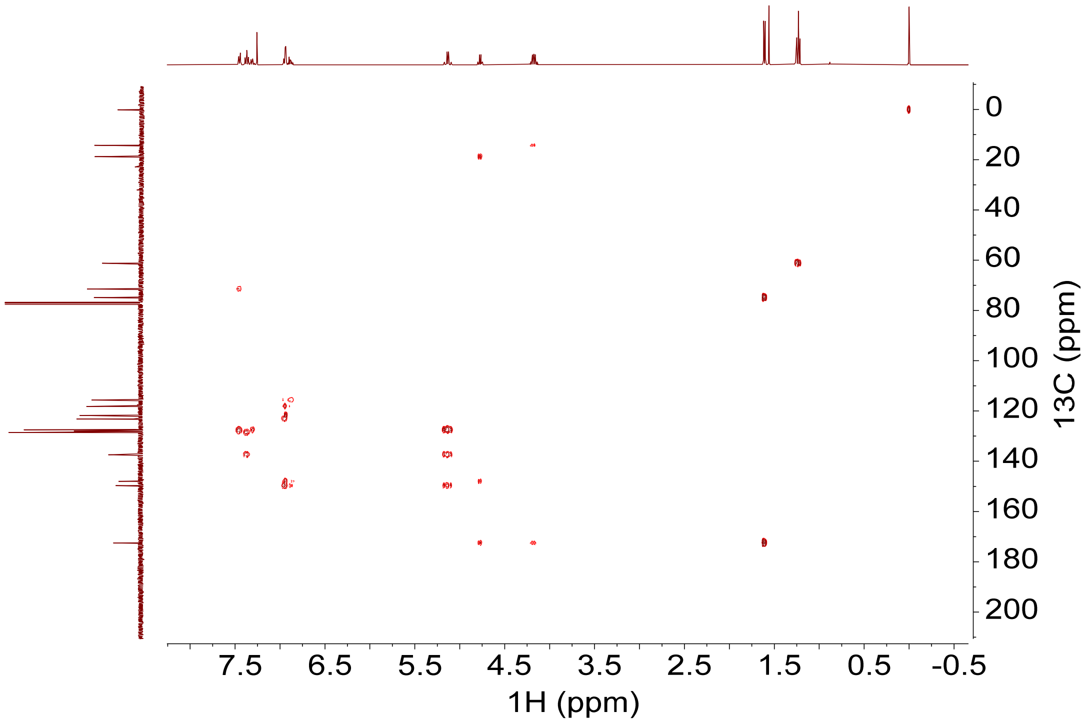

*Fig. S117. ^1H-^{13}C HMBC NMR spectrum of (R)-**5** (400 MHz, $CDCl_3$, 298 K).*

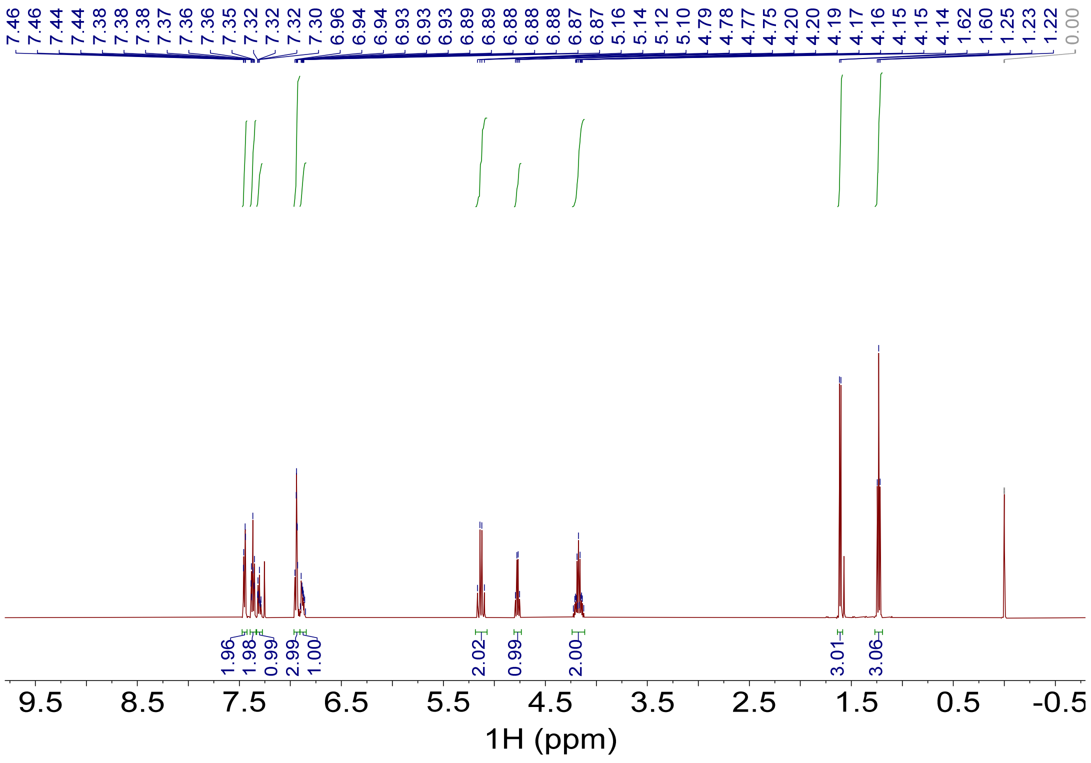

*Fig. S118. ^1H NMR spectrum of (S)-**5** (500 MHz, $CDCl_3$, 298 K).*

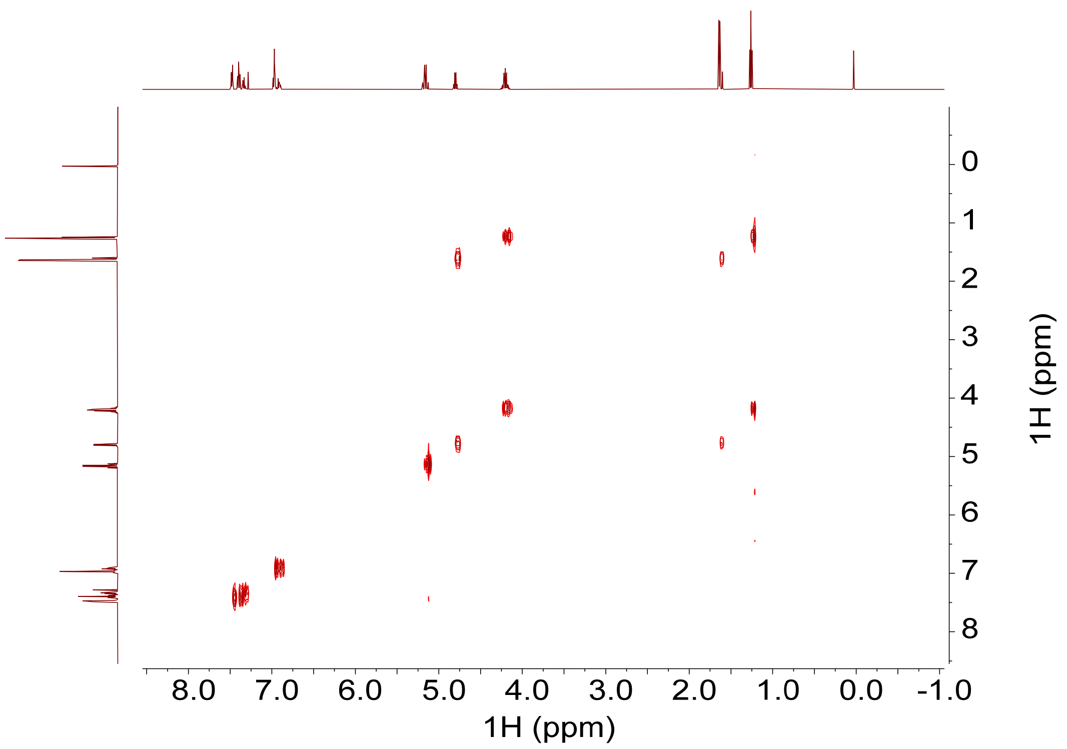

*Fig. S119. ^{1}H-^{1}H COSY NMR spectrum of (S)-**5** (500 MHz, $CDCl_3$, 298 K).*

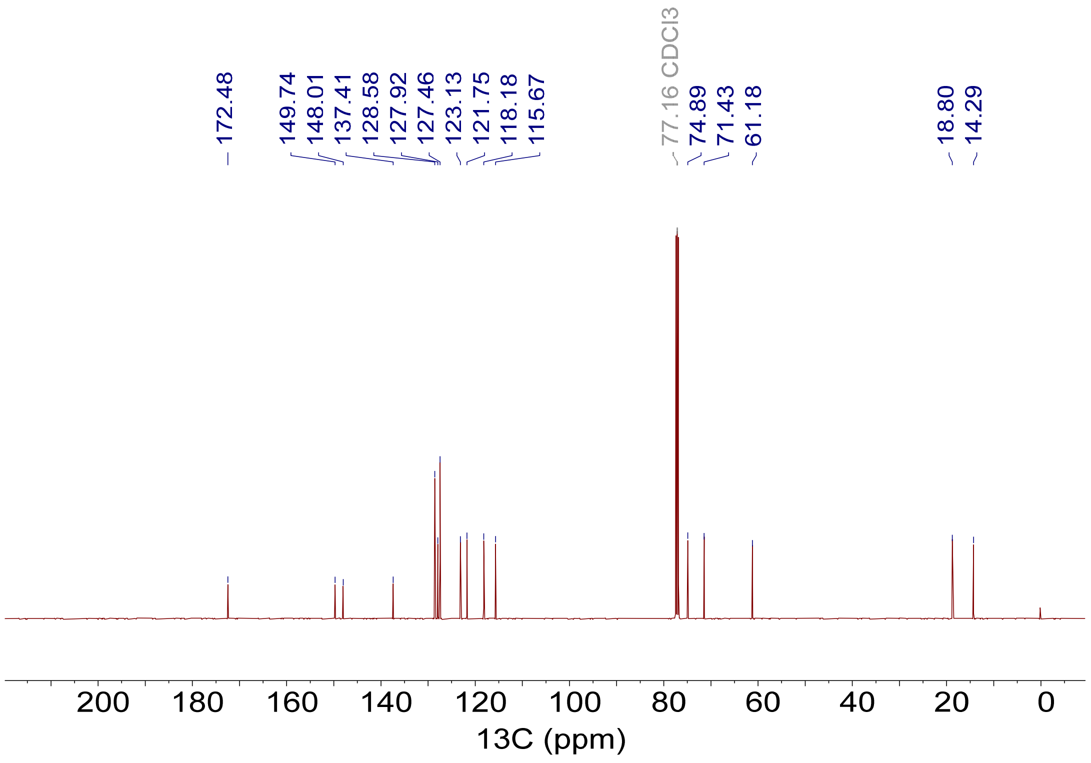

*Fig. S120. ^{13}C NMR spectrum of (S)-**5** (CPD, 126 MHz, $CDCl_3$, 298 K).*

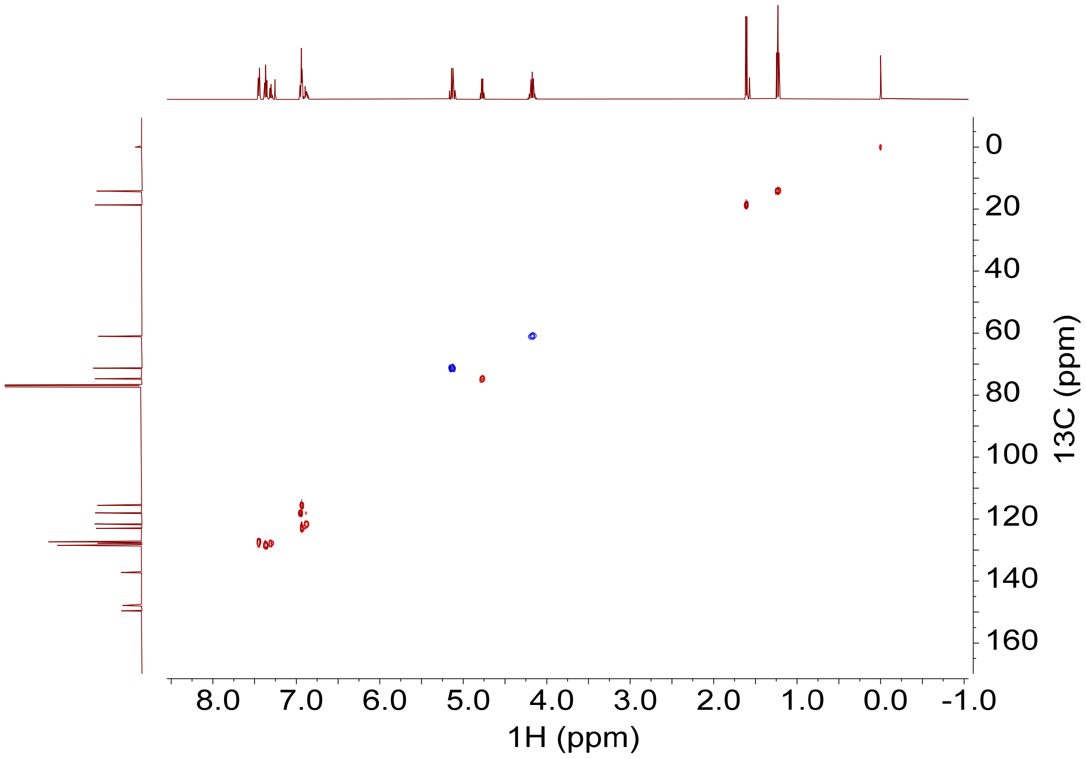

*Fig. S121. ^{1}H-^{13}C HSQC NMR spectrum of (S)-**5** (500 MHz, $CDCl_3$, 298 K).*

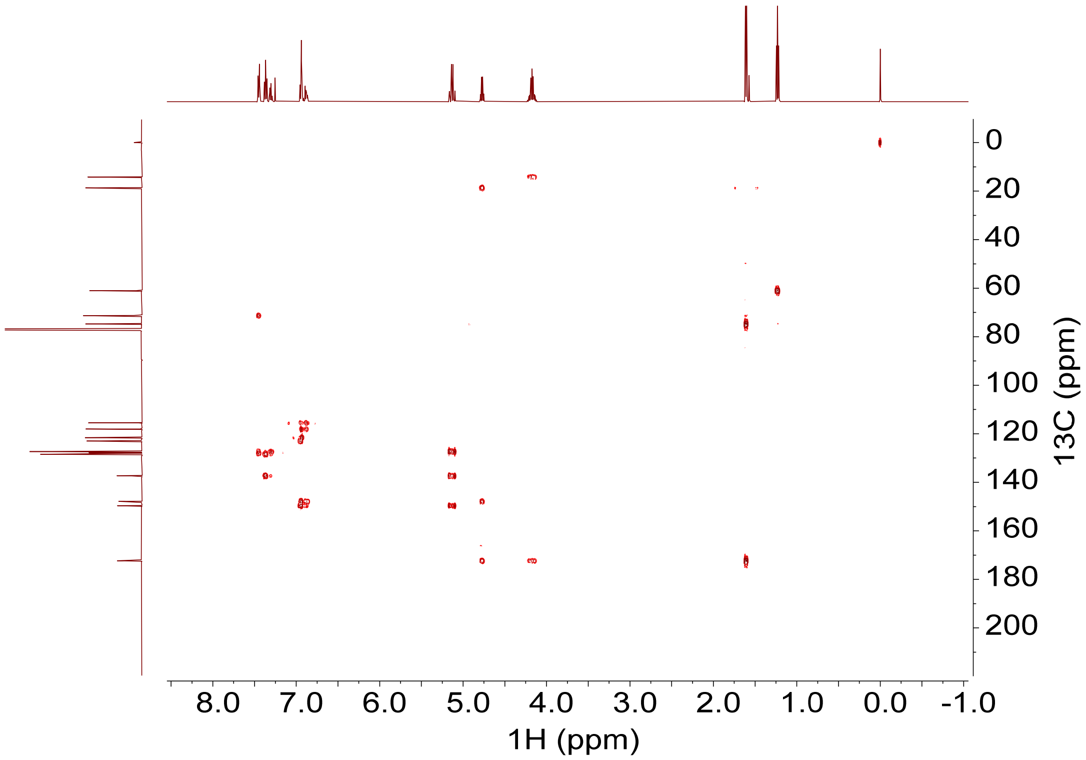

*Fig. S122. ^{1}H-^{13}C HMBC NMR spectrum of (S)-**5** (500 MHz, $CDCl_3$, 298 K).*

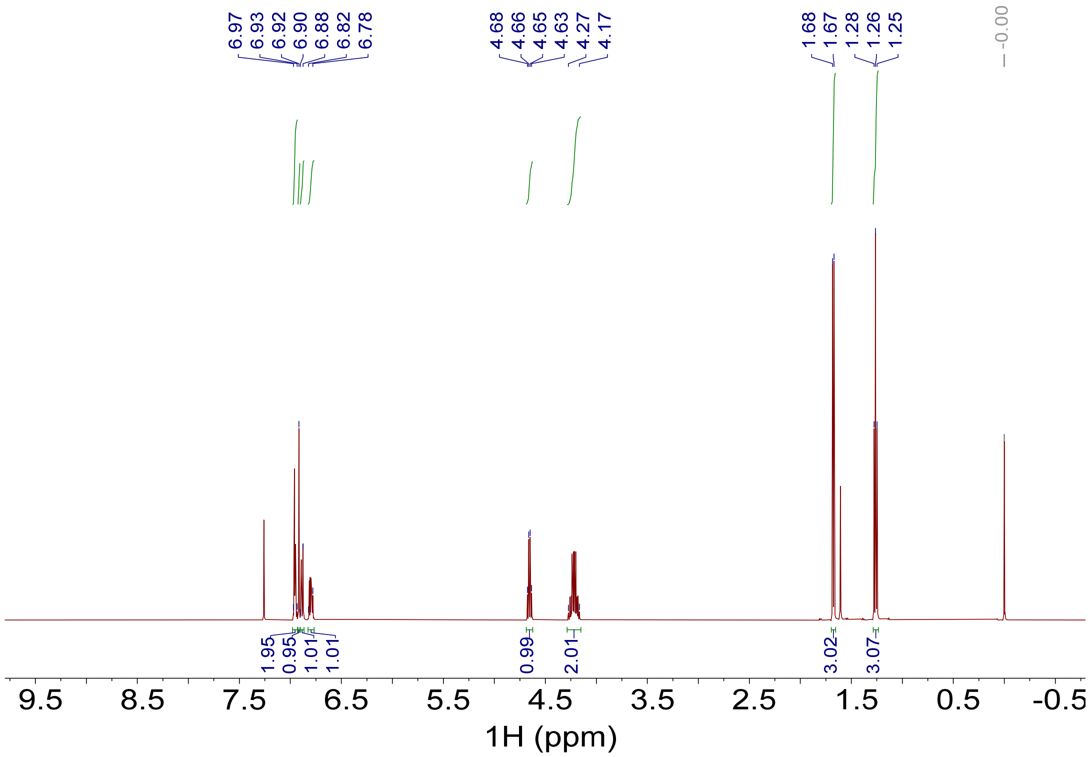

*Fig. S123. 1H NMR spectrum of (R)-**6** (500 MHz, CDCl$_3$, 298 K).*

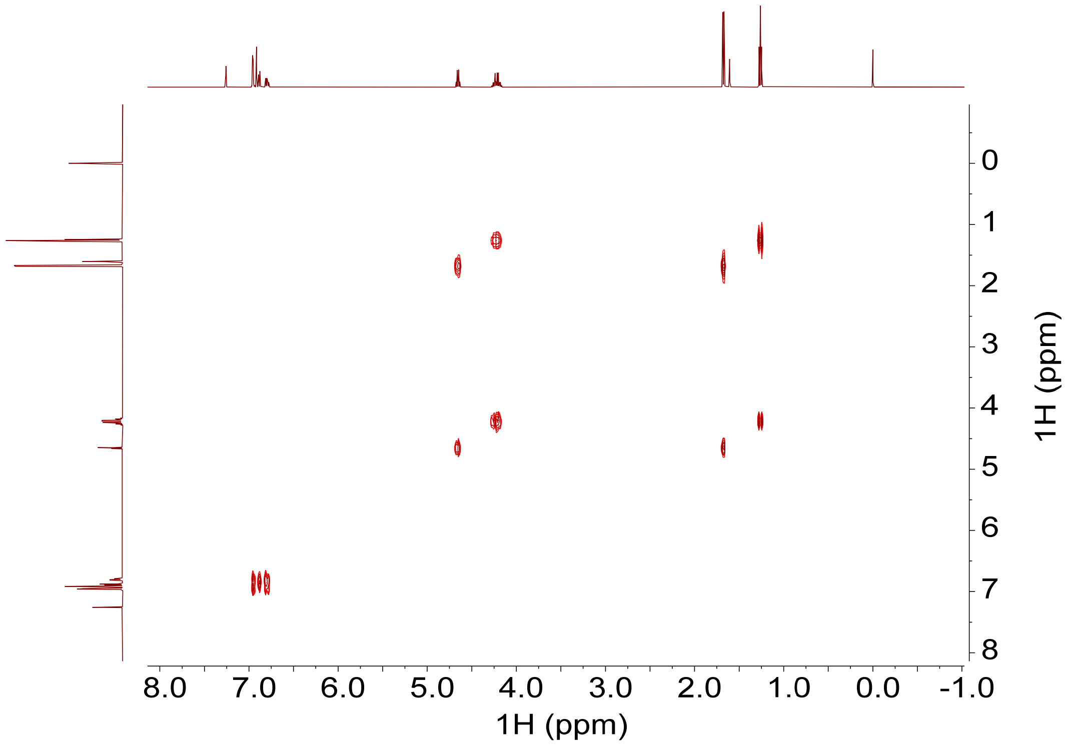

*Fig. S124. 1H-1H COSY NMR spectrum of (R)-**6** (500 MHz, CDCl$_3$, 298 K).*

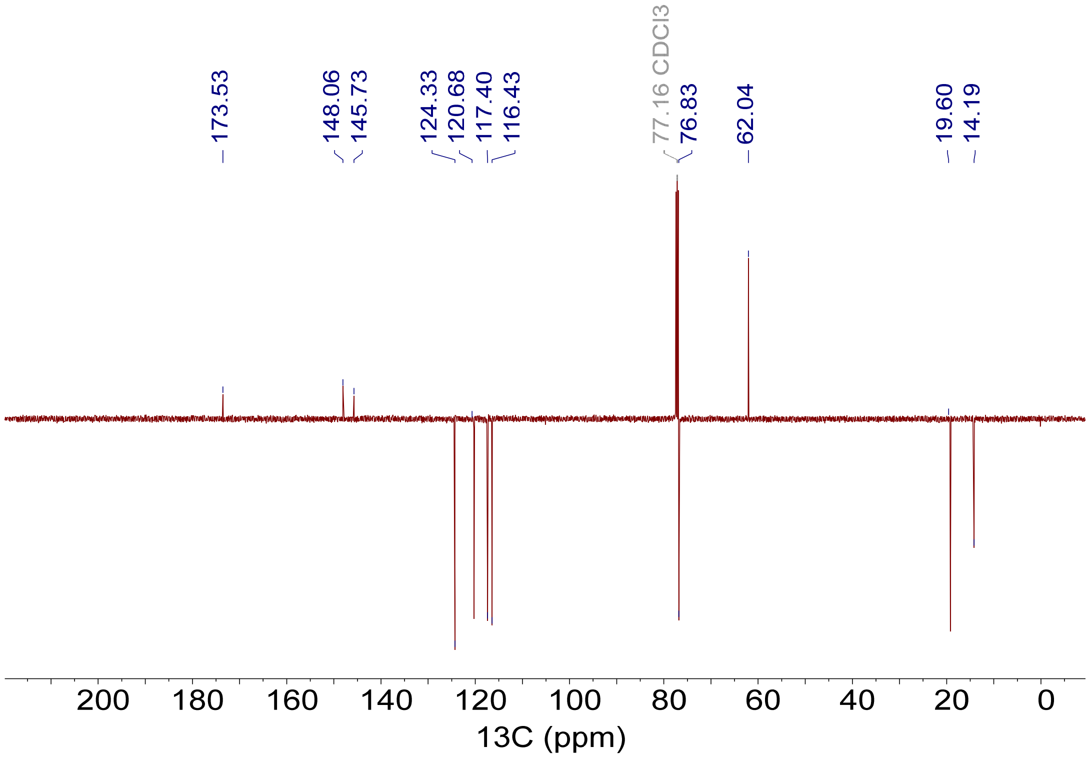

*Fig. S125. ^{13}C NMR spectrum of (R)-**6** (APT, 126 MHz, $CDCl_3$, 298 K).*

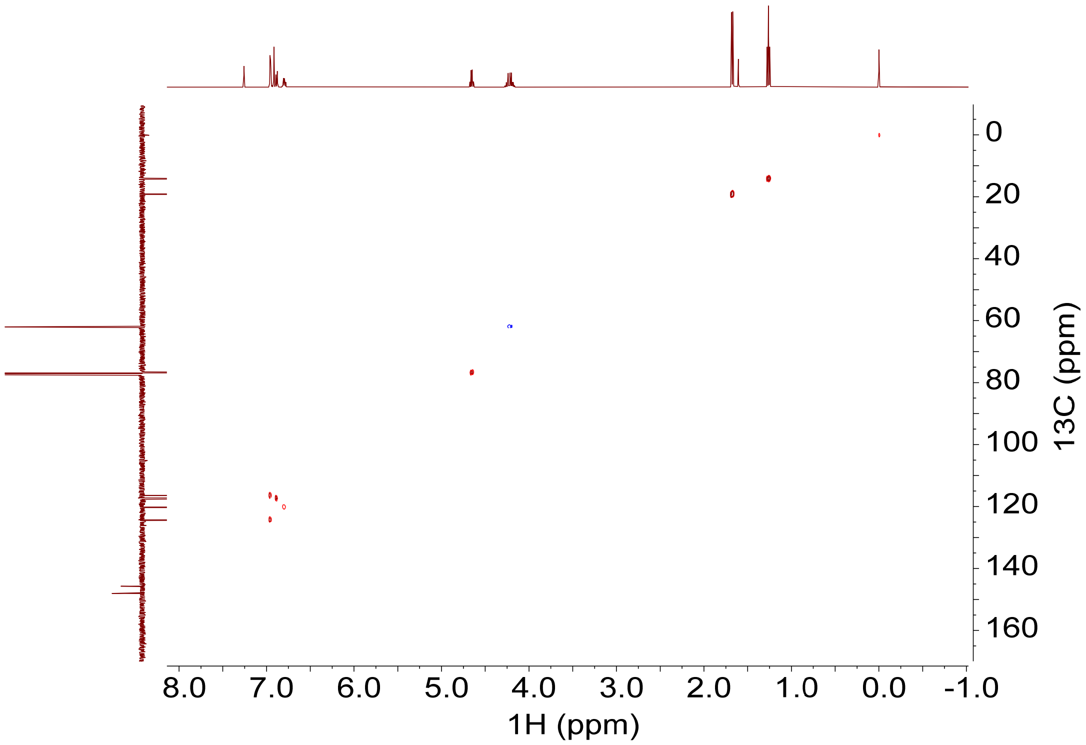

*Fig. 126. ^{1}H-^{13}C HSQC NMR spectrum of (R)-**6** (500 MHz, $CDCl_3$, 298 K).*

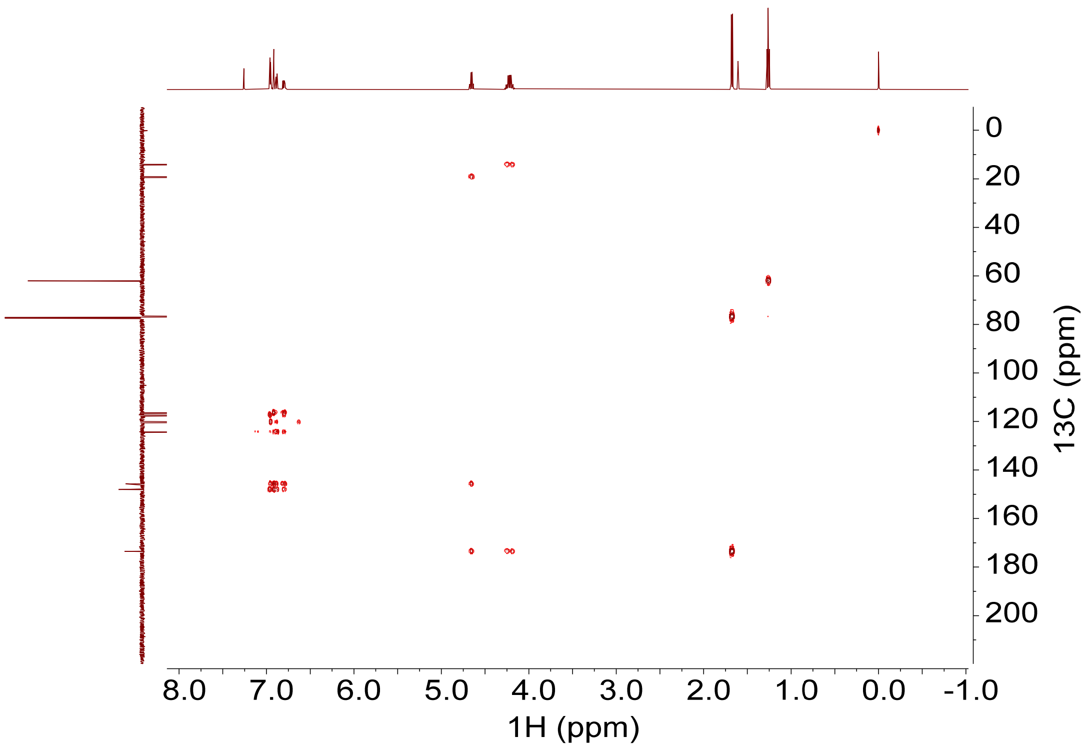

*Fig. S127. ^{1}H-^{13}C HMBC NMR spectrum of (R)-**6** (500 MHz, $CDCl_3$, 298 K).*

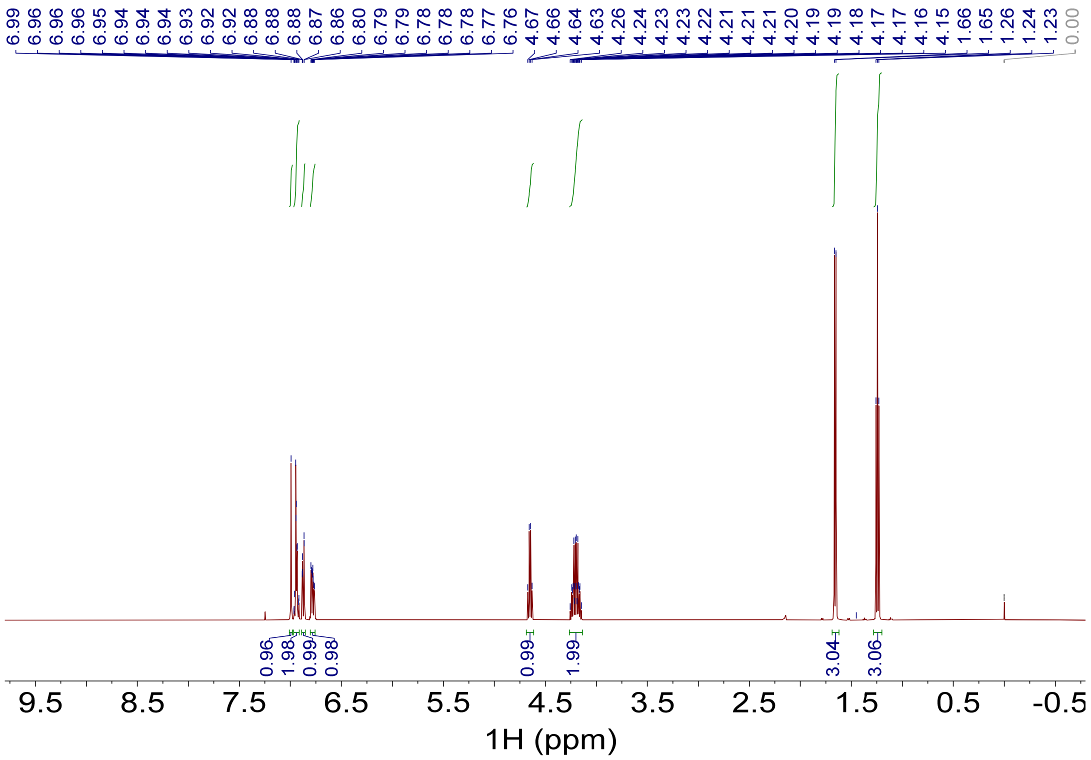

*Fig. S128. ^{1}H NMR spectrum of (S)-**6** (500 MHz, $CDCl_3$, 298 K).*

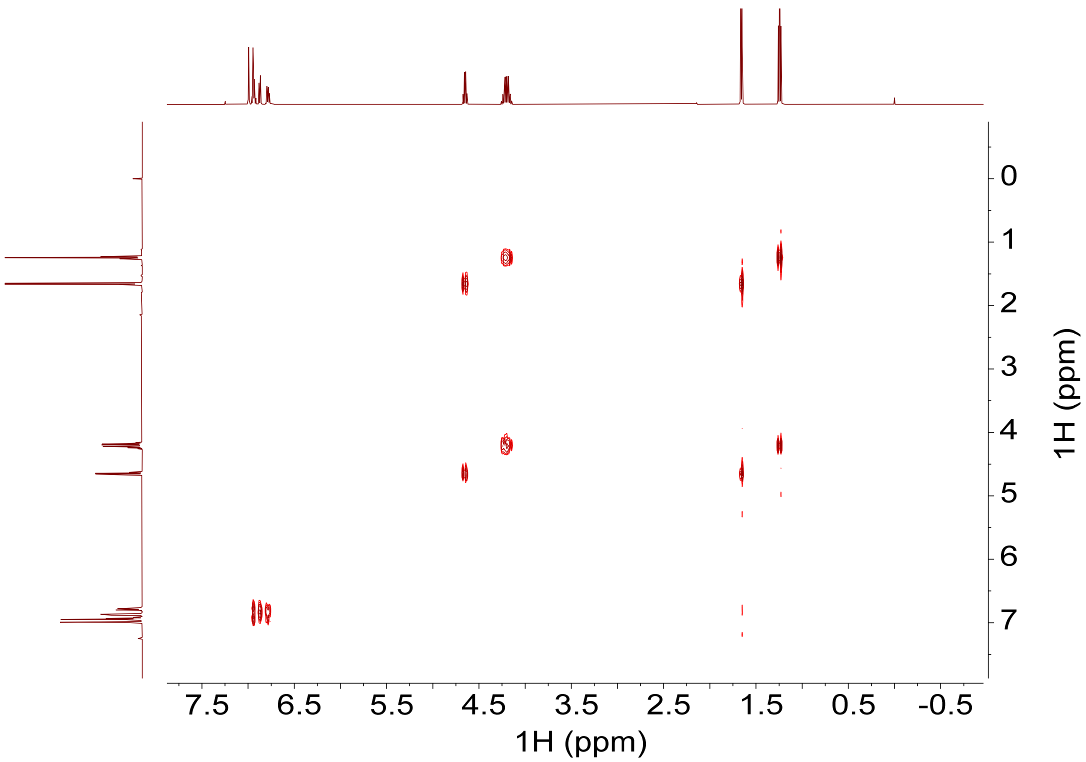

*Fig. S129. ^{1}H-^{1}H COSY NMR spectrum of (S)-**6** (500 MHz, $CDCl_3$, 298 K).*

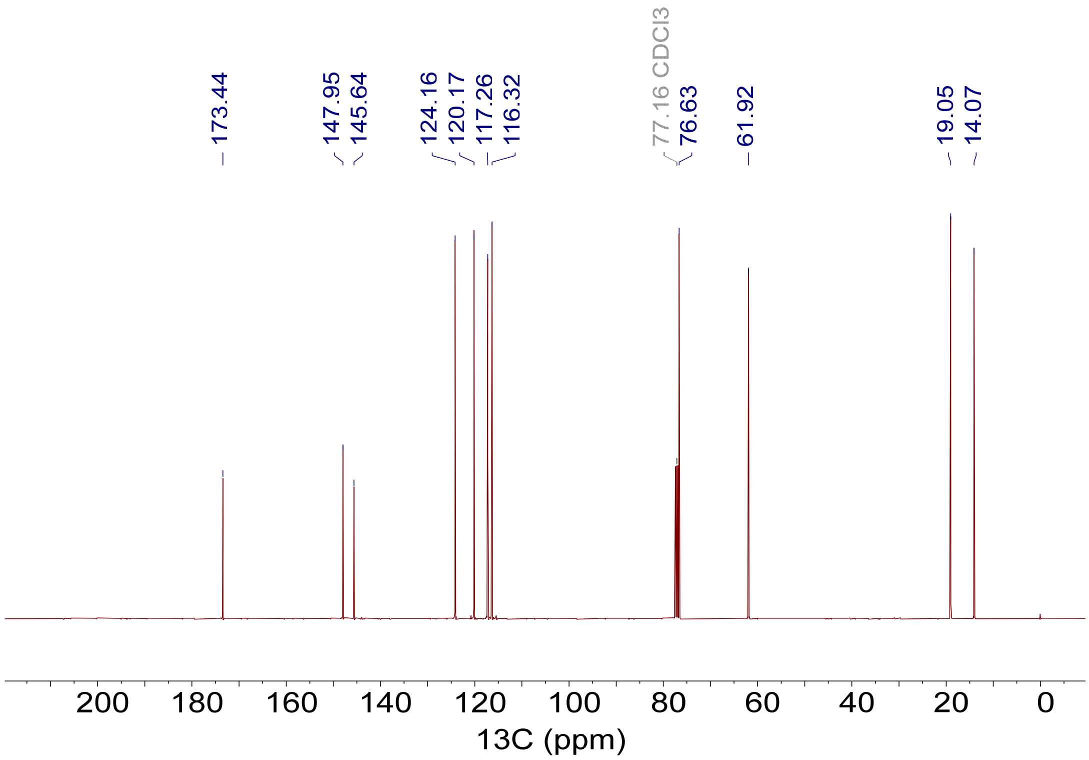

*Fig. S130. ^{13}C NMR spectrum of (S)-**6** (CPD, 126 MHz, $CDCl_3$, 298 K).*

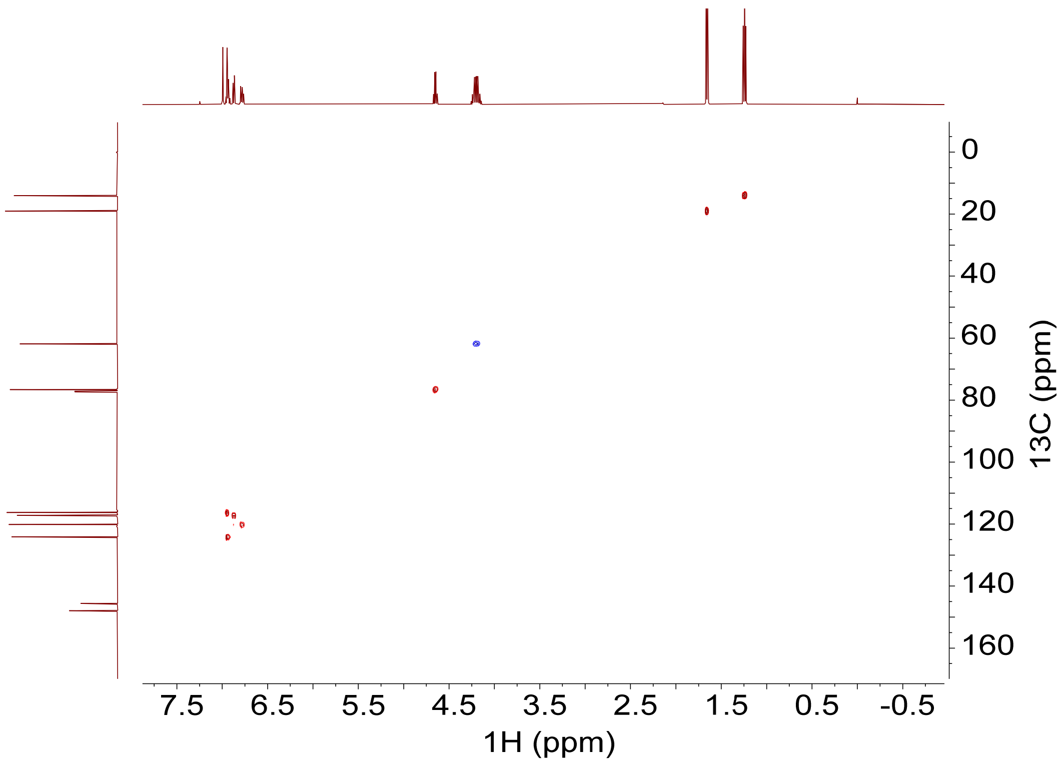

Fig. S131. *^{1}H-^{13}C HSQC NMR spectrum of (S)-**6** (500 MHz, $CDCl_3$, 298 K).*

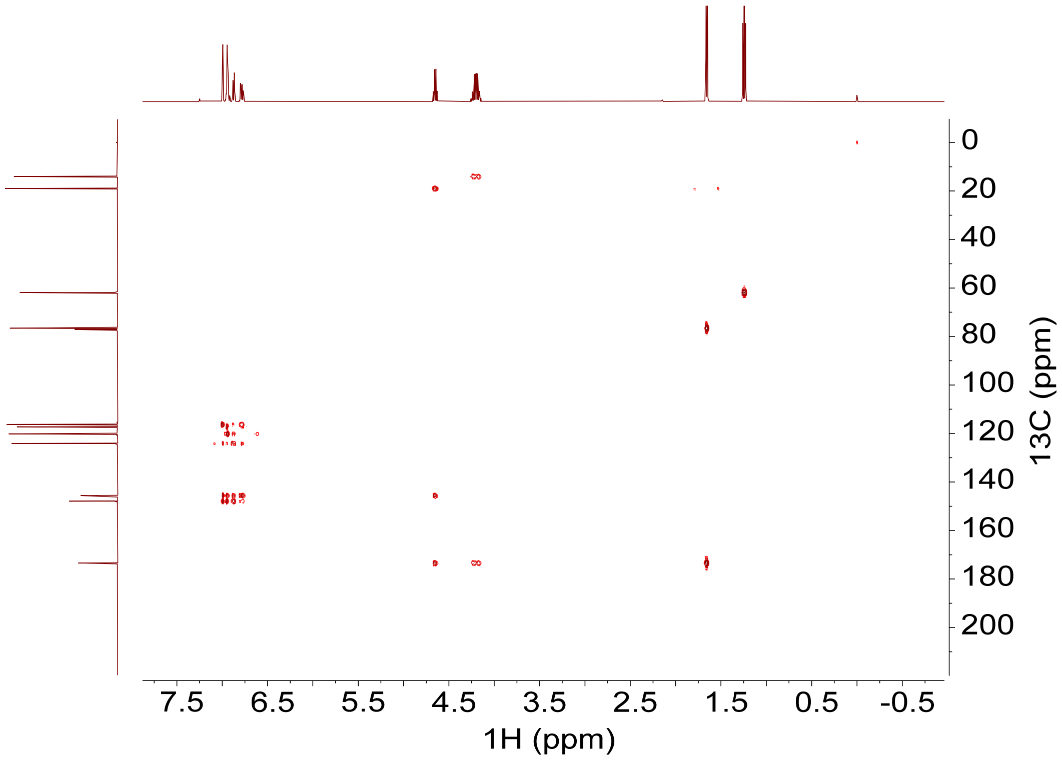

Fig. S132. *^{1}H-^{13}C HMBC NMR spectrum of (S)-**6** (500 MHz, $CDCl_3$, 298 K).*

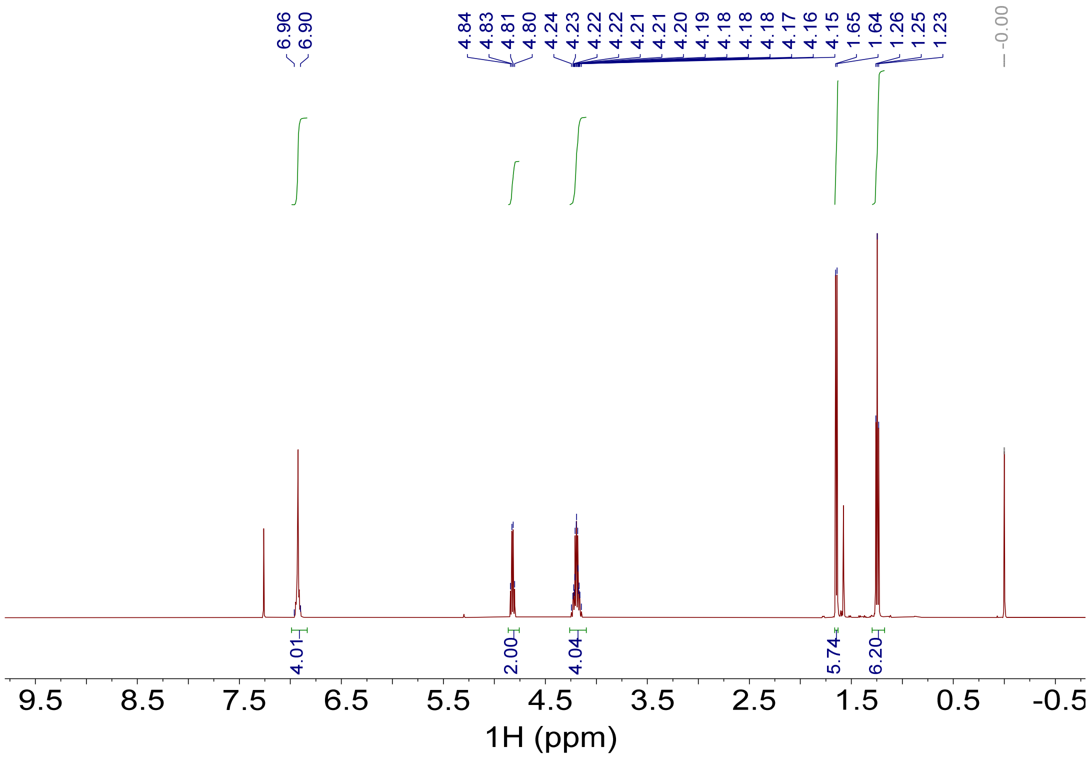

*Fig. S133. ^{1}H NMR spectrum of (R,R)-**7** (500 MHz, $CDCl_3$, 298 K).*

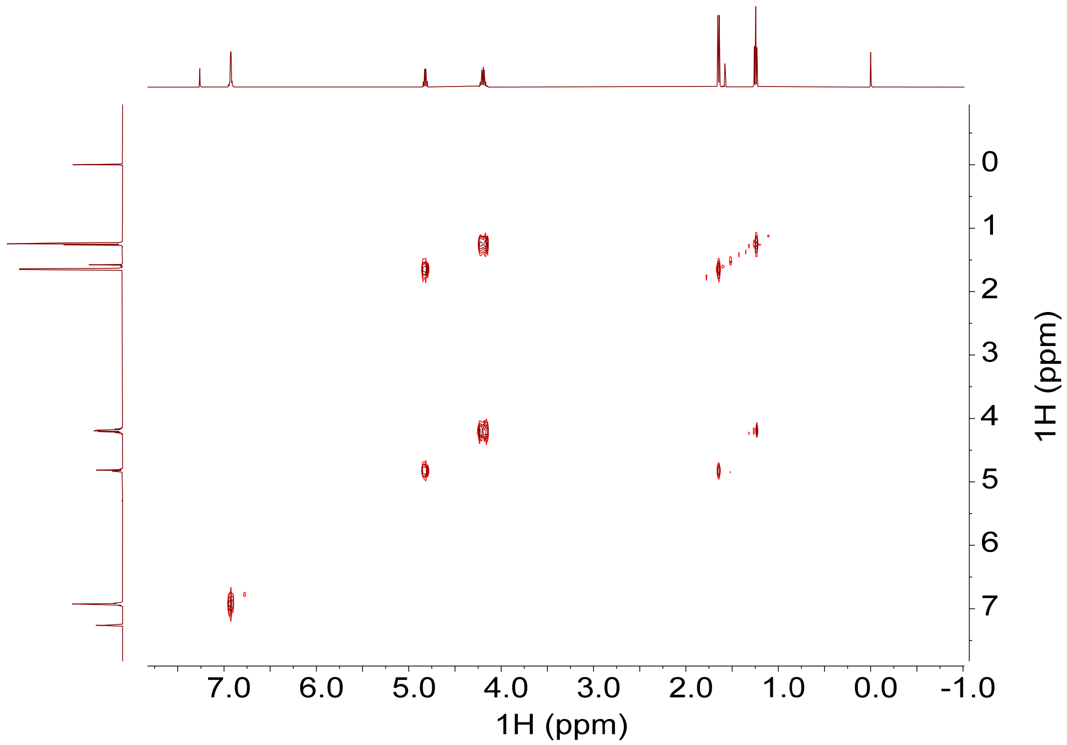

*Fig. S134. ^{1}H-^{1}H COSY NMR spectrum of (R,R)-**7** (500 MHz, $CDCl_3$, 298 K).*

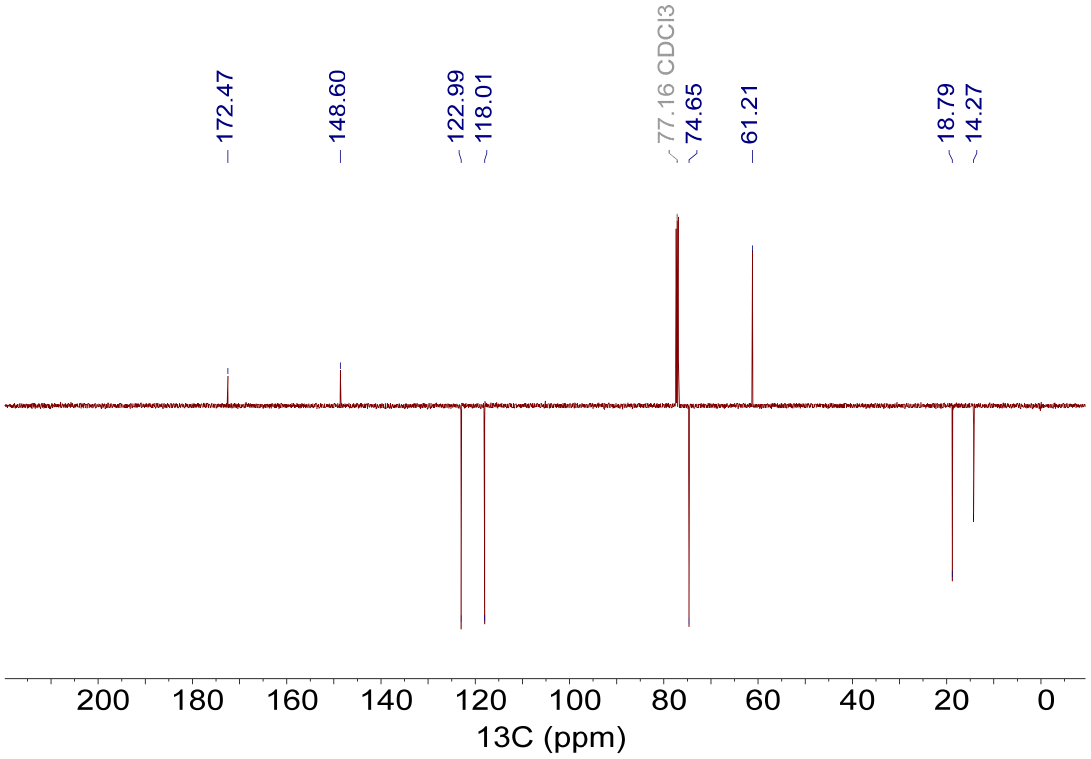

*Fig. S135. ^{13}C NMR spectrum of (R,R)-**7** (APT, 126 MHz, $CDCl_3$, 298 K).*

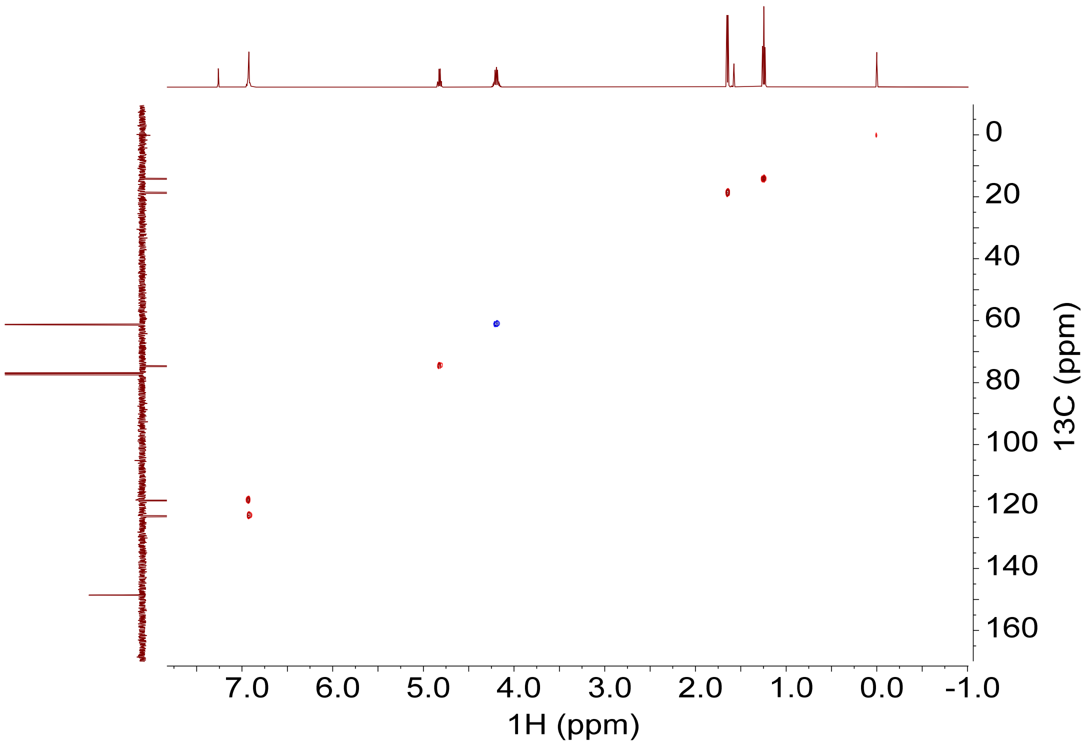

*Fig. S136. ^{1}H-^{13}C HSQC NMR spectrum of (R,R)-**7** (500 MHz, $CDCl_3$, 298 K).*

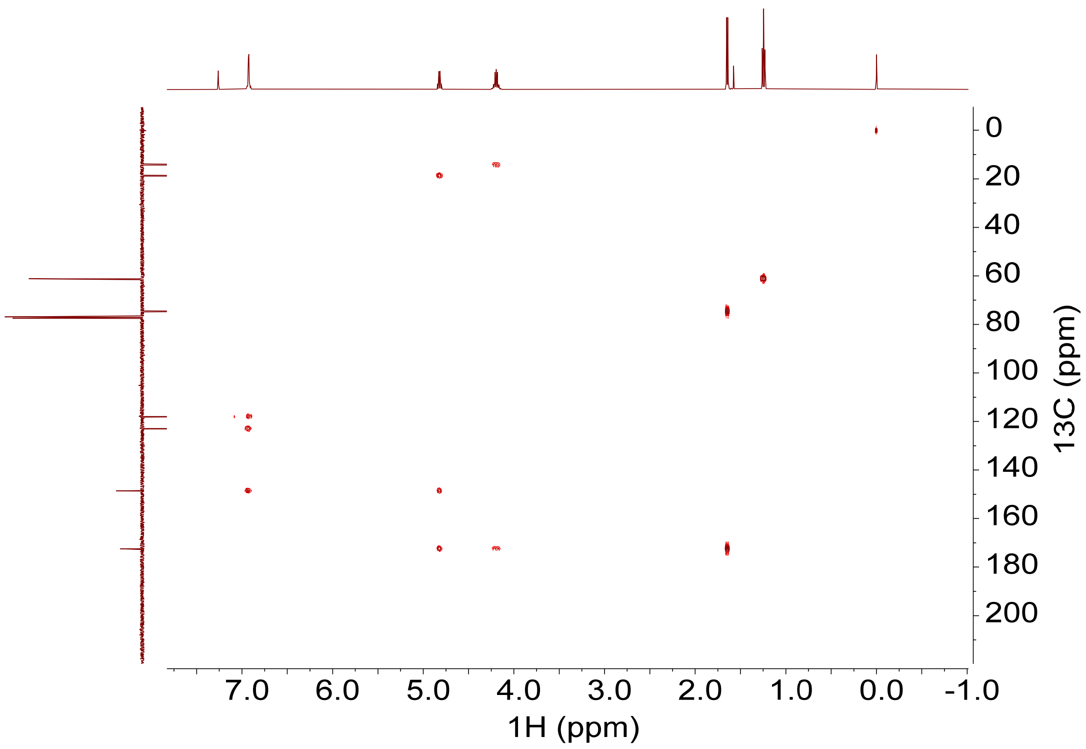

*Fig. S137. ^{1}H-^{13}C HMBC NMR spectrum of (R,R)-**7** (500 MHz, $CDCl_3$, 298 K).*

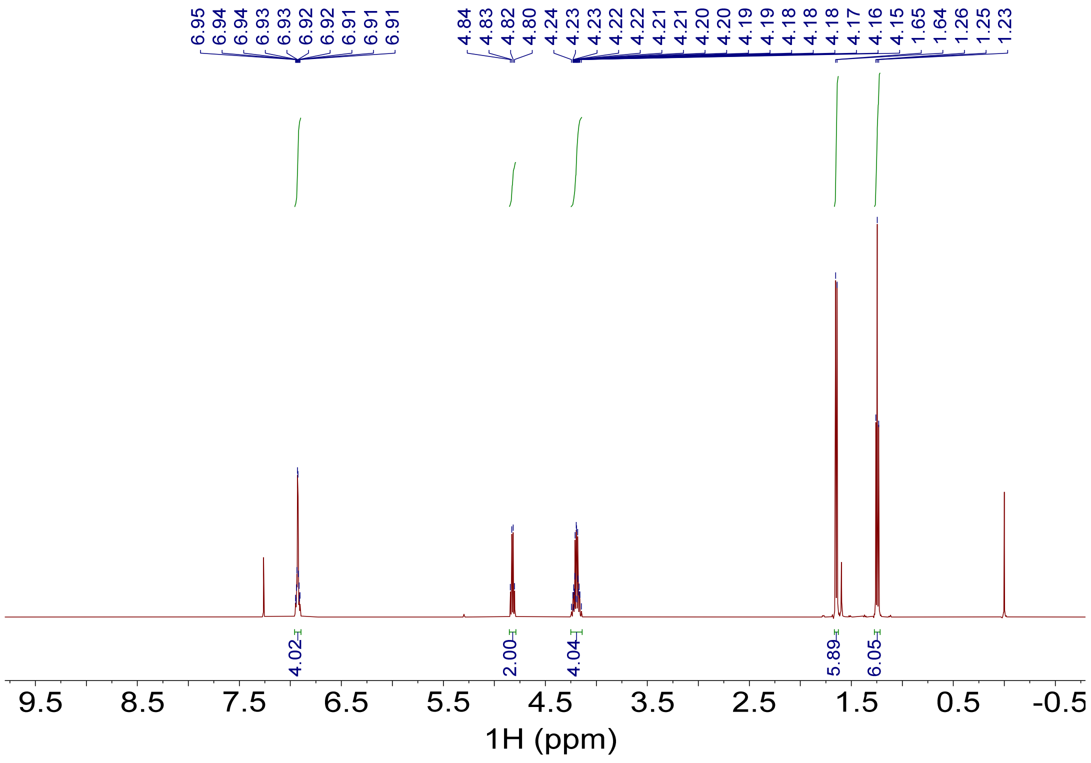

*Fig. S138. ^{1}H NMR spectrum of (S,S)-**7** (500 MHz, $CDCl_3$, 298 K).*

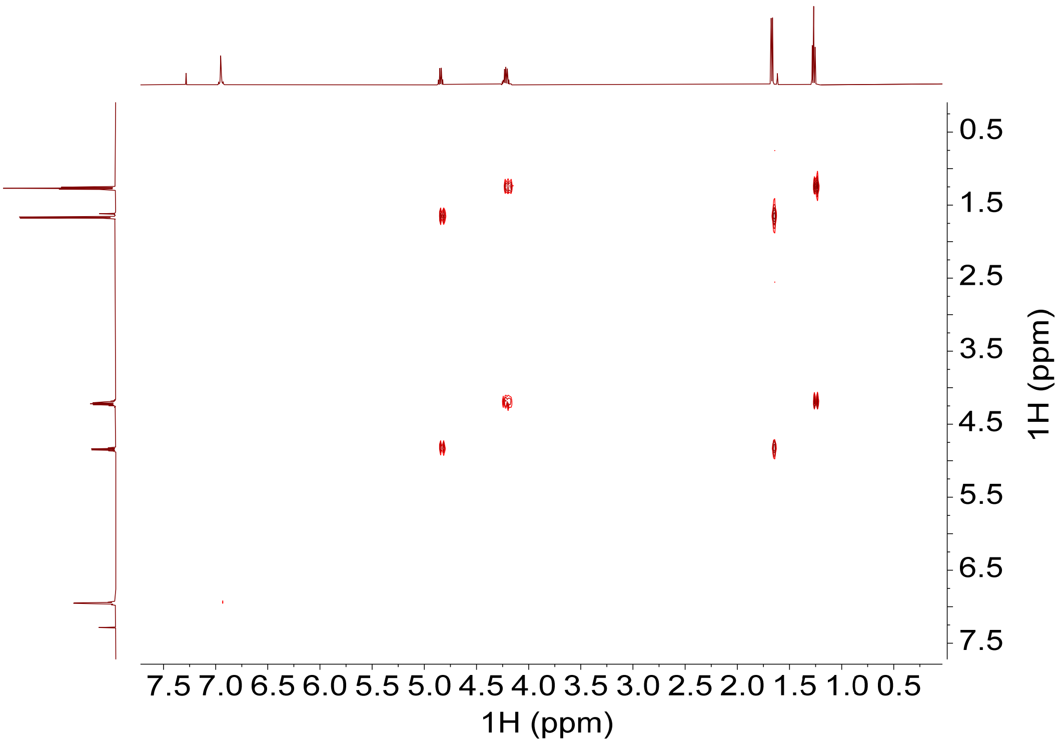

*Fig. S139. ^{1}H-^{1}H COSY NMR spectrum of (S,S)-**7** (500 MHz, $CDCl_3$, 298 K).*

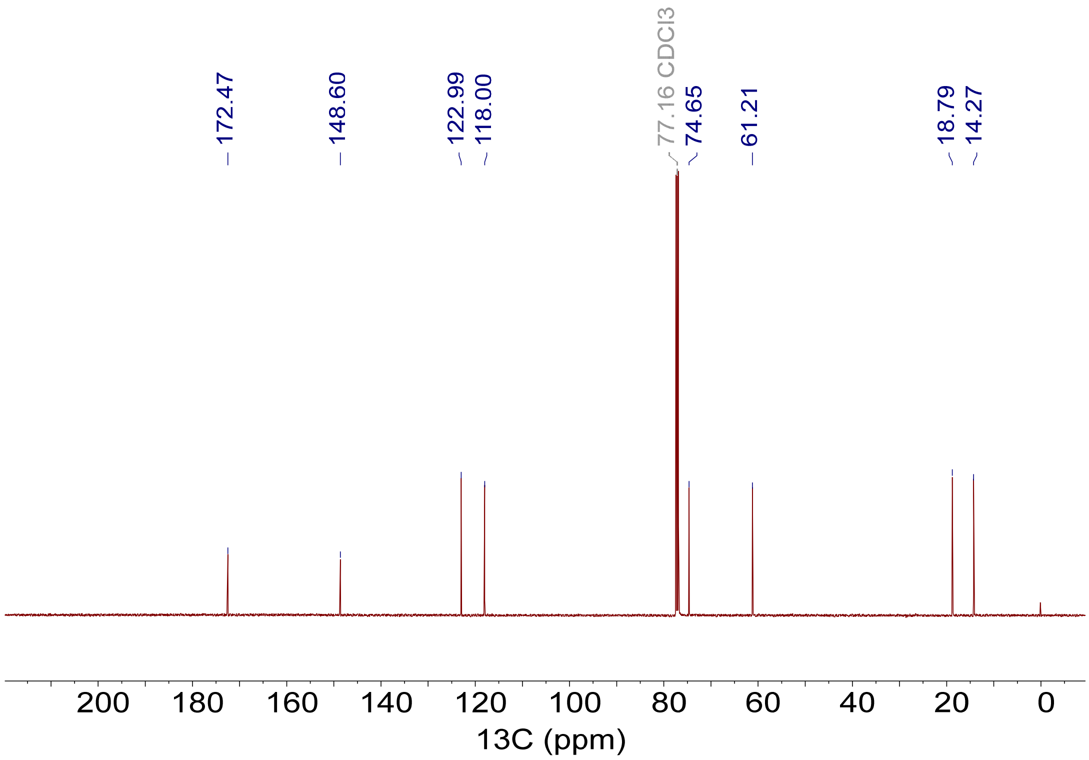

*Fig. S140. ^{13}C NMR spectrum of (S,S)-**7** (CPD, 126 MHz, $CDCl_3$, 298 K).*

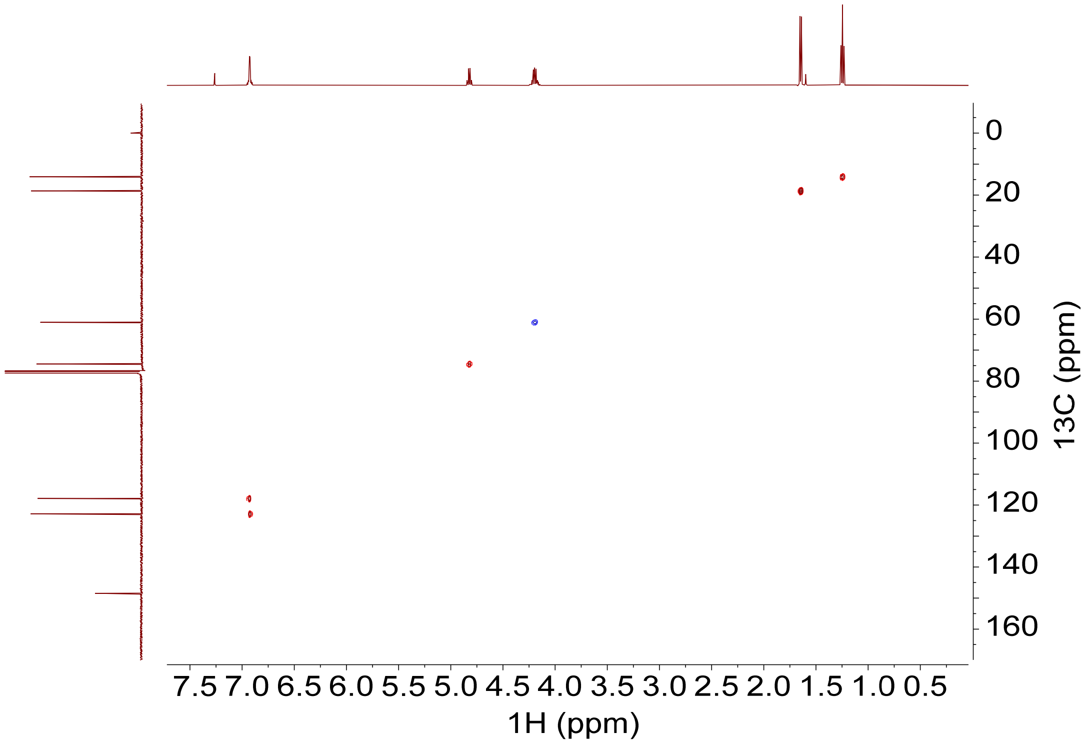

*Fig. S141. ^{1}H-^{13}C HSQC NMR spectrum of (S,S)-**7** (500 MHz, $CDCl_3$, 298 K).*

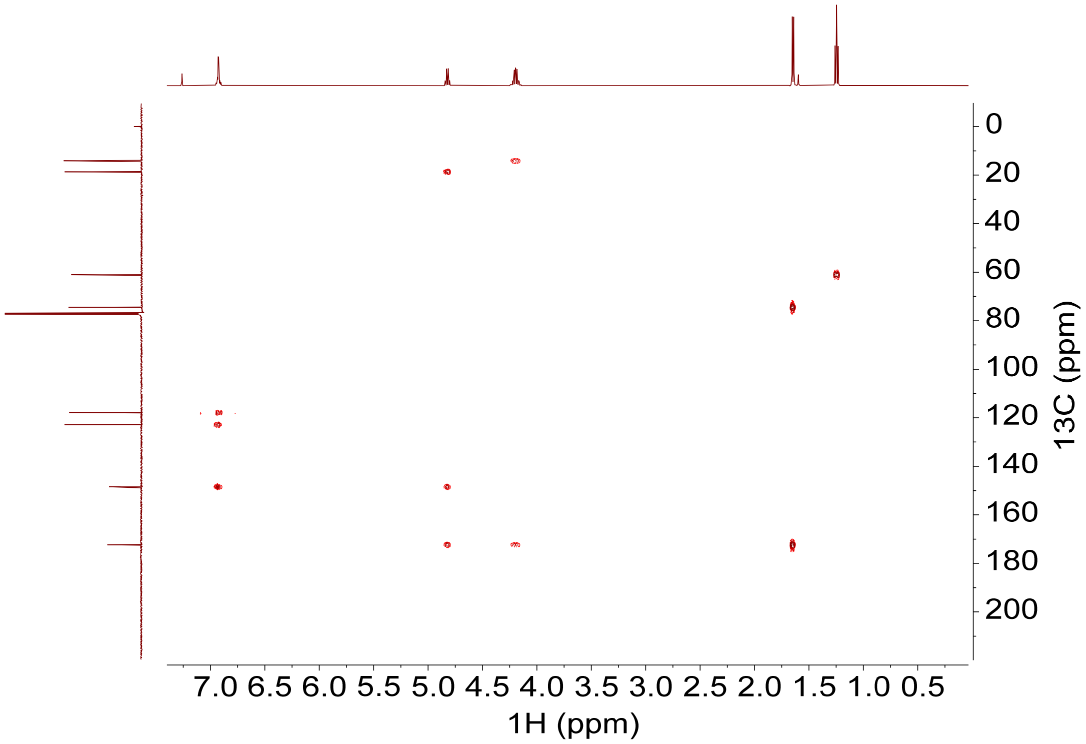

*Fig. S142. ^{1}H-^{13}C HMBC NMR spectrum of (S,S)-**7** (500 MHz, $CDCl_3$, 298 K).*

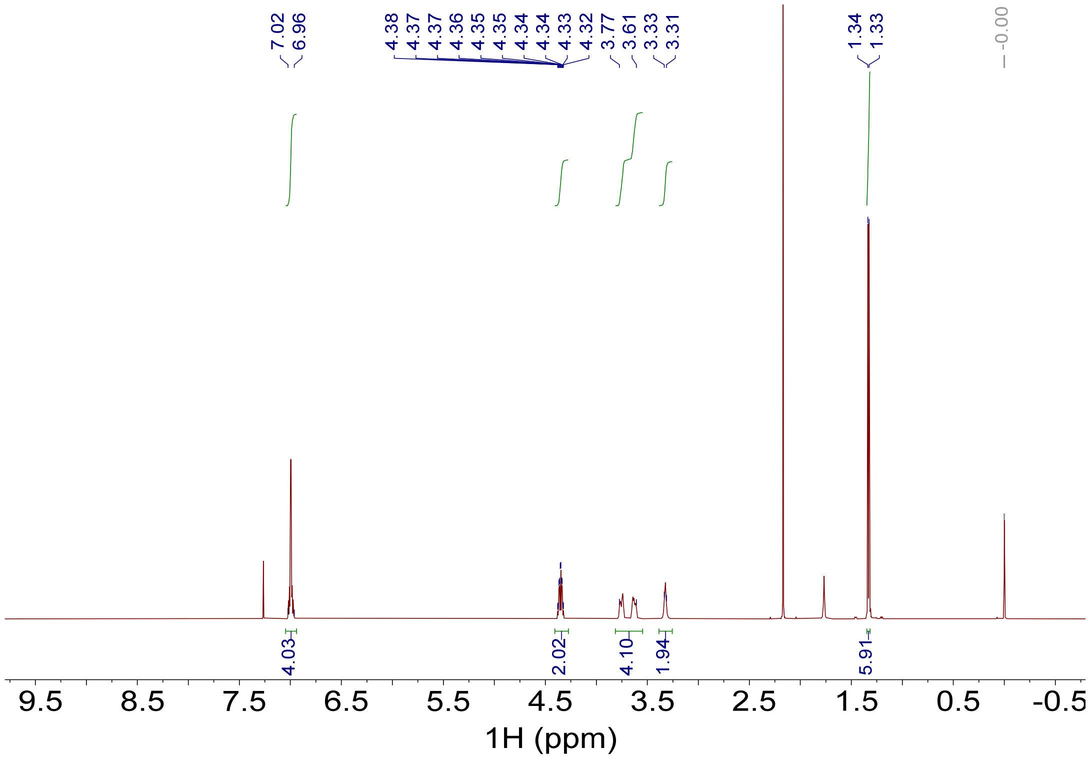

*Fig. S143. ^{1}H NMR spectrum of (R,R)-**8** (500 MHz, $CDCl_3$, 298 K).*

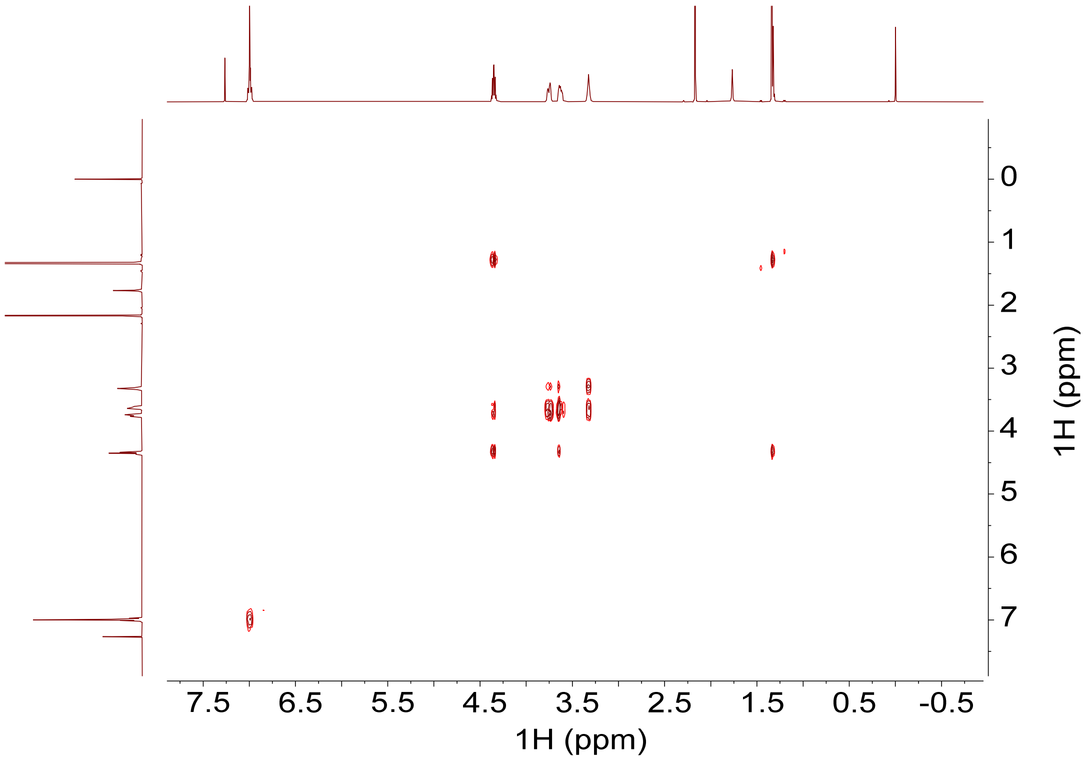

*Fig. S144. ^{1}H-^{1}H COSY NMR spectrum of (R,R)-**8** (500 MHz, $CDCl_3$, 298 K).*

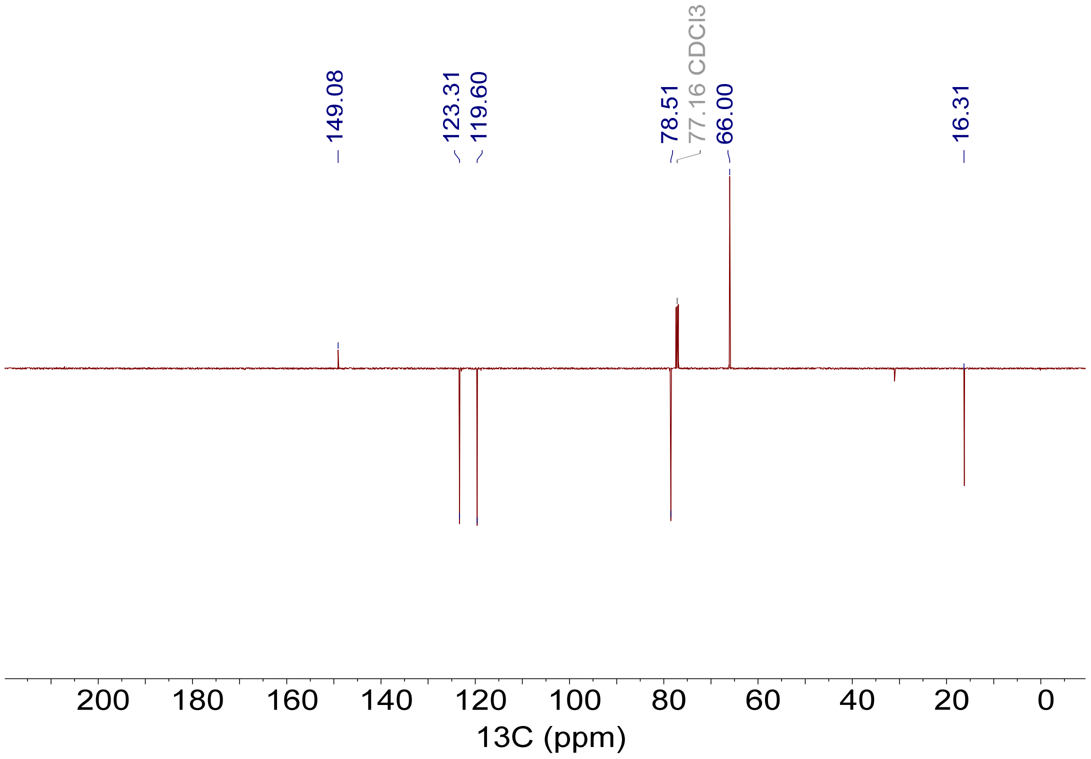

*Fig. S145. ^{13}C NMR spectrum of (R,R)-**8** (APT, 126 MHz, $CDCl_3$, 298 K).*

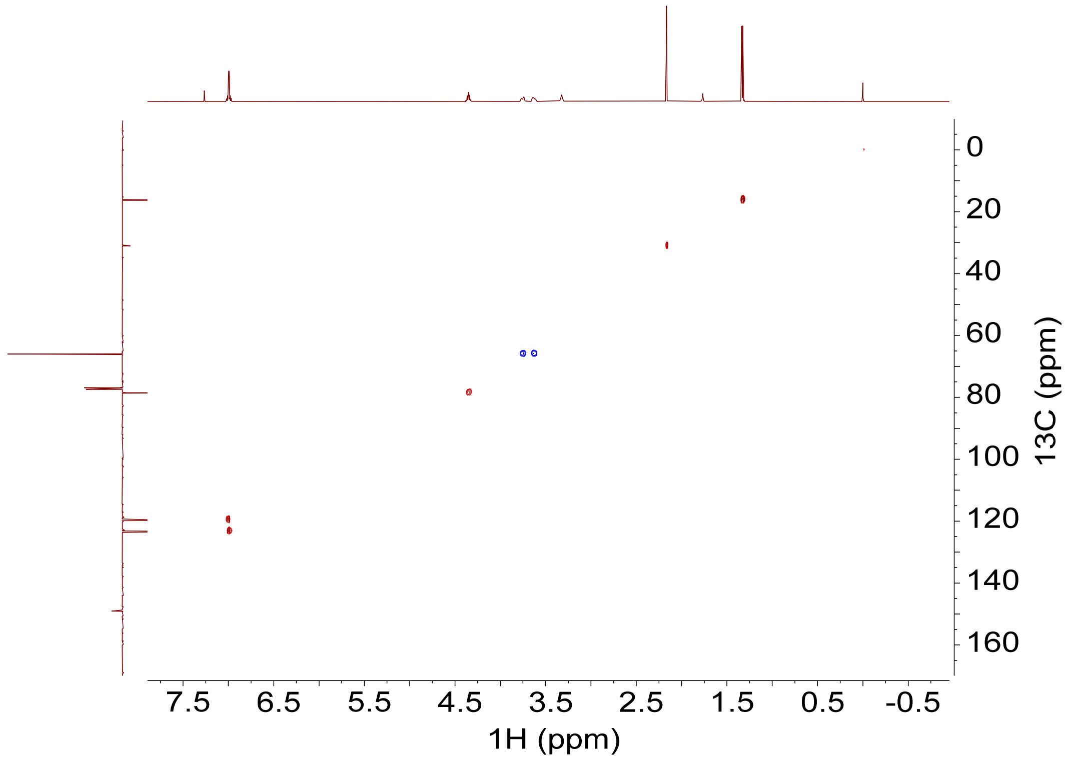

*Fig. S146. ^{1}H-^{13}C HSQC NMR spectrum of (R,R)-**8** (500 MHz, $CDCl_3$, 298 K).*

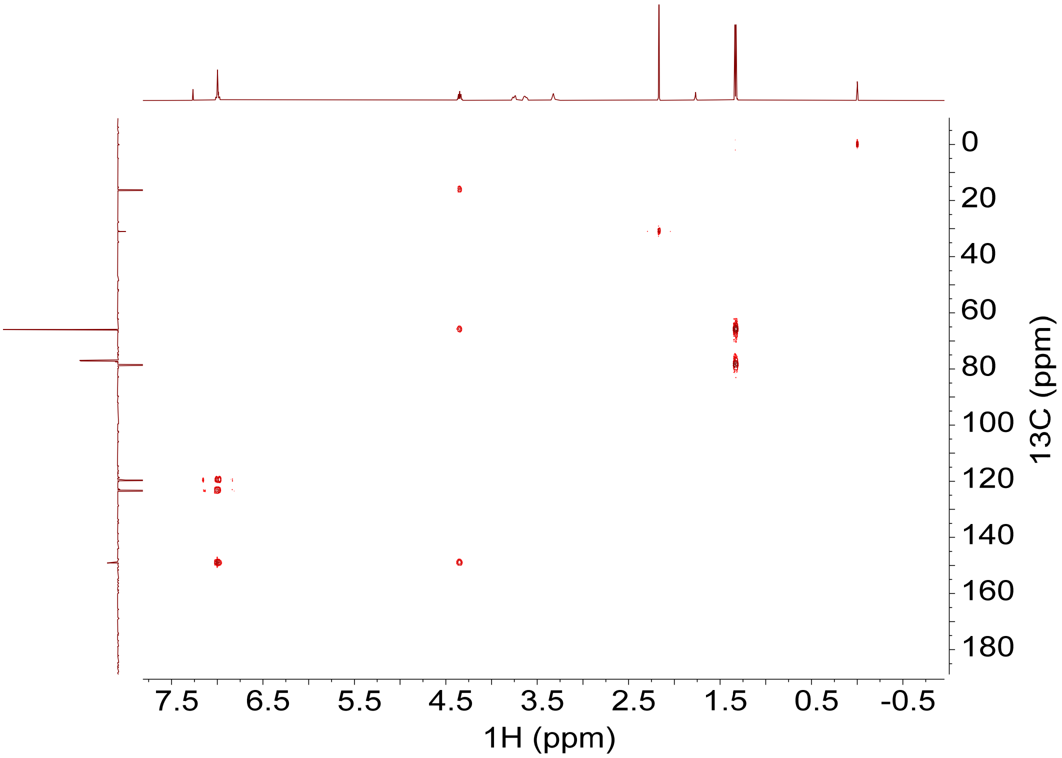

*Fig. S147. ^{1}H-^{13}C HMBC NMR spectrum of (R,R)-**8** (500 MHz, $CDCl_3$, 298 K).*

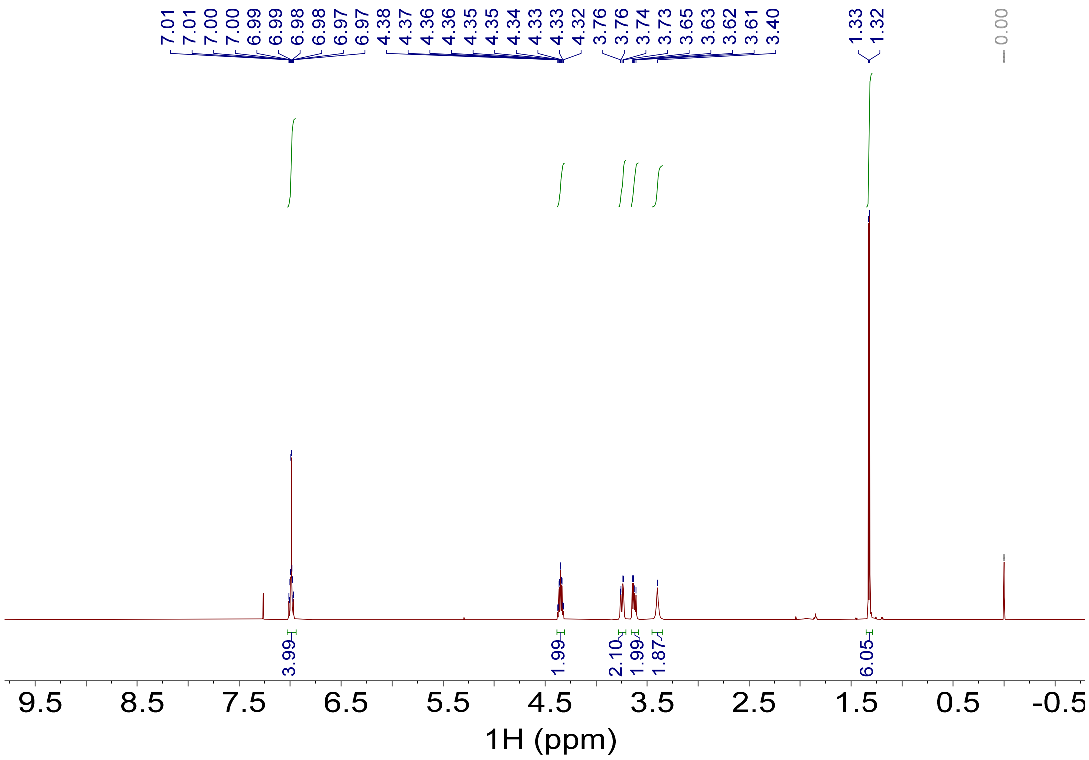

*Fig. S148. ^{1}H NMR spectrum of (S,S)-**8** (500 MHz, $CDCl_3$, 298 K).*

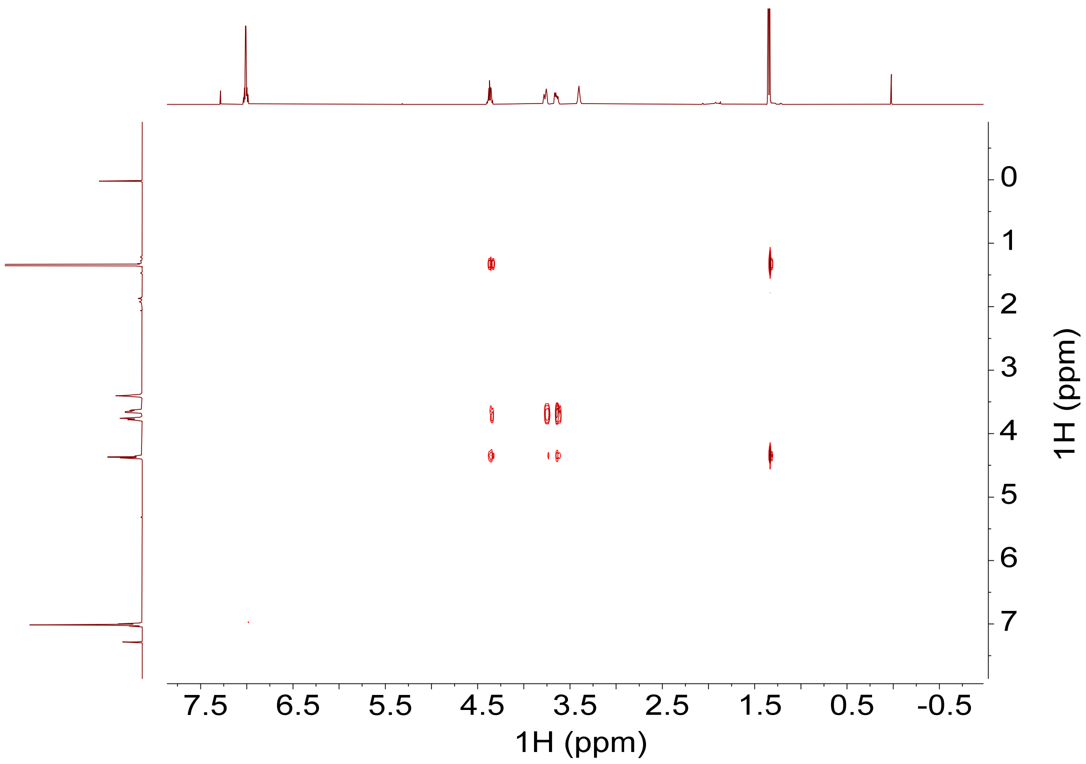

*Fig. S149. ¹H-¹H COSY NMR spectrum of (S,S)-**8** (500 MHz, $CDCl_3$, 298 K).*

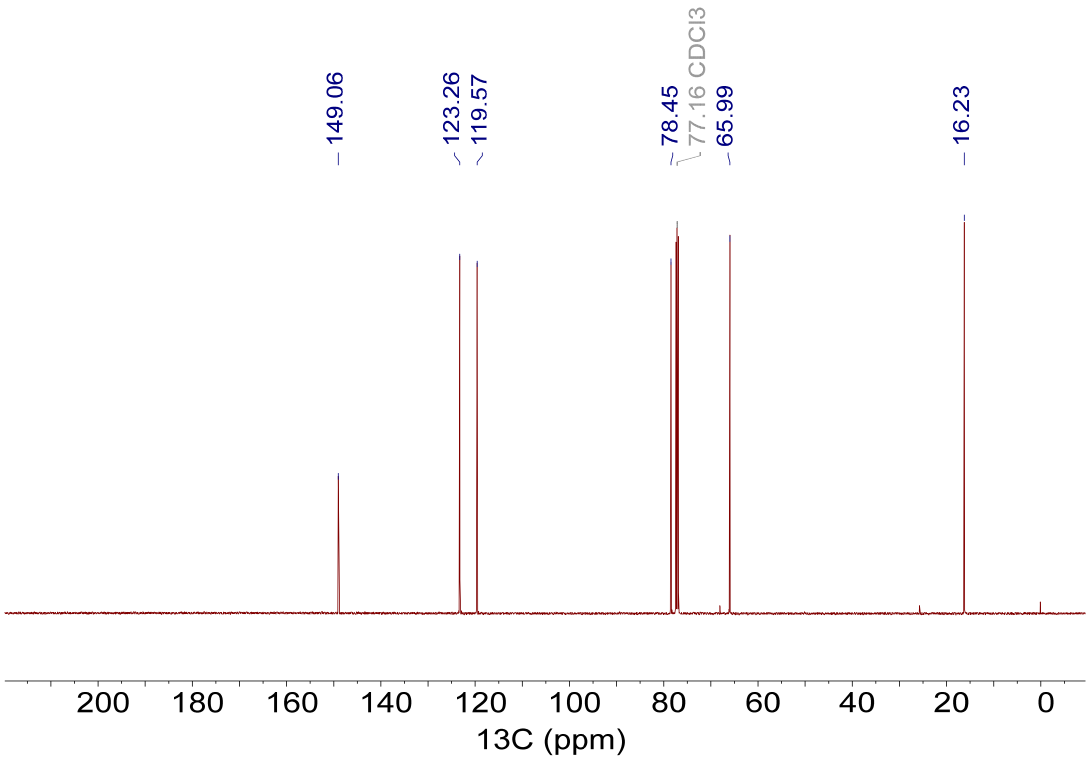

*Fig. S150. ¹³C NMR spectrum of (S,S)-**8** (CPD, 126 MHz, $CDCl_3$, 298 K).*

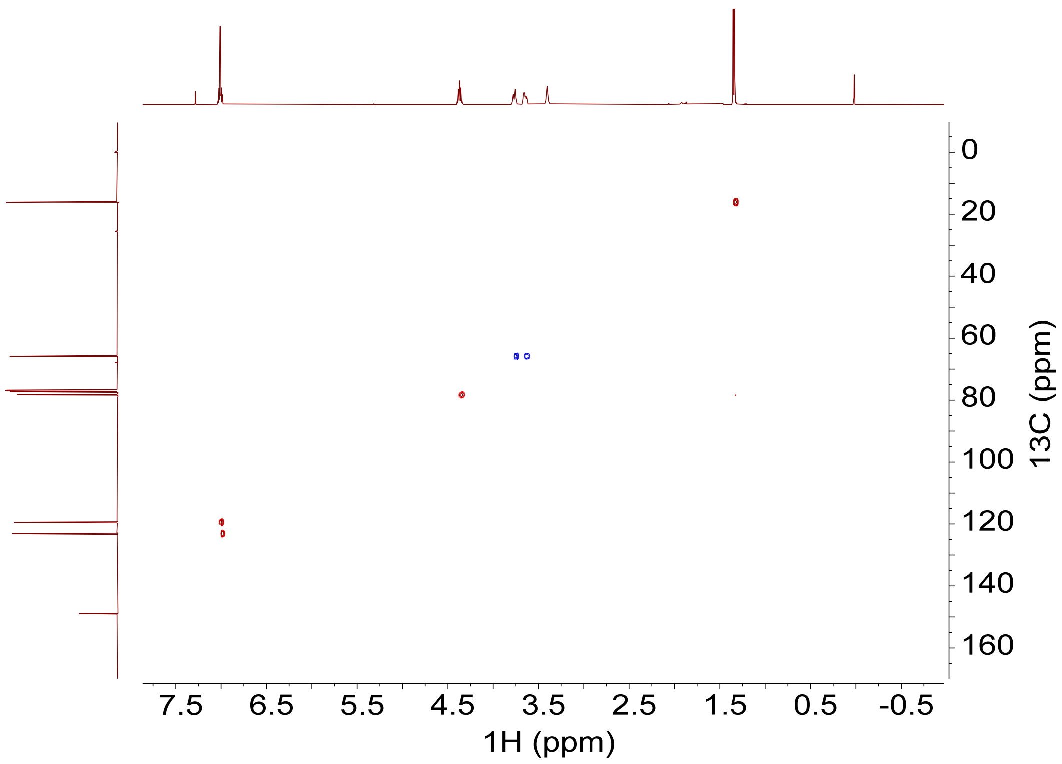

*Fig. S151. ^1H-^{13}C HSQC NMR spectrum of (S,S)-**8** (500 MHz, $CDCl_3$, 298 K).*

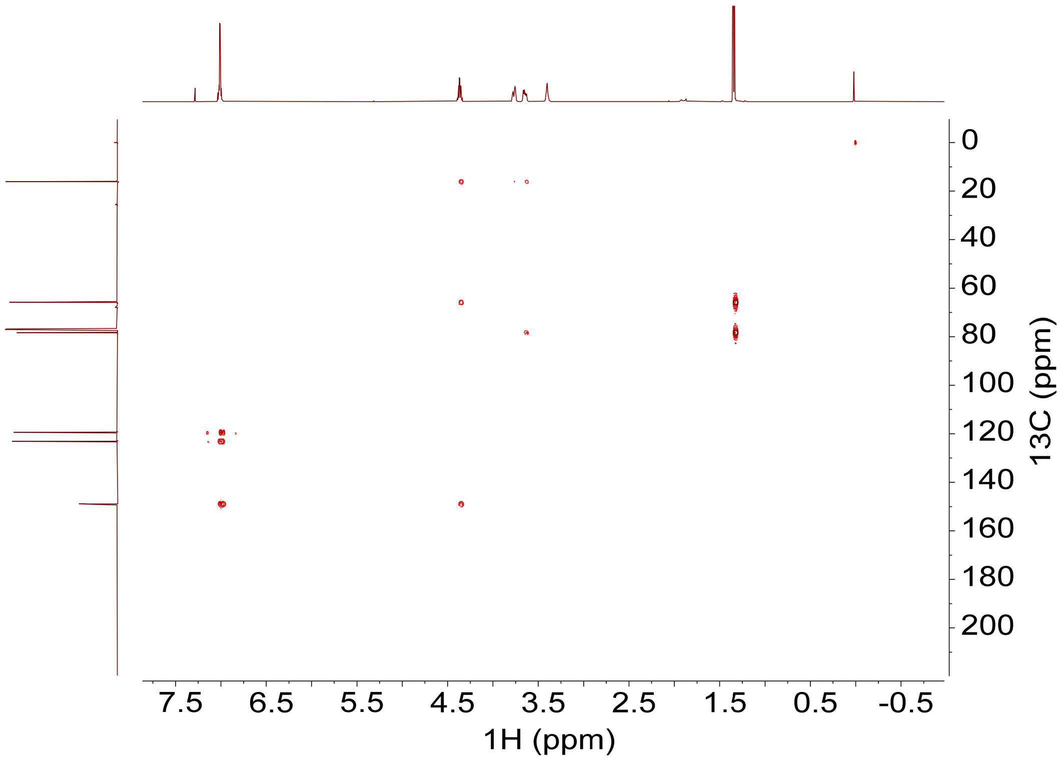

*Fig. S152. ^1H-^{13}C HMBC NMR spectrum of (S,S)-**8** (500 MHz, $CDCl_3$, 298 K).*

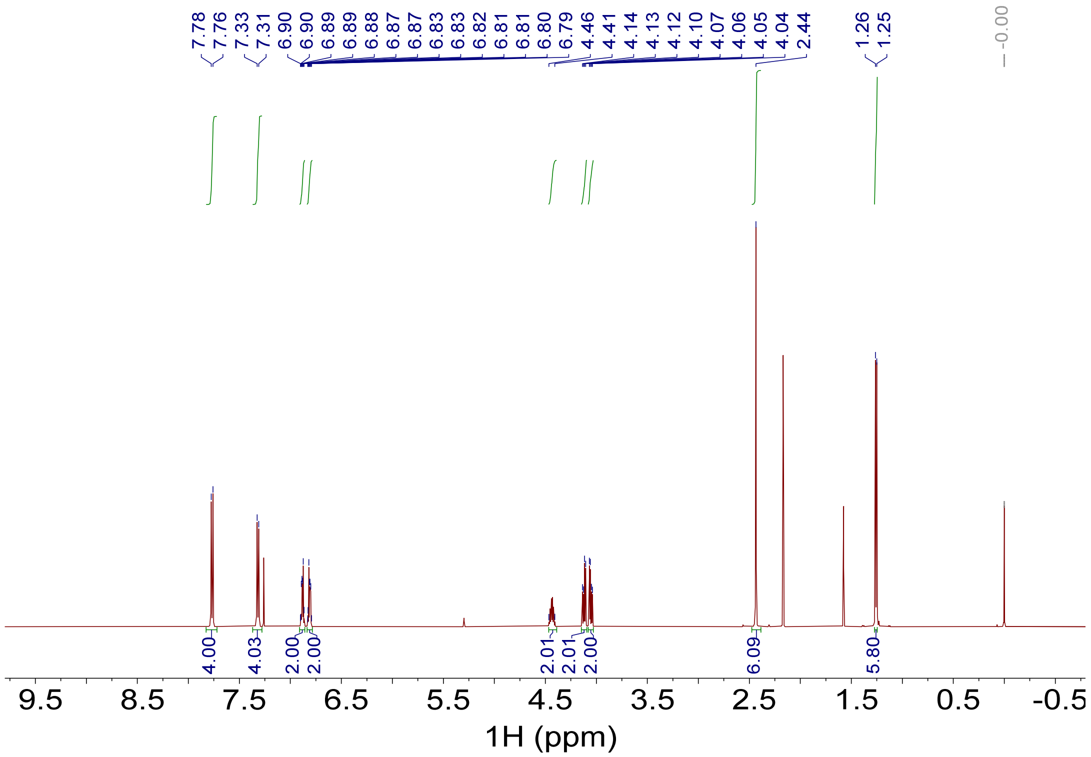

*Fig. S153. ^{1}H NMR spectrum of (R,R)-**9** (500 MHz, CDCl$_3$, 298 K).*

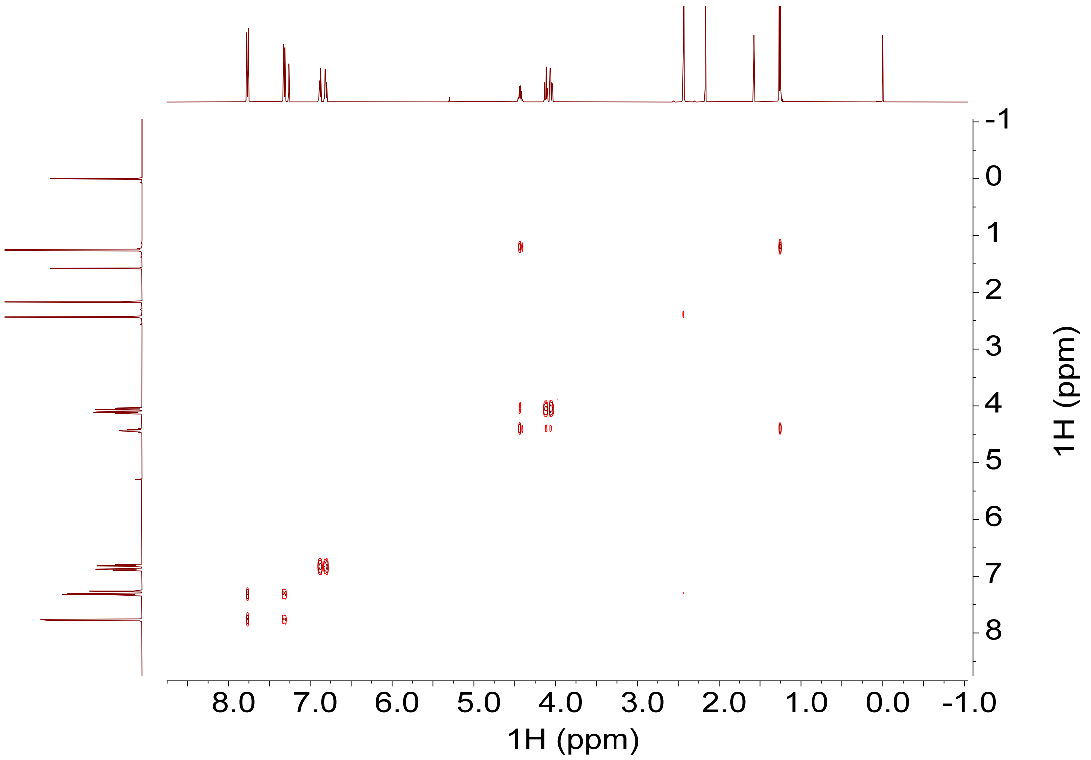

*Fig. S154. ^{1}H-^{1}H COSY NMR spectrum of (R,R)-**9** (500 MHz, CDCl$_3$, 298 K).*

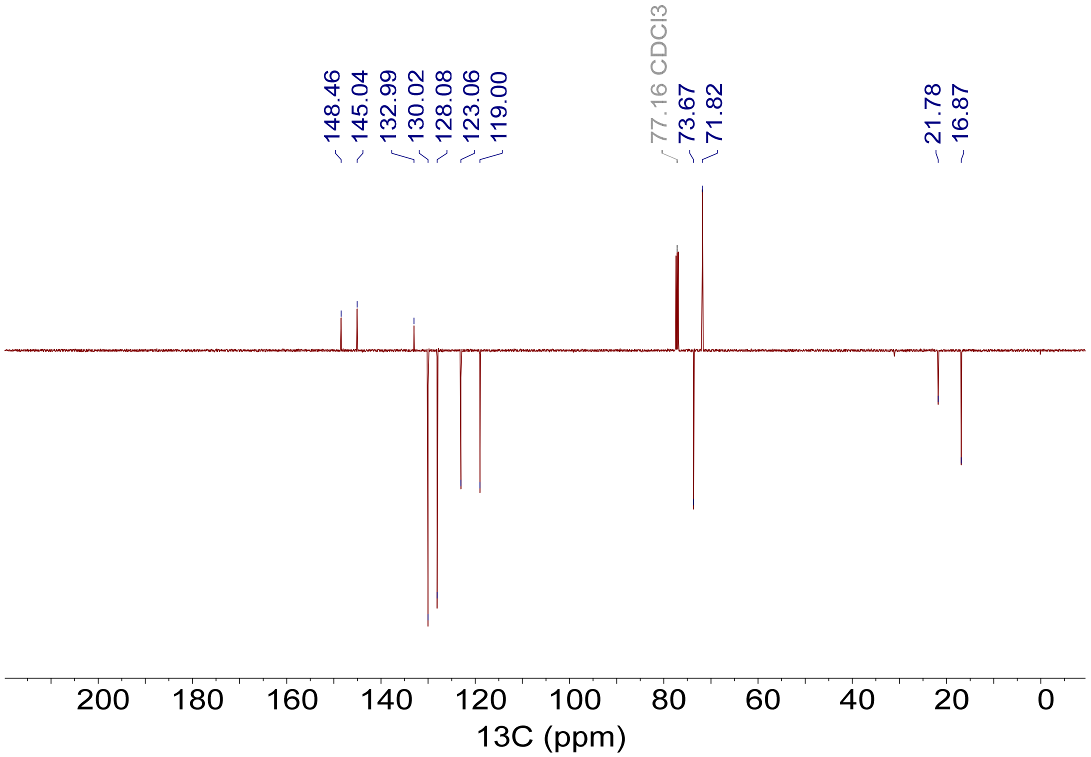

*Fig. S155. ^{13}C NMR spectrum of (R,R)-**9** (APT, 126 MHz, $CDCl_3$, 298 K).*

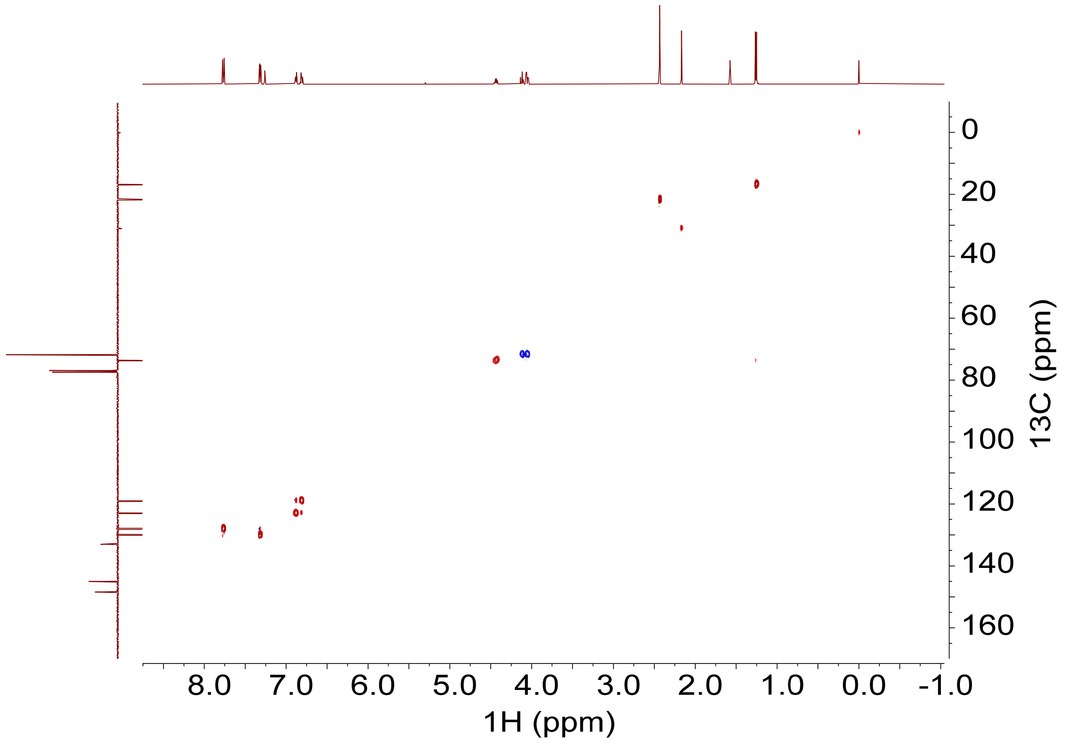

*Fig. S156. ^{1}H-^{13}C HSQC NMR spectrum of (R,R)-**9** (500 MHz, $CDCl_3$, 298 K).*

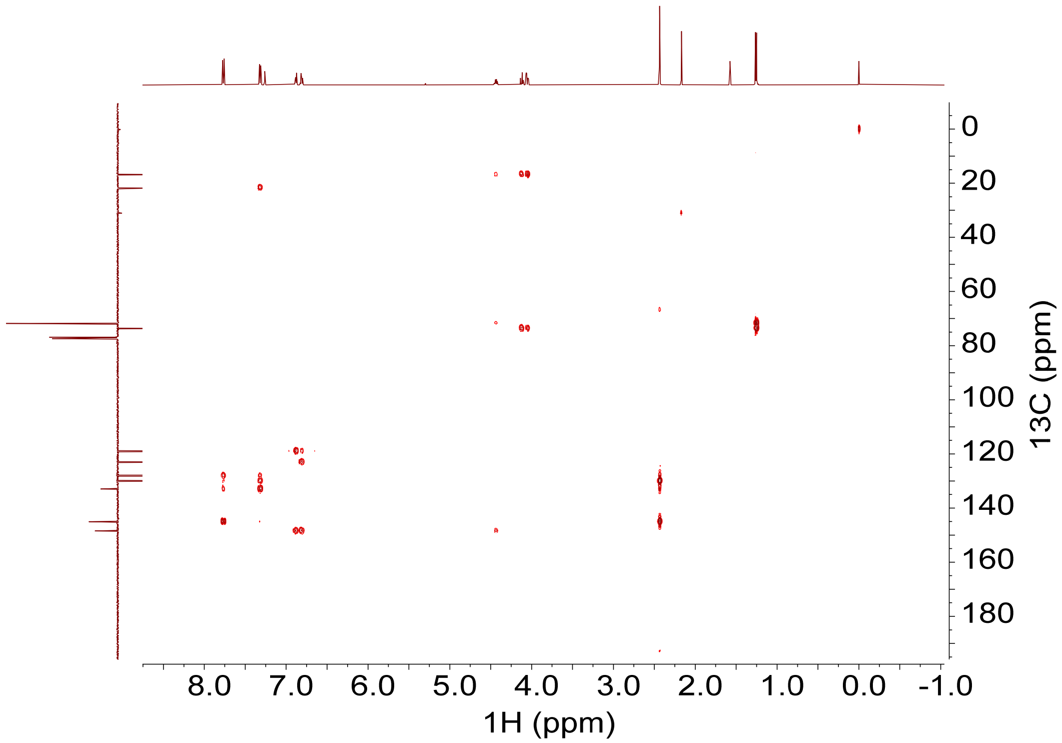

*Fig. S157. ^{1}H-^{13}C HMBC NMR spectrum of (R,R)-**9** (500 MHz, $CDCl_3$, 298 K).*

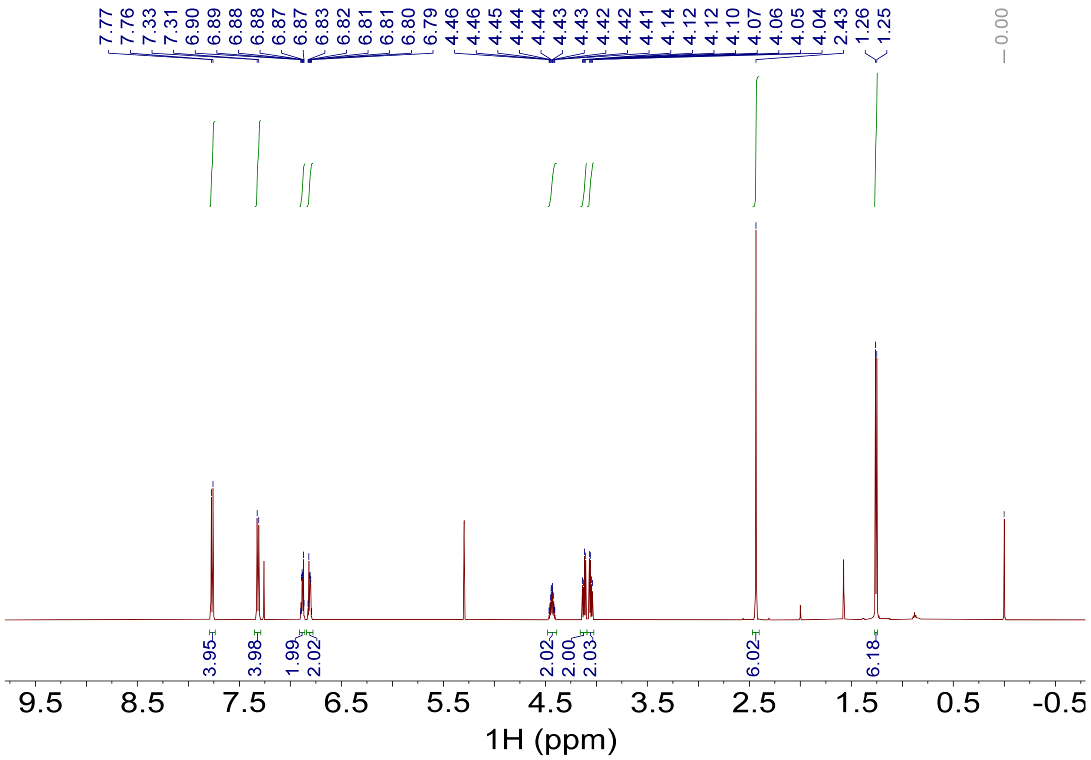

*Fig. S158. ^{1}H NMR spectrum of (S,S)-**9** (500 MHz, $CDCl_3$, 298 K).*

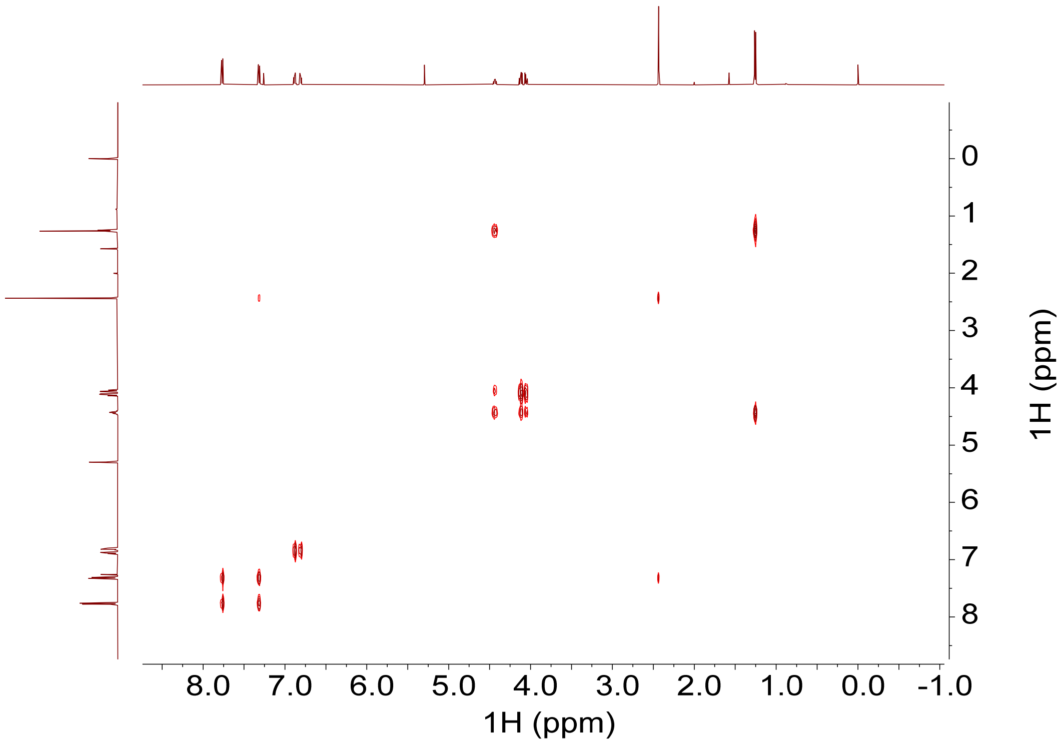

Fig. S159. [1]*H-*[1]*H COSY NMR spectrum of (S,S)-**9** (500 MHz, $CDCl_3$, 298 K).*

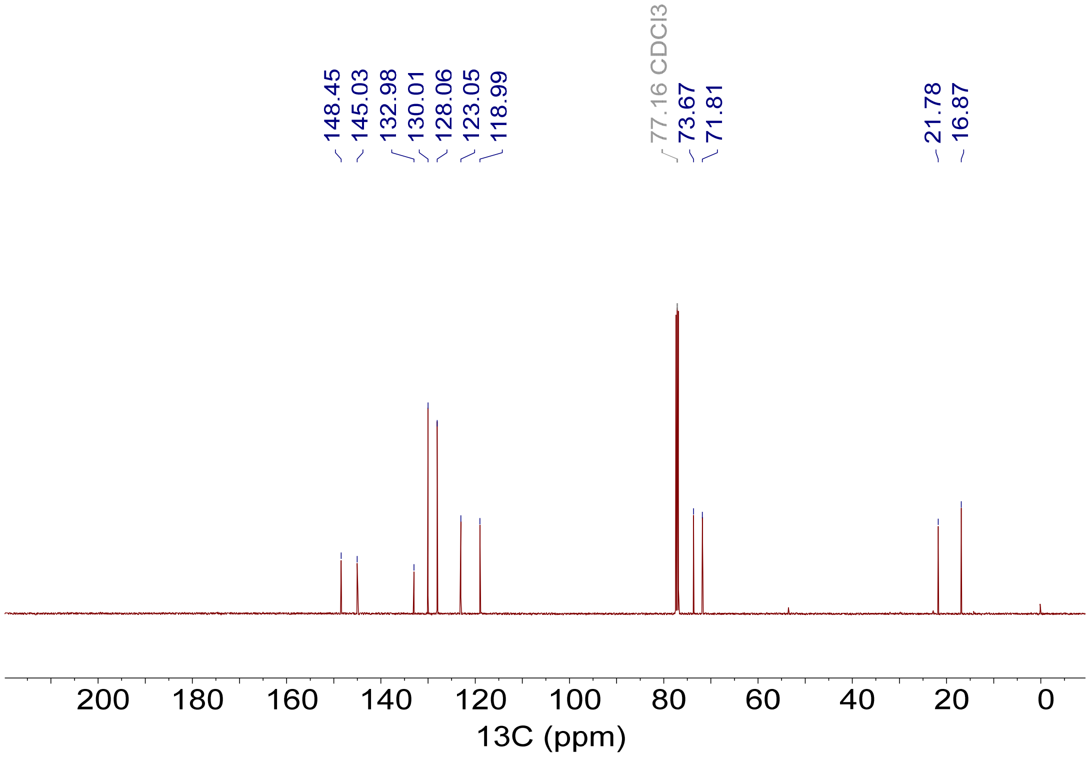

Fig. S160. [13]*C NMR spectrum of (S,S)-**9** (CPD, 126 MHz, $CDCl_3$, 298 K).*

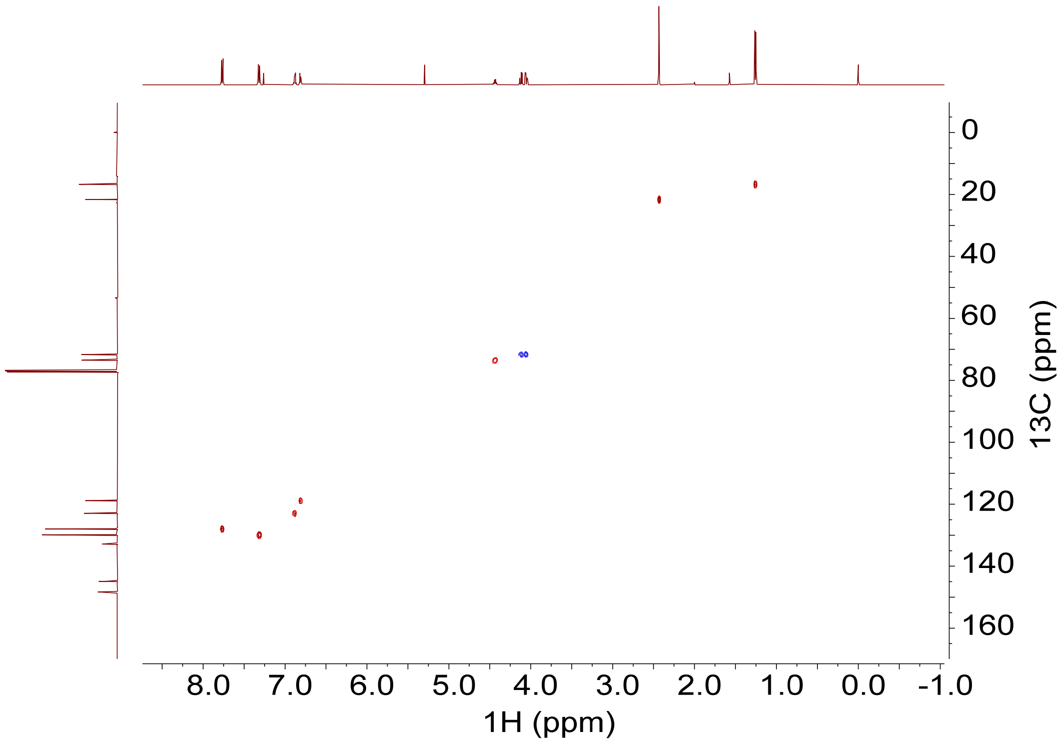

*Fig. S161. ^{1}H-^{13}C HSQC NMR spectrum of (S,S)-**9** (500 MHz, $CDCl_3$, 298 K).*

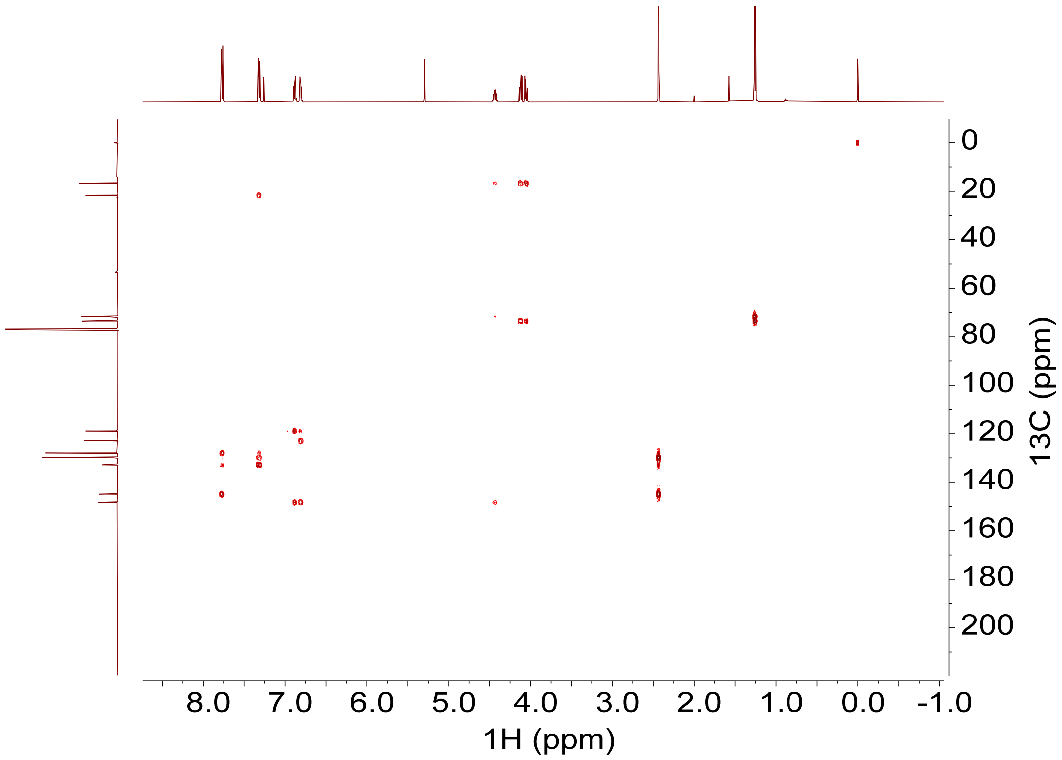

*Fig. S162. ^{1}H-^{13}C HMBC NMR spectrum of (S,S)-**9** (500 MHz, $CDCl_3$, 298 K).*

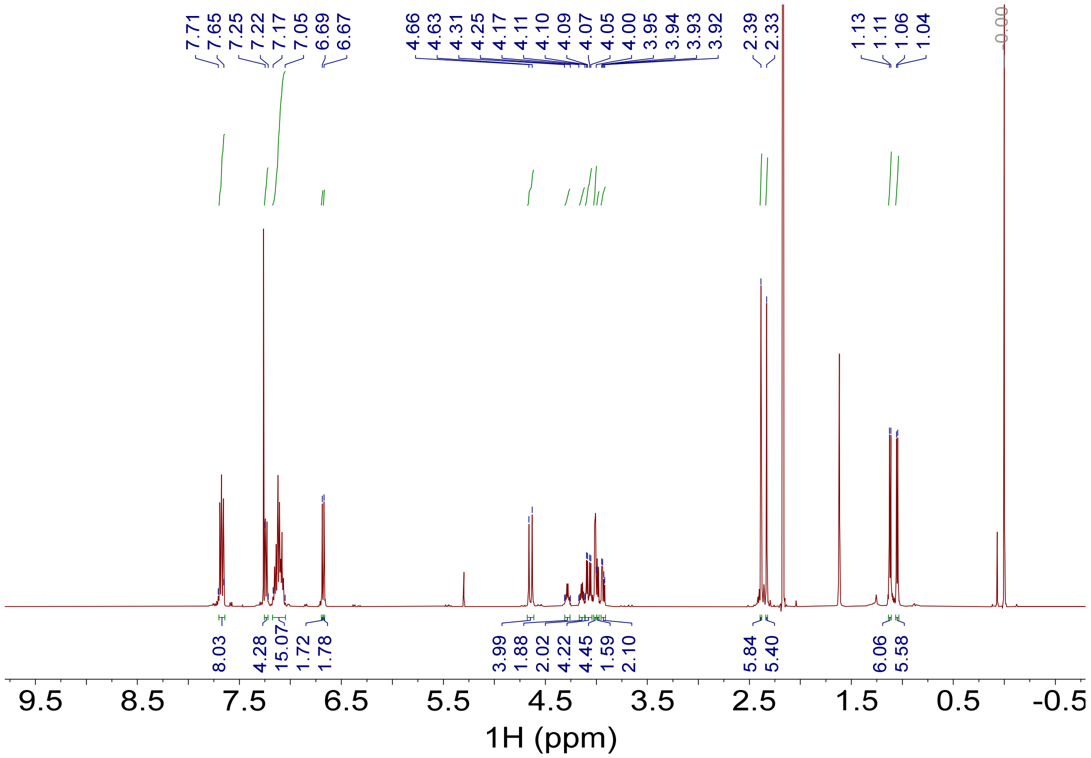

*Fig. S163. ^{1}H NMR spectrum of (R,R,R,R)-**11** (500 MHz, $CDCl_3$, 298 K).*

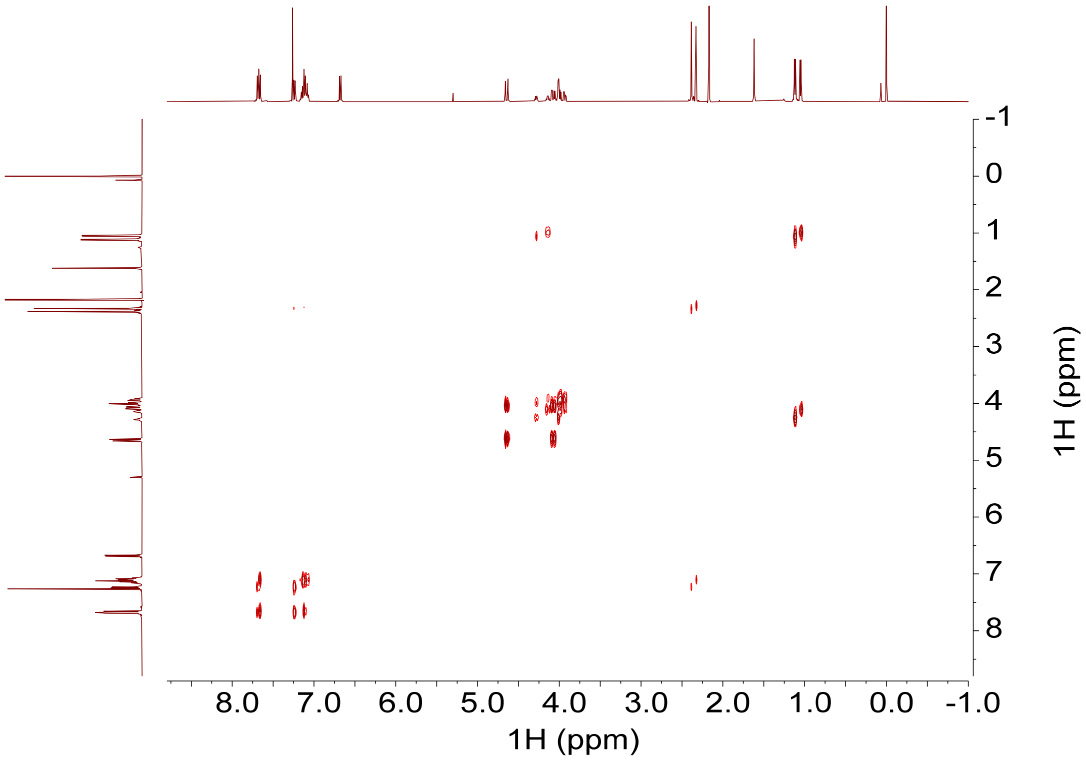

*Fig. S164. ^{1}H-^{1}H COSY NMR spectrum of (R,R,R,R)-**11** (500 MHz, $CDCl_3$, 298 K).*

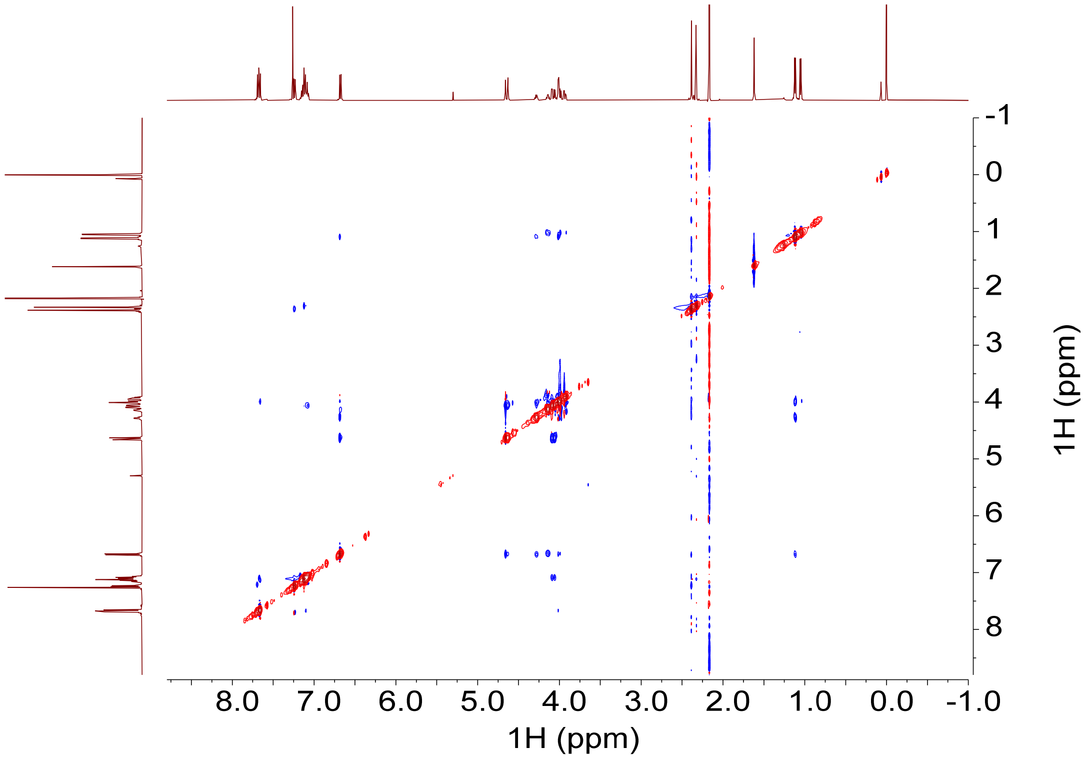

*Fig. S165. ^{1}H-^{1}H ROESY NMR spectrum of (R,R,R,R)-**11** (500 MHz, $CDCl_3$, 298 K).*

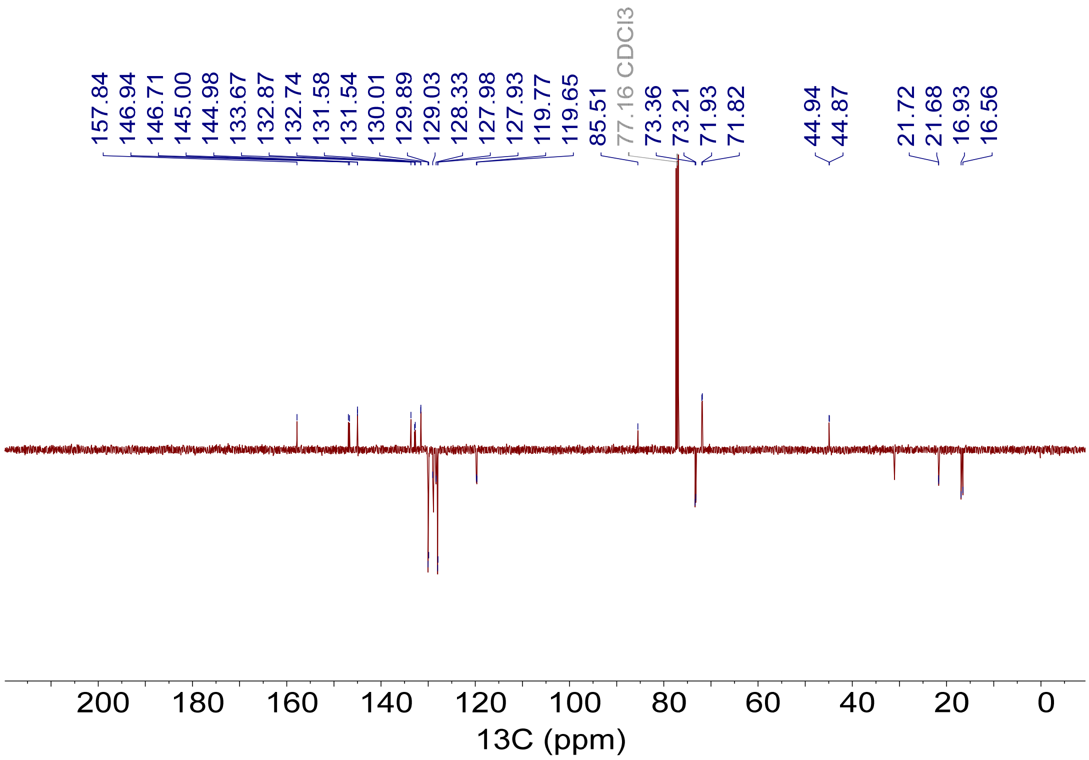

*Fig. S166. ^{13}C NMR spectrum of (R,R,R,R)-**11** (APT, 126 MHz, $CDCl_3$, 298 K).*

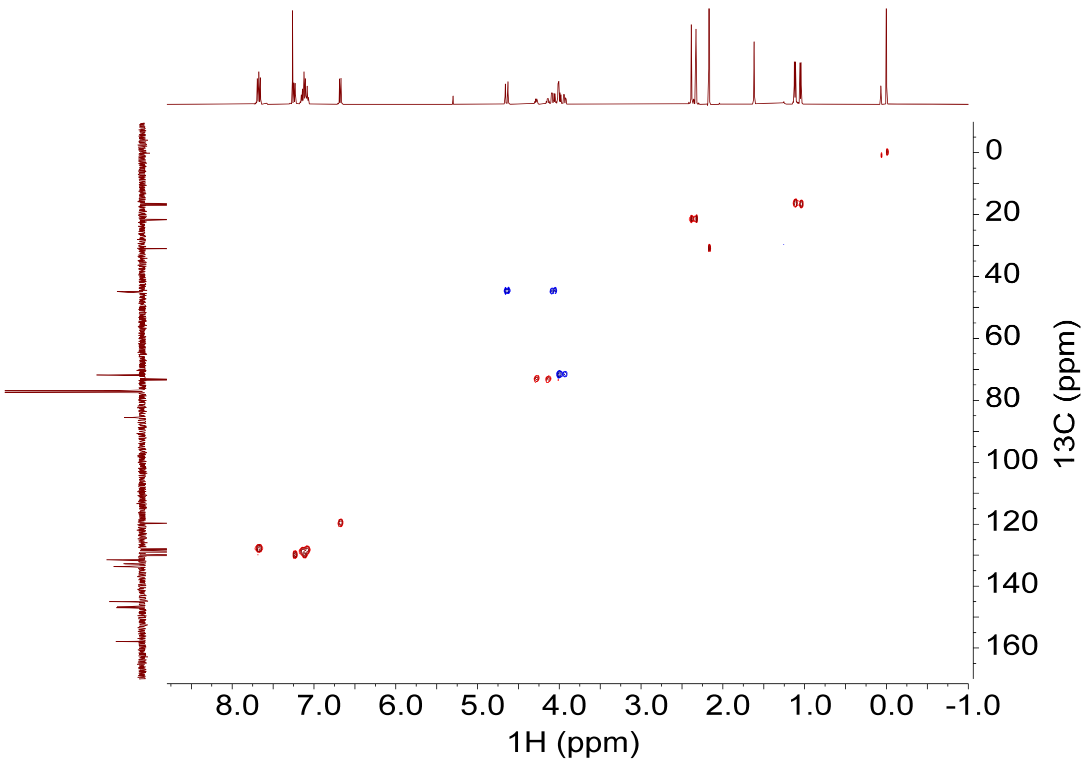

Fig. S167. *^{1}H-^{13}C HSQC NMR spectrum of (R,R,R,R)-**11** (500 MHz, $CDCl_3$, 298 K).*

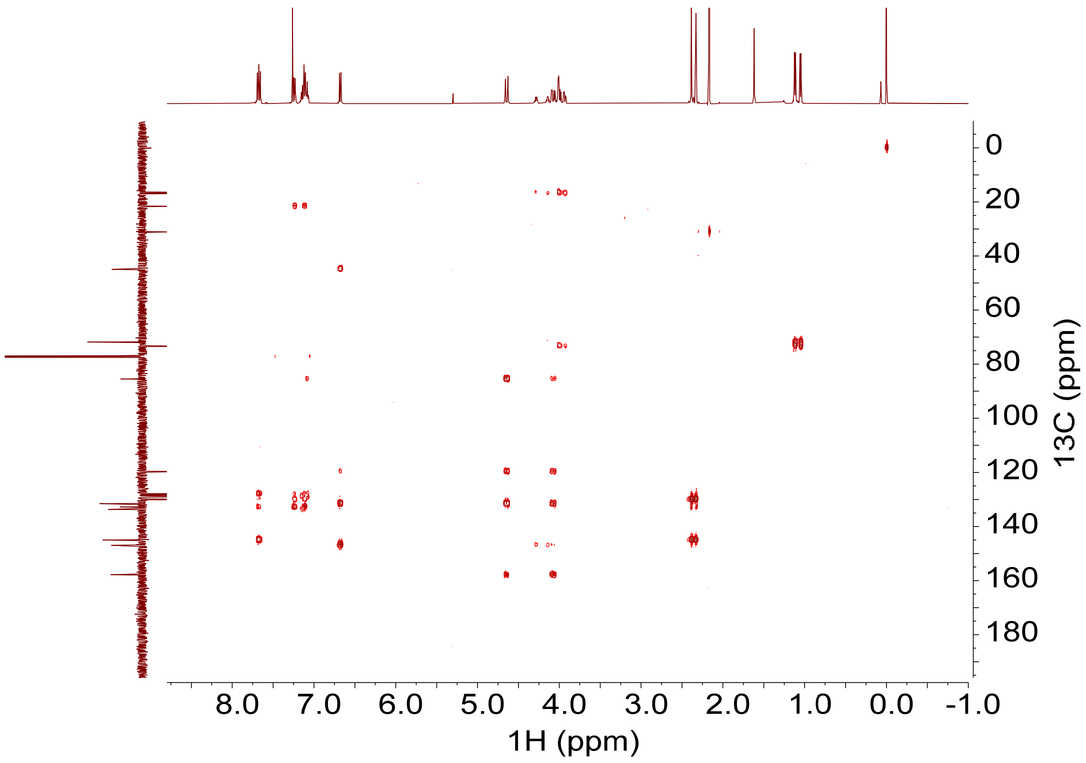

Fig. S168. *^{1}H-^{13}C HMBC NMR spectrum of (R,R,R,R)-**11** (500 MHz, $CDCl_3$, 298 K).*

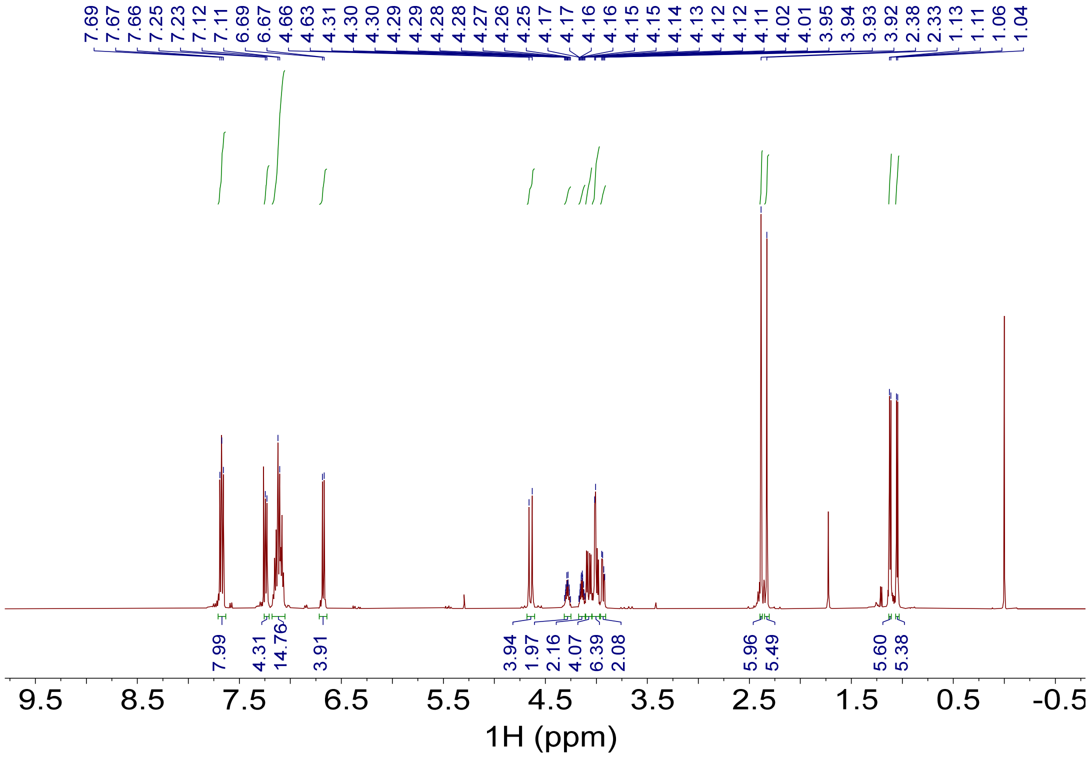

Fig. S169. [1]*H NMR spectrum of (S,S,S,S)-**11** (500 MHz, CDCl*$_3$*, 298 K).*

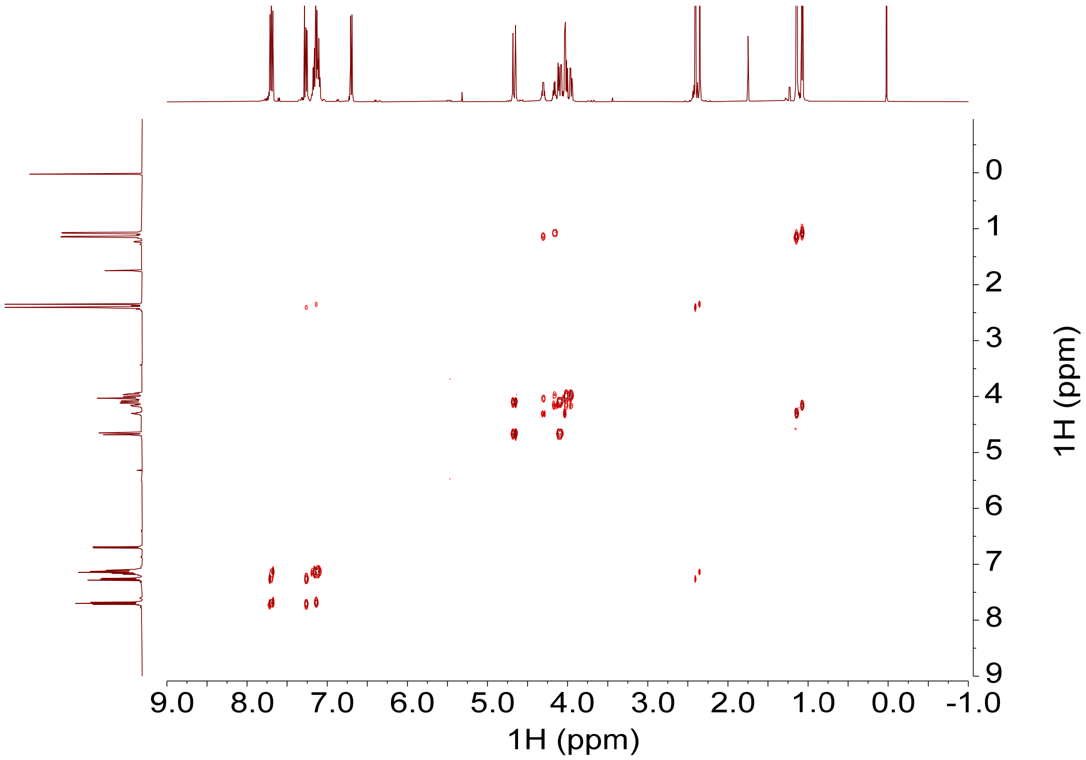

Fig. S170. [1]*H-*[1]*H COSY NMR spectrum of (S,S,S,S)-**11** (500 MHz, CDCl*$_3$*, 298 K).*

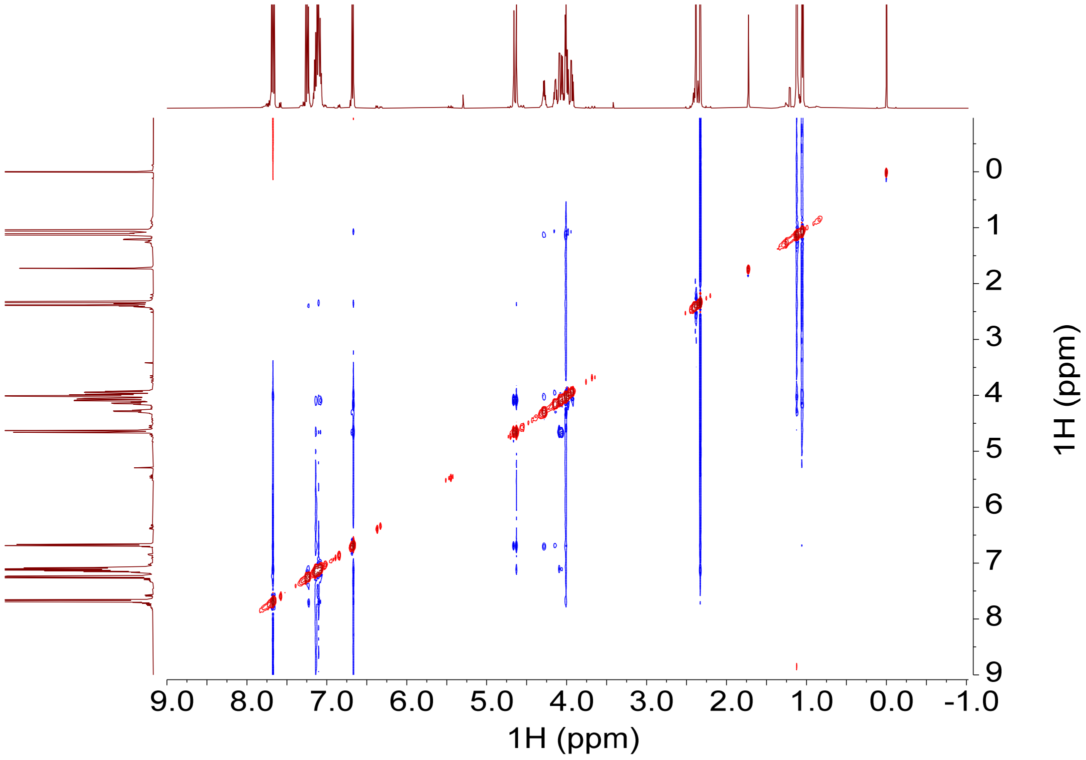

*Fig. S171. ^{1}H-^{1}H ROESY NMR spectrum of (S,S,S,S)-**11** (500 MHz, $CDCl_3$, 298 K).*

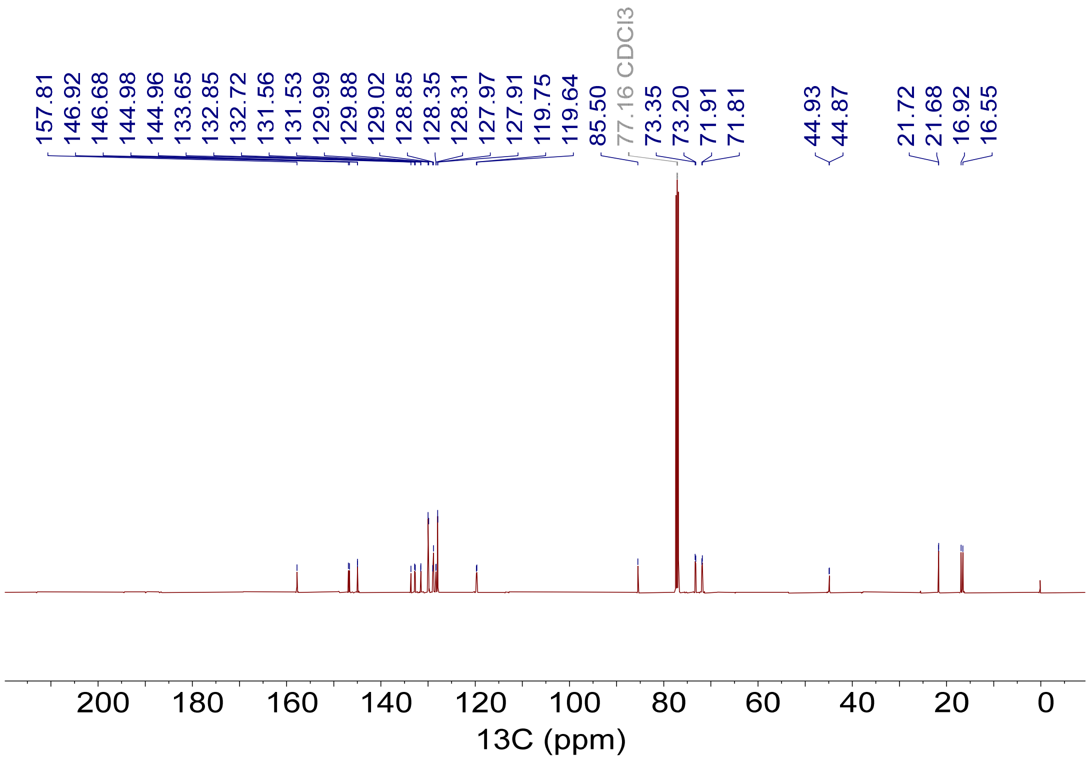

*Fig. S172. ^{13}C NMR spectrum of (S,S,S,S)-**11** (CPD, 126 MHz, $CDCl_3$, 298 K).*

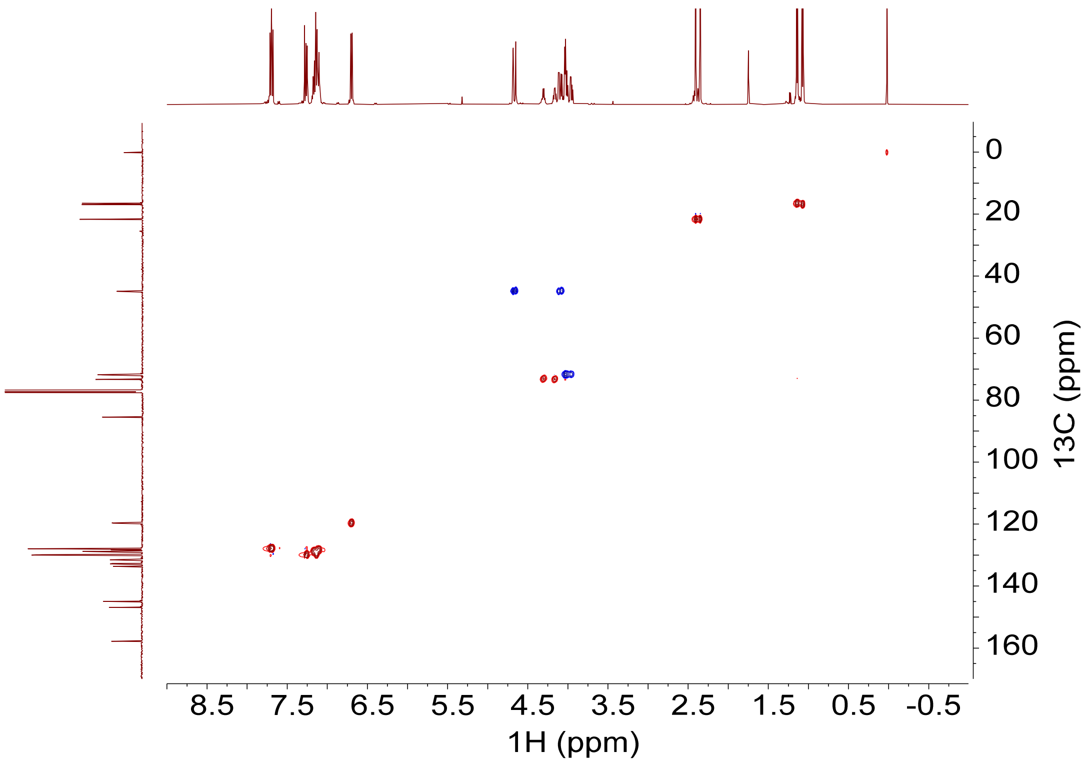

*Fig. S173. ^{1}H-^{13}C HSQC NMR spectrum of (S,S,S,S)-**11** (500 MHz, $CDCl_3$, 298 K).*

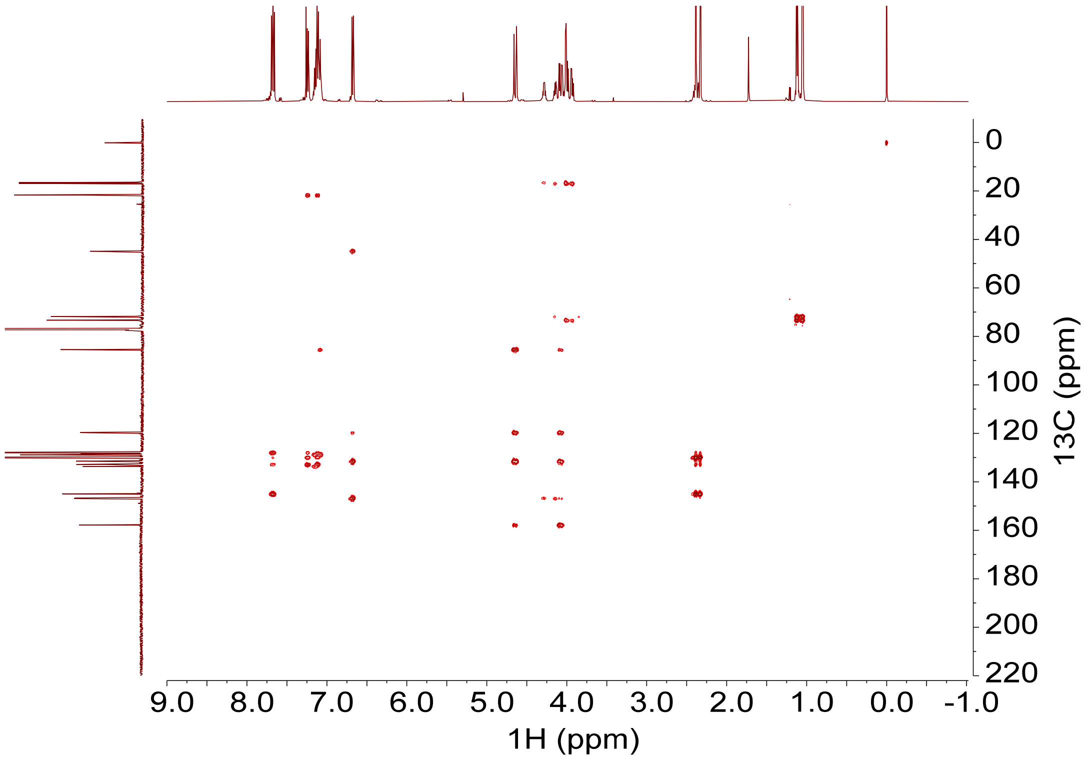

*Fig. S174. ^{1}H-^{13}C HMBC NMR spectrum of (S,S,S,S)-**11** (500 MHz, $CDCl_3$, 298 K).*

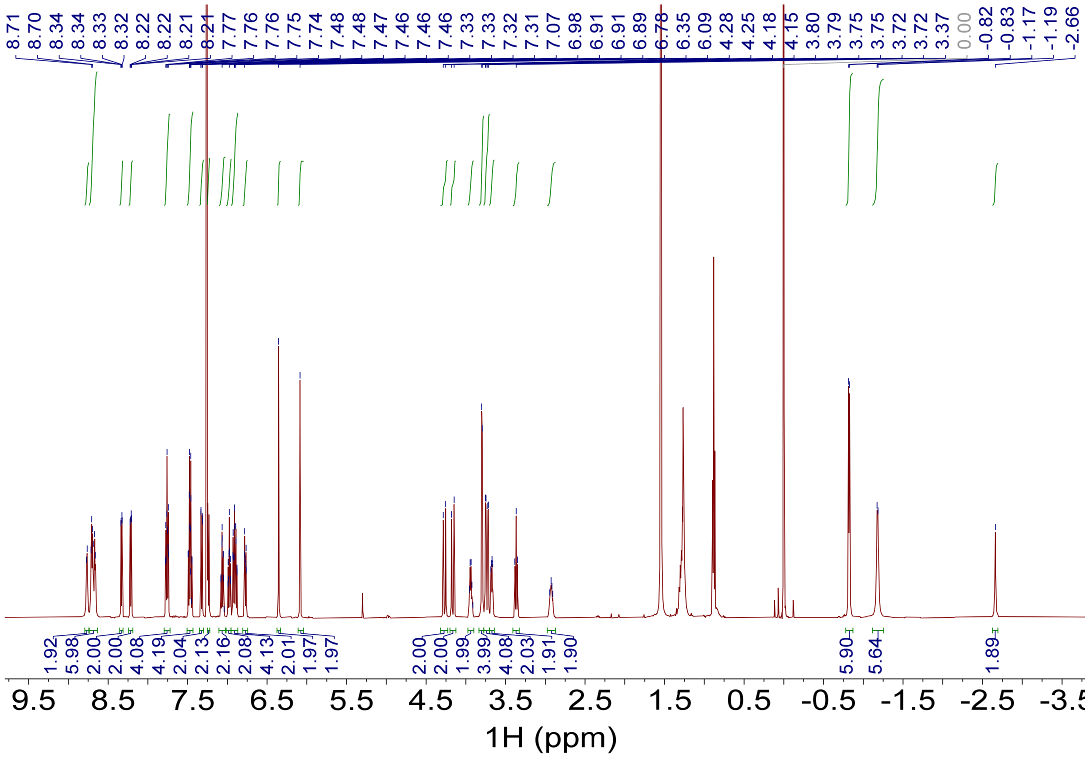

*Fig. S175. ^{1}H NMR spectrum of (R,R,R,R)-**H$_2$3** (500 MHz, $CDCl_3$, 298 K).*

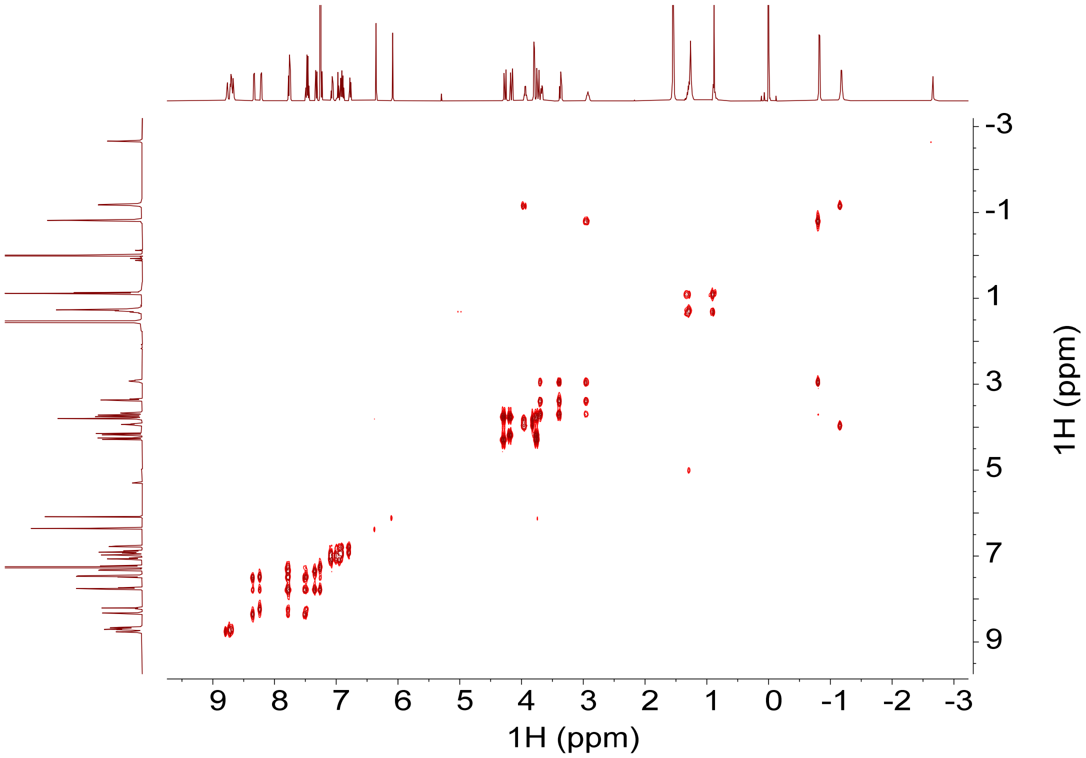

*Fig. S176. ^{1}H-^{1}H COSY NMR spectrum of (R,R,R,R)-**H$_2$3** (500 MHz, $CDCl_3$, 298 K).*

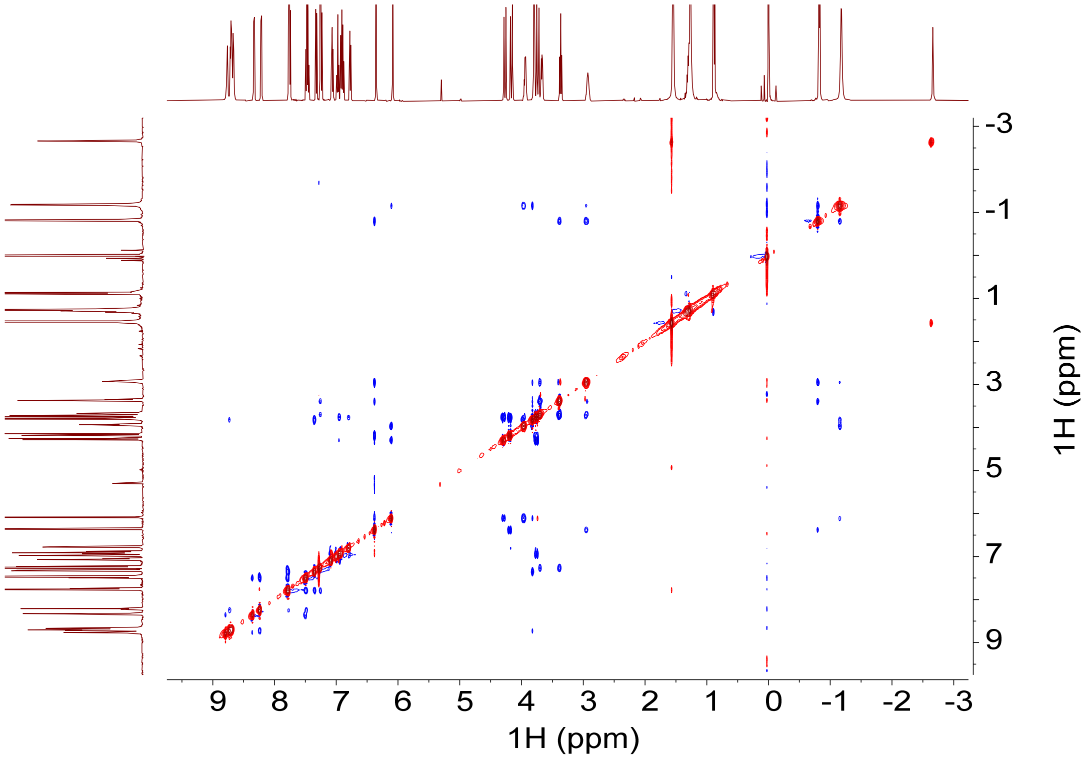

*Fig. S177. ^{1}H-^{1}H ROESY NMR spectrum of (R,R,R,R)-**$H_2$3** (500 MHz, $CDCl_3$, 298 K).*

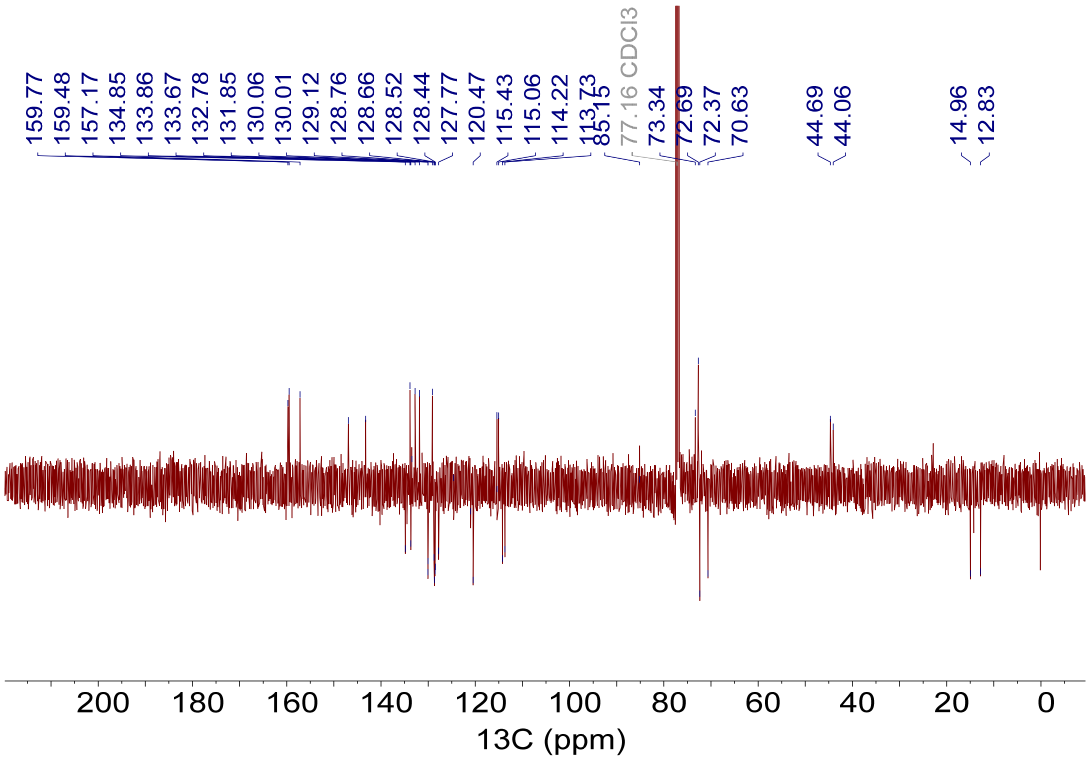

*Fig. S178. ^{13}C NMR spectrum of (R,R,R,R)-**$H_2$3** (APT, 126 MHz, $CDCl_3$, 298 K).*

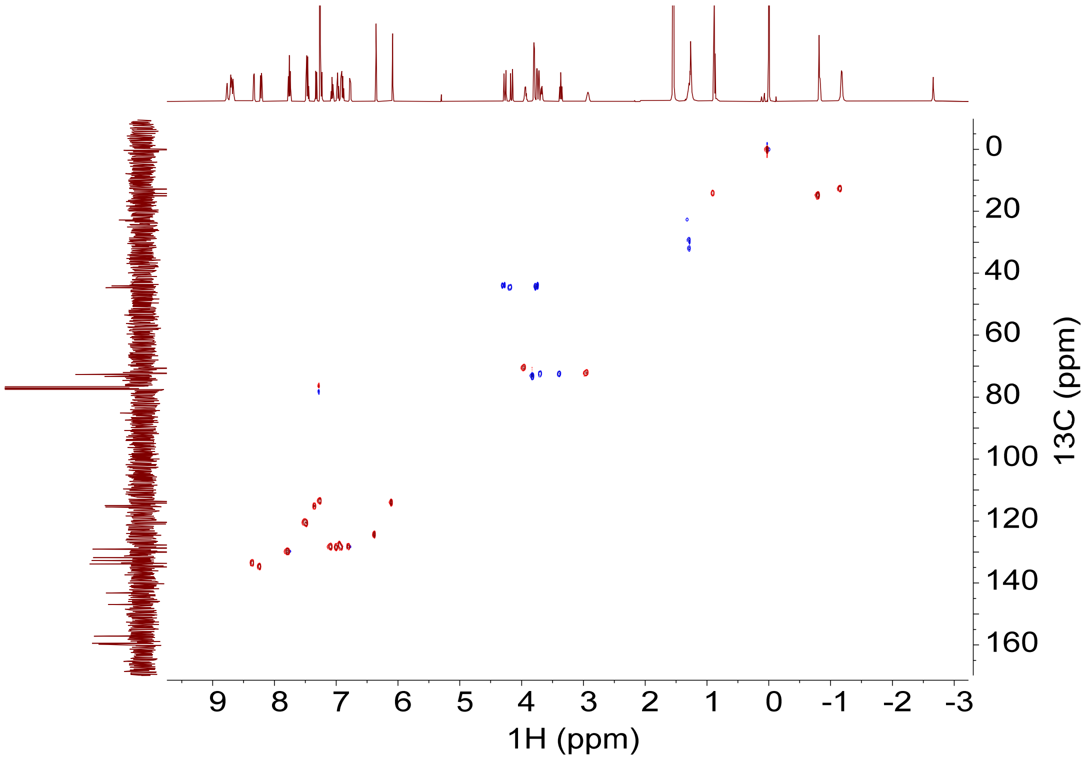

*Fig. S179. ^{1}H-^{13}C HSQC NMR spectrum of (R,R,R,R)-**$H_2$3** (500 MHz, $CDCl_3$, 298 K).*

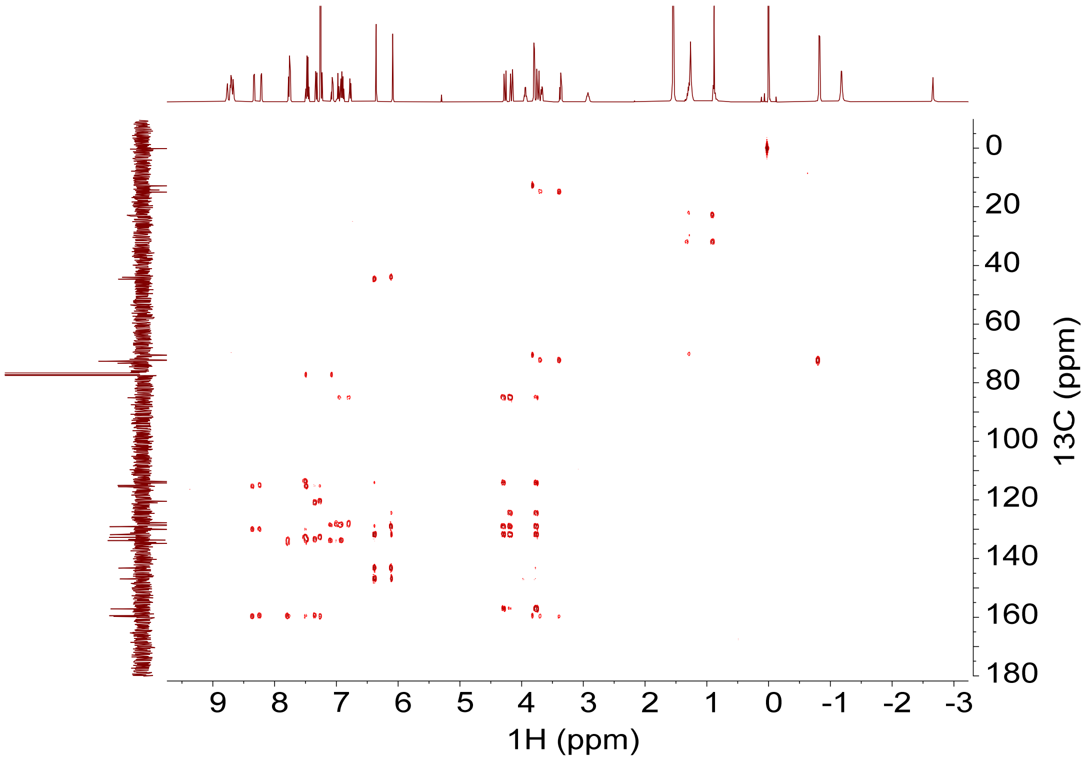

*Fig. S180. ^{1}H-^{13}C HMBC NMR spectrum of (R,R,R,R)-**$H_2$3** (500 MHz, $CDCl_3$, 298 K).*

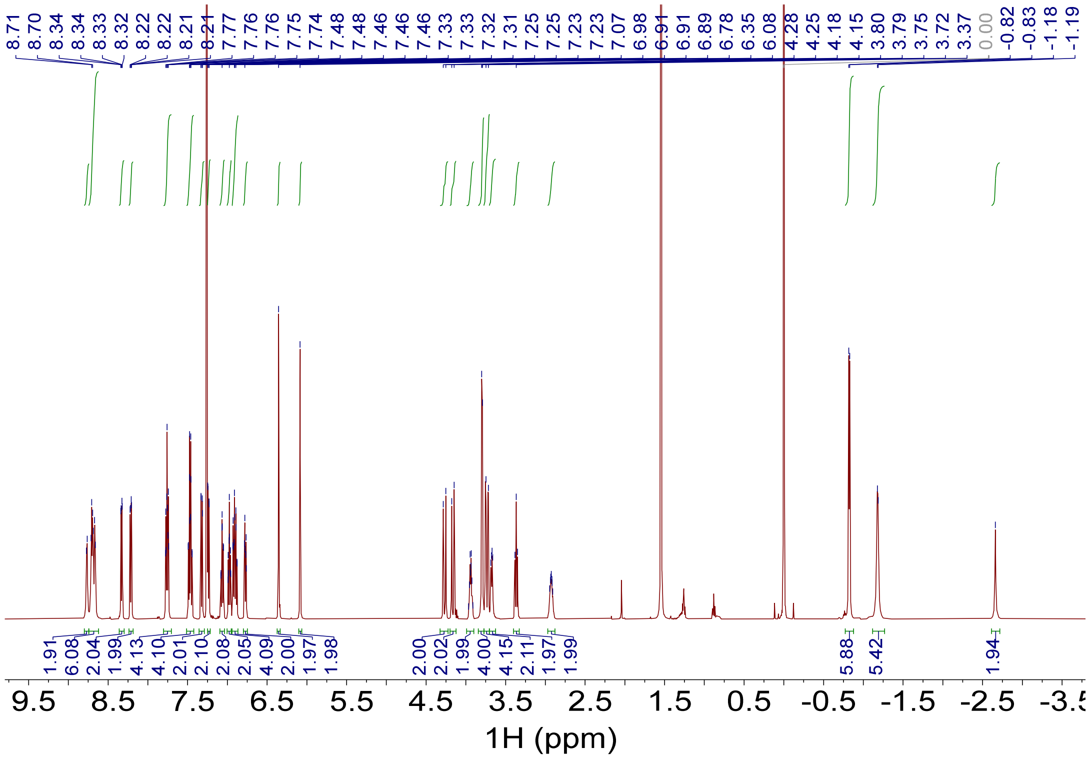

Fig. S181. *^{1}H NMR spectrum of (S,S,S,S)-**$H_2$3** (500 MHz, $CDCl_3$, 298 K).*

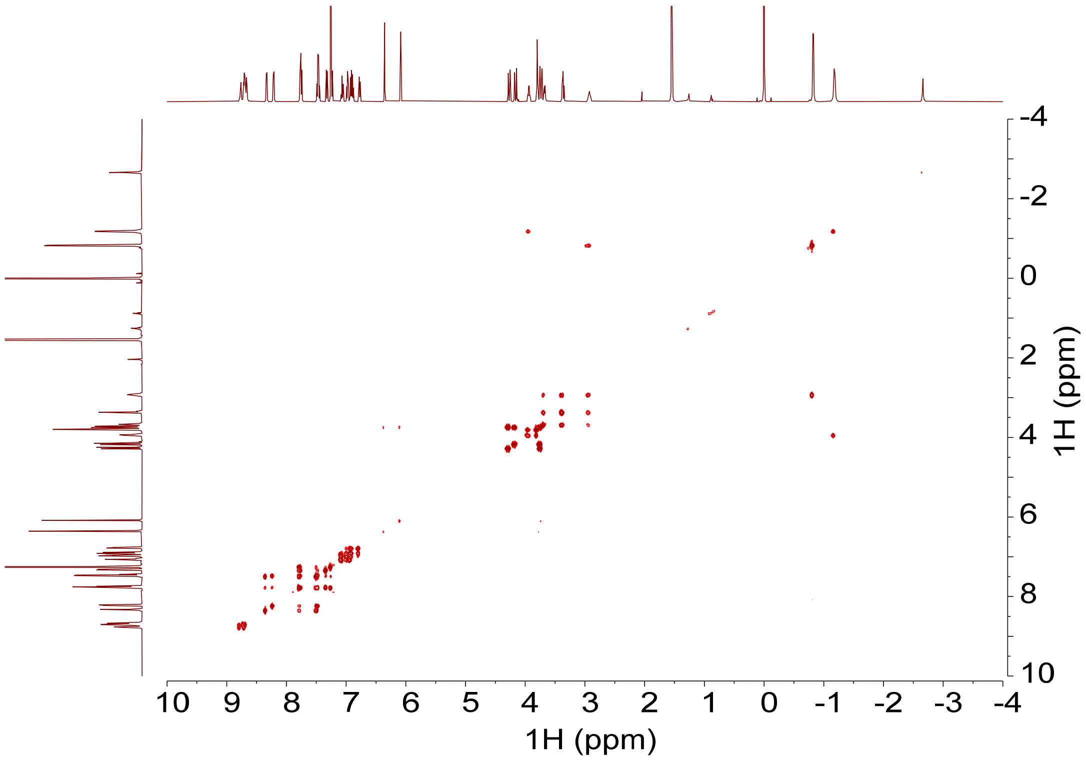

Fig. S182. *^{1}H-^{1}H COSY NMR spectrum of (S,S,S,S)-**$H_2$3** (500 MHz, $CDCl_3$, 298 K).*

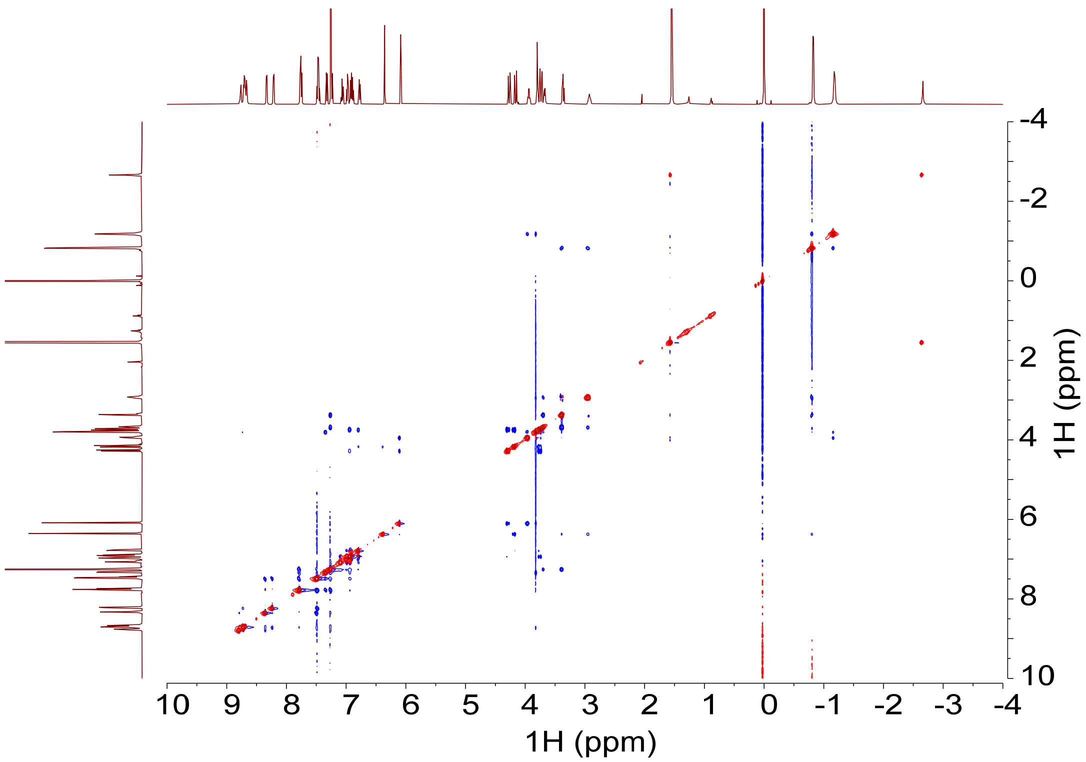

*Fig. S183. ^{1}H-^{1}H ROESY NMR spectrum of (S,S,S,S)-**$H_2$3** (500 MHz, $CDCl_3$, 298 K).*

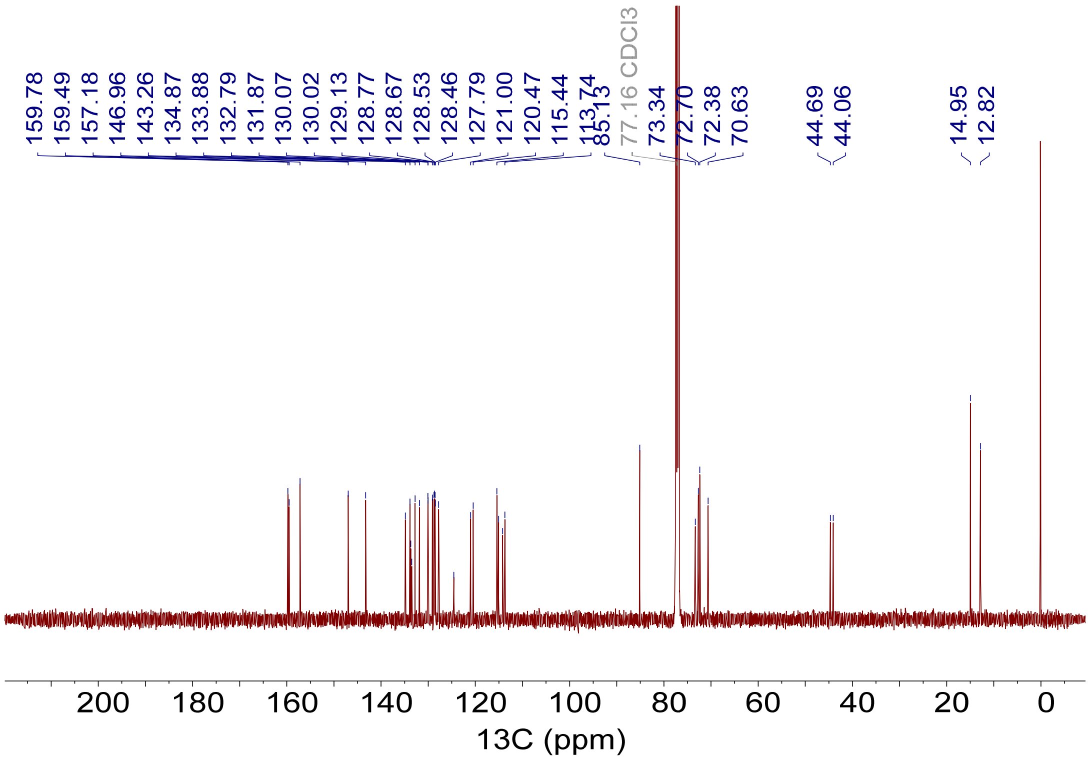

*Fig. S184. ^{13}C NMR spectrum of (S,S,S,S)-**$H_2$3** (CPD, 126 MHz, $CDCl_3$, 298 K).*

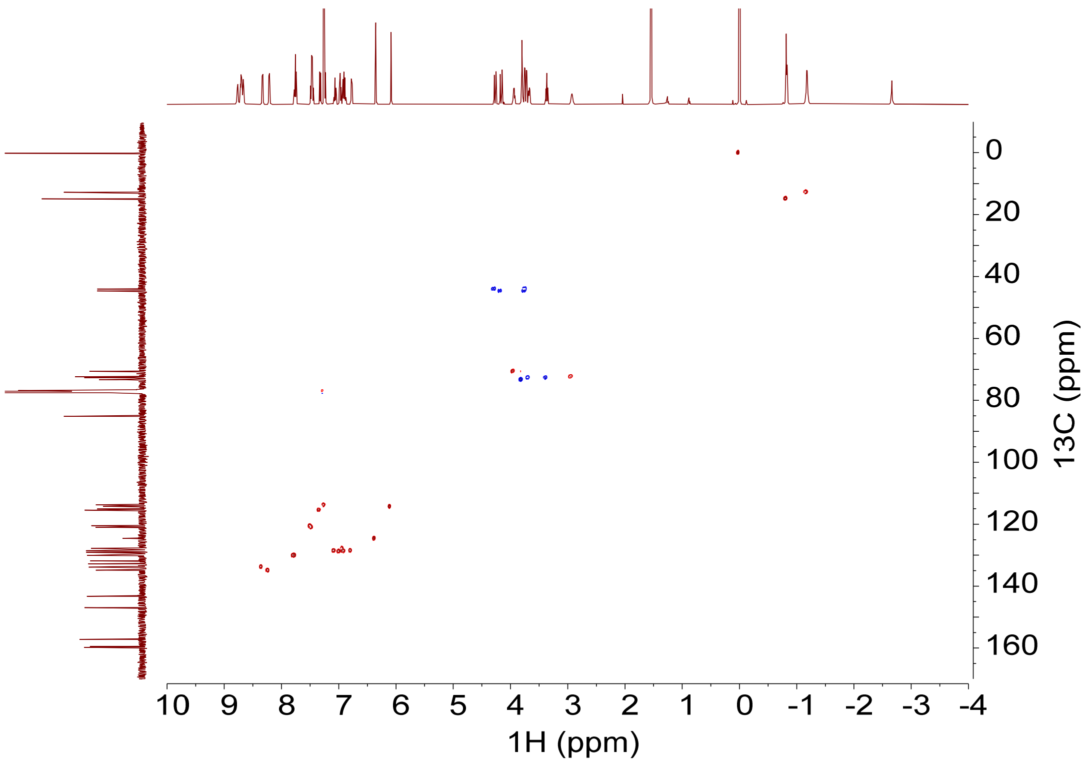

*Fig. S185. ^{1}H-^{13}C HSQC NMR spectrum of (S,S,S,S)-**H$_2$3** (500 MHz, $CDCl_3$, 298 K).*

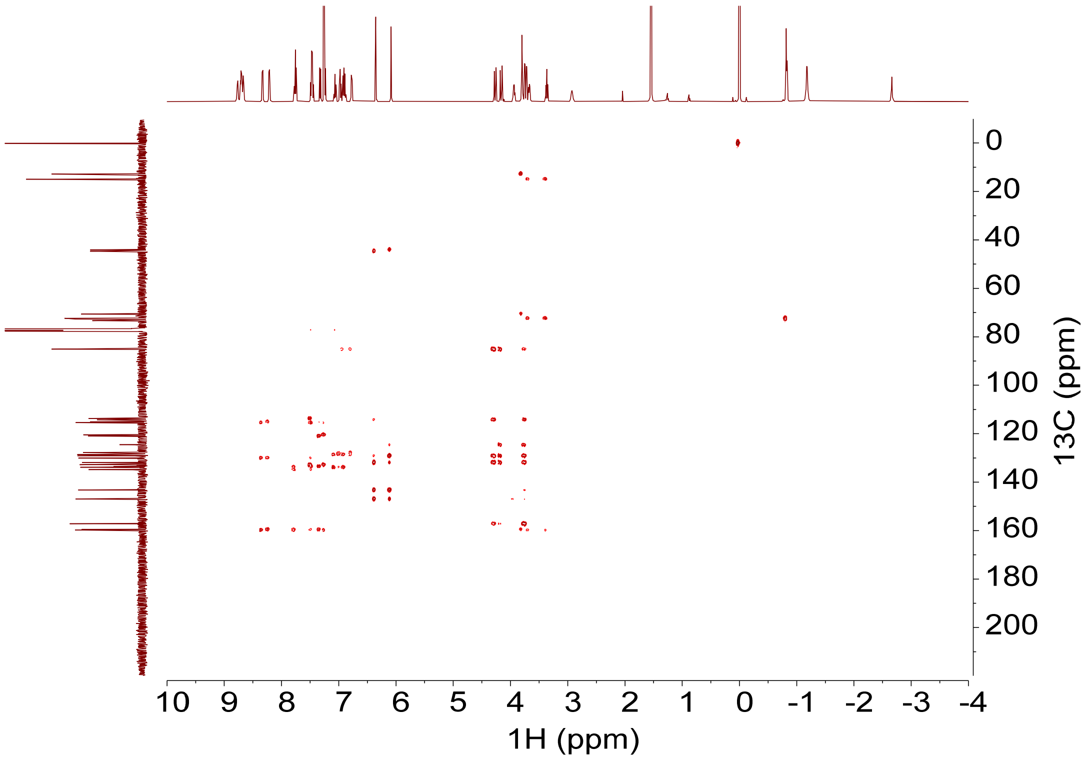

*Fig. S186. ^{1}H-^{13}C HMBC NMR spectrum of (S,S,S,S)-**H$_2$3** (500 MHz, $CDCl_3$, 298 K).*

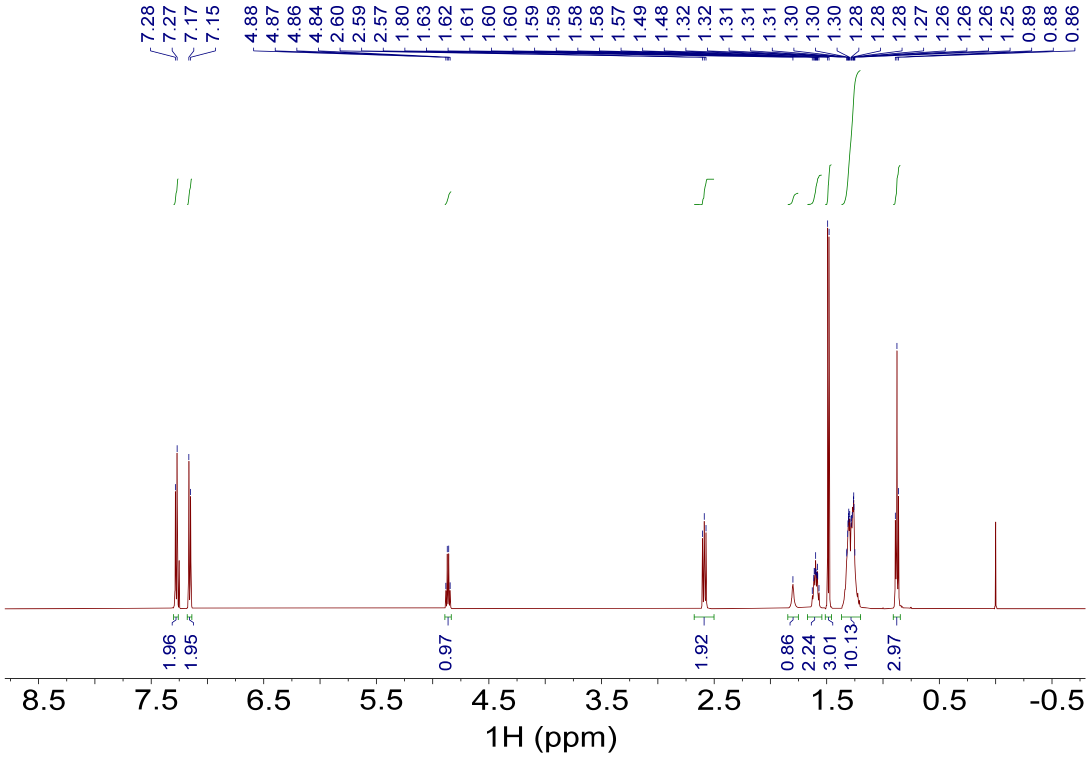

*Fig. S187. ^{1}H NMR spectrum of **21** (500 MHz, $CDCl_3$, 298 K).*

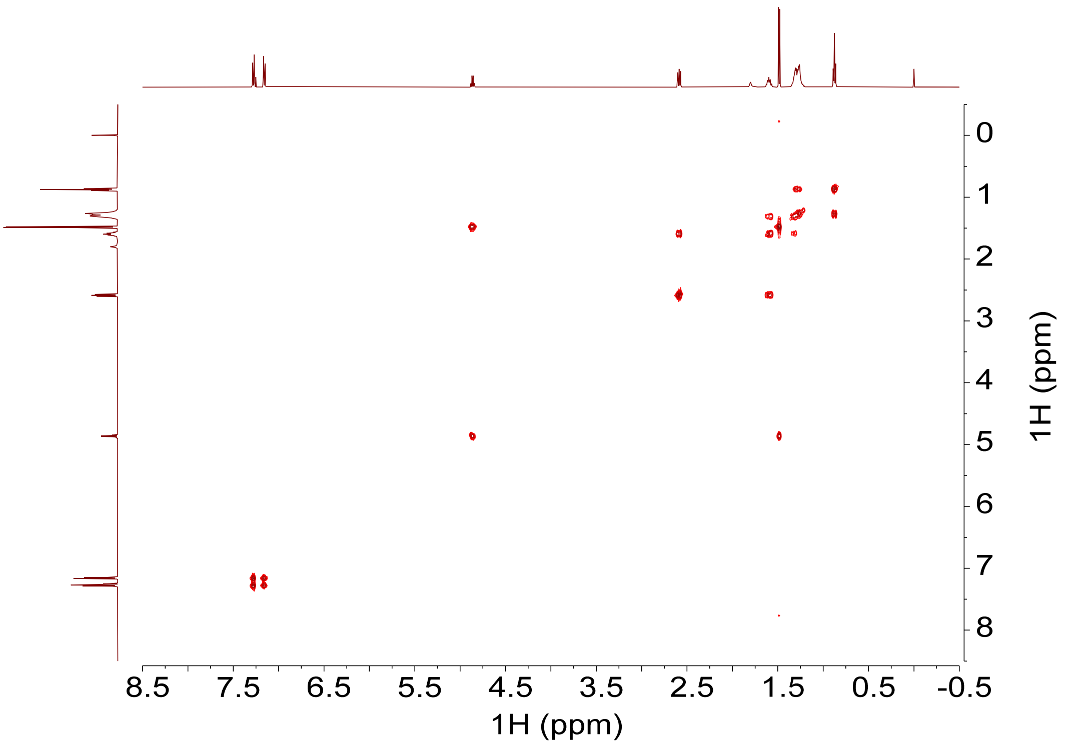

*Fig. S188. ^{1}H-^{1}H COSY NMR spectrum of **21** (500 MHz, $CDCl_3$, 298 K).*

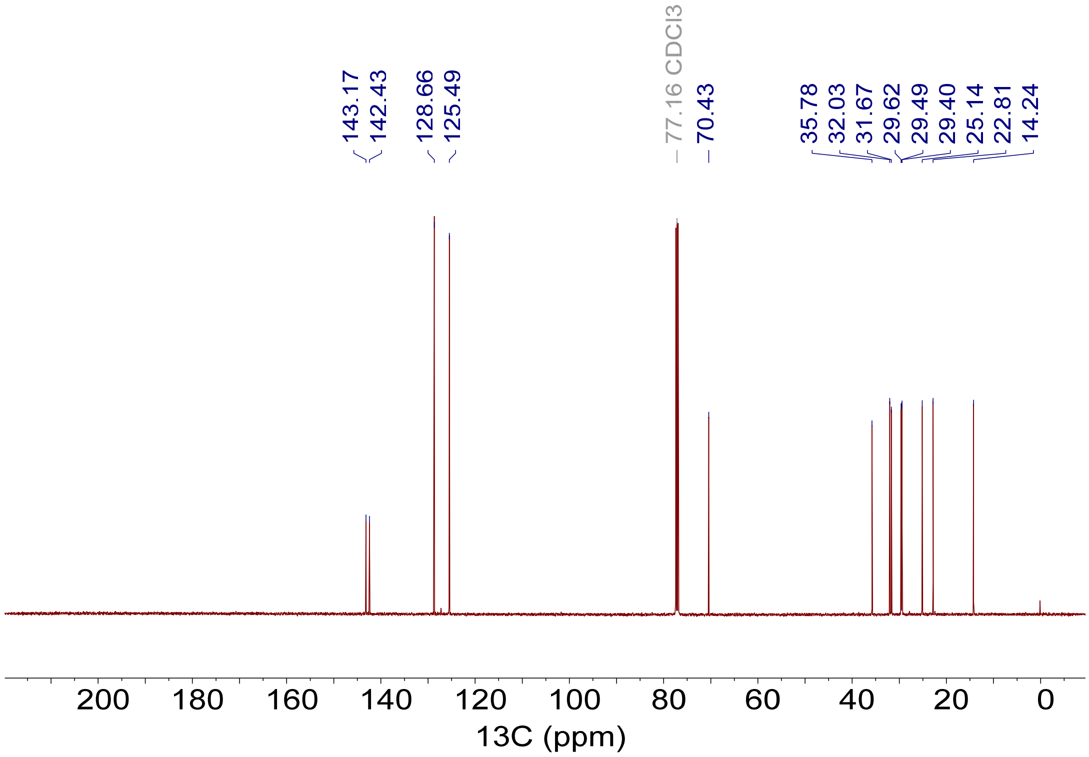

Fig. S189. ^{13}C NMR spectrum of ***21*** *(CPD, 126 MHz, $CDCl_3$, 298 K).*

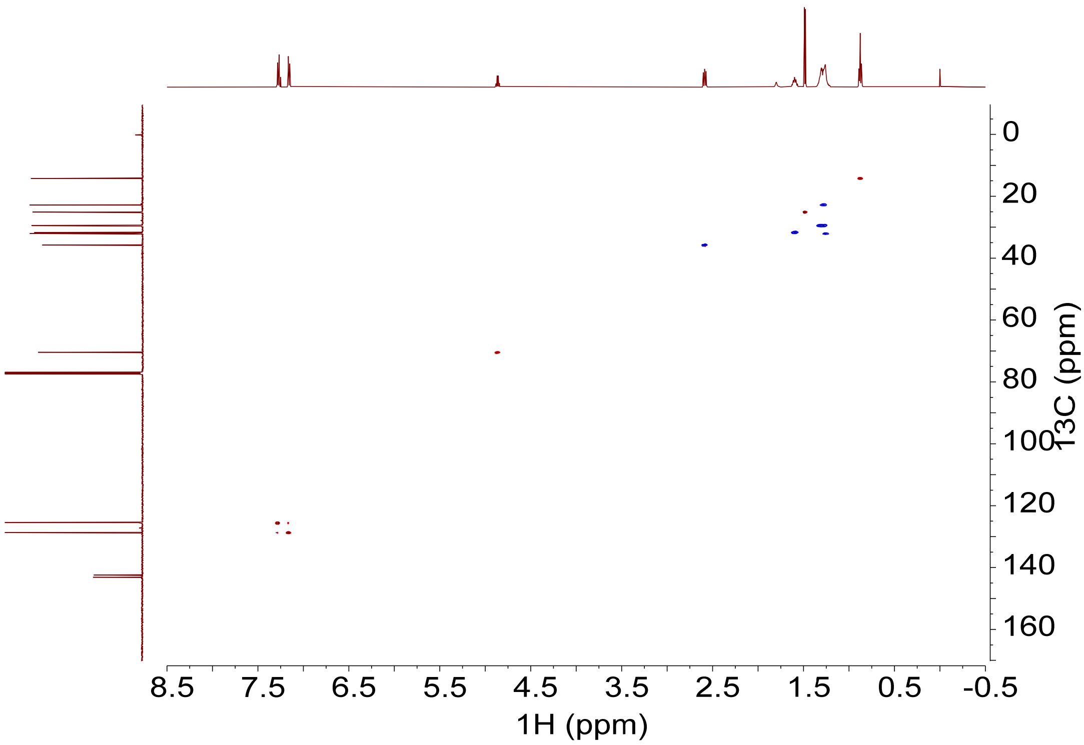

Fig. S190. ^{1}H-^{13}C HSQC NMR spectrum of ***21*** *(500 MHz, $CDCl_3$, 298 K).*

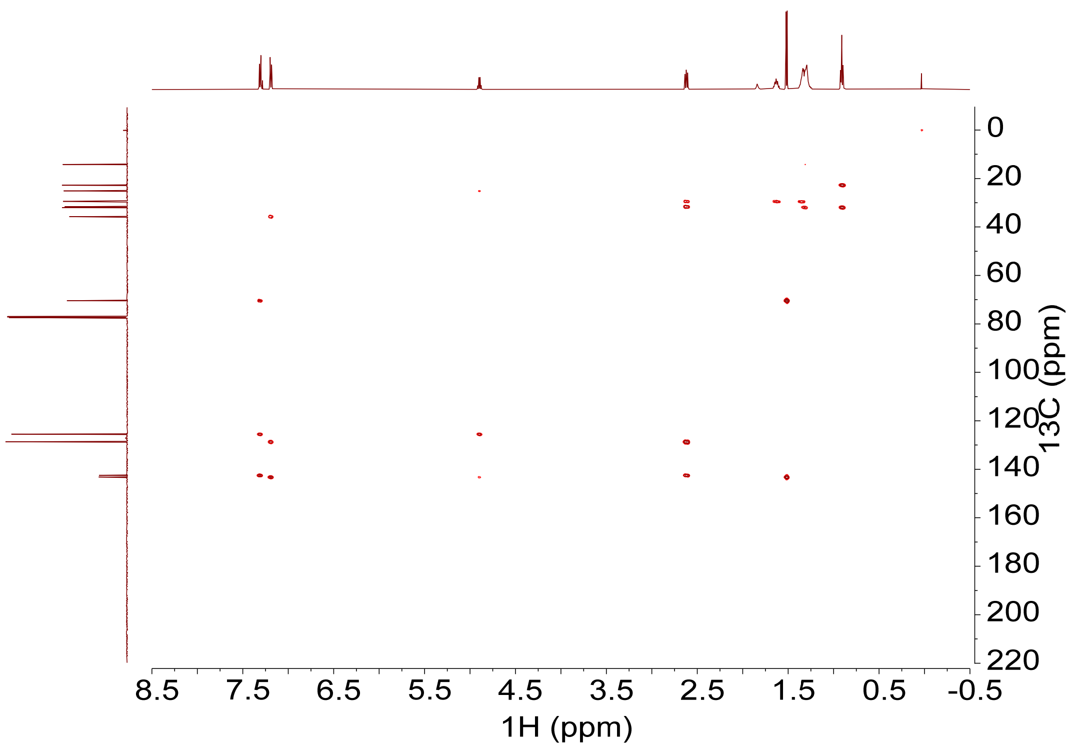

Fig. S191. *^{1}H-^{13}C HMBC NMR spectrum of **21** (500 MHz, $CDCl_3$, 298 K).*

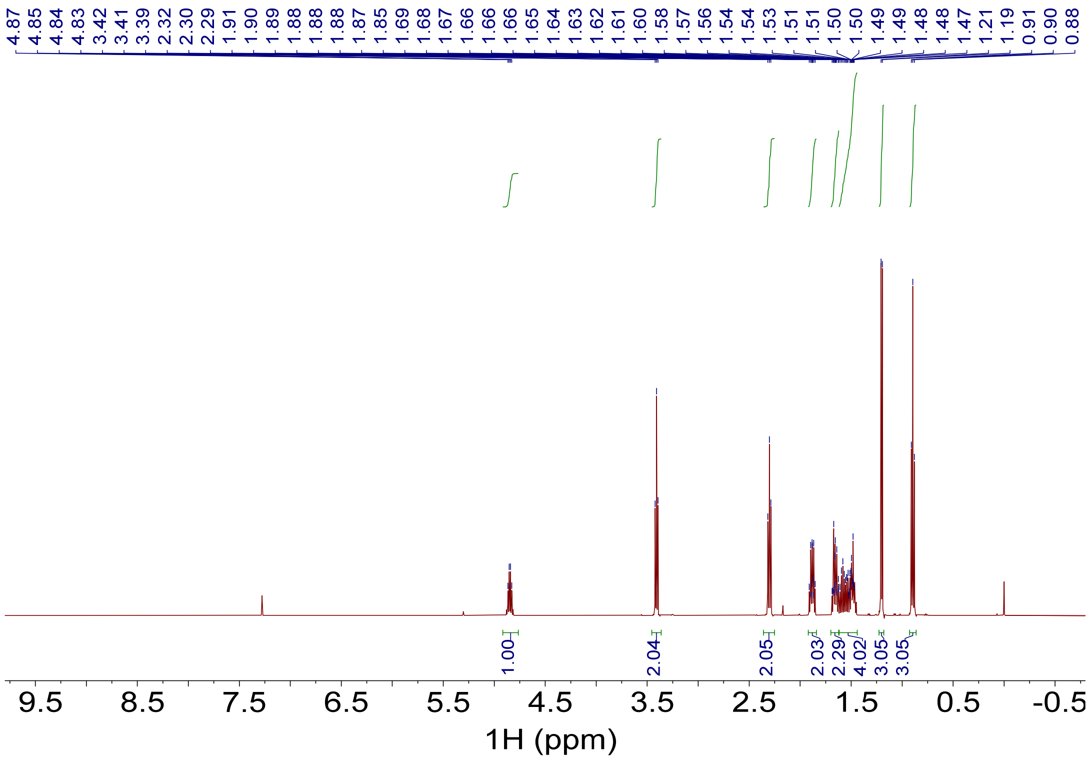

Fig. S192. *^{1}H NMR spectrum of (R)-**23** (500 MHz, $CDCl_3$, 298 K).*

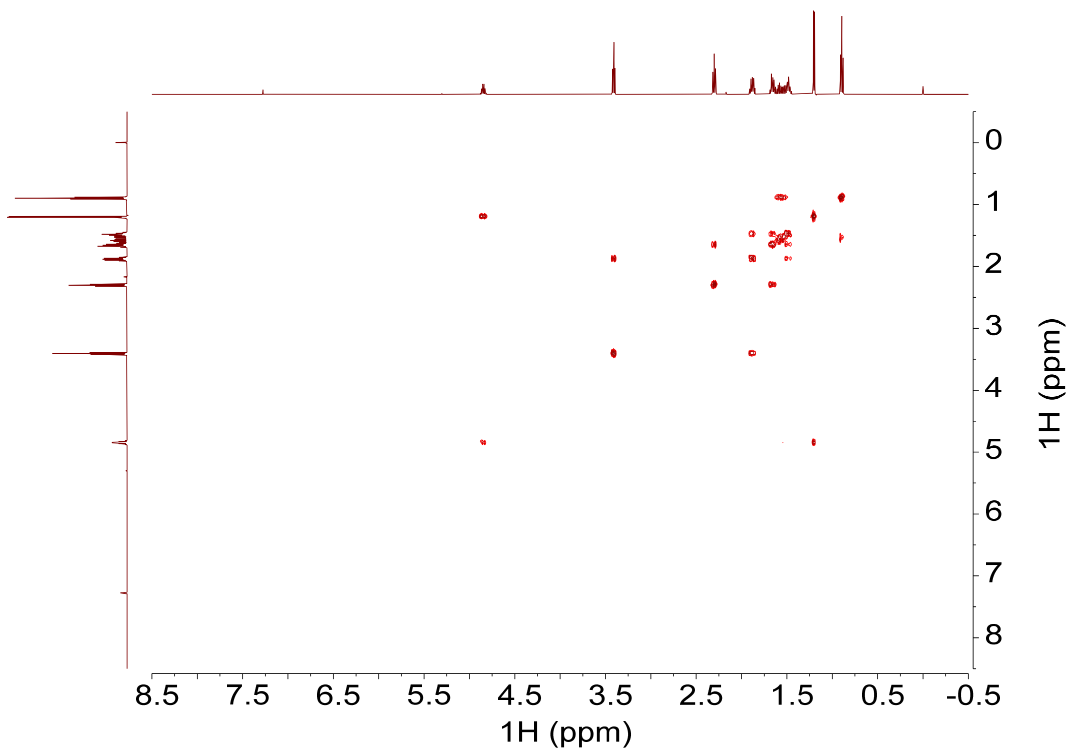

*Fig. S193. ^{1}H-^{1}H COSY NMR spectrum of (R)-**23** (500 MHz, $CDCl_3$, 298 K).*

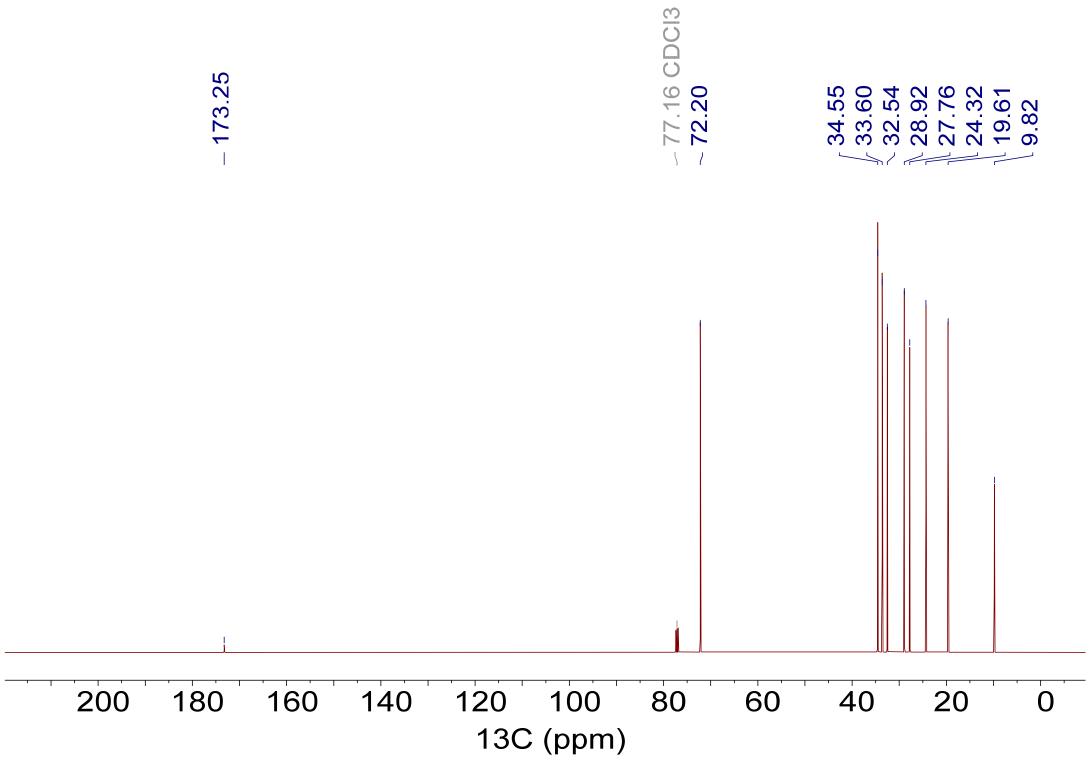

*Fig. S194. ^{13}C NMR spectrum of (R)-**23** (CPD, 126 MHz, $CDCl_3$, 298 K).*

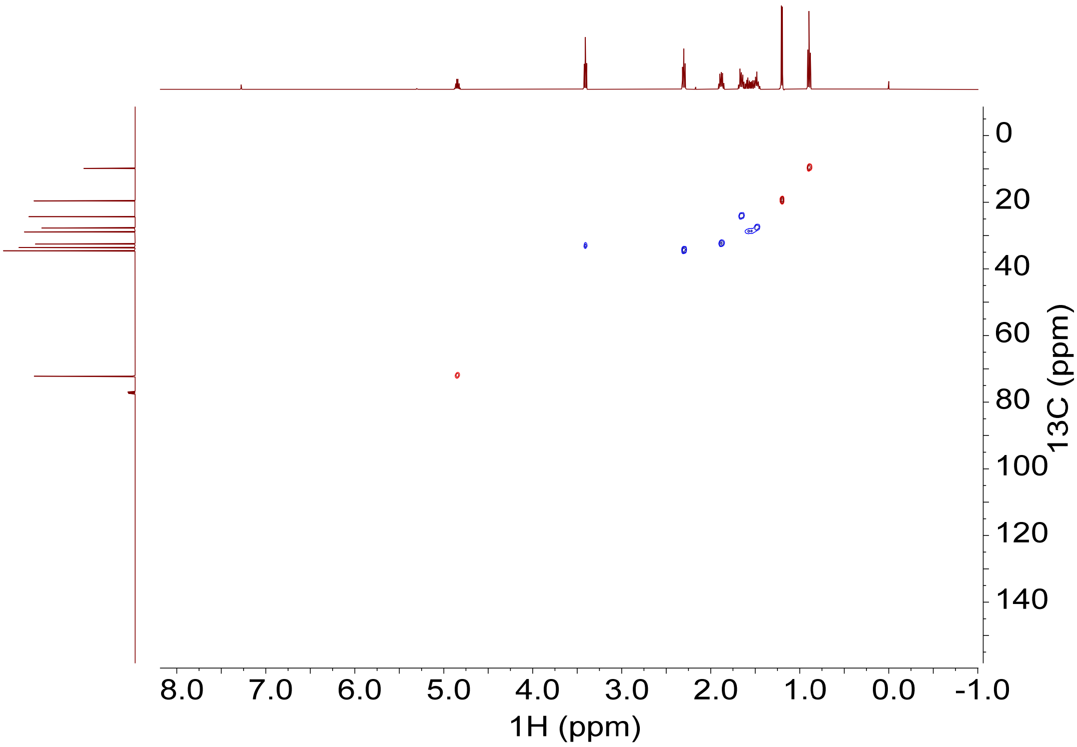

*Fig. S195. ¹H-¹³C HSQC NMR spectrum of (R)-**23** (500 MHz, $CDCl_3$, 298 K).*

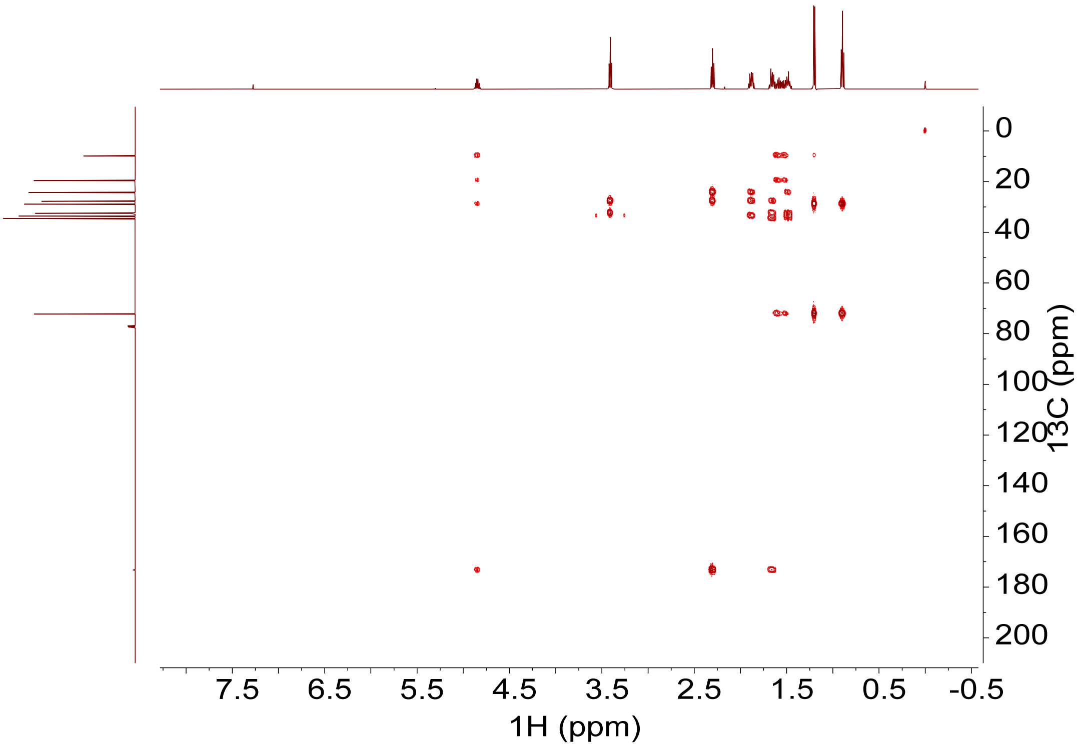

*Fig. S196. ¹H-¹³C HMBC NMR spectrum of (R)-**23** (500 MHz, $CDCl_3$, 298 K).*

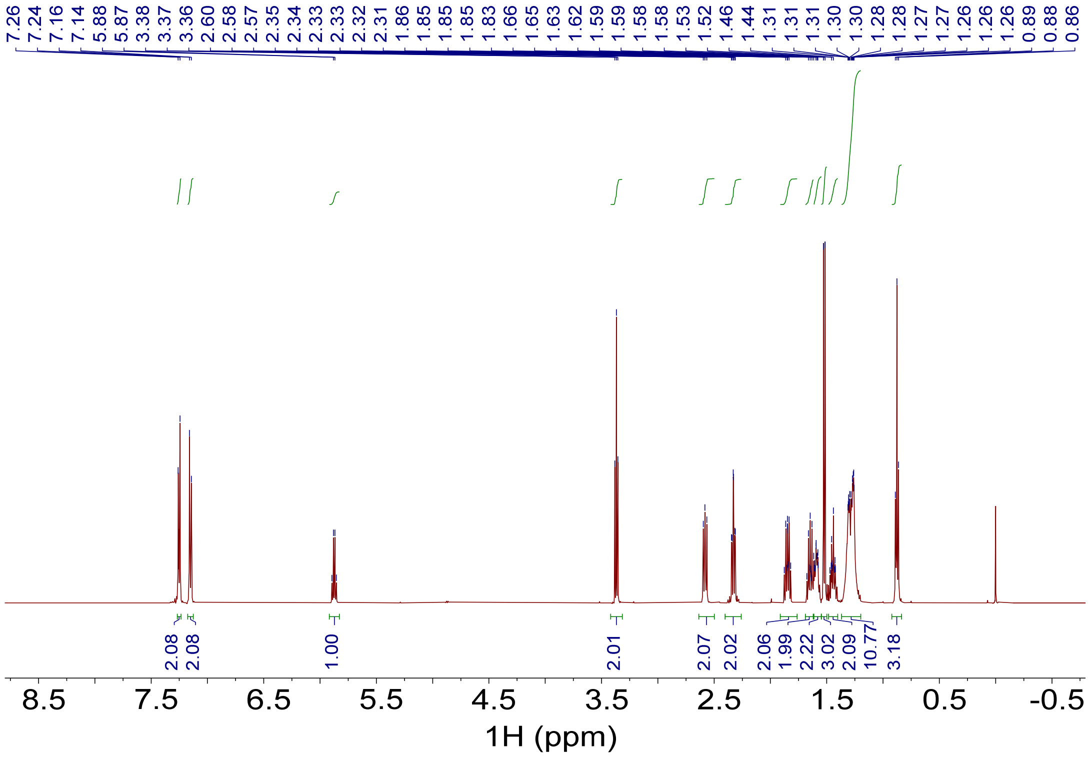

*Fig. S197. ¹H NMR spectrum of (R)-**24** (500 MHz, $CDCl_3$, 298 K).*

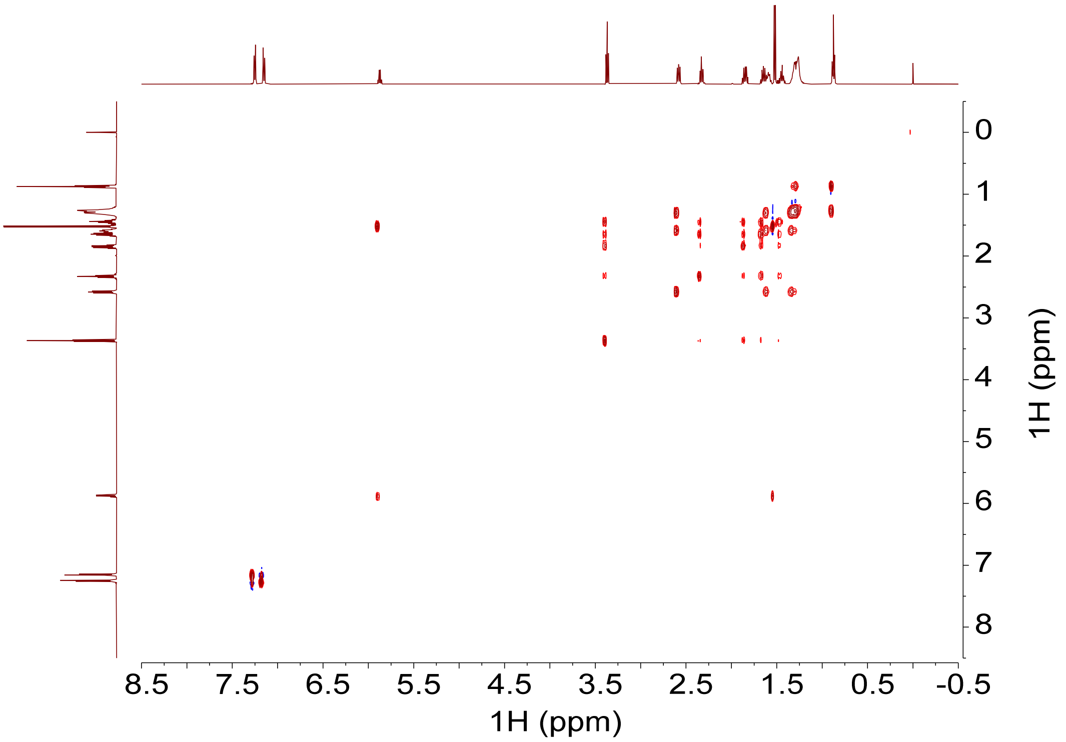

*Fig. S198. ¹H-¹H COSY NMR spectrum of (R)-**24** (500 MHz, $CDCl_3$, 298 K).*

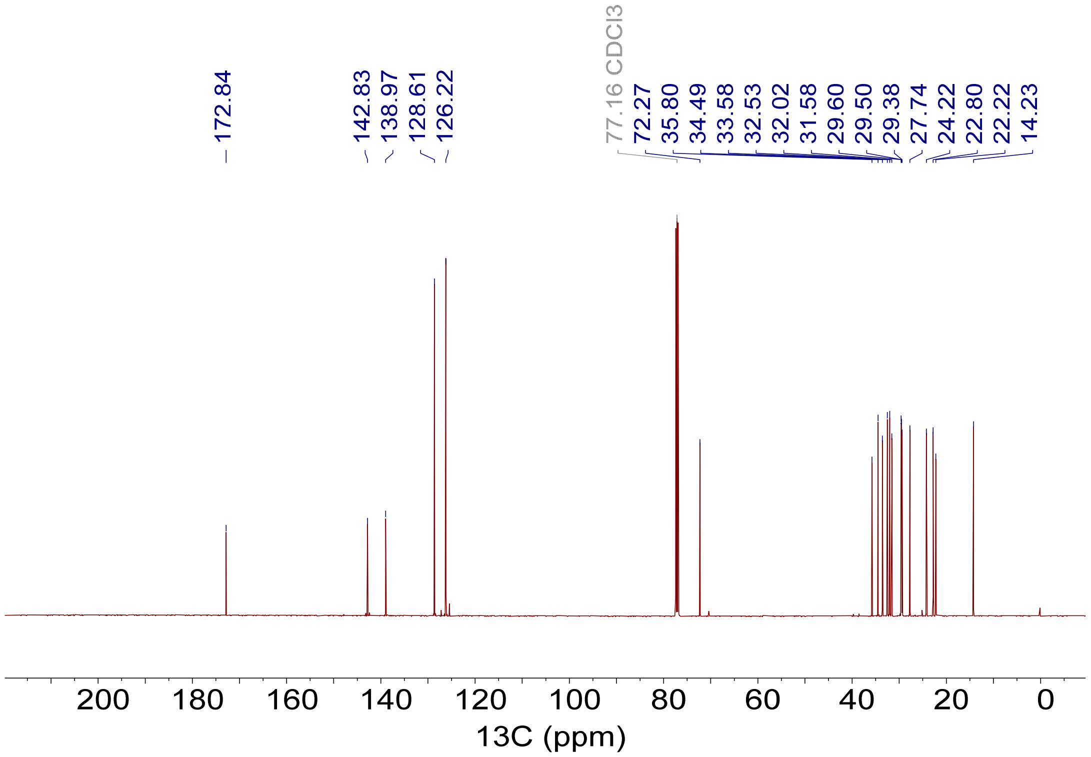

*Fig. S199. ^{13}C NMR spectrum of (R)-**24** (CPD, 126 MHz, $CDCl_3$, 298 K).*

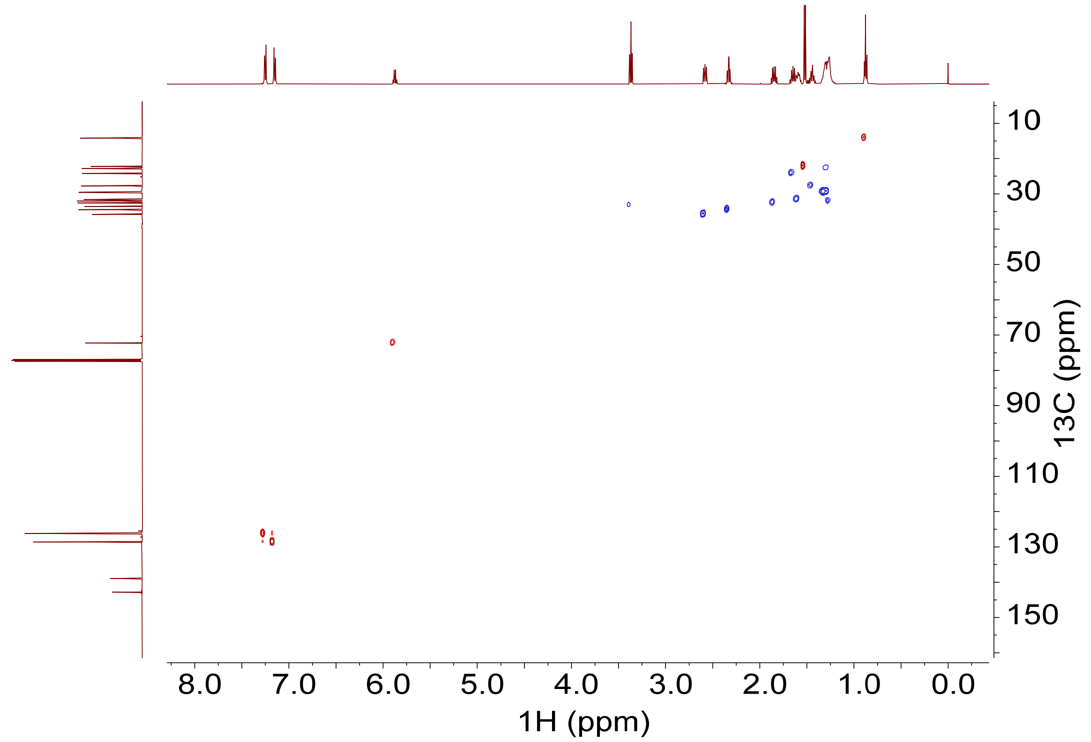

*Fig. S200. ^{1}H-^{13}C HSQC NMR spectrum of (R)-**24** (500 MHz, $CDCl_3$, 298 K).*

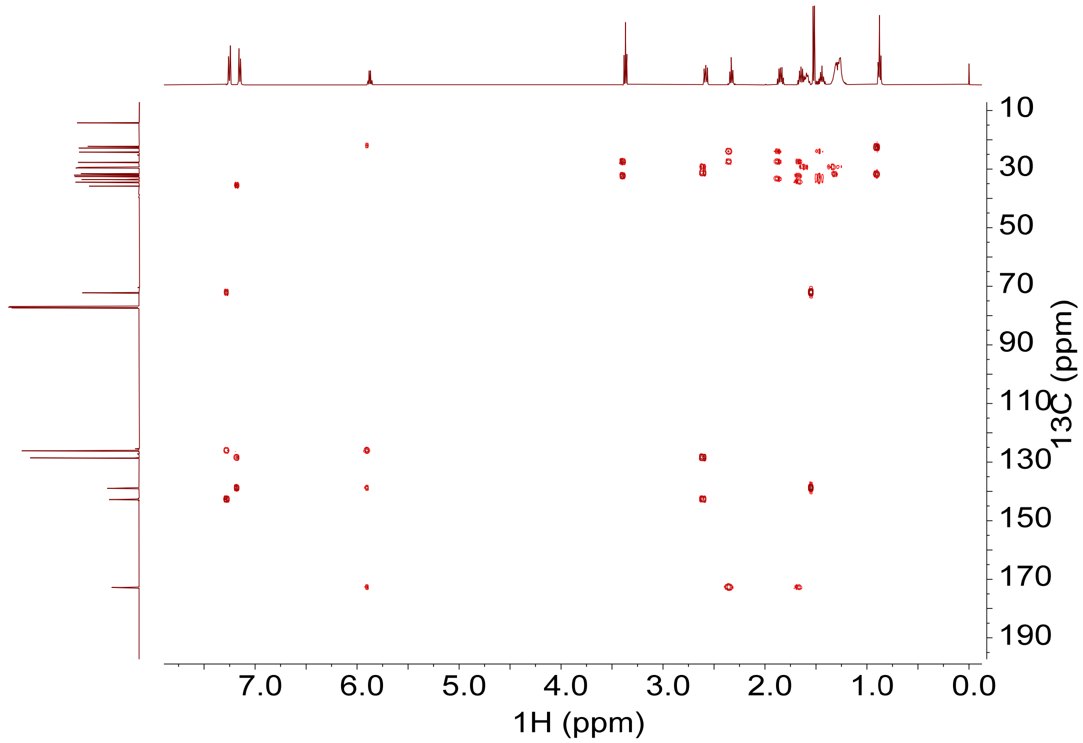

*Fig. S201. ¹H-¹³C HMBC NMR spectrum of (R)-**24** (500 MHz, CDCl$_3$, 298 K).*

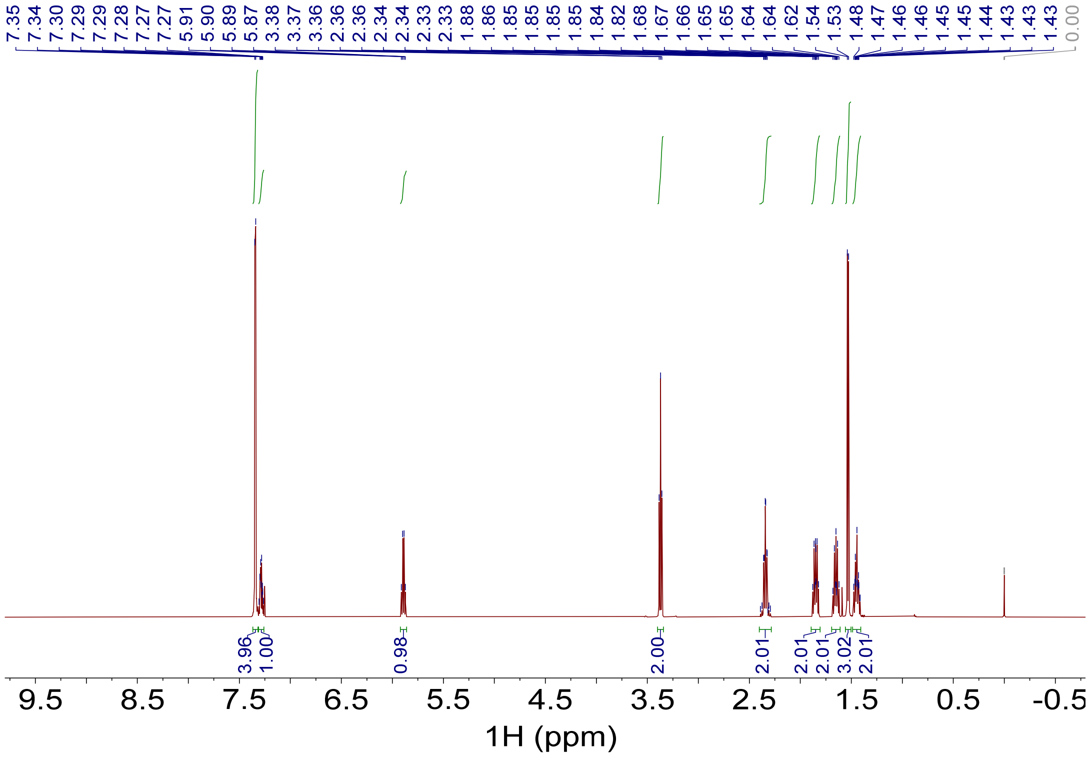

*Fig. S202. ¹H NMR spectrum of (S)-**25** (500 MHz, CDCl$_3$, 298 K).*

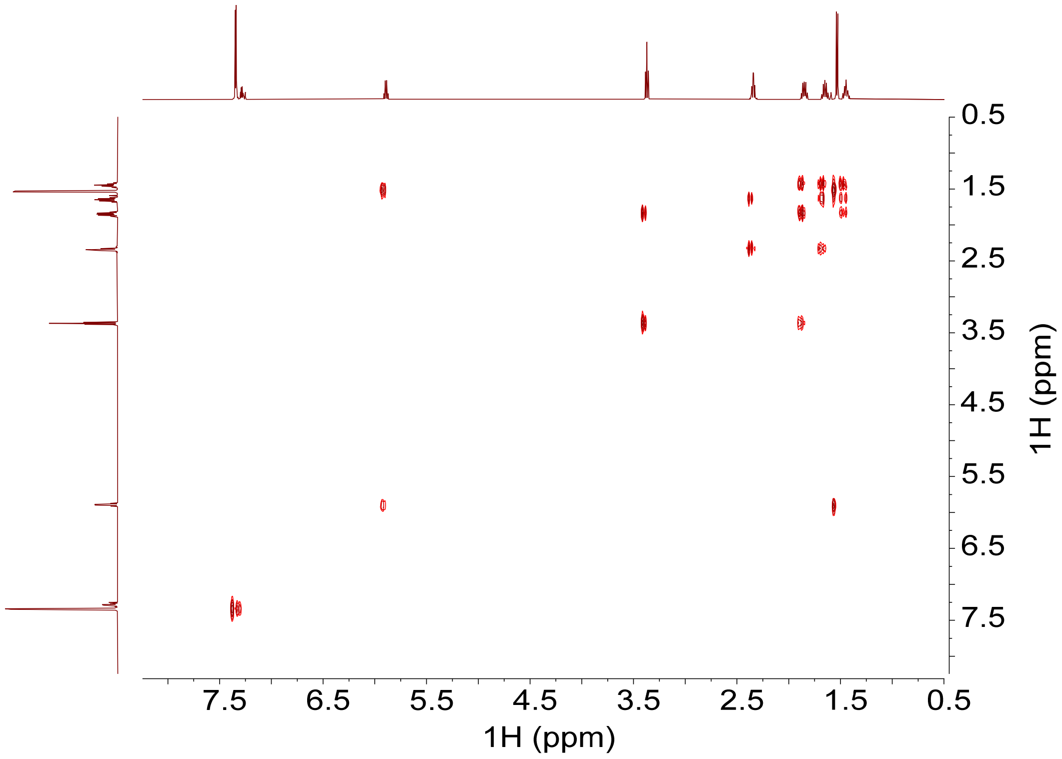

Fig. S203. *¹H-¹H COSY NMR spectrum of (S)-**25** (500 MHz, $CDCl_3$, 298 K).*

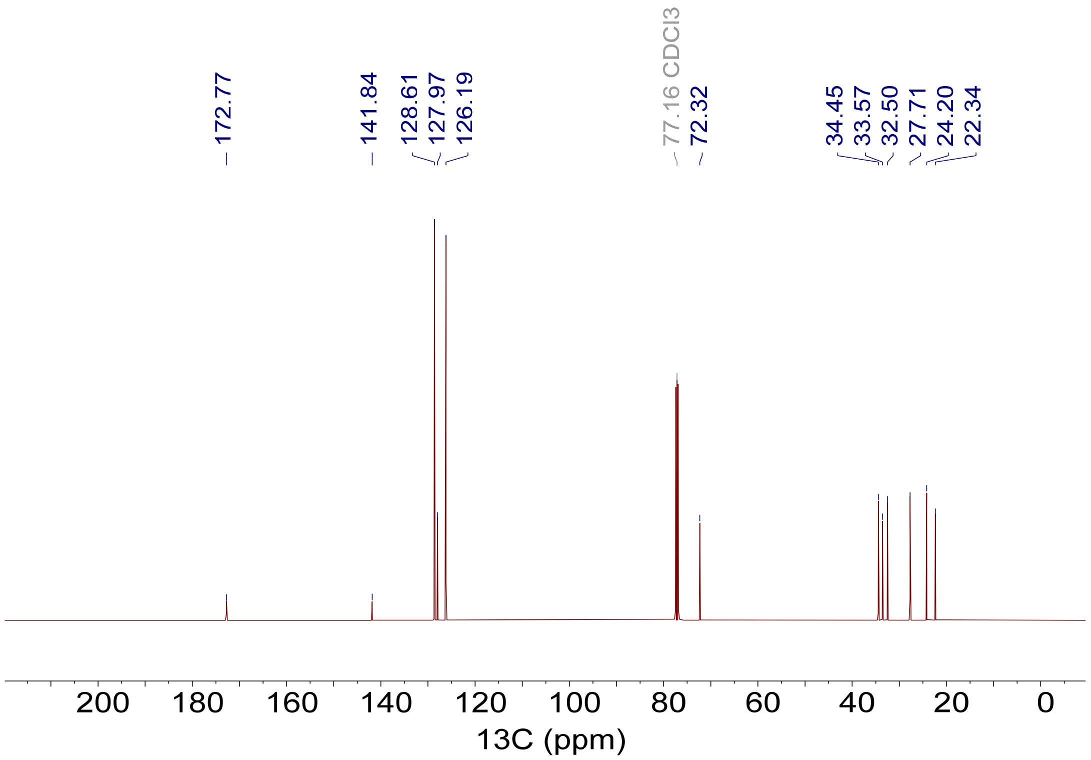

Fig. S204. *¹³C NMR spectrum of (S)-**25** (CPD, 126 MHz, $CDCl_3$, 298 K).*

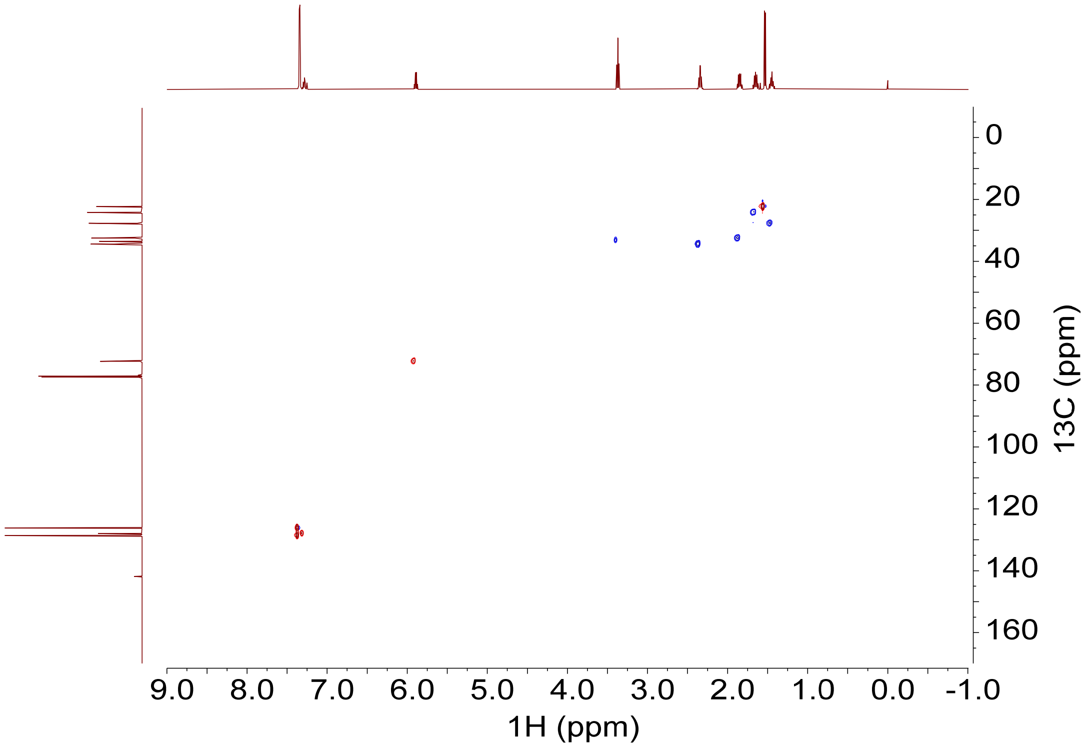

*Fig. S205. ^{1}H-^{13}C HSQC NMR spectrum of (S)-**25** (500 MHz, $CDCl_3$, 298 K).*

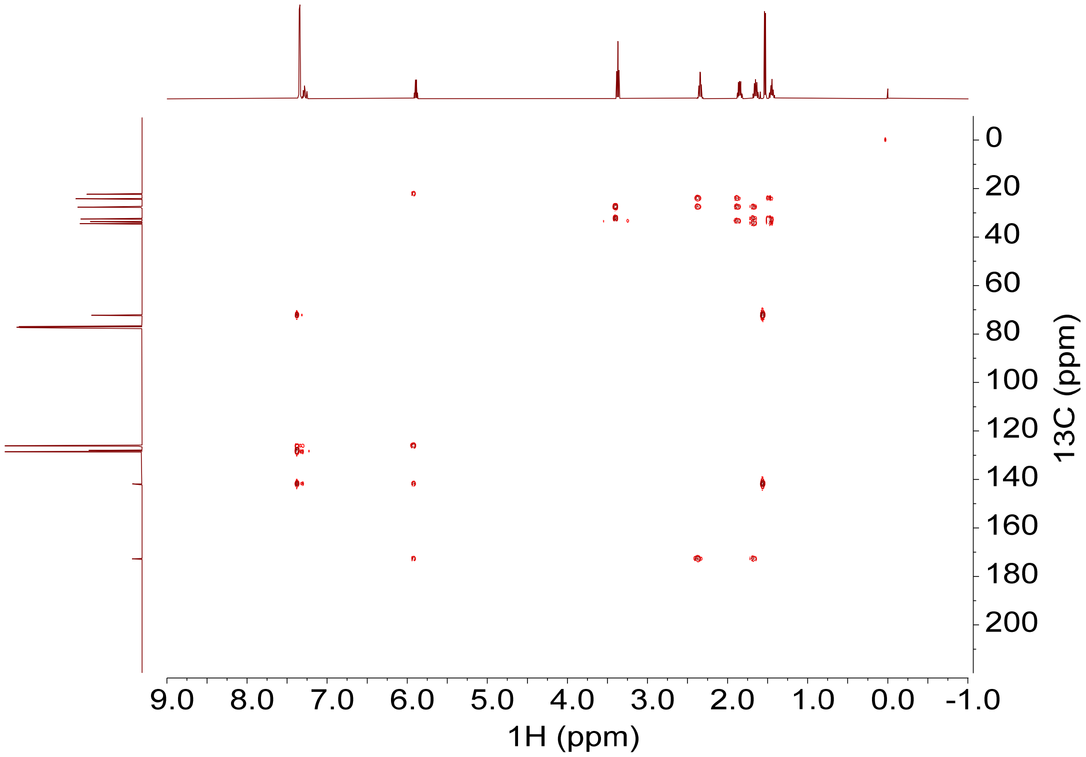

*Fig. S206. ^{1}H-^{13}C HMBC NMR spectrum of (S)-**25** (500 MHz, $CDCl_3$, 298 K).*

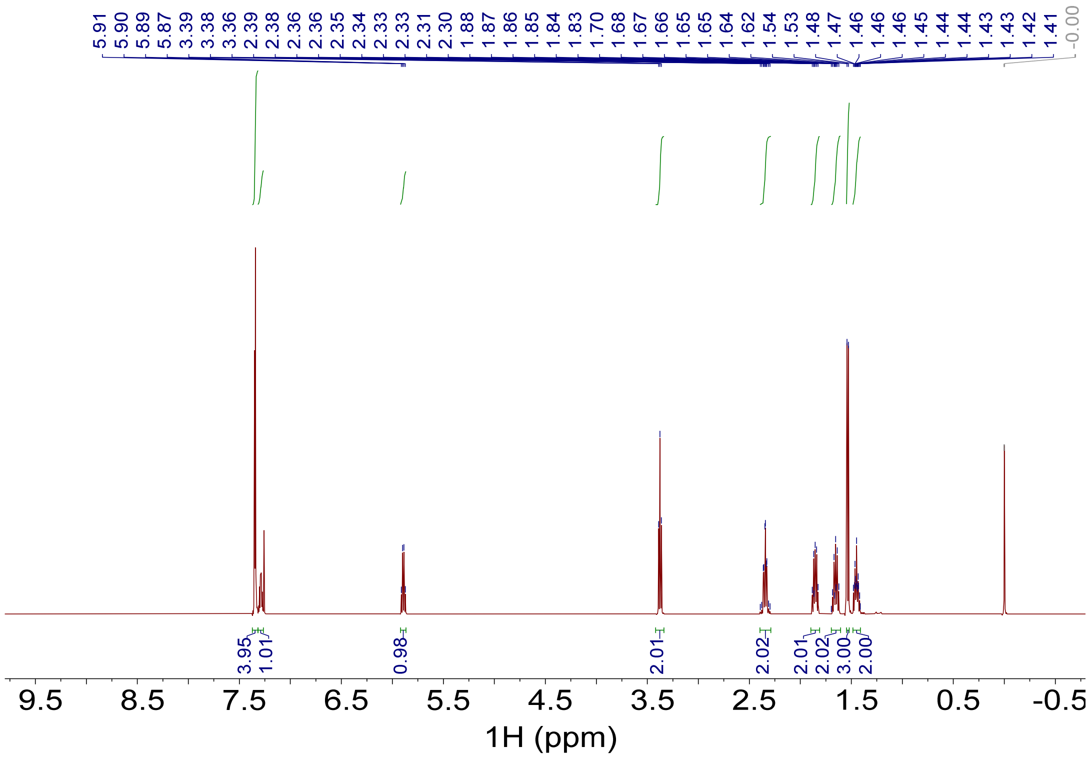

Fig. S207. [1]*H NMR spectrum of (R)-**25** (500 MHz, $CDCl_3$, 298 K).*

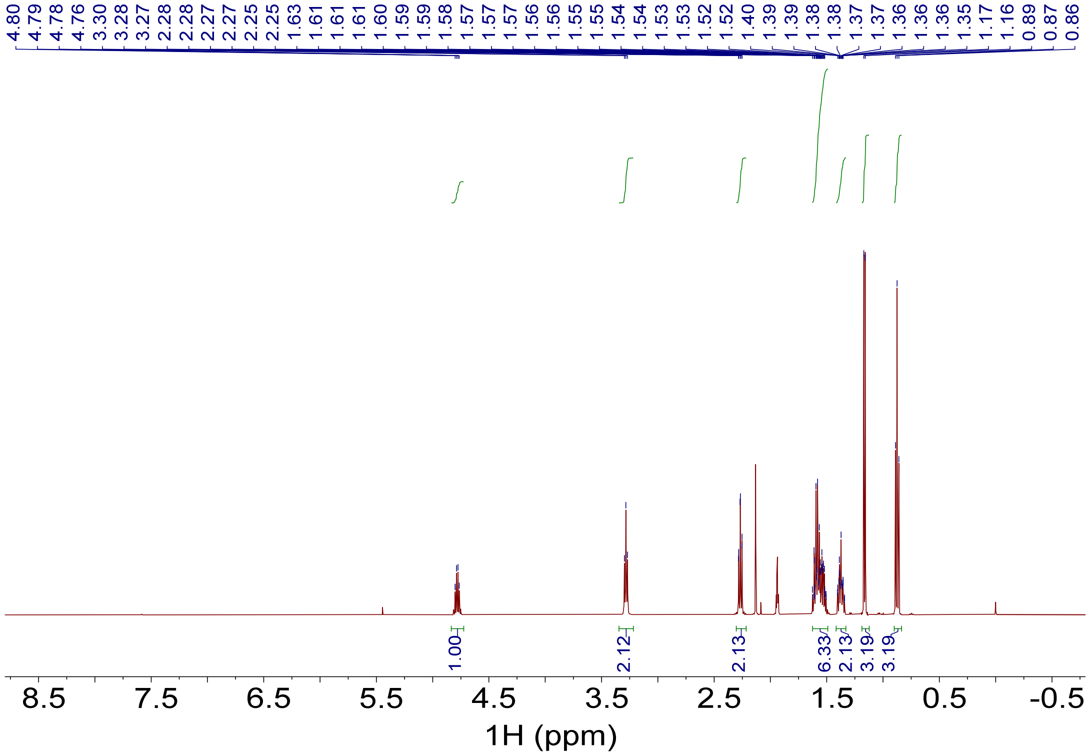

Fig. S208. [1]*H NMR spectrum of (R)-**26** (500 MHz, CD_3CN, 298 K).*

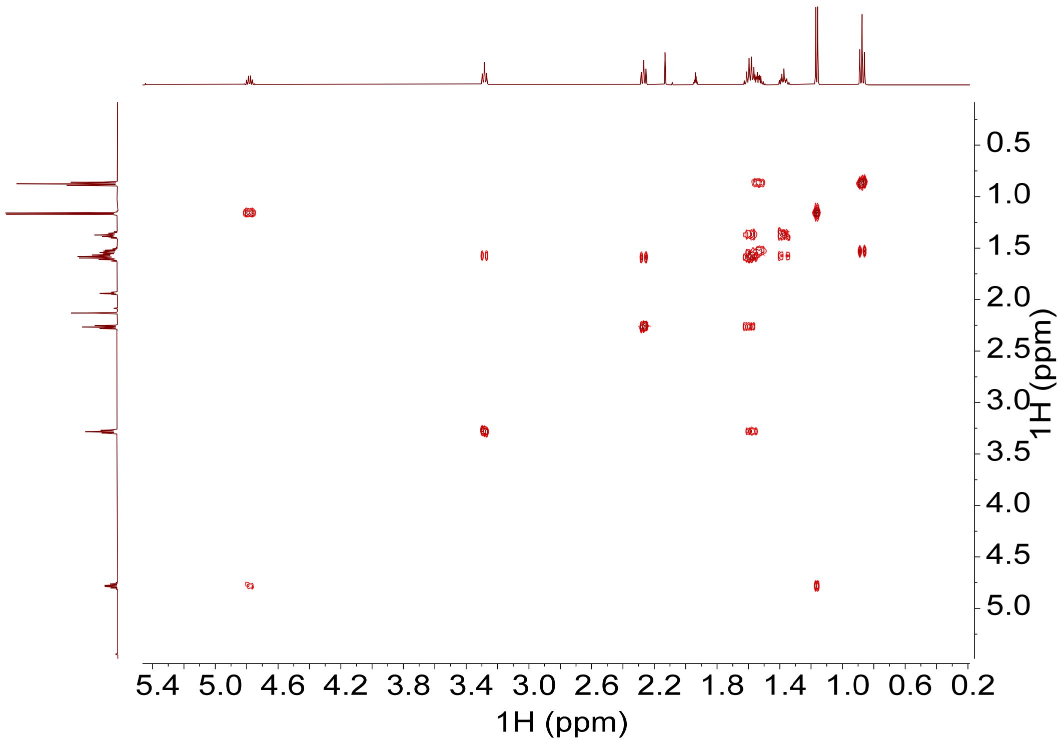

*Fig. S209. ^{1}H-^{1}H COSY NMR spectrum of (R)-**26** (500 MHz, CD_3CN, 298 K).*

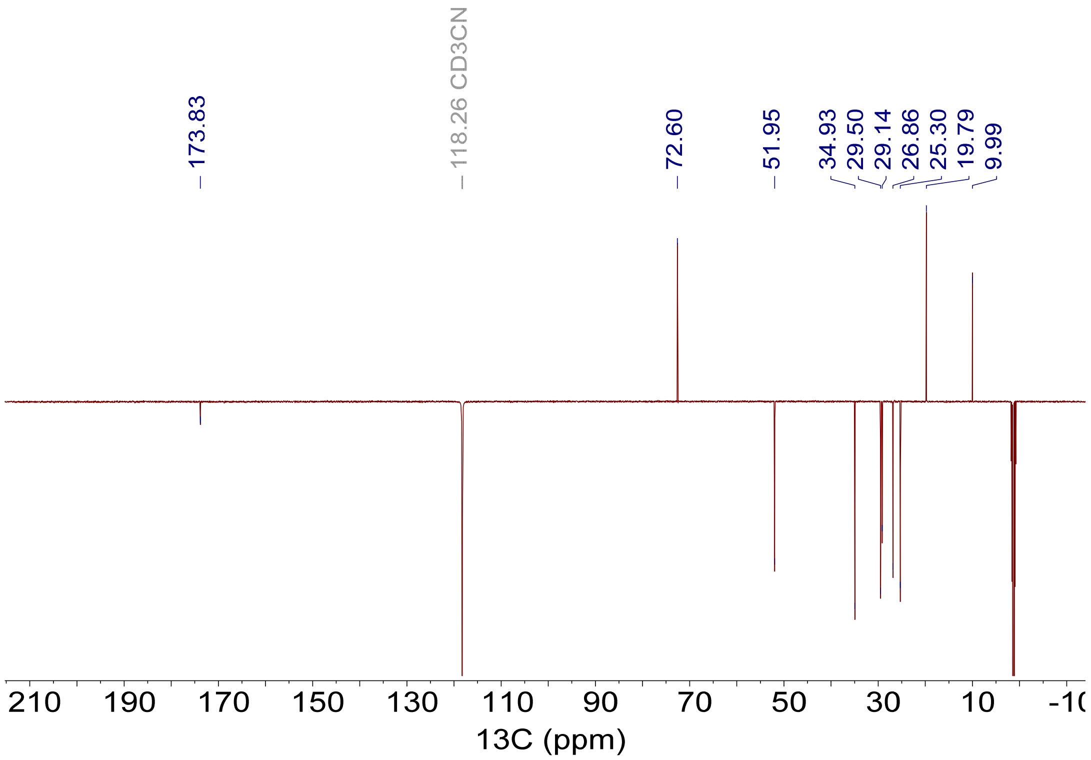

*Fig. S210. ^{13}C NMR spectrum of (R)-**26** (APT, 126 MHz, CD_3CN, 298 K).*

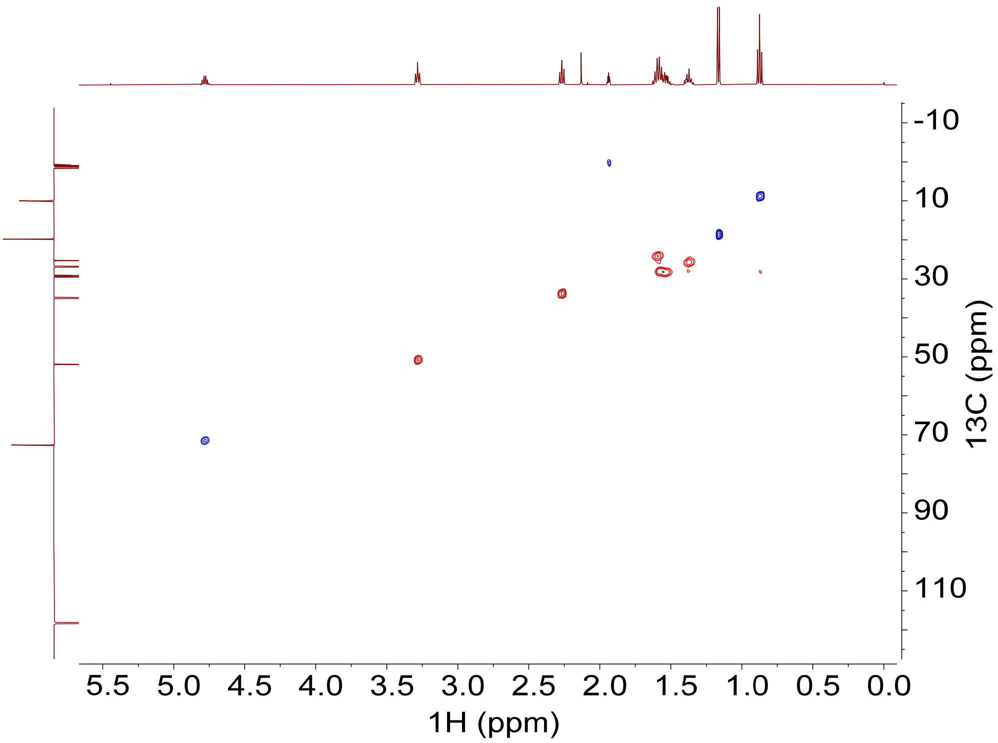

*Fig. S211. ^{1}H-^{13}C HSQC NMR spectrum of (R)-**26** (500 MHz, CD_3CN, 298 K).*

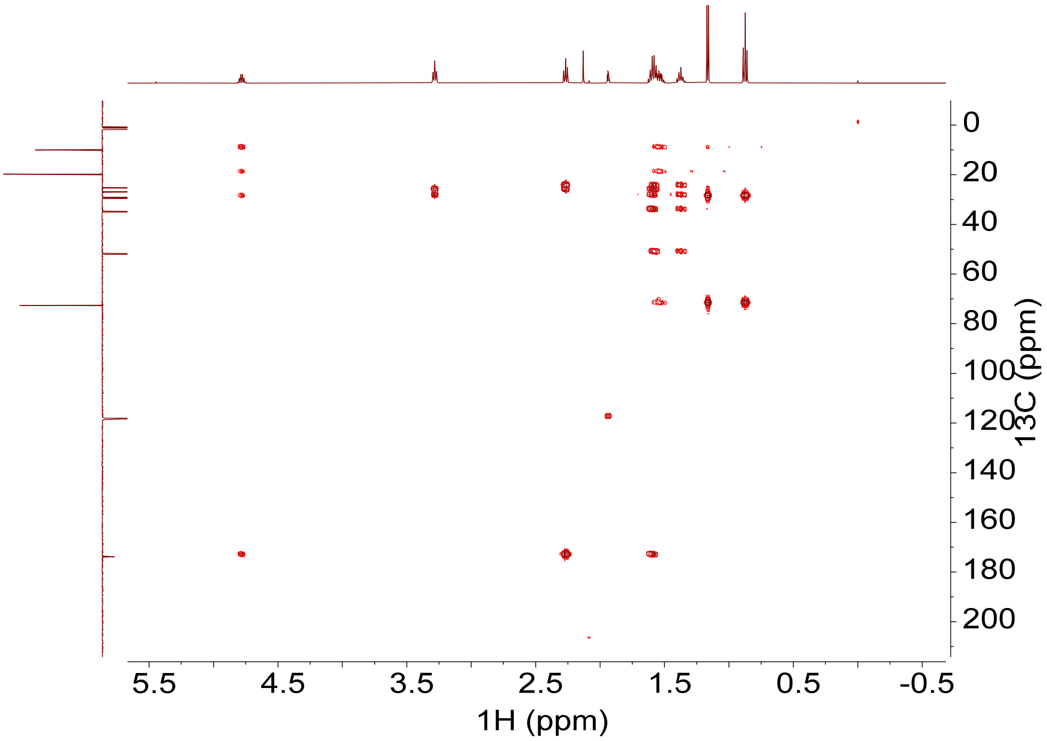

*Fig. S212. ^{1}H-^{13}C HMBC NMR spectrum of (R)-**26** (500 MHz, CD_3CN, 298 K).*

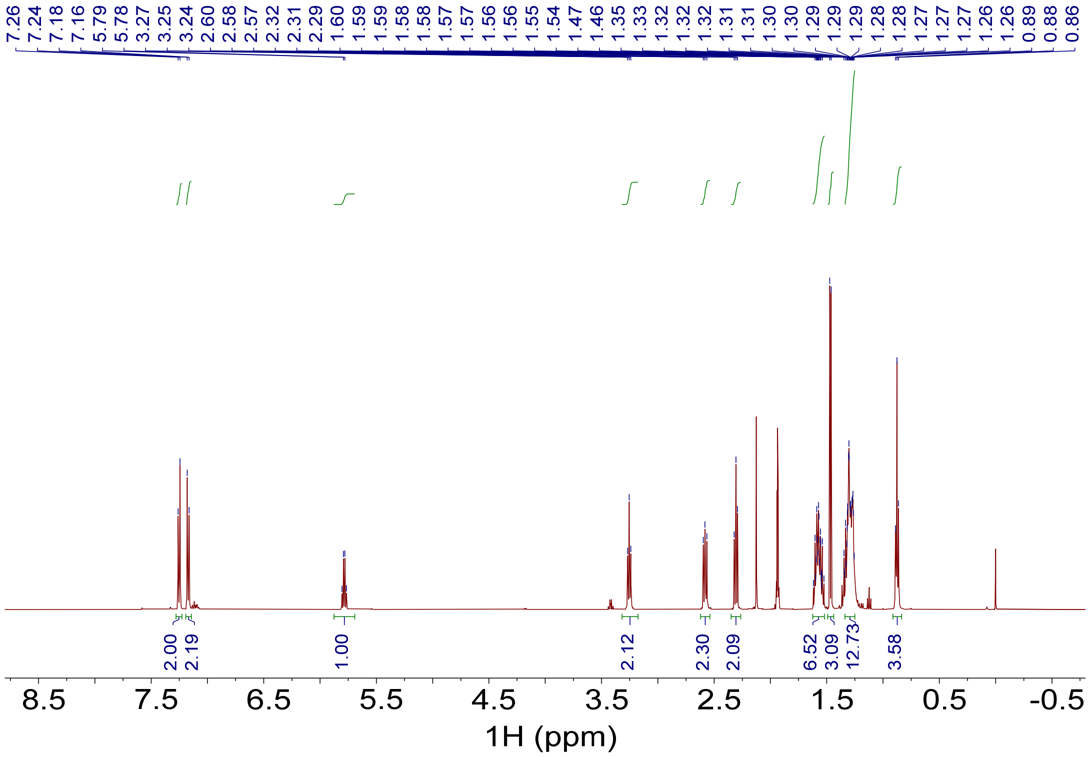

*Fig. S213. ^{1}H NMR spectrum of (R)-**27** (500 MHz, CD_3CN, 298 K).*

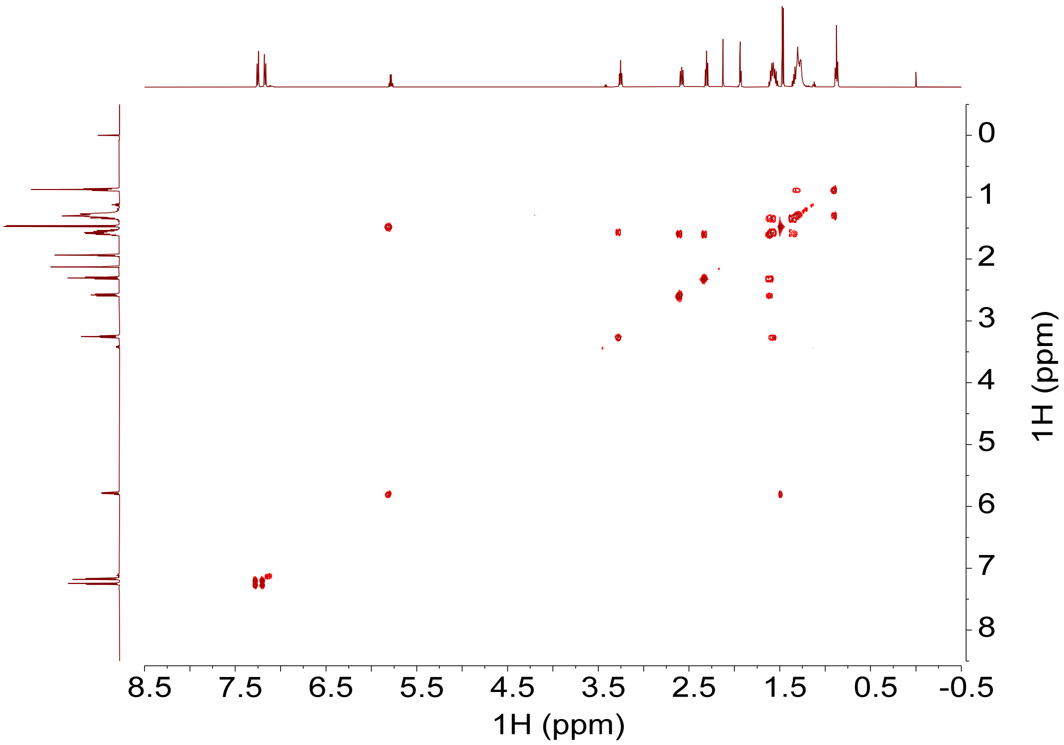

*Fig. S214. ^{1}H-^{1}H COSY NMR spectrum of (R)-**27** (500 MHz, CD_3CN, 298 K).*

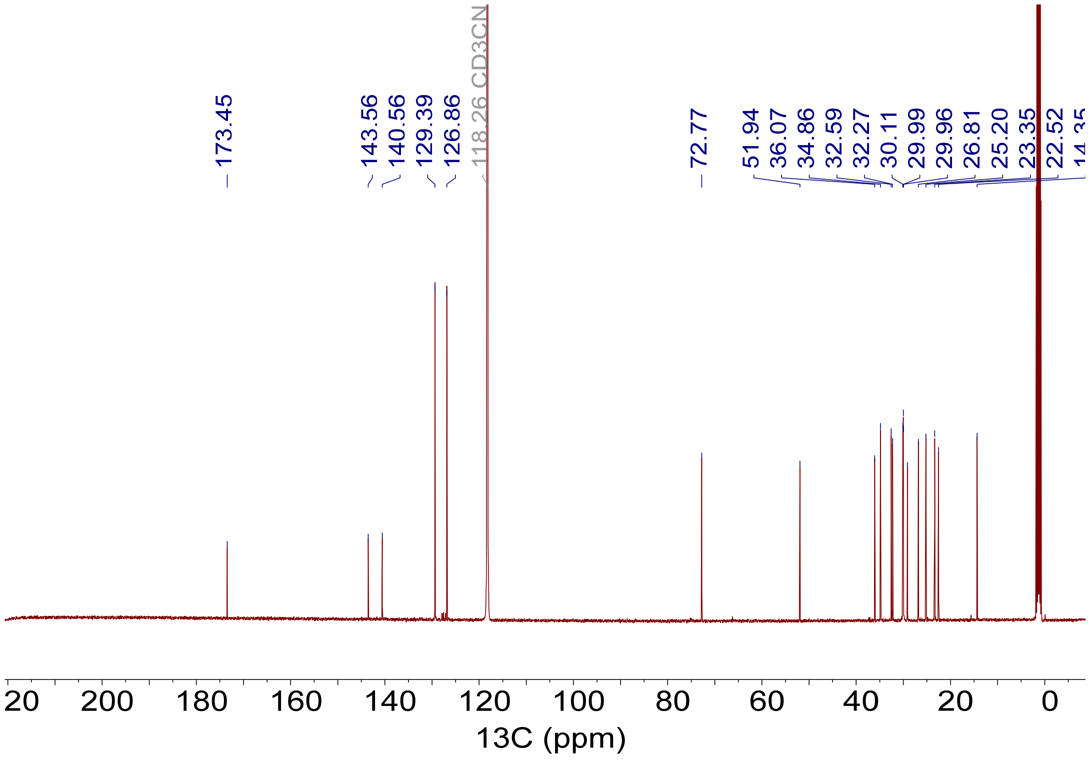

*Fig. S215. ^{13}C NMR spectrum of (R)-**27** (CPD, 126 MHz, CD_3CN, 298 K).*

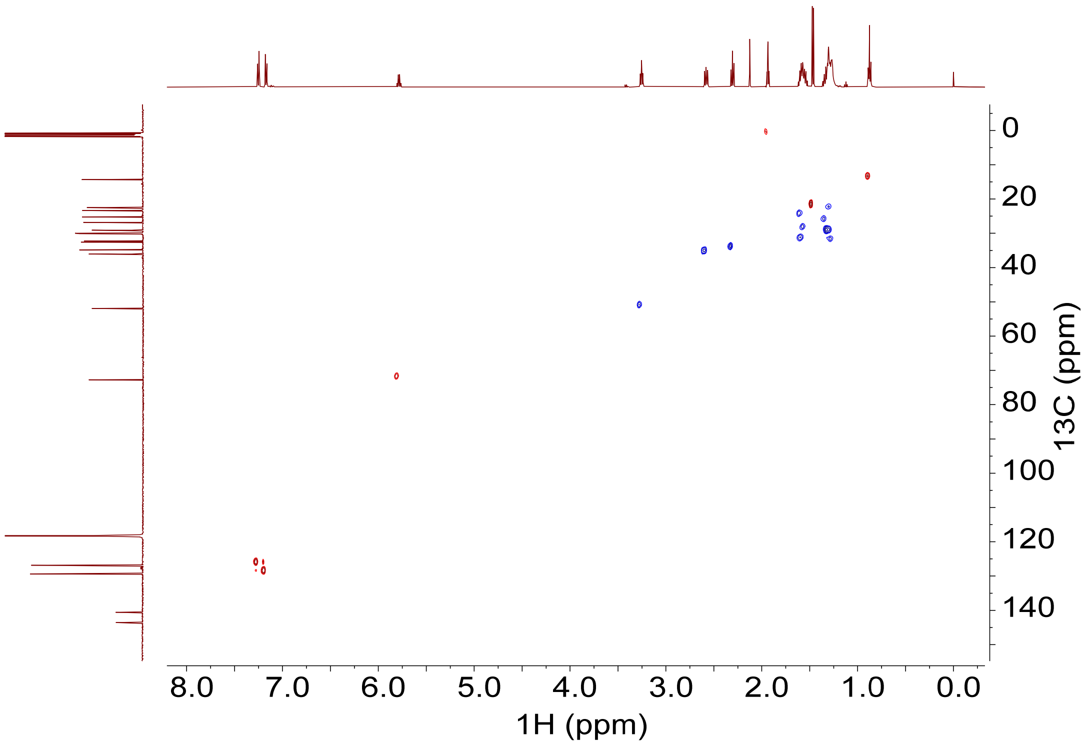

*Fig. S216. ^{1}H-^{13}C HSQC NMR spectrum of (R)-**27** (500 MHz, CD_3CN, 298 K).*

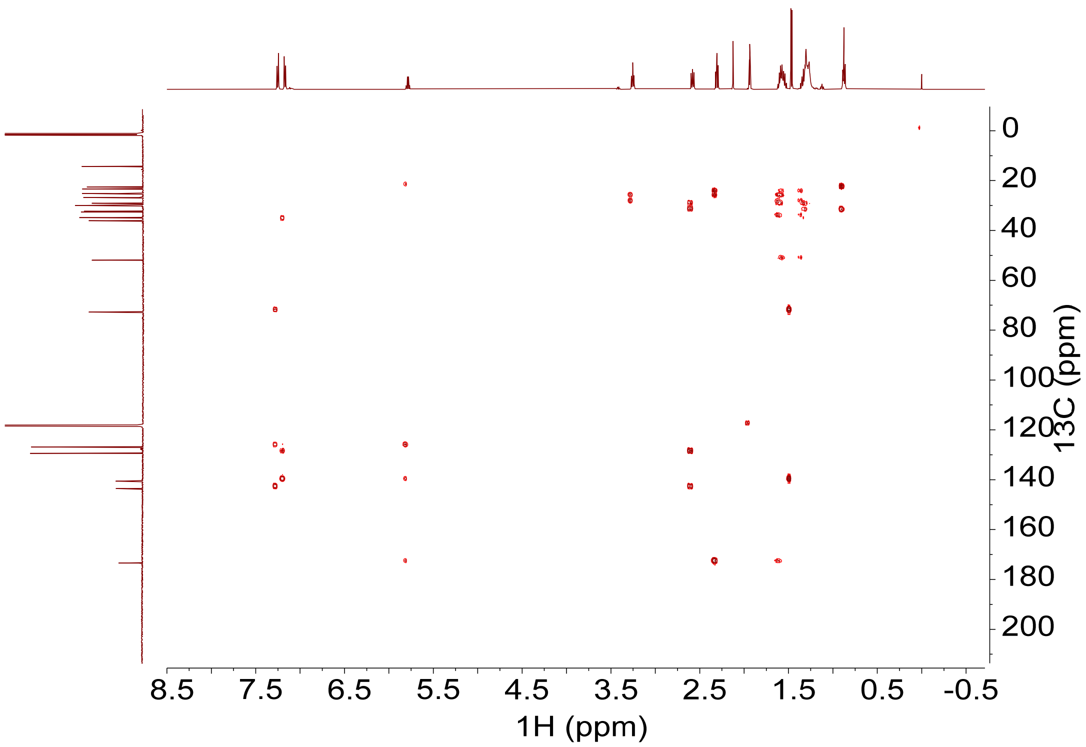

Fig. S217. *^{1}H-^{13}C HMBC NMR spectrum of (R)-**27** (500 MHz, CD_3CN, 298 K).*

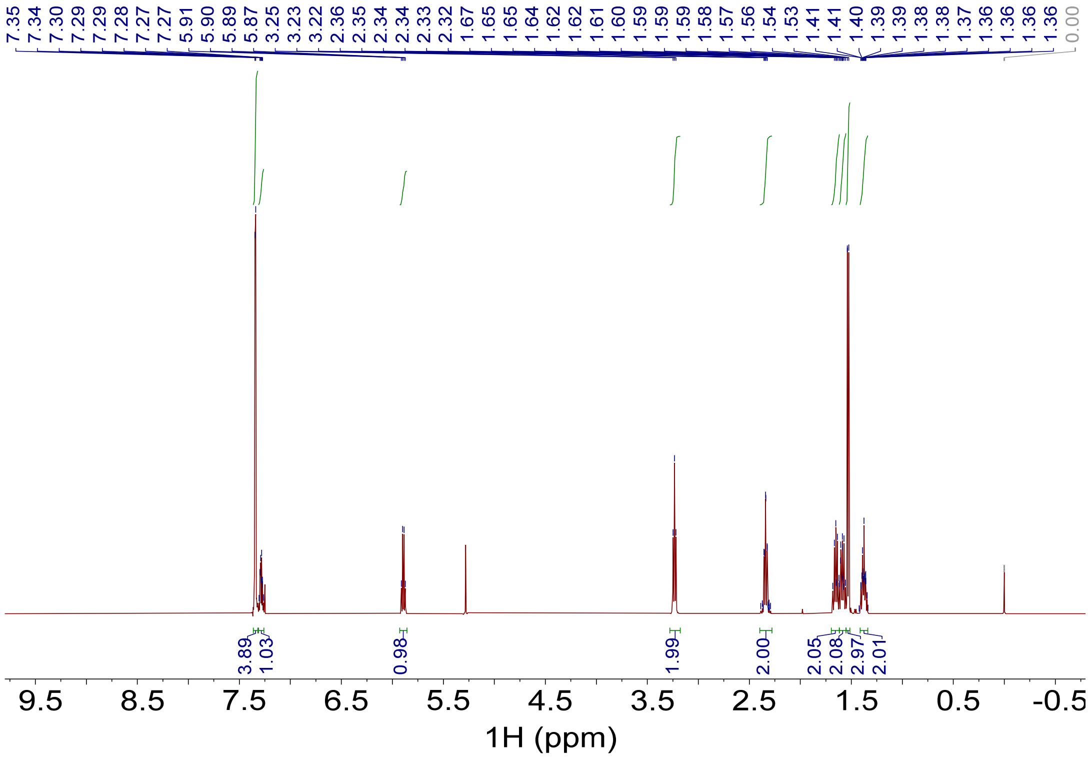

Fig. S218. *^{1}H NMR spectrum of (S)-**28** (500 MHz, $CDCl_3$, 298 K).*

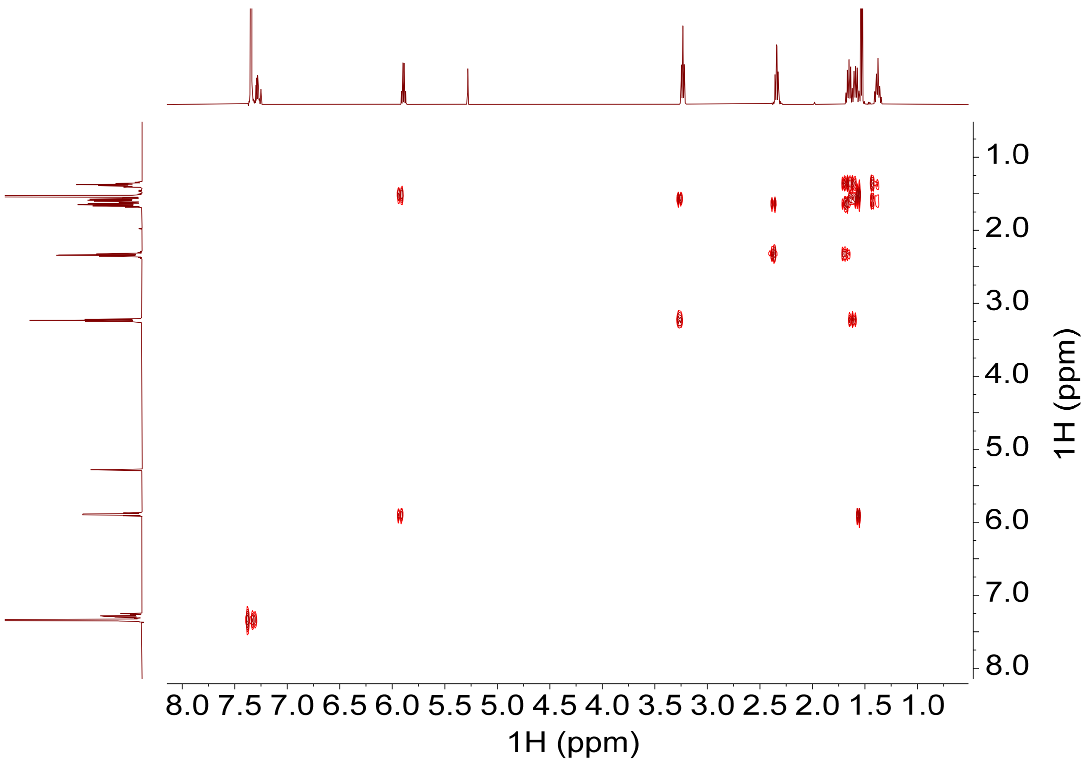

*Fig. S219. ^{1}H-^{1}H COSY NMR spectrum of (S)-**28** (500 MHz, $CDCl_3$, 298 K).*

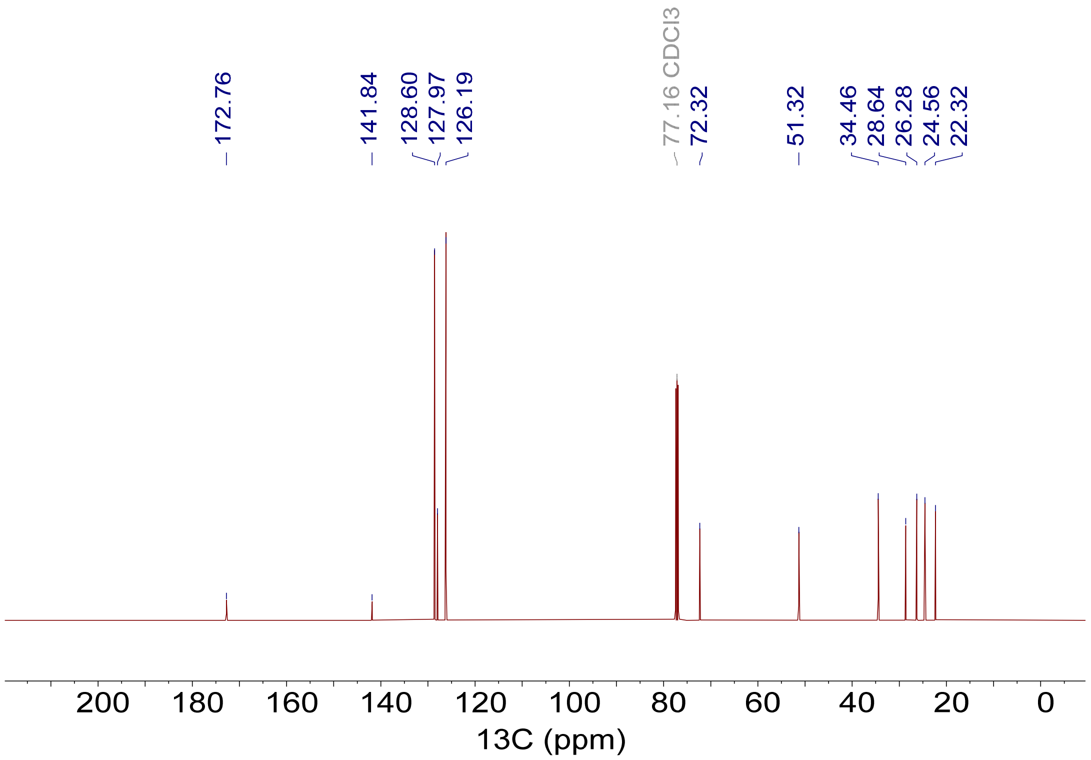

*Fig. S220. ^{13}C NMR spectrum of (S)-**28** (CPD, 126 MHz, $CDCl_3$, 298 K).*

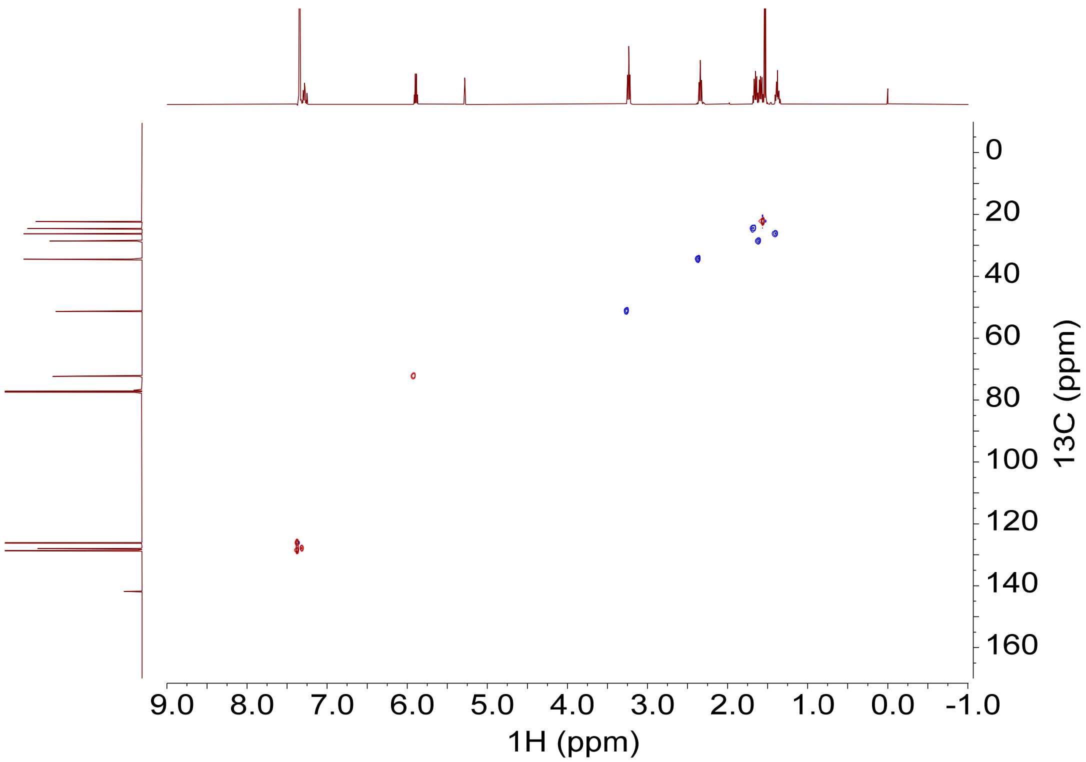

*Fig. S221. ^{1}H-^{13}C HSQC NMR spectrum of (S)-**28** (500 MHz, $CDCl_3$, 298 K).*

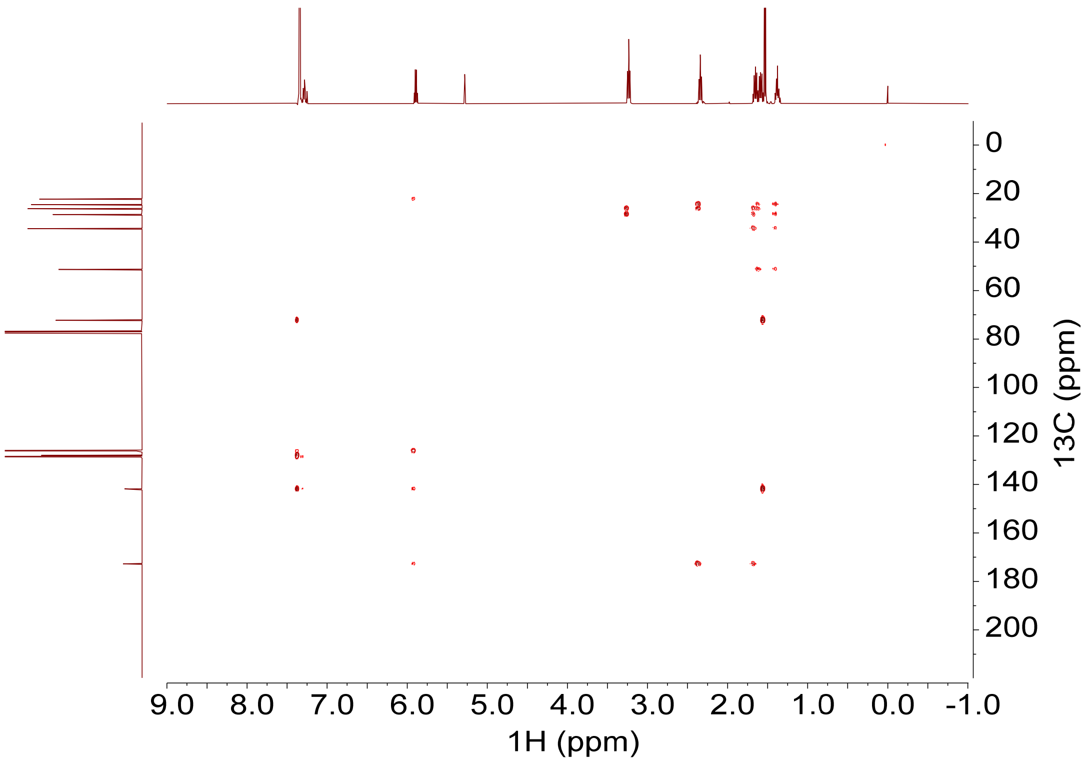

*Fig. S222. ^{1}H-^{13}C HMBC NMR spectrum of (S)-**28** (500 MHz, $CDCl_3$, 298 K).*

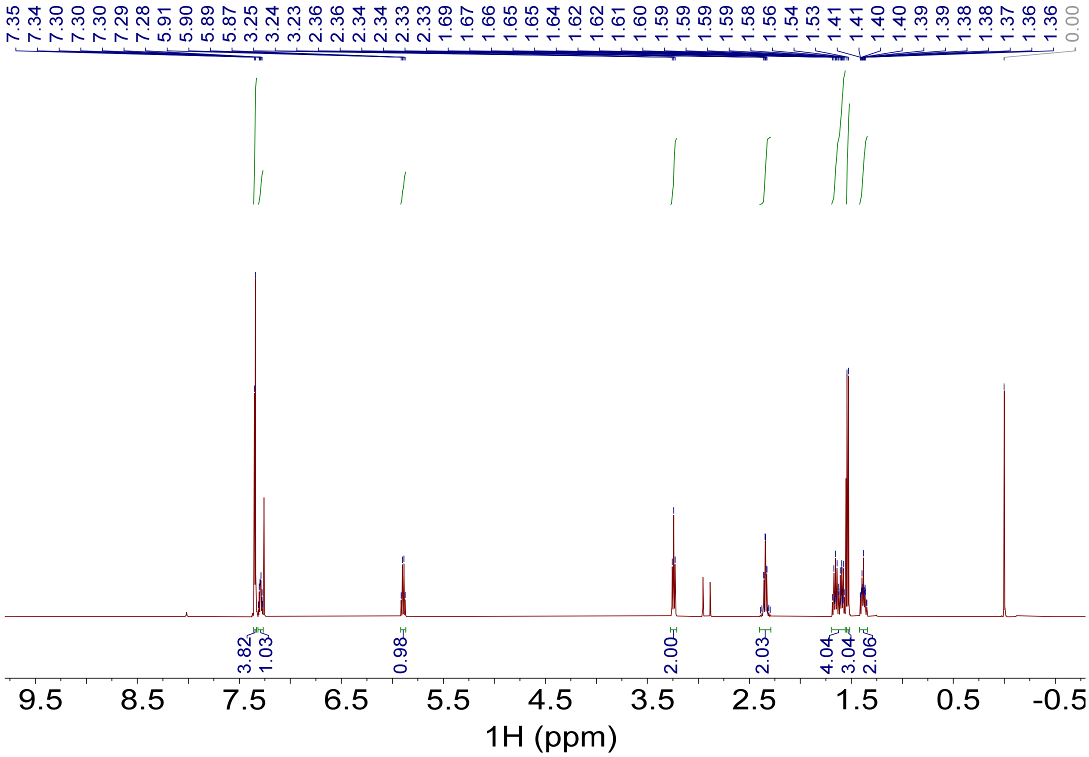

Fig. S223. [1]*H NMR spectrum of (R)-**28** (500 MHz, $CDCl_3$, 298 K).*

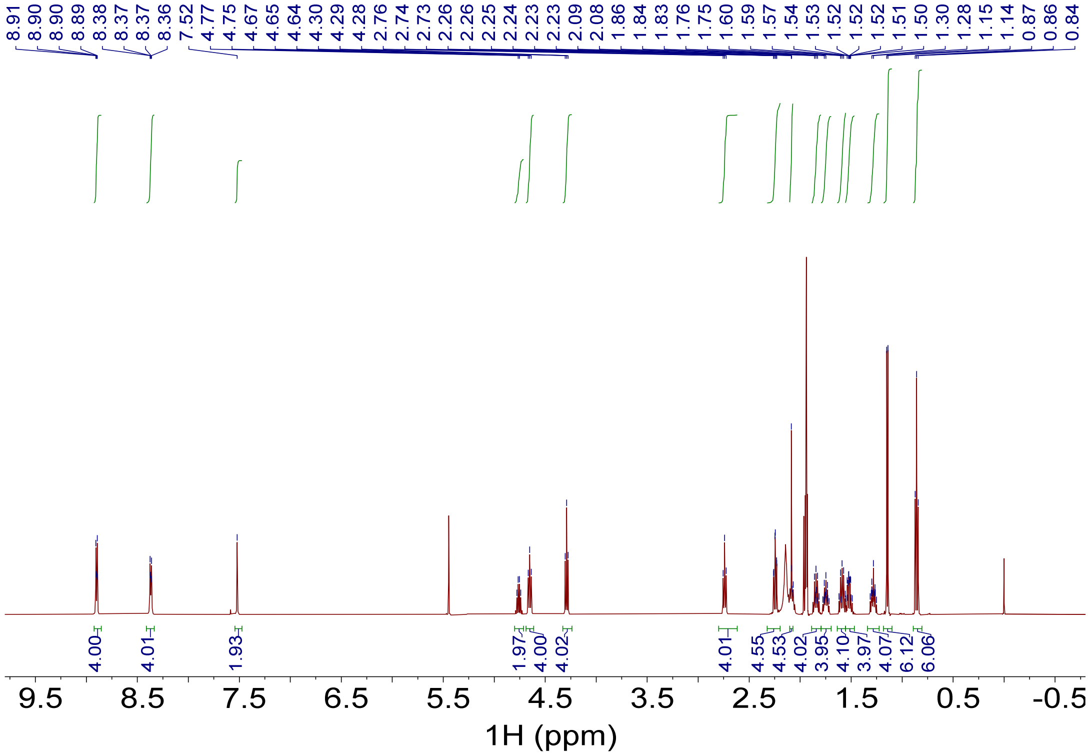

Fig. S224. [1]*H NMR spectrum of (R,R)-**16** (500 MHz, CD_3CN, 298 K).*

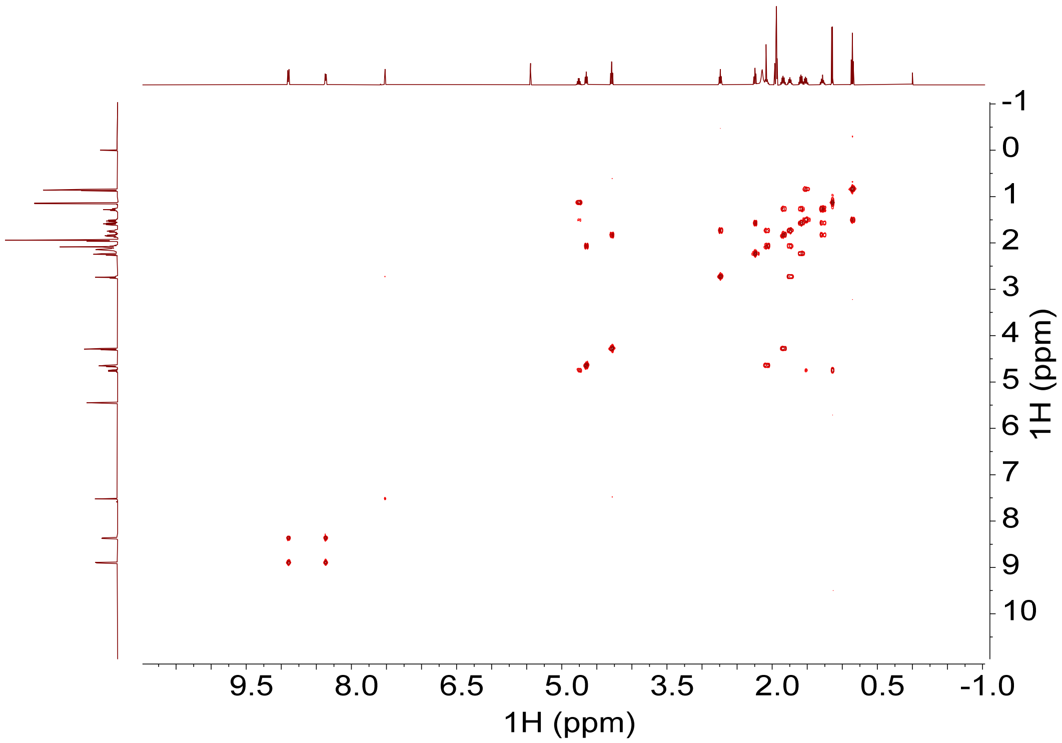

*Fig. S225. ^{1}H-^{1}H COSY NMR spectrum of (R,R)-**16** (500 MHz, CD_3CN, 298 K).*

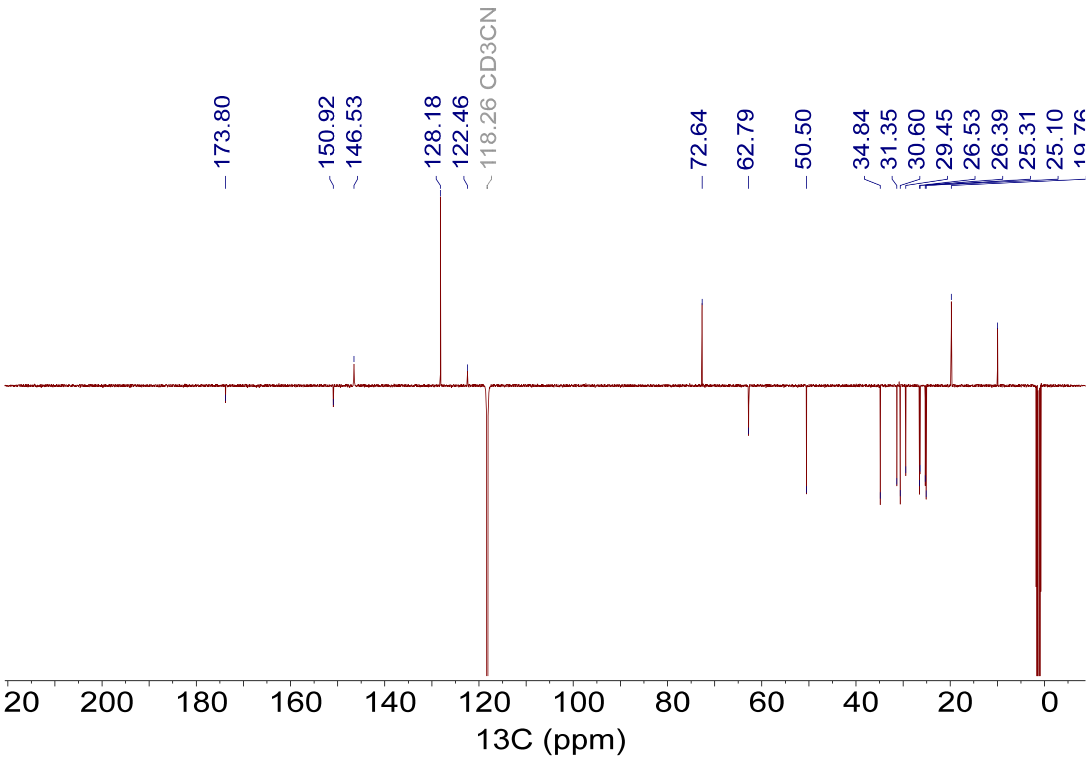

*Fig. S226. ^{13}C NMR spectrum of (R,R)-**16** (APT, 126 MHz, CD_3CN, 298 K).*

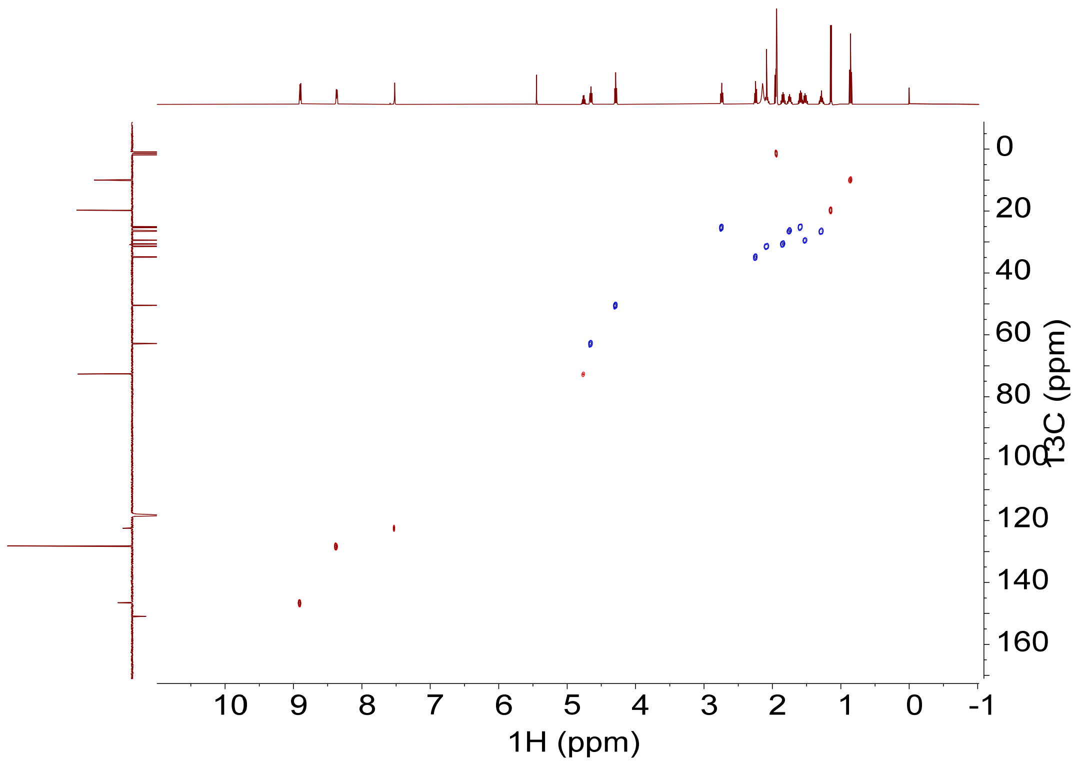

*Fig. S227. ^{1}H-^{13}C HSQC NMR spectrum of (R,R)-**16** (500 MHz, CD_3CN, 298 K).*

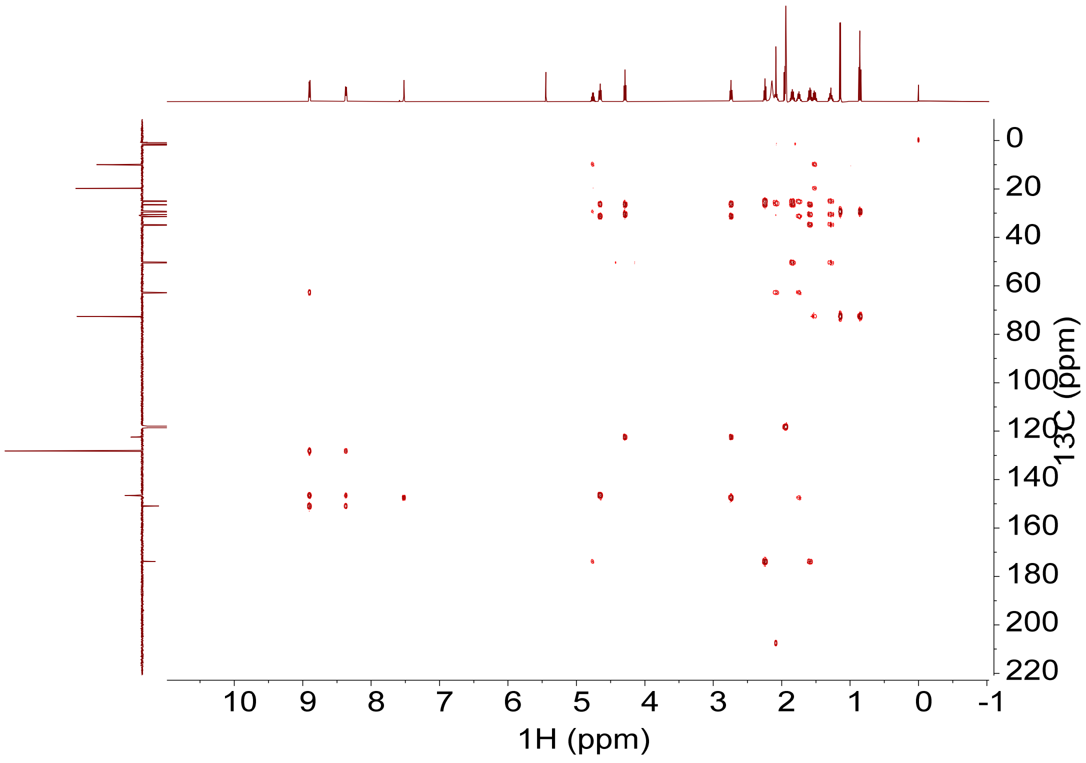

*Fig. S228. ^{1}H-^{13}C HMBC NMR spectrum of (R,R)-**16** (500 MHz, CD_3CN, 298 K).*

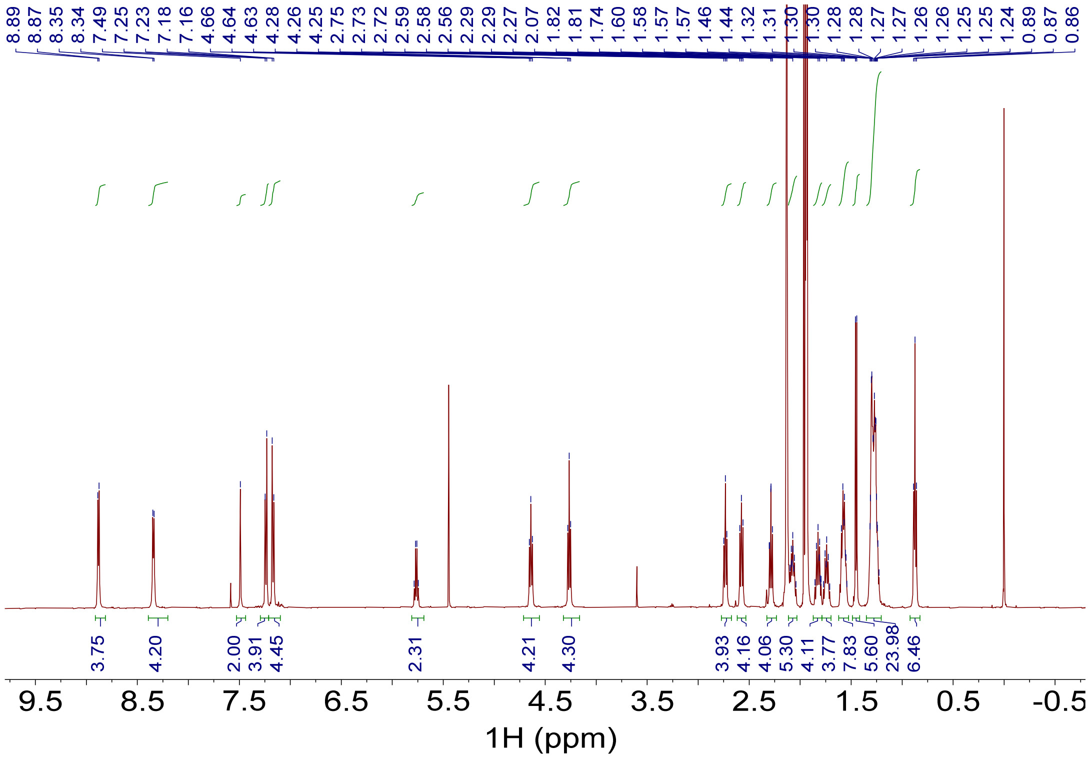

*Fig. S229. ^1H NMR spectrum of (R,R)-**17** (500 MHz, CD_3CN, 298 K).*

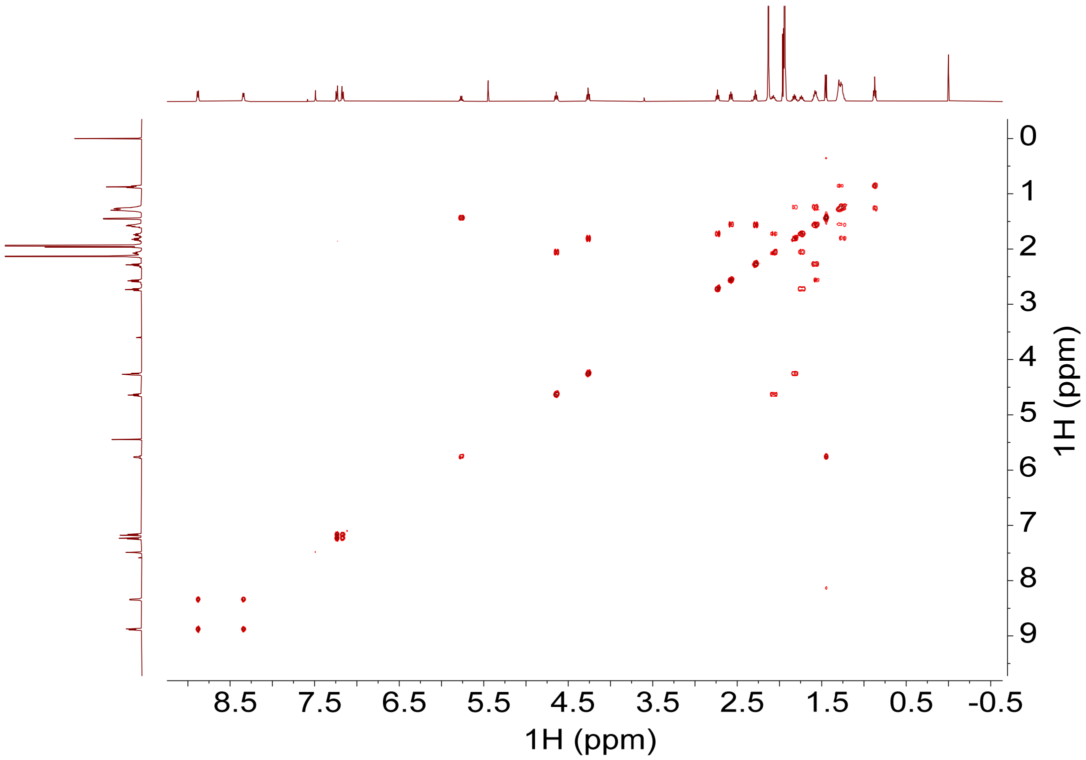

*Fig. S230. ^1H-^1H COSY NMR spectrum of (R,R)-**17** (500 MHz, CD_3CN, 298 K).*

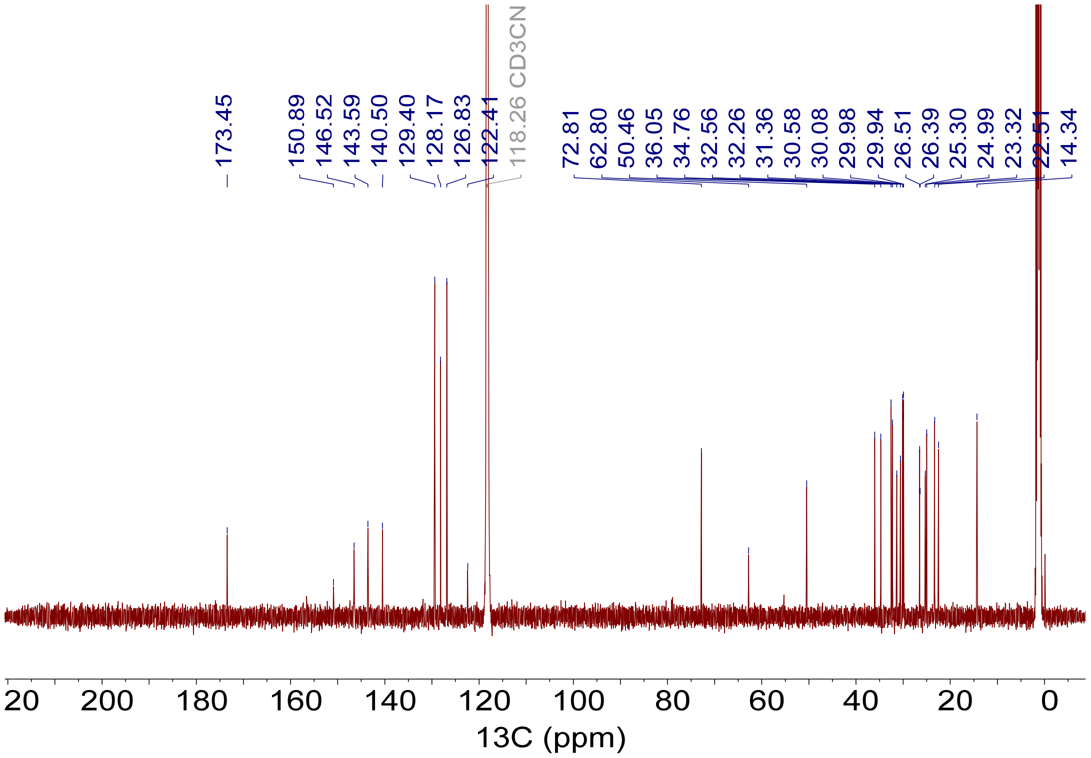

Fig. S231. ^{13}*C NMR spectrum of (R,R)-**17** (CPD, 126 MHz,* CD_3CN*, 298 K).*

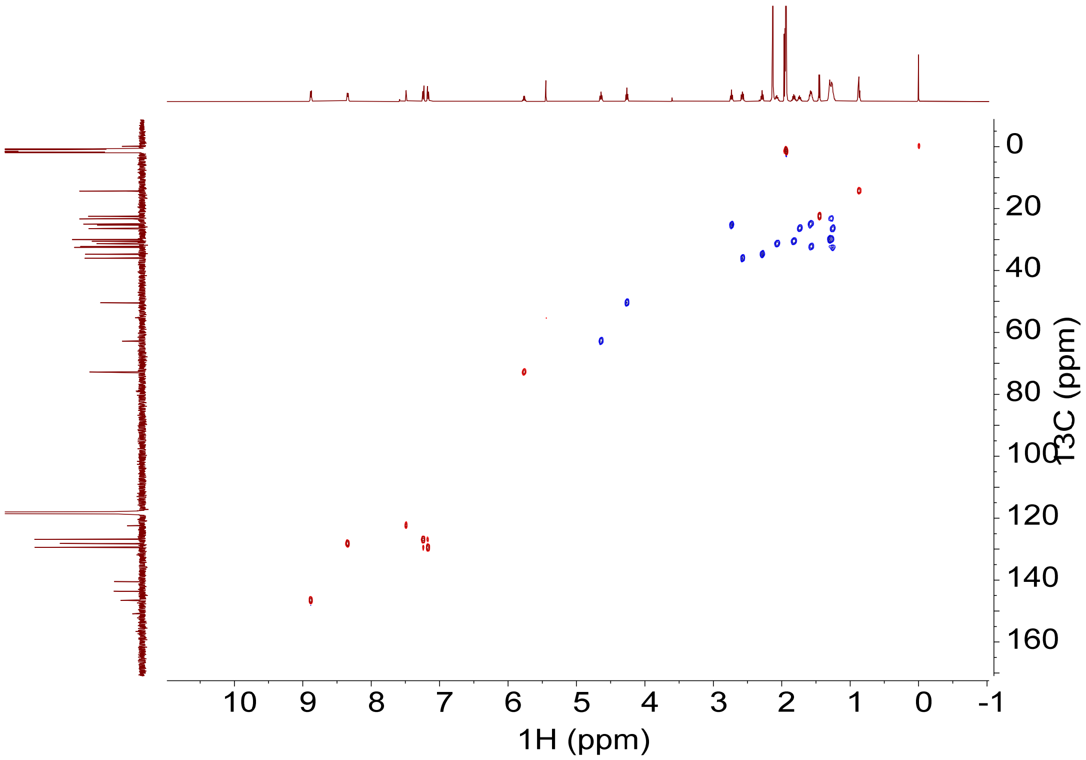

Fig. S232. ^{1}*H-*^{13}*C HSQC NMR spectrum of (R,R)-**17** (500 MHz,* CD_3CN*, 298 K).*

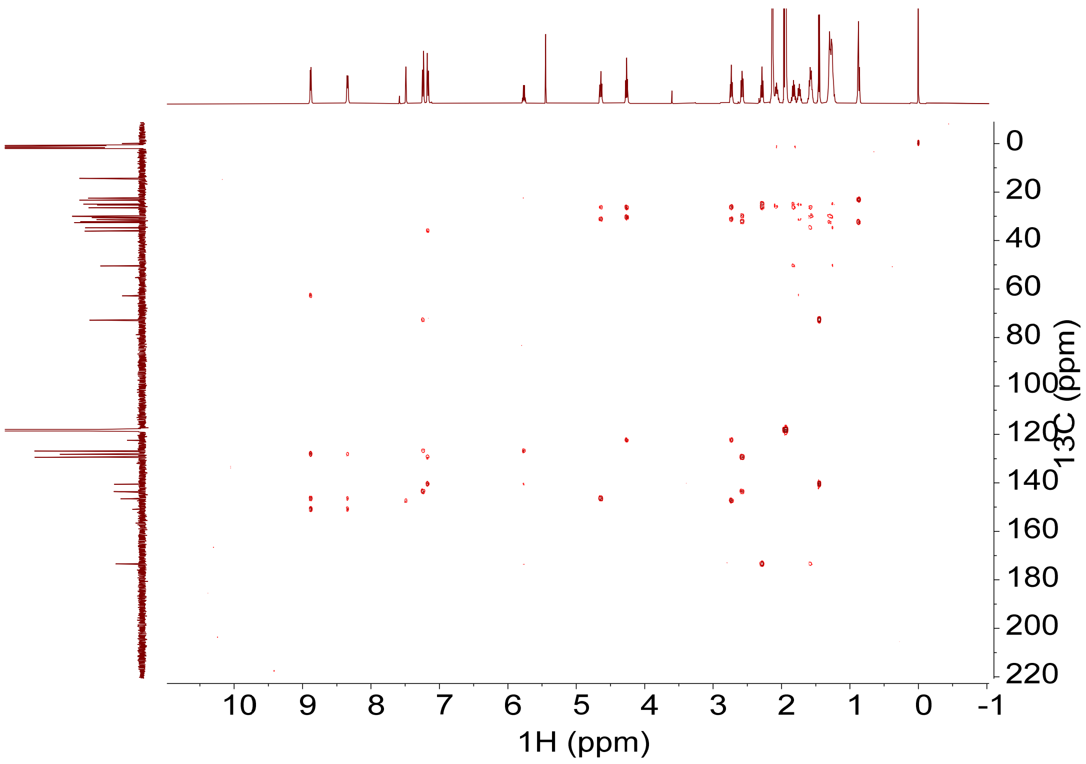

*Fig. S233. ^{1}H-^{13}C HMBC NMR spectrum of (R,R)-**17** (500 MHz, CD_3CN, 298 K).*

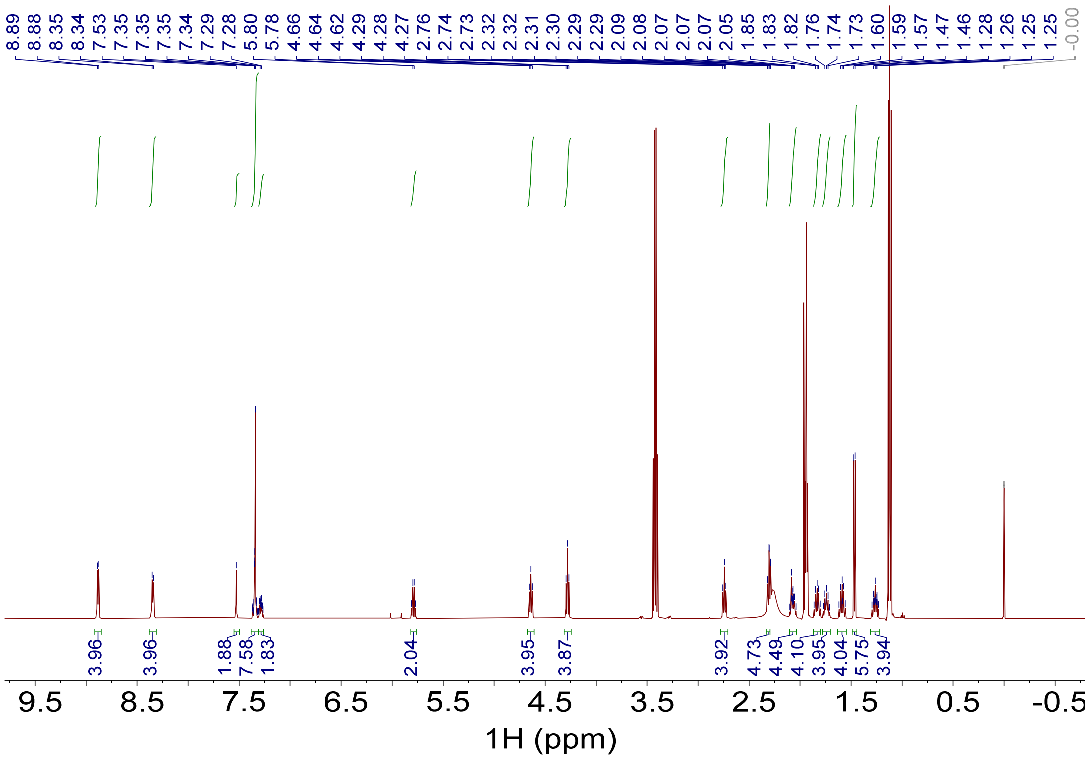

*Fig. S234. ^{1}H NMR spectrum of (S,S)-**18** (500 MHz, CD_3CN, 298 K).*

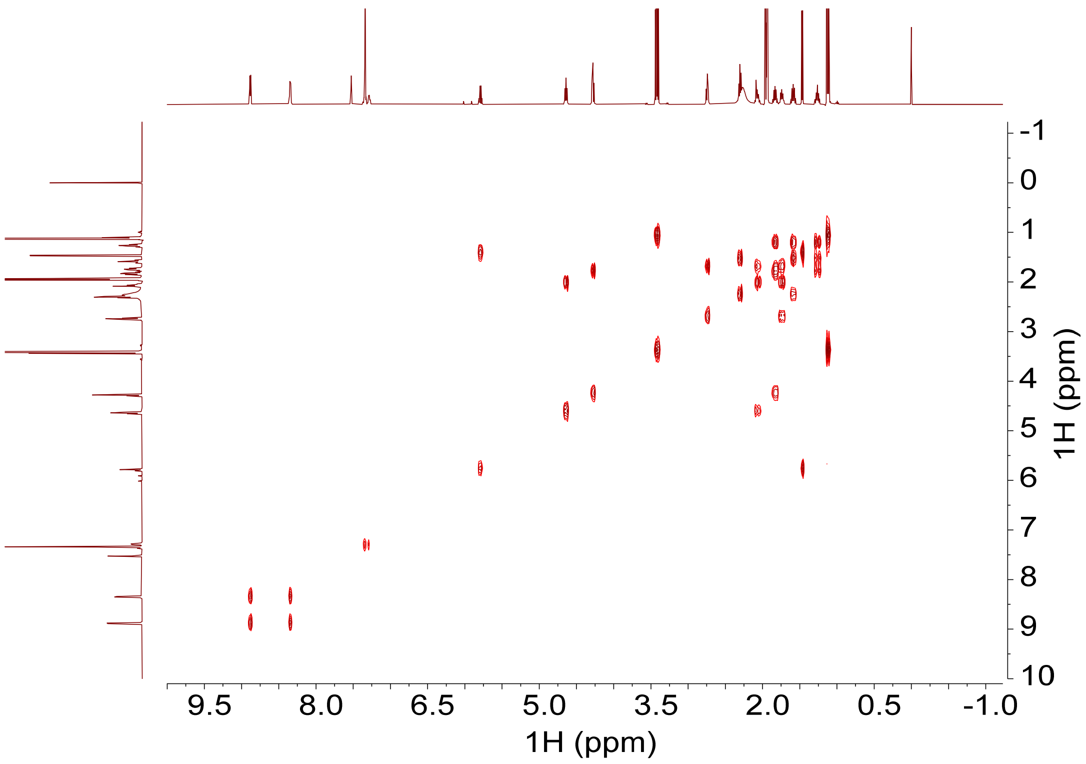

Fig. S235. [1]*H-*[1]*H COSY NMR spectrum of (S,S)-**18** (500 MHz, CD_3CN, 298 K).*

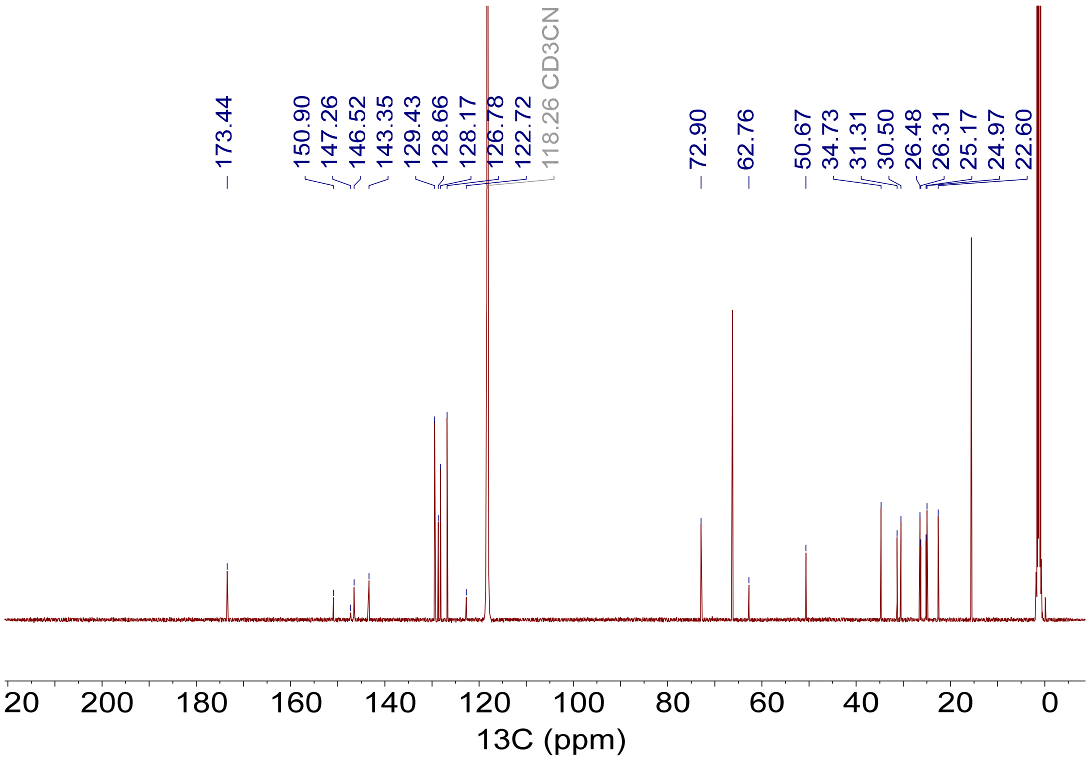

Fig. S236. [13]*C NMR spectrum of (S,S)-**18** (CPD, 126 MHz, CD_3CN, 298 K).*

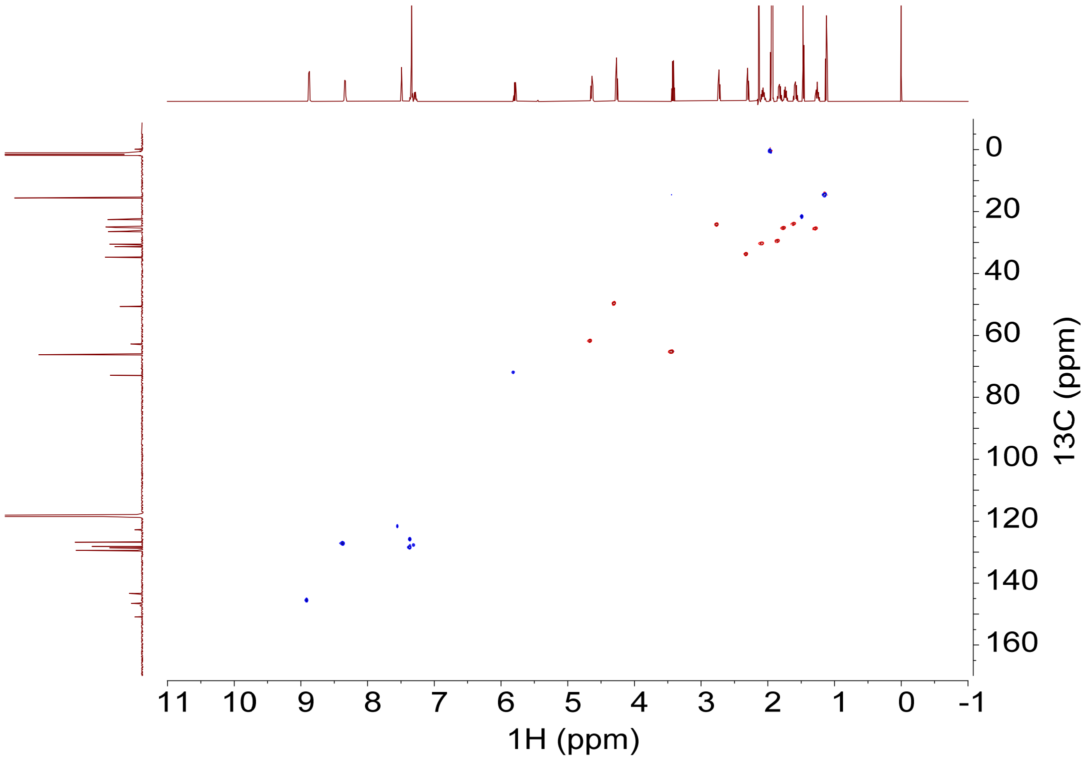

*Fig. S237. ¹H-¹³C HSQC NMR spectrum of (S,S)-**18** (500 MHz, CD_3CN, 298 K).*

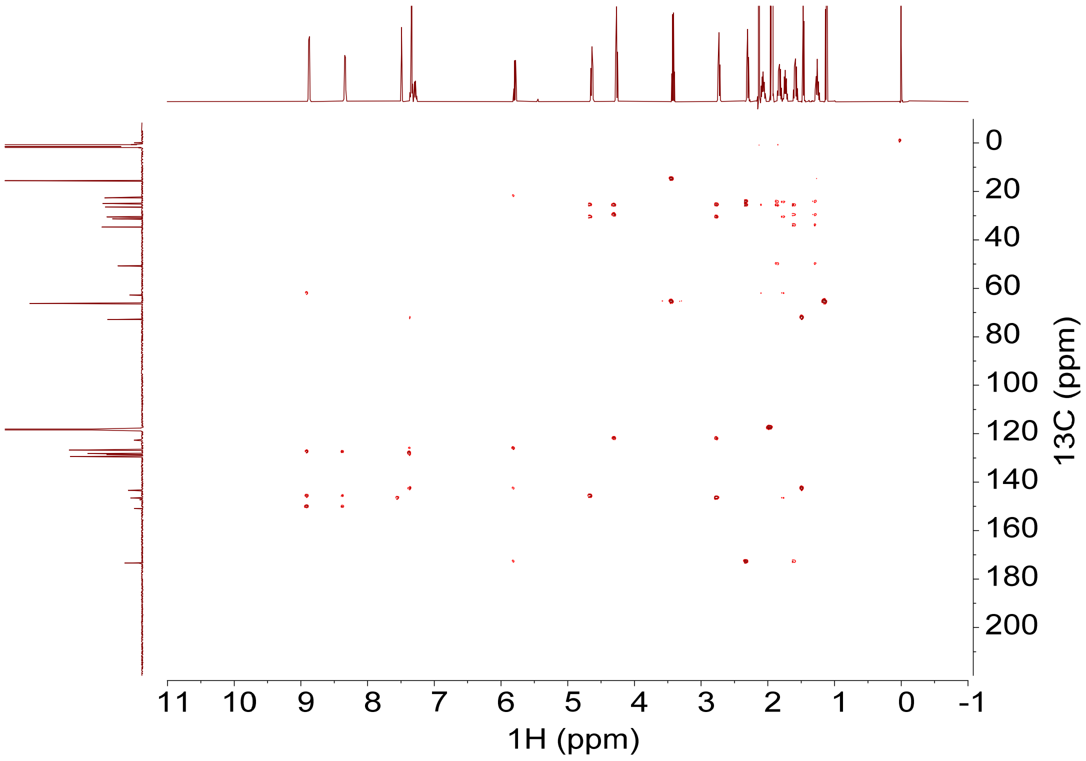

*Fig. S238. ¹H-¹³C HMBC NMR spectrum of (S,S)-**18** (500 MHz, CD_3CN, 298 K).*

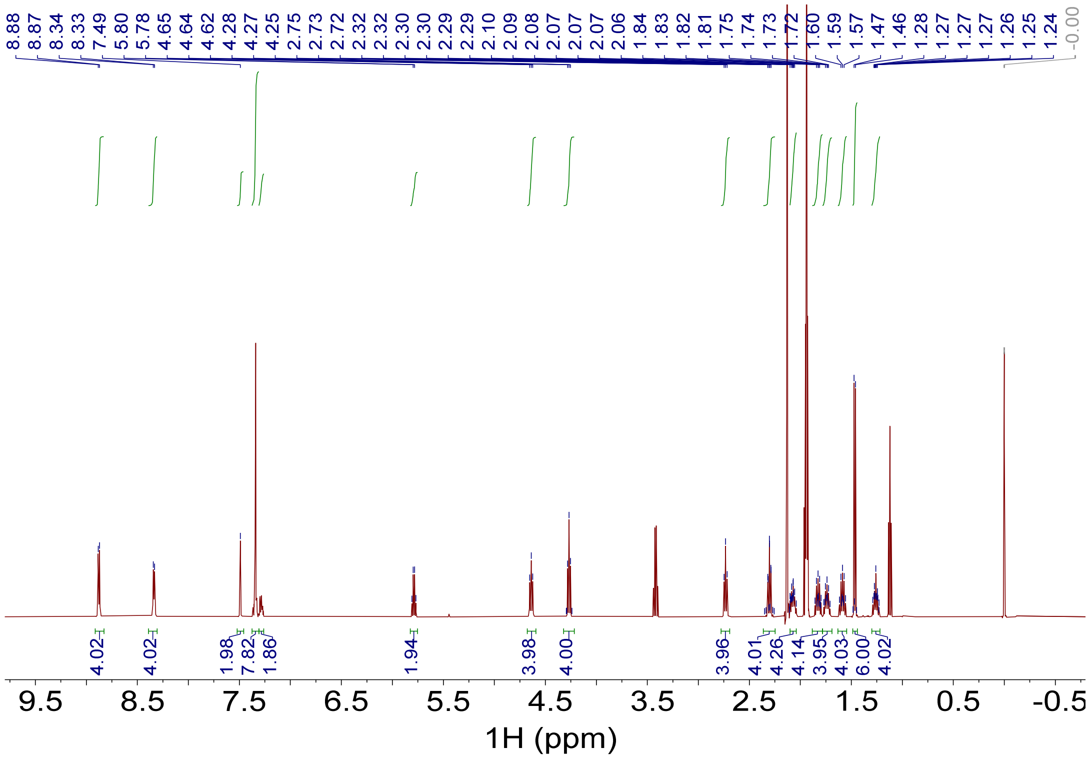

*Fig. S239. ^{1}H NMR spectrum of (R,R)-**18** (500 MHz, CD_3CN, 298 K).*

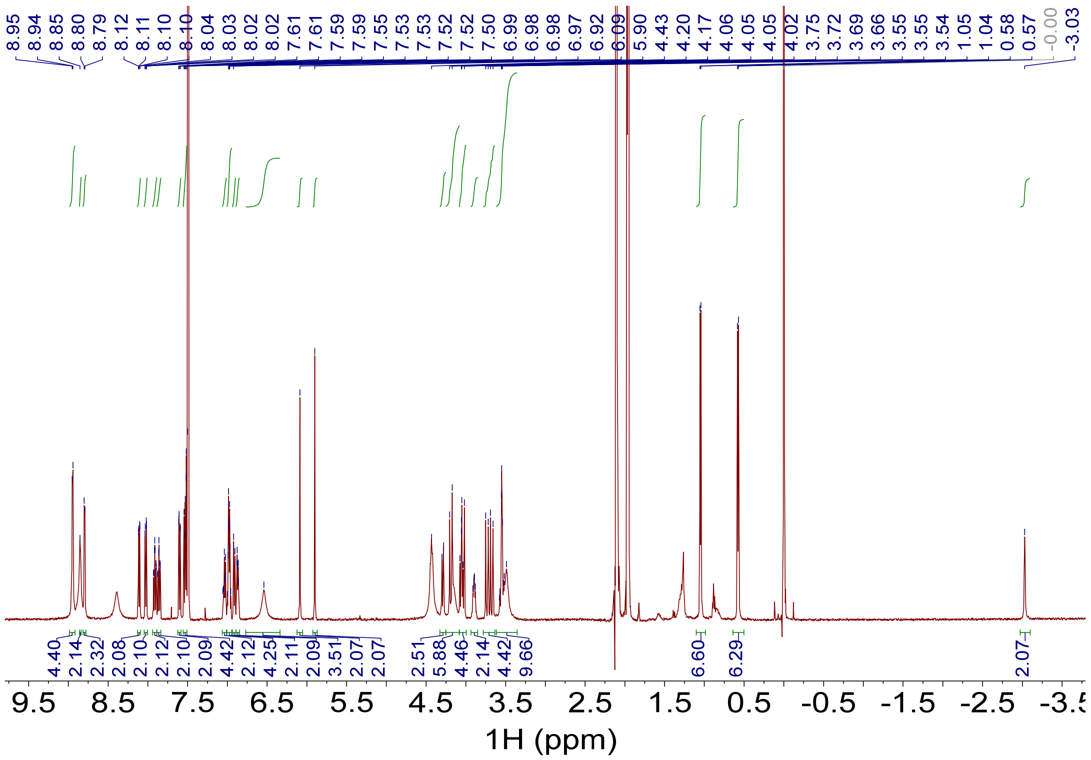

*Fig. S240. ^{1}H NMR spectrum of (R,R,R,R)-**$H_2$3·13** (500 MHz, $CDCl_3$/CD_3CN, 1:1, v/v, 298 K).*

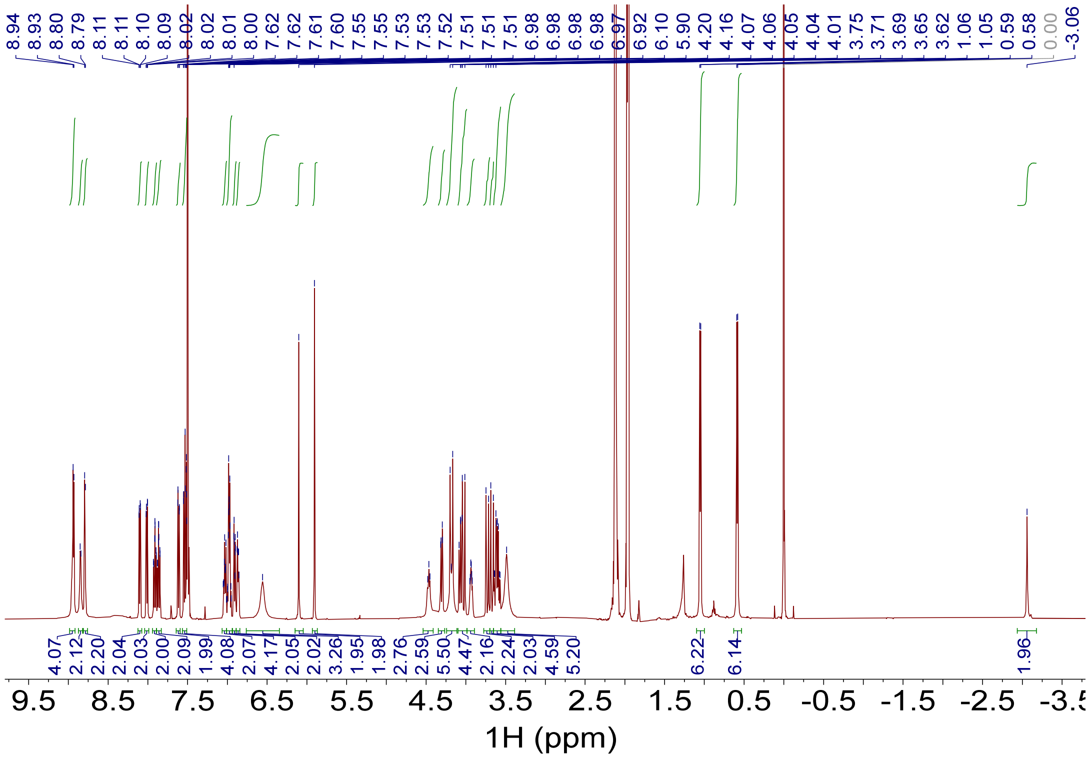

Fig. S241. *^{1}H NMR spectrum of (S,S,S,S)-**$H_2$3·13** (500 MHz, $CDCl_3$/CD_3CN, 1:1, v/v, 298 K).*

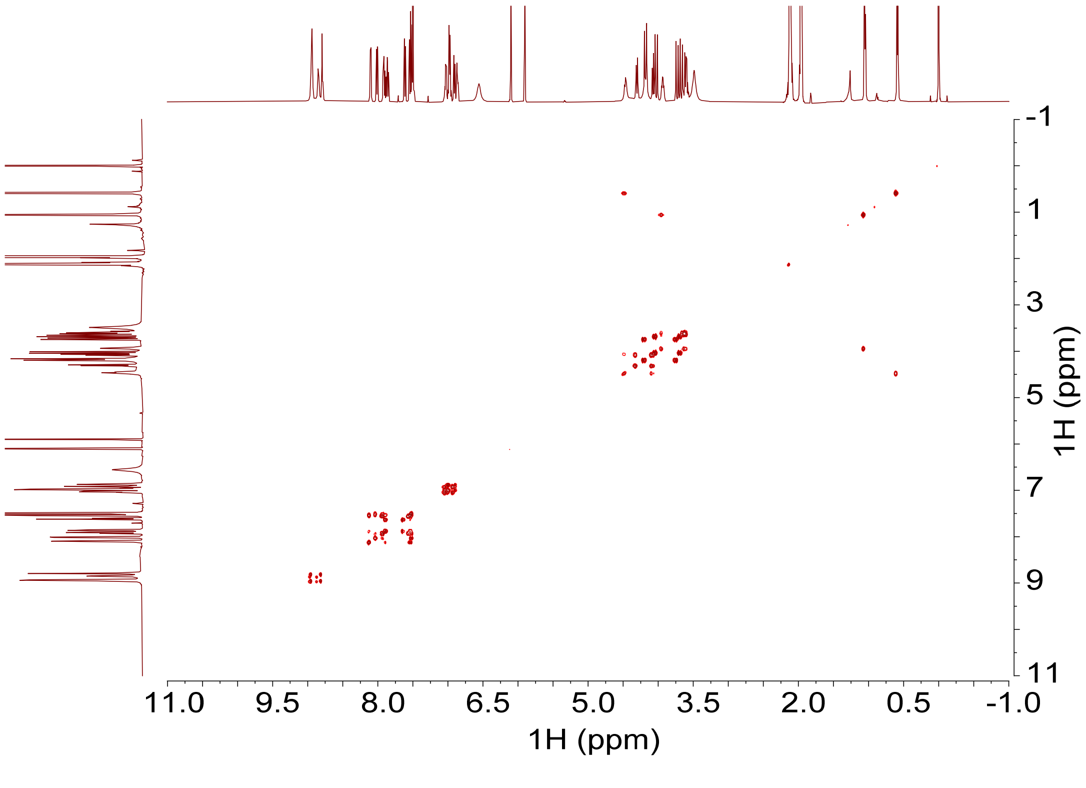

Fig. S242. *^{1}H-^{1}H COSY NMR spectrum of (S,S,S,S)-**$H_2$3·13** (500 MHz, $CDCl_3$/CD_3CN, 1:1, v/v, 298 K).*

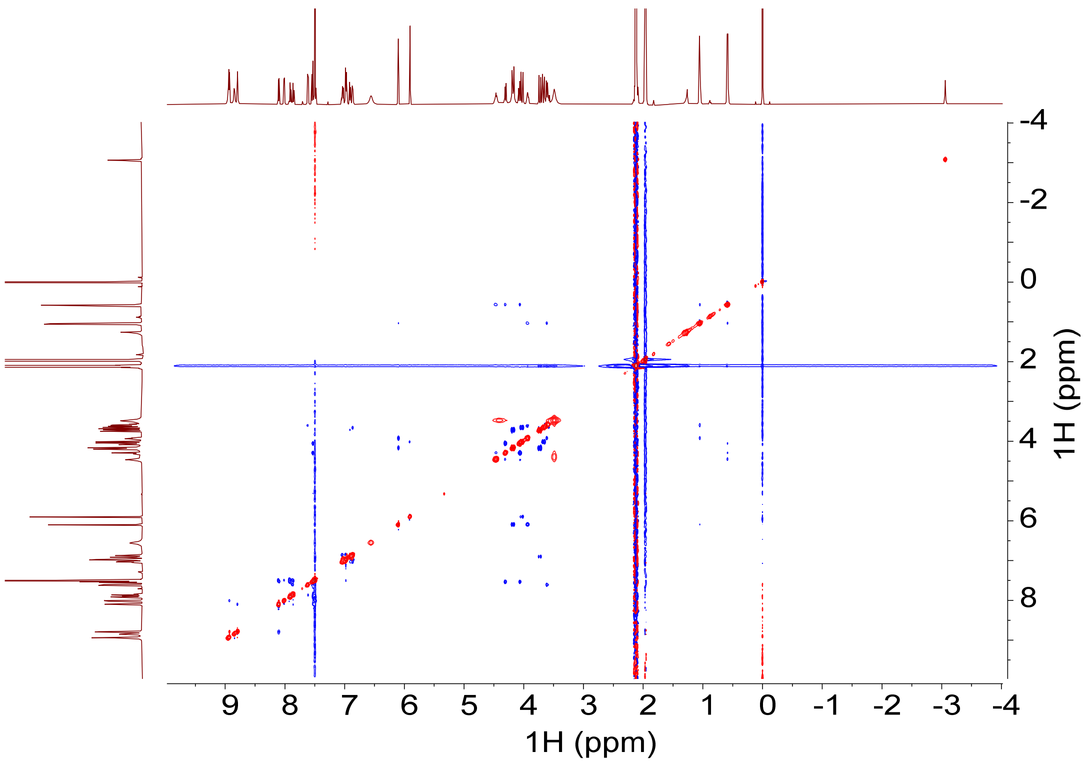

Fig. S243. ^{1}H-^{1}H *ROESY NMR spectrum of (S,S,S,S)-**$H_2$3·13** (500 MHz, $CDCl_3$/CD_3CN, 1:1, v/v, 298 K).*

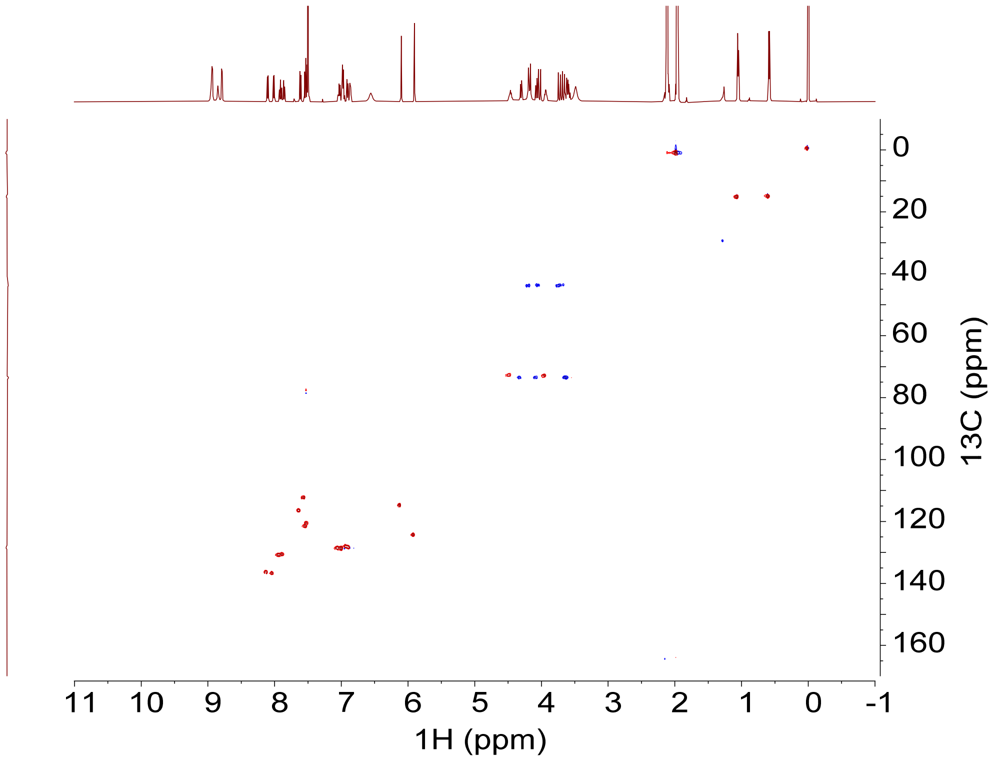

Fig. S244. ^{1}H-^{13}C *HSQC NMR spectrum of (S,S,S,S)-**$H_2$3·13** (500 MHz, $CDCl_3$/CD_3CN, 1:1, v/v, 298 K).*

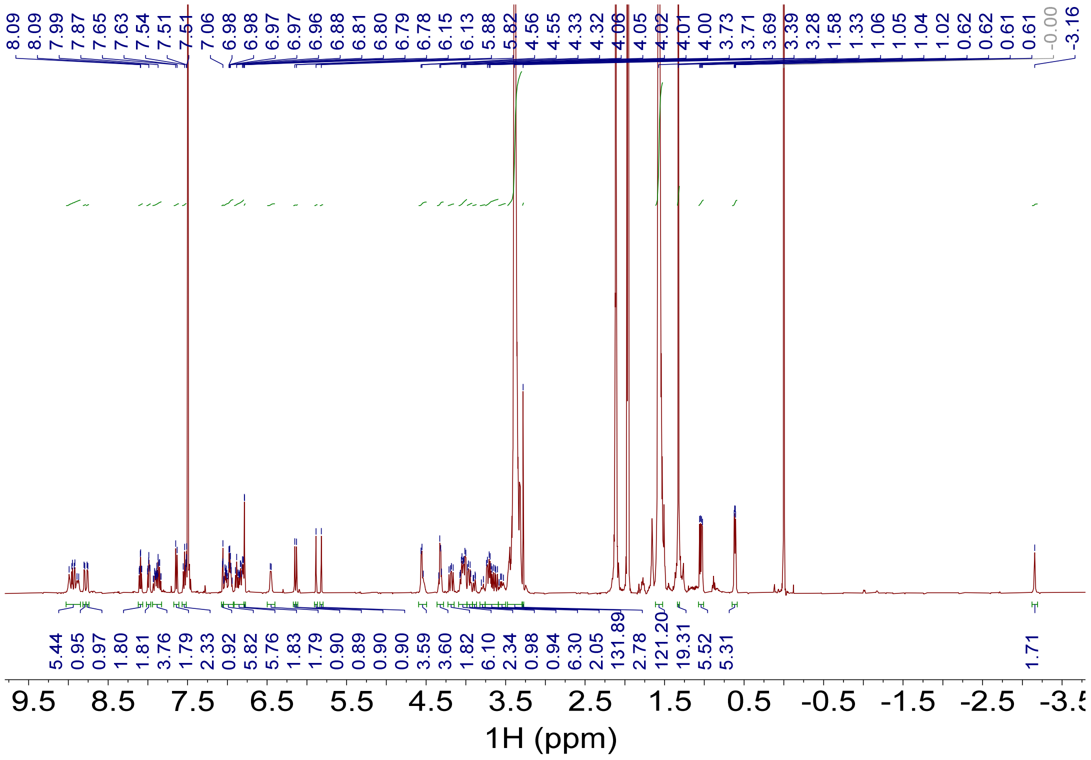

*Fig. S245. ^{1}H NMR spectrum of (R,R,R,R)-**$H_2$3·14** (500 MHz, $CDCl_3$/CD_3CN, 1:1, v/v, 298 K).*

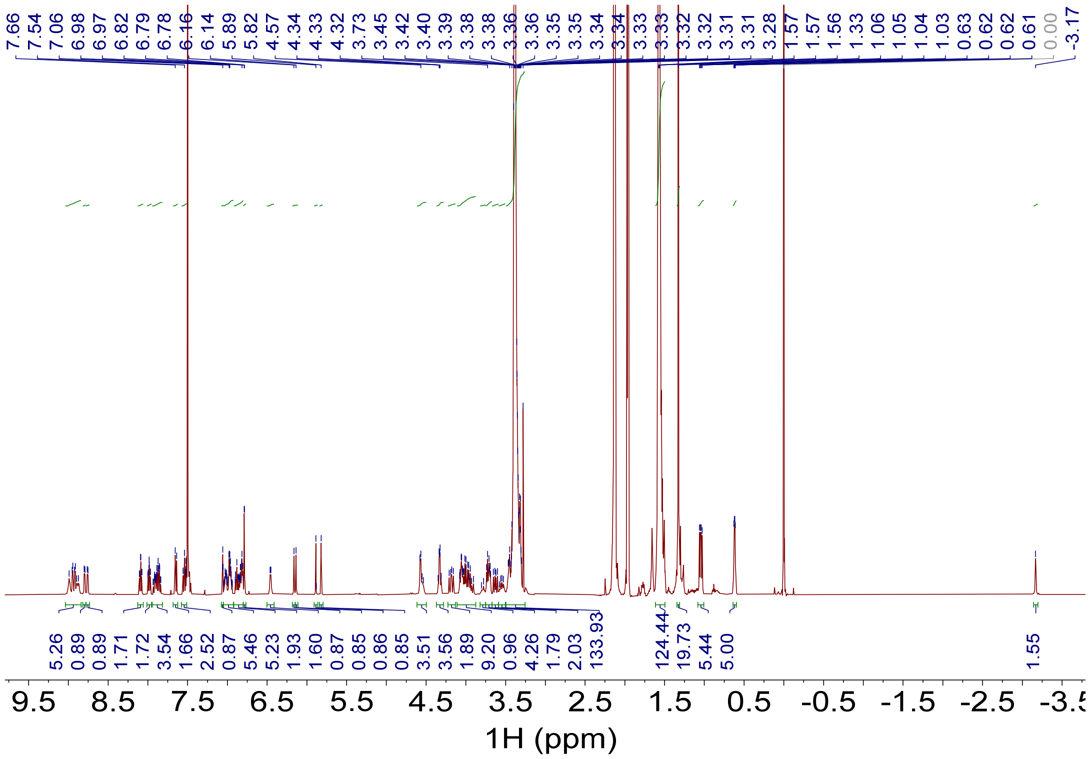

*Fig. S246. ^{1}H NMR spectrum of (S,S,S,S)-**$H_2$3·14** (500 MHz, $CDCl_3$/CD_3CN, 1:1, v/v, 298 K).*

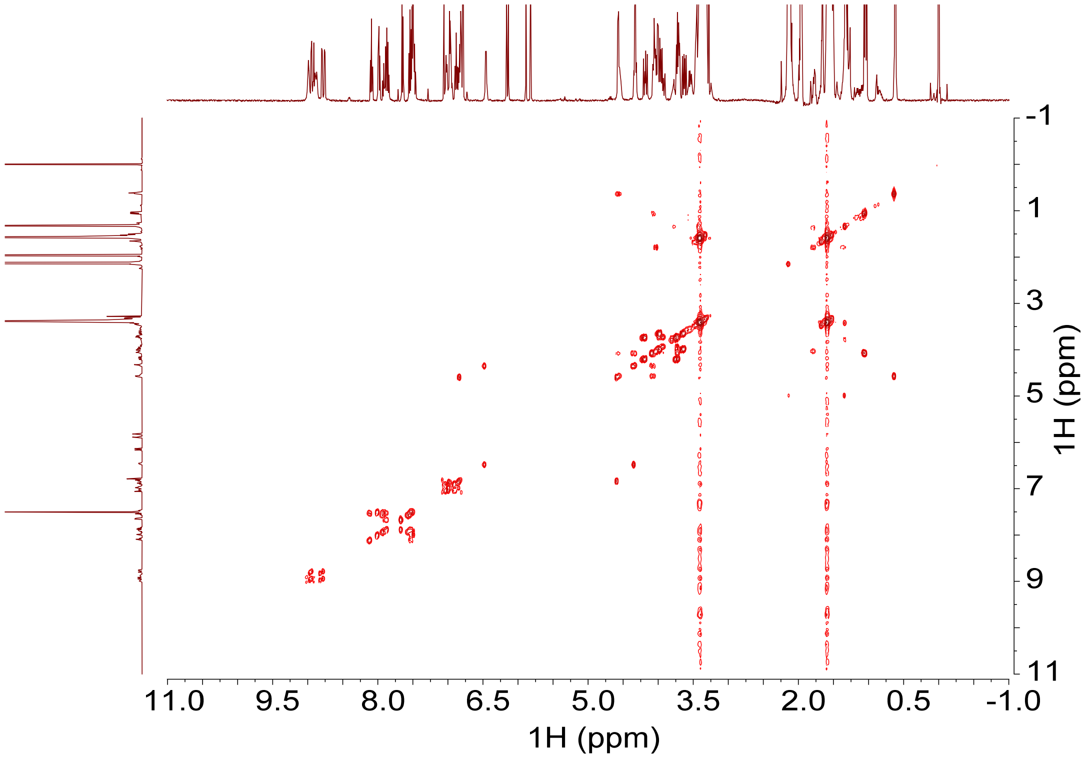

Fig. S247. [1]*H-*[1]*H COSY NMR spectrum of (S,S,S,S)-****$H_2$3·14*** *(500 MHz, $CDCl_3$/CD_3CN, 1:1, v/v, 298 K).*

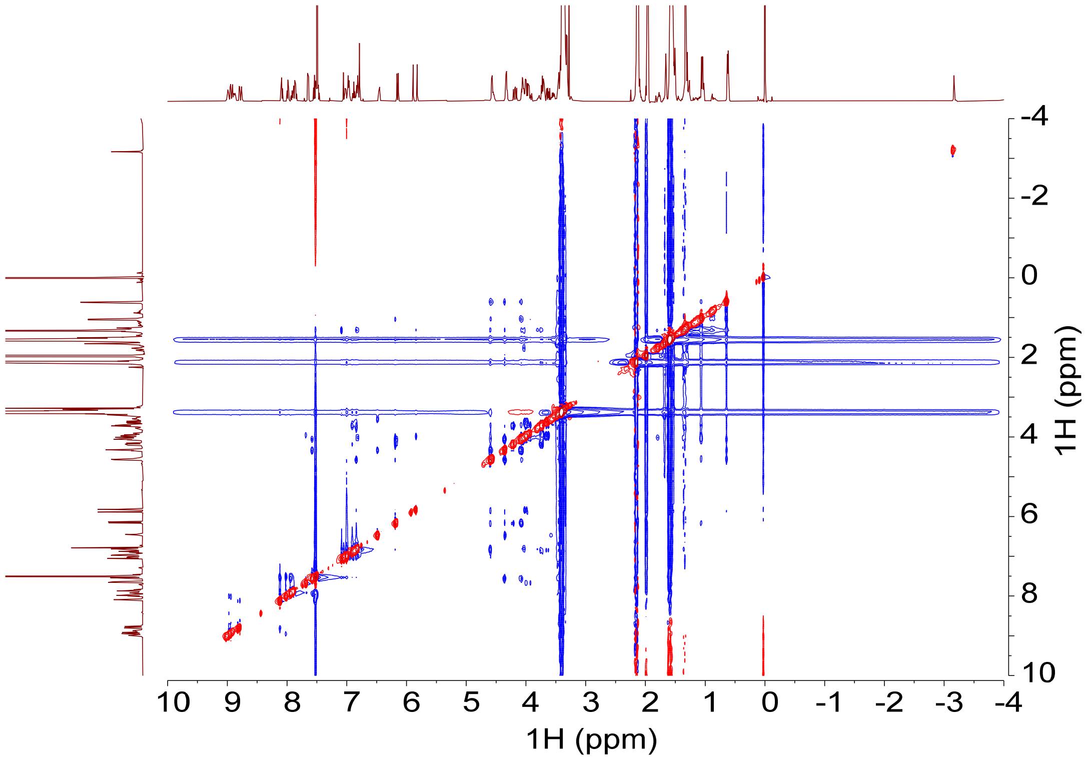

Fig. S248. [1]*H-*[1]*H ROESY NMR spectrum of (S,S,S,S)-****$H_2$3·14*** *(500 MHz, $CDCl_3$/CD_3CN, 1:1, v/v, 298 K).*

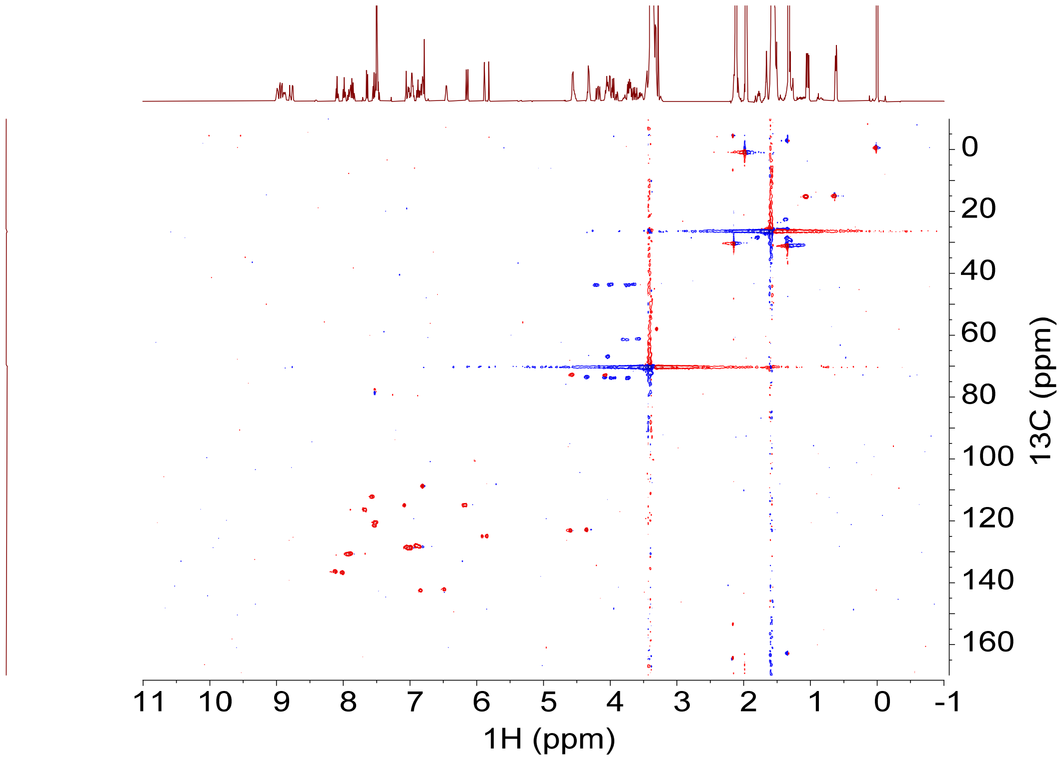

*Fig. S249. ^{1}H-^{13}C HSQC NMR spectrum of (S,S,S,S)-**$H_2$3·14** (500 MHz, $CDCl_3$/CD_3CN, 1:1, v/v, 298 K).*

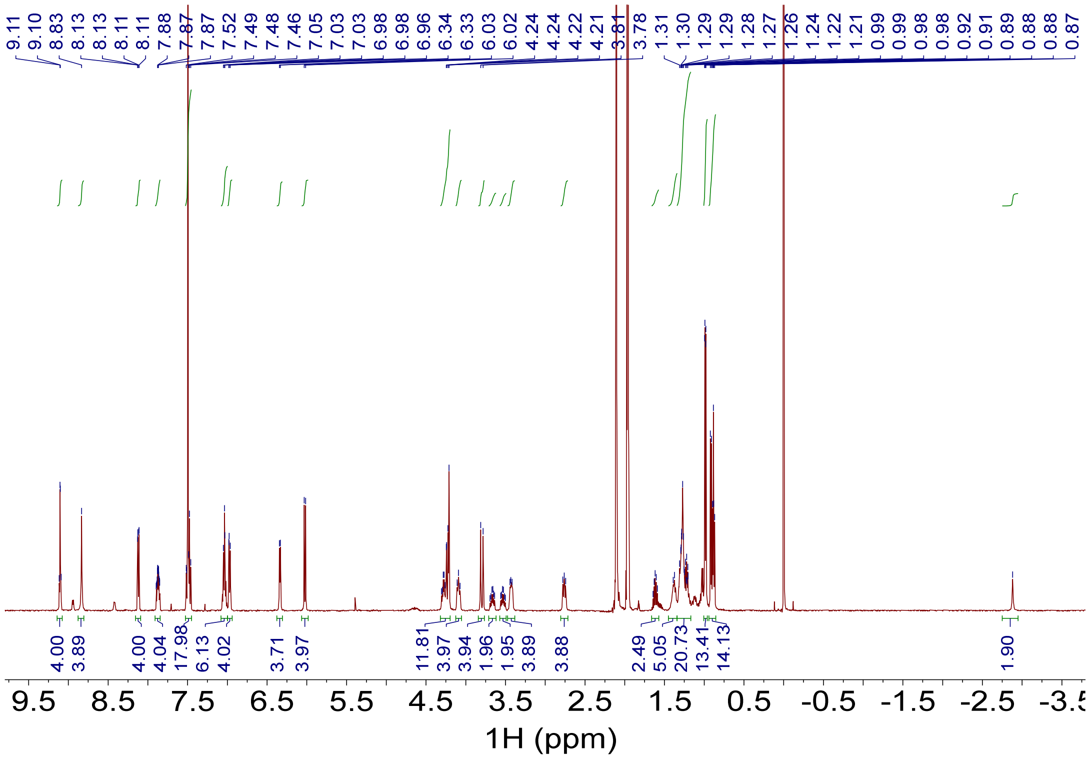

*Fig. S250. ^{1}H NMR spectrum of **$H_2$1·**(R,R)-**15** (500 MHz, $CDCl_3$/CD_3CN, 1:1, v/v, 298 K).*

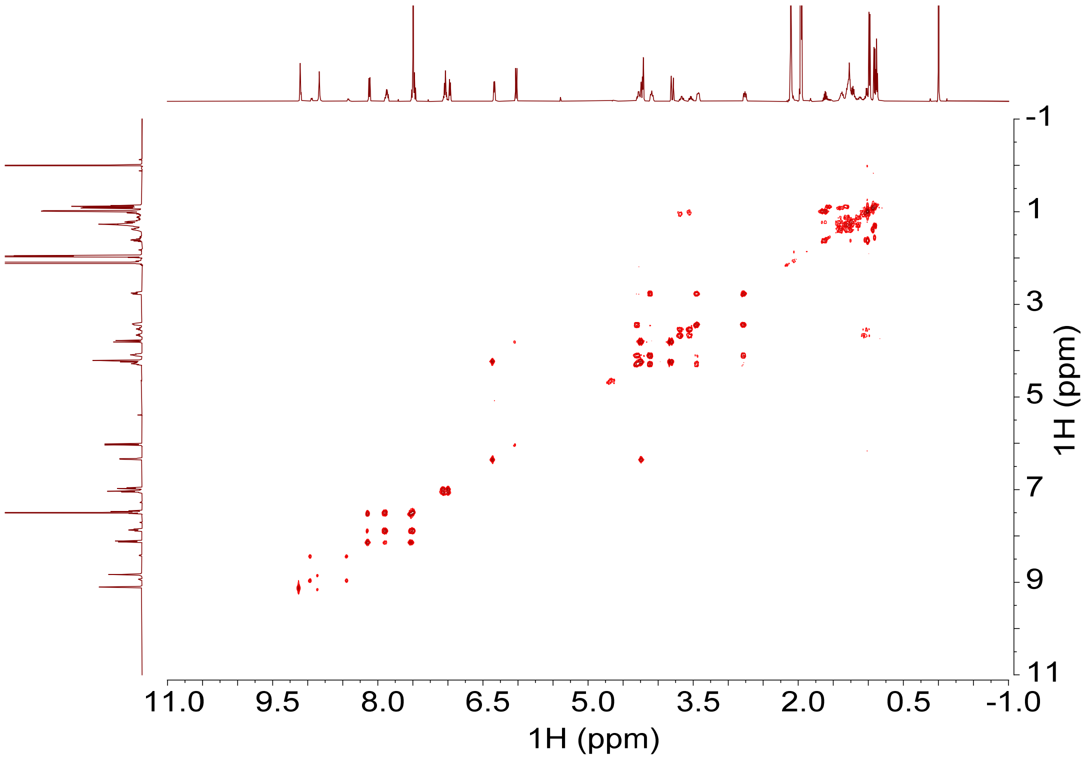

*Fig. S251. ^1H-^1H COSY NMR spectrum of **$H_2$1·**(R,R)-**15** (500 MHz, $CDCl_3$/CD_3CN, 1:1, v/v, 298 K).*

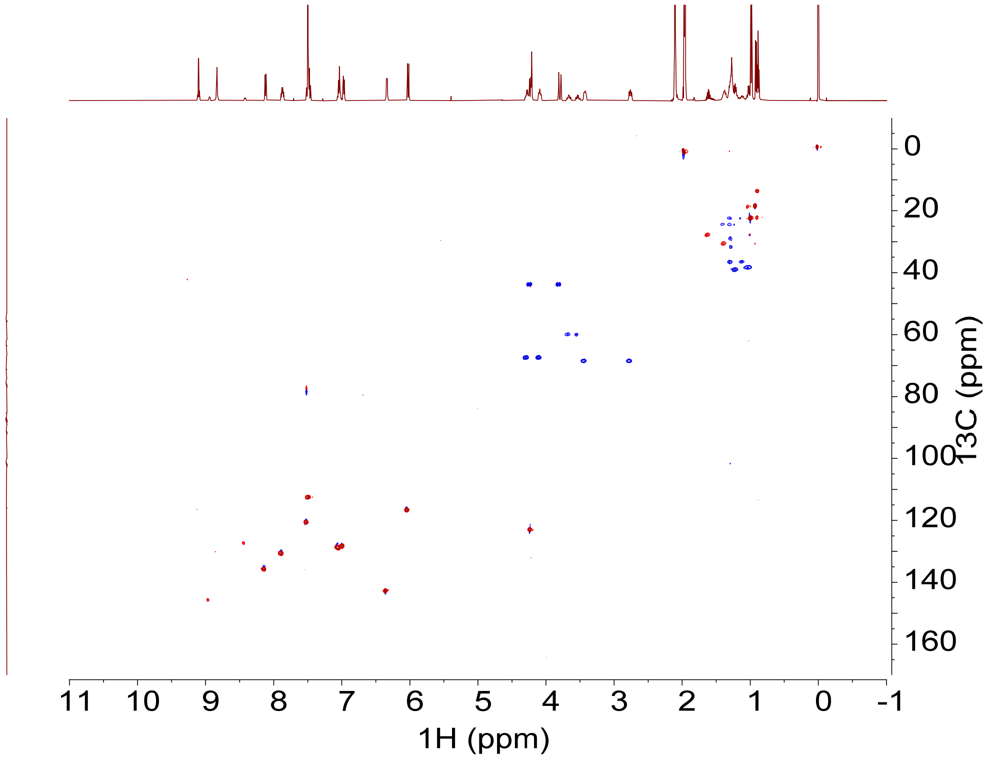

*Fig. S252. ^1H-^{13}C HSQC NMR spectrum of **$H_2$1·**(R,R)-**15** (500 MHz, $CDCl_3$/CD_3CN, 1:1, v/v, 298 K).*

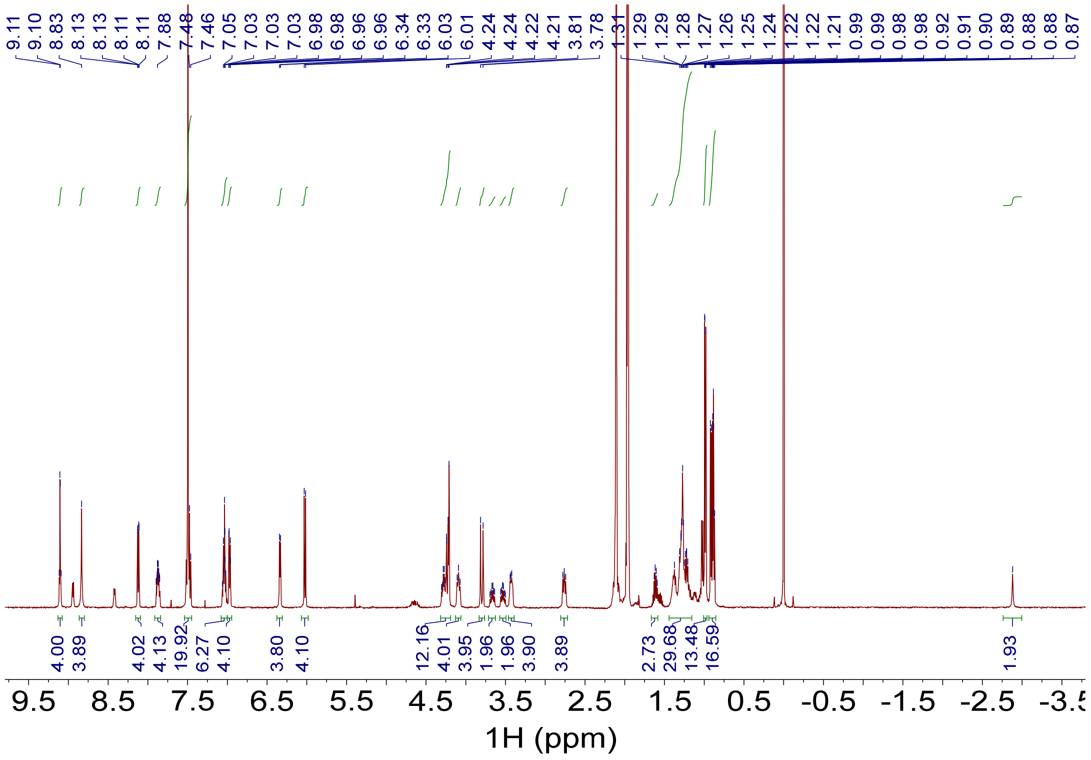

Fig. S253. ¹H NMR spectrum of ***$H_2$1***·*(S,S)-***15** *(500 MHz, $CDCl_3$/CD_3CN, 1:1, v/v, 298 K).*

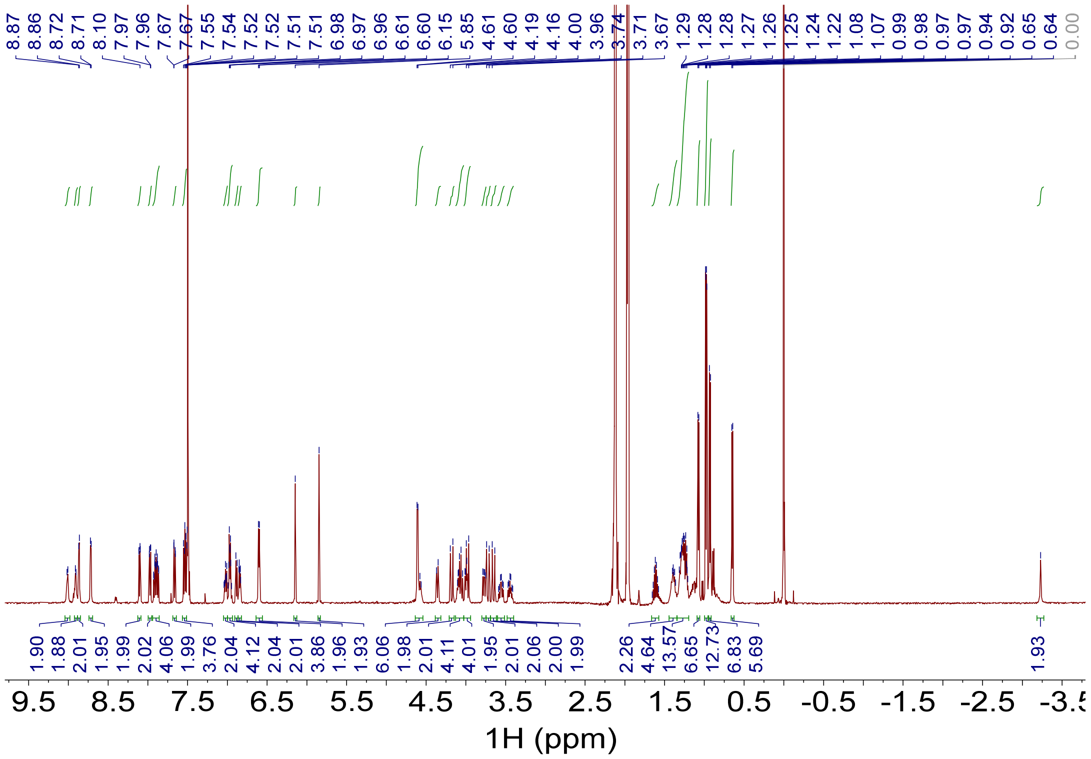

*Fig. S254. ¹H NMR spectrum of (R,R,R,R)-***$H_2$3***·(R,R)-***15** *(500 MHz, $CDCl_3$/CD_3CN, 1:1, v/v, 298 K).*

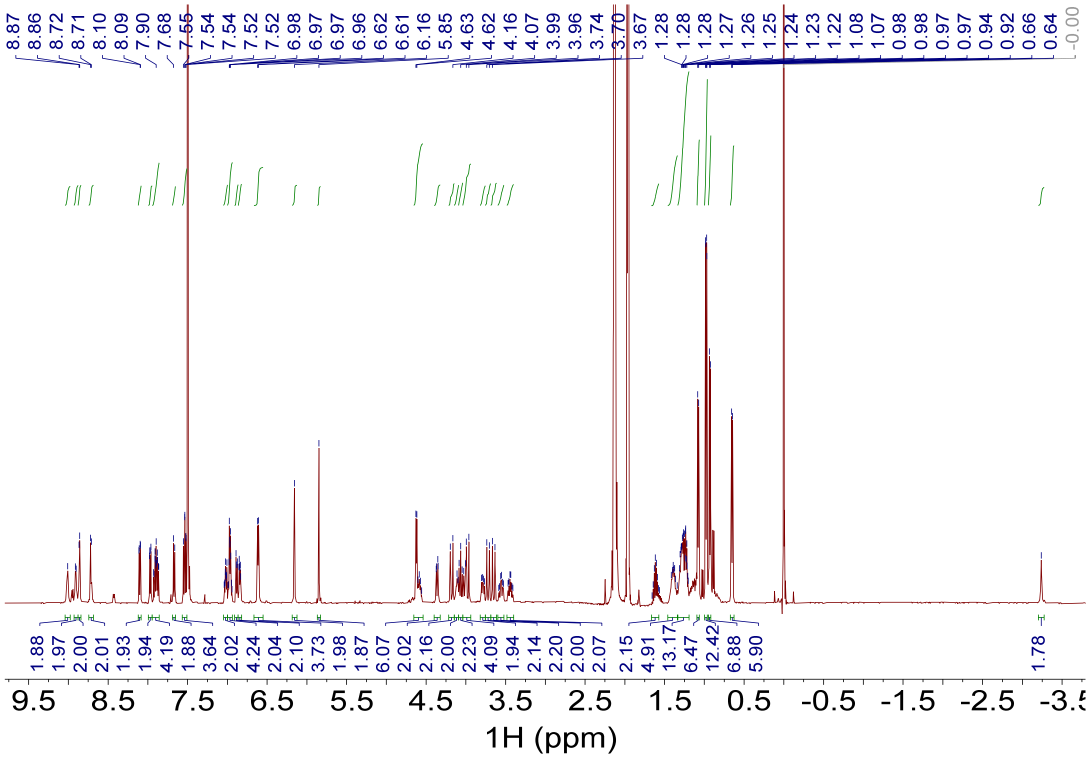

*Fig. S255. ^{1}H NMR spectrum of (S,S,S,S)-**$H_2$3**·(S,S)-**15** (500 MHz, $CDCl_3/CD_3CN$, 1:1, v/v, 298 K).*

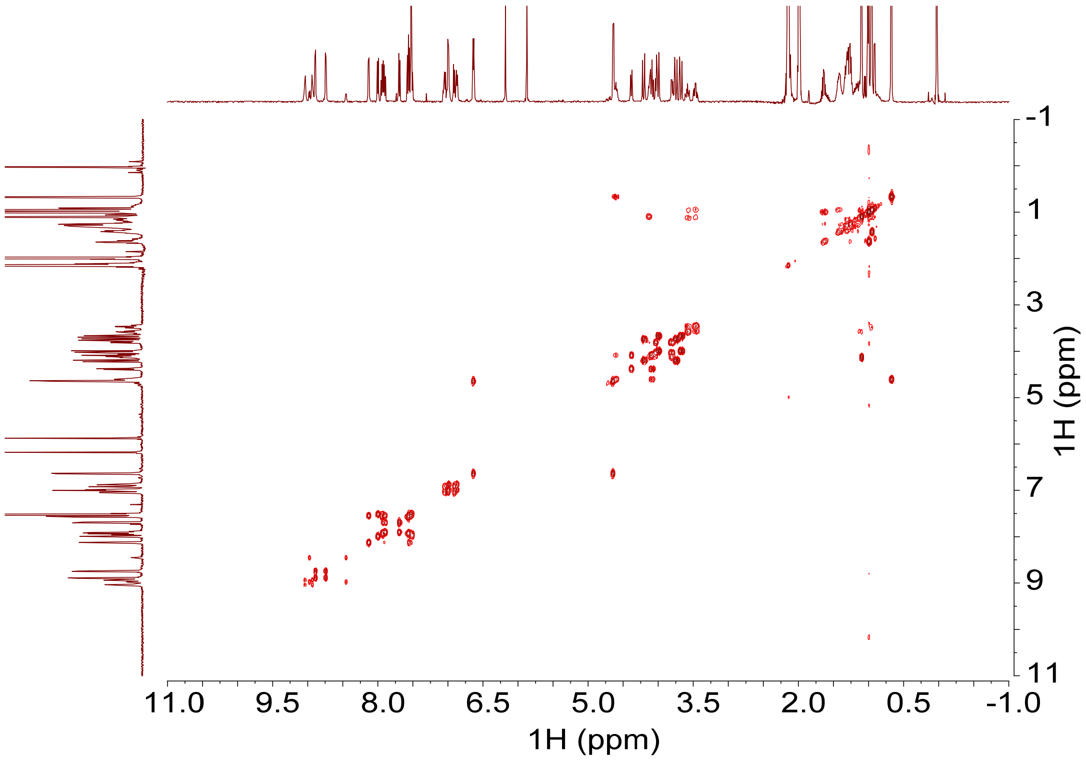

*Fig. S256. ^{1}H-^{1}H COSY NMR spectrum of (S,S,S,S)-**$H_2$3**·(S,S)-**15** (500 MHz, $CDCl_3/CD_3CN$, 1:1, v/v, 298 K).*

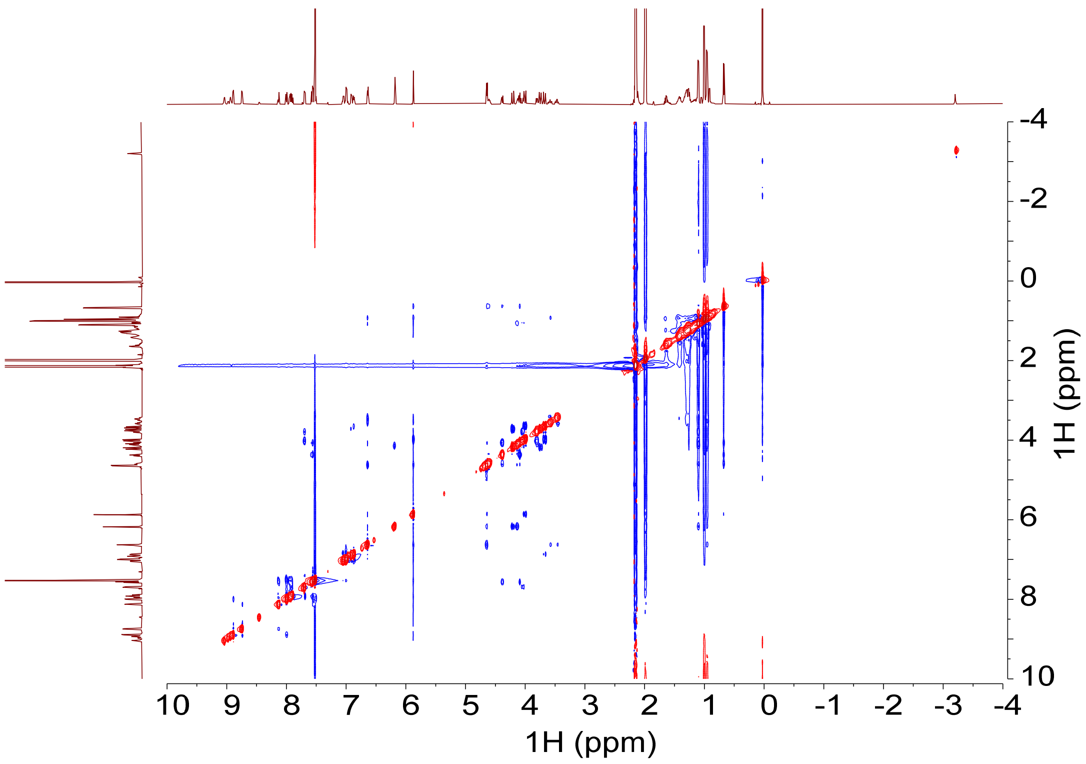

*Fig. S257. ^{1}H-^{1}H ROESY NMR spectrum of (S,S,S,S)-**$H_2$3**·(S,S)-**15** (500 MHz, $CDCl_3/CD_3CN$, 1:1, v/v, 298 K).*

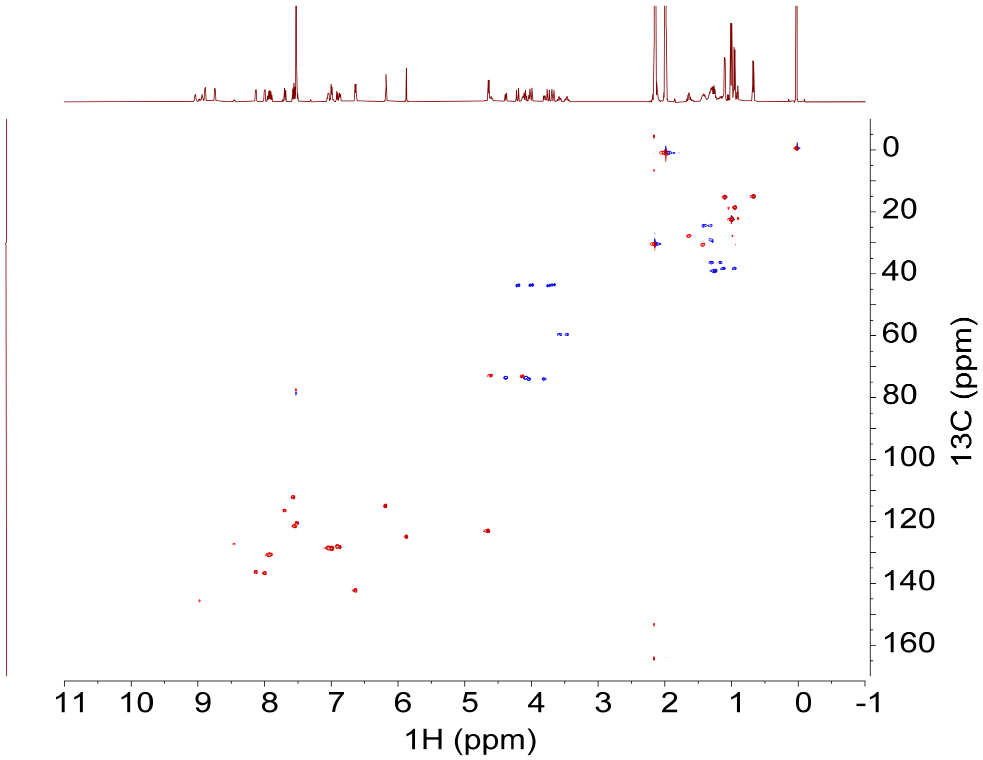

*Fig. S258. ^{1}H-^{13}C HSQC NMR spectrum of (S,S,S,S)-**$H_2$3**·(S,S)-**15** (500 MHz, $CDCl_3/CD_3CN$, 1:1, v/v, 298 K).*

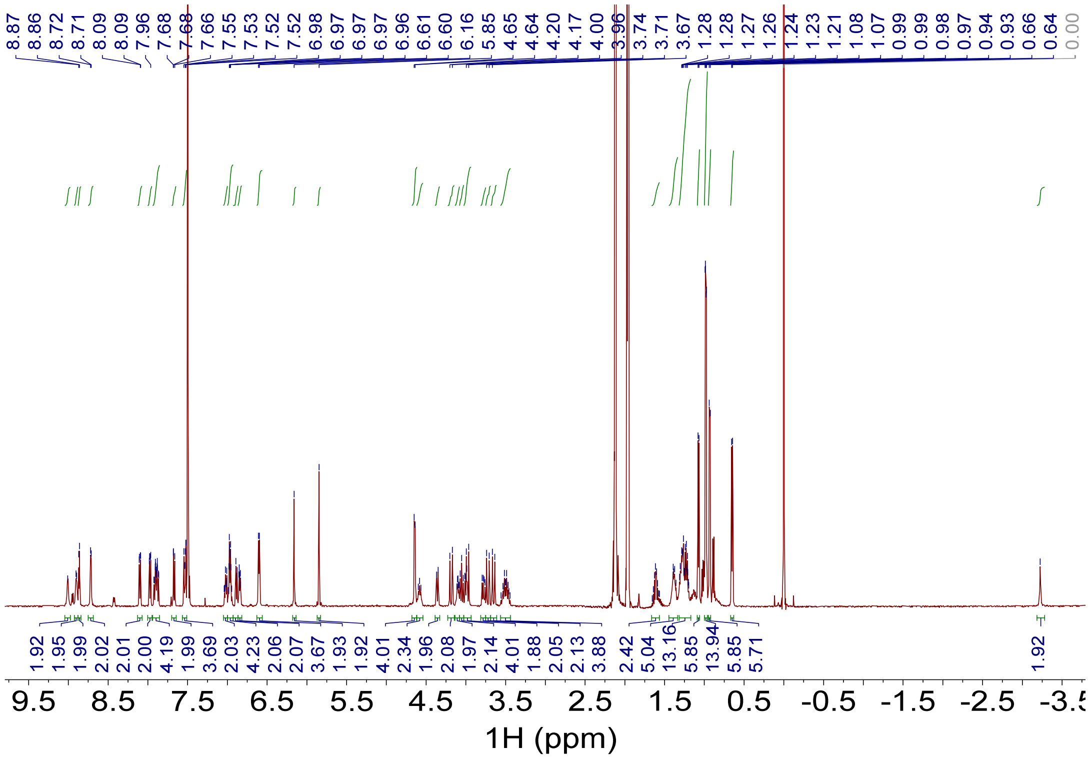

*Fig. S259. ^{1}H NMR spectrum of (R,R,R,R)-**$H_2$3**·(S,S)-**15** (500 MHz, $CDCl_3/CD_3CN$, 1:1, v/v, 298 K).*

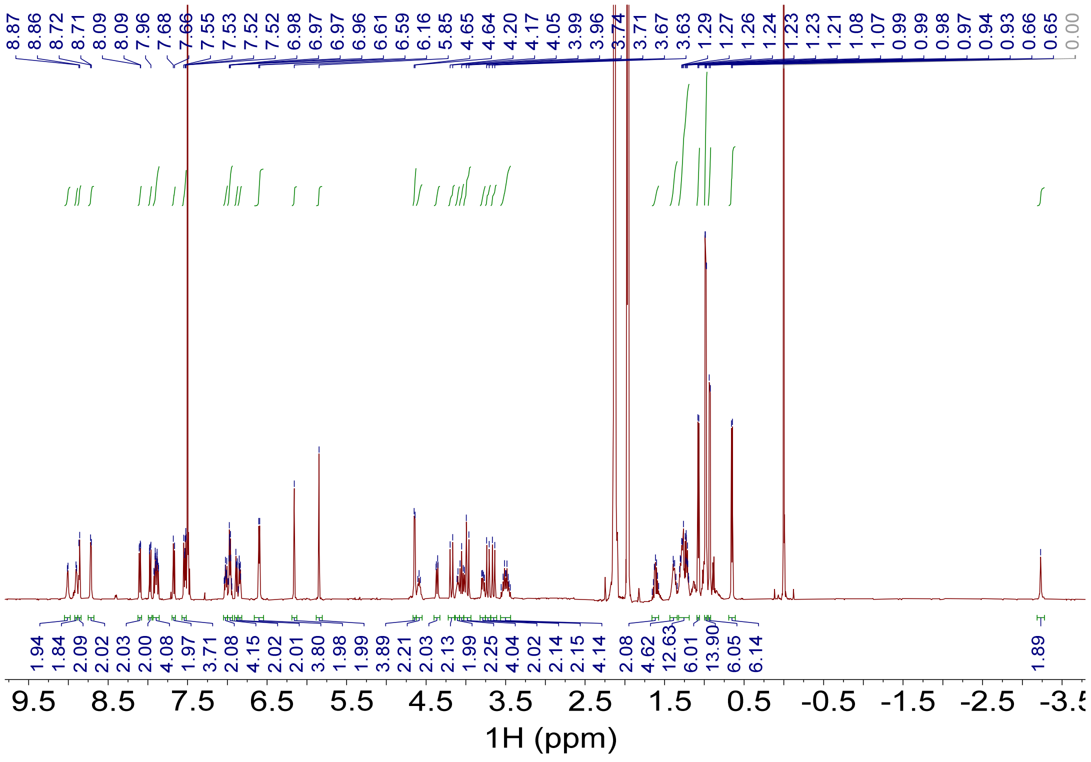

*Fig. S260. ^{1}H NMR spectrum of (S,S,S,S)-**$H_2$3**·(R,R)-**15** (500 MHz, $CDCl_3/CD_3CN$, 1:1, v/v, 298 K).*

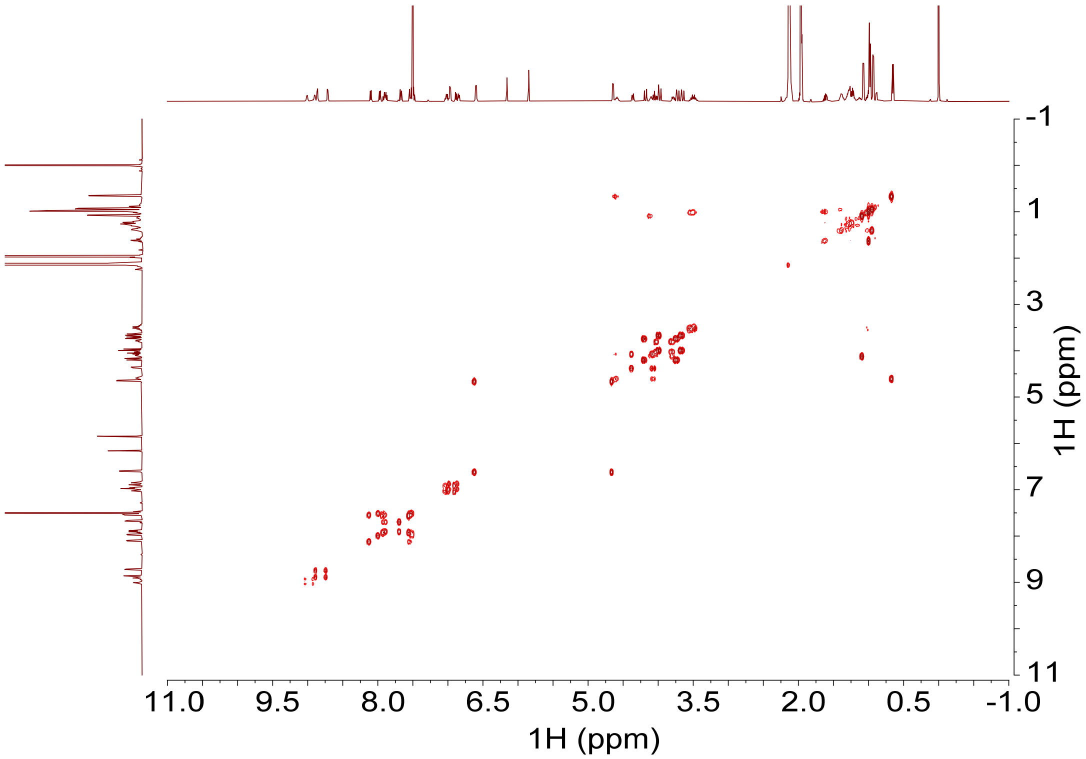

*Fig. S261. ^{1}H-^{1}H COSY NMR spectrum of (S,S,S,S)-**$H_2$3**·(R,R)-**15** (500 MHz, $CDCl_3/CD_3CN$, 1:1, v/v, 298 K).*

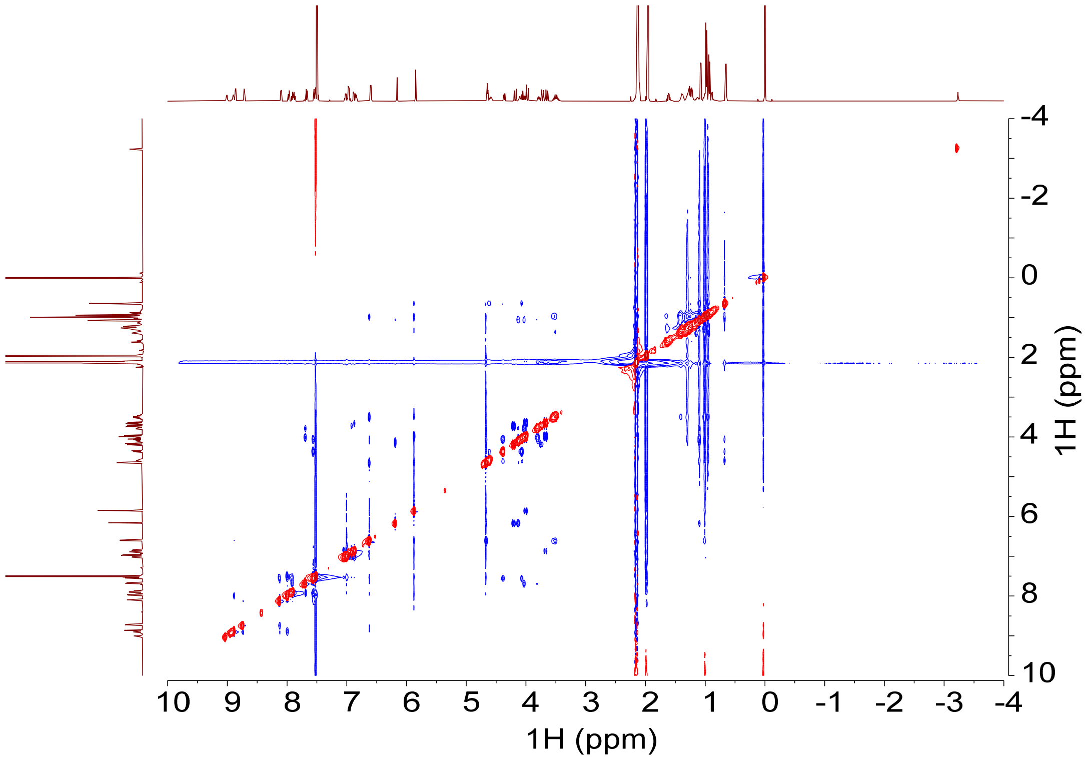

*Fig. S262. ^{1}H-^{1}H ROESY NMR spectrum of (S,S,S,S)-**$H_2$3**·(R,R)-**15** (500 MHz, $CDCl_3/CD_3CN$, 1:1, v/v, 298 K).*

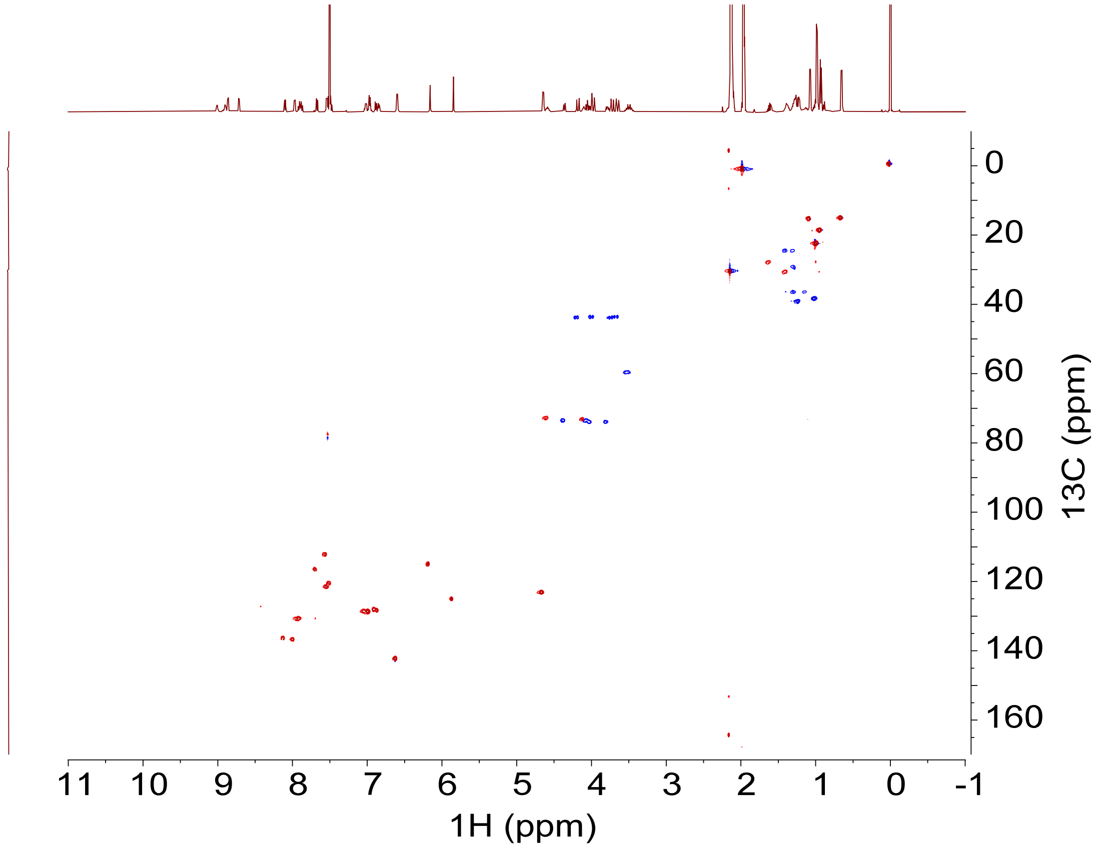

*Fig. S263. ^{1}H-^{13}C HSQC NMR spectrum of (S,S,S,S)-**H$_2$3**·(R,R)-**15** (500 MHz, CDCl$_3$/CD$_3$CN, 1:1, v/v, 298 K).*

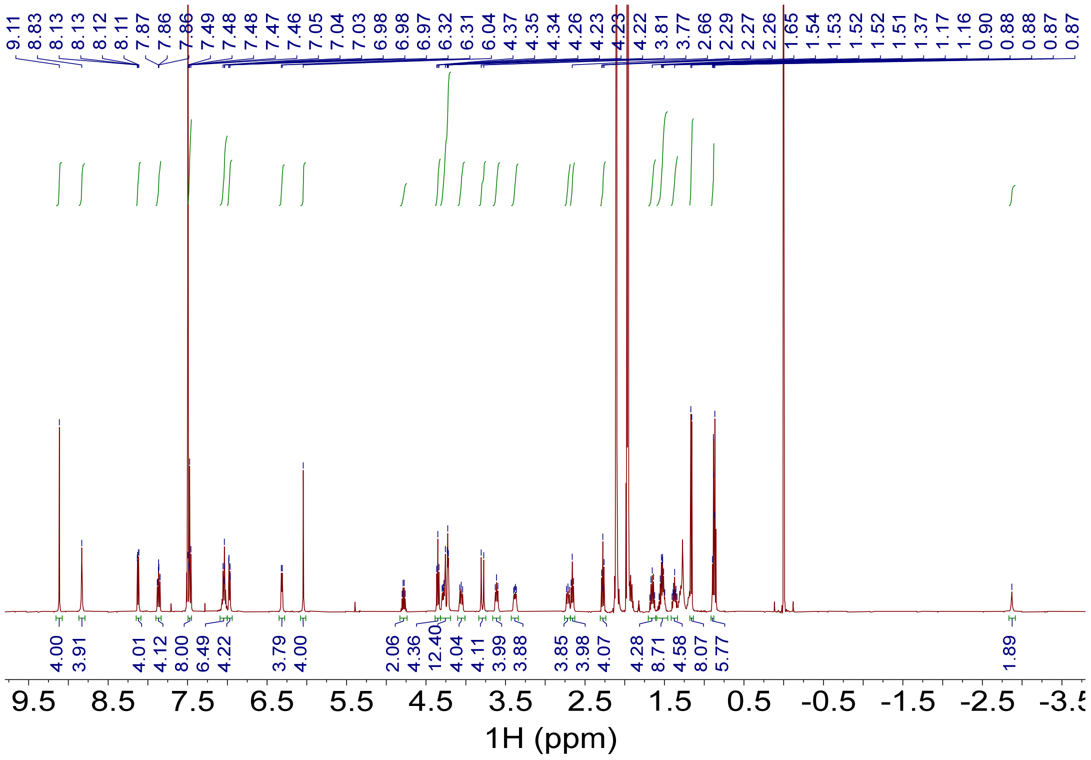

*Fig. S264. ^{1}H NMR spectrum of **H$_2$1**·(R,R)-**16** (500 MHz, CDCl$_3$/CD$_3$CN, 1:1, v/v, 298 K).*

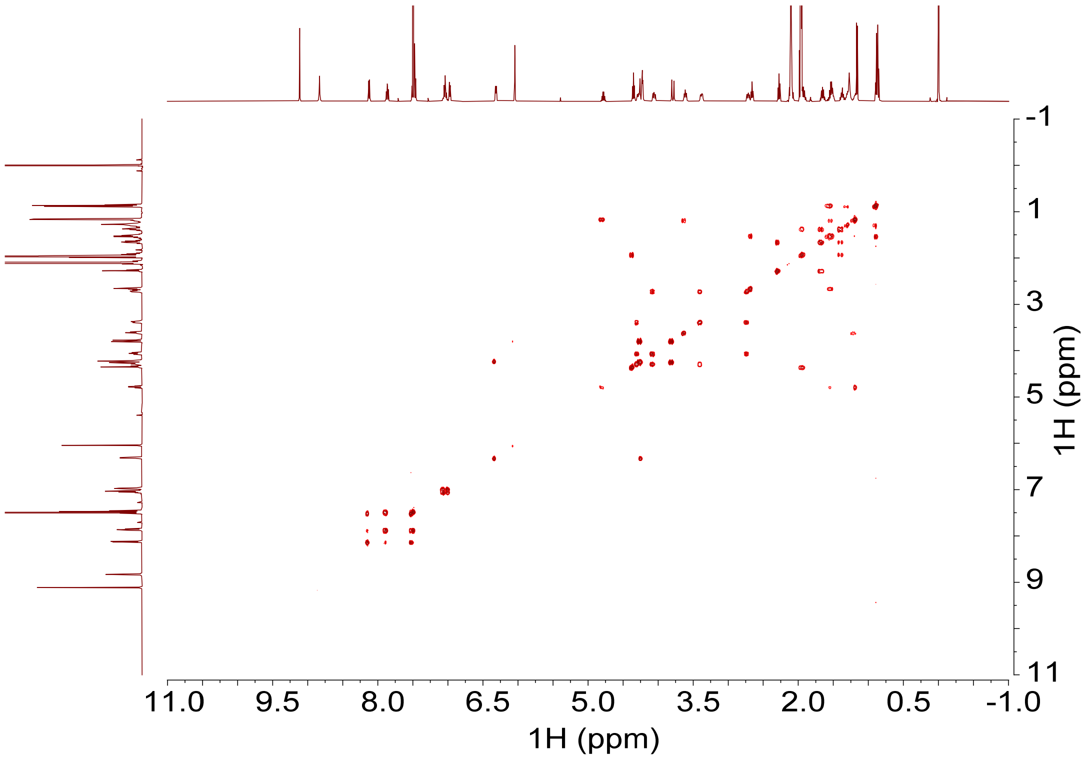

*Fig. S265. ^{1}H-^{1}H COSY NMR spectrum of **$H_2$1·**(R,R)-**16** (500 MHz, $CDCl_3/CD_3CN$, 1:1, v/v, 298 K).*

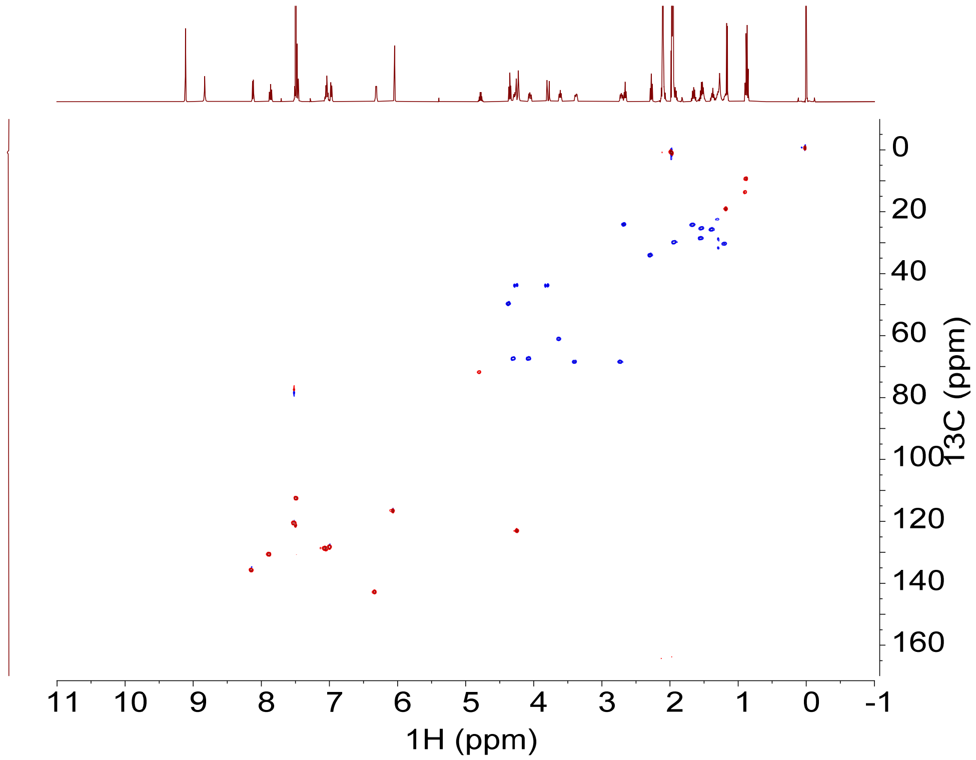

*Fig. S266. ^{1}H-^{13}C HSQC NMR spectrum of **$H_2$1·**(R,R)-**16** (500 MHz, $CDCl_3/CD_3CN$, 1:1, v/v, 298 K).*

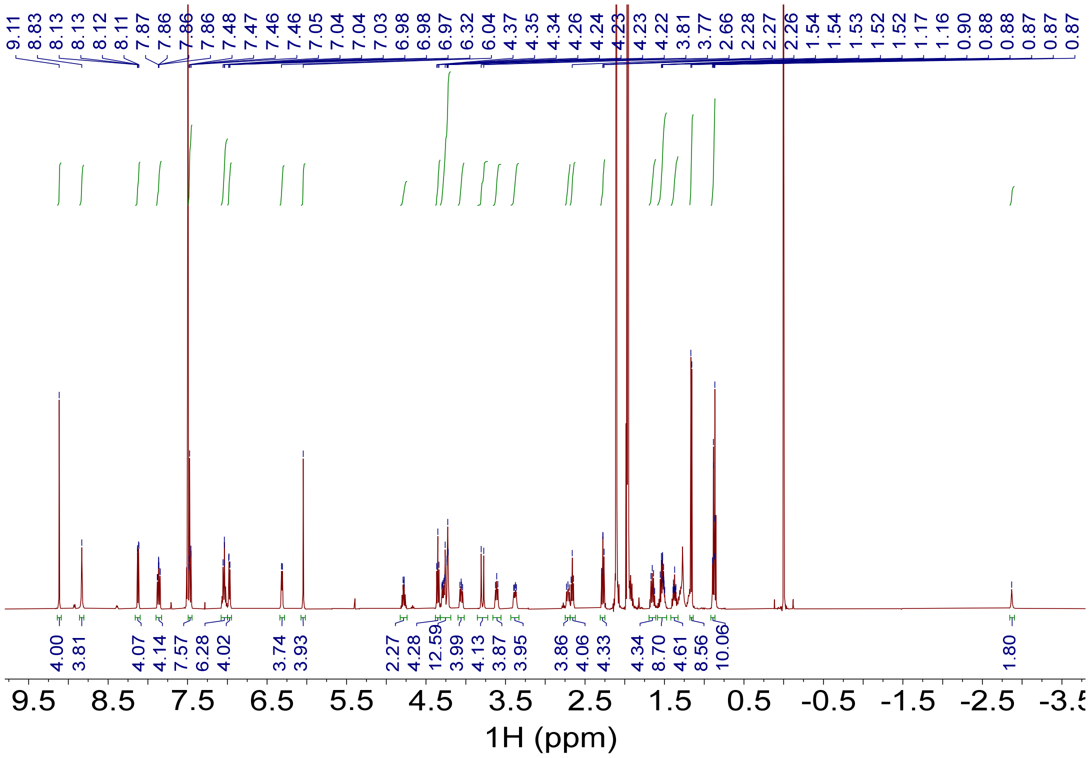

*Fig. S267. ^1H NMR spectrum of **$H_2$1**·(S,S)-**16** (500 MHz, $CDCl_3/CD_3CN$, 1:1, v/v, 298 K).*

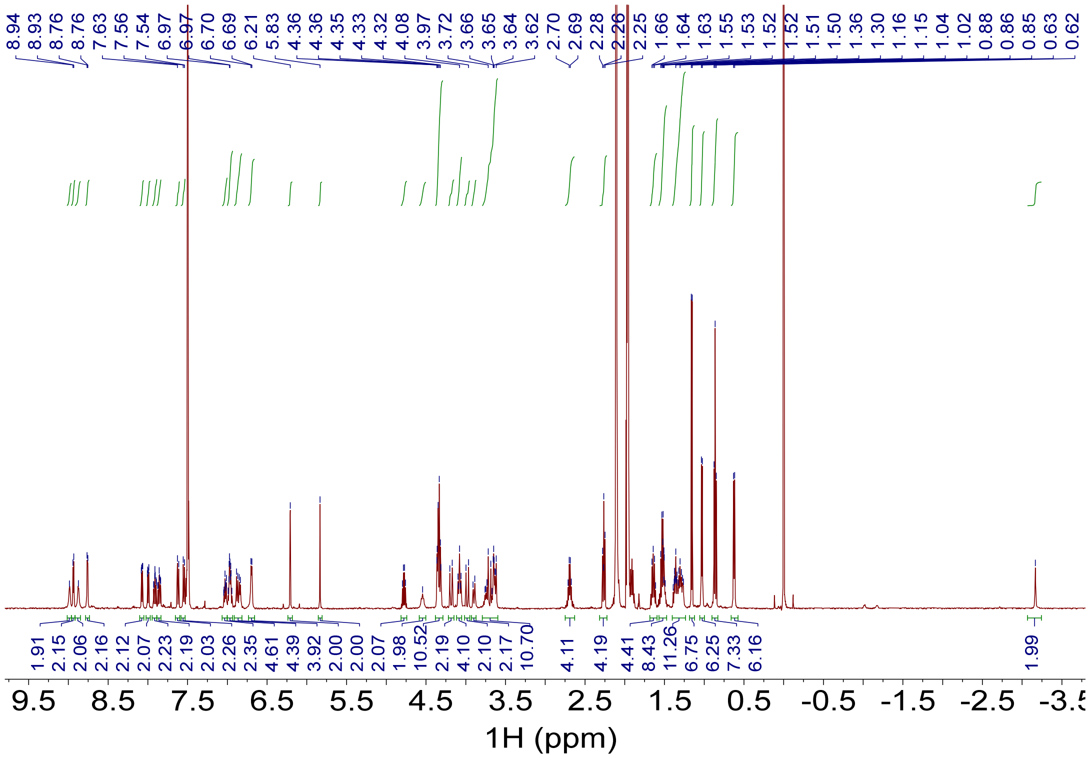

*Fig. S268. ^1H NMR spectrum of (R,R,R,R)-**$H_2$3**·(R,R)-**16** (500 MHz, $CDCl_3/CD_3CN$, 1:1, v/v, 298 K).*

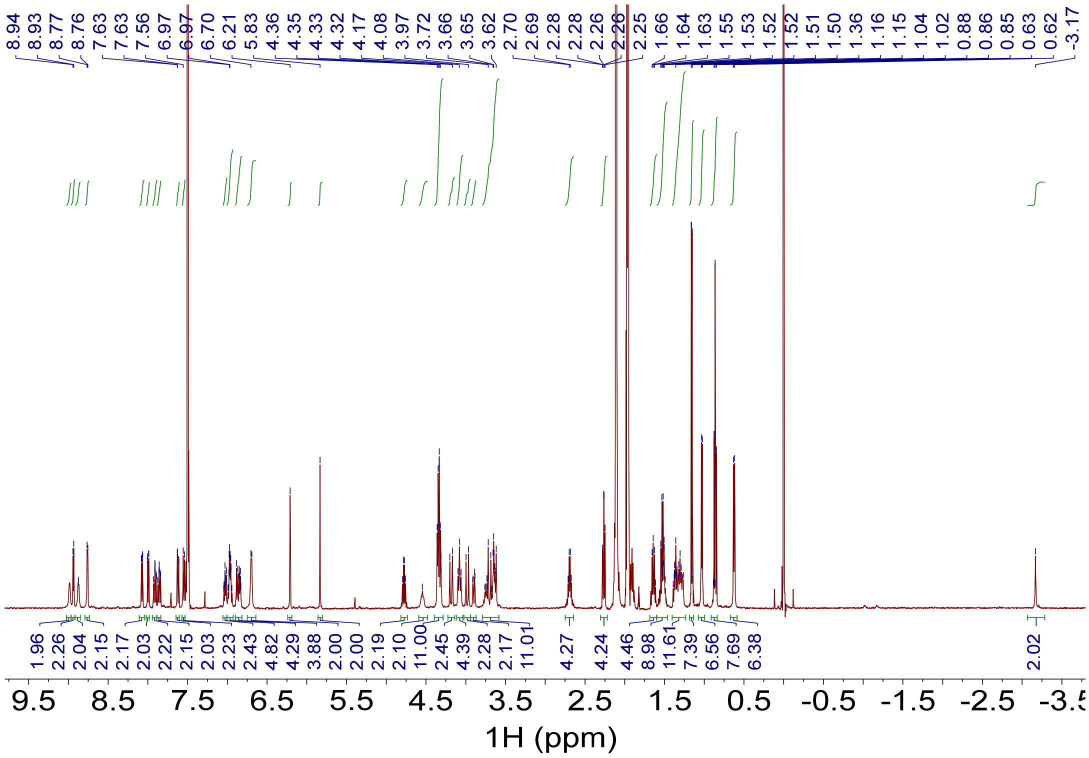

*Fig. S269. ^{1}H NMR spectrum of (S,S,S,S)-**$H_2$3**·(R,R)-**16** (500 MHz, $CDCl_3/CD_3CN$, 1:1, v/v, 298 K).*

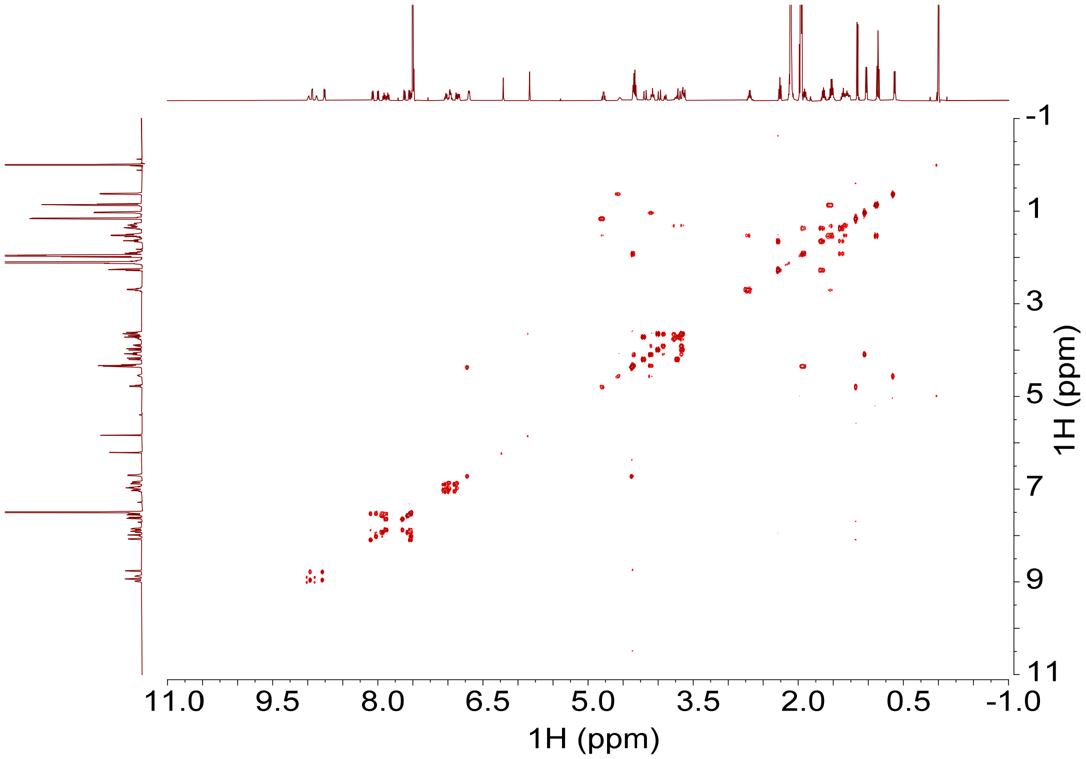

*Fig. S270. ^{1}H-^{1}H COSY NMR spectrum of (S,S,S,S)-**$H_2$3**·(R,R)-**16** (500 MHz, $CDCl_3/CD_3CN$, 1:1, v/v, 298 K).*

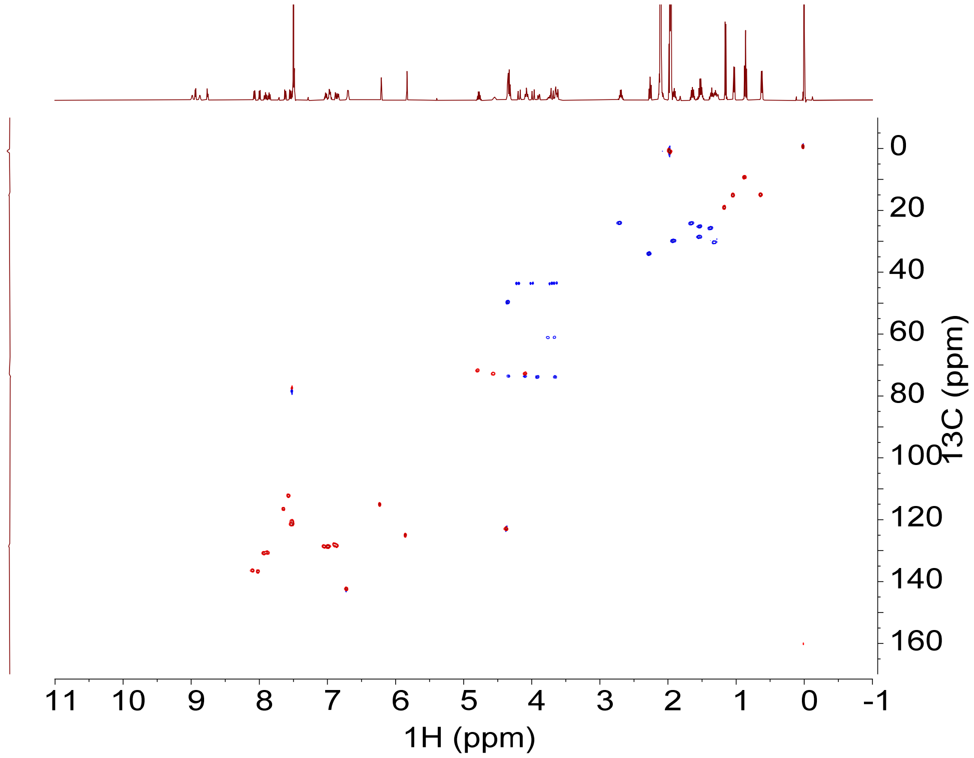

*Fig. S271. ^{1}H-^{13}C HSQC NMR spectrum of (S,S,S,S)-**$H_2$3**·(R,R)-**16** (500 MHz, $CDCl_3$/CD_3CN, 1:1, v/v, 298 K).*

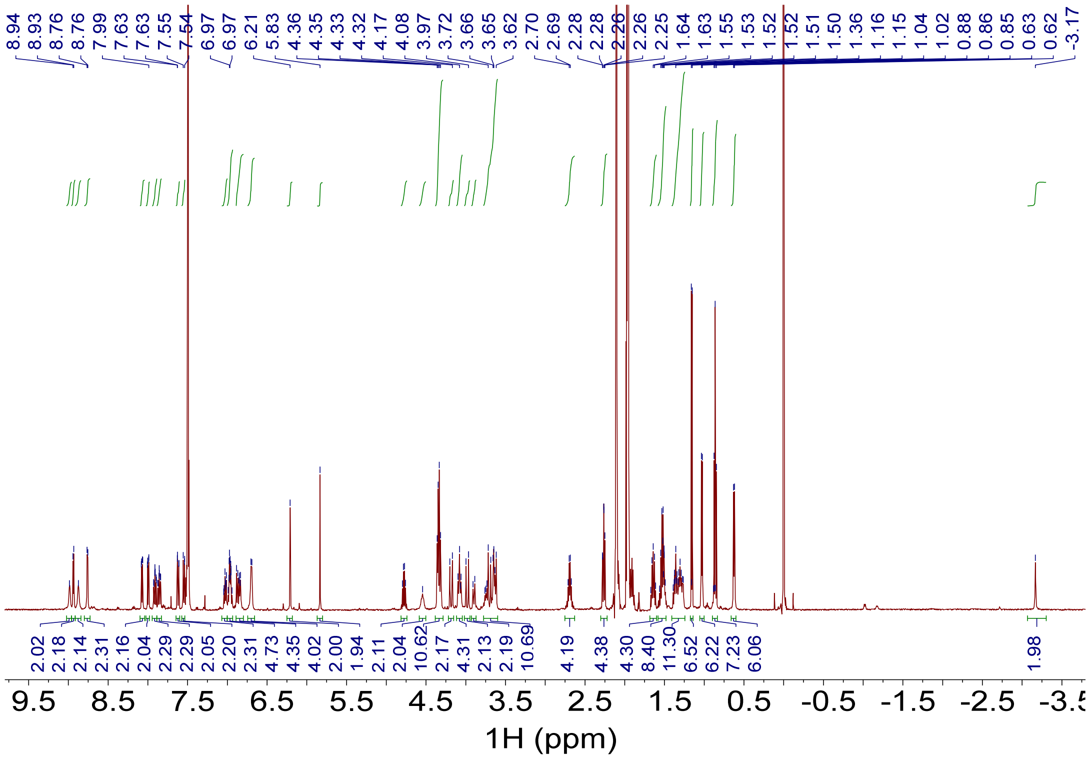

*Fig. S272. ^{1}H NMR spectrum of (R,R,R,R)-**$H_2$3**·(S,S)-**16** (500 MHz, $CDCl_3$/CD_3CN, 1:1, v/v, 298 K).*

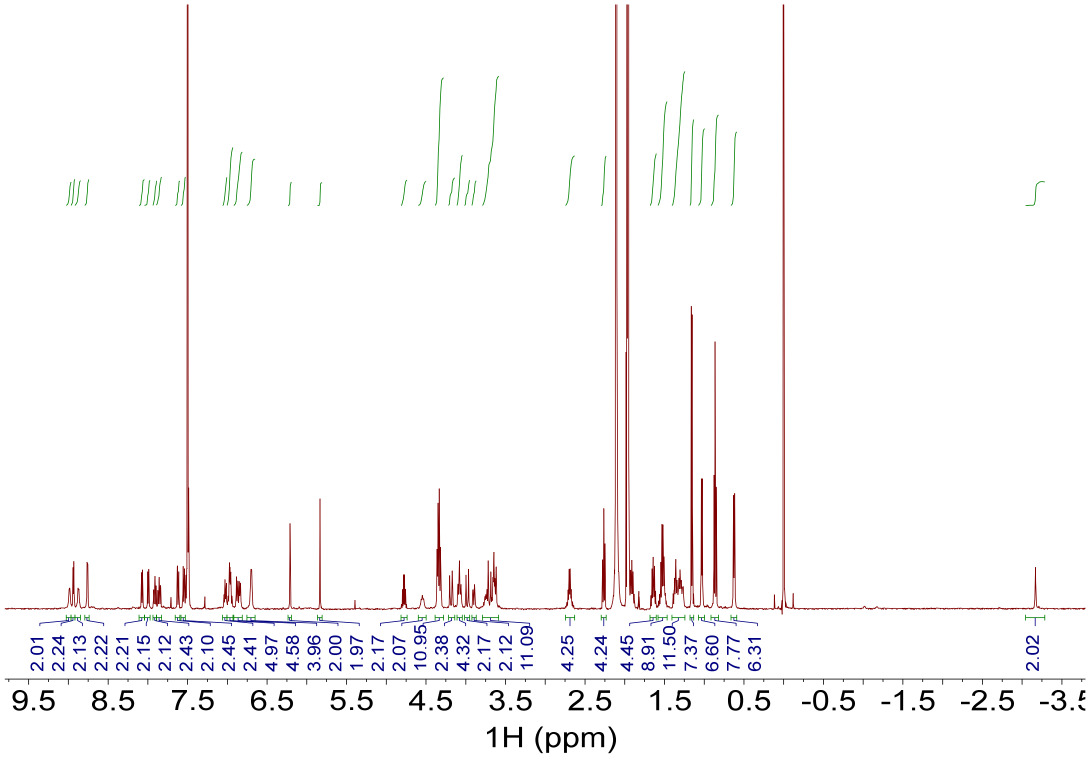

*Fig. S273. ^{1}H NMR spectrum of (S,S,S,S)-**H₂3**·(S,S)-**16** (500 MHz, $CDCl_3/CD_3CN$, 1:1, v/v, 298 K).*

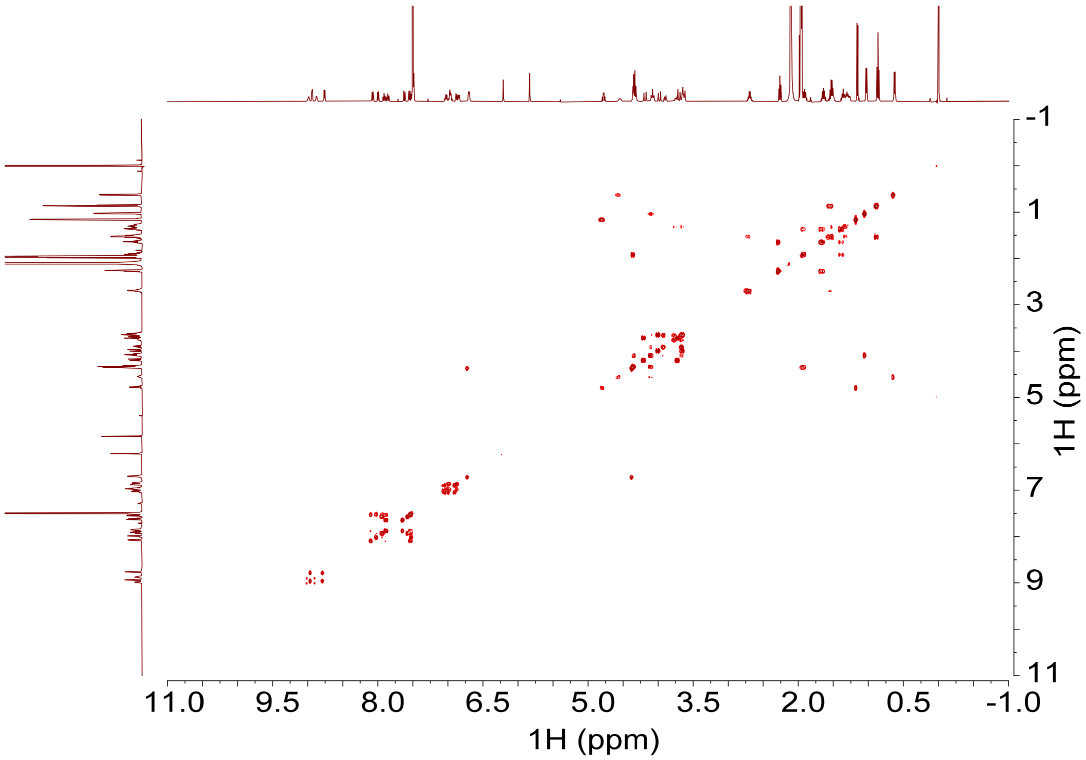

*Fig. S274. ^{1}H-^{1}H COSY NMR spectrum of (S,S,S,S)-**H₂3**·(S,S)-**16** (500 MHz, $CDCl_3/CD_3CN$, 1:1, v/v, 298 K).*

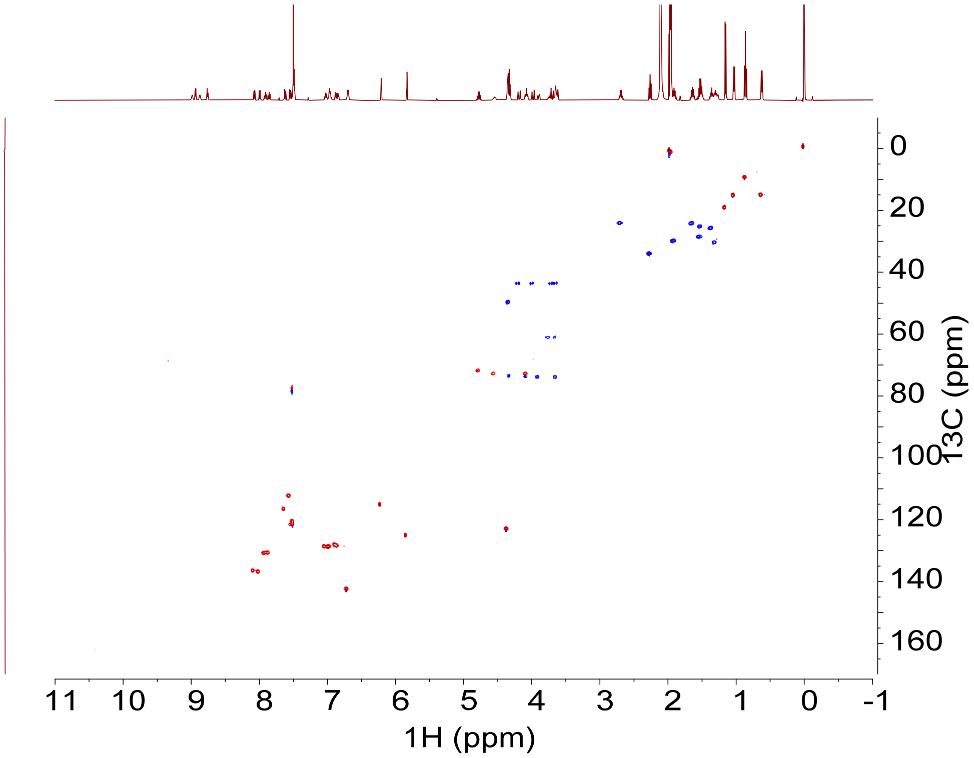

*Fig. S275. ^1H-^{13}C HSQC NMR spectrum of (S,S,S,S)-**$H_2$3**·(S,S)-**16** (500 MHz, $CDCl_3/CD_3CN$, 1:1, v/v, 298 K).*

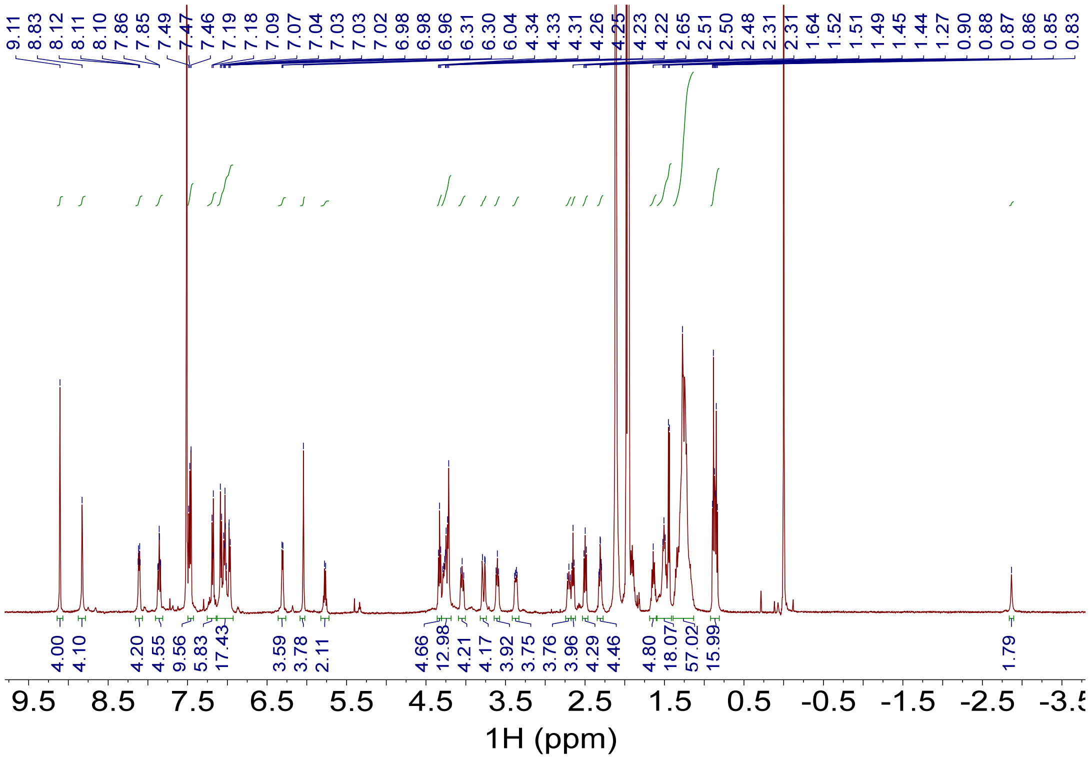

*Fig. S276. ^1H NMR spectrum of **$H_2$1**·(R,R)-**17** (500 MHz, $CDCl_3/CD_3CN$, 1:1, v/v, 298 K).*

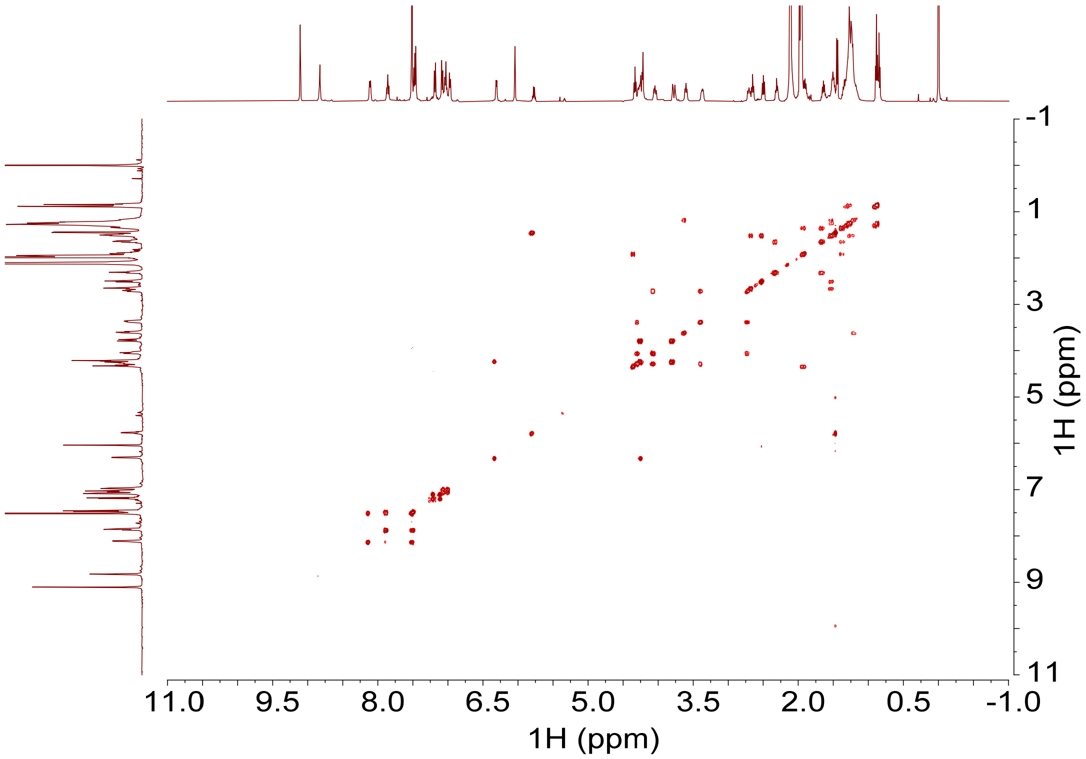

*Fig. S277. ^{1}H-^{1}H COSY NMR spectrum of **H₂1**·(R,R)-**17** (500 MHz, CDCl₃/CD₃CN, 1:1, v/v, 298 K).*

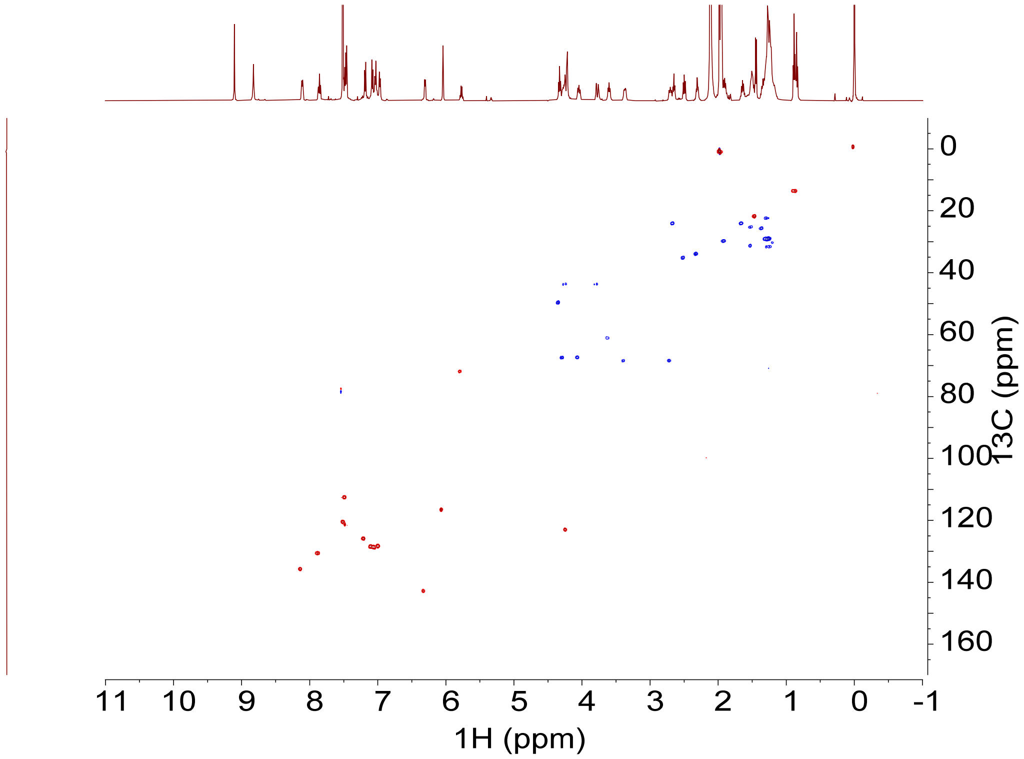

*Fig. S278. ^{1}H-^{13}C HSQC NMR spectrum of **H₂1**·(R,R)-**17** (500 MHz, CDCl₃/CD₃CN, 1:1, v/v, 298 K).*

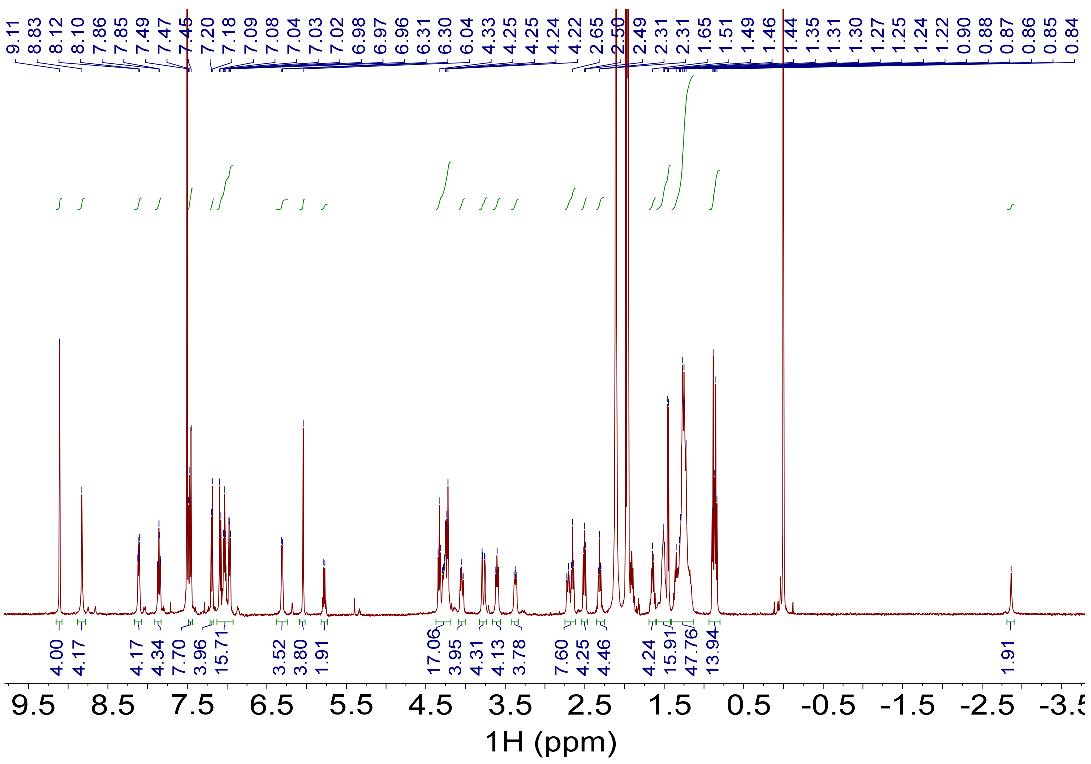

*Fig. S279. ^{1}H NMR spectrum of **$H_2$1**·(S,S)-**17** (500 MHz, $CDCl_3$/CD_3CN, 1:1, v/v, 298 K).*

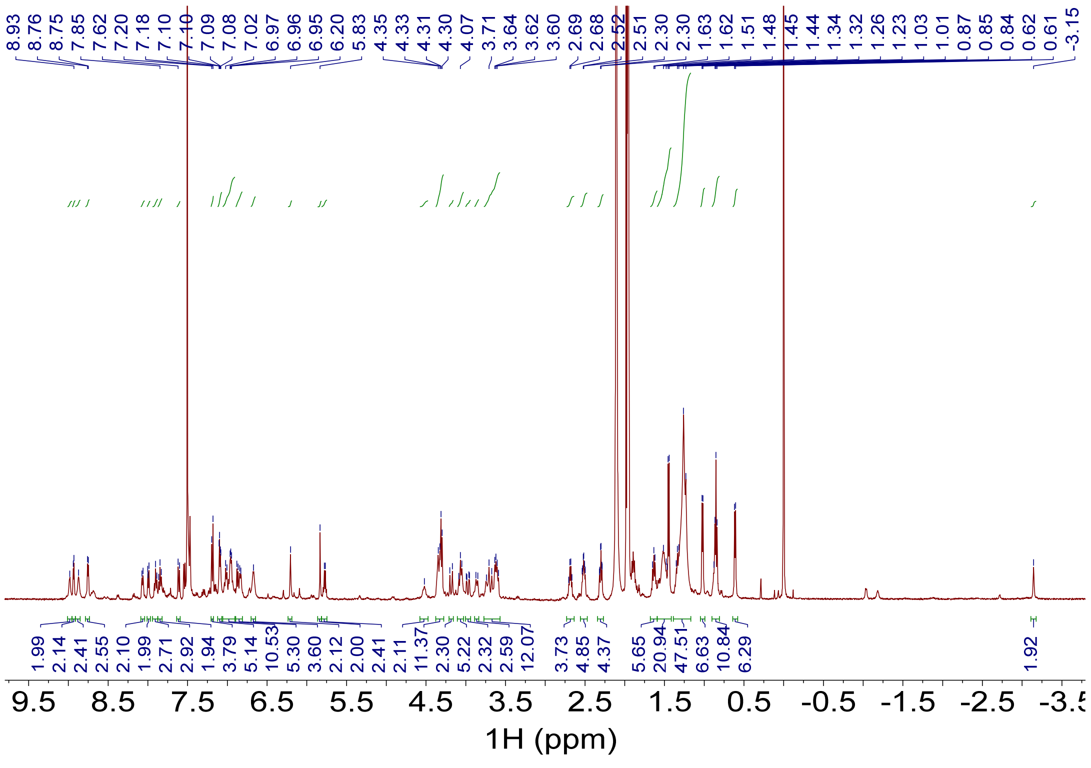

*Fig. S280. ^{1}H NMR spectrum of (R,R,R,R)-**$H_2$3**·(R,R)-**17** (500 MHz, $CDCl_3$/CD_3CN, 1:1, v/v, 298 K).*

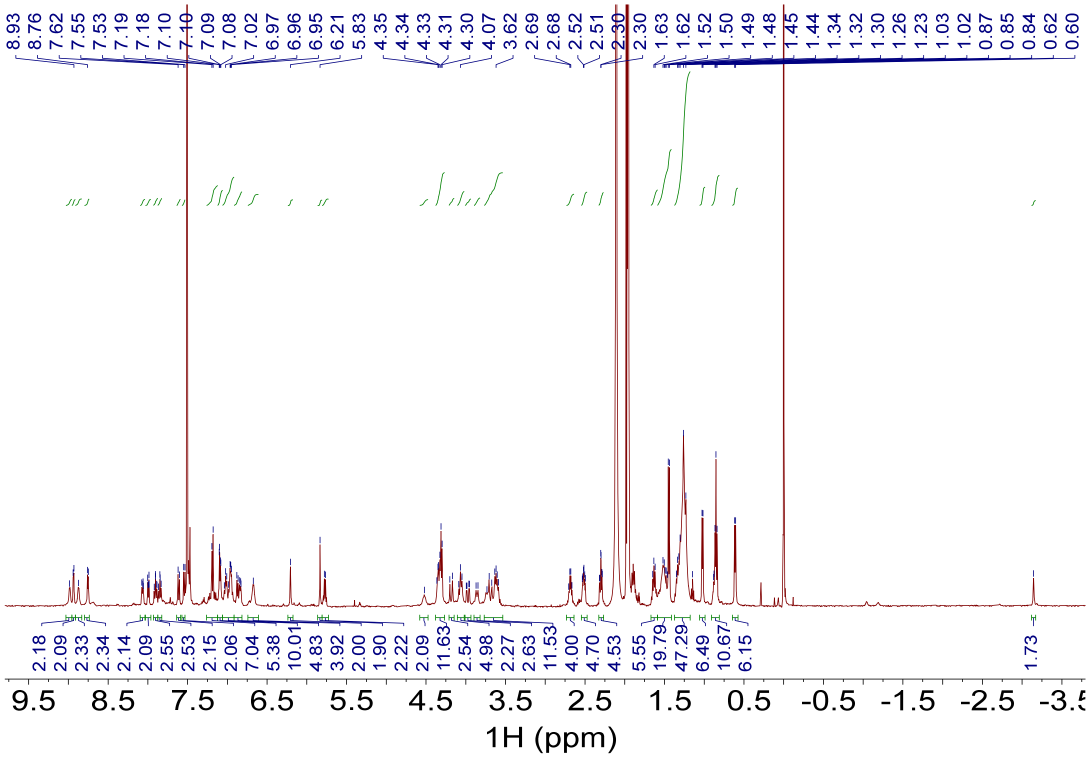

Fig. S281. ^{1}H *NMR spectrum of (S,S,S,S)-**$H_2$3**·(R,R)-**17** (500 MHz,* $CDCl_3/CD_3CN$*, 1:1, v/v, 298 K).*

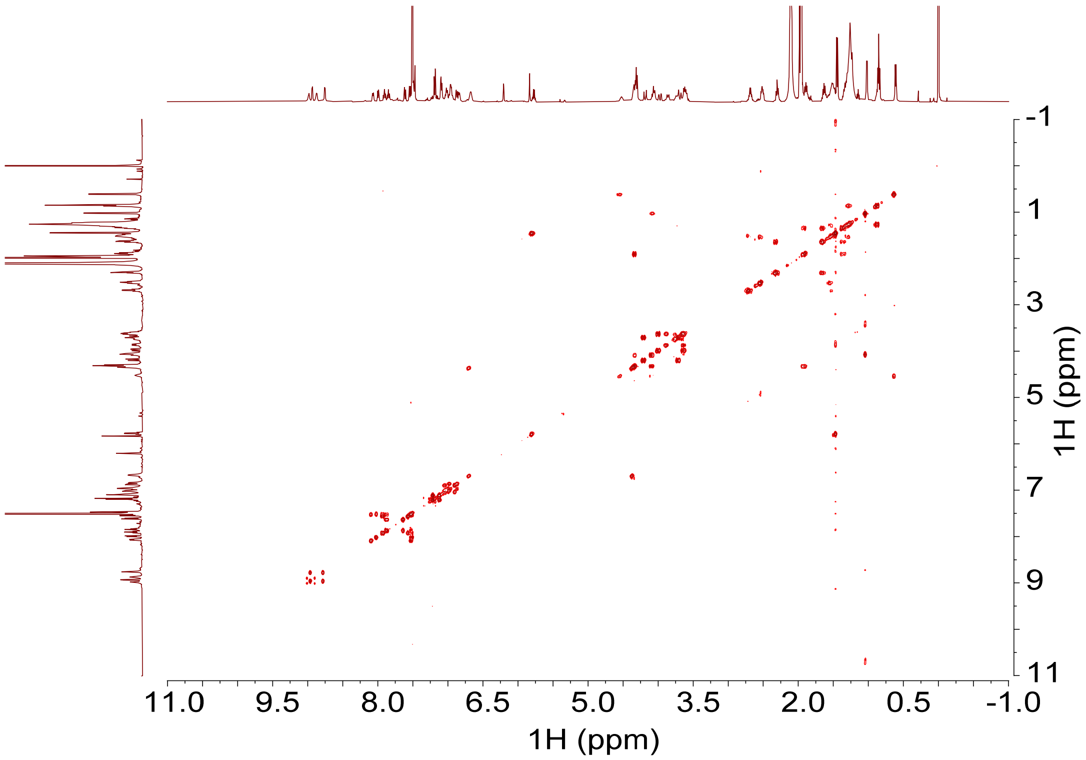

Fig. S282. ^{1}H-^{1}H *COSY NMR spectrum of (S,S,S,S)-**$H_2$3**·(R,R)-**17** (500 MHz,* $CDCl_3/CD_3CN$*, 1:1, v/v, 298 K).*

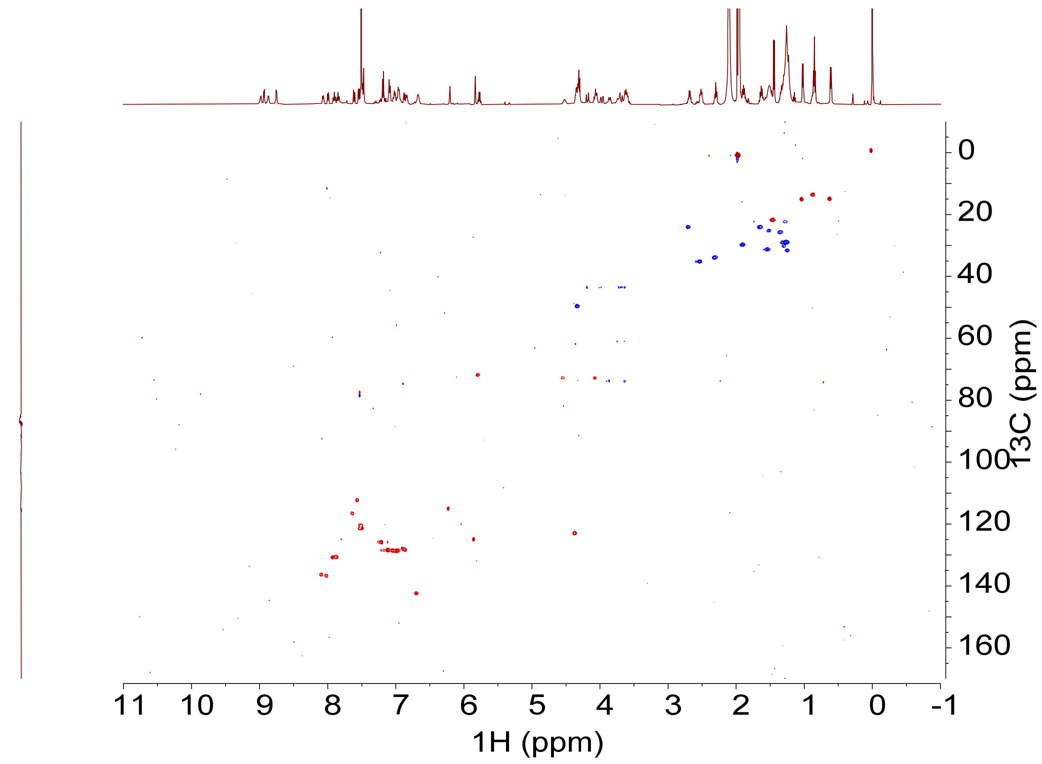

*Fig. S283. ^{1}H-^{13}C HSQC NMR spectrum of (S,S,S,S)-**$H_2$3**·(R,R)-**17** (500 MHz, $CDCl_3$/CD_3CN, 1:1, v/v, 298 K).*

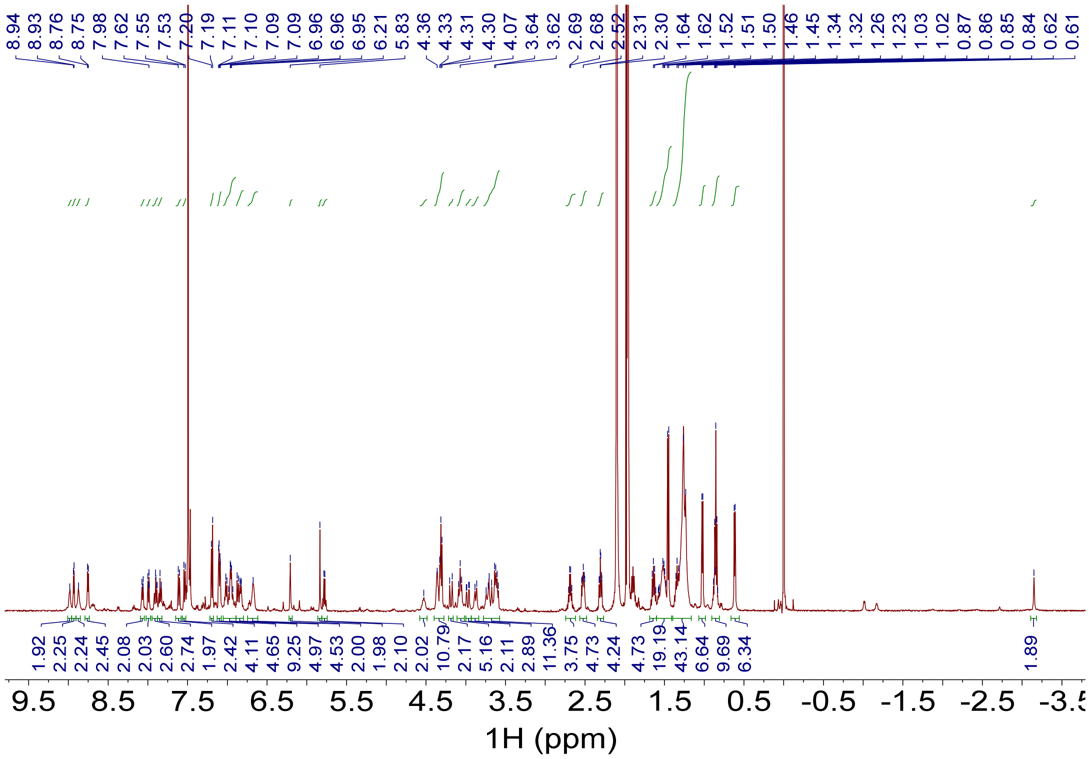

*Fig. S284. ^{1}H NMR spectrum of (R,R,R,R)-**$H_2$3**·(S,S)-**17** (500 MHz, $CDCl_3$/CD_3CN, 1:1, v/v, 298 K).*

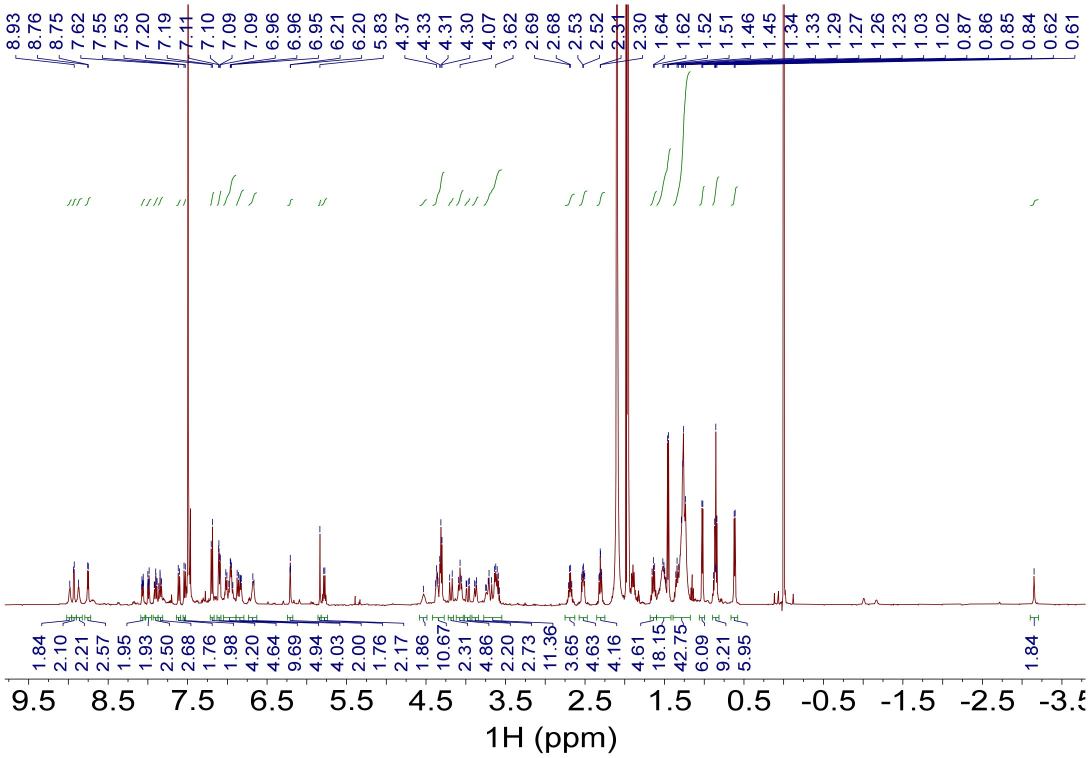

*Fig. S285. ^{1}H NMR spectrum of (S,S,S,S)-**H₂3**·(S,S)-**17** (500 MHz, CDCl₃/CD₃CN, 1:1, v/v, 298 K).*

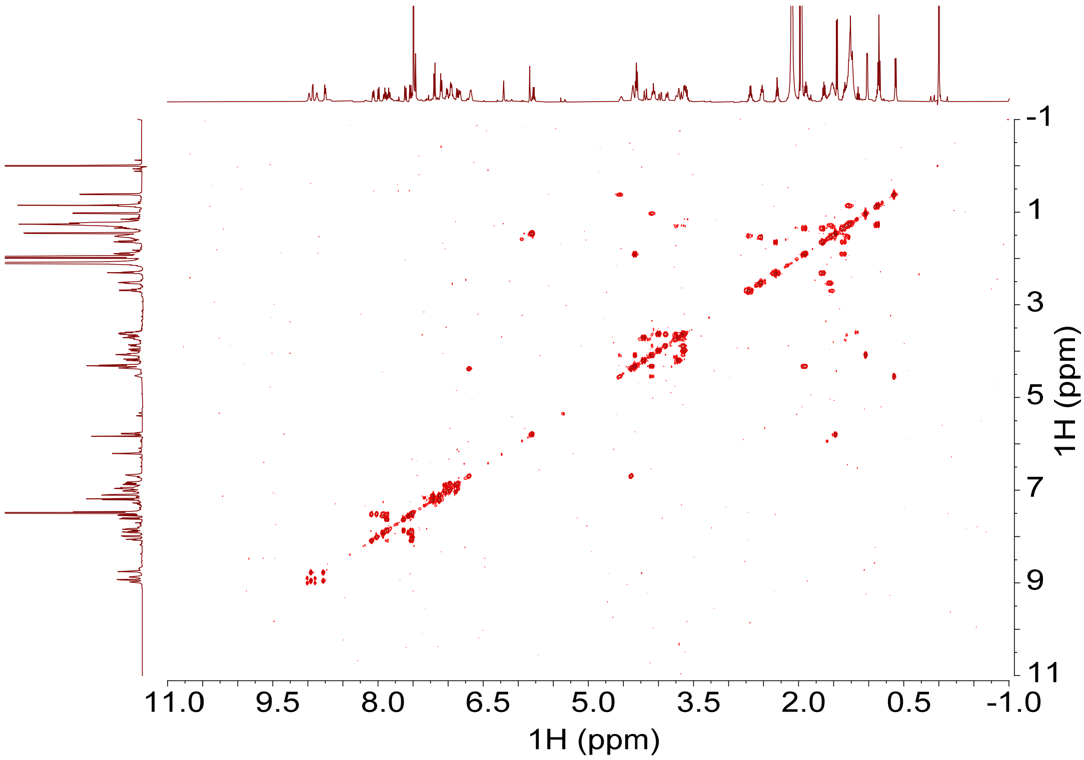

*Fig. S286. ^{1}H-^{1}H COSY NMR spectrum of (S,S,S,S)-**H₂3**·(S,S)-**17** (500 MHz, CDCl₃/CD₃CN, 1:1, v/v, 298 K).*

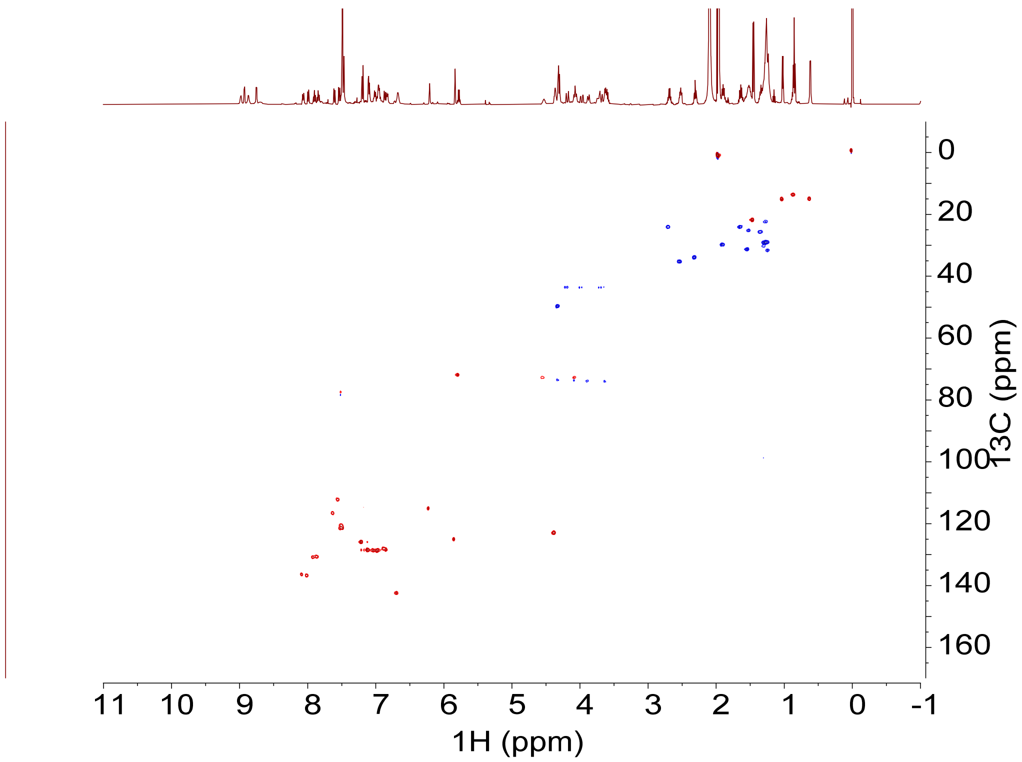

*Fig. S287. ^{1}H-^{13}C HSQC NMR spectrum of (S,S,S,S)-**$H_2$3**·(S,S)-**17** (500 MHz, $CDCl_3$/CD_3CN, 1:1, v/v, 298 K).*

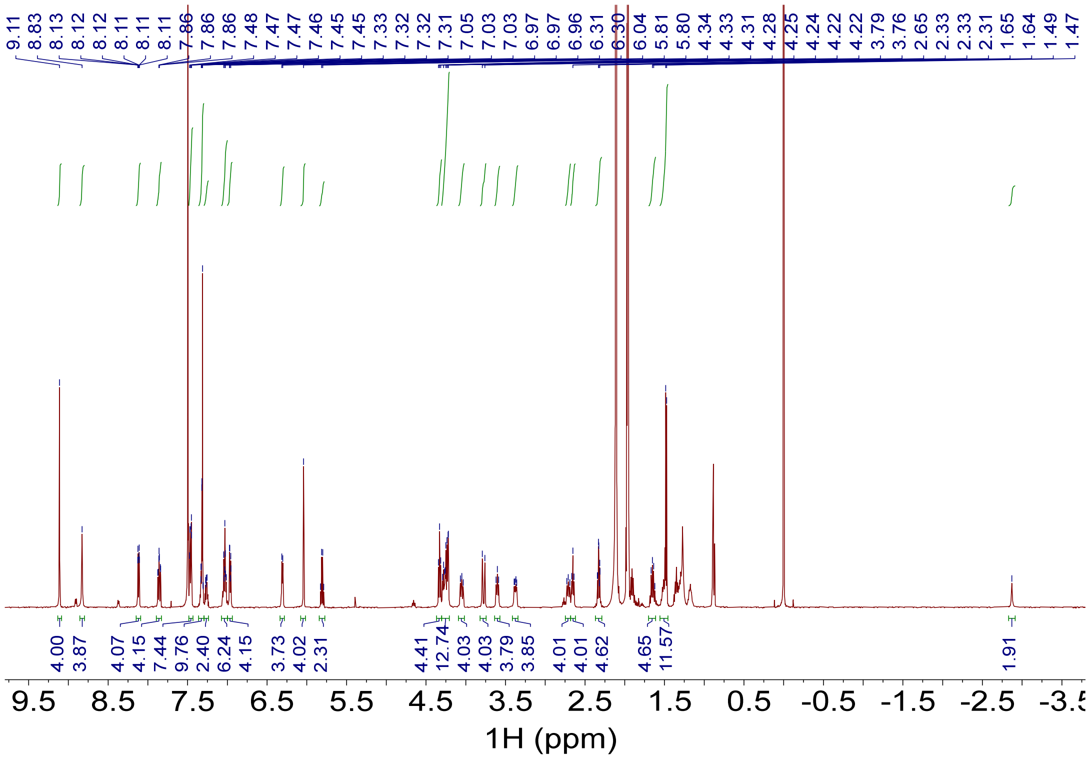

*Fig. S288. ^{1}H NMR spectrum of **$H_2$1**·(R,R)-**18** (500 MHz, $CDCl_3$/CD_3CN, 1:1, v/v, 298 K).*

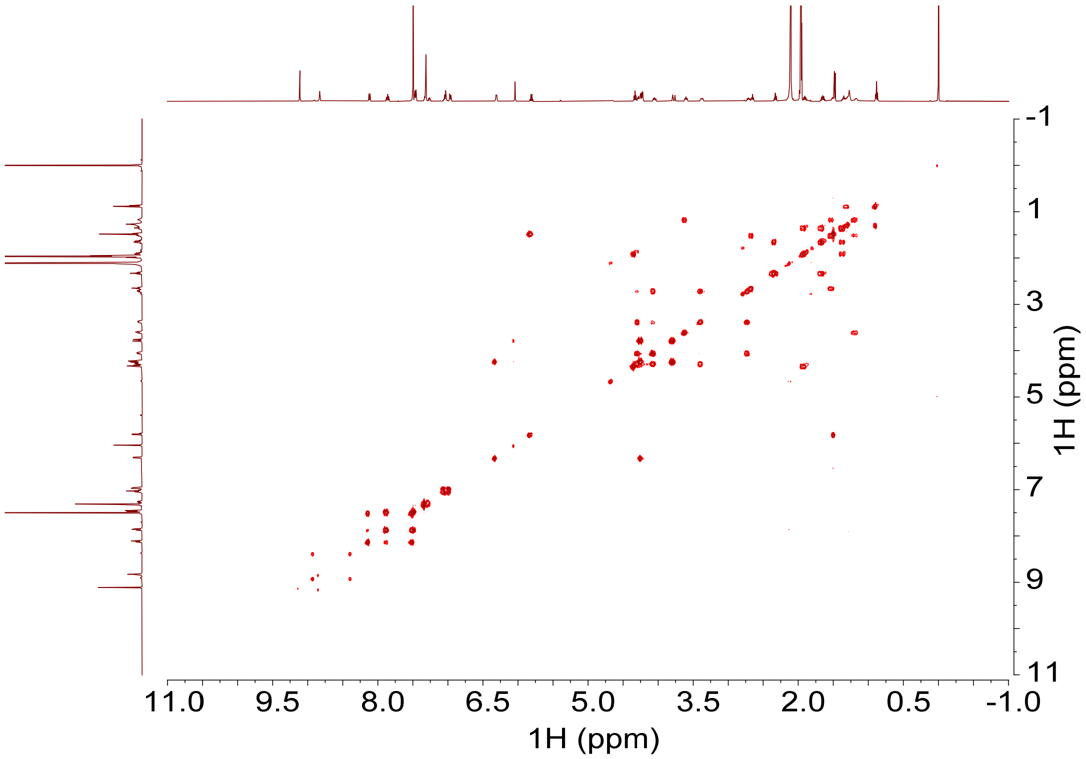

Fig. S289. ^{1}H-^{1}H COSY NMR spectrum of ***H₂1**·(R,R)-**18*** *(500 MHz, $CDCl_3/CD_3CN$, 1:1, v/v, 298 K).*

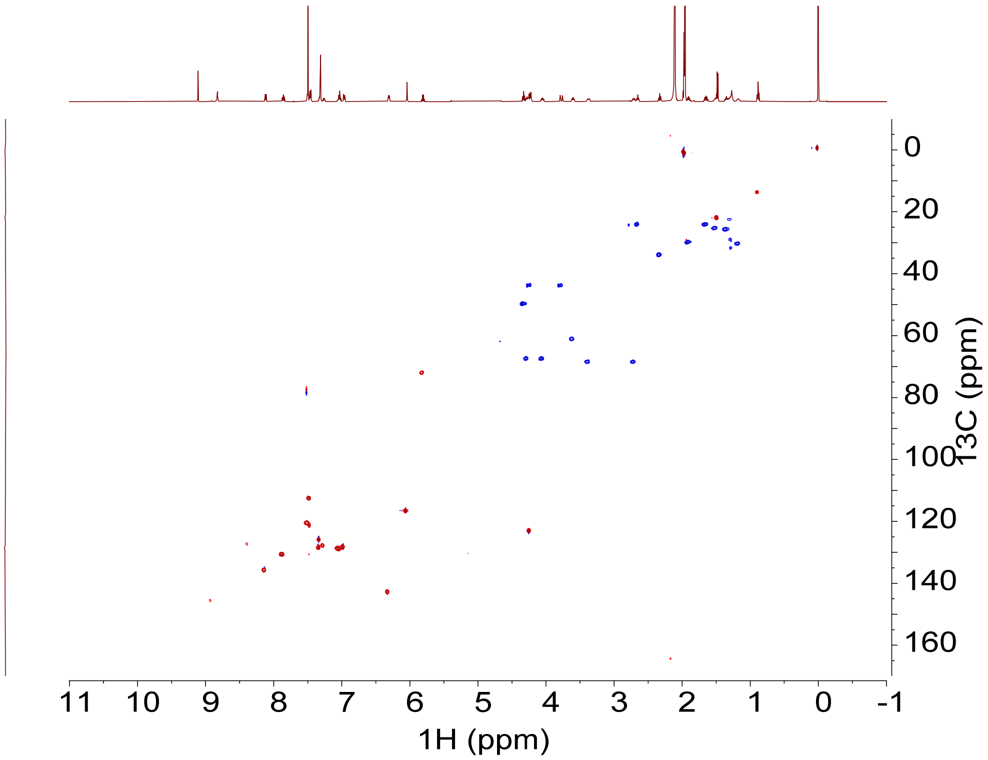

Fig. S290. ^{1}H-^{13}C HSQC NMR spectrum of ***H₂1**·(R,R)-**18*** *(500 MHz, $CDCl_3/CD_3CN$, 1:1, v/v, 298 K).*

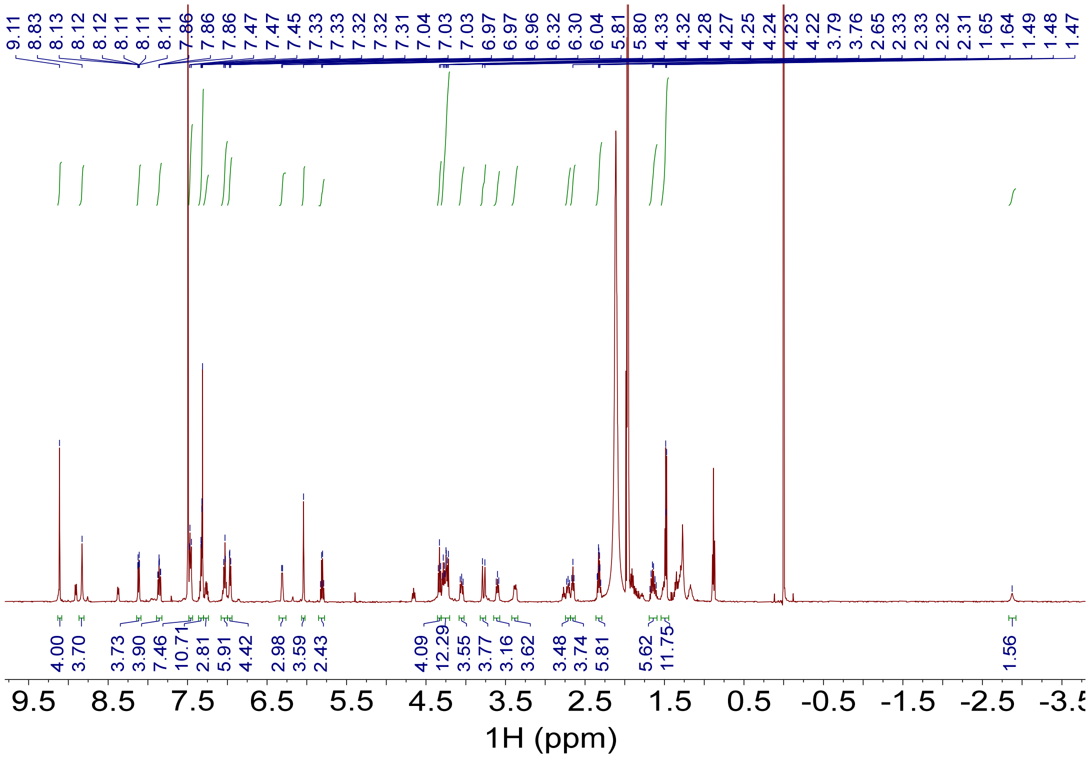

Fig. S291. ¹H NMR spectrum of ***H₂1***·*(S,S)-****18*** *(500 MHz, $CDCl_3/CD_3CN$, 1:1, v/v, 298 K).*

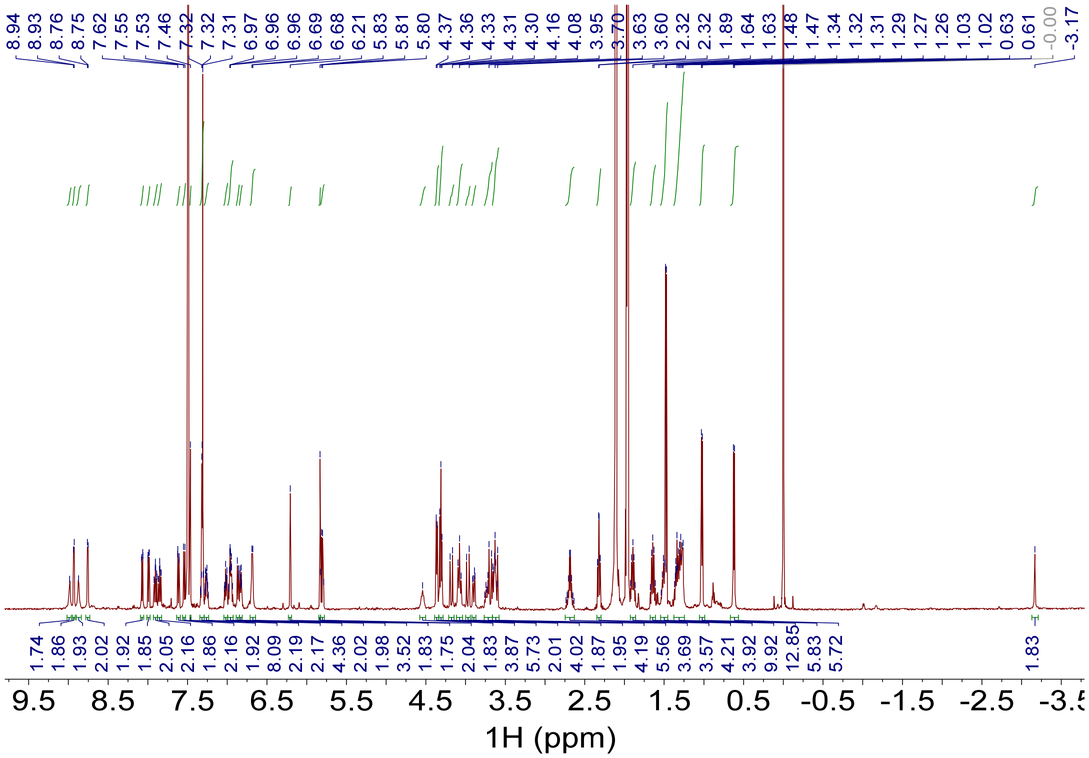

*Fig. S292. ¹H NMR spectrum of (R,R,R,R)-****H₂3***·*(R,R)-****18*** *(500 MHz, $CDCl_3/CD_3CN$, 1:1, v/v, 298 K).*

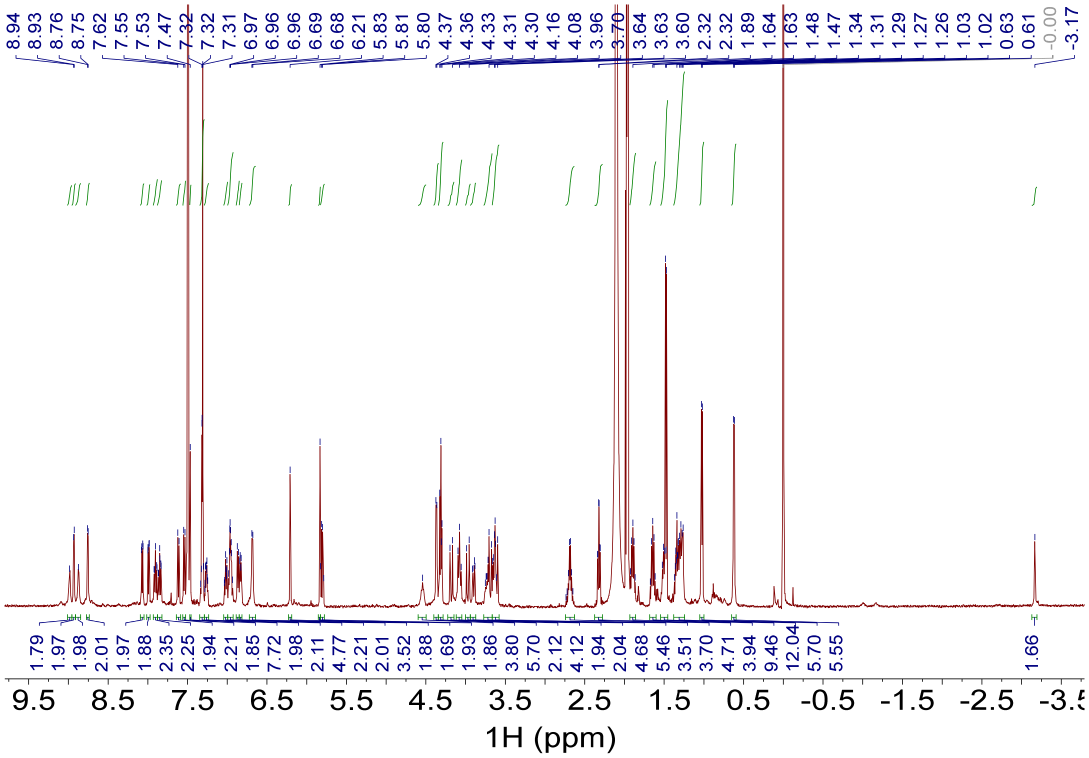

*Fig. S293. ^{1}H NMR spectrum of (S,S,S,S)-**$H_2$3**·(S,S)-**18** (500 MHz, $CDCl_3/CD_3CN$, 1:1, v/v, 298 K).*

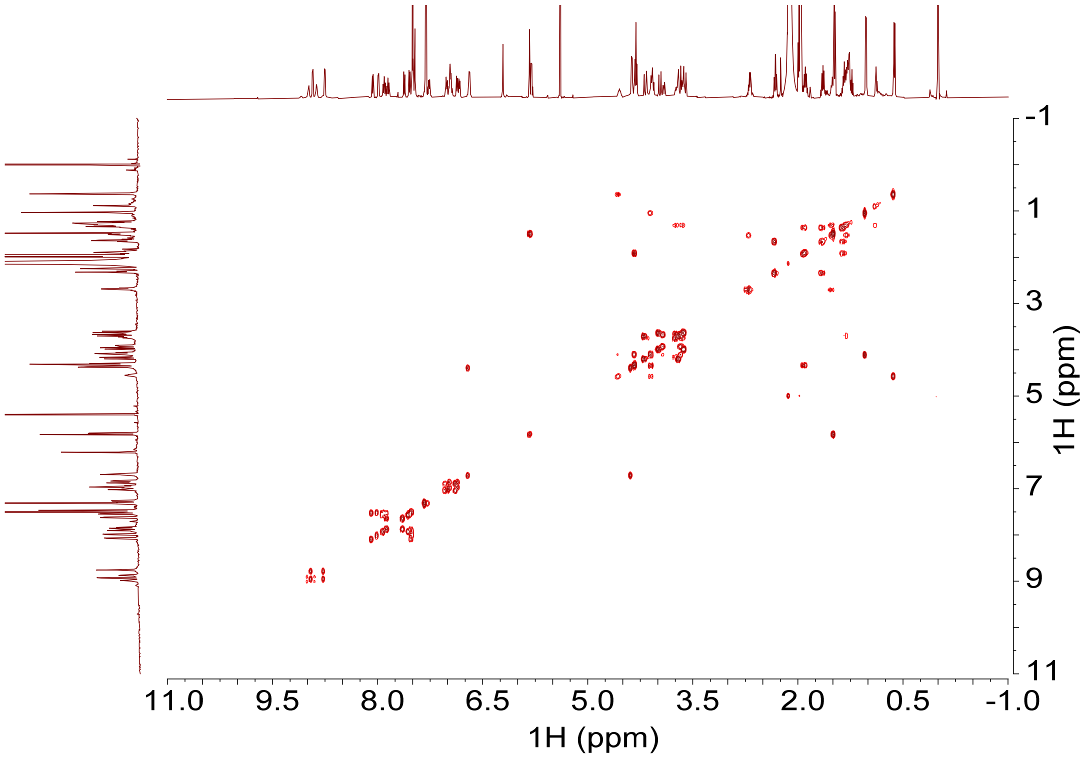

*Fig. S294. ^{1}H-^{1}H COSY NMR spectrum of (S,S,S,S)-**$H_2$3**·(S,S)-**18** (500 MHz, $CDCl_3/CD_3CN$, 1:1, v/v, 298 K).*

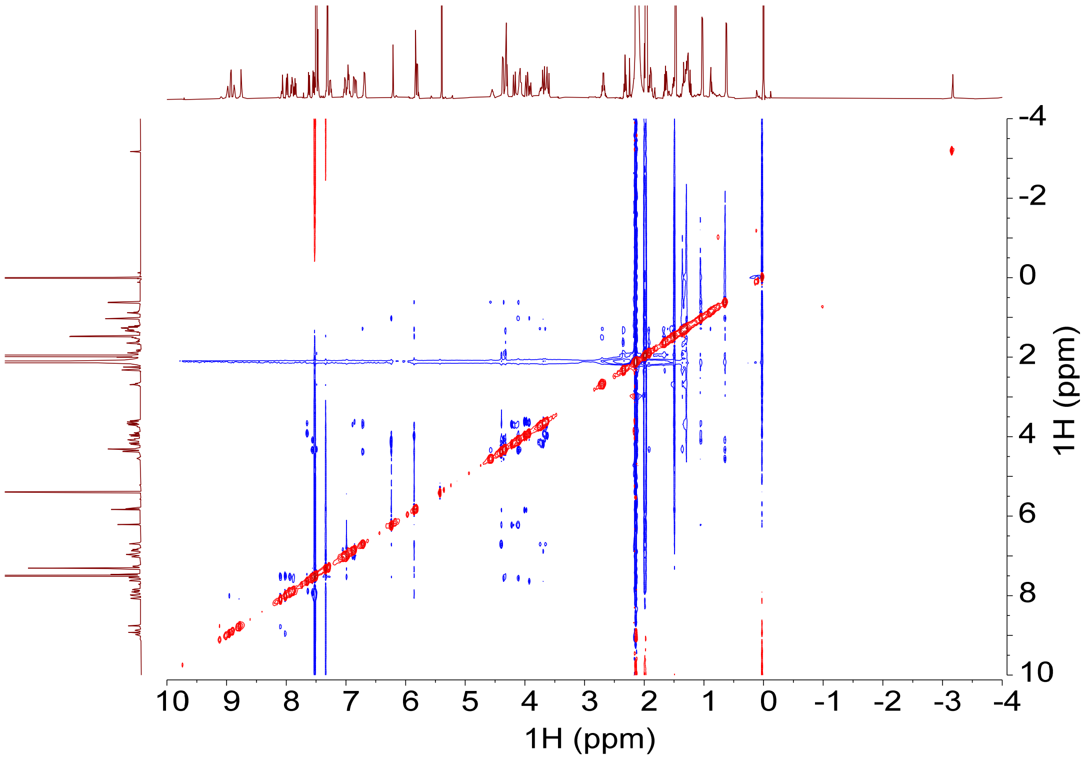

*Fig. S295. ^{1}H-^{1}H ROESY NMR spectrum of (S,S,S,S)-**$H_2$3**·(S,S)-**18** (500 MHz, $CDCl_3$/CD_3CN, 1:1, v/v, 298 K).*

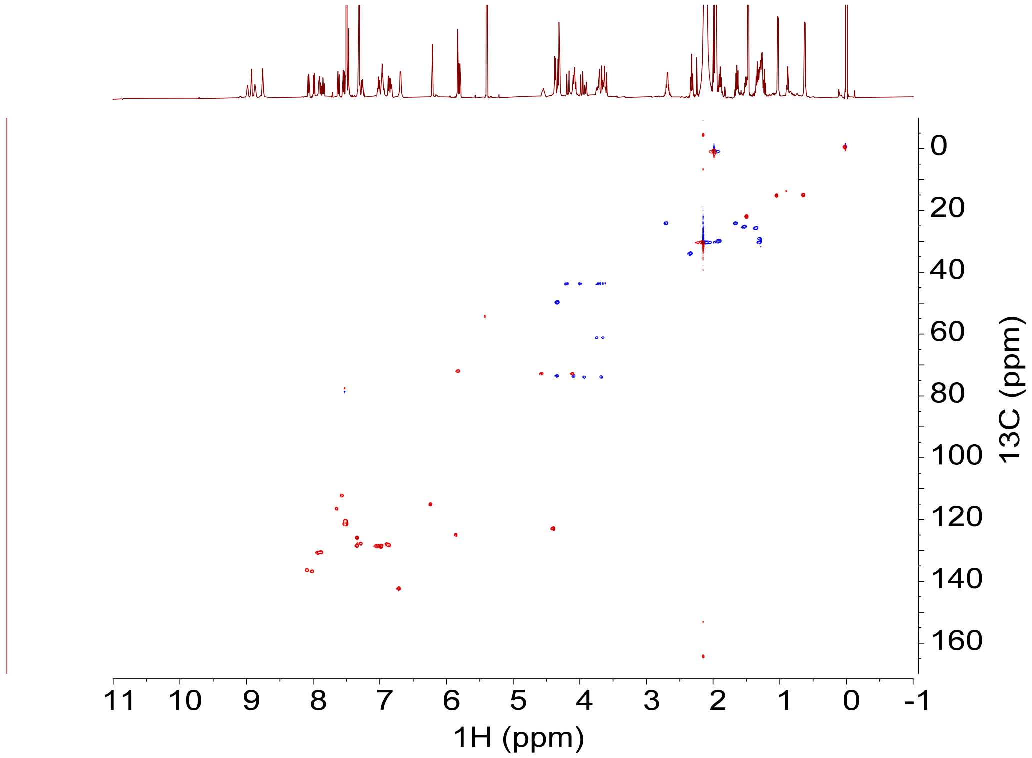

*Fig. S296. ^{1}H-^{13}C HSQC NMR spectrum of (S,S,S,S)-**$H_2$3**·(S,S)-**18** (500 MHz, $CDCl_3$/CD_3CN, 1:1, v/v, 298 K).*

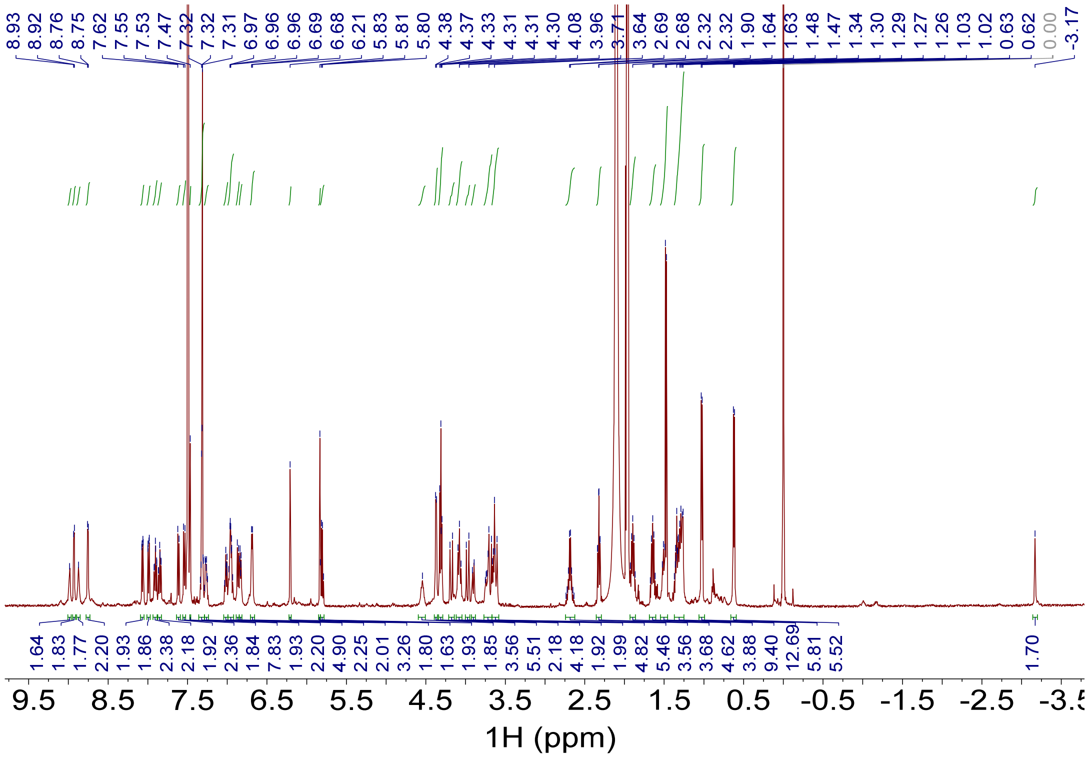

*Fig. S297. ^{1}H NMR spectrum of (R,R,R,R)-**$H_2$3**·(S,S)-**18** (500 MHz, $CDCl_3$/CD_3CN, 1:1, v/v, 298 K).*

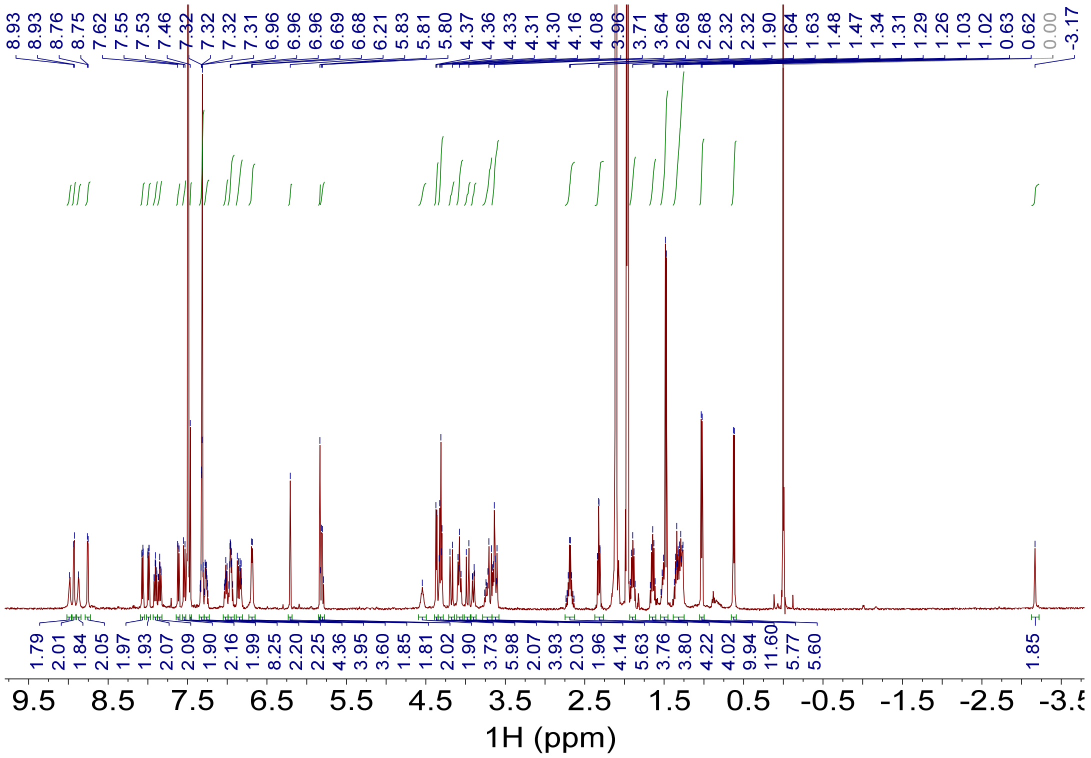

*Fig. S298. ^{1}H NMR spectrum of (S,S,S,S)-**$H_2$3**·(R,R)-**18** (500 MHz, $CDCl_3$/CD_3CN, 1:1, v/v, 298 K).*

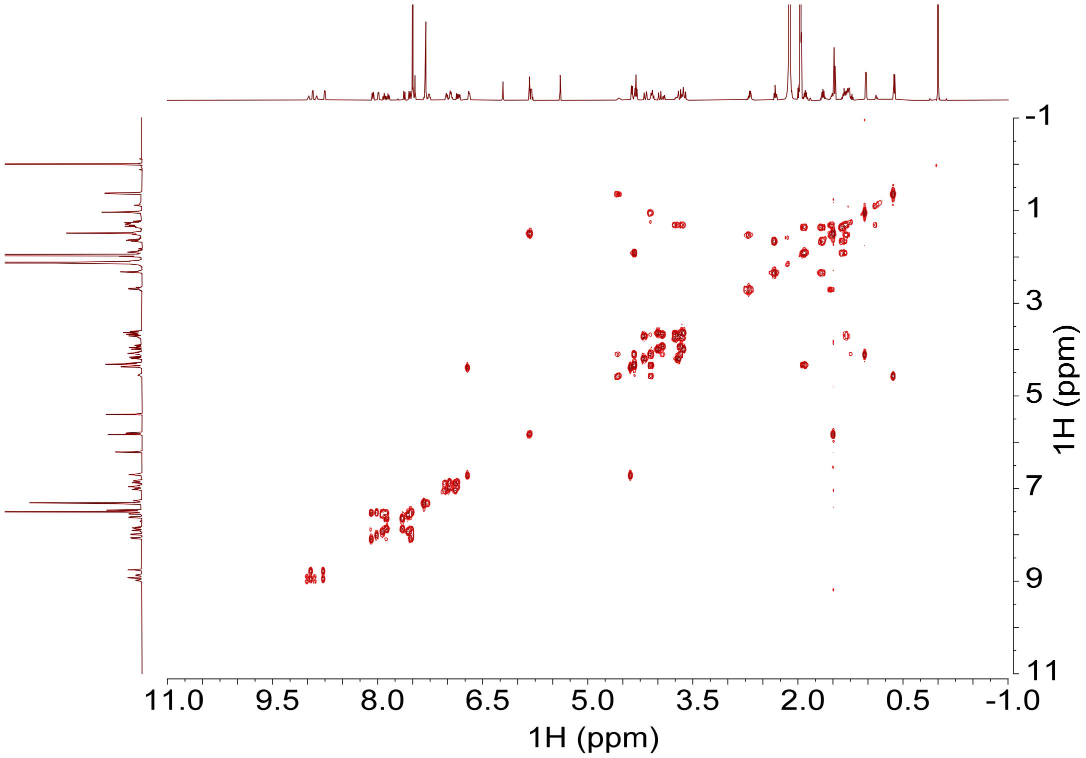

*Fig. S299. ^{1}H-^{1}H COSY NMR spectrum of (S,S,S,S)-**$H_2$3**·(R,R)-**18** (500 MHz, $CDCl_3/CD_3CN$, 1:1, v/v, 298 K).*

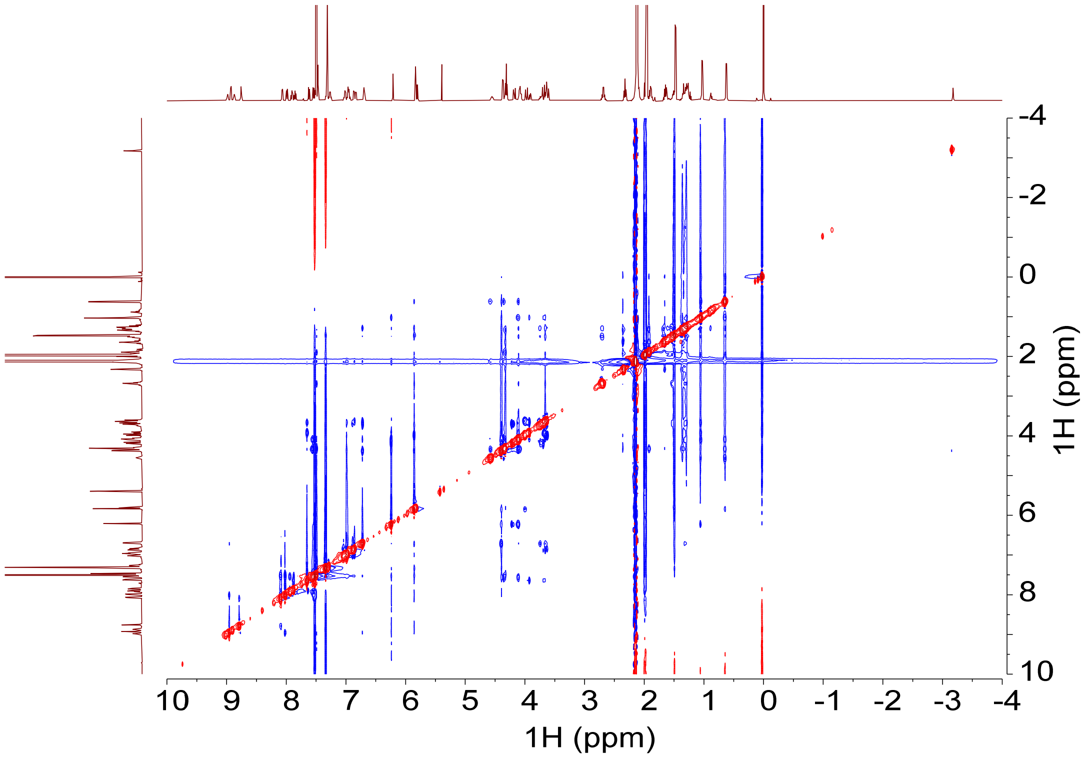

*Fig. S300. ^{1}H-^{1}H ROESY NMR spectrum of (S,S,S,S)-**$H_2$3**·(R,R)-**18** (500 MHz, $CDCl_3/CD_3CN$, 1:1, v/v, 298 K).*

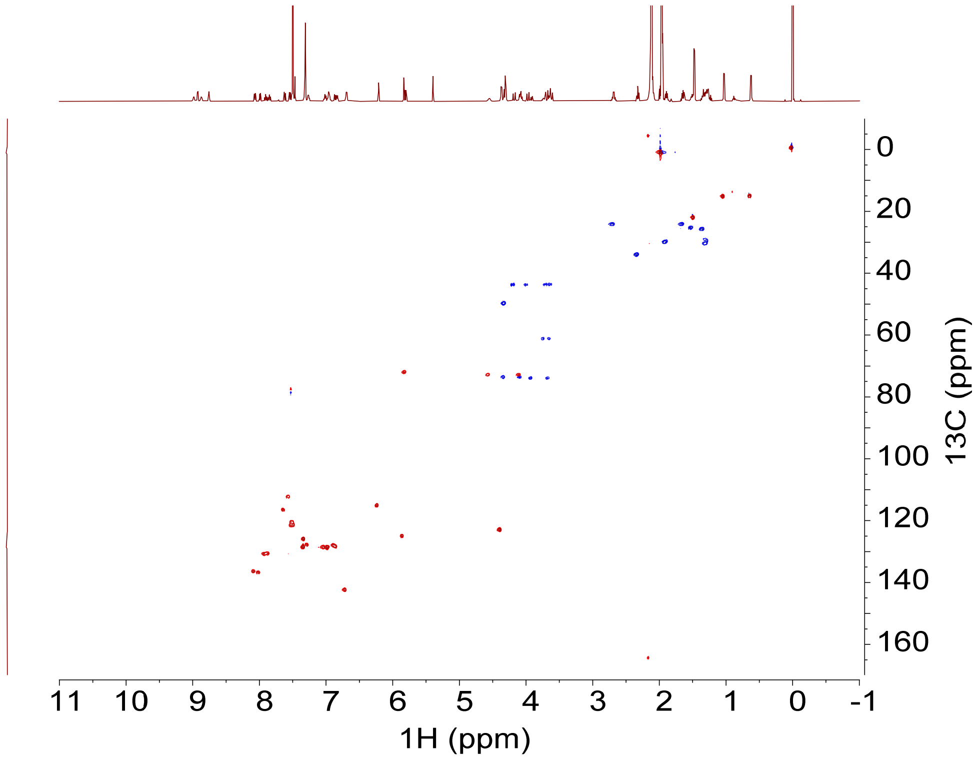

*Fig. S301. ^{1}H-^{13}C HSQC NMR spectrum of (S,S,S,S)-**$H_2$3**·(R,R)-**18** (500 MHz, $CDCl_3$/CD_3CN, 1:1, v/v, 298 K).*

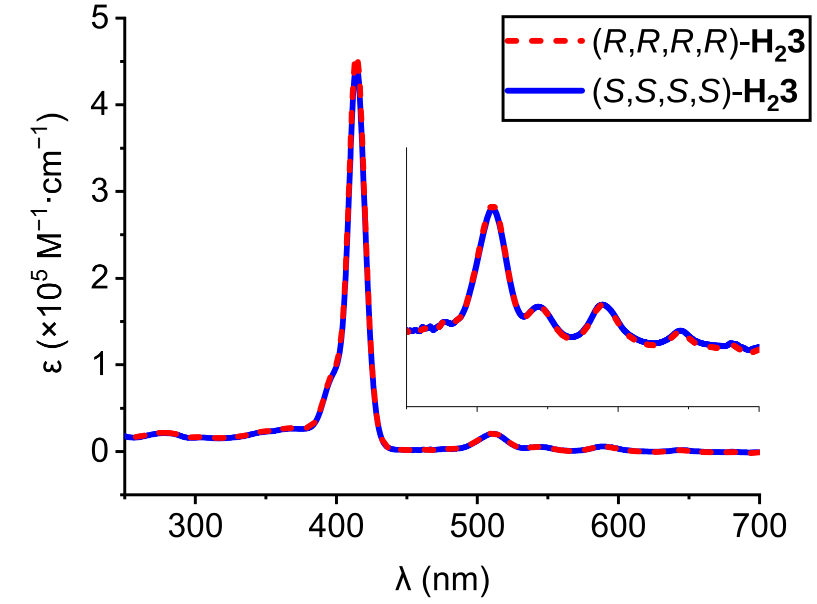

*Fig. S302. UV-vis spectra of both enantiomers of **$H_2$3** (c = 10^{-6} M in $CHCl_3$/CH_3CN, 1:1, v/v, 298 K). Inset: zoom of the Q-band region.*

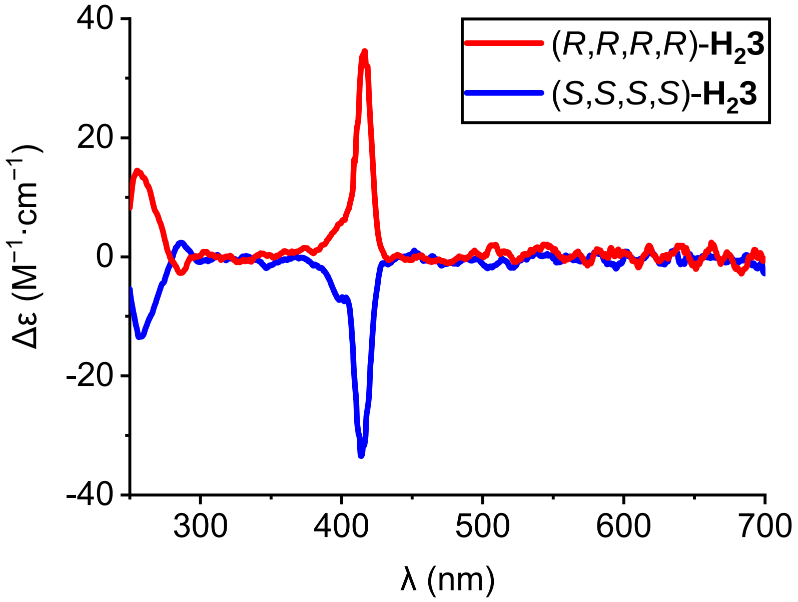

*Fig. S303. ECD spectra of both enantiomers of **$H_2$3** (c = 3 × 10^{-5} M in $CHCl_3$/CH_3CN, 1:1, v/v, 298 K).*

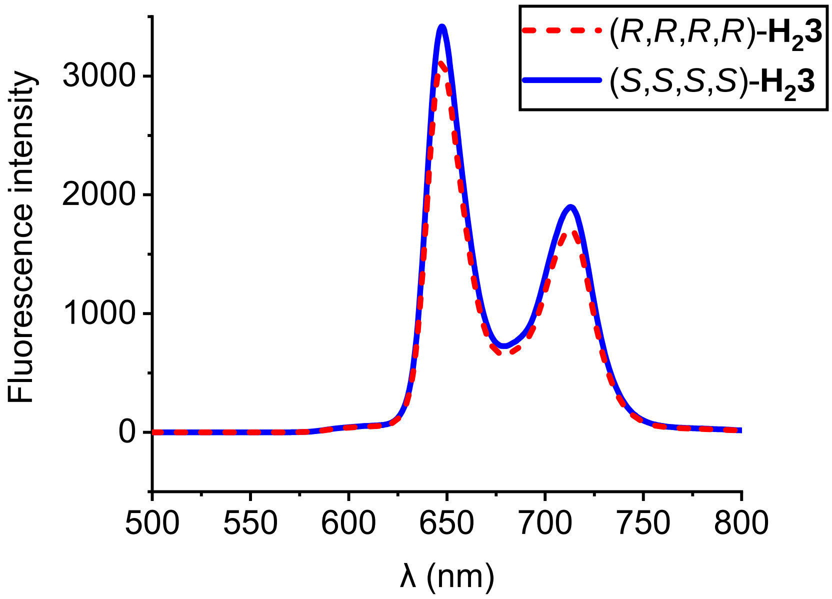

*Fig. S304. Fluorescence spectra of both enantiomers of **$H_2$3** (λ_{ex} = 416 nm, c = 10^{-6} M in $CHCl_3$/CH_3CN, 1:1, v/v, 298 K).*
